# *Eimeria* Species and Genetic Background Influence the Serum Protein Profile of Broilers with Coccidiosis

**DOI:** 10.1371/journal.pone.0014636

**Published:** 2011-01-31

**Authors:** Elizabeth R. Gilbert, Chasity M. Cox, Patricia M. Williams, Audrey P. McElroy, Rami A. Dalloul, W. Keith Ray, Adriana Barri, Derek A. Emmerson, Eric A. Wong, Kenneth E. Webb

**Affiliations:** 1 Department of Animal and Poultry Sciences, Virginia Tech, Blacksburg, Virginia, United States of America; 2 Department of Biochemistry, Virginia Tech, Blacksburg, Virginia, United States of America; 3 Aviagen, Huntsville, Alabama, United States of America; University of Dayton, United States of America

## Abstract

**Background:**

Coccidiosis is an intestinal disease caused by protozoal parasites of the genus *Eimeria*. Despite the advent of anti-coccidial drugs and vaccines, the disease continues to result in substantial annual economic losses to the poultry industry. There is still much unknown about the host response to infection and to date there are no reports of protein profiles in the blood of *Eimeria*-infected animals. The objective of this study was to evaluate the serum proteome of two genetic lines of broiler chickens after infection with one of three species of *Eimeria*.

**Methodology/Principal Findings:**

Birds from lines A and B were either not infected or inoculated with sporulated oocysts from one of the three *Eimeria* strains at 15 d post-hatch. At 21 d (6 d post-infection), whole blood was collected and lesion scoring was performed. Serum was harvested and used for 2-dimensional gel electrophoresis. A total of 1,266 spots were quantitatively assessed by densitometry. Protein spots showing a significant effect of coccidia strain and/or broiler genetic line on density at *P*<0.05−0.01 (250 spots), *P*<0.01−0.001 (248 spots), and *P*<0.001 (314 spots) were excised and analyzed by matrix-assisted laser desorption/ionization tandem time-of-flight mass spectrometry. Proteins were identified in 172 spots. A total of 46 different proteins were identified. Of the spots with a corresponding protein identification, 57 showed a main effect of coccidia infection and/or 2-way interaction of coccidia infection×broiler genetic line at *P*<0.001.

**Conclusions/Significance:**

Several of the metabolic enzymes identified in this study are potential candidates for early diagnostic markers of *E. acervulina* infection including malate dehydrogenase 2, NADH dehydrogenase 1 alpha subcomplex 9, and an ATP synthase. These proteins were detected only in Line A birds that were inoculated with *E. acervulina*. Results from this study provide a basic framework for future research aimed at uncovering the complex biochemical mechanisms involved in host response to *Eimeria* infection and in identifying molecular targets for diagnostic screening and development of alternative preventative and therapeutic methods.

## Introduction

Protozoal parasites of the genus *Eimeria* are responsible for coccidiosis, a host- and infection site-specific intestinal disease characterized by destruction of the mucosa [1]. Broiler chickens are most commonly infected by *E. acervulina*, *E. maxima* and *E. tenella* [2]. *Eimeria acervulina* infects the duodenum, *E. maxima* the jejunum, and *E. tenella* the ceca [3]. The life cycle is complex, consisting of both sexual and asexual stages. Infection occurs when sporulated oocysts ingested by a susceptible host release sporozoites that invade the epithelium and eventually cause the enterocytes to rupture [3], [4]. Oocysts are released with the feces and the disease is transmitted among birds through ingestion of infective oocysts during feeding. Infected birds display reduced feed intake, bloody diarrhea and hampered weight gain.

The immune response to the parasite is complex, involving both nonspecific and specific immunity. Nonspecific factors include physical barriers, phagocytes and leukocytes, and complement; specific immunity is mediated by antibodies, lymphocytes, and cytokines [3]. Both antibody- and cell-mediated immune responses are activated following infection. Chickens infected with *Eimeria* spp. produce three classes of antibodies, IgY (orthologue to mammalian IgG), IgA and IgM, in response to *Eimeria* [4], which are detected in the blood of infected animals [3]. Although antibodies may reduce invasion at the luminal-mucosal interface, the parasite is effective at entering host cells where contact with antibodies is avoided [3]. Furthermore, studies with bursectomized birds revealed that antibodies play a minute role in the host immune response [5], [6]. Cell-mediated immunity, on the other hand, involving antigen-specific and nonspecific activation of T lymphocytes and macrophages, is key to disease resistance [3], [4], [7]. The lymphocytes, macrophages and other effector cells secrete cytokines and proinflammatory molecules, targeting the appropriate immune responses to the invading parasite.

Coccidiosis has a devastating effect on the poultry industry, costing billions of dollars annually to producers worldwide [2]. Vaccines are costly and time consuming to produce and have shown limited effectiveness on a commercial scale [2]. Drug design is challenging due to lack of known antigens or molecular targets and increasing prevalence of drug-resistant parasites. Public concern over chemical residues in agricultural products further compounds the issue. This dilemma underscores the importance of developing effective preventative and alternative therapeutic approaches to improve bird health and reduce economic losses. Identifying genetic markers of resistance could help in artificial selection of resistant lines through breeding strategies. Genetic markers have been successfully utilized for diagnostic and preventative purposes [8], [9]. Such markers may also lead to elucidation of the biochemical mechanisms responsible for host response and resistance to infection. Identification of early-stage markers could lead to a cost-effective scheme for screening young flocks as well as design of novel therapeutic strategies. Currently, there is no effective diagnostic tool or method for prevention.

Changes in concentrations of plasma proteins can provide insight into the molecular origin of a disease [10]. We hypothesize that the presence of serum proteins in response to *Eimeria* infection is influenced both by broiler genetic background and by the species of *Eimeria*. Lines A and B are two commercial broiler lines that originated from the same genetic stock but were selected for at least 10 generations under different nutritional environments [11] of a corn-based diet for Line A and a wheat-based diet with a greater amino acid density for Line B. This selection led to differences in growth performance.

We used 2-dimensional gel electrophoresis (2DE) followed by matrix-assisted laser desorption/ionization time-of-flight/time-of-flight mass spectrometry (MALDI-TOF/TOF) to evaluate the serum proteome from broilers inoculated with one of three common *Eimeria* species. Protein spot density was quantitatively assessed to identify proteins that displayed significant changes in response to infection. To our knowledge this is the first undertaking of a study to evaluate global changes in serum protein levels in response to *Eimeria* infection in chickens. Changes in the expression profiles of identified proteins provide a valuable resource for future studies aimed at understanding the host response to coccidiosis and identifying diagnostic and pharmacological targets.

## Results

### Bird performance and lesion scores

**Figure 1:**
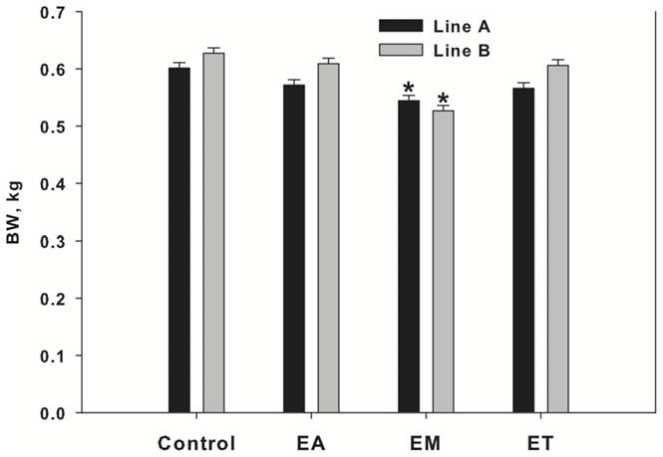
Body weights at Day 21 for Line A and B birds infected with 1 of 3 *Eimeria* strains. Birds were inoculated at day 15 with vehicle (control.), *E. acervulina* (EA), *E. maxima* (EM) or *E. tenella* (ET). A. = Line A, B. = Line B. Bars represent means ± pooled SEM. Asterisks represent difference from respective control group within genetic line (*P*<0.01).

**Figure 2:**
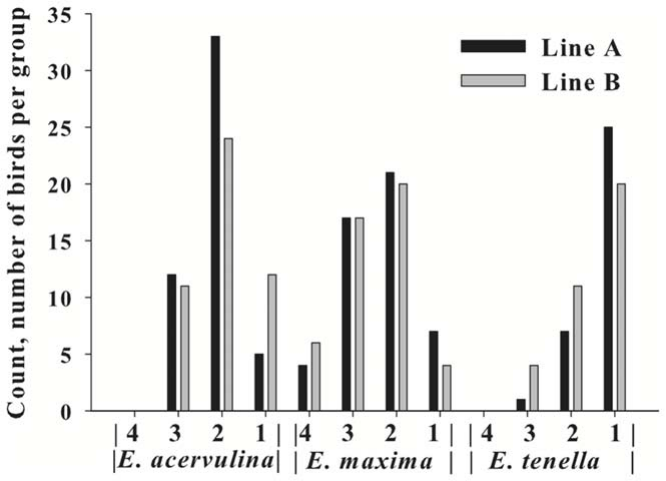
Lesion scores at day 21 (6 d post-infection) for Line A and Line B broilers infected with *E. acervulina*, *E. maxima* or *E. tenella*. Birds were inoculated at 15 d of age. Intestines were evaluated at day 21 and scored for lesions by a single expert [46]. Scores ranged from 0 (no lesions) to 4 (numerous severe lesions).

At day 15 post-hatch (pre-infection), there was no difference in BW among infection groups. At day 21 (6 d post-infection), Line A and B birds that received inoculation with *E. maxima* showed a decreased (*P*<0.01) body weight as compared with A and B control birds, respectively (Figure 1). Body weights at day 21 of birds inoculated with the other two *Eimeria* species were not significantly different from the control birds. The three species of *Eimeria* caused a difference in lesion score distribution (*P* = 0.0001). There were no birds in the *Eimeria*-treated groups with a lesion score of 0. The *E. acervulina*-infected group showed the greatest frequency of birds in the lesion score category 2, the *E. maxima*-infected group in categories of 2 and 3, and the *E. tenella*-infected group in category 1 (Figure 2). Birds with lesion scores of 4 were absent in *E. acervulina*- and *E. tenella*-infected birds. There was no significant difference among Line A and B birds in incidence of lesion scores in *E. acervulina*- (*P* = 0.11), *E. maxima*- (*P* = 0.79) or *E. tenella*-infected (*P* = 0.15) birds. Birds chosen for further analysis were selected based on severity of infection and sample size within the infection group (Table 1). The objective was to select birds with the highest lesion score but with ample sample size for conducting the proteomic analysis (12 birds/infection group).

**Table 1:** Experimental design for proteomics.^a^

Genetic Line	*Eimeria* species	Lesion Score
A	None	0
B	None	0
A	*E. acervulina*	3
A	*E. maxima*	3
A	*E. tenella*	1
B	*E. acervulina*	3^b^
B	*E. maxima*	3
B	*E. tenella*	2^c^

a Total 12 birds within each group. Three birds were composited (N = 4).

b Included 1 bird with a score of “2”.

c Included 1 bird with a score of “3”.

### Protein spots with density that differed among infected and non-infected birds

There were numerous proteins that were absent in the serum of Line A control birds but present in Line A infected birds, including 39, 6 and 11 proteins in birds infected with *E. acervulina*, *E. maxima* and *E. tenella*, respectively. None of these protein spots were common to all infected birds. Conversely, there were numerous spots present in Line A control birds but absent in Line A infected birds including 59, 62 and 31 spots in birds infected with *E. acervulina*, *E. maxima* and *E. tenella*, respectively. Nine of these spots were common to all infected birds. Similarly, there were spots absent in Line B control birds that were present in Line B infected birds, including four spots in the *E. acervulina* group, seven in the *E. maxima* group and one in the *E. tenella* group which was common to all infected birds. There were also spots present in Line B control birds that were absent in Line B infected birds including 40, 6 and 51 spots in the *E. acervulina*, *E. maxima*, and *E. tenella* groups, respectively, and one that was common to all groups. Table S1 contains the summary of density data for all of these spots.

### Summary of spot density and protein identification

**Figure 3:**
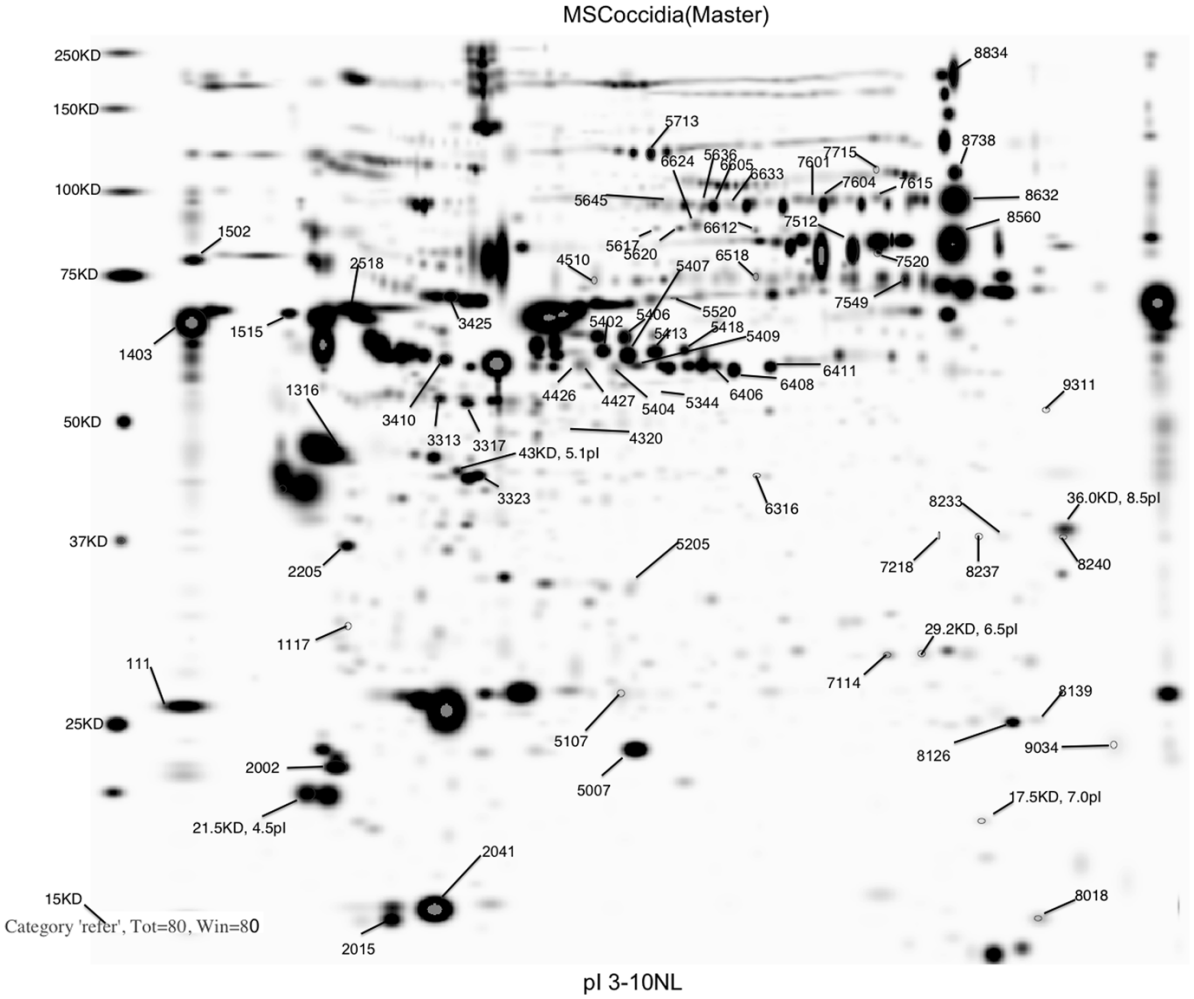
Master image of gel stained with Flamingo™ fluorescent stain following 2-dimensional gel electrophoresis of chicken serum protein. The first dimension was carried out using pH 3–10 immobilized pH gradient strips and the second dimension was performed using 8–16% acrylamide gels. Annotated spots are those showing a significant effect of coccidia infection and/or genetic line×coccidia infection interaction at P<0.001 (Table 2).

A total of 1,266 spots were matched across gels (Figure 3). Protein spots showing a significant effect of coccidia strain and/or broiler genetic line on density at *P*<0.05 were excised (812 spots) and proteins were identified using mass spectrometry analysis following in-gel trypsin digestion for 172 of those spots (Table S2). Forty-six different proteins were identified in these 172 spots (i.e., some of the proteins showed up in multiple spots). To focus on proteins showing the most dramatic changes in response to coccidiosis, only those showing changes at *P*<0.001 are discussed. Of the spots with a corresponding protein identification, 57 showed a main effect of coccidia infection and/or coccidia infection×genetic line interactions (Tables 2 and 3; significant spots annotated in Figure 3). Table S2 shows the complete listing of all identified proteins, expected and observed molecular weights and pIs, as well as Mascot search information. Table S3 shows the least squares means from the statistical analysis of spot density for all spots across treatment groups.

**Table 2:** Summary of proteins displaying an effect of infection and genetic line on protein spot density.

Protein name (SSP#)	Accession ID	Coccidia Strain Main Effect^1^						Coccidia×Genetic Line Interaction
		Control	*Acervulina*	*Maxima*	*Tenella*	SEM	*P*-value	*P*-value
Actin, cytoplasmic (3323)	gi|45382927	9,665,191^a^	4,883,637^c^	5,588,646b^c^	7,519,192^ab^	772,841	*P*<0.001	*P*<0.01
Apolipoprotein A-I preprotein (111)	gi|45382961	28,968,937^a^	7,288,613^b^	12,251,714^b^	24,469,078^a^	1,873,202	*P*<0.001	*P*<0.001
Apolipoprotein A-IV (2205)	gi|45384392	5,964,406^a^	4,010,302^b^	2,862,515^b^	6,931,731^a^	539,937	*P*<0.001	*P*<0.001
Carbonic anhydrase 2 (7114)	gi|46048696	575,841	558,483	789,704	772,827	86,784	*P* = 0.12	*P*<0.001
Collagen alpha-1(II) chain (5205)	gi|45383309	1,301,860^a^	697,723^bc^	557,024^c^	786,662^b^	59,132	*P*<0.001	*P*<0.001
Complement component 3 precursor (5713)	gi|45382303	5,039,469	4,729,898	3,411,610	4,811,415	736,689	*P*>0.05	*P*<0.001
Fetuin B (3313)	gi|50752383	4,695,221^a^	2,540,536^b^	1,237,817^c^	3,094,304^b^	395,239	*P*<0.001	*P*<0.01
Fetuin B (3317)	gi|50752383	7,968,363^a^	4,270,167^b^	1,773,342^c^	4,913,902^b^	856,324	*P*<0.001	*P* = 0.10
Fructose-bisphosphate aldolase C (6316)	gi|226855	396,698^a^	347,692^a^	170,120^b^	390,163^a^	59,186	*P*<0.001	*P*<0.001
Gelsolin precursor (5617)	gi|45384386	463,532^a^	463,584^a^	216,636^c^	341,640^b^	49,410	*P*<0.01	*P*<0.001
Gelsolin precursor (5620)	gi|45384386	2,047,781^a^	891,623^bc^	755,739^c^	1,312,074^b^	193,810	*P*<0.001	*P*<0.001
Gelsolin precursor (6624)	gi|45384386	426,575^b^	1,447,885^a^	422,763^b^	296,345^b^	187,843	*P*<0.001	*P* = 0.2
Glutathione peroxidase 3 (8126)	gi|253735708	5,998,302^a^	4,625,830^b^	2,861,563^c^	5,842,558^ab^	580,341	*P*<0.01	*P*<0.001
Glutathione peroxidase 3 (8139)	gi|253735708	1,861,718^a^	782,050^bc^	488,504^c^	1,055,000^b^	212,366	*P*<0.01	*P* = 0.01
Hemoglobin subunit beta (8018)	gi|49169791	1,978,726	1,782,621	1,577,696	1,611,241	279,892	*P* = 0.73	*P*<0.001
NADH dehydrogenase 1 alpha subcomplex 9 (8240)	gi|57529307	0^b^	1,863,400^a^	0^b^	0^b^	324,960	*P*<0.001	*P*<0.001
Predicted: similar to alpha-1-antitrypsin (4320)	gi|118091960	738,374^a^	321,710^b^	113,369^c^	274,277^bc^	83,160	*P*<0.001	*P*<0.01
Predicted: similar to alpha-2-macroglobulin (8834)	gi|118083276	14,102,798^a^	966,298^b^	10,517,534^a^	14,441,607^a^	2,283,081	*P*<0.001	*P*<0.01
Predicted: similar to alpha-2-macroglobulin (5636)	gi|118083282	1,358,710^a^	996,939^b^	819,770^b^	0^c^	158,444	*P*<0.001	*P*<0.05
Predicted: similar to alpha-2-macroglobulin (5645)	gi|118083282	1,521,002^a^	1,056,752^ab^	762,881^b^	1,414,633^a^	194,277	*P*<0.01	*P*<0.001
Predicted: similar to antithrombin (3410)	gi|118094218	5,753,778	4,171,238	4,412,753	5,826,403	567,184	*P* = 0.09	*P*<0.001
Predicted: similar to fetuin (1316)	gi|50752381	56,000,000^a^	42,000,000^a^	17,000,000^b^	42,000,000^a^	5557492	*P*<0.001	*P*<0.001
Predicted: similar to malate dehydrogenase 2 (8233)	gi|50758110	0^b^	313,303^a^	0^b^	0^b^	50,262	*P*<0.001	*P*<0.001
Predicted: similar to malate dehydrogenase 2 (8237)	gi|50758110	0^b^	261,109^a^	0^b^	0^b^	50,380	*P*<0.001	*P*<0.001

1 Values represent mean spot intensity and standard error of the mean (SEM).

a,b,c Pairwise comparisons, *P*<0.05, Tukey's test.

2 Protein sequence derived from the Gnomon database, no corresponding sequence available in the NCBI non-redundant protein database.

**Table 3:** Summary of proteins displaying an effect of infection and genetic line on protein spot density.

Protein name (SSP#)	Accession ID	Coccidia Strain Main Effect^1^						Coccidia×Genetic Line Interaction
		Control	*Acervulina*	*Maxima*	*Tenella*	SEM	*P*-value	*P*-value
Predicted: similar to plasminogen (7715)	gi|118088308	900,375^b^	1,361,307^a^	452,880^c^	537,023b,c	142,492	*P*<0.001	*P*>0.05
Predicted: similar to plasminogen (8738)	gi|118088308	10,615,797^a^	1,601,869^c^	6,834,921^b^	19,651,558^a^	1,106,292	*P*<0.001	*P*<0.001
Predicted: similar to serpina1d-prov protein (4426)	gi|118091958	3,348,566^a^	2,116,874^b^	842,613^c^	2,512,351^ab^	309,652	*P*<0.001	*P*<0.001
Predicted: similar to serpina1d-prov protein (4427)	gi|118091958	3,570,692^a^	1,747,022^b^	846,797^c^	2,416,778^b^	328,609	*P*<0.001	*P*<0.01
Predicted: similar to serpina1d-prov protein (5344)	gi|118091958	326,643^a^	180,907^b^	49,285^c^	137,577^b^	23,630	*P*<0.001	*P*<0.001
Predicted: similar to serpina1d-prov protein (5404)	gi|118091958	4,194,250^a^	1,703,542^c^	1,040,027^c^	2,134,891^bc^	260,723	*P*<0.001	*P*<0.001
Predicted: similar to thiolase-prov protein (9311)	gi|50745166	136,841^b^	1,270,182^a^	113,660^b^	153,075^b^	220,933	*P*<0.001	*P*<0.01
Predicted: similar to Vanin I (2518)	gi|118088529	20,515,625^a^	12,288,737^b^	10,518,991^b^	6,430,615^c^	1,817,794	*P*<0.001	*P*<0.01
Putative mitochondrial ATP synthase O delta (9034)	gi|118083809	0^b^	729,387^a^	0^b^	0^b^	136,208	*P*<0.001	*P*<0.001
Retinol-binding protein 4 precursor (5007)	gi|45382541	24,000,000^a^	13,000,000^d^	16,000,000^c^	20,000,000^b^	890,467	*P*<0.001	*P*<0.001
Similar to alpha-2-macroglobulin (6605)	hmm36420^2^	5,748,367^a^	3,076,667^b^	3,108,265^b^	6,062,853^a^	546,745	*P*<0.001	*P*<0.05
Similar to alpha-2-macroglobulin (6633)	hmm36420^2^	385,535^c^	424,506^b^	401,584^b^	953,037^a^	85,645	*P*<0.001	*P* = 0.06
Similar to alpha-2-macroglobulin (7601)	hmm36420^2^	2,381,103^ab^	1,846,921^bc^	1,247,989^c^	3,474,678^a^	461,643	*P*<0.05	*P*<0.001
Similar to alpha-2-macroglobulin (7604)	hmm36420^2^	5,943,564^a^	3,111,371^b^	3,260,819^b^	4,823,749^a^	517,461	*P*<0.001	*P* = 0.4
Similar to alpha-2-macroglobulin (7615)	hmm36420^2^	2,887,627^ab^	3,811,717^a^	1,702,215^b^	3,871,481^a^	666,299	*P* = 0.09	*P*<0.001
Similar to alpha-2-macroglobulin (8632)	hmm36420^2^	21,255,505^a^	3,587,960^b^	13,721,198^b^	26,129,323^a^	3,597,494	*P*<0.001	*P*<0.01
Ovoinhibitor precursor (5402)	gi|71895337	18,000,000^a^	11,000,000^c^	9,523,165^c^	15,000,000^b^	941,457	*P*<0.001	*P*<0.05
Ovoinhibitor precursor (5406)	gi|71895337	8,810,696^a^	7,058,396^b^	4,950,679^b^	8,129,741^a^	751,190	*P*<0.01	*P*<0.001
Ovoinhibitor precursor (5407)	gi|71895337	30,000,000^a^	19,000,000^b^	18,000,000^b^	27,000,000^a^	1,526,231	*P*<0.001	*P*<0.05
Ovoinhibitor precursor (5413)	gi|71895337	18,000,000^a^	12,000,000^b^	10,000,000^b^	16,000,000^a^	895,862	*P*<0.001	*P*<0.001
Ovoinhibitor precursor (5418)	gi|71895337	5,348,187^a^	3,890,585^b^	2,660,976^c^	4,641,192^a^	357,541	*P*<0.001	*P*<0.001
Ovoinhibitor precursor (6406)	gi|71895337	6,575,008^a^	4,766,226^b^	3,466,971^c^	6,002,083^ab^	799,061	*P*<0.05	*P*<0.001
Ovotransferrin, type indeterminate (6612)	gi|71274075	1,359,688^a^	771,291^b^	592,526^b^	1,229,787^a^	114,630	*P*<0.001	*P*<0.001
Ovotransferrin, type indeterminate (7520)	gi|71274075	1,514,129^b^	8,266,097^a^	584,638^b^	969,613^b^	1,196,575	*P*<0.001	*P*<0.001
Ovotransferrin, type indeterminate (8560)	gi|71274075	51,404,511^a^	4,926,704^b^	25,721,585^b^	47,693,242^a^	7,430,877	*P*<0.001	*P*>0.05
Ovotransferrin, type indeterminate (7512)	gi|71274075	23,095,199a,b	20,570,819a,b	17,587,155^b^	23,541,588^a^	2,017,930	*P* = 0.15	*P*<0.001
Ovotransferrin, type indeterminate (1502)	gi|71274075	9,924,322a,b	5,778,143^c^	7,662,680^b^	11,046,185^a^	1,081,616	*P*<0.01	*P*<0.001
Transthyretin precursor (2015)	gi|45384444	11,000,000^a^	6,877,510^b^	6,529,719^b^	9,422,214^a^	720,754	*P*<0.001	*P*<0.01
Transthyretin precursor (2041)	gi|45384444	110,000,000^a^	70,000,000^b^	58,000,000^b^	64,000,000^b^	7,075,221	*P*<0.001	*P*<0.0001
Vitamin D-binding protein (6408)	gi|45382425	6,575,008^a^	4,766,226^bc^	3,466,971^c^	6,002,083^b^	799,061	*P*<0.001	*P*<0.0001
Vitamin D-binding protein (5409)	gi|45382425	2,302,999^a^	2,203,807^b^	1,627,338^b^	2,731,565^a^	345,994	*P* = 0.1	*P*<0.001
Vitamin D-binding protein (6411)	gi|45382425	10,000,000^a^	4,405,014^b^	4,537,317^b^	9,442,958^a^	720,232	*P*<0.001	*P*<0.0001
Vitelline membrane outer layer protein 1 (2002)	gi|268370086	32,000,000^a^	23,000,000^b^	15,000,000^c^	30,000,000^a^	1,785,242	*P*<0.0001	*P*<0.0001

1 Values represent mean spot intensity and standard error of the mean (SEM).

a,b,c Pairwise comparisons, *P*<0.05, Tukey's test.

2 Protein sequence derived from the Gnomon database, no corresponding sequence available in the NCBI non-redundant protein database.

### Proteins influenced by broiler genetic line and *Eimeria* strain

**Figure 4:**
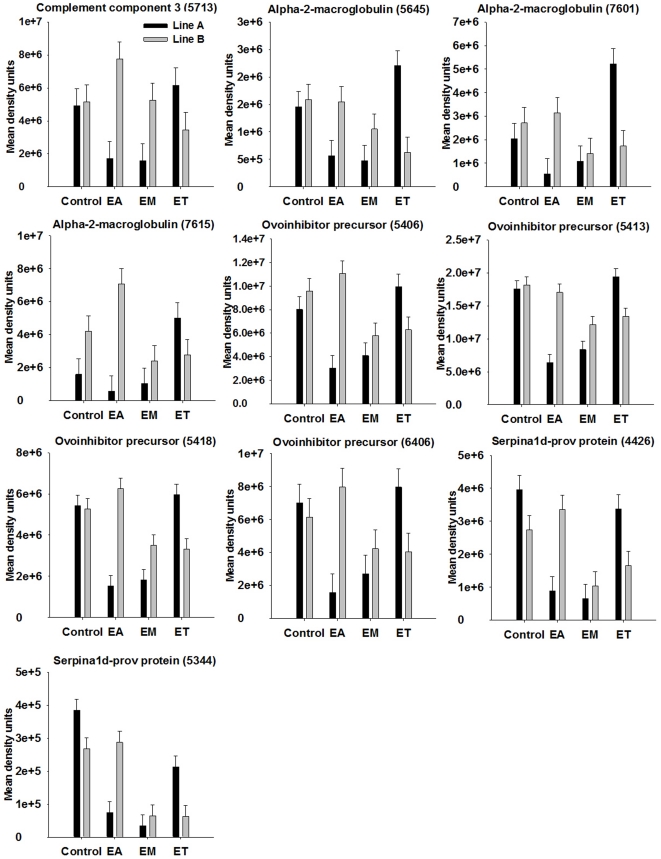
Broiler genetic line×coccidia infection interactions (*P*<0.001) for complement component 3 and protease inhibitors. Line A and B broiler chicks were either not infected (control) or were inoculated with 1 of 3 *Eimeria* species, *E. acervulina* (EA), *E. maxima* (EM) or *E. tenella* (ET) at 15 d post-hatch. At 21 d (6 d post-infection) serum was harvested from whole blood samples and used for 2D gel electrophoresis followed by MALDI TOF/TOF. Protein density was evaluated by densitometric analysis of spot intensity followed by ANOVA. Values represent mean density units ± SEM (N = 6).

The proteins that were influenced by coccidia infection are shown in Tables 2 and 3 with means for the different groups and pooled SEM. For proteins showing significant two-way interaction of genetic line and coccidia infection at *P*<0.001 (*P*-values shown in Tables 2 and 3) on spot density, proteins patterns among genetic lines in response to infection are illustrated in Figures 4–7. Among the significant main effects (Tables 2 and 3), it is interesting to note that increases of a protein in response to infection were detected mainly in *E. acervulina* and *E. tenella*-infected birds. For most of the proteins, spot density was greatest in the control group. Several proteins were detected only in *E. acervulina*-infected birds. Further inspection of the 2-way interactions reveals even more interesting differences (Figures 4–7). The concentration of most of these proteins was similar among Line A and B control birds. The genetic line differences were manifested in response to coccidia infection, with distinct *Eimeria* species-specific responses. The proteins that were only detected in infected birds, including malate dehydrogenase 2, NADH dehydrogenase alpha subunit complex 9 and an ATP synthase, only appear in Line A birds infected with *E. acervulina*. In general, the most dramatic changes in response to infection (>2-fold) occurred in Line A birds infected with *E. acervulina* or *E. maxima* (Figures 4–7).

**Figure 5 pone-0014636-g005:**
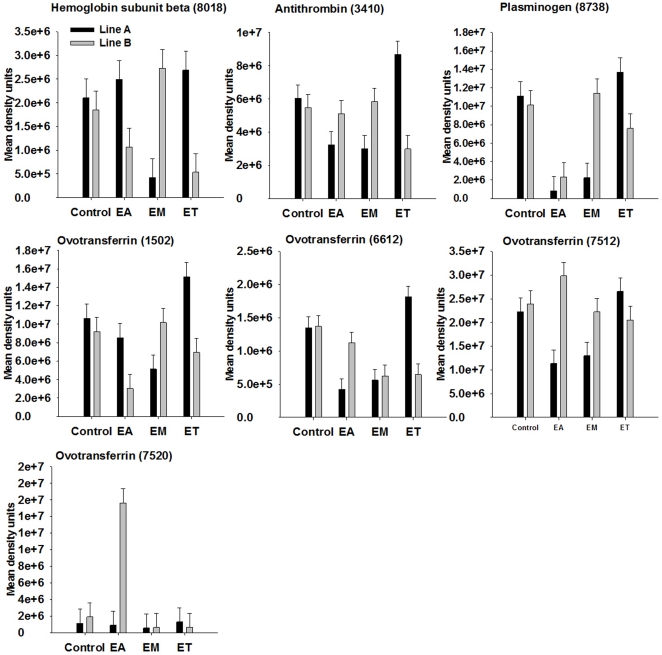
Broiler genetic line×coccidia infection interactions (*P*<0.001) for iron-binding and blood-clotting proteins. Line A and B broiler chicks were either not infected (control) or were inoculated with 1 of 3 *Eimeria* species, *E. acervulina* (EA), *E. maxima* (EM) or *E. tenella* (ET) at 15 d post-hatch. At 21 d (6 d post-infection) serum was harvested from whole blood samples and used for 2D gel electrophoresis followed by MALDI TOF/TOF. Protein density was evaluated by densitometric analysis of spot intensity followed by ANOVA. Values represent mean density units ± SEM (N = 6).

**Figure 6 pone-0014636-g006:**
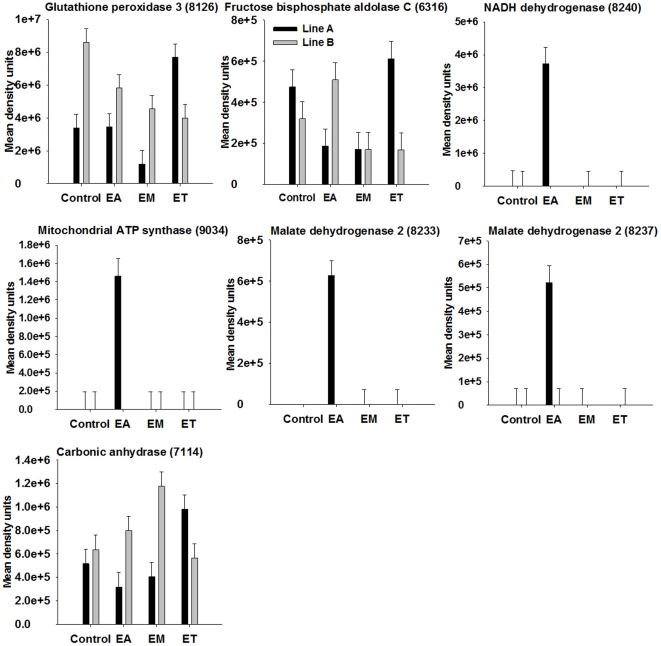
Broiler genetic line×coccidia infection interactions (*P*<0.001) for metabolic and oxidative-stress related proteins. Line A and B broiler chicks were either not infected (control) or were inoculated with 1 of 3 *Eimeria* species, *E. acervulina* (EA), *E. maxima* (EM) or *E. tenella* (ET) at 15 d post-hatch. At 21 d (6 d post-infection) serum was harvested from whole blood samples and used for 2D gel electrophoresis followed by MALDI TOF/TOF. Protein density was evaluated by densitometric analysis of spot intensity followed by ANOVA. Values represent mean density units ± SEM (N = 6).

**Figure 7:**
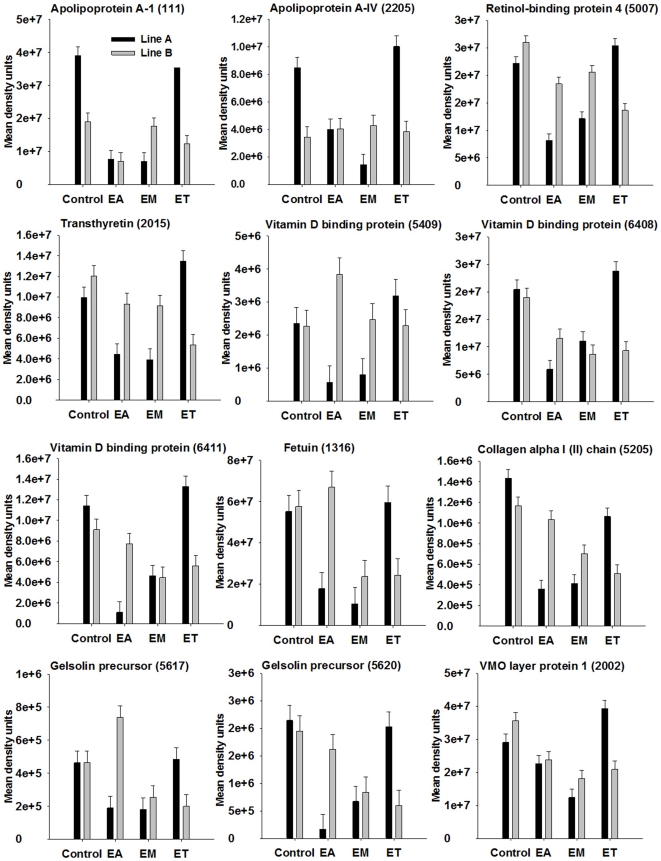
Broiler genetic line×coccidia infection interactions (*P*<0.001) for cargo and structural proteins. Line A and B broiler chicks were either not infected (control) or were inoculated with 1 of 3 *Eimeria* species, *E. acervulina* (EA), *E. maxima* (EM) or *E. tenella* (ET) at 15 d post-hatch. At 21 d (6 d post-infection) serum was harvested from whole blood samples and used for 2D gel electrophoresis followed by MALDI TOF/TOF. Protein density was evaluated by densitometric analysis of spot intensity followed by ANOVA. Values represent mean density units ± SEM (N = 6).

### Changes in complement and protease inhibitors

Complement component 3 (C3) was identified in six spots (Table S2). Of these six spots, one displayed a significant genetic line×coccidia infection interaction (Figure 4). We identified alpha-2-macroglobulin in 36 spots, nine of which showed an effect of coccidia infection on density (Table S2, Tables 2 and 3). We identified ovoinhibitor precursor in 13 spots (Table S2). Six of these spots displayed a significant change in density in response to coccidia infection (Table 3; Figure 4). Serpina1d-prov protein was identified in 8 spots of which 4 were influenced by coccidia infection (Table 3; Figure 4). Antithrombin was identified in 3 spots, 1 of which changed in response to *Eimeria* infection (Figure 4).

### Changes in iron-binding and blood-clotting related proteins

Hemoglobin subunit beta showed an interaction of broiler genetic line×coccidia infection (Figure 5). We identified plasminogen in two spots (Table S2), both of which changed in response to *Eimeria* infection (Table 3; Figure 5). Ovotransferrin was identified in 9 spots (Table S2). Five of these spots showed a coccidia infection×genetic line interaction (Figure 5).

### Changes in metabolic and oxidative-stress related enzymes

Vanin-1 was identified in one spot and was influenced by coccidia infection, with at least a 2-fold reduction in concentration in response to infection with any of the three species of *Eimeria* (Table 3). Glutathione peroxidase 3 was identified in three spots, two of which changed in response to infection (Table 2; Figure 6). In general, there was a decrease in spot concentration in response to infection with either *E. acervulina* or *E. maxima*.

Fructose bisphosphate aldolase C was identified in one spot and density was down-regulated by more than 2-fold in Line A birds infected with *E. acervulina* or *E. maxima*, but was less affected in other infection groups (Figure 6). The NADH dehydrogenase (ubiquinone) 1 alpha subcomplex 9 was identified in one spot which displayed a significant coccidia infection×genetic line interaction where the protein was detected only in Line A birds infected with *E. acervulina* (Figure 6).

A subunit of ATP synthase, an H^+^ transporting, mitochondrial complex, was identified in one spot which was influenced by coccidia infection and showed a coccidia infection×genetic line interaction (Table 3; Figure 6). This protein was detected only in Line A birds inoculated with *E. acervulina*, identical to the pattern observed for NADH dehydrogenase 1 alpha subcomplex 9, a functionally related protein.

We identified malate dehydrogenase 2 in two spots. Density of both spots showed an effect of coccidia infection×genetic line (Table 2). For both spots, only Line A birds infected with *E. acervulina* contained this protein in their sera (Figure 6).

Carbonic anhydrase 2 was identified in a single spot. It displayed a significant 2-way interaction (Figure 6). Density was similar among all control birds, but differed among genetic lines in response to infection. In Line A birds, there was an almost 2-fold increase in response to *E. tenella*. In Line B birds, density increased approximately 2-fold in response to *E. maxima* infection. Thiolase-prov protein (an acetyl-CoA acetyltransferase) spots showed main effects of coccidia infection where density was reduced at least 9-fold in birds infected with *E. acervulina* (Table 3).

### Changes in carrier and structural proteins

Apolipoprotein A-1 (identified in 11 spots) and A-IV (identified in one spot), retinol-binding protein 4 (identified in one spot), transthyretin (identified in three spots), vitamin D binding protein (identified in 5 spots) and fetuin protein spot densities were identified in 11, 1, 1, 3, 5 and 4 spots, respectively, and all decreased by at least 2-fold in Line A birds infected with *E. acervulina* or *E. maxima* (Figure 7). Density of apolipoprotein A-I and A-IV was almost 2.5-fold greater in Line A control birds as compared with Line B control birds.

Collagen alpha 1 (II) chain and gelsolin were identified in 1 and 3 spots, respectively, and also decreased more than 2-fold in Line A birds infected with *E. acervulina* or *E. maxima* (Figure 7). The vitelline membrane outer layer protein 1 was identified in a single spot which decreased in density in both Line A and B birds in response to *E. maxima* inoculation, and was almost 2-fold greater in abundance in Line A birds infected with *E. tenella* in comparison to Line B birds (Figure 7).

## Discussion

The objective of this study was to identify proteins in the serum of broilers that are differentially detected in response to coccidia infection and broiler genetic line. Birds were orally inoculated with *E. acervulina*, *E. maxima*, or *E. tenella* at day 15 post-hatch and euthanized at day 21 (6 d post-infection). In both Line A and B birds, BW gain was decreased in response to *E. maxima*-infection. Although not statistically significant, there was obviously a trend for differences in distribution of lesion scores within *Eimeria* species infections between the two genetic lines of broilers. In *E. acervulina*-infected birds, there were more Line B birds with a lesion score of 1 and more Line A birds with a lesion score of 2. In *E. tenella*-infected birds, there were more Line A birds with a lesion score of 1 and more Line B birds with a lesion score of 2. A number of factors determine an animal's resistance to a disease. In a commercial setting, broilers can be infected with more than one *Eimeria* species at a time, further complicating the etiopathogenesis of the disease. In the present study, the lesion score and body weight data alone suggest that Line A and B responses were similar in response to *E. maxima*-infection, whereas there was a greater severity of infection in Line A birds in response to *E. acervulina* and in Line B birds in response to *E. tenella*. In order to evaluate the serum proteome in response to infection, we chose to use groups of birds for the proteomics that exhibited the same lesion score but also provided enough numbers to allow for adequate statistical analyses. The goal was to have birds with the greatest severity of infection in order to provide a suitable model of coccidiosis.

As described in the methods, we used a commercial kit to remove albumin and immunoglobulins from the serum in order to enhance the density of rare proteins that could be masked by the overwhelming density of these proteins. However, the use of the kit did not prove to be 100% efficient since we did identify albumin and immunoglobulins in several spots. We identified 2 spots as “hypothetical protein” (Table S2). In the only other documented report of the chicken serum proteome, Huang et al. [12] identified novel serum proteins that we observed in our protein list, including PIT54. A number of proteins were identified in multiple spots with differing masses. Due to possible post-translation modifications (e.g. glycosylation, phosphorylation, etc.) a single protein may be detected at multiple molecular weights and/or pI's.

The density of a number of proteins detected in serum was influenced by *Eimeria* species. The differential responses elicited in response to the three *Eimeria* species may relate to the region-specific infection of these strains. For example, the response to *E. tenella* which colonizes the ceca would be expected to differ from the response to *E. acervulina* which colonizes the proximal small intestine. At 6 d post-infection in broilers, an *E. acervulina* or *E. maxima* infection induced a CD8+ T-cell and macrophage response in their respective intestinal sites, whereas *E. tenella* induced a cecal CD4+ T-cell and macrophage response [13]. A differing profile of cytokine gene expression was also observed among the infected groups. In general, we observed that proteins involved in the innate immune response, mitochondrial metabolism, blood clotting and iron metabolism showed changes in density in response to infection with coccidiosis. These groups of proteins all fit the model of systemic change activated by cytokines in response to tissue invasion by a microorganism [14]. This includes a decrease in levels of circulating lipoproteins, activation of complement and blood clotting, a decrease in serum levels of vitamins and minerals, an increase in concentration of acute phase proteins in the blood and decrease in normal blood proteins such as transthyretin, retinol binding protein, transferrin and albumin. Many of these changes are triggered by alterations in hepatic metabolism due to activation of the immune system. In our study, these very proteins were the ones that changed dramatically in response to coccidiosis.

The C3 protein is a member of the thioester bond-containing alpha-2 macroglobulin family of proteins [15]. It consists of two chains including a ∼118 kDa alpha-chain and ∼68 kDa beta-chain that are released upon cleavage of the thioester linkage [16]. Hence, the protein that we detected is most likely the complete alpha chain. The complement system participates in the inflammatory response through formation of anaphylatoxins that stimulate chemotaxis, cell activation and the triggering of phagocytosis, degranulation and cell lysis [17]. The complement system plays an important role in host defense against microorganisms and promoting the adaptive immune response [17]. The C3 protein plays a pivotal role as the convergence point for the classical, alternative and lectin pathways of complement activation [15], [17], [18].

The alpha-2 macroglobulin is an acute phase protein that functions as a protease inhibitor comprised of four identical 180 kDa subunits [15]. Ovoinhibitor and antithrombin also function as protease inhibitors [19]. Ovoinhibitor precursor was previously identified in several spots from 2D gels containing chicken serum protein [12]. Antithrombin, as the name implies, is involved in inhibition of the blood clotting cascade [20]. Serpina 1d-prov, a serine protease inhibitor, was previously identified as a protein that changed in response to infection of cells with an intracellular protozoal parasite [21]. The serpin proteins are of particular relevance to the study of host-*Eimeria* interactions, due to the known secretion of serine proteases by *Eimeria* spp. [22], [23]. Protease inhibitors were protective in virus-infected chickens where viruses use plasmin to become infectious [24]. Protease inhibitors were also effective at reducing cell invasion by *Eimeria* sporozoites, suggesting that proteases are critical in facilitating *Eimeria* infection [25], [26].

The changes in density of serum iron-binding proteins in response to coccidia infection could be related to both the anti-microbial properties of the proteins as well as their responsiveness to blood iron levels. Reduced plasma iron levels were observed in chicks infected with various *Eimeria* spp. [27], [28]. This reduction in iron status could be attributable to reduced intestinal absorption of iron, blood loss from intestinal hemorrhaging, and secondary bacterial infection [28]. The acute phase response is known to cause hypoferremia [27]. Serum transferrin, as the name implies, is an iron-binding glycoprotein produced in the liver and subsequently released into the bloodstream [29], [30]. Ovotransferrin was reduced in birds infected with *E. acervulina* or *E. maxima*. In chickens, serum transferrin functions as both an iron-carrier as well as an anti-bacterial and anti-viral protein, in contrast to mammals, where serum transferrin is an iron carrier and defensive properties are attributable to a closely related protein, lactoferrin, which is absent in chickens [30], [31], [32].

Plasminogen is a zymogen that circulates until activated to plasmin by urokinase type Pg activator or tissue-type plasminogen activator [33]. It functions in fibrinolysis and tissue remodeling, and was shown to play a role in pathogen-induced inflammation by stimulating release of cytokines by macrophages [33]. Similar to the effect of *Eimeria* infection on iron-binding proteins, the effect on blood-clotting factors and protease inhibitors may relate to the clinical manifestations in the gut including destruction of the mucosa and severe hemorrhaging, readily visible upon inspection of the gut.

Proteins involved in antioxidant mechanisms are likely to be involved in the response to coccidiosis. Impaired oxidative balance was demonstrated in a model of *Eimeria* infection in chickens through evaluation of blood plasma oxidative stress markers [34]. Glutathione peroxidase 3, an extracellular enzyme that is abundant in the bloodstream, is a member of a family of enzymes that play an important role in reducing peroxides into less toxic products [35]. Glutathione peroxidase 3 was reduced in the serum of birds infected with either *E. acervulina* or *maxima* but not *tenella*. Vanin 1 is a pantotheinase that cleaves pantotheinine into vitamin B_5_ (pantothenic acid) and cysteamine, a sulfhydral compound that inhibits glutathione synthesis [36]. Vanin 1was described as a cellular sensor for oxidative stress that orchestrates the innate immune response [37]. Vanin 1 is of interest for further studies aimed at characterizing the host response to coccidiosis since we observed at least a 2-fold reduction in serum concentrations of this protein in response to infection with all 3 species of *Eimeria*, with the biggest drop in response to *E. tenella* infection.

We identified other proteins that may also provide clues about the response to *Eimeria* infection, including a number of proteins that exhibit reduced circulating levels in the bloodstream during the response to tissue invasion by microorganisms [14]. Apolipoprotein A-IV is a lipid-binding plasma protein made by enterocytes and enters the bloodstream attached to chylomicrons [38]. Interestingly, in a recent study of two chicken lines that differ in coccidiosis susceptibility, apolipoprotein A-IV was identified as an intestinal gene that showed more than a 2-fold reduction in gene expression several days after infection with *E. maxima* in 4-wk old coccidia-resistant chickens [39]. Expression levels were not influenced by coccidia infection in the disease-susceptible chicken strain. In our study, this protein was not influenced by infection in Line B birds, but showed a decrease in response to *E. acervulina* and *maxima* infection in Line A birds. Apolipoprotein A-1, a component of high density lipoproteins in plasma, also decreased in Line A birds infected with *E. acervulina* and *maxima*. Other proteins that function as cargo carriers in the blood and several structural proteins showed similar changes. Fetuin spot density was reduced the most in birds that were infected with *E. maxima*. Fetuin, which transports various cargo in the blood, inhibited cell invasion by *E. tenella* due to inhibition of surface lectins [40].

Gelsolin is an actin-binding protein that circulates in the blood and plays a role in tissue damage [41]. Depletion of this protein is associated with poor prognosis in animal models of sepsis, although the mechanism is unclear. Line A birds infected with *E. acervulina* showed a decrease in this protein at levels that were lower than in Line B birds infected with *E. acervulina*.

Interestingly, many of the proteins that were differentially detected among Line A and B birds (Figures 4–7) showed a similar pattern whereby concentration was reduced in Line A birds infected with either *E. acervulina* or *maxima*. Furthermore, while there was no difference in these proteins between A and B birds infected with *E. maxima*, there was clearly a difference for most of the proteins in *E. acervulina*-infected birds, with a greater concentration in Line B birds. In general, for *E. acervulina* or *E. maxima*-infected birds, proteins were more abundant in Line B, whereas for *E. tenella*-infected birds, proteins were more abundant in Line A. Some of the proteins that were influenced by infection were identified in multiple spots. Finding these proteins in multiple spots but with only a subset exhibiting density differences based upon genetic line and/or infecting species suggests that changes in post-translational modifications to these proteins are critical to understanding the infection response. The lesion score data suggest a greater severity of infection in Line A birds infected with *E. acervulina* as compared to Line B, and the patterns in protein spot density may provide clues about the differences in severity of infection.

Blood plasma can contain proteins that are secreted by cells or that are released during tissue leakage, necrosis, apoptosis and hemolysis [42]. This may explain our detection of proteins such as mitochondrial metabolic proteins and carbonic anhydrase 2. Additionally, there can be premature release of plasma proteins from the liver, representing incompletely modified proteins, single polypeptides of multi-chain complexes and others that “escape” [43]. Changes in these forms of proteins can provide clues about disease states and serve as important clinical markers.

Several mitochondrial proteins were only detected in Line A birds infected with *E. acervulina* and at relatively high density (>100,000 density units). The NADH dehydrogenase is a multi-subunit complex that functions during the electron transport chain to transfer electrons from NADH to coenzyme Q [44]. This complex is also known as NADH:quinine oxidoreductase. The ATP synthase is an H^+^ transporting, mitochondrial complex. Malate dehydrogenase catalyzes the NAD-dependent reversible conversion of malate into oxaloacetate [45]. Most animals express two isoforms, one being cytosolic and the other localizing to the mitochondria.

In summary, coccidiosis is an intestinal disease that causes substantial economic losses for the poultry industry. There is a dire need for more research aimed at fully unraveling the complex biology of host-parasite interactions and the host response, as well as research to elucidate potential diagnostic markers for early detection of the disease and targets for preventative and alternative therapeutic strategies. Using the powerful method of 2DE followed by MALDI-TOF-TOF mass spectrometry, we identified numerous proteins that are detected at different levels in the blood after 6 d in response to inoculation with one of three common species of *Eimeria*. We detected changes in proteins associated with the acute phase response, antioxidant status, nutrient transport, blood clotting, and iron binding. This is unsurprising, given the effect of *Eimeria* infection on gut morphology and mucosal integrity and the known systemic response elicited by animals infected by a microorganism. We identified several proteins that were detected in only a subset of infected birds, and a number of additional spots without identified proteins that showed such a response. All of these identified proteins were associated with mitochondrial metabolism and therefore unusual to detect in the blood. These proteins are ideal candidates for future time-course studies to determine the timing of induction coinciding with stage of infection. These proteins could serve as early diagnostic markers for screening flocks or pharmacological targets once the physiological basis for their presence in the blood is understood. All of the proteins with ambiguous identities are appealing targets for future studies and may represent novel proteins that play an important role in host defense. Further research will be aimed at better characterizing the biochemical pathways associated with these proteins and developing them as targets for disease prevention tools.

## Materials and Methods

### Birds and *Eimeria* infection

**Figure 8:**
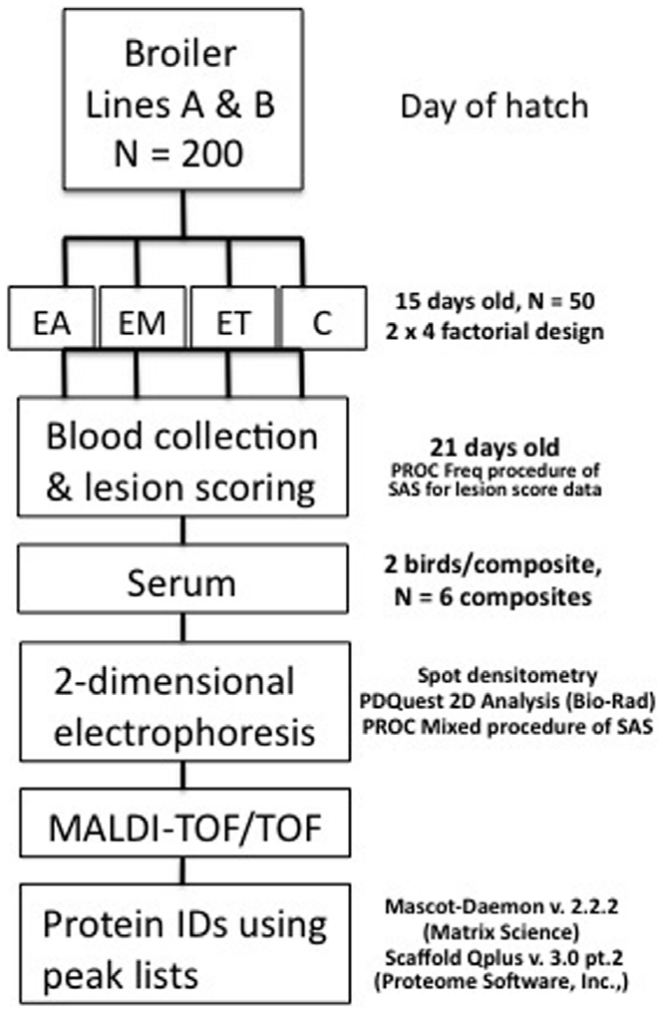
Experimental design and workflow. Line A and B chicks were inoculated at day 15 with *E. acervulina* (EA), *E. maxima* (EM) or *E. tenella* (ET), or the vehicle (control; C). Blood was collected and lesion scoring was performed on the small intestine. Serum samples were depleted of IgG and albumin and composited. Two-dimensional electrophoresis was perfomed and gels were stained with Flamingo stain. Protein spot density was analyzed and spots showing a significant effect of coccidia strain and/or broiler genetic line at *P*<0.05−0.01 (250 spots), *P*<0.01−0.001 (248 spots), and *P*<0.001 (314 spots) were excised and analyzed by matrix-assisted laser desorption/ionization tandem time-of-flight mass spectrometry.

The current study was approved by the Institutional Animal Care and Use Committee at Virginia Tech. Line A and B eggs were obtained from Aviagen (Huntsville, AL). Birds were hatched and reared in floor pens at the Virginia Tech chicken farm with ad-libitum access to a standard corn-soy commercial diet [11] that was non-medicated and free access to drinking water. Individual birds were inoculated at 15 d of age with sporulated oocysts from *E. acervulina* (30,000), *E. maxima* (10,000) or *E. tenella* (2,000). The design and outline of procedures is shown in Figure 8. Each infection group contained 50 birds. The oocyst dose and decision to inoculate with single *Eimeria* species was based on a preliminary study using birds at similar ages from the same genetic backgrounds. We found that mixed inoculations resulted in a high incidence of mortality (data not shown).

### Serum collection

Whole blood and intestinal samples were collected at 21 d of age (6 d post-inoculation). Birds were euthanized by CO_2_ asphyxiation followed by cervical dislocation. Intestines were evaluated and scored for lesions by a single expert with scores ranging from 0 (no lesions) to 4 (numerous severe lesions) [46]. Blood was collected via the jugular vein and tubes were incubated at a slant overnight at 4°C, centrifuged for 20 min, 4°C, at 2,500×g (IEC Centra GP8R), and sera were removed and added to cryovials in 1-mL aliquots, and stored at −80°C. Blood samples from birds with the highest lesion score from each group were composited as shown in Table 1. For example, in *E. acervulina*-infected birds, the highest lesion score associated with enough birds to conduct further analyses was “3”.

### Serum protein sample preparation

Serum samples were thawed in a slurry bath, vortexed and composited with equal amounts as shown in Table 1 with six composites for each infection group and each composite comprised of two birds. Albumin and IgG proteins were removed from serum samples with a commercial kit following the manufacturer's protocol and stored at −80°C (ProteoExtract® Albumin/IgG Removal Kit, Maxi, Calbiochem, Darmstadt, Germany). Proteins from albumin/IgG-depleted samples were then concentrated using the ProteoExtract® kit, following the manufacturer's instructions (Calbiochem). Protein concentrations were determined using the 2-D Quant Kit (GE Healthcare; Piscataway, NJ) and a spectrophotometer (Spectronic Genesys).

### Two-dimensional electrophoresis

For each composite, 300 µg of protein were added to running buffer (8.1 *M* urea, 2 *M* thiourea, 4% CHAPS, 0.2% CA (Biolyte) (3–10), 2 m*M* TBP and 0.05 *M* DTT) and added to wells of isoelectric focusing (IEF) trays in PROTEAN IEF cell units. Isoelectric focusing was performed as previously described [47]. Acrylamide gradient gels (8 and 16%) were prepared and electrophoresis performed as before [47]. Two-dimensional SDS-PAGE standards (Bio-Rad) were run using the same gel preparation and running conditions as the samples. Molecular weight ladders (Precision Plus Protein Standard Plugs, Bio-Rad) were run on every gel to assist in both molecular weight assignments and gel orientation. Gels were stained with Flamingo (Bio-Rad) overnight. Gels were washed (10% methanol, 7% glacial acetic acid) for 1 h and scanned using PD Quest software (Molecular Imager Fx).

### Protein spot analysis

A total of 1,266 protein spots were identified and a matched set was generated using PDQuest 2-D Analysis Software. The gel images from the standards and samples were combined in the master image. The molecular weight and pI were assigned to the unknowns based on the known molecular weights and pIs of the standards. A total of 96 gels were included in the analysis (2 genetic lines×4 infections×6 composite samples×duplicate gels) and spots were evaluated by densitometry. To encompass the largest possible number of infection-influenced proteins, spots were excised that showed main effects of coccidia infection and/or a 2-way interaction of genetic line×infection at *P*<0.05−0.01 (250 spots), *P*<0.01−0.001 (248 spots) and *P*<0.001 (314 spots). For each spot, gels for excision were chosen based on maximum spot density in order to maximize likelihood of mass spectrometric detection. Spots were excised using the Proteome Works Spot Cutter and transferred to sterile microtiter plates containing 0.1% acetic acid. Gel cores were de-stained for 12 h in 25 m*M* ammonium bicarbonate/50% acetonitrile and further dehydrated in 100% acetonitrile. Cores were rehydrated in 10 µg/mL of trypsin (Sigma Aldrich) with ProteaseMAX Surfactant, Trypsin Enhancer (Promega; Madison, WI) in 25 m*M* ammonium bicarbonate and incubated for 3 h at 37°C.

### Mass spectrometry and data acquisition

Digested samples were spotted onto a polished steel MALDI plate followed by 4 mg/mL α-cyano-4-hydroxycinnamic acid solution prepared in 50% acetonitrile enhanced with ammonium citrate, formic acid, and trifluoroacetic acid. Data were acquired utilizing an Applied Biosystems 4800 MALDI TOF/TOF as previously reported [47].

### Protein identification

Mascot Daemon v. 2.2.2 (Matrix Science Inc., Boston, MA) was used to automatically submit peak lists to a local Mascot Server v. 2.2 (Matrix Science Inc., Boston, MA) search engine. Two separate searches for each peak list were performed using two reverse concatenated databases derived from *Gallus gallus* specific protein databases available from the NCBI website ftp://ftp.ncbi.nih.gov/genomes/Gallus_gallus/protein/ containing only annotated proteins (protein.fsa.gz, 37058 sequences) or containing ab initio protein predictions (Gnomon_prot.fsa.gz, 83750 sequences) generated utilizing the NCBI eukaryotic gene prediction tool Gnomon as previously described [47]. Results for both sets of searches were imported into Scaffold Qplus v. 3.00.02 (Proteome Software, Inc., Portland, OR) utilizing Java 1.6.0_18, amd64, 64 bit, and simultaneously searched using the same parameters described above utilizing a second search engine, X! Tandem. The tandem MS spectra were inspected manually to confirm that the spectra contained at least four consecutive -y or -b ions matching the predicted amino acid sequence.

### Statistical analyses

Lesion score data were analyzed using the PROC Freq procedure of SAS (Cary, NC) and Fisher's Exact test to compare differences in incidence of lesion scores among the two genetic lines within coccidia infection. A Chi-square test was used to evaluate differences in distribution of lesion scores among infections within genetic lines. Results were considered significant at *P*<0.05. Body weight data were analyzed by the PROC Mixed procedure of SAS for day 21 (BW were not different at day 15). Tukey's test was used to further evaluate significant effects.

The spot density data were analyzed as a 2×4 factorial arrangement with two genetic lines (A and B)×4 infections (control, *E. acervulina*, *E. maxima* and *E. tenella*). The PROC Mixed procedure of SAS was used to evaluate the main effects of genetic line and infection and the 2-way interaction. Tukey's test was used to further evaluate significant effects. Results were considered significant at *P*<0.05.

## Supporting Information

Table S1 Protein spots present in only a subset of groups.
(0.15 MB PDF)

Table S2 Information for all proteins that were identified.
(0.07 MB PDF)

Table S3 Least squares means for effects of broiler line, coccidia infection and the interaction of broiler line×coccidia infection on spot density.
(1.69 MB PDF)
