# Correction: *Eimeria* Species and Genetic Background Influence the Serum Protein Profile of Broilers with Coccidiosis

**DOI:** 10.1371/annotation/e9373e8a-b316-49c6-b33f-f49557453b48

**Published:** 2011-02-11

**Authors:** Elizabeth R. Gilbert, Chasity M. Cox, Patricia M. Williams, Audrey P. McElroy, Rami A. Dalloul, W. Keith Ray, Adriana Barri, Derek A. Emmerson, Eric A. Wong, Kenneth E. Webb

Several authors were omitted from the contributions to the "Wrote the paper" section. The correct Author Contributions are: Conceived and designed the experiments: CMC APM DAE EAW KEWJ. Performed the experiments: CMC PMW APM WKR AB. Analyzed the data: ERG CMC PMW RD WKR KEWJ. Contributed reagents/materials/analysis tools: APM WKR DAE KEWJ. Wrote the paper: ERG RAD EAW KEW.

