# Supplementary material for: Eimeria Species and Genetic Background Influence the Serum Protein Profile of Broilers with Coccidiosis
Source: PLoS One. 2011 Jan 31;6(1):e14636. doi: 10.1371/journal.pone.0014636 (PMC3031500; doi:10.1371/journal.pone.0014636)
Supplement: Table S1 — Protein spots present in only a subset of groups. (0.15 MB PDF) [file pone.0014636.s001.pdf]

Supplemental Table 1. Protein spots present in only a subset of groups[1]

	Line A				Line B			
**SSP**	**Control**	**Acervulina**	**Maxima**	**Tenella**	**Control**	**Acervulina**	**Maxima**	**Tenella**
6801	0	0	0	0	540199	721757	378240	694804
6809	0	0	0	0	419606	959423	646532	702657
6814	0	0	0	0	400978	1192378	467732	592458
8224	0	5480236	0	275665	0	0	41418	0
4522	0	0	0	0	729066	0	313591	156491
6807	0	0	0	0	393711	593029	494869	503459
8421	0	0	0	0	598499	116502	44121	327214
3334	0	0	0	0	191039	580745	85004	0
5127	0	0	0	0	171555	630409	223144	110190
2304	0	78298	52862	0	116363	307600	197192	242374
5327	0	0	0	0	472004	399932	169330	0
8441	0	570396	0	0	0	0	0	0
8233	0	626607	0	0	0	0	0	0
7005	0	33331	0	0	56248	36366	44778	23880
1016	0	0	0	0	25643	0	26052	24016
5101	0	0	0	0	16406	0	59605	0
7221	0	294419	0	0	52647	120838	35583	64996
8025	0	71245	0	0	0	0	0	0
2130	0	0	0	0	47715	121466	24046	92374
1419	0	0	0	0	227496	0	1095760	1016619
3116	0	0	0	0	0	398888	545515	0
5828	0	0	0	0	153458	333831	202518	366102
8003	0	0	0	0	23219	0	109871	0
7001	0	0	0	0	105859	44794	53819	85485
3228	0	0	0	0	74318	51072	30652	34930
5726	0	0	0	0	0	602799	422112	178753
3028	0	26198	0	0	0	0	0	0
5125	0	47031	105997	0	0	0	0	0
6732	0	56445	0	0	0	0	0	0
3027	0	84961	0	0	0	0	0	0
3202	0	100879	0	116557	0	171009	84018	0
8115	0	102615	0	0	0	146037	104066	0
7219	0	151196	0	0	0	0	0	0
7332	0	170546	0	0	0	0	0	0
8026	0	185539	0	0	0	0	0	0
7220	0	226286	0	0	0	0	0	0
7328	0	343358	231755	0	0	0	0	0
9035	0	386185	0	0	0	0	0	0
7130	0	471167	0	0	0	0	0	0
8237	0	522218	0	0	0	0	0	0

8149	0	638465	0	158329	0	0	144456	0
8027	0	919810	0	0	0	0	0	0
8141	0	1069617	0	0	0	0	0	0
8241	0	1974787	0	0	0	0	0	0
7012	0	2038828	0	71406	0	0	19939	0
7418	0	2237627	182504	0	27684	0	37700	0
8148	0	4050928	0	161561	0	0	0	0
8146	0	5791287	0	0	0	0	0	0
7218	0	79660	0	0	0	0	0	0
5134	0	15721	0	0	6570	30743	7602	36492
5131	0	85901	0	44752	59757	96196	19010	0
7111	0	30273	26082	0	13177	162515	23658	12013
6338	0	13590	0	14283	17348	0	11822	0
4519	0	0	0	154457	141373	136066	0	12431
4521	0	0	0	96524	68575	0	0	0
5005	0	0	0	0	52654	66454	32149	50506
8234	0	0	0	0	190337	56323	102485	68752
7801	0	0	0	0	362939	850621	303975	471644
1006	0	0	0	0	52379	159161	66973	69651
2423	0	0	165188	0	595299	682757	325464	699758
8440	0	561006	0	0	0	0	0	0
8439	0	705759	0	0	0	0	0	0
9610	0	1040168	0	195334	0	0	0	0
5712	0	0	0	0	219688	168692	167566	32571
4719	0	0	0	0	172705	726402	77918	34235
5839	0	0	0	0	135073	419441	292339	327153
4312	0	0	0	0	762455	722643	772391	160669
4629	0	0	0	0	368139	610385	178739	160886
9034	0	1458774	0	0	0	0	0	0
4326	0	0	0	0	406354	0	71950	0
5840	0	0	0	0	126730	160532	210640	55176
5647	0	0	0	0	401015	0	154110	0
6810	0	0	0	0	496211	845798	618879	697882
6817	0	0	0	0	414289	747265	521517	564889
6805	0	0	0	0	327006	949396	353541	565237
7805	0	0	0	0	468329	1192067	374193	605668
7544	0	260659	0	755000	1417518	1673395	261969	0
8240	1373	3726800	0	0	0	0	0	0
8236	6427	0	0	94267	31608	45582	32523	55550
6015	7361	442276	0	0	32398	36320	19820	16835
6219	8826	0	25479	0	21552	54407	11615	0
6022	8916	10630	22350	15262	0	0	0	0
3003	9608	95129	27820	20252	15531	15483	17888	0
6202	10494	21137	30687	39736	8098	57373	13030	0
8028	10667	94659	100994	0	36117	84567	11689	25574
7214	10749	73901	0	21089	14655	0	9377	31327
6339	11688	332678	85340	32561	48879	156786	5032	0
5335	11886	0	0	29864	84074	0	18611	110486

5216	12081	0	0	11111	6889	18824	14914	0
6350	12589	0	38604	87456	352515	446070	8027	0
7123	12782	13492	15687	16065	10836	15990	7902	0
5348	13116	0	0	12279	9569	18671	11721	0
7011	13711	0	0	12563	21210	0	12226	38897
6403	14089	79542	76071	76974	62678	121206	24399	0
7215	14573	13721	0	96271	426236	1866029	10824	27440
6222	14797	0	0	0	10522	0	31390	0
7304	15128	90193	0	37798	21707	112359	11378	28346
7211	16112	0	0	18451	25613	32823	21414	10330
8243	16943	335424	41048	56008	0	0	0	0
7330	18980	0	0	26347	45632	21922	25347	92374
2233	19030	142904	0	0	18511	36844	27968	87722
6231	20989	19154	0	90393	20328	0	14220	18024
3010	21383	20913	27979	41527	31110	32372	20796	0
6355	22021	0	30278	53199	167722	0	12131	0
23	22763	69865	45965	32554	107163	0	39168	16814
8334	23397	94503	35280	102822	58290	114013	39689	0
6002	24309	0	38995	44443	68317	74813	45043	17665
7327	24534	55936	0	84440	84441	0	68700	0
2217	26324	0	0	28359	37698	75061	30273	24977
2228	26542	768696	0	21913	27311	30100	24946	27436
6321	26705	13500	0	29846	26907	213641	9303	39511
6348	27794	0	0	26844	18271	15109	10988	0
1018	28189	0	0	0	24396	0	25123	49983
8203	28950	0	37568	86305	38069	168265	17812	35540
6011	29391	0	63160	67745	73604	39900	64098	42916
2316	29905	0	23739	63306	76881	231948	10802	77585
6233	30980	7655	27708	31877	34516	0	17051	0
22	31228	30508	14626	27160	47189	0	28770	55730
7119	31493	0	41348	0	57493	192467	69792	39581
7302	33785	0	0	10746	45964	361029	32449	88739
4826	35277	47998	0	60274	56076	49474	14130	41453
5212	36923	76266	25891	48747	64614	0	25794	32564
6236	37248	0	53487	145382	71077	169796	25954	19479
5330	41736	6539	25875	74964	17408	0	13669	0
8204	43137	0	69754	59331	55146	100550	40285	31384
20	44919	62969	39352	60920	76734	0	63415	71539
2311	48921	165190	0	31380	60623	114148	27763	0
5301	48963	0	0	62845	35143	0	8340	44627
3331	49123	0	0	0	67356	998907	52073	0
4824	50883	25097	0	50064	39542	43545	12387	34959
4340	51162	79489	0	163678	83524	0	76061	25592
8143	51921	242047	0	187237	362546	253487	26982	17079
5326	52560	0	27020	42813	77745	58889	43878	32125
5328	53799	0	0	103340	57399	0	83653	0
4832	56123	9953	0	67759	53375	37960	30021	31652
8244	57564	608103	77227	92578	87652	62553	97259	0

6356	58006	0	159806	86438	74485	120209	69365	32209
6737	58649	36771	0	36018	52970	0	57069	0
5337	62743	0	31852	100167	79025	350777	41027	36530
5322	66658	0	43201	86039	70658	74698	37366	48428
5728	67810	100702	0	0	0	0	0	0
2044	67912	18301	25209	47000	22242	0	41586	61599
5320	68762	0	30917	84996	44654	72720	36480	38116
7301	73052	5708	0	44214	22522	0	9656	0
1022	76655	0	0	0	703503	2846597	0	0
4518	79041	0	0	166617	35156	0	15341	34939
2230	81877	59291	35625	150759	129164	177693	159769	104488
5304	85581	87909	0	98506	223964	132508	64021	93366
6325	85984	0	0	55826	45134	0	15246	40423
2225	91461	61956	58947	113360	170347	218460	105251	35079
8142	97844	166236	0	20140	135285	144128	79879	105148
8330	98735	1370487	110907	179459	0	0	0	0
5646	98777	0	0	0	47736	146859	26027	0
3518	102555	0	31753	298476	48341	145816	83014	61325
6740	102685	84009	0	0	0	0	0	0
6220	105152	83559	0	0	39476	178087	17149	0
5218	106775	21451	0	64668	38147	110280	17707	106107
8323	109280	787486	180774	38521	220365	188515	266115	0
8420	110146	364191	0	0	103528	229369	40635	99777
4633	110756	0	0	170253	50041	50330	0	30965
2224	114998	72093	28459	114191	123586	77248	50716	56456
9412	115027	47646	0	0	358480	91350	190009	161244
5321	119761	0	37504	126829	133013	306284	143058	82929
7333	120061	277142	37177	90625	0	0	0	0
7403	124635	83944	0	71732	28428	0	53213	0
4634	126747	133445	119011	305061	0	0	0	0
5517	146964	198035	0	0	178346	398142	146035	0
5648	150760	150912	0	240315	216686	244166	187985	34681
5111	166284	0	47238	171600	157306	207344	128596	97917
2223	179549	116304	77749	155789	121962	164033	75920	60573
4516	186518	1692363	4882794	0	0	0	0	0
8019	198218	69203	0	0	111736	15547	216731	41376
1635	202186	0	29476	156237	627294	196003	124870	86238
2220	206035	128900	55255	165532	300195	276976	162975	74649
8331	211580	670258	144636	249529	0	0	0	0
2531	243153	0	77258	144399	893073	422	141881	0
6635	254761	77095	0	0	467556	0	201133	495561
2321	258393	0	125938	288787	121747	264617	69347	49791
5613	267800	0	136128	0	170591	0	102660	62130
6434	277672	50279	0	0	220294	349854	107643	0
1636	295453	0	41646	256300	1095156	518540	153593	388674
2232	295564	289768	56553	579855	231306	103548	171097	114332
2630	297081	0	26362	774387	457243	524764	165963	507076
7422	336234	0	0	410042	330602	320830	162771	231347

8442	360551	1111514	127949	455163	0	0	0	0
6524	364572	190795	0	0	179442	230436	59382	0
1423	368060	0	0	408072	122698	0	353155	0
2412	375403	0	108596	1374509	419119	0	209833	0
1642	379524	0	46108	693754	1553515	573879	159232	367830
5618	416556	0	0	0	171217	0	77090	49348
6536	431119	211074	226141	466672	317313	887665	105776	0
9911	482469	0	154054	135161	248185	116724	146942	515098
2410	491767	1396175	127651	735780	516696	357910	75375	0
9714	516777	0	0	0	269815	498798	237122	105403
2218	530544	790685	470445	681624	389935	796696	360511	302727
3229	568303	0	0	0	92796	0	55237	0
8567	621895	1950280	933018	0	2659840	783494	295540	315216
9801	640020	0	165348	62423	89182	189035	293523	319564
6517	689017	131955	147775	774332	61718	322919	18323	0
5649	696182	232528	220121	291668	0	0	0	0
6431	710751	158808	177162	0	167840	405712	73700	0
3333	838956	1141649	488085	1288624	538318	524025	432772	1644471
9802	906880	0	131064	100025	438256	212825	206664	285383
5636	1033509	337258	442022	0	1683911	1656620	1197519	0
7501	1290469	357198	0	910454	357717	334916	1893220	131020
5128	1316705	712428	0	0	547967	0	0	432744
7503	1485373	0	0	1406659	1645156	802974	1343698	1203304
2222	1740789	2063465	498549	1808678	1596456	1461442	1423100	905000
9712	2028179	0	0	0	908892	642241	1519065	867634
1528	2336925	0	376768	2109173	2729742	1154026	1892639	585474
1529	3376938	0	1197140	5380353	2165168	3467656	0	1158478

[1]Protein spots that were absent in some groups but present in others. SSP = unique protein identifier. LSmeans are shown for spot density data, separated into treatment group within genetic line
