# Supplementary material for: Eimeria Species and Genetic Background Influence the Serum Protein Profile of Broilers with Coccidiosis
Source: PLoS One. 2011 Jan 31;6(1):e14636. doi: 10.1371/journal.pone.0014636 (PMC3031500; doi:10.1371/journal.pone.0014636)
Supplement: Table S2 — Information for all proteins that were identified. (0.07 MB PDF) [file pone.0014636.s002.pdf]

Supplemental Table 2. Information for all identified proteins

Protein name	Accession ID[1]	Predicted MW (kDa)	Observed MW (kDa)[2]	Predicted pI	Observed pI[3]	SSP[4]	Protein probability[5]	Number of peptides[6]
actin, cytoplasmic	gi|45382927, gi|56119084	41.7	63.7	5.5	4.5	1519	100%	1
actin, cytoplasmic		41.7	42.6	5.5	5.6	3318	100%	6
actin, cytoplasmic		41.7	42.8	5.5	5.6	3323	100%	7
amylase, alpha 2A; pancreatic	gi|47825395	57.5	54.8	6.1	5.8	5403	100%	2
amylase, alpha 2A; pancreatic		57.5	208.3	6.1	6.4	7812	100%	1
apolipoprotein A-I preprotein	gi|45382961	30.7	26.0	5.7	3.0	111	100%	2
apolipoprotein A-I preprotein		30.7	64.7	5.7	3.0	421	100%	1
apolipoprotein A-I preprotein		30.7	26.4	5.7	5.0	3102	100%	5
apolipoprotein A-I preprotein		30.7	25.8	5.7	5.1	3108	100%	8
apolipoprotein A-I preprotein		30.7	26.8	5.7	5.6	3117	100%	2
apolipoprotein A-I preprotein		30.7	26.8	5.7	5.6	3117	100%	5
apolipoprotein A-I preprotein		30.7	26.7	5.7	5.6	4120	100%	2
apolipoprotein A-I preprotein		30.7	26.7	5.7	5.6	4120	100%	4
apolipoprotein A-I preprotein		30.7	26.9	5.7	5.9	6116	100%	1
apolipoprotein A-I preprotein		30.7	38.3	5.7	6.4	7206	100%	1
apolipoprotein A-IV	gi|45384392	40.9	35.5	4.9	4.7	2205	100%	4
ATP synthase subunit alpha, mitochondrial	gi|45383566	60.2	56.0	9.3	7.0	8415	100%	2
ATP synthase subunit beta, mitochondrial precursor	gi|71897237	56.6	51.3	5.9	4.9	3304	99%	2
calcium-binding mitochondrial carrier protein Aralar2	gi|61098440	74.1	75.3	8.8	8.0	8569	100%	2
carbonic anhydrase 2	gi|46048696	29.3	29.1	7.2	6.4	7114	91%	1
collagen alpha-1(II) chain	gi|45383309	135.0	32.8	8.3	5.8	5205	91%	1
complement C3 precursor	gi|45382303	184.1	137.4	7.1	5.6	3852	100%	2
complement C3 precursor		184.1	120.9	7.1	5.8	5710	100%	2
complement C3 precursor		184.1	120.3	7.1	5.8	5713	100%	4
complement C3 precursor		184.1	123.4	7.1	5.9	5720	100%	2
complement C3 precursor		184.1	123.0	7.1	5.8	5724	100%	1
complement C3 precursor		184.1	74.8	7.1	7.4	8549	100%	2
fetuin B	gi|50752383	44.1	51.8	5.8	5.0	3311	100%	3
fetuin B		44.1	52.7	5.8	5.0	3313	100%	3
fetuin B		44.1	52.2	5.8	5.6	3317	100%	5
fetuin B		44.1	52.5	5.8	5.6	3324	100%	3
fetuin B		44.1	53.2	5.8	5.0	3403	100%	1
fetuin B		44.1	52.9	5.8	5.5	3412	100%	2
fructose-bisphosphate aldolase C	gi|226855	38.6	42.8	6.1	5.9	6316	77%	1
gelsolin precursor	gi|45384386	85.8	84.2	6.3	5.8	5617	100%	1
gelsolin precursor		85.8	84.2	6.3	5.8	5620	100%	4
gelsolin precursor		85.8	85.5	6.3	5.9	6624	100%	1

glutathione peroxidase 3	gi|253735708	24.6	25.2	8.6	6.9	8110	100%	1
glutathione peroxidase 3		24.6	25.2	8.6	7.5	8126	100%	4
glutathione peroxidase 3		24.6	25.3	8.6	8.0	8139	100%	2
hemoglobin subunit alpha-A	gi|52138655	15.4	11.2	8.5	6.7	8003	100%	1
hemoglobin subunit alpha-A		15.4	11.1	8.5	7.2	8012	100%	3
hemoglobin subunit alpha-A		15.4	11.8	8.5	7.7	8017	100%	3
hemoglobin subunit alpha-D	gi|52138645	15.7	13.0	7.6	6.7	8006	91%	1
hemoglobin subunit beta	gi|49169791	16.5	15.1	8.6	8.0	8018	100%	2
hemopexin	gi|16805334	29.4	58.7	6.4	4.9	2408	100%	2
hemopexin		29.4	58.7	6.4	4.9	2408	100%	1
hemopexin		29.4	60.8	6.4	9.4	9406	100%	1
hemopexin		29.4	62.6	6.4	9.5	9410	100%	1
Ig gamma chain	gi|86318	53.6	74.8	7.1	5.9	6518	91%	1
Ig light chain	gi|212066	14.8	26.8	5.0	5.8	5107	80%	1
Ig mu chain C region	gi|127513	48.2	73.4	6.4	5.7	4510	88%	1
NADH dehydrogenase [ubiquinone] 1 alpha subcomplex subunit 9, mitochondrial	gi|57529307	43.1	35.9	9.4	8.5	8240	100%	4
ovoinhibitor precursor	gi|71895337	51.9	56.8	6.6	5.7	4410	100%	6
ovoinhibitor precursor		51.9	58.3	6.6	5.7	4411	100%	1
ovoinhibitor precursor		51.9	58.3	6.6	5.7	4411	100%	4
ovoinhibitor precursor		51.9	60.9	6.6	5.7	5401	100%	1
ovoinhibitor precursor		51.9	58.8	6.6	5.8	5402	100%	2
ovoinhibitor precursor		51.9	60.9	6.6	5.8	5406	100%	1
ovoinhibitor precursor		51.9	58.4	6.6	5.8	5407	100%	2
ovoinhibitor precursor		51.9	58.7	6.6	5.8	5413	100%	2
ovoinhibitor precursor		51.9	59.0	6.6	5.8	5418	100%	1
ovoinhibitor precursor		51.9	57.0	6.6	5.9	6406	100%	1
ovotransferrin	gi|71274075, gi|71274079, etc.	77.8 / 77.6	75.8	7.12 / 7.25	3.0	1502	100%	3
ovotransferrin		77.8 / 77.6	78.8	7.12 / 7.25	5.9	6542	100%	1
ovotransferrin		77.8 / 77.6	83.4	7.12 / 7.25	5.9	6612	100%	1
ovotransferrin		77.8 / 77.6	76.1	7.12 / 7.25	6.3	7512	100%	4
ovotransferrin		77.8 / 77.6	79.1	7.12 / 7.25	6.3	7519	100%	1
ovotransferrin		77.8 / 77.6	76.0	7.12 / 7.25	6.3	7520	100%	5
ovotransferrin		77.8 / 77.6	79.7	7.12 / 7.25	6.4	7543	100%	5
ovotransferrin		77.8 / 77.6	79.7	7.12 / 7.25	6.4	7543	100%	1

ovotransferrin		77.8 / 77.6	78.4	7.12 / 7.25	6.8	8560	100%	4
PIT 54 protein	gi|46395491	50.8	56.4	4.7	4.6	1422	100%	2
PREDICTED: hypothetical protein	gi|50749164	24.5	29.5	4.9	4.7	2103	91%	1
PREDICTED: hypothetical protein	gi|50749164	24.5	29.4	4.9	4.7	2107	91%	1
PREDICTED: similar to 2,4-dienoyl-CoA reductase	gi|50731694	35.7	34.4	9.4	8.2	8224	91%	1
PREDICTED: similar to alpha-1-antitrypsin	gi|118091960	43.1	48.3	5.6	5.7	3329	100%	1
PREDICTED: similar to alpha-1-antitrypsin		43.1	52.4	5.6	5.6	4306	100%	1
PREDICTED: similar to alpha-1-antitrypsin		43.1	51.2	5.6	5.7	4310	100%	2
PREDICTED: similar to alpha-1-antitrypsin		43.1	52.1	5.6	5.7	4313	100%	2
PREDICTED: similar to alpha-1-antitrypsin		43.1	50.0	5.6	5.7	4316	100%	1
PREDICTED: similar to alpha-1-antitrypsin		43.1	49.1	5.6	5.7	4320	100%	2
PREDICTED: similar to alpha-1-antitrypsin		43.1	53.1	5.6	5.7	4403	100%	4
PREDICTED: similar to alpha-2-macroglobulin	gi|118083276	186.8	203.3	5.6	4.7	1832	100%	2
PREDICTED: similar to alpha-2-macroglobulin		186.8	194.0	5.6	4.7	2909	100%	1
PREDICTED: similar to alpha-2-macroglobulin		186.8	205.7	5.6	6.8	8834	100%	1
PREDICTED: similar to Alpha-2-macroglobulin precursor	gi|118083282	162.8	183.1	6.5	3.0	1802	100%	2
PREDICTED: similar to Alpha-2-macroglobulin precursor		162.8	93.9	6.5	5.9	5636	100%	2
PREDICTED: similar to Alpha-2-macroglobulin precursor		162.8	93.8	6.5	5.8	5645	100%	2
PREDICTED: similar to Alpha-2-macroglobulin precursor		162.8	186.0	6.5	5.8	5801	100%	1
PREDICTED: similar to Alpha-2-macroglobulin precursor		162.8	173.4	6.5	5.8	5840	100%	2
PREDICTED: similar to Alpha-2-macroglobulin precursor		162.8	203.8	6.5	6.7	8833	100%	4
PREDICTED: similar to antithrombin	gi|118094218	41.8	57.2	7.7	4.9	2429	1	3
PREDICTED: similar to antithrombin		41.8	58.3723	7.7	4.9708	3405	1	4
PREDICTED: similar to antithrombin		41.8	57.8	7.7	5	3410	1	3
PREDICTED: similar to fetuin	gi|50752381	37.2	45.2728	6.21	4.5978	1316	1	4
PREDICTED: similar to fetuin		37.2	35	6.21	5	1323	1	1
PREDICTED: similar to fetuin		37.2	46.5883	6.21	4.5161	1324	1	2
PREDICTED: similar to fetuin		37.2	44.2412	6.21	4.6823	2302	1	2
PREDICTED: similar to inter-alpha (globulin) inhibitor H3	gi|118096699	99.9	114.9501	6.23	4.6111	1726	0.999	2
PREDICTED: similar to inter-alpha (globulin) inhibitor H3		99.9	126.1	6.23	6.5	7736	0.999	3
PREDICTED: similar to K12 keratin	gi|118102980	54.1	23.5	4.93	5.7	3029	0.998	1
PREDICTED: similar to K12 keratin		54.1	32	4.93	5.7	4215	0.998	1
PREDICTED: similar to K12 keratin		54.1	51.4	4.93	5.7	4338	0.998	1
PREDICTED: similar to K12 keratin		54.1	73.1715	4.93	5.7673	5504	0.998	1
PREDICTED: similar to K12 keratin		54.1	107.9933	4.93	6.4636	6724	0.998	2
PREDICTED: similar to malate dehydrogenase 2, NAD (mitochondrial)	gi|50758110	37	35.9	8.56	7.4	8233	1	5
PREDICTED: similar to malate dehydrogenase 2, NAD (mitochondrial)		37	35.9	8.56	7	8237	1	1

PREDICTED: similar to plasminogen	gi|118088308	90.8	111.5	7.66	6.3	7715	0.974	2
PREDICTED: similar to plasminogen		90.8	109.6	7.66	6.8	8738	1	3
PREDICTED: similar to Serpina1d-prov protein	gi|118091958	47.7	53.9342	7.2	5.7693	4342	1	1
PREDICTED: similar to Serpina1d-prov protein		47.7	56.8	7.2	5.7	4408	1	1
PREDICTED: similar to Serpina1d-prov protein		47.7	56.8	7.2	5.7	4408	1	1
PREDICTED: similar to Serpina1d-prov protein		47.7	57.0732	7.2	5.7246	4426	1	1
PREDICTED: similar to Serpina1d-prov protein		47.7	57.0505	7.2	5.7317	4427	1	4
PREDICTED: similar to Serpina1d-prov protein		47.7	53.2863	7.2	5.8045	5344	1	1
PREDICTED: similar to Serpina1d-prov protein		47.7	54.7701	7.2	5.7635	5403	1	1
PREDICTED: similar to Serpina1d-prov protein		47.7	56.6681	7.2	5.7668	5404	1	3
PREDICTED: similar to thiolase-prov protein	gi|50745166	50.8	51.4	9.31	8.2	9311	1	3
PREDICTED: similar to vanin 1	gi|118088529	54.6	64.8933	5.42	4.6892	2518	0.999	3
putative keratin 4	gi|118129660	58.9	31.1	7.42	6	6129	0.979	1
putative mitochondrial ATP synthase O (delta) subunit	gi|118083809	22.8	23.9	9.88	9	9034	0.998	2
retinol-binding protein 4 precursor	gi|45382541	22.5	23.6985	6.34	5.7891	5007	0.981	3
serum albumin precursor	gi|45383974	69.9	64.6707	5.74	3	421	1	2
serum albumin precursor		69.9	49.0104	5.74	3	1304	1	1
serum albumin precursor		69.9	59.9	5.74	3	1402	1	1
serum albumin precursor		69.9	62.9	5.74	3	1403	1	4
serum albumin precursor		69.9	55.3932	5.74	3	1404	1	1
serum albumin precursor		69.9	64.4	5.74	3.2	1515	1	4
serum albumin precursor		69.9	67.742	5.74	5.0162	2419	1	4
serum albumin precursor		69.9	67.9652	5.74	4.975	2420	1	3
serum albumin precursor		69.9	61.6	5.74	5	2426	1	1
serum albumin precursor		69.9	71.4694	5.74	4.8539	2514	1	1
serum albumin precursor		69.9	63.6	5.74	4.7	2520	1	5
serum albumin precursor		69.9	63.6	5.74	4.7	2521	1	1
serum albumin precursor		69.9	63.6	5.74	4.7	2521	1	5
serum albumin precursor		69.9	35.3183	5.74	5.0574	3212	1	5
serum albumin precursor		69.9	35.304	5.74	5.6071	3225	1	1
serum albumin precursor		69.9	55	5.74	5	3408	1	4
serum albumin precursor		69.9	57.8	5.74	5	3410	1	2
serum albumin precursor		69.9	66.2	5.74	5.6	3424	1	4
serum albumin precursor		69.9	67.7	5.74	5.1	3425	1	4
serum albumin precursor		69.9	75.3373	5.74	5.1987	3520	1	1
serum albumin precursor		69.9	233.3747	5.74	5.6066	3920	1	3
serum albumin precursor		69.9	224.931	5.74	5.6163	3924	1	3
serum albumin precursor		69.9	51.9	5.74	5.7	4319	1	4
serum albumin precursor		69.9	56.8	5.74	5.7	4408	1	2

serum albumin precursor		69.9	65.5	5.74	5.8	4429	1	5
serum albumin precursor		69.9	75.8292	5.74	5.6381	4512	1	2
serum albumin precursor		69.9	60.9	5.74	5.7	5401	1	3
serum albumin precursor		69.9	67.2	5.74	5.8	5520	1	4
serum albumin precursor		69.9	63.2652	5.74	5.9629	6526	1	2
serum albumin precursor		69.9	70.008	5.74	5.9794	6540	1	5
serum albumin precursor		69.9	68.6053	5.74	5.9238	6543	1	4
serum albumin precursor		69.9	68.3582	5.74	5.9448	6544	1	3
serum albumin precursor		69.9	69.6	5.74	6	7505	1	5
serum albumin precursor		69.9	74	5.74	6.2	7508	1	4
serum albumin precursor		69.9	70.7338	5.74	6.3559	7522	1	4
serum albumin precursor		69.9	65.985	5.74	9.4001	9508	1	5
similar to alpha-2-macroglobulin	hmm36420	159.2	94.5649	6.14	4.9927	2626	1	1
similar to alpha-2-macroglobulin		159.2	94.5649	6.14	4.9927	2626	1	4
similar to alpha-2-macroglobulin		159.2	93.4108	6.14	4.9702	2627	1	3
similar to alpha-2-macroglobulin		159.2	90.5518	6.14	5.0956	3612	1	3
similar to alpha-2-macroglobulin		159.2	90.816	6.14	5.6053	3627	1	2
similar to alpha-2-macroglobulin		159.2	96.4807	6.14	5.6759	4623	1	5
similar to alpha-2-macroglobulin		159.2	93.3008	6.14	5.8784	6605	1	2
similar to alpha-2-macroglobulin		159.2	93.1	6.14	5.9	6611	1	5
similar to alpha-2-macroglobulin		159.2	92.6218	6.14	5.9031	6633	1	1
similar to alpha-2-macroglobulin		159.2	94.6064	6.14	5.9227	6637	1	1
similar to alpha-2-macroglobulin		159.2	94.6064	6.14	5.9227	6637	1	4
similar to alpha-2-macroglobulin		159.2	96.2303	6.14	5.9888	7601	1	6
similar to alpha-2-macroglobulin		159.2	93.9382	6.14	6.0135	7604	1	6
similar to alpha-2-macroglobulin		159.2	96.7572	6.14	6.335	7615	1	1
similar to alpha-2-macroglobulin		159.2	95.2801	6.14	6.4488	7622	1	1
similar to alpha-2-macroglobulin		159.2	96.5044	6.14	6.4757	8622	1	1
similar to alpha-2-macroglobulin		159.2	96.5044	6.14	6.4757	8622	1	6
similar to alpha-2-macroglobulin		159.2	95.9	6.14	6.8	8632	1	6
transthyretin precursor	gi|45384444	16.3	15.0644	5.24	4.8419	2015	1	3
transthyretin precursor		16.3	15.3563	5.24	4.8467	2016	1	1
transthyretin precursor		16.3	15.3178	5.24	5.0116	2041	1	5
vitamin D-binding protein	gi|45382425	53.7	56.9092	6.87	5.7921	5409	1	5
vitamin D-binding protein		53.7	56.6744	6.87	5.827	5415	1	5
vitamin D-binding protein		53.7	56.9897	6.87	5.8795	6406	1	2
vitamin D-binding protein		53.7	56.4187	6.87	5.9007	6408	1	4
vitamin D-binding protein		53.7	56.7946	6.87	5.943	6411	1	3
vitelline membrane outer layer protein 1	gi|268370086	20.2	22.8	8.48	4.6	2002	0.912	1
vitelline membrane outer layer protein 1		20.2	23.3301	8.48	4.6313	2003	0.912	1
vitronectin	gi|46048795	51.6	63.8	5.35	4.6	1524	0.997	2

[1]gi (general information identifier) is a unique identifier for a particular sequence. Each time a sequence is updated at NCBI it is given a new gi number.
hmm: hidden markove model protein family sequence identifier

[2]The observed molecular weight as determined from 2DE

[3]The observed isoelectric point as determined from 2DE

[4]Unique identifier for each protein spot. Multiple proteins identified in 421, 3410, 4408, 5403 and 6406

[5]Probability of protein identification being correct based upon Scaffold algorithm combining Mascot and X! Tandem search results.

[6]Number of unique peptides for which the amino acid sequence matches the listed protein ID and the amino acid sequence was confirmed by MSMS.
