# Supplementary material for: Eimeria Species and Genetic Background Influence the Serum Protein Profile of Broilers with Coccidiosis
Source: PLoS One. 2011 Jan 31;6(1):e14636. doi: 10.1371/journal.pone.0014636 (PMC3031500; doi:10.1371/journal.pone.0014636)
Supplement: Table S3 — Least squares means for effects of broiler line, coccidia infection and the interaction of broiler line×coccidia infection on spot density. (1.69 MB PDF) [file pone.0014636.s003.pdf]

Supplemental Table 3. Least Squares Means for main effects of broiler line, coccidia infection group and the interaction of broiler line x coccidia infection group on protein spot density.

Protein #	Effect	Line[1]	Coccidia[2]	Estimate[3]	StdErr[4]	DF[5]
14	line	1		455831.3	76899.87	40
14	line	2		878567.8	76899.87	40
14	coccidia		0	1029002	108752.8	40
14	coccidia		1	445347.8	108752.8	40
14	coccidia		2	780088.4	108752.8	40
14	coccidia		3	414360.6	108752.8	40
14	line*coccidia	1	0	582288	153799.7	40
14	line*coccidia	1	1	315081.5	153799.7	40
14	line*coccidia	1	2	164634.8	153799.7	40
14	line*coccidia	1	3	761321	153799.7	40
14	line*coccidia	2	0	1475715	153799.7	40
14	line*coccidia	2	1	575614	153799.7	40
14	line*coccidia	2	2	1395542	153799.7	40
14	line*coccidia	2	3	67400.17	153799.7	40
16	line	1		324207	38766.42	40
16	line	2		324128.3	38766.42	40
16	coccidia		0	487086	54823.99	40
16	coccidia		1	168088.7	54823.99	40
16	coccidia		2	221329.6	54823.99	40
16	coccidia		3	420166.3	54823.99	40
16	line*coccidia	1	0	491072	77532.83	40
16	line*coccidia	1	1	167030	77532.83	40
16	line*coccidia	1	2	126303.8	77532.83	40
16	line*coccidia	1	3	512422	77532.83	40
16	line*coccidia	2	0	483100	77532.83	40
16	line*coccidia	2	1	169147.3	77532.83	40
16	line*coccidia	2	2	316355.3	77532.83	40
16	line*coccidia	2	3	327910.5	77532.83	40
17	line	1		4089566	334360.4	40
17	line	2		3352466	334360.4	40
17	coccidia		0	5378774	472857	40
17	coccidia		1	1919145	472857	40
17	coccidia		2	2741945	472857	40
17	coccidia		3	4844200	472857	40
17	line*coccidia	1	0	5873594	668720.8	40
17	line*coccidia	1	1	2015085	668720.8	40
17	line*coccidia	1	2	1903272	668720.8	40
17	line*coccidia	1	3	6566314	668720.8	40
17	line*coccidia	2	0	4883954	668720.8	40
17	line*coccidia	2	1	1823204	668720.8	40
17	line*coccidia	2	2	3580619	668720.8	40
17	line*coccidia	2	3	3122087	668720.8	40
18	line	1		303618.3	48058.73	40
18	line	2		292975.9	48058.73	40

18	coccidia		0	390641.8	67965.3	40
18	coccidia		1	201289.1	67965.3	40
18	coccidia		2	262111	67965.3	40
18	coccidia		3	339146.6	67965.3	40
18	line*coccidia	1	0	312103.7	96117.46	40
18	line*coccidia	1	1	278080.3	96117.46	40
18	line*coccidia	1	2	96040.83	96117.46	40
18	line*coccidia	1	3	528248.5	96117.46	40
18	line*coccidia	2	0	469179.8	96117.46	40
18	line*coccidia	2	1	124497.8	96117.46	40
18	line*coccidia	2	2	428181.2	96117.46	40
18	line*coccidia	2	3	150044.7	96117.46	40
20	line	1		52039.71	10385.7	40
20	line	2		52921.88	10385.7	40
20	coccidia		0	60826.17	14687.6	40
20	coccidia		1	31484.67	14687.6	40
20	coccidia		2	51383.25	14687.6	40
20	coccidia		3	66229.08	14687.6	40
20	line*coccidia	1	0	44918.5	20771.41	40
20	line*coccidia	1	1	62969.33	20771.41	40
20	line*coccidia	1	2	39351.5	20771.41	40
20	line*coccidia	1	3	60919.5	20771.41	40
20	line*coccidia	2	0	76733.83	20771.41	40
20	line*coccidia	2	1	0	20771.41	40
20	line*coccidia	2	2	63415	20771.41	40
20	line*coccidia	2	3	71538.67	20771.41	40
22	line	1		25880.5	4843.365	40
22	line	2		32922.25	4843.365	40
22	coccidia		0	39208.67	6849.552	40
22	coccidia		1	15254.08	6849.552	40
22	coccidia		2	21697.67	6849.552	40
22	coccidia		3	41445.08	6849.552	40
22	line*coccidia	1	0	31228	9686.729	40
22	line*coccidia	1	1	30508.17	9686.729	40
22	line*coccidia	1	2	14625.83	9686.729	40
22	line*coccidia	1	3	27160	9686.729	40
22	line*coccidia	2	0	47189.33	9686.729	40
22	line*coccidia	2	1	0	9686.729	40
22	line*coccidia	2	2	28769.5	9686.729	40
22	line*coccidia	2	3	55730.17	9686.729	40
23	line	1		42786.75	12598.26	40
23	line	2		40786.08	12598.26	40
23	coccidia		0	64962.83	17816.63	40
23	coccidia		1	34932.58	17816.63	40
23	coccidia		2	42566.58	17816.63	40
23	coccidia		3	24683.67	17816.63	40
23	line*coccidia	1	0	22763	25196.52	40
23	line*coccidia	1	1	69865.17	25196.52	40
23	line*coccidia	1	2	45965.33	25196.52	40

23	line*coccidia	1	3	32553.5	25196.52	40
23	line*coccidia	2	0	107162.7	25196.52	40
23	line*coccidia	2	1	3.64E-12	25196.52	40
23	line*coccidia	2	2	39167.83	25196.52	40
23	line*coccidia	2	3	16813.83	25196.52	40
26	line	1		1920759	129602.6	40
26	line	2		808403.2	129602.6	40
26	coccidia		0	1616460	183285.8	40
26	coccidia		1	649629.5	183285.8	40
26	coccidia		2	693801.6	183285.8	40
26	coccidia		3	2498434	183285.8	40
26	line*coccidia	1	0	2378563	259205.2	40
26	line*coccidia	1	1	1059385	259205.2	40
26	line*coccidia	1	2	945899.3	259205.2	40
26	line*coccidia	1	3	3299190	259205.2	40
26	line*coccidia	2	0	854357.3	259205.2	40
26	line*coccidia	2	1	239874.3	259205.2	40
26	line*coccidia	2	2	441703.8	259205.2	40
26	line*coccidia	2	3	1697677	259205.2	40
111	line	1		22540675	1324554	40
111	line	2		13948497	1324554	40
111	coccidia		0	28968937	1873203	40
111	coccidia		1	7288614	1873203	40
111	coccidia		2	12251714	1873203	40
111	coccidia		3	24469078	1873203	40
111	line*coccidia	1	0	38954962	2649109	40
111	line*coccidia	1	1	7583494	2649109	40
111	line*coccidia	1	2	6923952	2649109	40
111	line*coccidia	1	3	36700290	2649109	40
111	line*coccidia	2	0	18982913	2649109	40
111	line*coccidia	2	1	6993734	2649109	40
111	line*coccidia	2	2	17579477	2649109	40
111	line*coccidia	2	3	12237866	2649109	40
112	line	1		583096.7	64502.86	40
112	line	2		401010.6	64502.86	40
112	coccidia		0	460355.8	91220.82	40
112	coccidia		1	554005.3	91220.82	40
112	coccidia		2	363700.3	91220.82	40
112	coccidia		3	590153.2	91220.82	40
112	line*coccidia	1	0	539271.2	129005.7	40
112	line*coccidia	1	1	732553.8	129005.7	40
112	line*coccidia	1	2	340867	129005.7	40
112	line*coccidia	1	3	719694.7	129005.7	40
112	line*coccidia	2	0	381440.3	129005.7	40
112	line*coccidia	2	1	375456.8	129005.7	40
112	line*coccidia	2	2	386533.5	129005.7	40
112	line*coccidia	2	3	460611.7	129005.7	40
211	line	1		524495.8	47317.86	40
211	line	2		406885	47317.86	40

211	coccidia		0	460922.3	66917.57	40
211	coccidia		1	430688	66917.57	40
211	coccidia		2	379648	66917.57	40
211	coccidia		3	591503.3	66917.57	40
211	line*coccidia	1	0	406647.2	94635.73	40
211	line*coccidia	1	1	648042.5	94635.73	40
211	line*coccidia	1	2	290512.2	94635.73	40
211	line*coccidia	1	3	752781.2	94635.73	40
211	line*coccidia	2	0	515197.5	94635.73	40
211	line*coccidia	2	1	213333.5	94635.73	40
211	line*coccidia	2	2	468783.8	94635.73	40
211	line*coccidia	2	3	430225.3	94635.73	40
421	line	1		4471813	557690.2	40
421	line	2		5185458	557690.2	40
421	coccidia		0	7415492	788693	40
421	coccidia		1	2854721	788693	40
421	coccidia		2	4426343	788693	40
421	coccidia		3	4617985	788693	40
421	line*coccidia	1	0	8709842	1115380	40
421	line*coccidia	1	1	2868346	1115380	40
421	line*coccidia	1	2	2951679	1115380	40
421	line*coccidia	1	3	3357385	1115380	40
421	line*coccidia	2	0	6121142	1115380	40
421	line*coccidia	2	1	2841097	1115380	40
421	line*coccidia	2	2	5901007	1115380	40
421	line*coccidia	2	3	5878585	1115380	40
824	line	1		845366.1	122239.3	40
824	line	2		584894.2	122239.3	40
824	coccidia		0	549916.7	172872.5	40
824	coccidia		1	572762.4	172872.5	40
824	coccidia		2	877518	172872.5	40
824	coccidia		3	860323.5	172872.5	40
824	line*coccidia	1	0	360224.8	244478.7	40
824	line*coccidia	1	1	911462.5	244478.7	40
824	line*coccidia	1	2	823749	244478.7	40
824	line*coccidia	1	3	1286028	244478.7	40
824	line*coccidia	2	0	739608.5	244478.7	40
824	line*coccidia	2	1	234062.3	244478.7	40
824	line*coccidia	2	2	931287	244478.7	40
824	line*coccidia	2	3	434619	244478.7	40
915	line	1		737766.9	104952.3	40
915	line	2		642318.5	104952.3	40
915	coccidia		0	421874.6	148425	40
915	coccidia		1	420024.8	148425	40
915	coccidia		2	653769.9	148425	40
915	coccidia		3	1264501	148425	40
915	line*coccidia	1	0	324184	209904.6	40
915	line*coccidia	1	1	749830.8	209904.6	40
915	line*coccidia	1	2	602357.3	209904.6	40

915	line*coccidia	1	3	1274695	209904.6	40
915	line*coccidia	2	0	519565.2	209904.6	40
915	line*coccidia	2	1	90218.83	209904.6	40
915	line*coccidia	2	2	705182.5	209904.6	40
915	line*coccidia	2	3	1254307	209904.6	40
916	line	1		2515062	438179.5	40
916	line	2		1913433	438179.5	40
916	coccidia		0	3667388	619679.4	40
916	coccidia		1	1615623	619679.4	40
916	coccidia		2	680389	619679.4	40
916	coccidia		3	2893591	619679.4	40
916	line*coccidia	1	0	4027883	876359	40
916	line*coccidia	1	1	1212898	876359	40
916	line*coccidia	1	2	557109.5	876359	40
916	line*coccidia	1	3	4262358	876359	40
916	line*coccidia	2	0	3306893	876359	40
916	line*coccidia	2	1	2018348	876359	40
916	line*coccidia	2	2	803668.5	876359	40
916	line*coccidia	2	3	1524823	876359	40
1005	line	1		127391.7	20400.69	40
1005	line	2		186652	20400.69	40
1005	coccidia		0	173535.8	28850.93	40
1005	coccidia		1	154283.7	28850.93	40
1005	coccidia		2	106700.9	28850.93	40
1005	coccidia		3	193567.2	28850.93	40
1005	line*coccidia	1	0	139299	40801.37	40
1005	line*coccidia	1	1	68342.33	40801.37	40
1005	line*coccidia	1	2	74610.17	40801.37	40
1005	line*coccidia	1	3	227315.3	40801.37	40
1005	line*coccidia	2	0	207772.5	40801.37	40
1005	line*coccidia	2	1	240225	40801.37	40
1005	line*coccidia	2	2	138791.7	40801.37	40
1005	line*coccidia	2	3	159819	40801.37	40
1006	line	1		1.46E-11	13993.23	40
1006	line	2		87040.88	13993.23	40
1006	coccidia		0	26189.33	19789.42	40
1006	coccidia		1	79580.33	19789.42	40
1006	coccidia		2	33486.42	19789.42	40
1006	coccidia		3	34825.67	19789.42	40
1006	line*coccidia	1	0	1.46E-11	27986.47	40
1006	line*coccidia	1	1	1.46E-11	27986.47	40
1006	line*coccidia	1	2	1.46E-11	27986.47	40
1006	line*coccidia	1	3	1.46E-11	27986.47	40
1006	line*coccidia	2	0	52378.67	27986.47	40
1006	line*coccidia	2	1	159160.7	27986.47	40
1006	line*coccidia	2	2	66972.83	27986.47	40
1006	line*coccidia	2	3	69651.33	27986.47	40
1010	line	1		40235.5	7696.829	40
1010	line	2		58107.33	7696.829	40

1010	coccidia		0	34866.08	10884.96	40
1010	coccidia		1	42545.75	10884.96	40
1010	coccidia		2	33867.83	10884.96	40
1010	coccidia		3	85406	10884.96	40
1010	line*coccidia	1	0	23005.67	15393.66	40
1010	line*coccidia	1	1	53183.67	15393.66	40
1010	line*coccidia	1	2	25639.83	15393.66	40
1010	line*coccidia	1	3	59112.83	15393.66	40
1010	line*coccidia	2	0	46726.5	15393.66	40
1010	line*coccidia	2	1	31907.83	15393.66	40
1010	line*coccidia	2	2	42095.83	15393.66	40
1010	line*coccidia	2	3	111699.2	15393.66	40
1011	line	1		68748.08	13379.19	40
1011	line	2		87869.21	13379.19	40
1011	coccidia		0	88030.92	18921.03	40
1011	coccidia		1	66994.67	18921.03	40
1011	coccidia		2	54157.25	18921.03	40
1011	coccidia		3	104051.8	18921.03	40
1011	line*coccidia	1	0	55795.67	26758.38	40
1011	line*coccidia	1	1	58363.5	26758.38	40
1011	line*coccidia	1	2	62910.5	26758.38	40
1011	line*coccidia	1	3	97922.67	26758.38	40
1011	line*coccidia	2	0	120266.2	26758.38	40
1011	line*coccidia	2	1	75625.83	26758.38	40
1011	line*coccidia	2	2	45404	26758.38	40
1011	line*coccidia	2	3	110180.8	26758.38	40
1013	line	1		957401.6	112930.1	40
1013	line	2		700081	112930.1	40
1013	coccidia		0	845027.8	159707.2	40
1013	coccidia		1	836055.2	159707.2	40
1013	coccidia		2	636208.7	159707.2	40
1013	coccidia		3	997673.4	159707.2	40
1013	line*coccidia	1	0	907602.3	225860.1	40
1013	line*coccidia	1	1	733056.2	225860.1	40
1013	line*coccidia	1	2	480816.2	225860.1	40
1013	line*coccidia	1	3	1708132	225860.1	40
1013	line*coccidia	2	0	782453.3	225860.1	40
1013	line*coccidia	2	1	939054.2	225860.1	40
1013	line*coccidia	2	2	791601.2	225860.1	40
1013	line*coccidia	2	3	287215.2	225860.1	40
1014	line	1		14682105	812882	40
1014	line	2		10091697	812882	40
1014	coccidia		0	16353501	1149589	40
1014	coccidia		1	10910132	1149589	40
1014	coccidia		2	7490255	1149589	40
1014	coccidia		3	14793717	1149589	40
1014	line*coccidia	1	0	18350763	1625764	40
1014	line*coccidia	1	1	10740285	1625764	40
1014	line*coccidia	1	2	6489495	1625764	40

1014	line*coccidia	1	3	23147878	1625764	40
1014	line*coccidia	2	0	14356240	1625764	40
1014	line*coccidia	2	1	11079980	1625764	40
1014	line*coccidia	2	2	8491014	1625764	40
1014	line*coccidia	2	3	6439555	1625764	40
1015	line	1		50047.21	8168.769	40
1015	line	2		111766.7	8168.769	40
1015	coccidia		0	74911.25	11552.38	40
1015	coccidia		1	52374.5	11552.38	40
1015	coccidia		2	92843.67	11552.38	40
1015	coccidia		3	103498.4	11552.38	40
1015	line*coccidia	1	0	51851.67	16337.54	40
1015	line*coccidia	1	1	22065.67	16337.54	40
1015	line*coccidia	1	2	52951.83	16337.54	40
1015	line*coccidia	1	3	73319.67	16337.54	40
1015	line*coccidia	2	0	97970.83	16337.54	40
1015	line*coccidia	2	1	82683.33	16337.54	40
1015	line*coccidia	2	2	132735.5	16337.54	40
1015	line*coccidia	2	3	133677.2	16337.54	40
1016	line	1		-5.5E-12	2157.691	40
1016	line	2		18927.54	2157.691	40
1016	coccidia		0	12821.25	3051.436	40
1016	coccidia		1	-7.3E-12	3051.436	40
1016	coccidia		2	13026	3051.436	40
1016	coccidia		3	12007.83	3051.436	40
1016	line*coccidia	1	0	-3.9E-12	4315.383	40
1016	line*coccidia	1	1	-7.3E-12	4315.383	40
1016	line*coccidia	1	2	-3.6E-12	4315.383	40
1016	line*coccidia	1	3	-7.3E-12	4315.383	40
1016	line*coccidia	2	0	25642.5	4315.383	40
1016	line*coccidia	2	1	-7.3E-12	4315.383	40
1016	line*coccidia	2	2	26052	4315.383	40
1016	line*coccidia	2	3	24015.67	4315.383	40
1018	line	1		7047.292	4184.537	40
1018	line	2		24875.42	4184.537	40
1018	coccidia		0	26292.5	5917.829	40
1018	coccidia		1	7.28E-12	5917.829	40
1018	coccidia		2	12561.67	5917.829	40
1018	coccidia		3	24991.25	5917.829	40
1018	line*coccidia	1	0	28189.17	8369.073	40
1018	line*coccidia	1	1	7.28E-12	8369.073	40
1018	line*coccidia	1	2	7.28E-12	8369.073	40
1018	line*coccidia	1	3	7.28E-12	8369.073	40
1018	line*coccidia	2	0	24395.83	8369.073	40
1018	line*coccidia	2	1	7.28E-12	8369.073	40
1018	line*coccidia	2	2	25123.33	8369.073	40
1018	line*coccidia	2	3	49982.5	8369.073	40
1021	line	1		42862.38	595860.8	40
1021	line	2		1328687	595860.8	40

1021	coccidia		0	1104507	842674.4	40
1021	coccidia		1	1521410	842674.4	40
1021	coccidia		2	15059.92	842674.4	40
1021	coccidia		3	102122.2	842674.4	40
1021	line*coccidia	1	0	62428.5	1191722	40
1021	line*coccidia	1	1	8953.333	1191722	40
1021	line*coccidia	1	2	12652.67	1191722	40
1021	line*coccidia	1	3	87415	1191722	40
1021	line*coccidia	2	0	2146585	1191722	40
1021	line*coccidia	2	1	3033866	1191722	40
1021	line*coccidia	2	2	17467.17	1191722	40
1021	line*coccidia	2	3	116829.3	1191722	40
1022	line	1		19163.67	504374.7	40
1022	line	2		887524.9	504374.7	40
1022	coccidia		0	390078.7	713293.5	40
1022	coccidia		1	1423298	713293.5	40
1022	coccidia		2	0	713293.5	40
1022	coccidia		3	0	713293.5	40
1022	line*coccidia	1	0	76654.67	1008749	40
1022	line*coccidia	1	1	-8.5E-11	1008749	40
1022	line*coccidia	1	2	7.07E-11	1008749	40
1022	line*coccidia	1	3	-8.5E-11	1008749	40
1022	line*coccidia	2	0	703502.7	1008749	40
1022	line*coccidia	2	1	2846597	1008749	40
1022	line*coccidia	2	2	-7.1E-11	1008749	40
1022	line*coccidia	2	3	8.45E-11	1008749	40
1101	line	1		312491.8	44624.52	40
1101	line	2		78686.63	44624.52	40
1101	coccidia		0	201904.3	63108.61	40
1101	coccidia		1	207966	63108.61	40
1101	coccidia		2	100004.9	63108.61	40
1101	coccidia		3	272481.5	63108.61	40
1101	line*coccidia	1	0	325834.8	89249.05	40
1101	line*coccidia	1	1	346162.5	89249.05	40
1101	line*coccidia	1	2	96423.17	89249.05	40
1101	line*coccidia	1	3	481546.5	89249.05	40
1101	line*coccidia	2	0	77973.83	89249.05	40
1101	line*coccidia	2	1	69769.5	89249.05	40
1101	line*coccidia	2	2	103586.7	89249.05	40
1101	line*coccidia	2	3	63416.5	89249.05	40
1102	line	1		265484	32134.37	40
1102	line	2		131673.2	32134.37	40
1102	coccidia		0	195818.1	45444.86	40
1102	coccidia		1	190617.7	45444.86	40
1102	coccidia		2	175176.3	45444.86	40
1102	coccidia		3	232702.3	45444.86	40
1102	line*coccidia	1	0	178436.2	64268.73	40
1102	line*coccidia	1	1	272067.7	64268.73	40
1102	line*coccidia	1	2	218243.8	64268.73	40

1102	line*coccidia	1	3	393188.3	64268.73	40
1102	line*coccidia	2	0	213200	64268.73	40
1102	line*coccidia	2	1	109167.7	64268.73	40
1102	line*coccidia	2	2	132108.8	64268.73	40
1102	line*coccidia	2	3	72216.17	64268.73	40
1103	line	1		387183	34140.38	40
1103	line	2		260813.1	34140.38	40
1103	coccidia		0	264785.6	48281.79	40
1103	coccidia		1	352353.4	48281.79	40
1103	coccidia		2	291208.8	48281.79	40
1103	coccidia		3	387644.5	48281.79	40
1103	line*coccidia	1	0	274829.5	68280.76	40
1103	line*coccidia	1	1	553111	68280.76	40
1103	line*coccidia	1	2	217545.3	68280.76	40
1103	line*coccidia	1	3	503246.2	68280.76	40
1103	line*coccidia	2	0	254741.7	68280.76	40
1103	line*coccidia	2	1	151595.8	68280.76	40
1103	line*coccidia	2	2	364872.2	68280.76	40
1103	line*coccidia	2	3	272042.8	68280.76	40
1106	line	1		1637493	240724.5	40
1106	line	2		1160183	240724.5	40
1106	coccidia		0	1935620	340435.8	40
1106	coccidia		1	2035544	340435.8	40
1106	coccidia		2	463121.8	340435.8	40
1106	coccidia		3	1161067	340435.8	40
1106	line*coccidia	1	0	3301020	481449	40
1106	line*coccidia	1	1	396448.7	481449	40
1106	line*coccidia	1	2	696256.7	481449	40
1106	line*coccidia	1	3	2156248	481449	40
1106	line*coccidia	2	0	570219.3	481449	40
1106	line*coccidia	2	1	3674639	481449	40
1106	line*coccidia	2	2	229986.8	481449	40
1106	line*coccidia	2	3	165885.2	481449	40
1114	line	1		311610	30469.36	40
1114	line	2		288415.2	30469.36	40
1114	coccidia		0	340436.9	43090.18	40
1114	coccidia		1	349237.7	43090.18	40
1114	coccidia		2	170234.8	43090.18	40
1114	coccidia		3	340141.1	43090.18	40
1114	line*coccidia	1	0	320808.5	60938.72	40
1114	line*coccidia	1	1	290472.8	60938.72	40
1114	line*coccidia	1	2	112849.2	60938.72	40
1114	line*coccidia	1	3	522309.7	60938.72	40
1114	line*coccidia	2	0	360065.3	60938.72	40
1114	line*coccidia	2	1	408002.5	60938.72	40
1114	line*coccidia	2	2	227620.5	60938.72	40
1114	line*coccidia	2	3	157972.5	60938.72	40
1116	line	1		96975.42	14629.66	40
1116	line	2		37010.79	14629.66	40

1116	coccidia		0	63997.83	20689.47	40
1116	coccidia		1	22679.08	20689.47	40
1116	coccidia		2	43366.75	20689.47	40
1116	coccidia		3	137928.8	20689.47	40
1116	line*coccidia	1	0	100153.7	29259.32	40
1116	line*coccidia	1	1	18870.83	29259.32	40
1116	line*coccidia	1	2	45151.67	29259.32	40
1116	line*coccidia	1	3	223725.5	29259.32	40
1116	line*coccidia	2	0	27842	29259.32	40
1116	line*coccidia	2	1	26487.33	29259.32	40
1116	line*coccidia	2	2	41581.83	29259.32	40
1116	line*coccidia	2	3	52132	29259.32	40
1117	line	1		248340	33399.25	40
1117	line	2		264213.3	33399.25	40
1117	coccidia		0	294469.5	47233.67	40
1117	coccidia		1	329052	47233.67	40
1117	coccidia		2	114308.7	47233.67	40
1117	coccidia		3	287276.4	47233.67	40
1117	line*coccidia	1	0	303946.7	66798.5	40
1117	line*coccidia	1	1	139804.2	66798.5	40
1117	line*coccidia	1	2	53470.5	66798.5	40
1117	line*coccidia	1	3	496138.5	66798.5	40
1117	line*coccidia	2	0	284992.3	66798.5	40
1117	line*coccidia	2	1	518299.8	66798.5	40
1117	line*coccidia	2	2	175146.8	66798.5	40
1117	line*coccidia	2	3	78414.33	66798.5	40
1201	line	1		224713	29615.69	40
1201	line	2		206363	29615.69	40
1201	coccidia		0	214039.8	41882.91	40
1201	coccidia		1	126249.6	41882.91	40
1201	coccidia		2	204157.3	41882.91	40
1201	coccidia		3	317705.3	41882.91	40
1201	line*coccidia	1	0	236229.2	59231.38	40
1201	line*coccidia	1	1	120223	59231.38	40
1201	line*coccidia	1	2	147794.2	59231.38	40
1201	line*coccidia	1	3	394605.5	59231.38	40
1201	line*coccidia	2	0	191850.3	59231.38	40
1201	line*coccidia	2	1	132276.2	59231.38	40
1201	line*coccidia	2	2	260520.3	59231.38	40
1201	line*coccidia	2	3	240805	59231.38	40
1202	line	1		398098.2	43139.79	40
1202	line	2		275450.1	43139.79	40
1202	coccidia		0	339081.3	61008.88	40
1202	coccidia		1	256488	61008.88	40
1202	coccidia		2	254247.4	61008.88	40
1202	coccidia		3	497279.9	61008.88	40
1202	line*coccidia	1	0	391719.7	86279.59	40
1202	line*coccidia	1	1	389073.7	86279.59	40
1202	line*coccidia	1	2	181716.5	86279.59	40

1202	line*coccidia	1	3	629883	86279.59	40
1202	line*coccidia	2	0	286442.8	86279.59	40
1202	line*coccidia	2	1	123902.3	86279.59	40
1202	line*coccidia	2	2	326778.3	86279.59	40
1202	line*coccidia	2	3	364676.8	86279.59	40
1205	line	1		1517426	215215.4	40
1205	line	2		884810	215215.4	40
1205	coccidia		0	1057801	304360.5	40
1205	coccidia		1	1367869	304360.5	40
1205	coccidia		2	773373	304360.5	40
1205	coccidia		3	1605430	304360.5	40
1205	line*coccidia	1	0	962472.8	430430.8	40
1205	line*coccidia	1	1	2153666	430430.8	40
1205	line*coccidia	1	2	700936.5	430430.8	40
1205	line*coccidia	1	3	2252630	430430.8	40
1205	line*coccidia	2	0	1153129	430430.8	40
1205	line*coccidia	2	1	582072.7	430430.8	40
1205	line*coccidia	2	2	845809.5	430430.8	40
1205	line*coccidia	2	3	958229.5	430430.8	40
1210	line	1		20686838	3523521	40
1210	line	2		31333599	3523521	40
1210	coccidia		0	19717717	4983011	40
1210	coccidia		1	17439969	4983011	40
1210	coccidia		2	18904388	4983011	40
1210	coccidia		3	47978801	4983011	40
1210	line*coccidia	1	0	24176072	7047042	40
1210	line*coccidia	1	1	8295742	7047042	40
1210	line*coccidia	1	2	9238511	7047042	40
1210	line*coccidia	1	3	41037028	7047042	40
1210	line*coccidia	2	0	15259362	7047042	40
1210	line*coccidia	2	1	26584196	7047042	40
1210	line*coccidia	2	2	28570265	7047042	40
1210	line*coccidia	2	3	54920574	7047042	40
1216	line	1		1037874	158463.4	40
1216	line	2		995509	158463.4	40
1216	coccidia		0	1087704	224101.2	40
1216	coccidia		1	563637.4	224101.2	40
1216	coccidia		2	815420.4	224101.2	40
1216	coccidia		3	1600004	224101.2	40
1216	line*coccidia	1	0	965717.7	316926.9	40
1216	line*coccidia	1	1	629639	316926.9	40
1216	line*coccidia	1	2	643231.7	316926.9	40
1216	line*coccidia	1	3	1912907	316926.9	40
1216	line*coccidia	2	0	1209690	316926.9	40
1216	line*coccidia	2	1	497635.8	316926.9	40
1216	line*coccidia	2	2	987609.2	316926.9	40
1216	line*coccidia	2	3	1287101	316926.9	40
1220	line	1		52976.5	7927.947	40
1220	line	2		55696.96	7927.947	40

1220	coccidia		0	63617.75	11211.81	40
1220	coccidia		1	55475.58	11211.81	40
1220	coccidia		2	41507.58	11211.81	40
1220	coccidia		3	56746	11211.81	40
1220	line*coccidia	1	0	63589.67	15855.89	40
1220	line*coccidia	1	1	32497.17	15855.89	40
1220	line*coccidia	1	2	37303.83	15855.89	40
1220	line*coccidia	1	3	78515.33	15855.89	40
1220	line*coccidia	2	0	63645.83	15855.89	40
1220	line*coccidia	2	1	78454	15855.89	40
1220	line*coccidia	2	2	45711.33	15855.89	40
1220	line*coccidia	2	3	34976.67	15855.89	40
1222	line	1		7461427	1449792	40
1222	line	2		8312651	1449792	40
1222	coccidia		0	9528695	2050316	40
1222	coccidia		1	7183621	2050316	40
1222	coccidia		2	3634243	2050316	40
1222	coccidia		3	11201598	2050316	40
1222	line*coccidia	1	0	6547645	2899585	40
1222	line*coccidia	1	1	10410716	2899585	40
1222	line*coccidia	1	2	3134654	2899585	40
1222	line*coccidia	1	3	9752694	2899585	40
1222	line*coccidia	2	0	12509746	2899585	40
1222	line*coccidia	2	1	3956526	2899585	40
1222	line*coccidia	2	2	4133832	2899585	40
1222	line*coccidia	2	3	12650502	2899585	40
1223	line	1		544596.8	51332.47	40
1223	line	2		288460	51332.47	40
1223	coccidia		0	661146.4	72595.08	40
1223	coccidia		1	242325.7	72595.08	40
1223	coccidia		2	92136.83	72595.08	40
1223	coccidia		3	670504.7	72595.08	40
1223	line*coccidia	1	0	997445	102664.9	40
1223	line*coccidia	1	1	130442	102664.9	40
1223	line*coccidia	1	2	57287.33	102664.9	40
1223	line*coccidia	1	3	993212.7	102664.9	40
1223	line*coccidia	2	0	324847.8	102664.9	40
1223	line*coccidia	2	1	354209.3	102664.9	40
1223	line*coccidia	2	2	126986.3	102664.9	40
1223	line*coccidia	2	3	347796.7	102664.9	40
1225	line	1		2848936	569917	40
1225	line	2		2103772	569917	40
1225	coccidia		0	2280521	805984.3	40
1225	coccidia		1	2423825	805984.3	40
1225	coccidia		2	746730	805984.3	40
1225	coccidia		3	4454341	805984.3	40
1225	line*coccidia	1	0	2377944	1139834	40
1225	line*coccidia	1	1	3303388	1139834	40
1225	line*coccidia	1	2	201804.3	1139834	40

1225	line*coccidia	1	3	5512609	1139834	40
1225	line*coccidia	2	0	2183098	1139834	40
1225	line*coccidia	2	1	1544262	1139834	40
1225	line*coccidia	2	2	1291656	1139834	40
1225	line*coccidia	2	3	3396073	1139834	40
1226	line	1		274973.1	40960.13	40
1226	line	2		311575.8	40960.13	40
1226	coccidia		0	286007.2	57926.37	40
1226	coccidia		1	358248.6	57926.37	40
1226	coccidia		2	119318.3	57926.37	40
1226	coccidia		3	409523.7	57926.37	40
1226	line*coccidia	1	0	355387.2	81920.26	40
1226	line*coccidia	1	1	303829.8	81920.26	40
1226	line*coccidia	1	2	62681.83	81920.26	40
1226	line*coccidia	1	3	377993.5	81920.26	40
1226	line*coccidia	2	0	216627.2	81920.26	40
1226	line*coccidia	2	1	412667.3	81920.26	40
1226	line*coccidia	2	2	175954.8	81920.26	40
1226	line*coccidia	2	3	441053.8	81920.26	40
1227	line	1		1764340	300222.4	40
1227	line	2		1022789	300222.4	40
1227	coccidia		0	1533685	424578.5	40
1227	coccidia		1	1482401	424578.5	40
1227	coccidia		2	614755.9	424578.5	40
1227	coccidia		3	1943417	424578.5	40
1227	line*coccidia	1	0	2599040	600444.7	40
1227	line*coccidia	1	1	2137637	600444.7	40
1227	line*coccidia	1	2	757476.2	600444.7	40
1227	line*coccidia	1	3	1563208	600444.7	40
1227	line*coccidia	2	0	468329.7	600444.7	40
1227	line*coccidia	2	1	827164.2	600444.7	40
1227	line*coccidia	2	2	472035.7	600444.7	40
1227	line*coccidia	2	3	2323627	600444.7	40
1228	line	1		92687.42	14348.05	40
1228	line	2		183156.8	14348.05	40
1228	coccidia		0	202041.9	20291.21	40
1228	coccidia		1	139318.3	20291.21	40
1228	coccidia		2	83573.42	20291.21	40
1228	coccidia		3	126754.7	20291.21	40
1228	line*coccidia	1	0	110954.5	28696.11	40
1228	line*coccidia	1	1	66497	28696.11	40
1228	line*coccidia	1	2	41376	28696.11	40
1228	line*coccidia	1	3	151922.2	28696.11	40
1228	line*coccidia	2	0	293129.3	28696.11	40
1228	line*coccidia	2	1	212139.7	28696.11	40
1228	line*coccidia	2	2	125770.8	28696.11	40
1228	line*coccidia	2	3	101587.2	28696.11	40
1301	line	1		1076633	223906.4	40
1301	line	2		920740.8	223906.4	40

1301	coccidia		0	1357911	316651.5	40
1301	coccidia		1	765531.9	316651.5	40
1301	coccidia		2	498827.9	316651.5	40
1301	coccidia		3	1372478	316651.5	40
1301	line*coccidia	1	0	763925.3	447812.8	40
1301	line*coccidia	1	1	702749.2	447812.8	40
1301	line*coccidia	1	2	387975	447812.8	40
1301	line*coccidia	1	3	2451884	447812.8	40
1301	line*coccidia	2	0	1951896	447812.8	40
1301	line*coccidia	2	1	828314.7	447812.8	40
1301	line*coccidia	2	2	609680.8	447812.8	40
1301	line*coccidia	2	3	293071.2	447812.8	40
1302	line	1		1196215	211809	40
1302	line	2		1221963	211809	40
1302	coccidia		0	1547504	299543.2	40
1302	coccidia		1	650511.2	299543.2	40
1302	coccidia		2	1056005	299543.2	40
1302	coccidia		3	1582337	299543.2	40
1302	line*coccidia	1	0	992473.3	423618.1	40
1302	line*coccidia	1	1	852553.7	423618.1	40
1302	line*coccidia	1	2	398128.3	423618.1	40
1302	line*coccidia	1	3	2541706	423618.1	40
1302	line*coccidia	2	0	2102535	423618.1	40
1302	line*coccidia	2	1	448468.7	423618.1	40
1302	line*coccidia	2	2	1713881	423618.1	40
1302	line*coccidia	2	3	622967.5	423618.1	40
1303	line	1		1464179	183453	40
1303	line	2		1592456	183453	40
1303	coccidia		0	1961713	259441.8	40
1303	coccidia		1	1177628	259441.8	40
1303	coccidia		2	1105364	259441.8	40
1303	coccidia		3	1868567	259441.8	40
1303	line*coccidia	1	0	2384971	366906.1	40
1303	line*coccidia	1	1	1505011	366906.1	40
1303	line*coccidia	1	2	628345	366906.1	40
1303	line*coccidia	1	3	1338391	366906.1	40
1303	line*coccidia	2	0	1538454	366906.1	40
1303	line*coccidia	2	1	850245.3	366906.1	40
1303	line*coccidia	2	2	1582382	366906.1	40
1303	line*coccidia	2	3	2398744	366906.1	40
1304	line	1		1632565	178910.1	40
1304	line	2		583813.7	178910.1	40
1304	coccidia		0	1506472	253017	40
1304	coccidia		1	638814.6	253017	40
1304	coccidia		2	528510.7	253017	40
1304	coccidia		3	1758959	253017	40
1304	line*coccidia	1	0	1840503	357820.1	40
1304	line*coccidia	1	1	926253.7	357820.1	40
1304	line*coccidia	1	2	552431.8	357820.1	40

1304	line*coccidia	1	3	3211071	357820.1	40
1304	line*coccidia	2	0	1172442	357820.1	40
1304	line*coccidia	2	1	351375.5	357820.1	40
1304	line*coccidia	2	2	504589.5	357820.1	40
1304	line*coccidia	2	3	306848	357820.1	40
1312	line	1		331768.8	249925.6	40
1312	line	2		778574.5	249925.6	40
1312	coccidia		0	563711.9	353448.2	40
1312	coccidia		1	1017116	353448.2	40
1312	coccidia		2	230044.3	353448.2	40
1312	coccidia		3	409814.5	353448.2	40
1312	line*coccidia	1	0	453953.3	499851.2	40
1312	line*coccidia	1	1	114643.5	499851.2	40
1312	line*coccidia	1	2	57997.17	499851.2	40
1312	line*coccidia	1	3	700481	499851.2	40
1312	line*coccidia	2	0	673470.5	499851.2	40
1312	line*coccidia	2	1	1919588	499851.2	40
1312	line*coccidia	2	2	402091.3	499851.2	40
1312	line*coccidia	2	3	119148	499851.2	40
1313	line	1		2198608	652836.9	40
1313	line	2		4324382	652836.9	40
1313	coccidia		0	5160270	923250.9	40
1313	coccidia		1	3158875	923250.9	40
1313	coccidia		2	2014379	923250.9	40
1313	coccidia		3	2712458	923250.9	40
1313	line*coccidia	1	0	2388458	1305674	40
1313	line*coccidia	1	1	1733442	1305674	40
1313	line*coccidia	1	2	1012039	1305674	40
1313	line*coccidia	1	3	3660495	1305674	40
1313	line*coccidia	2	0	7932082	1305674	40
1313	line*coccidia	2	1	4584308	1305674	40
1313	line*coccidia	2	2	3016718	1305674	40
1313	line*coccidia	2	3	1764420	1305674	40
1314	line	1		21684953	3528235	40
1314	line	2		16548207	3528235	40
1314	coccidia		0	21353713	4989678	40
1314	coccidia		1	12248628	4989678	40
1314	coccidia		2	10734317	4989678	40
1314	coccidia		3	32129662	4989678	40
1314	line*coccidia	1	0	21030931	7056470	40
1314	line*coccidia	1	1	8086143	7056470	40
1314	line*coccidia	1	2	8135776	7056470	40
1314	line*coccidia	1	3	49486962	7056470	40
1314	line*coccidia	2	0	21676496	7056470	40
1314	line*coccidia	2	1	16411114	7056470	40
1314	line*coccidia	2	2	13332859	7056470	40
1314	line*coccidia	2	3	14772361	7056470	40
1315	line	1		156043.3	45499.61	40
1315	line	2		210944.2	45499.61	40

1315	coccidia		0	231374.8	64346.17	40
1315	coccidia		1	176346.2	64346.17	40
1315	coccidia		2	126500.1	64346.17	40
1315	coccidia		3	199753.8	64346.17	40
1315	line*coccidia	1	0	115339	90999.23	40
1315	line*coccidia	1	1	178155.7	90999.23	40
1315	line*coccidia	1	2	48887.33	90999.23	40
1315	line*coccidia	1	3	281791.2	90999.23	40
1315	line*coccidia	2	0	347410.7	90999.23	40
1315	line*coccidia	2	1	174536.7	90999.23	40
1315	line*coccidia	2	2	204112.8	90999.23	40
1315	line*coccidia	2	3	117716.5	90999.23	40
1316	line	1		35739505	3929741	40
1316	line	2		43111270	3929741	40
1316	coccidia		0	56419258	5557492	40
1316	coccidia		1	42310138	5557492	40
1316	coccidia		2	16981845	5557492	40
1316	coccidia		3	41990308	5557492	40
1316	line*coccidia	1	0	55207621	7859481	40
1316	line*coccidia	1	1	17726345	7859481	40
1316	line*coccidia	1	2	10397128	7859481	40
1316	line*coccidia	1	3	59626925	7859481	40
1316	line*coccidia	2	0	57630896	7859481	40
1316	line*coccidia	2	1	66893930	7859481	40
1316	line*coccidia	2	2	23566563	7859481	40
1316	line*coccidia	2	3	24353691	7859481	40
1317	line	1		7426522	1506409	40
1317	line	2		10025605	1506409	40
1317	coccidia		0	9552115	2130384	40
1317	coccidia		1	8162670	2130384	40
1317	coccidia		2	5887059	2130384	40
1317	coccidia		3	11302410	2130384	40
1317	line*coccidia	1	0	9934486	3012818	40
1317	line*coccidia	1	1	5865070	3012818	40
1317	line*coccidia	1	2	1117336	3012818	40
1317	line*coccidia	1	3	12789198	3012818	40
1317	line*coccidia	2	0	9169744	3012818	40
1317	line*coccidia	2	1	10460271	3012818	40
1317	line*coccidia	2	2	10656782	3012818	40
1317	line*coccidia	2	3	9815623	3012818	40
1321	line	1		318756.8	48444.27	40
1321	line	2		291517.2	48444.27	40
1321	coccidia		0	460474.8	68510.54	40
1321	coccidia		1	305940.3	68510.54	40
1321	coccidia		2	147985.1	68510.54	40
1321	coccidia		3	306147.8	68510.54	40
1321	line*coccidia	1	0	342045.5	96888.54	40
1321	line*coccidia	1	1	235989	96888.54	40
1321	line*coccidia	1	2	148259.8	96888.54	40

1321	line*coccidia	1	3	548733	96888.54	40
1321	line*coccidia	2	0	578904.2	96888.54	40
1321	line*coccidia	2	1	375891.7	96888.54	40
1321	line*coccidia	2	2	147710.3	96888.54	40
1321	line*coccidia	2	3	63562.5	96888.54	40
1322	line	1		243135	40191.81	40
1322	line	2		116596.4	40191.81	40
1322	coccidia		0	156392	56839.81	40
1322	coccidia		1	233963.8	56839.81	40
1322	coccidia		2	88657.5	56839.81	40
1322	coccidia		3	240449.6	56839.81	40
1322	line*coccidia	1	0	194556.2	80383.63	40
1322	line*coccidia	1	1	273503.3	80383.63	40
1322	line*coccidia	1	2	77758.33	80383.63	40
1322	line*coccidia	1	3	426722.2	80383.63	40
1322	line*coccidia	2	0	118227.8	80383.63	40
1322	line*coccidia	2	1	194424.2	80383.63	40
1322	line*coccidia	2	2	99556.67	80383.63	40
1322	line*coccidia	2	3	54177	80383.63	40
1323	line	1		9957011	1847048	40
1323	line	2		8694491	1847048	40
1323	coccidia		0	13302673	2612120	40
1323	coccidia		1	12611790	2612120	40
1323	coccidia		2	3883558	2612120	40
1323	coccidia		3	7504984	2612120	40
1323	line*coccidia	1	0	10047478	3694096	40
1323	line*coccidia	1	1	17688470	3694096	40
1323	line*coccidia	1	2	3743980	3694096	40
1323	line*coccidia	1	3	8348115	3694096	40
1323	line*coccidia	2	0	16557867	3694096	40
1323	line*coccidia	2	1	7535109	3694096	40
1323	line*coccidia	2	2	4023135	3694096	40
1323	line*coccidia	2	3	6661853	3694096	40
1324	line	1		13289448	2243218	40
1324	line	2		14993416	2243218	40
1324	coccidia		0	22252004	3172389	40
1324	coccidia		1	12450678	3172389	40
1324	coccidia		2	9784893	3172389	40
1324	coccidia		3	12078151	3172389	40
1324	line*coccidia	1	0	25867614	4486436	40
1324	line*coccidia	1	1	10441284	4486436	40
1324	line*coccidia	1	2	7232055	4486436	40
1324	line*coccidia	1	3	9616837	4486436	40
1324	line*coccidia	2	0	18636395	4486436	40
1324	line*coccidia	2	1	14460071	4486436	40
1324	line*coccidia	2	2	12337732	4486436	40
1324	line*coccidia	2	3	14539465	4486436	40
1401	line	1		6050566	783612.3	40
1401	line	2		6065587	783612.3	40

1401	coccidia		0	5598151	1108195	40
1401	coccidia		1	5502181	1108195	40
1401	coccidia		2	4290611	1108195	40
1401	coccidia		3	8841362	1108195	40
1401	line*coccidia	1	0	5642961	1567225	40
1401	line*coccidia	1	1	6957465	1567225	40
1401	line*coccidia	1	2	3282449	1567225	40
1401	line*coccidia	1	3	8319389	1567225	40
1401	line*coccidia	2	0	5553342	1567225	40
1401	line*coccidia	2	1	4046896	1567225	40
1401	line*coccidia	2	2	5298772	1567225	40
1401	line*coccidia	2	3	9363336	1567225	40
1402	line	1		8608278	976068.9	40
1402	line	2		7279846	976068.9	40
1402	coccidia		0	10880489	1380370	40
1402	coccidia		1	5775371	1380370	40
1402	coccidia		2	6068671	1380370	40
1402	coccidia		3	9051716	1380370	40
1402	line*coccidia	1	0	12534528	1952138	40
1402	line*coccidia	1	1	7386383	1952138	40
1402	line*coccidia	1	2	5895417	1952138	40
1402	line*coccidia	1	3	8616785	1952138	40
1402	line*coccidia	2	0	9226450	1952138	40
1402	line*coccidia	2	1	4164360	1952138	40
1402	line*coccidia	2	2	6241925	1952138	40
1402	line*coccidia	2	3	9486648	1952138	40
1403	line	1		70908951	5565516	40
1403	line	2		69549645	5565516	40
1403	coccidia		0	74002300	7870828	40
1403	coccidia		1	31693746	7870828	40
1403	coccidia		2	70943090	7870828	40
1403	coccidia		3	1.04E+08	7870828	40
1403	line*coccidia	1	0	64660383	11131032	40
1403	line*coccidia	1	1	45675942	11131032	40
1403	line*coccidia	1	2	47886919	11131032	40
1403	line*coccidia	1	3	1.25E+08	11131032	40
1403	line*coccidia	2	0	83344216	11131032	40
1403	line*coccidia	2	1	17711549	11131032	40
1403	line*coccidia	2	2	93999261	11131032	40
1403	line*coccidia	2	3	83143553	11131032	40
1404	line	1		5712988	577015	40
1404	line	2		4359067	577015	40
1404	coccidia		0	4083000	816022.4	40
1404	coccidia		1	4630107	816022.4	40
1404	coccidia		2	3011291	816022.4	40
1404	coccidia		3	8419712	816022.4	40
1404	line*coccidia	1	0	3489915	1154030	40
1404	line*coccidia	1	1	7166360	1154030	40
1404	line*coccidia	1	2	2286480	1154030	40

1404	line*coccidia	1	3	9909197	1154030	40
1404	line*coccidia	2	0	4676086	1154030	40
1404	line*coccidia	2	1	2093855	1154030	40
1404	line*coccidia	2	2	3736102	1154030	40
1404	line*coccidia	2	3	6930228	1154030	40
1405	line	1		1435513	154728.2	40
1405	line	2		741216	154728.2	40
1405	coccidia		0	927004.7	218818.7	40
1405	coccidia		1	1410722	218818.7	40
1405	coccidia		2	376263.1	218818.7	40
1405	coccidia		3	1639468	218818.7	40
1405	line*coccidia	1	0	1482891	309456.3	40
1405	line*coccidia	1	1	1882161	309456.3	40
1405	line*coccidia	1	2	288587	309456.3	40
1405	line*coccidia	1	3	2088412	309456.3	40
1405	line*coccidia	2	0	371118.5	309456.3	40
1405	line*coccidia	2	1	939283.3	309456.3	40
1405	line*coccidia	2	2	463939.2	309456.3	40
1405	line*coccidia	2	3	1190523	309456.3	40
1415	line	1		326876.1	73959.62	40
1415	line	2		381850.1	73959.62	40
1415	coccidia		0	635778.9	104594.7	40
1415	coccidia		1	319751.8	104594.7	40
1415	coccidia		2	210079.1	104594.7	40
1415	coccidia		3	251842.7	104594.7	40
1415	line*coccidia	1	0	838374.7	147919.2	40
1415	line*coccidia	1	1	90342.83	147919.2	40
1415	line*coccidia	1	2	37596	147919.2	40
1415	line*coccidia	1	3	341191	147919.2	40
1415	line*coccidia	2	0	433183.2	147919.2	40
1415	line*coccidia	2	1	549160.7	147919.2	40
1415	line*coccidia	2	2	382562.2	147919.2	40
1415	line*coccidia	2	3	162494.3	147919.2	40
1417	line	1		1756478	1534010	40
1417	line	2		4867328	1534010	40
1417	coccidia		0	6412226	2169417	40
1417	coccidia		1	4404374	2169417	40
1417	coccidia		2	1297571	2169417	40
1417	coccidia		3	1133440	2169417	40
1417	line*coccidia	1	0	1571291	3068019	40
1417	line*coccidia	1	1	3821928	3068019	40
1417	line*coccidia	1	2	441778.3	3068019	40
1417	line*coccidia	1	3	1190915	3068019	40
1417	line*coccidia	2	0	11253161	3068019	40
1417	line*coccidia	2	1	4986820	3068019	40
1417	line*coccidia	2	2	2153364	3068019	40
1417	line*coccidia	2	3	1075965	3068019	40
1419	line	1		0	80865.32	40
1419	line	2		584968.7	80865.32	40

1419	coccidia		0	113747.8	114360.8	40
1419	coccidia		1	0	114360.8	40
1419	coccidia		2	547880.1	114360.8	40
1419	coccidia		3	508309.4	114360.8	40
1419	line*coccidia	1	0	0	161730.6	40
1419	line*coccidia	1	1	0	161730.6	40
1419	line*coccidia	1	2	0	161730.6	40
1419	line*coccidia	1	3	0	161730.6	40
1419	line*coccidia	2	0	227495.7	161730.6	40
1419	line*coccidia	2	1	0	161730.6	40
1419	line*coccidia	2	2	1095760	161730.6	40
1419	line*coccidia	2	3	1016619	161730.6	40
1420	line	1		1662562	227876	40
1420	line	2		1114006	227876	40
1420	coccidia		0	1526161	322265.4	40
1420	coccidia		1	1733475	322265.4	40
1420	coccidia		2	696691.3	322265.4	40
1420	coccidia		3	1596809	322265.4	40
1420	line*coccidia	1	0	1504536	455752.1	40
1420	line*coccidia	1	1	2011442	455752.1	40
1420	line*coccidia	1	2	783728.7	455752.1	40
1420	line*coccidia	1	3	2350541	455752.1	40
1420	line*coccidia	2	0	1547785	455752.1	40
1420	line*coccidia	2	1	1455509	455752.1	40
1420	line*coccidia	2	2	609654	455752.1	40
1420	line*coccidia	2	3	843075.8	455752.1	40
1421	line	1		80758223	9481696	40
1421	line	2		76748792	9481696	40
1421	coccidia		0	78645623	13409143	40
1421	coccidia		1	75971803	13409143	40
1421	coccidia		2	79567695	13409143	40
1421	coccidia		3	80828909	13409143	40
1421	line*coccidia	1	0	67143522	18963392	40
1421	line*coccidia	1	1	82053534	18963392	40
1421	line*coccidia	1	2	86268732	18963392	40
1421	line*coccidia	1	3	87567104	18963392	40
1421	line*coccidia	2	0	90147723	18963392	40
1421	line*coccidia	2	1	69890072	18963392	40
1421	line*coccidia	2	2	72866658	18963392	40
1421	line*coccidia	2	3	74090713	18963392	40
1422	line	1		3342666	434136.8	40
1422	line	2		1950582	434136.8	40
1422	coccidia		0	3423179	613962.2	40
1422	coccidia		1	2578010	613962.2	40
1422	coccidia		2	1451135	613962.2	40
1422	coccidia		3	3134173	613962.2	40
1422	line*coccidia	1	0	4629889	868273.7	40
1422	line*coccidia	1	1	3223383	868273.7	40
1422	line*coccidia	1	2	1788274	868273.7	40

1422	line*coccidia	1	3	3729119	868273.7	40
1422	line*coccidia	2	0	2216469	868273.7	40
1422	line*coccidia	2	1	1932637	868273.7	40
1422	line*coccidia	2	2	1113996	868273.7	40
1422	line*coccidia	2	3	2539227	868273.7	40
1423	line	1		194033	40781.16	40
1423	line	2		118963.3	40781.16	40
1423	coccidia		0	245378.8	57673.27	40
1423	coccidia		1	-2.9E-11	57673.27	40
1423	coccidia		2	176577.7	57673.27	40
1423	coccidia		3	204036.1	57673.27	40
1423	line*coccidia	1	0	368059.7	81562.32	40
1423	line*coccidia	1	1	-5.8E-11	81562.32	40
1423	line*coccidia	1	2	-5.8E-11	81562.32	40
1423	line*coccidia	1	3	408072.2	81562.32	40
1423	line*coccidia	2	0	122698	81562.32	40
1423	line*coccidia	2	1	0	81562.32	40
1423	line*coccidia	2	2	353155.3	81562.32	40
1423	line*coccidia	2	3	0	81562.32	40
1502	line	1		9867018	764818.1	40
1502	line	2		7338647	764818.1	40
1502	coccidia		0	9924322	1081616	40
1502	coccidia		1	5778143	1081616	40
1502	coccidia		2	7662680	1081616	40
1502	coccidia		3	11046185	1081616	40
1502	line*coccidia	1	0	10644946	1529636	40
1502	line*coccidia	1	1	8529779	1529636	40
1502	line*coccidia	1	2	5131228	1529636	40
1502	line*coccidia	1	3	15162120	1529636	40
1502	line*coccidia	2	0	9203698	1529636	40
1502	line*coccidia	2	1	3026507	1529636	40
1502	line*coccidia	2	2	10194132	1529636	40
1502	line*coccidia	2	3	6930250	1529636	40
1503	line	1		864527.4	78837.81	40
1503	line	2		1285039	78837.81	40
1503	coccidia		0	1482299	111493.5	40
1503	coccidia		1	800006.6	111493.5	40
1503	coccidia		2	718621.8	111493.5	40
1503	coccidia		3	1298205	111493.5	40
1503	line*coccidia	1	0	1214022	157675.6	40
1503	line*coccidia	1	1	898521.7	157675.6	40
1503	line*coccidia	1	2	323481.8	157675.6	40
1503	line*coccidia	1	3	1022084	157675.6	40
1503	line*coccidia	2	0	1750576	157675.6	40
1503	line*coccidia	2	1	701491.5	157675.6	40
1503	line*coccidia	2	2	1113762	157675.6	40
1503	line*coccidia	2	3	1574327	157675.6	40
1508	line	1		3444693	517253.2	40
1508	line	2		4721954	517253.2	40

1508	coccidia		0	7584354	731506.5	40
1508	coccidia		1	1257833	731506.5	40
1508	coccidia		2	2953407	731506.5	40
1508	coccidia		3	4537700	731506.5	40
1508	line*coccidia	1	0	7145066	1034506	40
1508	line*coccidia	1	1	509387.8	1034506	40
1508	line*coccidia	1	2	1572707	1034506	40
1508	line*coccidia	1	3	4551612	1034506	40
1508	line*coccidia	2	0	8023642	1034506	40
1508	line*coccidia	2	1	2006278	1034506	40
1508	line*coccidia	2	2	4334107	1034506	40
1508	line*coccidia	2	3	4523789	1034506	40
1515	line	1		13212170	1986879	40
1515	line	2		13826846	1986879	40
1515	coccidia		0	22322089	2809871	40
1515	coccidia		1	4551557	2809871	40
1515	coccidia		2	13949717	2809871	40
1515	coccidia		3	13254670	2809871	40
1515	line*coccidia	1	0	26153886	3973757	40
1515	line*coccidia	1	1	3633213	3973757	40
1515	line*coccidia	1	2	6235201	3973757	40
1515	line*coccidia	1	3	16826381	3973757	40
1515	line*coccidia	2	0	18490293	3973757	40
1515	line*coccidia	2	1	5469901	3973757	40
1515	line*coccidia	2	2	21664233	3973757	40
1515	line*coccidia	2	3	9682959	3973757	40
1517	line	1		4416558	339087	40
1517	line	2		3366301	339087	40
1517	coccidia		0	5920840	479541.5	40
1517	coccidia		1	3328197	479541.5	40
1517	coccidia		2	2581806	479541.5	40
1517	coccidia		3	3734873	479541.5	40
1517	line*coccidia	1	0	8043068	678174.1	40
1517	line*coccidia	1	1	2499651	678174.1	40
1517	line*coccidia	1	2	1215114	678174.1	40
1517	line*coccidia	1	3	5908398	678174.1	40
1517	line*coccidia	2	0	3798611	678174.1	40
1517	line*coccidia	2	1	4156744	678174.1	40
1517	line*coccidia	2	2	3948499	678174.1	40
1517	line*coccidia	2	3	1561349	678174.1	40
1519	line	1		11087592	1652225	40
1519	line	2		23016699	1652225	40
1519	coccidia		0	23318090	2336599	40
1519	coccidia		1	12560164	2336599	40
1519	coccidia		2	19267057	2336599	40
1519	coccidia		3	13063272	2336599	40
1519	line*coccidia	1	0	16459827	3304449	40
1519	line*coccidia	1	1	7033600	3304449	40
1519	line*coccidia	1	2	8250538	3304449	40

1519	line*coccidia	1	3	12606406	3304449	40
1519	line*coccidia	2	0	30176353	3304449	40
1519	line*coccidia	2	1	18086729	3304449	40
1519	line*coccidia	2	2	30283576	3304449	40
1519	line*coccidia	2	3	13520139	3304449	40
1521	line	1		4490227	763163.5	40
1521	line	2		5501804	763163.5	40
1521	coccidia		0	5731474	1079276	40
1521	coccidia		1	4415570	1079276	40
1521	coccidia		2	5381253	1079276	40
1521	coccidia		3	4455764	1079276	40
1521	line*coccidia	1	0	6463083	1526327	40
1521	line*coccidia	1	1	5820064	1526327	40
1521	line*coccidia	1	2	1826084	1526327	40
1521	line*coccidia	1	3	3851677	1526327	40
1521	line*coccidia	2	0	4999866	1526327	40
1521	line*coccidia	2	1	3011076	1526327	40
1521	line*coccidia	2	2	8936421	1526327	40
1521	line*coccidia	2	3	5059851	1526327	40
1523	line	1		2482816	317710.1	40
1523	line	2		2593649	317710.1	40
1523	coccidia		0	2878616	449310	40
1523	coccidia		1	3212878	449310	40
1523	coccidia		2	1621073	449310	40
1523	coccidia		3	2440364	449310	40
1523	line*coccidia	1	0	2853613	635420.3	40
1523	line*coccidia	1	1	3679306	635420.3	40
1523	line*coccidia	1	2	771165.5	635420.3	40
1523	line*coccidia	1	3	2627180	635420.3	40
1523	line*coccidia	2	0	2903619	635420.3	40
1523	line*coccidia	2	1	2746450	635420.3	40
1523	line*coccidia	2	2	2470981	635420.3	40
1523	line*coccidia	2	3	2253549	635420.3	40
1524	line	1		15488569	2887706	40
1524	line	2		22097140	2887706	40
1524	coccidia		0	25975838	4083832	40
1524	coccidia		1	16177272	4083832	40
1524	coccidia		2	15235467	4083832	40
1524	coccidia		3	17782840	4083832	40
1524	line*coccidia	1	0	20062594	5775411	40
1524	line*coccidia	1	1	6413246	5775411	40
1524	line*coccidia	1	2	10244931	5775411	40
1524	line*coccidia	1	3	25233503	5775411	40
1524	line*coccidia	2	0	31889082	5775411	40
1524	line*coccidia	2	1	25941298	5775411	40
1524	line*coccidia	2	2	20226002	5775411	40
1524	line*coccidia	2	3	10332177	5775411	40
1527	line	1		3680226	401779.5	40
1527	line	2		1796412	401779.5	40

1527	coccidia		0	2010905	568202	40
1527	coccidia		1	2265699	568202	40
1527	coccidia		2	1955453	568202	40
1527	coccidia		3	4721218	568202	40
1527	line*coccidia	1	0	2788086	803558.9	40
1527	line*coccidia	1	1	2145665	803558.9	40
1527	line*coccidia	1	2	1529816	803558.9	40
1527	line*coccidia	1	3	8257337	803558.9	40
1527	line*coccidia	2	0	1233725	803558.9	40
1527	line*coccidia	2	1	2385733	803558.9	40
1527	line*coccidia	2	2	2381091	803558.9	40
1527	line*coccidia	2	3	1185099	803558.9	40
1528	line	1		1205717	230542.5	40
1528	line	2		1590470	230542.5	40
1528	coccidia		0	2533333	326036.4	40
1528	coccidia		1	577012.9	326036.4	40
1528	coccidia		2	1134704	326036.4	40
1528	coccidia		3	1347324	326036.4	40
1528	line*coccidia	1	0	2336925	461085.1	40
1528	line*coccidia	1	1	2.33E-10	461085.1	40
1528	line*coccidia	1	2	376767.8	461085.1	40
1528	line*coccidia	1	3	2109173	461085.1	40
1528	line*coccidia	2	0	2729742	461085.1	40
1528	line*coccidia	2	1	1154026	461085.1	40
1528	line*coccidia	2	2	1892639	461085.1	40
1528	line*coccidia	2	3	585474.2	461085.1	40
1529	line	1		2488608	313265.9	40
1529	line	2		1697826	313265.9	40
1529	coccidia		0	2771053	443024.9	40
1529	coccidia		1	1733828	443024.9	40
1529	coccidia		2	598570.2	443024.9	40
1529	coccidia		3	3269416	443024.9	40
1529	line*coccidia	1	0	3376938	626531.9	40
1529	line*coccidia	1	1	9.31E-10	626531.9	40
1529	line*coccidia	1	2	1197140	626531.9	40
1529	line*coccidia	1	3	5380353	626531.9	40
1529	line*coccidia	2	0	2165168	626531.9	40
1529	line*coccidia	2	1	3467656	626531.9	40
1529	line*coccidia	2	2	2.33E-10	626531.9	40
1529	line*coccidia	2	3	1158478	626531.9	40
1601	line	1		1542451	293116.1	40
1601	line	2		1628471	293116.1	40
1601	coccidia		0	1791730	414528.7	40
1601	coccidia		1	877282.6	414528.7	40
1601	coccidia		2	1336309	414528.7	40
1601	coccidia		3	2336522	414528.7	40
1601	line*coccidia	1	0	1281602	586232.1	40
1601	line*coccidia	1	1	1382736	586232.1	40
1601	line*coccidia	1	2	782628.3	586232.1	40

1601	line*coccidia	1	3	2722838	586232.1	40
1601	line*coccidia	2	0	2301858	586232.1	40
1601	line*coccidia	2	1	371829.2	586232.1	40
1601	line*coccidia	2	2	1889990	586232.1	40
1601	line*coccidia	2	3	1950206	586232.1	40
1602	line	1		907348.3	92794.32	40
1602	line	2		729093.1	92794.32	40
1602	coccidia		0	693632.8	131231	40
1602	coccidia		1	542923.9	131231	40
1602	coccidia		2	821126.4	131231	40
1602	coccidia		3	1215200	131231	40
1602	line*coccidia	1	0	706538.3	185588.6	40
1602	line*coccidia	1	1	803080.8	185588.6	40
1602	line*coccidia	1	2	868476.2	185588.6	40
1602	line*coccidia	1	3	1251298	185588.6	40
1602	line*coccidia	2	0	680727.2	185588.6	40
1602	line*coccidia	2	1	282767	185588.6	40
1602	line*coccidia	2	2	773776.7	185588.6	40
1602	line*coccidia	2	3	1179102	185588.6	40
1603	line	1		3560940	375372.5	40
1603	line	2		2703738	375372.5	40
1603	coccidia		0	3985045	530856.8	40
1603	coccidia		1	1662398	530856.8	40
1603	coccidia		2	2571386	530856.8	40
1603	coccidia		3	4310528	530856.8	40
1603	line*coccidia	1	0	4375330	750744.9	40
1603	line*coccidia	1	1	2427283	750744.9	40
1603	line*coccidia	1	2	1715308	750744.9	40
1603	line*coccidia	1	3	5725839	750744.9	40
1603	line*coccidia	2	0	3594760	750744.9	40
1603	line*coccidia	2	1	897512.7	750744.9	40
1603	line*coccidia	2	2	3427463	750744.9	40
1603	line*coccidia	2	3	2895217	750744.9	40
1604	line	1		1483623	151892.4	40
1604	line	2		1042215	151892.4	40
1604	coccidia		0	1412132	214808.3	40
1604	coccidia		1	915898.3	214808.3	40
1604	coccidia		2	1189570	214808.3	40
1604	coccidia		3	1534076	214808.3	40
1604	line*coccidia	1	0	1396630	303784.9	40
1604	line*coccidia	1	1	1240404	303784.9	40
1604	line*coccidia	1	2	903059.8	303784.9	40
1604	line*coccidia	1	3	2394398	303784.9	40
1604	line*coccidia	2	0	1427633	303784.9	40
1604	line*coccidia	2	1	591392.5	303784.9	40
1604	line*coccidia	2	2	1476081	303784.9	40
1604	line*coccidia	2	3	673754	303784.9	40
1605	line	1		334352.1	64938.57	40
1605	line	2		567880.7	64938.57	40

1605	coccidia		0	763268.4	91837.01	40
1605	coccidia		1	333321.8	91837.01	40
1605	coccidia		2	375381.4	91837.01	40
1605	coccidia		3	332493.9	91837.01	40
1605	line*coccidia	1	0	736916.3	129877.1	40
1605	line*coccidia	1	1	203294.5	129877.1	40
1605	line*coccidia	1	2	148716.8	129877.1	40
1605	line*coccidia	1	3	248480.8	129877.1	40
1605	line*coccidia	2	0	789620.5	129877.1	40
1605	line*coccidia	2	1	463349.2	129877.1	40
1605	line*coccidia	2	2	602046	129877.1	40
1605	line*coccidia	2	3	416507	129877.1	40
1619	line	1		575161.3	92502.47	40
1619	line	2		720674.1	92502.47	40
1619	coccidia		0	1247918	130818.2	40
1619	coccidia		1	507260.8	130818.2	40
1619	coccidia		2	229980.8	130818.2	40
1619	coccidia		3	606511.3	130818.2	40
1619	line*coccidia	1	0	1207066	185004.9	40
1619	line*coccidia	1	1	145818.2	185004.9	40
1619	line*coccidia	1	2	165090.5	185004.9	40
1619	line*coccidia	1	3	782671	185004.9	40
1619	line*coccidia	2	0	1288771	185004.9	40
1619	line*coccidia	2	1	868703.3	185004.9	40
1619	line*coccidia	2	2	294871	185004.9	40
1619	line*coccidia	2	3	430351.7	185004.9	40
1620	line	1		571669.8	103786.6	40
1620	line	2		708520.2	103786.6	40
1620	coccidia		0	1139620	146776.4	40
1620	coccidia		1	459211.3	146776.4	40
1620	coccidia		2	289595.3	146776.4	40
1620	coccidia		3	671953.8	146776.4	40
1620	line*coccidia	1	0	965865.7	207573.1	40
1620	line*coccidia	1	1	184755.7	207573.1	40
1620	line*coccidia	1	2	144825.7	207573.1	40
1620	line*coccidia	1	3	991232	207573.1	40
1620	line*coccidia	2	0	1313374	207573.1	40
1620	line*coccidia	2	1	733666.8	207573.1	40
1620	line*coccidia	2	2	434364.8	207573.1	40
1620	line*coccidia	2	3	352675.7	207573.1	40
1621	line	1		2566171	326069.5	40
1621	line	2		2074987	326069.5	40
1621	coccidia		0	2803848	461131.9	40
1621	coccidia		1	1876889	461131.9	40
1621	coccidia		2	1810482	461131.9	40
1621	coccidia		3	2791097	461131.9	40
1621	line*coccidia	1	0	3615999	652139	40
1621	line*coccidia	1	1	1220046	652139	40
1621	line*coccidia	1	2	1006804	652139	40

1621	line*coccidia	1	3	4421835	652139	40
1621	line*coccidia	2	0	1991698	652139	40
1621	line*coccidia	2	1	2533731	652139	40
1621	line*coccidia	2	2	2614160	652139	40
1621	line*coccidia	2	3	1160358	652139	40
1622	line	1		1451394	217405.2	40
1622	line	2		2332077	217405.2	40
1622	coccidia		0	2615542	307457.4	40
1622	coccidia		1	2042291	307457.4	40
1622	coccidia		2	1292121	307457.4	40
1622	coccidia		3	1616988	307457.4	40
1622	line*coccidia	1	0	2297390	434810.4	40
1622	line*coccidia	1	1	1687850	434810.4	40
1622	line*coccidia	1	2	345126.3	434810.4	40
1622	line*coccidia	1	3	1475211	434810.4	40
1622	line*coccidia	2	0	2933695	434810.4	40
1622	line*coccidia	2	1	2396732	434810.4	40
1622	line*coccidia	2	2	2239116	434810.4	40
1622	line*coccidia	2	3	1758765	434810.4	40
1626	line	1		250620.4	60441.82	40
1626	line	2		234267.6	60441.82	40
1626	coccidia		0	443411.7	85477.65	40
1626	coccidia		1	53241	85477.65	40
1626	coccidia		2	110221.2	85477.65	40
1626	coccidia		3	362902.3	85477.65	40
1626	line*coccidia	1	0	467131.2	120883.6	40
1626	line*coccidia	1	1	23629	120883.6	40
1626	line*coccidia	1	2	86075.83	120883.6	40
1626	line*coccidia	1	3	425645.7	120883.6	40
1626	line*coccidia	2	0	419692.2	120883.6	40
1626	line*coccidia	2	1	82853	120883.6	40
1626	line*coccidia	2	2	134366.5	120883.6	40
1626	line*coccidia	2	3	300158.8	120883.6	40
1627	line	1		489121.4	46991.33	40
1627	line	2		391423.6	46991.33	40
1627	coccidia		0	544270.8	66455.77	40
1627	coccidia		1	136636.2	66455.77	40
1627	coccidia		2	346781.6	66455.77	40
1627	coccidia		3	733401.5	66455.77	40
1627	line*coccidia	1	0	722898.3	93982.65	40
1627	line*coccidia	1	1	165858.3	93982.65	40
1627	line*coccidia	1	2	282561	93982.65	40
1627	line*coccidia	1	3	785167.8	93982.65	40
1627	line*coccidia	2	0	365643.2	93982.65	40
1627	line*coccidia	2	1	107414	93982.65	40
1627	line*coccidia	2	2	411002.2	93982.65	40
1627	line*coccidia	2	3	681635.2	93982.65	40
1633	line	1		291542.8	86942.64	40
1633	line	2		341681.2	86942.64	40

1633	coccidia		0	227606.1	122955.5	40
1633	coccidia		1	363945.3	122955.5	40
1633	coccidia		2	161041.8	122955.5	40
1633	coccidia		3	513854.8	122955.5	40
1633	line*coccidia	1	0	153254	173885.3	40
1633	line*coccidia	1	1	174492.2	173885.3	40
1633	line*coccidia	1	2	141962	173885.3	40
1633	line*coccidia	1	3	696462.8	173885.3	40
1633	line*coccidia	2	0	301958.2	173885.3	40
1633	line*coccidia	2	1	553398.3	173885.3	40
1633	line*coccidia	2	2	180121.7	173885.3	40
1633	line*coccidia	2	3	331246.7	173885.3	40
1635	line	1		96974.75	70731.37	40
1635	line	2		258601.1	70731.37	40
1635	coccidia		0	414740.2	100029.3	40
1635	coccidia		1	98001.42	100029.3	40
1635	coccidia		2	77172.83	100029.3	40
1635	coccidia		3	121237.3	100029.3	40
1635	line*coccidia	1	0	202186.2	141462.7	40
1635	line*coccidia	1	1	1.46E-11	141462.7	40
1635	line*coccidia	1	2	29475.83	141462.7	40
1635	line*coccidia	1	3	156237	141462.7	40
1635	line*coccidia	2	0	627294.2	141462.7	40
1635	line*coccidia	2	1	196002.8	141462.7	40
1635	line*coccidia	2	2	124869.8	141462.7	40
1635	line*coccidia	2	3	86237.5	141462.7	40
1636	line	1		148349.6	105164.1	40
1636	line	2		538990.8	105164.1	40
1636	coccidia		0	695304.3	148724.5	40
1636	coccidia		1	259269.8	148724.5	40
1636	coccidia		2	97619.25	148724.5	40
1636	coccidia		3	322487.3	148724.5	40
1636	line*coccidia	1	0	295452.7	210328.2	40
1636	line*coccidia	1	1	7.28E-11	210328.2	40
1636	line*coccidia	1	2	41645.5	210328.2	40
1636	line*coccidia	1	3	256300.2	210328.2	40
1636	line*coccidia	2	0	1095156	210328.2	40
1636	line*coccidia	2	1	518539.7	210328.2	40
1636	line*coccidia	2	2	153593	210328.2	40
1636	line*coccidia	2	3	388674.3	210328.2	40
1637	line	1		432507.8	69106.91	40
1637	line	2		455145.6	69106.91	40
1637	coccidia		0	809861.8	97731.93	40
1637	coccidia		1	255994.4	97731.93	40
1637	coccidia		2	228187.3	97731.93	40
1637	coccidia		3	481263.2	97731.93	40
1637	line*coccidia	1	0	978968.3	138213.8	40
1637	line*coccidia	1	1	44534.67	138213.8	40
1637	line*coccidia	1	2	120618.8	138213.8	40

1637	line*coccidia	1	3	585909.2	138213.8	40
1637	line*coccidia	2	0	640755.3	138213.8	40
1637	line*coccidia	2	1	467454.2	138213.8	40
1637	line*coccidia	2	2	335755.8	138213.8	40
1637	line*coccidia	2	3	376617.2	138213.8	40
1638	line	1		271833.1	70451.3	40
1638	line	2		217910.1	70451.3	40
1638	coccidia		0	223083.8	99633.18	40
1638	coccidia		1	271424.9	99633.18	40
1638	coccidia		2	114018.3	99633.18	40
1638	coccidia		3	370959.4	99633.18	40
1638	line*coccidia	1	0	114061.5	140902.6	40
1638	line*coccidia	1	1	242937.2	140902.6	40
1638	line*coccidia	1	2	64089	140902.6	40
1638	line*coccidia	1	3	666244.8	140902.6	40
1638	line*coccidia	2	0	332106	140902.6	40
1638	line*coccidia	2	1	299912.7	140902.6	40
1638	line*coccidia	2	2	163947.7	140902.6	40
1638	line*coccidia	2	3	75674	140902.6	40
1642	line	1		279846.5	247546.5	40
1642	line	2		663613.7	247546.5	40
1642	coccidia		0	966519.3	350083.6	40
1642	coccidia		1	286939.4	350083.6	40
1642	coccidia		2	102669.6	350083.6	40
1642	coccidia		3	530792	350083.6	40
1642	line*coccidia	1	0	379524	495092.9	40
1642	line*coccidia	1	1	5.82E-11	495092.9	40
1642	line*coccidia	1	2	46107.5	495092.9	40
1642	line*coccidia	1	3	693754.3	495092.9	40
1642	line*coccidia	2	0	1553515	495092.9	40
1642	line*coccidia	2	1	573878.8	495092.9	40
1642	line*coccidia	2	2	159231.7	495092.9	40
1642	line*coccidia	2	3	367829.7	495092.9	40
1643	line	1		710684.4	229909.4	40
1643	line	2		308692.9	229909.4	40
1643	coccidia		0	341740.4	325141	40
1643	coccidia		1	1040488	325141	40
1643	coccidia		2	259103.6	325141	40
1643	coccidia		3	397422.3	325141	40
1643	line*coccidia	1	0	478749.7	459818.8	40
1643	line*coccidia	1	1	1373771	459818.8	40
1643	line*coccidia	1	2	397773.2	459818.8	40
1643	line*coccidia	1	3	592443.7	459818.8	40
1643	line*coccidia	2	0	204731.2	459818.8	40
1643	line*coccidia	2	1	707205.3	459818.8	40
1643	line*coccidia	2	2	120434	459818.8	40
1643	line*coccidia	2	3	202401	459818.8	40
1644	line	1		304424.5	150675.1	40
1644	line	2		446047.8	150675.1	40

1644	coccidia		0	502596.3	213086.8	40
1644	coccidia		1	728378.2	213086.8	40
1644	coccidia		2	102922	213086.8	40
1644	coccidia		3	167048	213086.8	40
1644	line*coccidia	1	0	458322.8	301350.2	40
1644	line*coccidia	1	1	451722.3	301350.2	40
1644	line*coccidia	1	2	79132.17	301350.2	40
1644	line*coccidia	1	3	228520.5	301350.2	40
1644	line*coccidia	2	0	546869.8	301350.2	40
1644	line*coccidia	2	1	1005034	301350.2	40
1644	line*coccidia	2	2	126711.8	301350.2	40
1644	line*coccidia	2	3	105575.5	301350.2	40
1702	line	1		1990470	337398.8	40
1702	line	2		1839799	337398.8	40
1702	coccidia		0	1812341	477154	40
1702	coccidia		1	920243.7	477154	40
1702	coccidia		2	2499944	477154	40
1702	coccidia		3	2428011	477154	40
1702	line*coccidia	1	0	1563786	674797.7	40
1702	line*coccidia	1	1	1416179	674797.7	40
1702	line*coccidia	1	2	1961113	674797.7	40
1702	line*coccidia	1	3	3020804	674797.7	40
1702	line*coccidia	2	0	2060896	674797.7	40
1702	line*coccidia	2	1	424308.2	674797.7	40
1702	line*coccidia	2	2	3038775	674797.7	40
1702	line*coccidia	2	3	1835218	674797.7	40
1707	line	1		973942	159023.9	40
1707	line	2		1926865	159023.9	40
1707	coccidia		0	1811301	224893.8	40
1707	coccidia		1	1178699	224893.8	40
1707	coccidia		2	1597813	224893.8	40
1707	coccidia		3	1213800	224893.8	40
1707	line*coccidia	1	0	1874354	318047.8	40
1707	line*coccidia	1	1	774452	318047.8	40
1707	line*coccidia	1	2	659343.2	318047.8	40
1707	line*coccidia	1	3	587618.8	318047.8	40
1707	line*coccidia	2	0	1748248	318047.8	40
1707	line*coccidia	2	1	1582946	318047.8	40
1707	line*coccidia	2	2	2536283	318047.8	40
1707	line*coccidia	2	3	1839982	318047.8	40
1713	line	1		1785494	377603.2	40
1713	line	2		2364963	377603.2	40
1713	coccidia		0	2735568	534011.6	40
1713	coccidia		1	1886023	534011.6	40
1713	coccidia		2	1505072	534011.6	40
1713	coccidia		3	2174253	534011.6	40
1713	line*coccidia	1	0	2648960	755206.5	40
1713	line*coccidia	1	1	450884.2	755206.5	40
1713	line*coccidia	1	2	748897.5	755206.5	40

1713	line*coccidia	1	3	3293236	755206.5	40
1713	line*coccidia	2	0	2822176	755206.5	40
1713	line*coccidia	2	1	3321161	755206.5	40
1713	line*coccidia	2	2	2261247	755206.5	40
1713	line*coccidia	2	3	1055269	755206.5	40
1724	line	1		2866508	401507	40
1724	line	2		3789083	401507	40
1724	coccidia		0	3391016	567816.6	40
1724	coccidia		1	2155669	567816.6	40
1724	coccidia		2	3543317	567816.6	40
1724	coccidia		3	4221181	567816.6	40
1724	line*coccidia	1	0	2756323	803013.9	40
1724	line*coccidia	1	1	1019888	803013.9	40
1724	line*coccidia	1	2	1141192	803013.9	40
1724	line*coccidia	1	3	6548630	803013.9	40
1724	line*coccidia	2	0	4025710	803013.9	40
1724	line*coccidia	2	1	3291449	803013.9	40
1724	line*coccidia	2	2	5945441	803013.9	40
1724	line*coccidia	2	3	1893732	803013.9	40
1726	line	1		3300177	407837	40
1726	line	2		2602229	407837	40
1726	coccidia		0	3681166	576768.6	40
1726	coccidia		1	2053923	576768.6	40
1726	coccidia		2	2166259	576768.6	40
1726	coccidia		3	3903464	576768.6	40
1726	line*coccidia	1	0	4360648	815674	40
1726	line*coccidia	1	1	2164078	815674	40
1726	line*coccidia	1	2	1112764	815674	40
1726	line*coccidia	1	3	5563219	815674	40
1726	line*coccidia	2	0	3001684	815674	40
1726	line*coccidia	2	1	1943768	815674	40
1726	line*coccidia	2	2	3219754	815674	40
1726	line*coccidia	2	3	2243710	815674	40
1731	line	1		838481.9	117593.9	40
1731	line	2		614632	117593.9	40
1731	coccidia		0	773321.5	166302.9	40
1731	coccidia		1	661815.4	166302.9	40
1731	coccidia		2	729014.5	166302.9	40
1731	coccidia		3	742076.3	166302.9	40
1731	line*coccidia	1	0	1012396	235187.9	40
1731	line*coccidia	1	1	708015.5	235187.9	40
1731	line*coccidia	1	2	427508.8	235187.9	40
1731	line*coccidia	1	3	1206008	235187.9	40
1731	line*coccidia	2	0	534247.5	235187.9	40
1731	line*coccidia	2	1	615615.3	235187.9	40
1731	line*coccidia	2	2	1030520	235187.9	40
1731	line*coccidia	2	3	278144.8	235187.9	40
1802	line	1		2058885	328853.1	40
1802	line	2		2898026	328853.1	40

1802	coccidia		0	2986840	465068.4	40
1802	coccidia		1	2346841	465068.4	40
1802	coccidia		2	1995681	465068.4	40
1802	coccidia		3	2584462	465068.4	40
1802	line*coccidia	1	0	2289348	657706.1	40
1802	line*coccidia	1	1	1448575	657706.1	40
1802	line*coccidia	1	2	871388.5	657706.1	40
1802	line*coccidia	1	3	3626229	657706.1	40
1802	line*coccidia	2	0	3684332	657706.1	40
1802	line*coccidia	2	1	3245107	657706.1	40
1802	line*coccidia	2	2	3119973	657706.1	40
1802	line*coccidia	2	3	1542695	657706.1	40
1808	line	1		1473941	312801.8	40
1808	line	2		2539300	312801.8	40
1808	coccidia		0	2064450	442368.6	40
1808	coccidia		1	2071710	442368.6	40
1808	coccidia		2	1105475	442368.6	40
1808	coccidia		3	2784848	442368.6	40
1808	line*coccidia	1	0	1760557	625603.6	40
1808	line*coccidia	1	1	1022168	625603.6	40
1808	line*coccidia	1	2	486193	625603.6	40
1808	line*coccidia	1	3	2626846	625603.6	40
1808	line*coccidia	2	0	2368343	625603.6	40
1808	line*coccidia	2	1	3121251	625603.6	40
1808	line*coccidia	2	2	1724758	625603.6	40
1808	line*coccidia	2	3	2942850	625603.6	40
1810	line	1		5169441	616421.8	40
1810	line	2		3569247	616421.8	40
1810	coccidia		0	6648509	871752.1	40
1810	coccidia		1	3421064	871752.1	40
1810	coccidia		2	1787910	871752.1	40
1810	coccidia		3	5619892	871752.1	40
1810	line*coccidia	1	0	7894745	1232844	40
1810	line*coccidia	1	1	1104481	1232844	40
1810	line*coccidia	1	2	1538057	1232844	40
1810	line*coccidia	1	3	10140480	1232844	40
1810	line*coccidia	2	0	5402272	1232844	40
1810	line*coccidia	2	1	5737647	1232844	40
1810	line*coccidia	2	2	2037763	1232844	40
1810	line*coccidia	2	3	1099304	1232844	40
1822	line	1		442778.6	59371.37	40
1822	line	2		473042.4	59371.37	40
1822	coccidia		0	559707.1	83963.8	40
1822	coccidia		1	477433.3	83963.8	40
1822	coccidia		2	340932.2	83963.8	40
1822	coccidia		3	453569.6	83963.8	40
1822	line*coccidia	1	0	683832.8	118742.7	40
1822	line*coccidia	1	1	465564.5	118742.7	40
1822	line*coccidia	1	2	254902.2	118742.7	40

1822	line*coccidia	1	3	366815	118742.7	40
1822	line*coccidia	2	0	435581.3	118742.7	40
1822	line*coccidia	2	1	489302	118742.7	40
1822	line*coccidia	2	2	426962.2	118742.7	40
1822	line*coccidia	2	3	540324.2	118742.7	40
1823	line	1		179250.8	26779.15	40
1823	line	2		207520	26779.15	40
1823	coccidia		0	292740.8	37871.44	40
1823	coccidia		1	121698.5	37871.44	40
1823	coccidia		2	137286.8	37871.44	40
1823	coccidia		3	221815.5	37871.44	40
1823	line*coccidia	1	0	294683	53558.31	40
1823	line*coccidia	1	1	108240.8	53558.31	40
1823	line*coccidia	1	2	38487.67	53558.31	40
1823	line*coccidia	1	3	275591.8	53558.31	40
1823	line*coccidia	2	0	290798.7	53558.31	40
1823	line*coccidia	2	1	135156.2	53558.31	40
1823	line*coccidia	2	2	236085.8	53558.31	40
1823	line*coccidia	2	3	168039.2	53558.31	40
1826	line	1		876328.5	139151.7	40
1826	line	2		1164242	139151.7	40
1826	coccidia		0	1162845	196790.2	40
1826	coccidia		1	1405707	196790.2	40
1826	coccidia		2	864418	196790.2	40
1826	coccidia		3	648171.4	196790.2	40
1826	line*coccidia	1	0	987597	278303.4	40
1826	line*coccidia	1	1	636459.8	278303.4	40
1826	line*coccidia	1	2	672142.8	278303.4	40
1826	line*coccidia	1	3	1209114	278303.4	40
1826	line*coccidia	2	0	1338093	278303.4	40
1826	line*coccidia	2	1	2174954	278303.4	40
1826	line*coccidia	2	2	1056693	278303.4	40
1826	line*coccidia	2	3	87228.5	278303.4	40
1832	line	1		5719813	741559.7	40
1832	line	2		4765086	741559.7	40
1832	coccidia		0	6832101	1048724	40
1832	coccidia		1	6721188	1048724	40
1832	coccidia		2	2836546	1048724	40
1832	coccidia		3	4579965	1048724	40
1832	line*coccidia	1	0	7850878	1483119	40
1832	line*coccidia	1	1	8273124	1483119	40
1832	line*coccidia	1	2	1593833	1483119	40
1832	line*coccidia	1	3	5161419	1483119	40
1832	line*coccidia	2	0	5813324	1483119	40
1832	line*coccidia	2	1	5169252	1483119	40
1832	line*coccidia	2	2	4079259	1483119	40
1832	line*coccidia	2	3	3998511	1483119	40
1917	line	1		1553828	255799.4	40
1917	line	2		1561854	255799.4	40

1917	coccidia		0	2677126	361755	40
1917	coccidia		1	1347310	361755	40
1917	coccidia		2	717932.3	361755	40
1917	coccidia		3	1488996	361755	40
1917	line*coccidia	1	0	3096111	511598.8	40
1917	line*coccidia	1	1	479986.3	511598.8	40
1917	line*coccidia	1	2	792798	511598.8	40
1917	line*coccidia	1	3	1846415	511598.8	40
1917	line*coccidia	2	0	2258140	511598.8	40
1917	line*coccidia	2	1	2214633	511598.8	40
1917	line*coccidia	2	2	643066.5	511598.8	40
1917	line*coccidia	2	3	1131577	511598.8	40
2002	line	1		25859039	1262357	40
2002	line	2		24616356	1262357	40
2002	coccidia		0	32377887	1785242	40
2002	coccidia		1	23208809	1785242	40
2002	coccidia		2	15246229	1785242	40
2002	coccidia		3	30117866	1785242	40
2002	line*coccidia	1	0	29093592	2524714	40
2002	line*coccidia	1	1	22628918	2524714	40
2002	line*coccidia	1	2	12389824	2524714	40
2002	line*coccidia	1	3	39323823	2524714	40
2002	line*coccidia	2	0	35662182	2524714	40
2002	line*coccidia	2	1	23788701	2524714	40
2002	line*coccidia	2	2	18102634	2524714	40
2002	line*coccidia	2	3	20911908	2524714	40
2003	line	1		6663693	595159.3	40
2003	line	2		6017144	595159.3	40
2003	coccidia		0	8677173	841682.4	40
2003	coccidia		1	5830272	841682.4	40
2003	coccidia		2	3658276	841682.4	40
2003	coccidia		3	7195953	841682.4	40
2003	line*coccidia	1	0	9772056	1190319	40
2003	line*coccidia	1	1	4324443	1190319	40
2003	line*coccidia	1	2	2852368	1190319	40
2003	line*coccidia	1	3	9705904	1190319	40
2003	line*coccidia	2	0	7582290	1190319	40
2003	line*coccidia	2	1	7336101	1190319	40
2003	line*coccidia	2	2	4464184	1190319	40
2003	line*coccidia	2	3	4686002	1190319	40
2004	line	1		50266.71	17520.43	40
2004	line	2		131009.8	17520.43	40
2004	coccidia		0	73186.42	24777.62	40
2004	coccidia		1	45757.92	24777.62	40
2004	coccidia		2	55747.25	24777.62	40
2004	coccidia		3	187861.5	24777.62	40
2004	line*coccidia	1	0	48472.33	35040.85	40
2004	line*coccidia	1	1	20364	35040.85	40
2004	line*coccidia	1	2	45526	35040.85	40

2004	line*coccidia	1	3	86704.5	35040.85	40
2004	line*coccidia	2	0	97900.5	35040.85	40
2004	line*coccidia	2	1	71151.83	35040.85	40
2004	line*coccidia	2	2	65968.5	35040.85	40
2004	line*coccidia	2	3	289018.5	35040.85	40
2007	line	1		471192.1	165392.5	40
2007	line	2		888224.8	165392.5	40
2007	coccidia		0	959077.3	233900.3	40
2007	coccidia		1	860447.6	233900.3	40
2007	coccidia		2	638639.3	233900.3	40
2007	coccidia		3	260669.6	233900.3	40
2007	line*coccidia	1	0	446453	330784.9	40
2007	line*coccidia	1	1	942882.8	330784.9	40
2007	line*coccidia	1	2	182045.7	330784.9	40
2007	line*coccidia	1	3	313386.8	330784.9	40
2007	line*coccidia	2	0	1471702	330784.9	40
2007	line*coccidia	2	1	778012.3	330784.9	40
2007	line*coccidia	2	2	1095233	330784.9	40
2007	line*coccidia	2	3	207952.3	330784.9	40
2008	line	1		979901.7	65450.17	40
2008	line	2		978008	65450.17	40
2008	coccidia		0	1309269	92560.52	40
2008	coccidia		1	912570.2	92560.52	40
2008	coccidia		2	655472	92560.52	40
2008	coccidia		3	1038508	92560.52	40
2008	line*coccidia	1	0	1321131	130900.3	40
2008	line*coccidia	1	1	682779.8	130900.3	40
2008	line*coccidia	1	2	413371.7	130900.3	40
2008	line*coccidia	1	3	1502325	130900.3	40
2008	line*coccidia	2	0	1297408	130900.3	40
2008	line*coccidia	2	1	1142361	130900.3	40
2008	line*coccidia	2	2	897572.3	130900.3	40
2008	line*coccidia	2	3	574691.5	130900.3	40
2009	line	1		426623.8	43860.58	40
2009	line	2		723734.8	43860.58	40
2009	coccidia		0	777019.3	62028.23	40
2009	coccidia		1	579933.9	62028.23	40
2009	coccidia		2	562769.6	62028.23	40
2009	coccidia		3	380994.5	62028.23	40
2009	line*coccidia	1	0	652343.5	87721.16	40
2009	line*coccidia	1	1	249159.3	87721.16	40
2009	line*coccidia	1	2	272720.2	87721.16	40
2009	line*coccidia	1	3	532272.2	87721.16	40
2009	line*coccidia	2	0	901695	87721.16	40
2009	line*coccidia	2	1	910708.5	87721.16	40
2009	line*coccidia	2	2	852819	87721.16	40
2009	line*coccidia	2	3	229716.8	87721.16	40
2011	line	1		1399335	73162.6	40
2011	line	2		1366937	73162.6	40

2011	coccidia		0	1778885	103467.5	40
2011	coccidia		1	1228819	103467.5	40
2011	coccidia		2	744134.4	103467.5	40
2011	coccidia		3	1780706	103467.5	40
2011	line*coccidia	1	0	1833686	146325.2	40
2011	line*coccidia	1	1	1037091	146325.2	40
2011	line*coccidia	1	2	484710.2	146325.2	40
2011	line*coccidia	1	3	2241855	146325.2	40
2011	line*coccidia	2	0	1724085	146325.2	40
2011	line*coccidia	2	1	1420546	146325.2	40
2011	line*coccidia	2	2	1003559	146325.2	40
2011	line*coccidia	2	3	1319557	146325.2	40
2014	line	1		57396.42	7970.695	40
2014	line	2		75272	7970.695	40
2014	coccidia		0	45466.17	11272.26	40
2014	coccidia		1	89796.5	11272.26	40
2014	coccidia		2	58081.17	11272.26	40
2014	coccidia		3	71993	11272.26	40
2014	line*coccidia	1	0	39224.67	15941.39	40
2014	line*coccidia	1	1	86609.67	15941.39	40
2014	line*coccidia	1	2	55776.17	15941.39	40
2014	line*coccidia	1	3	47975.17	15941.39	40
2014	line*coccidia	2	0	51707.67	15941.39	40
2014	line*coccidia	2	1	92983.33	15941.39	40
2014	line*coccidia	2	2	60386.17	15941.39	40
2014	line*coccidia	2	3	96010.83	15941.39	40
2015	line	1		7944924	509650.2	40
2015	line	2		8964406	509650.2	40
2015	coccidia		0	10989216	720754.2	40
2015	coccidia		1	6877510	720754.2	40
2015	coccidia		2	6529719	720754.2	40
2015	coccidia		3	9422214	720754.2	40
2015	line*coccidia	1	0	9939469	1019300	40
2015	line*coccidia	1	1	4430195	1019300	40
2015	line*coccidia	1	2	3928351	1019300	40
2015	line*coccidia	1	3	13481683	1019300	40
2015	line*coccidia	2	0	12038963	1019300	40
2015	line*coccidia	2	1	9324826	1019300	40
2015	line*coccidia	2	2	9131088	1019300	40
2015	line*coccidia	2	3	5362746	1019300	40
2016	line	1		7048472	674190.6	40
2016	line	2		7602572	674190.6	40
2016	coccidia		0	9496109	953449.5	40
2016	coccidia		1	8180116	953449.5	40
2016	coccidia		2	5230318	953449.5	40
2016	coccidia		3	6395544	953449.5	40
2016	line*coccidia	1	0	9375486	1348381	40
2016	line*coccidia	1	1	6341604	1348381	40
2016	line*coccidia	1	2	3167057	1348381	40

2016	line*coccidia	1	3	9309740	1348381	40
2016	line*coccidia	2	0	9616731	1348381	40
2016	line*coccidia	2	1	10018628	1348381	40
2016	line*coccidia	2	2	7293579	1348381	40
2016	line*coccidia	2	3	3481349	1348381	40
2017	line	1		147163.2	34808.07	40
2017	line	2		202327	34808.07	40
2017	coccidia		0	78995.83	49226.04	40
2017	coccidia		1	302111.6	49226.04	40
2017	coccidia		2	156764	49226.04	40
2017	coccidia		3	161109	49226.04	40
2017	line*coccidia	1	0	84270.17	69616.14	40
2017	line*coccidia	1	1	210769	69616.14	40
2017	line*coccidia	1	2	177084	69616.14	40
2017	line*coccidia	1	3	116529.7	69616.14	40
2017	line*coccidia	2	0	73721.5	69616.14	40
2017	line*coccidia	2	1	393454.2	69616.14	40
2017	line*coccidia	2	2	136444	69616.14	40
2017	line*coccidia	2	3	205688.3	69616.14	40
2018	line	1		203265.2	99002.87	40
2018	line	2		245481.6	99002.87	40
2018	coccidia		0	203880.8	140011.2	40
2018	coccidia		1	521775	140011.2	40
2018	coccidia		2	94996.25	140011.2	40
2018	coccidia		3	76841.67	140011.2	40
2018	line*coccidia	1	0	48328	198005.7	40
2018	line*coccidia	1	1	515841.5	198005.7	40
2018	line*coccidia	1	2	128587	198005.7	40
2018	line*coccidia	1	3	120304.3	198005.7	40
2018	line*coccidia	2	0	359433.5	198005.7	40
2018	line*coccidia	2	1	527708.5	198005.7	40
2018	line*coccidia	2	2	61405.5	198005.7	40
2018	line*coccidia	2	3	33379	198005.7	40
2020	line	1		55363	9703.693	40
2020	line	2		48564.5	9703.693	40
2020	coccidia		0	56380.5	13723.09	40
2020	coccidia		1	53146.08	13723.09	40
2020	coccidia		2	36517.67	13723.09	40
2020	coccidia		3	61810.75	13723.09	40
2020	line*coccidia	1	0	33451.17	19407.39	40
2020	line*coccidia	1	1	52372.5	19407.39	40
2020	line*coccidia	1	2	46228.33	19407.39	40
2020	line*coccidia	1	3	89400	19407.39	40
2020	line*coccidia	2	0	79309.83	19407.39	40
2020	line*coccidia	2	1	53919.67	19407.39	40
2020	line*coccidia	2	2	26807	19407.39	40
2020	line*coccidia	2	3	34221.5	19407.39	40
2028	line	1		254046.5	31893.76	40
2028	line	2		183704.5	31893.76	40

2028	coccidia		0	288821.1	45104.59	40
2028	coccidia		1	298188.3	45104.59	40
2028	coccidia		2	81872.75	45104.59	40
2028	coccidia		3	206619.9	45104.59	40
2028	line*coccidia	1	0	385862.5	63787.52	40
2028	line*coccidia	1	1	256234.8	63787.52	40
2028	line*coccidia	1	2	36789.83	63787.52	40
2028	line*coccidia	1	3	337299	63787.52	40
2028	line*coccidia	2	0	191779.7	63787.52	40
2028	line*coccidia	2	1	340141.8	63787.52	40
2028	line*coccidia	2	2	126955.7	63787.52	40
2028	line*coccidia	2	3	75940.83	63787.52	40
2034	line	1		115514.6	19150.48	40
2034	line	2		92237.17	19150.48	40
2034	coccidia		0	95131.83	27082.86	40
2034	coccidia		1	121372.6	27082.86	40
2034	coccidia		2	47412.75	27082.86	40
2034	coccidia		3	151586.4	27082.86	40
2034	line*coccidia	1	0	71588	38300.95	40
2034	line*coccidia	1	1	130636.7	38300.95	40
2034	line*coccidia	1	2	34396	38300.95	40
2034	line*coccidia	1	3	225437.8	38300.95	40
2034	line*coccidia	2	0	118675.7	38300.95	40
2034	line*coccidia	2	1	112108.5	38300.95	40
2034	line*coccidia	2	2	60429.5	38300.95	40
2034	line*coccidia	2	3	77735	38300.95	40
2041	line	1		68418740	5002937	40
2041	line	2		80109234	5002937	40
2041	coccidia		0	1.05E+08	7075221	40
2041	coccidia		1	69713141	7075221	40
2041	coccidia		2	57632767	7075221	40
2041	coccidia		3	64495451	7075221	40
2041	line*coccidia	1	0	78927337	10005873	40
2041	line*coccidia	1	1	62506990	10005873	40
2041	line*coccidia	1	2	51324413	10005873	40
2041	line*coccidia	1	3	80916220	10005873	40
2041	line*coccidia	2	0	1.32E+08	10005873	40
2041	line*coccidia	2	1	76919292	10005873	40
2041	line*coccidia	2	2	63941121	10005873	40
2041	line*coccidia	2	3	48074683	10005873	40
2044	line	1		39605.42	4842.822	40
2044	line	2		31356.46	4842.822	40
2044	coccidia		0	45076.67	6848.784	40
2044	coccidia		1	9150.667	6848.784	40
2044	coccidia		2	33397.25	6848.784	40
2044	coccidia		3	54299.17	6848.784	40
2044	line*coccidia	1	0	67911.83	9685.643	40
2044	line*coccidia	1	1	18301.33	9685.643	40
2044	line*coccidia	1	2	25209	9685.643	40

2044	line*coccidia	1	3	46999.5	9685.643	40
2044	line*coccidia	2	0	22241.5	9685.643	40
2044	line*coccidia	2	1	0	9685.643	40
2044	line*coccidia	2	2	41585.5	9685.643	40
2044	line*coccidia	2	3	61598.83	9685.643	40
2045	line	1		53410.67	125847.7	40
2045	line	2		255492.6	125847.7	40
2045	coccidia		0	60069.17	177975.5	40
2045	coccidia		1	441983.8	177975.5	40
2045	coccidia		2	45117.25	177975.5	40
2045	coccidia		3	70636.42	177975.5	40
2045	line*coccidia	1	0	35709.83	251695.4	40
2045	line*coccidia	1	1	92205	251695.4	40
2045	line*coccidia	1	2	32513.83	251695.4	40
2045	line*coccidia	1	3	53214	251695.4	40
2045	line*coccidia	2	0	84428.5	251695.4	40
2045	line*coccidia	2	1	791762.5	251695.4	40
2045	line*coccidia	2	2	57720.67	251695.4	40
2045	line*coccidia	2	3	88058.83	251695.4	40
2101	line	1		683101.3	57320.25	40
2101	line	2		465742	57320.25	40
2101	coccidia		0	684396.9	81063.07	40
2101	coccidia		1	467214.1	81063.07	40
2101	coccidia		2	489568.6	81063.07	40
2101	coccidia		3	656506.8	81063.07	40
2101	line*coccidia	1	0	794915.7	114640.5	40
2101	line*coccidia	1	1	406500.3	114640.5	40
2101	line*coccidia	1	2	479818.3	114640.5	40
2101	line*coccidia	1	3	1051171	114640.5	40
2101	line*coccidia	2	0	573878.2	114640.5	40
2101	line*coccidia	2	1	527927.8	114640.5	40
2101	line*coccidia	2	2	499318.8	114640.5	40
2101	line*coccidia	2	3	261843	114640.5	40
2102	line	1		1020107	93268.89	40
2102	line	2		620852.4	93268.89	40
2102	coccidia		0	997747.3	131902.1	40
2102	coccidia		1	869688.7	131902.1	40
2102	coccidia		2	439541.6	131902.1	40
2102	coccidia		3	974941.8	131902.1	40
2102	line*coccidia	1	0	1243440	186537.8	40
2102	line*coccidia	1	1	620832.3	186537.8	40
2102	line*coccidia	1	2	411717.5	186537.8	40
2102	line*coccidia	1	3	1804439	186537.8	40
2102	line*coccidia	2	0	752054.7	186537.8	40
2102	line*coccidia	2	1	1118545	186537.8	40
2102	line*coccidia	2	2	467365.7	186537.8	40
2102	line*coccidia	2	3	145444.3	186537.8	40
2103	line	1		2369944	191259.2	40
2103	line	2		728697.6	191259.2	40

2103	coccidia		0	1703042	270481.4	40
2103	coccidia		1	1553590	270481.4	40
2103	coccidia		2	895466.3	270481.4	40
2103	coccidia		3	2045185	270481.4	40
2103	line*coccidia	1	0	2489756	382518.4	40
2103	line*coccidia	1	1	2254615	382518.4	40
2103	line*coccidia	1	2	1104643	382518.4	40
2103	line*coccidia	1	3	3630764	382518.4	40
2103	line*coccidia	2	0	916329	382518.4	40
2103	line*coccidia	2	1	852565.7	382518.4	40
2103	line*coccidia	2	2	686290	382518.4	40
2103	line*coccidia	2	3	459605.7	382518.4	40
2104	line	1		701864.5	65386.48	40
2104	line	2		443323.6	65386.48	40
2104	coccidia		0	660821.2	92470.45	40
2104	coccidia		1	510753.8	92470.45	40
2104	coccidia		2	367582.9	92470.45	40
2104	coccidia		3	751218.3	92470.45	40
2104	line*coccidia	1	0	860407.3	130773	40
2104	line*coccidia	1	1	350857.3	130773	40
2104	line*coccidia	1	2	319824.3	130773	40
2104	line*coccidia	1	3	1276369	130773	40
2104	line*coccidia	2	0	461235	130773	40
2104	line*coccidia	2	1	670650.2	130773	40
2104	line*coccidia	2	2	415341.5	130773	40
2104	line*coccidia	2	3	226067.7	130773	40
2107	line	1		500459	48138.41	40
2107	line	2		180283.2	48138.41	40
2107	coccidia		0	384779.4	68078	40
2107	coccidia		1	279527.4	68078	40
2107	coccidia		2	232146.9	68078	40
2107	coccidia		3	465030.7	68078	40
2107	line*coccidia	1	0	559764.3	96276.83	40
2107	line*coccidia	1	1	333430.8	96276.83	40
2107	line*coccidia	1	2	284611.2	96276.83	40
2107	line*coccidia	1	3	824029.7	96276.83	40
2107	line*coccidia	2	0	209794.5	96276.83	40
2107	line*coccidia	2	1	225624	96276.83	40
2107	line*coccidia	2	2	179682.7	96276.83	40
2107	line*coccidia	2	3	106031.7	96276.83	40
2108	line	1		288094.6	23023.4	40
2108	line	2		188645.7	23023.4	40
2108	coccidia		0	328283.7	32560.01	40
2108	coccidia		1	207507	32560.01	40
2108	coccidia		2	125593.3	32560.01	40
2108	coccidia		3	292096.7	32560.01	40
2108	line*coccidia	1	0	391129.3	46046.8	40
2108	line*coccidia	1	1	189852	46046.8	40
2108	line*coccidia	1	2	92147	46046.8	40

2108	line*coccidia	1	3	479250.2	46046.8	40
2108	line*coccidia	2	0	265438	46046.8	40
2108	line*coccidia	2	1	225162	46046.8	40
2108	line*coccidia	2	2	159039.5	46046.8	40
2108	line*coccidia	2	3	104943.2	46046.8	40
2109	line	1		511918.4	82879.15	40
2109	line	2		1062467	82879.15	40
2109	coccidia		0	728146.5	117208.8	40
2109	coccidia		1	984041	117208.8	40
2109	coccidia		2	562959.8	117208.8	40
2109	coccidia		3	873622.7	117208.8	40
2109	line*coccidia	1	0	497410.3	165758.3	40
2109	line*coccidia	1	1	420757.3	165758.3	40
2109	line*coccidia	1	2	258025.3	165758.3	40
2109	line*coccidia	1	3	871480.5	165758.3	40
2109	line*coccidia	2	0	958882.7	165758.3	40
2109	line*coccidia	2	1	1547325	165758.3	40
2109	line*coccidia	2	2	867894.3	165758.3	40
2109	line*coccidia	2	3	875764.8	165758.3	40
2111	line	1		74372.33	9575.263	40
2111	line	2		52421.29	9575.263	40
2111	coccidia		0	54576.08	13541.47	40
2111	coccidia		1	101280.5	13541.47	40
2111	coccidia		2	58533.75	13541.47	40
2111	coccidia		3	39196.92	13541.47	40
2111	line*coccidia	1	0	47337	19150.53	40
2111	line*coccidia	1	1	142352.8	19150.53	40
2111	line*coccidia	1	2	64177	19150.53	40
2111	line*coccidia	1	3	43622.5	19150.53	40
2111	line*coccidia	2	0	61815.17	19150.53	40
2111	line*coccidia	2	1	60208.17	19150.53	40
2111	line*coccidia	2	2	52890.5	19150.53	40
2111	line*coccidia	2	3	34771.33	19150.53	40
2112	line	1		384836.6	70317.11	40
2112	line	2		216727.9	70317.11	40
2112	coccidia		0	294937.2	99443.41	40
2112	coccidia		1	321307.2	99443.41	40
2112	coccidia		2	108833.2	99443.41	40
2112	coccidia		3	478051.5	99443.41	40
2112	line*coccidia	1	0	304488.8	140634.2	40
2112	line*coccidia	1	1	307782.5	140634.2	40
2112	line*coccidia	1	2	82909.17	140634.2	40
2112	line*coccidia	1	3	844165.8	140634.2	40
2112	line*coccidia	2	0	285385.5	140634.2	40
2112	line*coccidia	2	1	334831.8	140634.2	40
2112	line*coccidia	2	2	134757.2	140634.2	40
2112	line*coccidia	2	3	111937.2	140634.2	40
2113	line	1		2916765	925357.8	40
2113	line	2		4860456	925357.8	40

2113	coccidia		0	6686135	1308654	40
2113	coccidia		1	3832639	1308654	40
2113	coccidia		2	2833883	1308654	40
2113	coccidia		3	2201784	1308654	40
2113	line*coccidia	1	0	2695707	1850716	40
2113	line*coccidia	1	1	4880704	1850716	40
2113	line*coccidia	1	2	1006847	1850716	40
2113	line*coccidia	1	3	3083803	1850716	40
2113	line*coccidia	2	0	10676564	1850716	40
2113	line*coccidia	2	1	2784575	1850716	40
2113	line*coccidia	2	2	4660920	1850716	40
2113	line*coccidia	2	3	1319765	1850716	40
2114	line	1		22814.58	6865.012	40
2114	line	2		41355.25	6865.012	40
2114	coccidia		0	27929.75	9708.593	40
2114	coccidia		1	33525	9708.593	40
2114	coccidia		2	26092.33	9708.593	40
2114	coccidia		3	40792.58	9708.593	40
2114	line*coccidia	1	0	17907.83	13730.02	40
2114	line*coccidia	1	1	11850.5	13730.02	40
2114	line*coccidia	1	2	19062.67	13730.02	40
2114	line*coccidia	1	3	42437.33	13730.02	40
2114	line*coccidia	2	0	37951.67	13730.02	40
2114	line*coccidia	2	1	55199.5	13730.02	40
2114	line*coccidia	2	2	33122	13730.02	40
2114	line*coccidia	2	3	39147.83	13730.02	40
2115	line	1		228248.1	63060.87	40
2115	line	2		543455.2	63060.87	40
2115	coccidia		0	268190.8	89181.54	40
2115	coccidia		1	501364.3	89181.54	40
2115	coccidia		2	248457.3	89181.54	40
2115	coccidia		3	525394.3	89181.54	40
2115	line*coccidia	1	0	124836.2	126121.7	40
2115	line*coccidia	1	1	165592.5	126121.7	40
2115	line*coccidia	1	2	94744.33	126121.7	40
2115	line*coccidia	1	3	527819.3	126121.7	40
2115	line*coccidia	2	0	411545.3	126121.7	40
2115	line*coccidia	2	1	837136	126121.7	40
2115	line*coccidia	2	2	402170.2	126121.7	40
2115	line*coccidia	2	3	522969.2	126121.7	40
2116	line	1		37087.5	15675.82	40
2116	line	2		56845.92	15675.82	40
2116	coccidia		0	24932.75	22168.95	40
2116	coccidia		1	85729.83	22168.95	40
2116	coccidia		2	42339.92	22168.95	40
2116	coccidia		3	34864.33	22168.95	40
2116	line*coccidia	1	0	18479.17	31351.63	40
2116	line*coccidia	1	1	38524.83	31351.63	40
2116	line*coccidia	1	2	39047.5	31351.63	40

2116	line*coccidia	1	3	52298.5	31351.63	40
2116	line*coccidia	2	0	31386.33	31351.63	40
2116	line*coccidia	2	1	132934.8	31351.63	40
2116	line*coccidia	2	2	45632.33	31351.63	40
2116	line*coccidia	2	3	17430.17	31351.63	40
2119	line	1		1157798	214216	40
2119	line	2		765240.8	214216	40
2119	coccidia		0	1040420	302947.2	40
2119	coccidia		1	917642.5	302947.2	40
2119	coccidia		2	436784.4	302947.2	40
2119	coccidia		3	1451231	302947.2	40
2119	line*coccidia	1	0	1285424	428432	40
2119	line*coccidia	1	1	599476.3	428432	40
2119	line*coccidia	1	2	462978.5	428432	40
2119	line*coccidia	1	3	2283313	428432	40
2119	line*coccidia	2	0	795415.7	428432	40
2119	line*coccidia	2	1	1235809	428432	40
2119	line*coccidia	2	2	410590.3	428432	40
2119	line*coccidia	2	3	619148.7	428432	40
2120	line	1		466148.9	48562.93	40
2120	line	2		308136.6	48562.93	40
2120	coccidia		0	539430.3	68678.35	40
2120	coccidia		1	308461.5	68678.35	40
2120	coccidia		2	358046.8	68678.35	40
2120	coccidia		3	342632.3	68678.35	40
2120	line*coccidia	1	0	614773.8	97125.86	40
2120	line*coccidia	1	1	318287	97125.86	40
2120	line*coccidia	1	2	384457.8	97125.86	40
2120	line*coccidia	1	3	547076.8	97125.86	40
2120	line*coccidia	2	0	464086.8	97125.86	40
2120	line*coccidia	2	1	298636	97125.86	40
2120	line*coccidia	2	2	331635.8	97125.86	40
2120	line*coccidia	2	3	138187.7	97125.86	40
2122	line	1		207622.3	25294.63	40
2122	line	2		276317	25294.63	40
2122	coccidia		0	324070.3	35772.01	40
2122	coccidia		1	231335.7	35772.01	40
2122	coccidia		2	237091.9	35772.01	40
2122	coccidia		3	175380.8	35772.01	40
2122	line*coccidia	1	0	251053.3	50589.26	40
2122	line*coccidia	1	1	66303.5	50589.26	40
2122	line*coccidia	1	2	242505.2	50589.26	40
2122	line*coccidia	1	3	270627.2	50589.26	40
2122	line*coccidia	2	0	397087.3	50589.26	40
2122	line*coccidia	2	1	396367.8	50589.26	40
2122	line*coccidia	2	2	231678.7	50589.26	40
2122	line*coccidia	2	3	80134.33	50589.26	40
2124	line	1		5691208	776651.3	40
2124	line	2		5781715	776651.3	40

2124	coccidia		0	8366446	1098351	40
2124	coccidia		1	4335862	1098351	40
2124	coccidia		2	3883673	1098351	40
2124	coccidia		3	6359864	1098351	40
2124	line*coccidia	1	0	7437869	1553303	40
2124	line*coccidia	1	1	3681018	1553303	40
2124	line*coccidia	1	2	1602566	1553303	40
2124	line*coccidia	1	3	10043378	1553303	40
2124	line*coccidia	2	0	9295023	1553303	40
2124	line*coccidia	2	1	4990706	1553303	40
2124	line*coccidia	2	2	6164781	1553303	40
2124	line*coccidia	2	3	2676351	1553303	40
2125	line	1		565833.6	59247.64	40
2125	line	2		296101.4	59247.64	40
2125	coccidia		0	557941.9	83788.81	40
2125	coccidia		1	448538.9	83788.81	40
2125	coccidia		2	214773.2	83788.81	40
2125	coccidia		3	502615.9	83788.81	40
2125	line*coccidia	1	0	732504.2	118495.3	40
2125	line*coccidia	1	1	434719.5	118495.3	40
2125	line*coccidia	1	2	206381.8	118495.3	40
2125	line*coccidia	1	3	889728.8	118495.3	40
2125	line*coccidia	2	0	383379.7	118495.3	40
2125	line*coccidia	2	1	462358.3	118495.3	40
2125	line*coccidia	2	2	223164.5	118495.3	40
2125	line*coccidia	2	3	115503	118495.3	40
2130	line	1		-4.5E-12	11732.93	40
2130	line	2		71400.17	11732.93	40
2130	coccidia		0	23857.67	16592.87	40
2130	coccidia		1	60733.08	16592.87	40
2130	coccidia		2	12022.83	16592.87	40
2130	coccidia		3	46186.75	16592.87	40
2130	line*coccidia	1	0	0	23465.86	40
2130	line*coccidia	1	1	-3.6E-12	23465.86	40
2130	line*coccidia	1	2	0	23465.86	40
2130	line*coccidia	1	3	-1.5E-11	23465.86	40
2130	line*coccidia	2	0	47715.33	23465.86	40
2130	line*coccidia	2	1	121466.2	23465.86	40
2130	line*coccidia	2	2	24045.67	23465.86	40
2130	line*coccidia	2	3	92373.5	23465.86	40
2132	line	1		356361.2	42456.89	40
2132	line	2		208967.9	42456.89	40
2132	coccidia		0	361031	60043.11	40
2132	coccidia		1	263790.2	60043.11	40
2132	coccidia		2	121133.7	60043.11	40
2132	coccidia		3	384703.3	60043.11	40
2132	line*coccidia	1	0	436109	84913.78	40
2132	line*coccidia	1	1	279918.7	84913.78	40
2132	line*coccidia	1	2	56535	84913.78	40

2132	line*coccidia	1	3	652882	84913.78	40
2132	line*coccidia	2	0	285953	84913.78	40
2132	line*coccidia	2	1	247661.7	84913.78	40
2132	line*coccidia	2	2	185732.3	84913.78	40
2132	line*coccidia	2	3	116524.7	84913.78	40
2133	line	1		47343.88	39026.38	40
2133	line	2		156168	39026.38	40
2133	coccidia		0	136560.8	55191.64	40
2133	coccidia		1	189625.8	55191.64	40
2133	coccidia		2	41590.33	55191.64	40
2133	coccidia		3	39246.67	55191.64	40
2133	line*coccidia	1	0	67231.17	78052.76	40
2133	line*coccidia	1	1	41694	78052.76	40
2133	line*coccidia	1	2	30686.17	78052.76	40
2133	line*coccidia	1	3	49764.17	78052.76	40
2133	line*coccidia	2	0	205890.5	78052.76	40
2133	line*coccidia	2	1	337557.7	78052.76	40
2133	line*coccidia	2	2	52494.5	78052.76	40
2133	line*coccidia	2	3	28729.17	78052.76	40
2201	line	1		286188.1	23859.64	40
2201	line	2		292346.7	23859.64	40
2201	coccidia		0	398643.6	33742.63	40
2201	coccidia		1	273934.8	33742.63	40
2201	coccidia		2	239825.8	33742.63	40
2201	coccidia		3	244665.5	33742.63	40
2201	line*coccidia	1	0	379710.8	47719.28	40
2201	line*coccidia	1	1	174527.8	47719.28	40
2201	line*coccidia	1	2	239124.3	47719.28	40
2201	line*coccidia	1	3	351389.3	47719.28	40
2201	line*coccidia	2	0	417576.3	47719.28	40
2201	line*coccidia	2	1	373341.7	47719.28	40
2201	line*coccidia	2	2	240527.2	47719.28	40
2201	line*coccidia	2	3	137941.7	47719.28	40
2205	line	1		5984875	381792.9	40
2205	line	2		3899602	381792.9	40
2205	coccidia		0	5964406	539936.8	40
2205	coccidia		1	4010302	539936.8	40
2205	coccidia		2	2862515	539936.8	40
2205	coccidia		3	6931731	539936.8	40
2205	line*coccidia	1	0	8491458	763585.9	40
2205	line*coccidia	1	1	3986778	763585.9	40
2205	line*coccidia	1	2	1433600	763585.9	40
2205	line*coccidia	1	3	10027665	763585.9	40
2205	line*coccidia	2	0	3437353	763585.9	40
2205	line*coccidia	2	1	4033826	763585.9	40
2205	line*coccidia	2	2	4291430	763585.9	40
2205	line*coccidia	2	3	3835797	763585.9	40
2206	line	1		364174.2	26956.12	40
2206	line	2		225652.2	26956.12	40

2206	coccidia		0	400103	38121.71	40
2206	coccidia		1	180487.7	38121.71	40
2206	coccidia		2	187321.2	38121.71	40
2206	coccidia		3	411740.9	38121.71	40
2206	line*coccidia	1	0	564289.2	53912.23	40
2206	line*coccidia	1	1	165082.5	53912.23	40
2206	line*coccidia	1	2	112379.3	53912.23	40
2206	line*coccidia	1	3	614945.8	53912.23	40
2206	line*coccidia	2	0	235916.8	53912.23	40
2206	line*coccidia	2	1	195892.8	53912.23	40
2206	line*coccidia	2	2	262263	53912.23	40
2206	line*coccidia	2	3	208536	53912.23	40
2207	line	1		667057.7	47535.88	40
2207	line	2		673796.8	47535.88	40
2207	coccidia		0	970774.1	67225.89	40
2207	coccidia		1	766783.9	67225.89	40
2207	coccidia		2	286920.8	67225.89	40
2207	coccidia		3	657230.2	67225.89	40
2207	line*coccidia	1	0	1011994	95071.77	40
2207	line*coccidia	1	1	497681.8	95071.77	40
2207	line*coccidia	1	2	217781.3	95071.77	40
2207	line*coccidia	1	3	940774.2	95071.77	40
2207	line*coccidia	2	0	929554.7	95071.77	40
2207	line*coccidia	2	1	1035886	95071.77	40
2207	line*coccidia	2	2	356060.3	95071.77	40
2207	line*coccidia	2	3	373686.2	95071.77	40
2208	line	1		288697.2	29354.96	40
2208	line	2		340786.5	29354.96	40
2208	coccidia		0	414261.2	41514.18	40
2208	coccidia		1	237836.9	41514.18	40
2208	coccidia		2	329857.3	41514.18	40
2208	coccidia		3	277011.9	41514.18	40
2208	line*coccidia	1	0	348015	58709.92	40
2208	line*coccidia	1	1	98635.33	58709.92	40
2208	line*coccidia	1	2	363778.7	58709.92	40
2208	line*coccidia	1	3	344359.7	58709.92	40
2208	line*coccidia	2	0	480507.3	58709.92	40
2208	line*coccidia	2	1	377038.5	58709.92	40
2208	line*coccidia	2	2	295936	58709.92	40
2208	line*coccidia	2	3	209664.2	58709.92	40
2210	line	1		539509.1	34509.04	40
2210	line	2		497564	34509.04	40
2210	coccidia		0	530991.8	48803.15	40
2210	coccidia		1	447255.8	48803.15	40
2210	coccidia		2	420846.8	48803.15	40
2210	coccidia		3	675051.8	48803.15	40
2210	line*coccidia	1	0	627917.7	69018.08	40
2210	line*coccidia	1	1	370905.2	69018.08	40
2210	line*coccidia	1	2	246148.3	69018.08	40

2210	line*coccidia	1	3	913065.3	69018.08	40
2210	line*coccidia	2	0	434066	69018.08	40
2210	line*coccidia	2	1	523606.3	69018.08	40
2210	line*coccidia	2	2	595545.3	69018.08	40
2210	line*coccidia	2	3	437038.2	69018.08	40
2212	line	1		179912.3	24815.85	40
2212	line	2		115158.8	24815.85	40
2212	coccidia		0	203260.8	35094.91	40
2212	coccidia		1	204001.9	35094.91	40
2212	coccidia		2	103465.2	35094.91	40
2212	coccidia		3	79414.25	35094.91	40
2212	line*coccidia	1	0	260145	49631.7	40
2212	line*coccidia	1	1	261896.8	49631.7	40
2212	line*coccidia	1	2	93728.5	49631.7	40
2212	line*coccidia	1	3	103878.7	49631.7	40
2212	line*coccidia	2	0	146376.5	49631.7	40
2212	line*coccidia	2	1	146107	49631.7	40
2212	line*coccidia	2	2	113201.8	49631.7	40
2212	line*coccidia	2	3	54949.83	49631.7	40
2215	line	1		471362.8	37819.77	40
2215	line	2		476607.2	37819.77	40
2215	coccidia		0	604968.4	53485.24	40
2215	coccidia		1	428994.9	53485.24	40
2215	coccidia		2	510359.8	53485.24	40
2215	coccidia		3	351616.8	53485.24	40
2215	line*coccidia	1	0	520120	75639.55	40
2215	line*coccidia	1	1	273099.8	75639.55	40
2215	line*coccidia	1	2	594932.2	75639.55	40
2215	line*coccidia	1	3	497299.3	75639.55	40
2215	line*coccidia	2	0	689816.8	75639.55	40
2215	line*coccidia	2	1	584890	75639.55	40
2215	line*coccidia	2	2	425787.5	75639.55	40
2215	line*coccidia	2	3	205934.3	75639.55	40
2217	line	1		13670.71	5225.333	40
2217	line	2		42002.21	5225.333	40
2217	coccidia		0	32010.92	7389.737	40
2217	coccidia		1	37530.67	7389.737	40
2217	coccidia		2	15136.33	7389.737	40
2217	coccidia		3	26667.92	7389.737	40
2217	line*coccidia	1	0	26323.67	10450.67	40
2217	line*coccidia	1	1	-5.5E-12	10450.67	40
2217	line*coccidia	1	2	0	10450.67	40
2217	line*coccidia	1	3	28359.17	10450.67	40
2217	line*coccidia	2	0	37698.17	10450.67	40
2217	line*coccidia	2	1	75061.33	10450.67	40
2217	line*coccidia	2	2	30272.67	10450.67	40
2217	line*coccidia	2	3	24976.67	10450.67	40
2218	line	1		618324.5	46349.14	40
2218	line	2		462467.1	46349.14	40

2218	coccidia		0	460239.3	65547.58	40
2218	coccidia		1	793690.4	65547.58	40
2218	coccidia		2	415477.8	65547.58	40
2218	coccidia		3	492175.6	65547.58	40
2218	line*coccidia	1	0	530544	92698.27	40
2218	line*coccidia	1	1	790685	92698.27	40
2218	line*coccidia	1	2	470444.7	92698.27	40
2218	line*coccidia	1	3	681624.2	92698.27	40
2218	line*coccidia	2	0	389934.7	92698.27	40
2218	line*coccidia	2	1	796695.8	92698.27	40
2218	line*coccidia	2	2	360510.8	92698.27	40
2218	line*coccidia	2	3	302727	92698.27	40
2220	line	1		138930.4	25134.45	40
2220	line	2		203698.3	25134.45	40
2220	coccidia		0	253114.6	35545.49	40
2220	coccidia		1	202937.9	35545.49	40
2220	coccidia		2	109114.7	35545.49	40
2220	coccidia		3	120090.3	35545.49	40
2220	line*coccidia	1	0	206034.7	50268.91	40
2220	line*coccidia	1	1	128900.3	50268.91	40
2220	line*coccidia	1	2	55254.67	50268.91	40
2220	line*coccidia	1	3	165531.8	50268.91	40
2220	line*coccidia	2	0	300194.5	50268.91	40
2220	line*coccidia	2	1	276975.5	50268.91	40
2220	line*coccidia	2	2	162974.7	50268.91	40
2220	line*coccidia	2	3	74648.67	50268.91	40
2222	line	1		1527870	141272.5	40
2222	line	2		1346499	141272.5	40
2222	coccidia		0	1668623	199789.5	40
2222	coccidia		1	1762454	199789.5	40
2222	coccidia		2	960824.7	199789.5	40
2222	coccidia		3	1356839	199789.5	40
2222	line*coccidia	1	0	1740789	282545	40
2222	line*coccidia	1	1	2063465	282545	40
2222	line*coccidia	1	2	498549.3	282545	40
2222	line*coccidia	1	3	1808678	282545	40
2222	line*coccidia	2	0	1596456	282545	40
2222	line*coccidia	2	1	1461442	282545	40
2222	line*coccidia	2	2	1423100	282545	40
2222	line*coccidia	2	3	904999.8	282545	40
2223	line	1		132347.4	10722.72	40
2223	line	2		105622	10722.72	40
2223	coccidia		0	150755.3	15164.21	40
2223	coccidia		1	140168.2	15164.21	40
2223	coccidia		2	76834.42	15164.21	40
2223	coccidia		3	108180.9	15164.21	40
2223	line*coccidia	1	0	179548.5	21445.44	40
2223	line*coccidia	1	1	116303.7	21445.44	40
2223	line*coccidia	1	2	77748.5	21445.44	40

2223	line*coccidia	1	3	155788.8	21445.44	40
2223	line*coccidia	2	0	121962	21445.44	40
2223	line*coccidia	2	1	164032.7	21445.44	40
2223	line*coccidia	2	2	75920.33	21445.44	40
2223	line*coccidia	2	3	60573	21445.44	40
2224	line	1		82435.08	16557.89	40
2224	line	2		77001.21	16557.89	40
2224	coccidia		0	119291.7	23416.39	40
2224	coccidia		1	74670.58	23416.39	40
2224	coccidia		2	39587	23416.39	40
2224	coccidia		3	85323.33	23416.39	40
2224	line*coccidia	1	0	114997.7	33115.78	40
2224	line*coccidia	1	1	72093	33115.78	40
2224	line*coccidia	1	2	28458.5	33115.78	40
2224	line*coccidia	1	3	114191.2	33115.78	40
2224	line*coccidia	2	0	123585.7	33115.78	40
2224	line*coccidia	2	1	77248.17	33115.78	40
2224	line*coccidia	2	2	50715.5	33115.78	40
2224	line*coccidia	2	3	56455.5	33115.78	40
2225	line	1		81431.08	24756.53	40
2225	line	2		132284.1	24756.53	40
2225	coccidia		0	130904	35011.02	40
2225	coccidia		1	140207.8	35011.02	40
2225	coccidia		2	82099.25	35011.02	40
2225	coccidia		3	74219.25	35011.02	40
2225	line*coccidia	1	0	91461.17	49513.07	40
2225	line*coccidia	1	1	61956.17	49513.07	40
2225	line*coccidia	1	2	58947.17	49513.07	40
2225	line*coccidia	1	3	113359.8	49513.07	40
2225	line*coccidia	2	0	170346.8	49513.07	40
2225	line*coccidia	2	1	218459.5	49513.07	40
2225	line*coccidia	2	2	105251.3	49513.07	40
2225	line*coccidia	2	3	35078.67	49513.07	40
2228	line	1		204287.8	41404.79	40
2228	line	2		27448.08	41404.79	40
2228	coccidia		0	26926.5	58555.22	40
2228	coccidia		1	399397.8	58555.22	40
2228	coccidia		2	12472.92	58555.22	40
2228	coccidia		3	24674.58	58555.22	40
2228	line*coccidia	1	0	26542	82809.58	40
2228	line*coccidia	1	1	768696	82809.58	40
2228	line*coccidia	1	2	-5.5E-12	82809.58	40
2228	line*coccidia	1	3	21913.33	82809.58	40
2228	line*coccidia	2	0	27311	82809.58	40
2228	line*coccidia	2	1	30099.67	82809.58	40
2228	line*coccidia	2	2	24945.83	82809.58	40
2228	line*coccidia	2	3	27435.83	82809.58	40
2230	line	1		81887.83	12434.65	40
2230	line	2		142778.4	12434.65	40

2230	coccidia		0	105520.5	17585.25	40
2230	coccidia		1	118491.8	17585.25	40
2230	coccidia		2	97696.75	17585.25	40
2230	coccidia		3	127623.4	17585.25	40
2230	line*coccidia	1	0	81877.17	24869.29	40
2230	line*coccidia	1	1	59290.67	24869.29	40
2230	line*coccidia	1	2	35624.67	24869.29	40
2230	line*coccidia	1	3	150758.8	24869.29	40
2230	line*coccidia	2	0	129163.8	24869.29	40
2230	line*coccidia	2	1	177692.8	24869.29	40
2230	line*coccidia	2	2	159768.8	24869.29	40
2230	line*coccidia	2	3	104488	24869.29	40
2232	line	1		305434.8	43312.71	40
2232	line	2		155070.8	43312.71	40
2232	coccidia		0	263434.8	61253.42	40
2232	coccidia		1	196657.8	61253.42	40
2232	coccidia		2	113824.8	61253.42	40
2232	coccidia		3	347093.6	61253.42	40
2232	line*coccidia	1	0	295563.7	86625.42	40
2232	line*coccidia	1	1	289767.7	86625.42	40
2232	line*coccidia	1	2	56552.5	86625.42	40
2232	line*coccidia	1	3	579855.3	86625.42	40
2232	line*coccidia	2	0	231306	86625.42	40
2232	line*coccidia	2	1	103548	86625.42	40
2232	line*coccidia	2	2	171097.2	86625.42	40
2232	line*coccidia	2	3	114331.8	86625.42	40
2233	line	1		40483.5	11964.49	40
2233	line	2		42761.29	11964.49	40
2233	coccidia		0	18770.58	16920.35	40
2233	coccidia		1	89874.08	16920.35	40
2233	coccidia		2	13984.17	16920.35	40
2233	coccidia		3	43860.75	16920.35	40
2233	line*coccidia	1	0	19029.83	23928.99	40
2233	line*coccidia	1	1	142904.2	23928.99	40
2233	line*coccidia	1	2	7.28E-12	23928.99	40
2233	line*coccidia	1	3	0	23928.99	40
2233	line*coccidia	2	0	18511.33	23928.99	40
2233	line*coccidia	2	1	36844	23928.99	40
2233	line*coccidia	2	2	27968.33	23928.99	40
2233	line*coccidia	2	3	87721.5	23928.99	40
2301	line	1		661978.3	71138.39	40
2301	line	2		514523.4	71138.39	40
2301	coccidia		0	691298.7	100604.9	40
2301	coccidia		1	540024.3	100604.9	40
2301	coccidia		2	350597.6	100604.9	40
2301	coccidia		3	771082.9	100604.9	40
2301	line*coccidia	1	0	671853.5	142276.8	40
2301	line*coccidia	1	1	435334.7	142276.8	40
2301	line*coccidia	1	2	310170.7	142276.8	40

2301	line*coccidia	1	3	1230555	142276.8	40
2301	line*coccidia	2	0	710743.8	142276.8	40
2301	line*coccidia	2	1	644714	142276.8	40
2301	line*coccidia	2	2	391024.5	142276.8	40
2301	line*coccidia	2	3	311611.3	142276.8	40
2302	line	1		1677178	203332.8	40
2302	line	2		1271105	203332.8	40
2302	coccidia		0	2261538	287556	40
2302	coccidia		1	1333316	287556	40
2302	coccidia		2	568634.3	287556	40
2302	coccidia		3	1733078	287556	40
2302	line*coccidia	1	0	2851057	406665.5	40
2302	line*coccidia	1	1	1354732	406665.5	40
2302	line*coccidia	1	2	279366.3	406665.5	40
2302	line*coccidia	1	3	2223557	406665.5	40
2302	line*coccidia	2	0	1672020	406665.5	40
2302	line*coccidia	2	1	1311900	406665.5	40
2302	line*coccidia	2	2	857902.2	406665.5	40
2302	line*coccidia	2	3	1242599	406665.5	40
2304	line	1		32790	25980.56	40
2304	line	2		215882.3	25980.56	40
2304	coccidia		0	58181.5	36742.06	40
2304	coccidia		1	192948.8	36742.06	40
2304	coccidia		2	125027	36742.06	40
2304	coccidia		3	121187.2	36742.06	40
2304	line*coccidia	1	0	-2.9E-11	51961.12	40
2304	line*coccidia	1	1	78298.17	51961.12	40
2304	line*coccidia	1	2	52861.83	51961.12	40
2304	line*coccidia	1	3	-2.9E-11	51961.12	40
2304	line*coccidia	2	0	116363	51961.12	40
2304	line*coccidia	2	1	307599.5	51961.12	40
2304	line*coccidia	2	2	197192.2	51961.12	40
2304	line*coccidia	2	3	242374.3	51961.12	40
2306	line	1		422793.4	61939.86	40
2306	line	2		505179.5	61939.86	40
2306	coccidia		0	523944.1	87596.18	40
2306	coccidia		1	489145.9	87596.18	40
2306	coccidia		2	366379.8	87596.18	40
2306	coccidia		3	476476	87596.18	40
2306	line*coccidia	1	0	385565.8	123879.7	40
2306	line*coccidia	1	1	378441.2	123879.7	40
2306	line*coccidia	1	2	233376.3	123879.7	40
2306	line*coccidia	1	3	693790.3	123879.7	40
2306	line*coccidia	2	0	662322.3	123879.7	40
2306	line*coccidia	2	1	599850.7	123879.7	40
2306	line*coccidia	2	2	499383.2	123879.7	40
2306	line*coccidia	2	3	259161.7	123879.7	40
2308	line	1		217491	49373.82	40
2308	line	2		118127.1	49373.82	40

2308	coccidia		0	121853.3	69825.12	40
2308	coccidia		1	352455.3	69825.12	40
2308	coccidia		2	82550.42	69825.12	40
2308	coccidia		3	114377.3	69825.12	40
2308	line*coccidia	1	0	135603.8	98747.64	40
2308	line*coccidia	1	1	469363.7	98747.64	40
2308	line*coccidia	1	2	107551.8	98747.64	40
2308	line*coccidia	1	3	157444.8	98747.64	40
2308	line*coccidia	2	0	108102.8	98747.64	40
2308	line*coccidia	2	1	235547	98747.64	40
2308	line*coccidia	2	2	57549	98747.64	40
2308	line*coccidia	2	3	71309.67	98747.64	40
2309	line	1		621899.3	134004.7	40
2309	line	2		460022.5	134004.7	40
2309	coccidia		0	880036.6	189511.3	40
2309	coccidia		1	550427.3	189511.3	40
2309	coccidia		2	264012.5	189511.3	40
2309	coccidia		3	469367.4	189511.3	40
2309	line*coccidia	1	0	1459843	268009.4	40
2309	line*coccidia	1	1	239264.5	268009.4	40
2309	line*coccidia	1	2	156857.8	268009.4	40
2309	line*coccidia	1	3	631632.2	268009.4	40
2309	line*coccidia	2	0	300230.3	268009.4	40
2309	line*coccidia	2	1	861590	268009.4	40
2309	line*coccidia	2	2	371167.2	268009.4	40
2309	line*coccidia	2	3	307102.7	268009.4	40
2311	line	1		61372.79	19235.89	40
2311	line	2		50633.63	19235.89	40
2311	coccidia		0	54772.25	27203.65	40
2311	coccidia		1	139669	27203.65	40
2311	coccidia		2	13881.5	27203.65	40
2311	coccidia		3	15690.08	27203.65	40
2311	line*coccidia	1	0	48921.33	38471.78	40
2311	line*coccidia	1	1	165189.7	38471.78	40
2311	line*coccidia	1	2	0	38471.78	40
2311	line*coccidia	1	3	31380.17	38471.78	40
2311	line*coccidia	2	0	60623.17	38471.78	40
2311	line*coccidia	2	1	114148.3	38471.78	40
2311	line*coccidia	2	2	27763	38471.78	40
2311	line*coccidia	2	3	2.55E-11	38471.78	40
2313	line	1		265709.1	63700.13	40
2313	line	2		394511	63700.13	40
2313	coccidia		0	405237.9	90085.59	40
2313	coccidia		1	430802.3	90085.59	40
2313	coccidia		2	204382.8	90085.59	40
2313	coccidia		3	280017.3	90085.59	40
2313	line*coccidia	1	0	403202.7	127400.3	40
2313	line*coccidia	1	1	238389.7	127400.3	40
2313	line*coccidia	1	2	94359.5	127400.3	40

2313	line*coccidia	1	3	326884.7	127400.3	40
2313	line*coccidia	2	0	407273.2	127400.3	40
2313	line*coccidia	2	1	623214.8	127400.3	40
2313	line*coccidia	2	2	314406.2	127400.3	40
2313	line*coccidia	2	3	233150	127400.3	40
2316	line	1		29237.54	36555.51	40
2316	line	2		99303.79	36555.51	40
2316	coccidia		0	53392.92	51697.3	40
2316	coccidia		1	115973.8	51697.3	40
2316	coccidia		2	17270.33	51697.3	40
2316	coccidia		3	70445.58	51697.3	40
2316	line*coccidia	1	0	29905.33	73111.02	40
2316	line*coccidia	1	1	-3.6E-12	73111.02	40
2316	line*coccidia	1	2	23738.83	73111.02	40
2316	line*coccidia	1	3	63306	73111.02	40
2316	line*coccidia	2	0	76880.5	73111.02	40
2316	line*coccidia	2	1	231947.7	73111.02	40
2316	line*coccidia	2	2	10801.83	73111.02	40
2316	line*coccidia	2	3	77585.17	73111.02	40
2317	line	1		1654145	354321.4	40
2317	line	2		894861.2	354321.4	40
2317	coccidia		0	998857.6	501086.1	40
2317	coccidia		1	2072793	501086.1	40
2317	coccidia		2	322460.4	501086.1	40
2317	coccidia		3	1703900	501086.1	40
2317	line*coccidia	1	0	828298.3	708642.7	40
2317	line*coccidia	1	1	3471572	708642.7	40
2317	line*coccidia	1	2	226985	708642.7	40
2317	line*coccidia	1	3	2089724	708642.7	40
2317	line*coccidia	2	0	1169417	708642.7	40
2317	line*coccidia	2	1	674014.8	708642.7	40
2317	line*coccidia	2	2	417935.8	708642.7	40
2317	line*coccidia	2	3	1318077	708642.7	40
2321	line	1		168279.2	39924.35	40
2321	line	2		126375.5	39924.35	40
2321	coccidia		0	190069.6	56461.56	40
2321	coccidia		1	132308.7	56461.56	40
2321	coccidia		2	97642.33	56461.56	40
2321	coccidia		3	169288.8	56461.56	40
2321	line*coccidia	1	0	258392.5	79848.71	40
2321	line*coccidia	1	1	-7.3E-12	79848.71	40
2321	line*coccidia	1	2	125937.5	79848.71	40
2321	line*coccidia	1	3	288786.8	79848.71	40
2321	line*coccidia	2	0	121746.7	79848.71	40
2321	line*coccidia	2	1	264617.3	79848.71	40
2321	line*coccidia	2	2	69347.17	79848.71	40
2321	line*coccidia	2	3	49790.83	79848.71	40
2402	line	1		288486.2	31391.74	40
2402	line	2		215776.3	31391.74	40

2402	coccidia		0	346182.6	44394.62	40
2402	coccidia		1	148203.5	44394.62	40
2402	coccidia		2	193430	44394.62	40
2402	coccidia		3	320708.8	44394.62	40
2402	line*coccidia	1	0	327097.3	62783.48	40
2402	line*coccidia	1	1	46830.67	62783.48	40
2402	line*coccidia	1	2	172844	62783.48	40
2402	line*coccidia	1	3	607172.7	62783.48	40
2402	line*coccidia	2	0	365267.8	62783.48	40
2402	line*coccidia	2	1	249576.3	62783.48	40
2402	line*coccidia	2	2	214016	62783.48	40
2402	line*coccidia	2	3	34245	62783.48	40
2404	line	1		1311858	368074.2	40
2404	line	2		1041215	368074.2	40
2404	coccidia		0	1339734	520535.6	40
2404	coccidia		1	1255952	520535.6	40
2404	coccidia		2	745405.6	520535.6	40
2404	coccidia		3	1365054	520535.6	40
2404	line*coccidia	1	0	728753.3	736148.5	40
2404	line*coccidia	1	1	2184332	736148.5	40
2404	line*coccidia	1	2	334396.3	736148.5	40
2404	line*coccidia	1	3	1999950	736148.5	40
2404	line*coccidia	2	0	1950715	736148.5	40
2404	line*coccidia	2	1	327573.2	736148.5	40
2404	line*coccidia	2	2	1156415	736148.5	40
2404	line*coccidia	2	3	730157.8	736148.5	40
2405	line	1		21059673	3022077	40
2405	line	2		22619633	3022077	40
2405	coccidia		0	21556382	4273862	40
2405	coccidia		1	18584938	4273862	40
2405	coccidia		2	17540895	4273862	40
2405	coccidia		3	29676398	4273862	40
2405	line*coccidia	1	0	15791214	6044154	40
2405	line*coccidia	1	1	20438914	6044154	40
2405	line*coccidia	1	2	17149633	6044154	40
2405	line*coccidia	1	3	30858931	6044154	40
2405	line*coccidia	2	0	27321550	6044154	40
2405	line*coccidia	2	1	16730962	6044154	40
2405	line*coccidia	2	2	17932158	6044154	40
2405	line*coccidia	2	3	28493864	6044154	40
2406	line	1		2773453	323674.4	40
2406	line	2		1694866	323674.4	40
2406	coccidia		0	2092881	457744.7	40
2406	coccidia		1	2274933	457744.7	40
2406	coccidia		2	856239.7	457744.7	40
2406	coccidia		3	3712584	457744.7	40
2406	line*coccidia	1	0	2367178	647348.8	40
2406	line*coccidia	1	1	3034751	647348.8	40
2406	line*coccidia	1	2	753008.5	647348.8	40

2406	line*coccidia	1	3	4938874	647348.8	40
2406	line*coccidia	2	0	1818585	647348.8	40
2406	line*coccidia	2	1	1515116	647348.8	40
2406	line*coccidia	2	2	959470.8	647348.8	40
2406	line*coccidia	2	3	2486293	647348.8	40
2407	line	1		27551842	4926517	40
2407	line	2		19768938	4926517	40
2407	coccidia		0	14505848	6967147	40
2407	coccidia		1	33831758	6967147	40
2407	coccidia		2	15758062	6967147	40
2407	coccidia		3	30545893	6967147	40
2407	line*coccidia	1	0	16178344	9853033	40
2407	line*coccidia	1	1	52904527	9853033	40
2407	line*coccidia	1	2	15848195	9853033	40
2407	line*coccidia	1	3	25276304	9853033	40
2407	line*coccidia	2	0	12833352	9853033	40
2407	line*coccidia	2	1	14758989	9853033	40
2407	line*coccidia	2	2	15667929	9853033	40
2407	line*coccidia	2	3	35815482	9853033	40
2408	line	1		19780873	2129437	40
2408	line	2		18746370	2129437	40
2408	coccidia		0	14132763	3011479	40
2408	coccidia		1	19690007	3011479	40
2408	coccidia		2	15379603	3011479	40
2408	coccidia		3	27852113	3011479	40
2408	line*coccidia	1	0	17138062	4258874	40
2408	line*coccidia	1	1	18857324	4258874	40
2408	line*coccidia	1	2	15340405	4258874	40
2408	line*coccidia	1	3	27787700	4258874	40
2408	line*coccidia	2	0	11127464	4258874	40
2408	line*coccidia	2	1	20522691	4258874	40
2408	line*coccidia	2	2	15418800	4258874	40
2408	line*coccidia	2	3	27916526	4258874	40
2409	line	1		2631228	344550	40
2409	line	2		1539687	344550	40
2409	coccidia		0	2147224	487267.3	40
2409	coccidia		1	2392707	487267.3	40
2409	coccidia		2	701503.5	487267.3	40
2409	coccidia		3	3100394	487267.3	40
2409	line*coccidia	1	0	2457042	689100.1	40
2409	line*coccidia	1	1	3430999	689100.1	40
2409	line*coccidia	1	2	540080.2	689100.1	40
2409	line*coccidia	1	3	4096789	689100.1	40
2409	line*coccidia	2	0	1837406	689100.1	40
2409	line*coccidia	2	1	1354414	689100.1	40
2409	line*coccidia	2	2	862926.8	689100.1	40
2409	line*coccidia	2	3	2103999	689100.1	40
2410	line	1		687843.2	100180.7	40
2410	line	2		237495.2	100180.7	40

2410	coccidia		0	504231.3	141677	40
2410	coccidia		1	877042.6	141677	40
2410	coccidia		2	101513	141677	40
2410	coccidia		3	367889.9	141677	40
2410	line*coccidia	1	0	491766.7	200361.5	40
2410	line*coccidia	1	1	1396175	200361.5	40
2410	line*coccidia	1	2	127651.3	200361.5	40
2410	line*coccidia	1	3	735779.8	200361.5	40
2410	line*coccidia	2	0	516695.8	200361.5	40
2410	line*coccidia	2	1	357910.3	200361.5	40
2410	line*coccidia	2	2	75374.67	200361.5	40
2410	line*coccidia	2	3	2.91E-11	200361.5	40
2412	line	1		464626.8	107974.5	40
2412	line	2		157237.8	107974.5	40
2412	coccidia		0	397260.6	152699	40
2412	coccidia		1	1.16E-10	152699	40
2412	coccidia		2	159214.3	152699	40
2412	coccidia		3	687254.3	152699	40
2412	line*coccidia	1	0	375402.7	215949	40
2412	line*coccidia	1	1	1.16E-10	215949	40
2412	line*coccidia	1	2	108596	215949	40
2412	line*coccidia	1	3	1374509	215949	40
2412	line*coccidia	2	0	419118.5	215949	40
2412	line*coccidia	2	1	1.16E-10	215949	40
2412	line*coccidia	2	2	209832.7	215949	40
2412	line*coccidia	2	3	2.91E-10	215949	40
2413	line	1		17127755	2274524	40
2413	line	2		26293032	2274524	40
2413	coccidia		0	23481161	3216663	40
2413	coccidia		1	28866403	3216663	40
2413	coccidia		2	15570362	3216663	40
2413	coccidia		3	18923648	3216663	40
2413	line*coccidia	1	0	19227787	4549048	40
2413	line*coccidia	1	1	20085627	4549048	40
2413	line*coccidia	1	2	9601209	4549048	40
2413	line*coccidia	1	3	19596397	4549048	40
2413	line*coccidia	2	0	27734535	4549048	40
2413	line*coccidia	2	1	37647178	4549048	40
2413	line*coccidia	2	2	21539515	4549048	40
2413	line*coccidia	2	3	18250899	4549048	40
2419	line	1		5612898	712908.2	40
2419	line	2		7419944	712908.2	40
2419	coccidia		0	8500178	1008204	40
2419	coccidia		1	5035508	1008204	40
2419	coccidia		2	7525305	1008204	40
2419	coccidia		3	5004693	1008204	40
2419	line*coccidia	1	0	9902346	1425816	40
2419	line*coccidia	1	1	3695225	1425816	40
2419	line*coccidia	1	2	2455401	1425816	40

2419	line*coccidia	1	3	6398621	1425816	40
2419	line*coccidia	2	0	7098010	1425816	40
2419	line*coccidia	2	1	6375792	1425816	40
2419	line*coccidia	2	2	12595210	1425816	40
2419	line*coccidia	2	3	3610766	1425816	40
2420	line	1		3267309	551371.1	40
2420	line	2		5269439	551371.1	40
2420	coccidia		0	6646451	779756.5	40
2420	coccidia		1	3275032	779756.5	40
2420	coccidia		2	5471180	779756.5	40
2420	coccidia		3	1680832	779756.5	40
2420	line*coccidia	1	0	8026891	1102742	40
2420	line*coccidia	1	1	1077641	1102742	40
2420	line*coccidia	1	2	1405556	1102742	40
2420	line*coccidia	1	3	2559148	1102742	40
2420	line*coccidia	2	0	5266012	1102742	40
2420	line*coccidia	2	1	5472423	1102742	40
2420	line*coccidia	2	2	9536803	1102742	40
2420	line*coccidia	2	3	802516.5	1102742	40
2422	line	1		2494030	979575.7	40
2422	line	2		963652.5	979575.7	40
2422	coccidia		0	1582105	1385329	40
2422	coccidia		1	3240133	1385329	40
2422	coccidia		2	546451.2	1385329	40
2422	coccidia		3	1546675	1385329	40
2422	line*coccidia	1	0	1283829	1959151	40
2422	line*coccidia	1	1	5803372	1959151	40
2422	line*coccidia	1	2	262605.3	1959151	40
2422	line*coccidia	1	3	2626313	1959151	40
2422	line*coccidia	2	0	1880381	1959151	40
2422	line*coccidia	2	1	676894.7	1959151	40
2422	line*coccidia	2	2	830297	1959151	40
2422	line*coccidia	2	3	467037.3	1959151	40
2423	line	1		41296.88	48802.69	40
2423	line	2		575819.6	48802.69	40
2423	coccidia		0	297649.7	69017.43	40
2423	coccidia		1	341378.6	69017.43	40
2423	coccidia		2	245325.7	69017.43	40
2423	coccidia		3	349879.1	69017.43	40
2423	line*coccidia	1	0	1.46E-11	97605.38	40
2423	line*coccidia	1	1	1.46E-11	97605.38	40
2423	line*coccidia	1	2	165187.5	97605.38	40
2423	line*coccidia	1	3	0	97605.38	40
2423	line*coccidia	2	0	595299.3	97605.38	40
2423	line*coccidia	2	1	682757.2	97605.38	40
2423	line*coccidia	2	2	325463.8	97605.38	40
2423	line*coccidia	2	3	699758.2	97605.38	40
2425	line	1		4121712	1015611	40
2425	line	2		875476.2	1015611	40

2425	coccidia		0	1554581	1436290	40
2425	coccidia		1	4772873	1436290	40
2425	coccidia		2	380695.3	1436290	40
2425	coccidia		3	3286226	1436290	40
2425	line*coccidia	1	0	1223426	2031221	40
2425	line*coccidia	1	1	8780047	2031221	40
2425	line*coccidia	1	2	394352.2	2031221	40
2425	line*coccidia	1	3	6089021	2031221	40
2425	line*coccidia	2	0	1885735	2031221	40
2425	line*coccidia	2	1	765699.8	2031221	40
2425	line*coccidia	2	2	367038.5	2031221	40
2425	line*coccidia	2	3	483431.5	2031221	40
2426	line	1		6526682	874302.3	40
2426	line	2		2327804	874302.3	40
2426	coccidia		0	4512184	1236450	40
2426	coccidia		1	6152539	1236450	40
2426	coccidia		2	3785850	1236450	40
2426	coccidia		3	3258399	1236450	40
2426	line*coccidia	1	0	5166922	1748605	40
2426	line*coccidia	1	1	10587005	1748605	40
2426	line*coccidia	1	2	4249648	1748605	40
2426	line*coccidia	1	3	6103153	1748605	40
2426	line*coccidia	2	0	3857446	1748605	40
2426	line*coccidia	2	1	1718074	1748605	40
2426	line*coccidia	2	2	3322051	1748605	40
2426	line*coccidia	2	3	413645.7	1748605	40
2427	line	1		3655401	922406.4	40
2427	line	2		1116632	922406.4	40
2427	coccidia		0	1104537	1304480	40
2427	coccidia		1	3749036	1304480	40
2427	coccidia		2	1724421	1304480	40
2427	coccidia		3	2966073	1304480	40
2427	line*coccidia	1	0	1285859	1844813	40
2427	line*coccidia	1	1	4832502	1844813	40
2427	line*coccidia	1	2	3082979	1844813	40
2427	line*coccidia	1	3	5420266	1844813	40
2427	line*coccidia	2	0	923215	1844813	40
2427	line*coccidia	2	1	2665570	1844813	40
2427	line*coccidia	2	2	365863.7	1844813	40
2427	line*coccidia	2	3	511880.5	1844813	40
2429	line	1		2734647	316806.3	40
2429	line	2		845065.2	316806.3	40
2429	coccidia		0	2862144	448031.8	40
2429	coccidia		1	1460879	448031.8	40
2429	coccidia		2	895249.7	448031.8	40
2429	coccidia		3	1941152	448031.8	40
2429	line*coccidia	1	0	4835567	633612.7	40
2429	line*coccidia	1	1	2508132	633612.7	40
2429	line*coccidia	1	2	1671047	633612.7	40

2429	line*coccidia	1	3	1923842	633612.7	40
2429	line*coccidia	2	0	888720.2	633612.7	40
2429	line*coccidia	2	1	413627	633612.7	40
2429	line*coccidia	2	2	119452.7	633612.7	40
2429	line*coccidia	2	3	1958461	633612.7	40
2501	line	1		101793.3	85301.53	40
2501	line	2		325891.1	85301.53	40
2501	coccidia		0	309469.6	120634.6	40
2501	coccidia		1	356343.3	120634.6	40
2501	coccidia		2	51613.67	120634.6	40
2501	coccidia		3	137942.3	120634.6	40
2501	line*coccidia	1	0	105901	170603.1	40
2501	line*coccidia	1	1	70008.67	170603.1	40
2501	line*coccidia	1	2	25055.67	170603.1	40
2501	line*coccidia	1	3	206208	170603.1	40
2501	line*coccidia	2	0	513038.2	170603.1	40
2501	line*coccidia	2	1	642678	170603.1	40
2501	line*coccidia	2	2	78171.67	170603.1	40
2501	line*coccidia	2	3	69676.5	170603.1	40
2504	line	1		904644	187890.4	40
2504	line	2		618130.2	187890.4	40
2504	coccidia		0	470480.4	265717.1	40
2504	coccidia		1	1665522	265717.1	40
2504	coccidia		2	193060.1	265717.1	40
2504	coccidia		3	716486	265717.1	40
2504	line*coccidia	1	0	630889.5	375780.7	40
2504	line*coccidia	1	1	1827326	375780.7	40
2504	line*coccidia	1	2	166300.7	375780.7	40
2504	line*coccidia	1	3	994059.5	375780.7	40
2504	line*coccidia	2	0	310071.3	375780.7	40
2504	line*coccidia	2	1	1503718	375780.7	40
2504	line*coccidia	2	2	219819.5	375780.7	40
2504	line*coccidia	2	3	438912.5	375780.7	40
2505	line	1		245164.1	63129.22	40
2505	line	2		374765.4	63129.22	40
2505	coccidia		0	310088.7	89278.2	40
2505	coccidia		1	468381.9	89278.2	40
2505	coccidia		2	238245.4	89278.2	40
2505	coccidia		3	223143	89278.2	40
2505	line*coccidia	1	0	389962.3	126258.4	40
2505	line*coccidia	1	1	66568.83	126258.4	40
2505	line*coccidia	1	2	177719.8	126258.4	40
2505	line*coccidia	1	3	346405.3	126258.4	40
2505	line*coccidia	2	0	230215	126258.4	40
2505	line*coccidia	2	1	870195	126258.4	40
2505	line*coccidia	2	2	298771	126258.4	40
2505	line*coccidia	2	3	99880.67	126258.4	40
2509	line	1		482277.7	82784.41	40
2509	line	2		635738	82784.41	40

2509	coccidia		0	626077.8	117074.8	40
2509	coccidia		1	783624.8	117074.8	40
2509	coccidia		2	294956.9	117074.8	40
2509	coccidia		3	531371.9	117074.8	40
2509	line*coccidia	1	0	678530.2	165568.8	40
2509	line*coccidia	1	1	377640.8	165568.8	40
2509	line*coccidia	1	2	209756.7	165568.8	40
2509	line*coccidia	1	3	663183.2	165568.8	40
2509	line*coccidia	2	0	573625.5	165568.8	40
2509	line*coccidia	2	1	1189609	165568.8	40
2509	line*coccidia	2	2	380157.2	165568.8	40
2509	line*coccidia	2	3	399560.7	165568.8	40
2510	line	1		827955.9	135319.7	40
2510	line	2		1037369	135319.7	40
2510	coccidia		0	1206920	191371	40
2510	coccidia		1	803108.4	191371	40
2510	coccidia		2	594262.8	191371	40
2510	coccidia		3	1126359	191371	40
2510	line*coccidia	1	0	1429000	270639.5	40
2510	line*coccidia	1	1	389180.5	270639.5	40
2510	line*coccidia	1	2	272557.5	270639.5	40
2510	line*coccidia	1	3	1221086	270639.5	40
2510	line*coccidia	2	0	984840.3	270639.5	40
2510	line*coccidia	2	1	1217036	270639.5	40
2510	line*coccidia	2	2	915968	270639.5	40
2510	line*coccidia	2	3	1031632	270639.5	40
2511	line	1		13433333	2907456	40
2511	line	2		7615428	2907456	40
2511	coccidia		0	9130792	4111763	40
2511	coccidia		1	10895588	4111763	40
2511	coccidia		2	12749179	4111763	40
2511	coccidia		3	9321964	4111763	40
2511	line*coccidia	1	0	8925389	5814912	40
2511	line*coccidia	1	1	16774966	5814912	40
2511	line*coccidia	1	2	13717359	5814912	40
2511	line*coccidia	1	3	14315620	5814912	40
2511	line*coccidia	2	0	9336195	5814912	40
2511	line*coccidia	2	1	5016211	5814912	40
2511	line*coccidia	2	2	11781000	5814912	40
2511	line*coccidia	2	3	4328308	5814912	40
2514	line	1		1094574	176177.5	40
2514	line	2		1278390	176177.5	40
2514	coccidia		0	2072240	249152.7	40
2514	coccidia		1	1082026	249152.7	40
2514	coccidia		2	795230.4	249152.7	40
2514	coccidia		3	796430.4	249152.7	40
2514	line*coccidia	1	0	2497148	352355.1	40
2514	line*coccidia	1	1	455427.7	352355.1	40
2514	line*coccidia	1	2	178359	352355.1	40

2514	line*coccidia	1	3	1247361	352355.1	40
2514	line*coccidia	2	0	1647332	352355.1	40
2514	line*coccidia	2	1	1708625	352355.1	40
2514	line*coccidia	2	2	1412102	352355.1	40
2514	line*coccidia	2	3	345500.2	352355.1	40
2515	line	1		361042.2	46116.93	40
2515	line	2		405461.3	46116.93	40
2515	coccidia		0	286982.4	65219.19	40
2515	coccidia		1	446958	65219.19	40
2515	coccidia		2	301939.3	65219.19	40
2515	coccidia		3	497127.3	65219.19	40
2515	line*coccidia	1	0	349036.2	92233.86	40
2515	line*coccidia	1	1	281338.8	92233.86	40
2515	line*coccidia	1	2	214847	92233.86	40
2515	line*coccidia	1	3	598946.7	92233.86	40
2515	line*coccidia	2	0	224928.7	92233.86	40
2515	line*coccidia	2	1	612577.2	92233.86	40
2515	line*coccidia	2	2	389031.5	92233.86	40
2515	line*coccidia	2	3	395308	92233.86	40
2516	line	1		1059503	359317.1	40
2516	line	2		1996208	359317.1	40
2516	coccidia		0	2197490	508151.1	40
2516	coccidia		1	1319074	508151.1	40
2516	coccidia		2	2013015	508151.1	40
2516	coccidia		3	581841.3	508151.1	40
2516	line*coccidia	1	0	2799124	718634.2	40
2516	line*coccidia	1	1	219058.8	718634.2	40
2516	line*coccidia	1	2	259870.7	718634.2	40
2516	line*coccidia	1	3	959957	718634.2	40
2516	line*coccidia	2	0	1595857	718634.2	40
2516	line*coccidia	2	1	2419089	718634.2	40
2516	line*coccidia	2	2	3766159	718634.2	40
2516	line*coccidia	2	3	203725.5	718634.2	40
2518	line	1		10744212	1285375	40
2518	line	2		14132772	1285375	40
2518	coccidia		0	20515625	1817794	40
2518	coccidia		1	12288737	1817794	40
2518	coccidia		2	10518991	1817794	40
2518	coccidia		3	6430615	1817794	40
2518	line*coccidia	1	0	19250337	2570749	40
2518	line*coccidia	1	1	11724289	2570749	40
2518	line*coccidia	1	2	3526815	2570749	40
2518	line*coccidia	1	3	8475408	2570749	40
2518	line*coccidia	2	0	21780913	2570749	40
2518	line*coccidia	2	1	12853185	2570749	40
2518	line*coccidia	2	2	17511167	2570749	40
2518	line*coccidia	2	3	4385822	2570749	40
2519	line	1		3830748	2332098	40
2519	line	2		8755923	2332098	40

2519	coccidia		0	11411562	3298085	40
2519	coccidia		1	3813771	3298085	40
2519	coccidia		2	6849256	3298085	40
2519	coccidia		3	3098754	3298085	40
2519	line*coccidia	1	0	6461226	4664196	40
2519	line*coccidia	1	1	3588653	4664196	40
2519	line*coccidia	1	2	1555672	4664196	40
2519	line*coccidia	1	3	3717442	4664196	40
2519	line*coccidia	2	0	16361898	4664196	40
2519	line*coccidia	2	1	4038889	4664196	40
2519	line*coccidia	2	2	12142841	4664196	40
2519	line*coccidia	2	3	2480066	4664196	40
2520	line	1		6014482	1319534	40
2520	line	2		6267322	1319534	40
2520	coccidia		0	7552889	1866103	40
2520	coccidia		1	4388719	1866103	40
2520	coccidia		2	7744922	1866103	40
2520	coccidia		3	4877077	1866103	40
2520	line*coccidia	1	0	11311836	2639068	40
2520	line*coccidia	1	1	3836628	2639068	40
2520	line*coccidia	1	2	2416209	2639068	40
2520	line*coccidia	1	3	6493257	2639068	40
2520	line*coccidia	2	0	3793942	2639068	40
2520	line*coccidia	2	1	4940811	2639068	40
2520	line*coccidia	2	2	13073636	2639068	40
2520	line*coccidia	2	3	3260898	2639068	40
2521	line	1		11106180	2478907	40
2521	line	2		14153143	2478907	40
2521	coccidia		0	15098614	3505703	40
2521	coccidia		1	7193171	3505703	40
2521	coccidia		2	11865033	3505703	40
2521	coccidia		3	16361828	3505703	40
2521	line*coccidia	1	0	9532178	4957813	40
2521	line*coccidia	1	1	3237869	4957813	40
2521	line*coccidia	1	2	5430759	4957813	40
2521	line*coccidia	1	3	26223914	4957813	40
2521	line*coccidia	2	0	20665051	4957813	40
2521	line*coccidia	2	1	11148473	4957813	40
2521	line*coccidia	2	2	18299307	4957813	40
2521	line*coccidia	2	3	6499741	4957813	40
2522	line	1		306704.3	53996.42	40
2522	line	2		367061.6	53996.42	40
2522	coccidia		0	204880.4	76362.47	40
2522	coccidia		1	305133.7	76362.47	40
2522	coccidia		2	330425.8	76362.47	40
2522	coccidia		3	507091.8	76362.47	40
2522	line*coccidia	1	0	208619.3	107992.8	40
2522	line*coccidia	1	1	236733.7	107992.8	40
2522	line*coccidia	1	2	259285.8	107992.8	40

2522	line*coccidia	1	3	522178.2	107992.8	40
2522	line*coccidia	2	0	201141.5	107992.8	40
2522	line*coccidia	2	1	373533.7	107992.8	40
2522	line*coccidia	2	2	401565.7	107992.8	40
2522	line*coccidia	2	3	492005.5	107992.8	40
2523	line	1		622039.7	129377.1	40
2523	line	2		784999.5	129377.1	40
2523	coccidia		0	435951.6	182966.8	40
2523	coccidia		1	1104585	182966.8	40
2523	coccidia		2	405967.7	182966.8	40
2523	coccidia		3	867574.4	182966.8	40
2523	line*coccidia	1	0	662950.5	258754.1	40
2523	line*coccidia	1	1	732398.7	258754.1	40
2523	line*coccidia	1	2	196733.2	258754.1	40
2523	line*coccidia	1	3	896076.3	258754.1	40
2523	line*coccidia	2	0	208952.7	258754.1	40
2523	line*coccidia	2	1	1476771	258754.1	40
2523	line*coccidia	2	2	615202.2	258754.1	40
2523	line*coccidia	2	3	839072.5	258754.1	40
2529	line	1		312259.2	83539.13	40
2529	line	2		534988.2	83539.13	40
2529	coccidia		0	687279.3	118142.2	40
2529	coccidia		1	398951.4	118142.2	40
2529	coccidia		2	361644.2	118142.2	40
2529	coccidia		3	246619.8	118142.2	40
2529	line*coccidia	1	0	536636.5	167078.3	40
2529	line*coccidia	1	1	151068.8	167078.3	40
2529	line*coccidia	1	2	158225.3	167078.3	40
2529	line*coccidia	1	3	403106	167078.3	40
2529	line*coccidia	2	0	837922.2	167078.3	40
2529	line*coccidia	2	1	646834	167078.3	40
2529	line*coccidia	2	2	565063	167078.3	40
2529	line*coccidia	2	3	90133.67	167078.3	40
2530	line	1		678103.3	137155.2	40
2530	line	2		541445.4	137155.2	40
2530	coccidia		0	383069.4	193966.7	40
2530	coccidia		1	729032.9	193966.7	40
2530	coccidia		2	300433.3	193966.7	40
2530	coccidia		3	1026562	193966.7	40
2530	line*coccidia	1	0	565145.3	274310.4	40
2530	line*coccidia	1	1	673217.2	274310.4	40
2530	line*coccidia	1	2	257931.2	274310.4	40
2530	line*coccidia	1	3	1216120	274310.4	40
2530	line*coccidia	2	0	200993.5	274310.4	40
2530	line*coccidia	2	1	784848.7	274310.4	40
2530	line*coccidia	2	2	342935.3	274310.4	40
2530	line*coccidia	2	3	837004.2	274310.4	40
2531	line	1		116202.5	83156.8	39
2531	line	2		258843.8	85210.36	39

2531	coccidia		0	568112.7	117601.5	39
2531	coccidia		1	210.9167	117601.5	39
2531	coccidia		2	109569.4	123341.5	39
2531	coccidia		3	72199.67	117601.5	39
2531	line*coccidia	1	0	243152.7	166313.6	39
2531	line*coccidia	1	1	-2.2E-11	166313.6	39
2531	line*coccidia	1	2	77258	166313.6	39
2531	line*coccidia	1	3	144399.3	166313.6	39
2531	line*coccidia	2	0	893072.7	166313.6	39
2531	line*coccidia	2	1	421.8333	166313.6	39
2531	line*coccidia	2	2	141880.8	182187.4	39
2531	line*coccidia	2	3	3.08E-11	166313.6	39
2603	line	1		1181511	185302.8	40
2603	line	2		1381372	185302.8	40
2603	coccidia		0	1987548	262057.7	40
2603	coccidia		1	1209755	262057.7	40
2603	coccidia		2	715494.7	262057.7	40
2603	coccidia		3	1212970	262057.7	40
2603	line*coccidia	1	0	1711166	370605.5	40
2603	line*coccidia	1	1	885103.8	370605.5	40
2603	line*coccidia	1	2	331036.5	370605.5	40
2603	line*coccidia	1	3	1798739	370605.5	40
2603	line*coccidia	2	0	2263929	370605.5	40
2603	line*coccidia	2	1	1534406	370605.5	40
2603	line*coccidia	2	2	1099953	370605.5	40
2603	line*coccidia	2	3	627200.7	370605.5	40
2610	line	1		120857.6	31779.64	40
2610	line	2		138658.8	31779.64	40
2610	coccidia		0	104303.6	44943.2	40
2610	coccidia		1	169754.4	44943.2	40
2610	coccidia		2	37785.33	44943.2	40
2610	coccidia		3	207189.6	44943.2	40
2610	line*coccidia	1	0	75657	63559.28	40
2610	line*coccidia	1	1	164254.8	63559.28	40
2610	line*coccidia	1	2	42785.67	63559.28	40
2610	line*coccidia	1	3	200733	63559.28	40
2610	line*coccidia	2	0	132950.2	63559.28	40
2610	line*coccidia	2	1	175254	63559.28	40
2610	line*coccidia	2	2	32785	63559.28	40
2610	line*coccidia	2	3	213646.2	63559.28	40
2613	line	1		84160.54	57183.66	40
2613	line	2		171861	57183.66	40
2613	coccidia		0	129195.8	80869.91	40
2613	coccidia		1	221449.8	80869.91	40
2613	coccidia		2	64035.75	80869.91	40
2613	coccidia		3	97361.75	80869.91	40
2613	line*coccidia	1	0	119072.2	114367.3	40
2613	line*coccidia	1	1	72244	114367.3	40
2613	line*coccidia	1	2	68005.5	114367.3	40

2613	line*coccidia	1	3	77320.5	114367.3	40
2613	line*coccidia	2	0	139319.5	114367.3	40
2613	line*coccidia	2	1	370655.5	114367.3	40
2613	line*coccidia	2	2	60066	114367.3	40
2613	line*coccidia	2	3	117403	114367.3	40
2616	line	1		573883.3	117763	40
2616	line	2		645761.2	117763	40
2616	coccidia		0	939658.9	166542.1	40
2616	coccidia		1	629601.1	166542.1	40
2616	coccidia		2	324888.7	166542.1	40
2616	coccidia		3	545140.2	166542.1	40
2616	line*coccidia	1	0	690129.8	235526.1	40
2616	line*coccidia	1	1	635201.2	235526.1	40
2616	line*coccidia	1	2	225396.5	235526.1	40
2616	line*coccidia	1	3	744805.5	235526.1	40
2616	line*coccidia	2	0	1189188	235526.1	40
2616	line*coccidia	2	1	624001	235526.1	40
2616	line*coccidia	2	2	424380.8	235526.1	40
2616	line*coccidia	2	3	345474.8	235526.1	40
2617	line	1		1085116	172303.7	40
2617	line	2		531319.5	172303.7	40
2617	coccidia		0	894191.1	243674.2	40
2617	coccidia		1	906111.8	243674.2	40
2617	coccidia		2	365356.9	243674.2	40
2617	coccidia		3	1067211	243674.2	40
2617	line*coccidia	1	0	1419175	344607.3	40
2617	line*coccidia	1	1	878588.3	344607.3	40
2617	line*coccidia	1	2	327553.3	344607.3	40
2617	line*coccidia	1	3	1715146	344607.3	40
2617	line*coccidia	2	0	369207	344607.3	40
2617	line*coccidia	2	1	933635.3	344607.3	40
2617	line*coccidia	2	2	403160.5	344607.3	40
2617	line*coccidia	2	3	419275	344607.3	40
2618	line	1		426225.9	138592.5	40
2618	line	2		194322.1	138592.5	40
2618	coccidia		0	332726.2	195999.4	40
2618	coccidia		1	316904.3	195999.4	40
2618	coccidia		2	84613.42	195999.4	40
2618	coccidia		3	506852	195999.4	40
2618	line*coccidia	1	0	298690.2	277185	40
2618	line*coccidia	1	1	483243	277185	40
2618	line*coccidia	1	2	84101.83	277185	40
2618	line*coccidia	1	3	838868.5	277185	40
2618	line*coccidia	2	0	366762.2	277185	40
2618	line*coccidia	2	1	150565.7	277185	40
2618	line*coccidia	2	2	85125	277185	40
2618	line*coccidia	2	3	174835.5	277185	40
2619	line	1		246794.1	63656.77	40
2619	line	2		351714.5	63656.77	40

2619	coccidia		0	330494.6	90024.27	40
2619	coccidia		1	356628	90024.27	40
2619	coccidia		2	202847.6	90024.27	40
2619	coccidia		3	307047.2	90024.27	40
2619	line*coccidia	1	0	405310.5	127313.5	40
2619	line*coccidia	1	1	98676.83	127313.5	40
2619	line*coccidia	1	2	167378.2	127313.5	40
2619	line*coccidia	1	3	315811	127313.5	40
2619	line*coccidia	2	0	255678.7	127313.5	40
2619	line*coccidia	2	1	614579.2	127313.5	40
2619	line*coccidia	2	2	238317	127313.5	40
2619	line*coccidia	2	3	298283.3	127313.5	40
2624	line	1		1490476	240824.9	40
2624	line	2		2228333	240824.9	40
2624	coccidia		0	2437996	340577.9	40
2624	coccidia		1	2294692	340577.9	40
2624	coccidia		2	1203665	340577.9	40
2624	coccidia		3	1501265	340577.9	40
2624	line*coccidia	1	0	2361891	481649.9	40
2624	line*coccidia	1	1	2040817	481649.9	40
2624	line*coccidia	1	2	259450.3	481649.9	40
2624	line*coccidia	1	3	1299746	481649.9	40
2624	line*coccidia	2	0	2514102	481649.9	40
2624	line*coccidia	2	1	2548567	481649.9	40
2624	line*coccidia	2	2	2147880	481649.9	40
2624	line*coccidia	2	3	1702783	481649.9	40
2626	line	1		3269721	590228	40
2626	line	2		3586349	590228	40
2626	coccidia		0	5259701	834708.5	40
2626	coccidia		1	3279950	834708.5	40
2626	coccidia		2	1767098	834708.5	40
2626	coccidia		3	3405390	834708.5	40
2626	line*coccidia	1	0	4624140	1180456	40
2626	line*coccidia	1	1	3962462	1180456	40
2626	line*coccidia	1	2	682435.5	1180456	40
2626	line*coccidia	1	3	3809846	1180456	40
2626	line*coccidia	2	0	5895262	1180456	40
2626	line*coccidia	2	1	2597438	1180456	40
2626	line*coccidia	2	2	2851761	1180456	40
2626	line*coccidia	2	3	3000935	1180456	40
2627	line	1		3794830	543205.2	40
2627	line	2		6265610	543205.2	40
2627	coccidia		0	6999082	768208.2	40
2627	coccidia		1	4123079	768208.2	40
2627	coccidia		2	4665309	768208.2	40
2627	coccidia		3	4333410	768208.2	40
2627	line*coccidia	1	0	5404843	1086410	40
2627	line*coccidia	1	1	4772149	1086410	40
2627	line*coccidia	1	2	1051302	1086410	40

2627	line*coccidia	1	3	3951028	1086410	40
2627	line*coccidia	2	0	8593321	1086410	40
2627	line*coccidia	2	1	3474010	1086410	40
2627	line*coccidia	2	2	8279316	1086410	40
2627	line*coccidia	2	3	4715793	1086410	40
2629	line	1		1205491	274533.1	40
2629	line	2		200362	274533.1	40
2629	coccidia		0	517531.2	388248.4	40
2629	coccidia		1	641505.5	388248.4	40
2629	coccidia		2	100039	388248.4	40
2629	coccidia		3	1552631	388248.4	40
2629	line*coccidia	1	0	901048.7	549066.2	40
2629	line*coccidia	1	1	914759.3	549066.2	40
2629	line*coccidia	1	2	126176.3	549066.2	40
2629	line*coccidia	1	3	2879980	549066.2	40
2629	line*coccidia	2	0	134013.7	549066.2	40
2629	line*coccidia	2	1	368251.7	549066.2	40
2629	line*coccidia	2	2	73901.67	549066.2	40
2629	line*coccidia	2	3	225281.2	549066.2	40
2630	line	1		274457.3	106006	40
2630	line	2		413761.5	106006	40
2630	coccidia		0	377162	149915.1	40
2630	coccidia		1	262381.8	149915.1	40
2630	coccidia		2	96162.33	149915.1	40
2630	coccidia		3	640731.4	149915.1	40
2630	line*coccidia	1	0	297080.8	212011.9	40
2630	line*coccidia	1	1	7.28E-11	212011.9	40
2630	line*coccidia	1	2	26361.5	212011.9	40
2630	line*coccidia	1	3	774386.7	212011.9	40
2630	line*coccidia	2	0	457243.2	212011.9	40
2630	line*coccidia	2	1	524763.5	212011.9	40
2630	line*coccidia	2	2	165963.2	212011.9	40
2630	line*coccidia	2	3	507076.2	212011.9	40
2701	line	1		2370896	426230.4	40
2701	line	2		2548526	426230.4	40
2701	coccidia		0	3239872	602780.8	40
2701	coccidia		1	1916712	602780.8	40
2701	coccidia		2	2053988	602780.8	40
2701	coccidia		3	2628272	602780.8	40
2701	line*coccidia	1	0	3832248	852460.8	40
2701	line*coccidia	1	1	815556.3	852460.8	40
2701	line*coccidia	1	2	711313.5	852460.8	40
2701	line*coccidia	1	3	4124465	852460.8	40
2701	line*coccidia	2	0	2647496	852460.8	40
2701	line*coccidia	2	1	3017868	852460.8	40
2701	line*coccidia	2	2	3396662	852460.8	40
2701	line*coccidia	2	3	1132078	852460.8	40
2703	line	1		2622806	543935.9	40
2703	line	2		3537235	543935.9	40

2703	coccidia		0	3228546	769241.5	40
2703	coccidia		1	2747386	769241.5	40
2703	coccidia		2	2442387	769241.5	40
2703	coccidia		3	3901763	769241.5	40
2703	line*coccidia	1	0	3254028	1087872	40
2703	line*coccidia	1	1	1868317	1087872	40
2703	line*coccidia	1	2	1029328	1087872	40
2703	line*coccidia	1	3	4339551	1087872	40
2703	line*coccidia	2	0	3203064	1087872	40
2703	line*coccidia	2	1	3626456	1087872	40
2703	line*coccidia	2	2	3855445	1087872	40
2703	line*coccidia	2	3	3463975	1087872	40
2705	line	1		866131.3	155034.4	40
2705	line	2		703018.8	155034.4	40
2705	coccidia		0	674928.3	219251.7	40
2705	coccidia		1	1234113	219251.7	40
2705	coccidia		2	362545.3	219251.7	40
2705	coccidia		3	866714.2	219251.7	40
2705	line*coccidia	1	0	584199.7	310068.7	40
2705	line*coccidia	1	1	1565302	310068.7	40
2705	line*coccidia	1	2	113805.7	310068.7	40
2705	line*coccidia	1	3	1201218	310068.7	40
2705	line*coccidia	2	0	765656.8	310068.7	40
2705	line*coccidia	2	1	902922.7	310068.7	40
2705	line*coccidia	2	2	611284.8	310068.7	40
2705	line*coccidia	2	3	532210.7	310068.7	40
2706	line	1		1608002	255693.7	40
2706	line	2		829762.1	255693.7	40
2706	coccidia		0	1599282	361605.5	40
2706	coccidia		1	2098649	361605.5	40
2706	coccidia		2	491679.5	361605.5	40
2706	coccidia		3	685918.4	361605.5	40
2706	line*coccidia	1	0	2324637	511387.3	40
2706	line*coccidia	1	1	3005015	511387.3	40
2706	line*coccidia	1	2	167677.2	511387.3	40
2706	line*coccidia	1	3	934680.3	511387.3	40
2706	line*coccidia	2	0	873927.3	511387.3	40
2706	line*coccidia	2	1	1192283	511387.3	40
2706	line*coccidia	2	2	815681.8	511387.3	40
2706	line*coccidia	2	3	437156.5	511387.3	40
2713	line	1		286336.2	47042.76	40
2713	line	2		135608.8	47042.76	40
2713	coccidia		0	216665.7	66528.51	40
2713	coccidia		1	211261.3	66528.51	40
2713	coccidia		2	201214.7	66528.51	40
2713	coccidia		3	214748.3	66528.51	40
2713	line*coccidia	1	0	292326.7	94085.52	40
2713	line*coccidia	1	1	255033.5	94085.52	40
2713	line*coccidia	1	2	262407.7	94085.52	40

2713	line*coccidia	1	3	335576.8	94085.52	40
2713	line*coccidia	2	0	141004.7	94085.52	40
2713	line*coccidia	2	1	167489	94085.52	40
2713	line*coccidia	2	2	140021.7	94085.52	40
2713	line*coccidia	2	3	93919.67	94085.52	40
2715	line	1		267589	58802.71	40
2715	line	2		172017.5	58802.71	40
2715	coccidia		0	246131.2	83159.59	40
2715	coccidia		1	290255.5	83159.59	40
2715	coccidia		2	207665.4	83159.59	40
2715	coccidia		3	135160.9	83159.59	40
2715	line*coccidia	1	0	388800	117605.4	40
2715	line*coccidia	1	1	183969.5	117605.4	40
2715	line*coccidia	1	2	299732	117605.4	40
2715	line*coccidia	1	3	197854.3	117605.4	40
2715	line*coccidia	2	0	103462.3	117605.4	40
2715	line*coccidia	2	1	396541.5	117605.4	40
2715	line*coccidia	2	2	115598.8	117605.4	40
2715	line*coccidia	2	3	72467.5	117605.4	40
2717	line	1		353300	70283.13	40
2717	line	2		219861.1	70283.13	40
2717	coccidia		0	335860.3	99395.35	40
2717	coccidia		1	316169.6	99395.35	40
2717	coccidia		2	321067.5	99395.35	40
2717	coccidia		3	173224.8	99395.35	40
2717	line*coccidia	1	0	466630.8	140566.3	40
2717	line*coccidia	1	1	292540.5	140566.3	40
2717	line*coccidia	1	2	396248.2	140566.3	40
2717	line*coccidia	1	3	257780.7	140566.3	40
2717	line*coccidia	2	0	205089.8	140566.3	40
2717	line*coccidia	2	1	339798.7	140566.3	40
2717	line*coccidia	2	2	245886.8	140566.3	40
2717	line*coccidia	2	3	88669	140566.3	40
2718	line	1		619194.6	118951.2	40
2718	line	2		476710.8	118951.2	40
2718	coccidia		0	660353.4	168222.4	40
2718	coccidia		1	623879.1	168222.4	40
2718	coccidia		2	576335	168222.4	40
2718	coccidia		3	331243.4	168222.4	40
2718	line*coccidia	1	0	811538.2	237902.4	40
2718	line*coccidia	1	1	495616.3	237902.4	40
2718	line*coccidia	1	2	693943.3	237902.4	40
2718	line*coccidia	1	3	475680.7	237902.4	40
2718	line*coccidia	2	0	509168.7	237902.4	40
2718	line*coccidia	2	1	752141.8	237902.4	40
2718	line*coccidia	2	2	458726.7	237902.4	40
2718	line*coccidia	2	3	186806.2	237902.4	40
2721	line	1		875524.8	140623.4	40
2721	line	2		482059.7	140623.4	40

2721	coccidia		0	663759.4	198871.5	40
2721	coccidia		1	890633.9	198871.5	40
2721	coccidia		2	422902.3	198871.5	40
2721	coccidia		3	737873.4	198871.5	40
2721	line*coccidia	1	0	910212	281246.8	40
2721	line*coccidia	1	1	988030.3	281246.8	40
2721	line*coccidia	1	2	478461.8	281246.8	40
2721	line*coccidia	1	3	1125395	281246.8	40
2721	line*coccidia	2	0	417306.8	281246.8	40
2721	line*coccidia	2	1	793237.5	281246.8	40
2721	line*coccidia	2	2	367342.7	281246.8	40
2721	line*coccidia	2	3	350351.8	281246.8	40
2722	line	1		816071.4	175419.9	40
2722	line	2		981037	175419.9	40
2722	coccidia		0	1092112	248081.2	40
2722	coccidia		1	1131149	248081.2	40
2722	coccidia		2	793593	248081.2	40
2722	coccidia		3	577362.8	248081.2	40
2722	line*coccidia	1	0	907654.7	350839.8	40
2722	line*coccidia	1	1	913398.8	350839.8	40
2722	line*coccidia	1	2	643304.2	350839.8	40
2722	line*coccidia	1	3	799927.8	350839.8	40
2722	line*coccidia	2	0	1276570	350839.8	40
2722	line*coccidia	2	1	1348898	350839.8	40
2722	line*coccidia	2	2	943881.8	350839.8	40
2722	line*coccidia	2	3	354797.8	350839.8	40
2723	line	1		2147387	328973.9	40
2723	line	2		1203685	328973.9	40
2723	coccidia		0	2726850	465239.4	40
2723	coccidia		1	1864050	465239.4	40
2723	coccidia		2	917028.3	465239.4	40
2723	coccidia		3	1194216	465239.4	40
2723	line*coccidia	1	0	4172498	657947.9	40
2723	line*coccidia	1	1	1675913	657947.9	40
2723	line*coccidia	1	2	773369.3	657947.9	40
2723	line*coccidia	1	3	1967769	657947.9	40
2723	line*coccidia	2	0	1281203	657947.9	40
2723	line*coccidia	2	1	2052188	657947.9	40
2723	line*coccidia	2	2	1060687	657947.9	40
2723	line*coccidia	2	3	420662.3	657947.9	40
2724	line	1		2077339	403512.4	40
2724	line	2		1919926	403512.4	40
2724	coccidia		0	2665204	570652.8	40
2724	coccidia		1	1330353	570652.8	40
2724	coccidia		2	1180359	570652.8	40
2724	coccidia		3	2818614	570652.8	40
2724	line*coccidia	1	0	3360309	807024.9	40
2724	line*coccidia	1	1	845538.3	807024.9	40
2724	line*coccidia	1	2	870296.7	807024.9	40

2724	line*coccidia	1	3	3233212	807024.9	40
2724	line*coccidia	2	0	1970100	807024.9	40
2724	line*coccidia	2	1	1815168	807024.9	40
2724	line*coccidia	2	2	1490420	807024.9	40
2724	line*coccidia	2	3	2404015	807024.9	40
2725	line	1		1563721	419693.3	40
2725	line	2		2133792	419693.3	40
2725	coccidia		0	1971747	593536	40
2725	coccidia		1	2106046	593536	40
2725	coccidia		2	1468907	593536	40
2725	coccidia		3	1848325	593536	40
2725	line*coccidia	1	0	964468.7	839386.6	40
2725	line*coccidia	1	1	1691024	839386.6	40
2725	line*coccidia	1	2	793426.5	839386.6	40
2725	line*coccidia	1	3	2805964	839386.6	40
2725	line*coccidia	2	0	2979025	839386.6	40
2725	line*coccidia	2	1	2521068	839386.6	40
2725	line*coccidia	2	2	2144388	839386.6	40
2725	line*coccidia	2	3	890686.7	839386.6	40
2726	line	1		1183157	182946.8	40
2726	line	2		1324947	182946.8	40
2726	coccidia		0	1644435	258725.8	40
2726	coccidia		1	1167826	258725.8	40
2726	coccidia		2	1071715	258725.8	40
2726	coccidia		3	1132232	258725.8	40
2726	line*coccidia	1	0	1531349	365893.6	40
2726	line*coccidia	1	1	1132575	365893.6	40
2726	line*coccidia	1	2	875198.3	365893.6	40
2726	line*coccidia	1	3	1193505	365893.6	40
2726	line*coccidia	2	0	1757521	365893.6	40
2726	line*coccidia	2	1	1203078	365893.6	40
2726	line*coccidia	2	2	1268232	365893.6	40
2726	line*coccidia	2	3	1070958	365893.6	40
2727	line	1		2539502	392620.9	40
2727	line	2		1639396	392620.9	40
2727	coccidia		0	2261388	555249.8	40
2727	coccidia		1	1778832	555249.8	40
2727	coccidia		2	1160613	555249.8	40
2727	coccidia		3	3156962	555249.8	40
2727	line*coccidia	1	0	2864348	785241.8	40
2727	line*coccidia	1	1	1913466	785241.8	40
2727	line*coccidia	1	2	696574.8	785241.8	40
2727	line*coccidia	1	3	4683618	785241.8	40
2727	line*coccidia	2	0	1658428	785241.8	40
2727	line*coccidia	2	1	1644199	785241.8	40
2727	line*coccidia	2	2	1624652	785241.8	40
2727	line*coccidia	2	3	1630306	785241.8	40
2728	line	1		3974898	658982	40
2728	line	2		4366951	658982	40

2728	coccidia		0	5791836	931941.3	40
2728	coccidia		1	4841623	931941.3	40
2728	coccidia		2	1335334	931941.3	40
2728	coccidia		3	4714904	931941.3	40
2728	line*coccidia	1	0	8752036	1317964	40
2728	line*coccidia	1	1	2451829	1317964	40
2728	line*coccidia	1	2	462016.3	1317964	40
2728	line*coccidia	1	3	4233710	1317964	40
2728	line*coccidia	2	0	2831637	1317964	40
2728	line*coccidia	2	1	7231417	1317964	40
2728	line*coccidia	2	2	2208652	1317964	40
2728	line*coccidia	2	3	5196099	1317964	40
2729	line	1		3641050	515164.8	40
2729	line	2		2683572	515164.8	40
2729	coccidia		0	3879342	728553.1	40
2729	coccidia		1	3175020	728553.1	40
2729	coccidia		2	1654746	728553.1	40
2729	coccidia		3	3940136	728553.1	40
2729	line*coccidia	1	0	5516102	1030330	40
2729	line*coccidia	1	1	2587675	1030330	40
2729	line*coccidia	1	2	1010817	1030330	40
2729	line*coccidia	1	3	5449607	1030330	40
2729	line*coccidia	2	0	2242583	1030330	40
2729	line*coccidia	2	1	3762365	1030330	40
2729	line*coccidia	2	2	2298676	1030330	40
2729	line*coccidia	2	3	2430665	1030330	40
2733	line	1		684904.8	161313.3	40
2733	line	2		315209.7	161313.3	40
2733	coccidia		0	464081.7	228131.5	40
2733	coccidia		1	400964.2	228131.5	40
2733	coccidia		2	312708	228131.5	40
2733	coccidia		3	822475.2	228131.5	40
2733	line*coccidia	1	0	664955	322626.7	40
2733	line*coccidia	1	1	411055.7	322626.7	40
2733	line*coccidia	1	2	511856.5	322626.7	40
2733	line*coccidia	1	3	1151752	322626.7	40
2733	line*coccidia	2	0	263208.3	322626.7	40
2733	line*coccidia	2	1	390872.7	322626.7	40
2733	line*coccidia	2	2	113559.5	322626.7	40
2733	line*coccidia	2	3	493198.2	322626.7	40
2735	line	1		1169770	302862.4	40
2735	line	2		1412695	302862.4	40
2735	coccidia		0	1696585	428312.2	40
2735	coccidia		1	1502018	428312.2	40
2735	coccidia		2	618065.5	428312.2	40
2735	coccidia		3	1348262	428312.2	40
2735	line*coccidia	1	0	2050793	605724.9	40
2735	line*coccidia	1	1	1693499	605724.9	40
2735	line*coccidia	1	2	246657.2	605724.9	40

2735	line*coccidia	1	3	688130.3	605724.9	40
2735	line*coccidia	2	0	1342377	605724.9	40
2735	line*coccidia	2	1	1310538	605724.9	40
2735	line*coccidia	2	2	989473.8	605724.9	40
2735	line*coccidia	2	3	2008393	605724.9	40
2818	line	1		731955.2	142363.9	40
2818	line	2		754503.6	142363.9	40
2818	coccidia		0	847975.3	201333	40
2818	coccidia		1	1109867	201333	40
2818	coccidia		2	311096.3	201333	40
2818	coccidia		3	703978.6	201333	40
2818	line*coccidia	1	0	1156258	284727.8	40
2818	line*coccidia	1	1	597544.5	284727.8	40
2818	line*coccidia	1	2	300332.8	284727.8	40
2818	line*coccidia	1	3	873685.5	284727.8	40
2818	line*coccidia	2	0	539692.7	284727.8	40
2818	line*coccidia	2	1	1622190	284727.8	40
2818	line*coccidia	2	2	321859.8	284727.8	40
2818	line*coccidia	2	3	534271.7	284727.8	40
2820	line	1		961969.1	133241.6	40
2820	line	2		773501.5	133241.6	40
2820	coccidia		0	1374001	188432	40
2820	coccidia		1	834431.9	188432	40
2820	coccidia		2	491749.4	188432	40
2820	coccidia		3	770759.1	188432	40
2820	line*coccidia	1	0	1868736	266483.1	40
2820	line*coccidia	1	1	722595.8	266483.1	40
2820	line*coccidia	1	2	345452.2	266483.1	40
2820	line*coccidia	1	3	911092.3	266483.1	40
2820	line*coccidia	2	0	879265.5	266483.1	40
2820	line*coccidia	2	1	946268	266483.1	40
2820	line*coccidia	2	2	638046.7	266483.1	40
2820	line*coccidia	2	3	630425.8	266483.1	40
2823	line	1		782917.9	176089.4	40
2823	line	2		1016501	176089.4	40
2823	coccidia		0	878187.4	249028	40
2823	coccidia		1	1262349	249028	40
2823	coccidia		2	735750.5	249028	40
2823	coccidia		3	722550.6	249028	40
2823	line*coccidia	1	0	927442.2	352178.8	40
2823	line*coccidia	1	1	999816.8	352178.8	40
2823	line*coccidia	1	2	618646.8	352178.8	40
2823	line*coccidia	1	3	585765.8	352178.8	40
2823	line*coccidia	2	0	828932.7	352178.8	40
2823	line*coccidia	2	1	1524882	352178.8	40
2823	line*coccidia	2	2	852854.2	352178.8	40
2823	line*coccidia	2	3	859335.3	352178.8	40
2824	line	1		1280903	169758.5	40
2824	line	2		1263292	169758.5	40

2824	coccidia		0	1574627	240074.8	40
2824	coccidia		1	1667596	240074.8	40
2824	coccidia		2	723064.5	240074.8	40
2824	coccidia		3	1123103	240074.8	40
2824	line*coccidia	1	0	1709685	339517	40
2824	line*coccidia	1	1	1668407	339517	40
2824	line*coccidia	1	2	361825.8	339517	40
2824	line*coccidia	1	3	1383696	339517	40
2824	line*coccidia	2	0	1439569	339517	40
2824	line*coccidia	2	1	1666786	339517	40
2824	line*coccidia	2	2	1084303	339517	40
2824	line*coccidia	2	3	862509.8	339517	40
2825	line	1		548052.3	121898.9	40
2825	line	2		679970.5	121898.9	40
2825	coccidia		0	665428.2	172391.1	40
2825	coccidia		1	457811.7	172391.1	40
2825	coccidia		2	744182.7	172391.1	40
2825	coccidia		3	588622.9	172391.1	40
2825	line*coccidia	1	0	596323.2	243797.8	40
2825	line*coccidia	1	1	354372.7	243797.8	40
2825	line*coccidia	1	2	519321.5	243797.8	40
2825	line*coccidia	1	3	722191.7	243797.8	40
2825	line*coccidia	2	0	734533.2	243797.8	40
2825	line*coccidia	2	1	561250.7	243797.8	40
2825	line*coccidia	2	2	969043.8	243797.8	40
2825	line*coccidia	2	3	455054.2	243797.8	40
2826	line	1		771388.7	196257	40
2826	line	2		799249.8	196257	40
2826	coccidia		0	655900.5	277549.4	40
2826	coccidia		1	1290836	277549.4	40
2826	coccidia		2	645495.5	277549.4	40
2826	coccidia		3	549044.8	277549.4	40
2826	line*coccidia	1	0	694379	392514.1	40
2826	line*coccidia	1	1	810826	392514.1	40
2826	line*coccidia	1	2	829927	392514.1	40
2826	line*coccidia	1	3	750422.7	392514.1	40
2826	line*coccidia	2	0	617422	392514.1	40
2826	line*coccidia	2	1	1770847	392514.1	40
2826	line*coccidia	2	2	461064	392514.1	40
2826	line*coccidia	2	3	347666.8	392514.1	40
2909	line	1		11590730	1799445	40
2909	line	2		11515236	1799445	40
2909	coccidia		0	14420108	2544800	40
2909	coccidia		1	16282997	2544800	40
2909	coccidia		2	6857909	2544800	40
2909	coccidia		3	8650918	2544800	40
2909	line*coccidia	1	0	15417925	3598890	40
2909	line*coccidia	1	1	18796230	3598890	40
2909	line*coccidia	1	2	2202860	3598890	40

2909	line*coccidia	1	3	9945903	3598890	40
2909	line*coccidia	2	0	13422292	3598890	40
2909	line*coccidia	2	1	13769765	3598890	40
2909	line*coccidia	2	2	11512957	3598890	40
2909	line*coccidia	2	3	7355932	3598890	40
3001	line	1		102605.8	9797.614	40
3001	line	2		124313.6	9797.614	40
3001	coccidia		0	82178.25	13855.92	40
3001	coccidia		1	150242.6	13855.92	40
3001	coccidia		2	134997.5	13855.92	40
3001	coccidia		3	86420.33	13855.92	40
3001	line*coccidia	1	0	76576	19595.23	40
3001	line*coccidia	1	1	111475.3	19595.23	40
3001	line*coccidia	1	2	115163	19595.23	40
3001	line*coccidia	1	3	107208.7	19595.23	40
3001	line*coccidia	2	0	87780.5	19595.23	40
3001	line*coccidia	2	1	189009.8	19595.23	40
3001	line*coccidia	2	2	154832	19595.23	40
3001	line*coccidia	2	3	65632	19595.23	40
3003	line	1		38202.17	9576.613	40
3003	line	2		12225.33	9576.613	40
3003	coccidia		0	12569.58	13543.38	40
3003	coccidia		1	55305.67	13543.38	40
3003	coccidia		2	22853.92	13543.38	40
3003	coccidia		3	10125.83	13543.38	40
3003	line*coccidia	1	0	9608.5	19153.23	40
3003	line*coccidia	1	1	95128.67	19153.23	40
3003	line*coccidia	1	2	27819.83	19153.23	40
3003	line*coccidia	1	3	20251.67	19153.23	40
3003	line*coccidia	2	0	15530.67	19153.23	40
3003	line*coccidia	2	1	15482.67	19153.23	40
3003	line*coccidia	2	2	17888	19153.23	40
3003	line*coccidia	2	3	6.37E-12	19153.23	40
3004	line	1		276636	340474.5	40
3004	line	2		1073196	340474.5	40
3004	coccidia		0	549283.8	481503.6	40
3004	coccidia		1	1422118	481503.6	40
3004	coccidia		2	211825.4	481503.6	40
3004	coccidia		3	516436.3	481503.6	40
3004	line*coccidia	1	0	229698.7	680948.9	40
3004	line*coccidia	1	1	366921.8	680948.9	40
3004	line*coccidia	1	2	136168.3	680948.9	40
3004	line*coccidia	1	3	373755	680948.9	40
3004	line*coccidia	2	0	868868.8	680948.9	40
3004	line*coccidia	2	1	2477313	680948.9	40
3004	line*coccidia	2	2	287482.5	680948.9	40
3004	line*coccidia	2	3	659117.7	680948.9	40
3007	line	1		363763.6	397031.3	40
3007	line	2		657937.1	397031.3	40

3007	coccidia		0	135191.6	561487	40
3007	coccidia		1	1390118	561487	40
3007	coccidia		2	341413.5	561487	40
3007	coccidia		3	176678.8	561487	40
3007	line*coccidia	1	0	211404.3	794062.5	40
3007	line*coccidia	1	1	400835	794062.5	40
3007	line*coccidia	1	2	621954	794062.5	40
3007	line*coccidia	1	3	220861	794062.5	40
3007	line*coccidia	2	0	58978.83	794062.5	40
3007	line*coccidia	2	1	2379400	794062.5	40
3007	line*coccidia	2	2	60873	794062.5	40
3007	line*coccidia	2	3	132496.7	794062.5	40
3008	line	1		128462.6	12274.58	40
3008	line	2		181815.6	12274.58	40
3008	coccidia		0	162418.3	17358.88	40
3008	coccidia		1	166636.1	17358.88	40
3008	coccidia		2	146236	17358.88	40
3008	coccidia		3	145266.1	17358.88	40
3008	line*coccidia	1	0	125794.7	24549.16	40
3008	line*coccidia	1	1	124853.3	24549.16	40
3008	line*coccidia	1	2	103896	24549.16	40
3008	line*coccidia	1	3	159306.3	24549.16	40
3008	line*coccidia	2	0	199041.8	24549.16	40
3008	line*coccidia	2	1	208418.8	24549.16	40
3008	line*coccidia	2	2	188576	24549.16	40
3008	line*coccidia	2	3	131225.8	24549.16	40
3010	line	1		27950.67	3936.166	40
3010	line	2		21069.21	3936.166	40
3010	coccidia		0	26246.33	5566.58	40
3010	coccidia		1	26642.33	5566.58	40
3010	coccidia		2	24387.42	5566.58	40
3010	coccidia		3	20763.67	5566.58	40
3010	line*coccidia	1	0	21383.17	7872.333	40
3010	line*coccidia	1	1	20913	7872.333	40
3010	line*coccidia	1	2	27979.17	7872.333	40
3010	line*coccidia	1	3	41527.33	7872.333	40
3010	line*coccidia	2	0	31109.5	7872.333	40
3010	line*coccidia	2	1	32371.67	7872.333	40
3010	line*coccidia	2	2	20795.67	7872.333	40
3010	line*coccidia	2	3	0	7872.333	40
3011	line	1		255978.3	25761.07	40
3011	line	2		443677.1	25761.07	40
3011	coccidia		0	325516.9	36431.65	40
3011	coccidia		1	399738	36431.65	40
3011	coccidia		2	354368.6	36431.65	40
3011	coccidia		3	319687.4	36431.65	40
3011	line*coccidia	1	0	312454.7	51522.13	40
3011	line*coccidia	1	1	239271.7	51522.13	40
3011	line*coccidia	1	2	160466.3	51522.13	40

3011	line*coccidia	1	3	311720.7	51522.13	40
3011	line*coccidia	2	0	338579.2	51522.13	40
3011	line*coccidia	2	1	560204.3	51522.13	40
3011	line*coccidia	2	2	548270.8	51522.13	40
3011	line*coccidia	2	3	327654.2	51522.13	40
3013	line	1		185242.8	23705.56	40
3013	line	2		170672.5	23705.56	40
3013	coccidia		0	195437.2	33524.72	40
3013	coccidia		1	219590.5	33524.72	40
3013	coccidia		2	147113.8	33524.72	40
3013	coccidia		3	149688.9	33524.72	40
3013	line*coccidia	1	0	206087.7	47411.11	40
3013	line*coccidia	1	1	156007	47411.11	40
3013	line*coccidia	1	2	153390	47411.11	40
3013	line*coccidia	1	3	225486.3	47411.11	40
3013	line*coccidia	2	0	184786.7	47411.11	40
3013	line*coccidia	2	1	283174	47411.11	40
3013	line*coccidia	2	2	140837.7	47411.11	40
3013	line*coccidia	2	3	73891.5	47411.11	40
3014	line	1		593945.5	44143.2	40
3014	line	2		588072	44143.2	40
3014	coccidia		0	582623.4	62427.91	40
3014	coccidia		1	772335.8	62427.91	40
3014	coccidia		2	495646.2	62427.91	40
3014	coccidia		3	513429.6	62427.91	40
3014	line*coccidia	1	0	615268.5	88286.39	40
3014	line*coccidia	1	1	763312.2	88286.39	40
3014	line*coccidia	1	2	405080.3	88286.39	40
3014	line*coccidia	1	3	592120.8	88286.39	40
3014	line*coccidia	2	0	549978.3	88286.39	40
3014	line*coccidia	2	1	781359.3	88286.39	40
3014	line*coccidia	2	2	586212	88286.39	40
3014	line*coccidia	2	3	434738.3	88286.39	40
3017	line	1		120112	33049.91	40
3017	line	2		174650.5	33049.91	40
3017	coccidia		0	122641.1	46739.63	40
3017	coccidia		1	181628.3	46739.63	40
3017	coccidia		2	163519.8	46739.63	40
3017	coccidia		3	121735.9	46739.63	40
3017	line*coccidia	1	0	129529.7	66099.81	40
3017	line*coccidia	1	1	54231.83	66099.81	40
3017	line*coccidia	1	2	166666.3	66099.81	40
3017	line*coccidia	1	3	130020.3	66099.81	40
3017	line*coccidia	2	0	115752.5	66099.81	40
3017	line*coccidia	2	1	309024.7	66099.81	40
3017	line*coccidia	2	2	160373.2	66099.81	40
3017	line*coccidia	2	3	113451.5	66099.81	40
3018	line	1		86771.83	9275.137	40
3018	line	2		119022.4	9275.137	40

3018	coccidia		0	126206.2	13117.03	40
3018	coccidia		1	68462.17	13117.03	40
3018	coccidia		2	115044.4	13117.03	40
3018	coccidia		3	101875.7	13117.03	40
3018	line*coccidia	1	0	107443.5	18550.27	40
3018	line*coccidia	1	1	46116.83	18550.27	40
3018	line*coccidia	1	2	89913.67	18550.27	40
3018	line*coccidia	1	3	103613.3	18550.27	40
3018	line*coccidia	2	0	144968.8	18550.27	40
3018	line*coccidia	2	1	90807.5	18550.27	40
3018	line*coccidia	2	2	140175.2	18550.27	40
3018	line*coccidia	2	3	100138	18550.27	40
3019	line	1		1357243	136089	40
3019	line	2		941883.8	136089	40
3019	coccidia		0	1126102	192459	40
3019	coccidia		1	1269443	192459	40
3019	coccidia		2	876987.8	192459	40
3019	coccidia		3	1325720	192459	40
3019	line*coccidia	1	0	1450155	272178.1	40
3019	line*coccidia	1	1	1394054	272178.1	40
3019	line*coccidia	1	2	869539.3	272178.1	40
3019	line*coccidia	1	3	1715223	272178.1	40
3019	line*coccidia	2	0	802048.7	272178.1	40
3019	line*coccidia	2	1	1144832	272178.1	40
3019	line*coccidia	2	2	884436.3	272178.1	40
3019	line*coccidia	2	3	936217.8	272178.1	40
3020	line	1		19522.79	9049.359	40
3020	line	2		74232.46	9049.359	40
3020	coccidia		0	29560.08	12797.73	40
3020	coccidia		1	83863.58	12797.73	40
3020	coccidia		2	32529.58	12797.73	40
3020	coccidia		3	41557.25	12797.73	40
3020	line*coccidia	1	0	25707	18098.72	40
3020	line*coccidia	1	1	16239.5	18098.72	40
3020	line*coccidia	1	2	9037.833	18098.72	40
3020	line*coccidia	1	3	27106.83	18098.72	40
3020	line*coccidia	2	0	33413.17	18098.72	40
3020	line*coccidia	2	1	151487.7	18098.72	40
3020	line*coccidia	2	2	56021.33	18098.72	40
3020	line*coccidia	2	3	56007.67	18098.72	40
3021	line	1		3034454	556847.6	40
3021	line	2		2635399	556847.6	40
3021	coccidia		0	3027972	787501.4	40
3021	coccidia		1	2995026	787501.4	40
3021	coccidia		2	1621296	787501.4	40
3021	coccidia		3	3695414	787501.4	40
3021	line*coccidia	1	0	3290463	1113695	40
3021	line*coccidia	1	1	2663291	1113695	40
3021	line*coccidia	1	2	729956	1113695	40

3021	line*coccidia	1	3	5454108	1113695	40
3021	line*coccidia	2	0	2765481	1113695	40
3021	line*coccidia	2	1	3326762	1113695	40
3021	line*coccidia	2	2	2512635	1113695	40
3021	line*coccidia	2	3	1936720	1113695	40
3022	line	1		405730.9	33040.07	40
3022	line	2		272048.2	33040.07	40
3022	coccidia		0	376891.5	46725.71	40
3022	coccidia		1	206727.3	46725.71	40
3022	coccidia		2	400094.1	46725.71	40
3022	coccidia		3	371845.3	46725.71	40
3022	line*coccidia	1	0	532338.2	66080.13	40
3022	line*coccidia	1	1	211062.2	66080.13	40
3022	line*coccidia	1	2	381248.5	66080.13	40
3022	line*coccidia	1	3	498274.7	66080.13	40
3022	line*coccidia	2	0	221444.8	66080.13	40
3022	line*coccidia	2	1	202392.3	66080.13	40
3022	line*coccidia	2	2	418939.7	66080.13	40
3022	line*coccidia	2	3	245415.8	66080.13	40
3023	line	1		24824.5	4608.47	40
3023	line	2		38665.92	4608.47	40
3023	coccidia		0	29581.5	6517.36	40
3023	coccidia		1	19632.92	6517.36	40
3023	coccidia		2	40149.17	6517.36	40
3023	coccidia		3	37617.25	6517.36	40
3023	line*coccidia	1	0	24863.5	9216.939	40
3023	line*coccidia	1	1	16405.83	9216.939	40
3023	line*coccidia	1	2	31475.33	9216.939	40
3023	line*coccidia	1	3	26553.33	9216.939	40
3023	line*coccidia	2	0	34299.5	9216.939	40
3023	line*coccidia	2	1	22860	9216.939	40
3023	line*coccidia	2	2	48823	9216.939	40
3023	line*coccidia	2	3	48681.17	9216.939	40
3027	line	1		21240.25	4527.372	40
3027	line	2		-1.3E-13	4527.372	40
3027	coccidia		0	0	6402.671	40
3027	coccidia		1	42480.5	6402.671	40
3027	coccidia		2	0	6402.671	40
3027	coccidia		3	0	6402.671	40
3027	line*coccidia	1	0	0	9054.744	40
3027	line*coccidia	1	1	84961	9054.744	40
3027	line*coccidia	1	2	1.79E-13	9054.744	40
3027	line*coccidia	1	3	1.79E-13	9054.744	40
3027	line*coccidia	2	0	0	9054.744	40
3027	line*coccidia	2	1	-1.8E-13	9054.744	40
3027	line*coccidia	2	2	-1.8E-13	9054.744	40
3027	line*coccidia	2	3	-1.8E-13	9054.744	40
3028	line	1		6549.458	1330.645	40
3028	line	2		2.73E-13	1330.645	40

3028	coccidia		0	0	1881.817	40
3028	coccidia		1	13098.92	1881.817	40
3028	coccidia		2	9.09E-13	1881.817	40
3028	coccidia		3	9.09E-13	1881.817	40
3028	line*coccidia	1	0	0	2661.291	40
3028	line*coccidia	1	1	26197.83	2661.291	40
3028	line*coccidia	1	2	8.49E-13	2661.291	40
3028	line*coccidia	1	3	8.49E-13	2661.291	40
3028	line*coccidia	2	0	0	2661.291	40
3028	line*coccidia	2	1	-8.5E-13	2661.291	40
3028	line*coccidia	2	2	9.7E-13	2661.291	40
3028	line*coccidia	2	3	9.7E-13	2661.291	40
3029	line	1		204541.3	40965.92	40
3029	line	2		305239	40965.92	40
3029	coccidia		0	377117.8	57934.56	40
3029	coccidia		1	297707.3	57934.56	40
3029	coccidia		2	123319.4	57934.56	40
3029	coccidia		3	221416.1	57934.56	40
3029	line*coccidia	1	0	238651.2	81931.84	40
3029	line*coccidia	1	1	214983	81931.84	40
3029	line*coccidia	1	2	106036	81931.84	40
3029	line*coccidia	1	3	258495	81931.84	40
3029	line*coccidia	2	0	515584.5	81931.84	40
3029	line*coccidia	2	1	380431.5	81931.84	40
3029	line*coccidia	2	2	140602.8	81931.84	40
3029	line*coccidia	2	3	184337.2	81931.84	40
3101	line	1		139589.1	36953	40
3101	line	2		329124.3	36953	40
3101	coccidia		0	296486.9	52259.43	40
3101	coccidia		1	185132.3	52259.43	40
3101	coccidia		2	209280.3	52259.43	40
3101	coccidia		3	246527.3	52259.43	40
3101	line*coccidia	1	0	89654	73905.99	40
3101	line*coccidia	1	1	46850.33	73905.99	40
3101	line*coccidia	1	2	107806	73905.99	40
3101	line*coccidia	1	3	314046.2	73905.99	40
3101	line*coccidia	2	0	503319.8	73905.99	40
3101	line*coccidia	2	1	323414.2	73905.99	40
3101	line*coccidia	2	2	310754.7	73905.99	40
3101	line*coccidia	2	3	179008.5	73905.99	40
3102	line	1		19479591	2228729	40
3102	line	2		19754215	2228729	40
3102	coccidia		0	25827313	3151899	40
3102	coccidia		1	12464914	3151899	40
3102	coccidia		2	15748983	3151899	40
3102	coccidia		3	24426402	3151899	40
3102	line*coccidia	1	0	26977060	4457458	40
3102	line*coccidia	1	1	6868370	4457458	40
3102	line*coccidia	1	2	10925988	4457458	40

3102	line*coccidia	1	3	33146947	4457458	40
3102	line*coccidia	2	0	24677567	4457458	40
3102	line*coccidia	2	1	18061458	4457458	40
3102	line*coccidia	2	2	20571979	4457458	40
3102	line*coccidia	2	3	15705857	4457458	40
3103	line	1		150208.8	20683.39	40
3103	line	2		118138.1	20683.39	40
3103	coccidia		0	182886.3	29250.73	40
3103	coccidia		1	128159.9	29250.73	40
3103	coccidia		2	101070.6	29250.73	40
3103	coccidia		3	124576.9	29250.73	40
3103	line*coccidia	1	0	182889.3	41366.78	40
3103	line*coccidia	1	1	125542.5	41366.78	40
3103	line*coccidia	1	2	73441.67	41366.78	40
3103	line*coccidia	1	3	218961.5	41366.78	40
3103	line*coccidia	2	0	182883.3	41366.78	40
3103	line*coccidia	2	1	130777.3	41366.78	40
3103	line*coccidia	2	2	128699.5	41366.78	40
3103	line*coccidia	2	3	30192.33	41366.78	40
3104	line	1		1262825	392582.4	40
3104	line	2		770377.3	392582.4	40
3104	coccidia		0	1054298	555195.4	40
3104	coccidia		1	816221.5	555195.4	40
3104	coccidia		2	398211.9	555195.4	40
3104	coccidia		3	1797673	555195.4	40
3104	line*coccidia	1	0	1055451	785164.9	40
3104	line*coccidia	1	1	503786.7	785164.9	40
3104	line*coccidia	1	2	318629.8	785164.9	40
3104	line*coccidia	1	3	3173431	785164.9	40
3104	line*coccidia	2	0	1053144	785164.9	40
3104	line*coccidia	2	1	1128656	785164.9	40
3104	line*coccidia	2	2	477794	785164.9	40
3104	line*coccidia	2	3	421914.8	785164.9	40
3105	line	1		430556.8	60162.73	40
3105	line	2		389372.5	60162.73	40
3105	coccidia		0	502922.2	85082.95	40
3105	coccidia		1	481777.4	85082.95	40
3105	coccidia		2	257023.5	85082.95	40
3105	coccidia		3	398135.4	85082.95	40
3105	line*coccidia	1	0	725748.7	120325.5	40
3105	line*coccidia	1	1	220226.8	120325.5	40
3105	line*coccidia	1	2	224621	120325.5	40
3105	line*coccidia	1	3	551630.7	120325.5	40
3105	line*coccidia	2	0	280095.7	120325.5	40
3105	line*coccidia	2	1	743328	120325.5	40
3105	line*coccidia	2	2	289426	120325.5	40
3105	line*coccidia	2	3	244640.2	120325.5	40
3107	line	1		938669.4	87214.33	40
3107	line	2		832019.3	87214.33	40

3107	coccidia		0	1015838	123339.7	40
3107	coccidia		1	756674.3	123339.7	40
3107	coccidia		2	1065347	123339.7	40
3107	coccidia		3	703518.7	123339.7	40
3107	line*coccidia	1	0	1001946	174428.7	40
3107	line*coccidia	1	1	504896.2	174428.7	40
3107	line*coccidia	1	2	1221219	174428.7	40
3107	line*coccidia	1	3	1026617	174428.7	40
3107	line*coccidia	2	0	1029730	174428.7	40
3107	line*coccidia	2	1	1008452	174428.7	40
3107	line*coccidia	2	2	909474.5	174428.7	40
3107	line*coccidia	2	3	380420.2	174428.7	40
3108	line	1		87903509	8271793	40
3108	line	2		1.12E+08	8271793	40
3108	coccidia		0	95496162	11698081	40
3108	coccidia		1	64979959	11698081	40
3108	coccidia		2	1.24E+08	11698081	40
3108	coccidia		3	1.15E+08	11698081	40
3108	line*coccidia	1	0	63971704	16543585	40
3108	line*coccidia	1	1	58410383	16543585	40
3108	line*coccidia	1	2	1.12E+08	16543585	40
3108	line*coccidia	1	3	1.17E+08	16543585	40
3108	line*coccidia	2	0	1.27E+08	16543585	40
3108	line*coccidia	2	1	71549535	16543585	40
3108	line*coccidia	2	2	1.36E+08	16543585	40
3108	line*coccidia	2	3	1.13E+08	16543585	40
3111	line	1		50799.83	6281.094	40
3111	line	2		76497.46	6281.094	40
3111	coccidia		0	71684.58	8882.809	40
3111	coccidia		1	80987.42	8882.809	40
3111	coccidia		2	48939.33	8882.809	40
3111	coccidia		3	52983.25	8882.809	40
3111	line*coccidia	1	0	48731	12562.19	40
3111	line*coccidia	1	1	47962	12562.19	40
3111	line*coccidia	1	2	45069.83	12562.19	40
3111	line*coccidia	1	3	61436.5	12562.19	40
3111	line*coccidia	2	0	94638.17	12562.19	40
3111	line*coccidia	2	1	114012.8	12562.19	40
3111	line*coccidia	2	2	52808.83	12562.19	40
3111	line*coccidia	2	3	44530	12562.19	40
3113	line	1		104624.5	25411.65	40
3113	line	2		208678.7	25411.65	40
3113	coccidia		0	166930.5	35937.51	40
3113	coccidia		1	189618.5	35937.51	40
3113	coccidia		2	129569	35937.51	40
3113	coccidia		3	140488.3	35937.51	40
3113	line*coccidia	1	0	174870.3	50823.31	40
3113	line*coccidia	1	1	85416.67	50823.31	40
3113	line*coccidia	1	2	63279	50823.31	40

3113	line*coccidia	1	3	94932	50823.31	40
3113	line*coccidia	2	0	158990.7	50823.31	40
3113	line*coccidia	2	1	293820.3	50823.31	40
3113	line*coccidia	2	2	195859	50823.31	40
3113	line*coccidia	2	3	186044.7	50823.31	40
3114	line	1		18246.17	8517.204	40
3114	line	2		47373.42	8517.204	40
3114	coccidia		0	20583.83	12045.15	40
3114	coccidia		1	28250.75	12045.15	40
3114	coccidia		2	28873.92	12045.15	40
3114	coccidia		3	53530.67	12045.15	40
3114	line*coccidia	1	0	23363.17	17034.41	40
3114	line*coccidia	1	1	-1.1E-11	17034.41	40
3114	line*coccidia	1	2	36122.17	17034.41	40
3114	line*coccidia	1	3	13499.33	17034.41	40
3114	line*coccidia	2	0	17804.5	17034.41	40
3114	line*coccidia	2	1	56501.5	17034.41	40
3114	line*coccidia	2	2	21625.67	17034.41	40
3114	line*coccidia	2	3	93562	17034.41	40
3115	line	1		238539.7	122521.7	40
3115	line	2		96302.17	122521.7	40
3115	coccidia		0	77411.33	173271.9	40
3115	coccidia		1	112168.4	173271.9	40
3115	coccidia		2	72139.92	173271.9	40
3115	coccidia		3	407964.1	173271.9	40
3115	line*coccidia	1	0	78666.17	245043.5	40
3115	line*coccidia	1	1	55645.33	245043.5	40
3115	line*coccidia	1	2	39692.5	245043.5	40
3115	line*coccidia	1	3	780154.8	245043.5	40
3115	line*coccidia	2	0	76156.5	245043.5	40
3115	line*coccidia	2	1	168691.5	245043.5	40
3115	line*coccidia	2	2	104587.3	245043.5	40
3115	line*coccidia	2	3	35773.33	245043.5	40
3116	line	1		0	20012.22	40
3116	line	2		236100.8	20012.22	40
3116	coccidia		0	0	28301.55	40
3116	coccidia		1	199444	28301.55	40
3116	coccidia		2	272757.5	28301.55	40
3116	coccidia		3	0	28301.55	40
3116	line*coccidia	1	0	0	40024.43	40
3116	line*coccidia	1	1	0	40024.43	40
3116	line*coccidia	1	2	0	40024.43	40
3116	line*coccidia	1	3	0	40024.43	40
3116	line*coccidia	2	0	0	40024.43	40
3116	line*coccidia	2	1	398888	40024.43	40
3116	line*coccidia	2	2	545515	40024.43	40
3116	line*coccidia	2	3	0	40024.43	40
3117	line	1		14596507	1944454	40
3117	line	2		7649970	1944454	40

3117	coccidia		0	14152372	2749873	40
3117	coccidia		1	11325137	2749873	40
3117	coccidia		2	5723837	2749873	40
3117	coccidia		3	13291608	2749873	40
3117	line*coccidia	1	0	21076819	3888907	40
3117	line*coccidia	1	1	13159176	3888907	40
3117	line*coccidia	1	2	3091889	3888907	40
3117	line*coccidia	1	3	21058143	3888907	40
3117	line*coccidia	2	0	7227925	3888907	40
3117	line*coccidia	2	1	9491098	3888907	40
3117	line*coccidia	2	2	8355786	3888907	40
3117	line*coccidia	2	3	5525072	3888907	40
3118	line	1		91840.75	18186.56	40
3118	line	2		137773	18186.56	40
3118	coccidia		0	155433.8	25719.69	40
3118	coccidia		1	114588.8	25719.69	40
3118	coccidia		2	80959.75	25719.69	40
3118	coccidia		3	108245.2	25719.69	40
3118	line*coccidia	1	0	195695.2	36373.13	40
3118	line*coccidia	1	1	15531.67	36373.13	40
3118	line*coccidia	1	2	38462.83	36373.13	40
3118	line*coccidia	1	3	117673.3	36373.13	40
3118	line*coccidia	2	0	115172.3	36373.13	40
3118	line*coccidia	2	1	213645.8	36373.13	40
3118	line*coccidia	2	2	123456.7	36373.13	40
3118	line*coccidia	2	3	98817	36373.13	40
3119	line	1		41894.67	8293.453	40
3119	line	2		41306	8293.453	40
3119	coccidia		0	63278.67	11728.71	40
3119	coccidia		1	29103.67	11728.71	40
3119	coccidia		2	44657.5	11728.71	40
3119	coccidia		3	29361.5	11728.71	40
3119	line*coccidia	1	0	57161.83	16586.91	40
3119	line*coccidia	1	1	28295.83	16586.91	40
3119	line*coccidia	1	2	54850.17	16586.91	40
3119	line*coccidia	1	3	27270.83	16586.91	40
3119	line*coccidia	2	0	69395.5	16586.91	40
3119	line*coccidia	2	1	29911.5	16586.91	40
3119	line*coccidia	2	2	34464.83	16586.91	40
3119	line*coccidia	2	3	31452.17	16586.91	40
3121	line	1		338666.5	28293.25	40
3121	line	2		411269.9	28293.25	40
3121	coccidia		0	403498.8	40012.7	40
3121	coccidia		1	401585.9	40012.7	40
3121	coccidia		2	293506.6	40012.7	40
3121	coccidia		3	401281.4	40012.7	40
3121	line*coccidia	1	0	425441.3	56586.51	40
3121	line*coccidia	1	1	252863.8	56586.51	40
3121	line*coccidia	1	2	174570.3	56586.51	40

3121	line*coccidia	1	3	501790.3	56586.51	40
3121	line*coccidia	2	0	381556.3	56586.51	40
3121	line*coccidia	2	1	550308	56586.51	40
3121	line*coccidia	2	2	412442.8	56586.51	40
3121	line*coccidia	2	3	300772.5	56586.51	40
3124	line	1		55505.17	142902.1	40
3124	line	2		263752.3	142902.1	40
3124	coccidia		0	39818.17	202094	40
3124	coccidia		1	527016.3	202094	40
3124	coccidia		2	25191.42	202094	40
3124	coccidia		3	46488.92	202094	40
3124	line*coccidia	1	0	23925.83	285804.1	40
3124	line*coccidia	1	1	198094.8	285804.1	40
3124	line*coccidia	1	2	5.82E-11	285804.1	40
3124	line*coccidia	1	3	1.46E-11	285804.1	40
3124	line*coccidia	2	0	55710.5	285804.1	40
3124	line*coccidia	2	1	855937.8	285804.1	40
3124	line*coccidia	2	2	50382.83	285804.1	40
3124	line*coccidia	2	3	92977.83	285804.1	40
3201	line	1		912007.3	58272.47	40
3201	line	2		1120631	58272.47	40
3201	coccidia		0	1505655	82409.72	40
3201	coccidia		1	1099084	82409.72	40
3201	coccidia		2	655468.8	82409.72	40
3201	coccidia		3	805069.3	82409.72	40
3201	line*coccidia	1	0	1538880	116544.9	40
3201	line*coccidia	1	1	568634.8	116544.9	40
3201	line*coccidia	1	2	447734.2	116544.9	40
3201	line*coccidia	1	3	1092781	116544.9	40
3201	line*coccidia	2	0	1472430	116544.9	40
3201	line*coccidia	2	1	1629534	116544.9	40
3201	line*coccidia	2	2	863203.3	116544.9	40
3201	line*coccidia	2	3	517358	116544.9	40
3202	line	1		54358.92	9531.138	40
3202	line	2		63756.75	9531.138	40
3202	coccidia		0	0	13479.07	40
3202	coccidia		1	135943.8	13479.07	40
3202	coccidia		2	42009.17	13479.07	40
3202	coccidia		3	58278.42	13479.07	40
3202	line*coccidia	1	0	0	19062.28	40
3202	line*coccidia	1	1	100878.8	19062.28	40
3202	line*coccidia	1	2	0	19062.28	40
3202	line*coccidia	1	3	116556.8	19062.28	40
3202	line*coccidia	2	0	0	19062.28	40
3202	line*coccidia	2	1	171008.7	19062.28	40
3202	line*coccidia	2	2	84018.33	19062.28	40
3202	line*coccidia	2	3	2.91E-11	19062.28	40
3203	line	1		125165.8	16770.01	40
3203	line	2		97381.58	16770.01	40

3203	coccidia		0	139374.6	23716.37	40
3203	coccidia		1	124123.1	23716.37	40
3203	coccidia		2	95406	23716.37	40
3203	coccidia		3	86191	23716.37	40
3203	line*coccidia	1	0	143239.7	33540.02	40
3203	line*coccidia	1	1	161894.3	33540.02	40
3203	line*coccidia	1	2	78618	33540.02	40
3203	line*coccidia	1	3	116911	33540.02	40
3203	line*coccidia	2	0	135509.5	33540.02	40
3203	line*coccidia	2	1	86351.83	33540.02	40
3203	line*coccidia	2	2	112194	33540.02	40
3203	line*coccidia	2	3	55471	33540.02	40
3204	line	1		93454.71	11020.97	40
3204	line	2		104392.9	11020.97	40
3204	coccidia		0	153078.3	15586.01	40
3204	coccidia		1	88515.25	15586.01	40
3204	coccidia		2	54631.08	15586.01	40
3204	coccidia		3	99470.67	15586.01	40
3204	line*coccidia	1	0	141516	22041.95	40
3204	line*coccidia	1	1	82200.67	22041.95	40
3204	line*coccidia	1	2	35717.33	22041.95	40
3204	line*coccidia	1	3	114384.8	22041.95	40
3204	line*coccidia	2	0	164640.5	22041.95	40
3204	line*coccidia	2	1	94829.83	22041.95	40
3204	line*coccidia	2	2	73544.83	22041.95	40
3204	line*coccidia	2	3	84556.5	22041.95	40
3205	line	1		300993.6	23863.74	40
3205	line	2		268803.8	23863.74	40
3205	coccidia		0	343037.2	33748.42	40
3205	coccidia		1	315501.5	33748.42	40
3205	coccidia		2	169641.6	33748.42	40
3205	coccidia		3	311414.6	33748.42	40
3205	line*coccidia	1	0	309312.3	47727.48	40
3205	line*coccidia	1	1	297099.8	47727.48	40
3205	line*coccidia	1	2	105737.8	47727.48	40
3205	line*coccidia	1	3	491824.3	47727.48	40
3205	line*coccidia	2	0	376762	47727.48	40
3205	line*coccidia	2	1	333903.2	47727.48	40
3205	line*coccidia	2	2	233545.3	47727.48	40
3205	line*coccidia	2	3	131004.8	47727.48	40
3207	line	1		151998.6	29255.88	40
3207	line	2		161249.7	29255.88	40
3207	coccidia		0	160208.5	41374.06	40
3207	coccidia		1	236385.8	41374.06	40
3207	coccidia		2	149105.9	41374.06	40
3207	coccidia		3	80796.42	41374.06	40
3207	line*coccidia	1	0	249167.2	58511.76	40
3207	line*coccidia	1	1	172381.7	58511.76	40
3207	line*coccidia	1	2	68927.33	58511.76	40

3207	line*coccidia	1	3	117518.2	58511.76	40
3207	line*coccidia	2	0	71249.83	58511.76	40
3207	line*coccidia	2	1	300389.8	58511.76	40
3207	line*coccidia	2	2	229284.5	58511.76	40
3207	line*coccidia	2	3	44074.67	58511.76	40
3208	line	1		200213.2	26376.86	40
3208	line	2		198833.3	26376.86	40
3208	coccidia		0	208995.8	37302.52	40
3208	coccidia		1	301092.2	37302.52	40
3208	coccidia		2	145607.7	37302.52	40
3208	coccidia		3	142397.2	37302.52	40
3208	line*coccidia	1	0	207746	52753.72	40
3208	line*coccidia	1	1	289290.5	52753.72	40
3208	line*coccidia	1	2	107781.8	52753.72	40
3208	line*coccidia	1	3	196034.3	52753.72	40
3208	line*coccidia	2	0	210245.7	52753.72	40
3208	line*coccidia	2	1	312893.8	52753.72	40
3208	line*coccidia	2	2	183433.5	52753.72	40
3208	line*coccidia	2	3	88760	52753.72	40
3210	line	1		957501.4	111366.5	40
3210	line	2		645737.3	111366.5	40
3210	coccidia		0	702750.8	157496	40
3210	coccidia		1	1346423	157496	40
3210	coccidia		2	529353.3	157496	40
3210	coccidia		3	627950.3	157496	40
3210	line*coccidia	1	0	789932.8	222732.9	40
3210	line*coccidia	1	1	1827430	222732.9	40
3210	line*coccidia	1	2	393841.5	222732.9	40
3210	line*coccidia	1	3	818801	222732.9	40
3210	line*coccidia	2	0	615568.7	222732.9	40
3210	line*coccidia	2	1	865416	222732.9	40
3210	line*coccidia	2	2	664865.2	222732.9	40
3210	line*coccidia	2	3	437099.5	222732.9	40
3211	line	1		358025	29711.16	40
3211	line	2		340355.6	29711.16	40
3211	coccidia		0	388670.3	42017.92	40
3211	coccidia		1	421337.1	42017.92	40
3211	coccidia		2	247068.1	42017.92	40
3211	coccidia		3	339685.8	42017.92	40
3211	line*coccidia	1	0	416218	59422.31	40
3211	line*coccidia	1	1	401982.8	59422.31	40
3211	line*coccidia	1	2	128918	59422.31	40
3211	line*coccidia	1	3	484981.2	59422.31	40
3211	line*coccidia	2	0	361122.5	59422.31	40
3211	line*coccidia	2	1	440691.3	59422.31	40
3211	line*coccidia	2	2	365218.2	59422.31	40
3211	line*coccidia	2	3	194390.5	59422.31	40
3212	line	1		1446870	107934.4	40
3212	line	2		1546845	107934.4	40

3212	coccidia		0	1803322	152642.3	40
3212	coccidia		1	1495311	152642.3	40
3212	coccidia		2	1060897	152642.3	40
3212	coccidia		3	1627899	152642.3	40
3212	line*coccidia	1	0	1607174	215868.8	40
3212	line*coccidia	1	1	1007812	215868.8	40
3212	line*coccidia	1	2	802470.5	215868.8	40
3212	line*coccidia	1	3	2370024	215868.8	40
3212	line*coccidia	2	0	1999471	215868.8	40
3212	line*coccidia	2	1	1982811	215868.8	40
3212	line*coccidia	2	2	1319323	215868.8	40
3212	line*coccidia	2	3	885774.7	215868.8	40
3213	line	1		296563.6	31190.8	40
3213	line	2		283595.3	31190.8	40
3213	coccidia		0	371320.3	44110.46	40
3213	coccidia		1	303785.3	44110.46	40
3213	coccidia		2	203581.7	44110.46	40
3213	coccidia		3	281630.5	44110.46	40
3213	line*coccidia	1	0	377314.7	62381.61	40
3213	line*coccidia	1	1	334506.5	62381.61	40
3213	line*coccidia	1	2	77688.17	62381.61	40
3213	line*coccidia	1	3	396745	62381.61	40
3213	line*coccidia	2	0	365325.8	62381.61	40
3213	line*coccidia	2	1	273064.2	62381.61	40
3213	line*coccidia	2	2	329475.2	62381.61	40
3213	line*coccidia	2	3	166516	62381.61	40
3214	line	1		865420.7	113625.2	40
3214	line	2		590622.6	113625.2	40
3214	coccidia		0	610911.8	160690.3	40
3214	coccidia		1	949368.7	160690.3	40
3214	coccidia		2	571539.7	160690.3	40
3214	coccidia		3	780266.5	160690.3	40
3214	line*coccidia	1	0	654078.2	227250.4	40
3214	line*coccidia	1	1	1253772	227250.4	40
3214	line*coccidia	1	2	522505.7	227250.4	40
3214	line*coccidia	1	3	1031327	227250.4	40
3214	line*coccidia	2	0	567745.3	227250.4	40
3214	line*coccidia	2	1	644965.3	227250.4	40
3214	line*coccidia	2	2	620573.7	227250.4	40
3214	line*coccidia	2	3	529206.2	227250.4	40
3215	line	1		1677177	158599.3	40
3215	line	2		1428107	158599.3	40
3215	coccidia		0	1796753	224293.2	40
3215	coccidia		1	1949144	224293.2	40
3215	coccidia		2	927453.1	224293.2	40
3215	coccidia		3	1537218	224293.2	40
3215	line*coccidia	1	0	2126245	317198.5	40
3215	line*coccidia	1	1	2117265	317198.5	40
3215	line*coccidia	1	2	516576.8	317198.5	40

3215	line*coccidia	1	3	1948620	317198.5	40
3215	line*coccidia	2	0	1467261	317198.5	40
3215	line*coccidia	2	1	1781022	317198.5	40
3215	line*coccidia	2	2	1338329	317198.5	40
3215	line*coccidia	2	3	1125817	317198.5	40
3216	line	1		538895.8	50614.97	40
3216	line	2		703290.2	50614.97	40
3216	coccidia		0	824102.6	71580.37	40
3216	coccidia		1	518721.3	71580.37	40
3216	coccidia		2	575230.5	71580.37	40
3216	coccidia		3	566317.8	71580.37	40
3216	line*coccidia	1	0	746798.7	101229.9	40
3216	line*coccidia	1	1	444180.8	101229.9	40
3216	line*coccidia	1	2	278894.8	101229.9	40
3216	line*coccidia	1	3	685709	101229.9	40
3216	line*coccidia	2	0	901406.5	101229.9	40
3216	line*coccidia	2	1	593261.7	101229.9	40
3216	line*coccidia	2	2	871566.2	101229.9	40
3216	line*coccidia	2	3	446926.5	101229.9	40
3218	line	1		493540.2	56068.41	40
3218	line	2		400609.7	56068.41	40
3218	coccidia		0	479010.7	79292.7	40
3218	coccidia		1	565314.4	79292.7	40
3218	coccidia		2	313678.3	79292.7	40
3218	coccidia		3	430296.3	79292.7	40
3218	line*coccidia	1	0	474679.2	112136.8	40
3218	line*coccidia	1	1	669848.5	112136.8	40
3218	line*coccidia	1	2	228862.5	112136.8	40
3218	line*coccidia	1	3	600770.5	112136.8	40
3218	line*coccidia	2	0	483342.2	112136.8	40
3218	line*coccidia	2	1	460780.3	112136.8	40
3218	line*coccidia	2	2	398494.2	112136.8	40
3218	line*coccidia	2	3	259822	112136.8	40
3221	line	1		66393.17	17855.77	40
3221	line	2		95114.46	17855.77	40
3221	coccidia		0	68886.83	25251.87	40
3221	coccidia		1	76489.42	25251.87	40
3221	coccidia		2	57536.83	25251.87	40
3221	coccidia		3	120102.2	25251.87	40
3221	line*coccidia	1	0	71846.33	35711.53	40
3221	line*coccidia	1	1	67285.33	35711.53	40
3221	line*coccidia	1	2	57453.33	35711.53	40
3221	line*coccidia	1	3	68987.67	35711.53	40
3221	line*coccidia	2	0	65927.33	35711.53	40
3221	line*coccidia	2	1	85693.5	35711.53	40
3221	line*coccidia	2	2	57620.33	35711.53	40
3221	line*coccidia	2	3	171216.7	35711.53	40
3222	line	1		1383463	131240.3	40
3222	line	2		1005948	131240.3	40

3222	coccidia		0	1171278	185601.8	40
3222	coccidia		1	1291081	185601.8	40
3222	coccidia		2	871061.8	185601.8	40
3222	coccidia		3	1445402	185601.8	40
3222	line*coccidia	1	0	1269529	262480.5	40
3222	line*coccidia	1	1	1598175	262480.5	40
3222	line*coccidia	1	2	765815.3	262480.5	40
3222	line*coccidia	1	3	1900333	262480.5	40
3222	line*coccidia	2	0	1073028	262480.5	40
3222	line*coccidia	2	1	983986.8	262480.5	40
3222	line*coccidia	2	2	976308.2	262480.5	40
3222	line*coccidia	2	3	990470.7	262480.5	40
3223	line	1		174089.6	31466.76	40
3223	line	2		97935	31466.76	40
3223	coccidia		0	160738.1	44500.72	40
3223	coccidia		1	198674	44500.72	40
3223	coccidia		2	78082	44500.72	40
3223	coccidia		3	106555.1	44500.72	40
3223	line*coccidia	1	0	134298.5	62933.52	40
3223	line*coccidia	1	1	305961.8	62933.52	40
3223	line*coccidia	1	2	81168.17	62933.52	40
3223	line*coccidia	1	3	174929.8	62933.52	40
3223	line*coccidia	2	0	187177.7	62933.52	40
3223	line*coccidia	2	1	91386.17	62933.52	40
3223	line*coccidia	2	2	74995.83	62933.52	40
3223	line*coccidia	2	3	38180.33	62933.52	40
3225	line	1		292665.2	33256.09	40
3225	line	2		240331.6	33256.09	40
3225	coccidia		0	379206.4	47031.22	40
3225	coccidia		1	273348.5	47031.22	40
3225	coccidia		2	143554.7	47031.22	40
3225	coccidia		3	269884	47031.22	40
3225	line*coccidia	1	0	379931	66512.19	40
3225	line*coccidia	1	1	277930.2	66512.19	40
3225	line*coccidia	1	2	84353.67	66512.19	40
3225	line*coccidia	1	3	428446	66512.19	40
3225	line*coccidia	2	0	378481.8	66512.19	40
3225	line*coccidia	2	1	268766.8	66512.19	40
3225	line*coccidia	2	2	202755.7	66512.19	40
3225	line*coccidia	2	3	111322	66512.19	40
3227	line	1		179702.6	55556.51	40
3227	line	2		57862.58	55556.51	40
3227	coccidia		0	87193.5	78568.78	40
3227	coccidia		1	156926.8	78568.78	40
3227	coccidia		2	78035.17	78568.78	40
3227	coccidia		3	152974.8	78568.78	40
3227	line*coccidia	1	0	66155.33	111113	40
3227	line*coccidia	1	1	263431.2	111113	40
3227	line*coccidia	1	2	95586.5	111113	40

3227	line*coccidia	1	3	293637.3	111113	40
3227	line*coccidia	2	0	108231.7	111113	40
3227	line*coccidia	2	1	50422.5	111113	40
3227	line*coccidia	2	2	60483.83	111113	40
3227	line*coccidia	2	3	12312.33	111113	40
3228	line	1		-1.4E-12	5746.774	40
3228	line	2		47743.08	5746.774	40
3228	coccidia		0	37159.17	8127.166	40
3228	coccidia		1	25535.83	8127.166	40
3228	coccidia		2	15326.08	8127.166	40
3228	coccidia		3	17465.08	8127.166	40
3228	line*coccidia	1	0	0	11493.55	40
3228	line*coccidia	1	1	1.82E-12	11493.55	40
3228	line*coccidia	1	2	0	11493.55	40
3228	line*coccidia	1	3	-7.3E-12	11493.55	40
3228	line*coccidia	2	0	74318.33	11493.55	40
3228	line*coccidia	2	1	51071.67	11493.55	40
3228	line*coccidia	2	2	30652.17	11493.55	40
3228	line*coccidia	2	3	34930.17	11493.55	40
3229	line	1		142075.8	26838.04	40
3229	line	2		37008.21	26838.04	40
3229	coccidia		0	330549.3	37954.72	40
3229	coccidia		1	5.46E-12	37954.72	40
3229	coccidia		2	27618.58	37954.72	40
3229	coccidia		3	1.46E-11	37954.72	40
3229	line*coccidia	1	0	568303	53676.07	40
3229	line*coccidia	1	1	7.28E-12	53676.07	40
3229	line*coccidia	1	2	7.28E-12	53676.07	40
3229	line*coccidia	1	3	7.28E-12	53676.07	40
3229	line*coccidia	2	0	92795.67	53676.07	40
3229	line*coccidia	2	1	3.64E-12	53676.07	40
3229	line*coccidia	2	2	55237.17	53676.07	40
3229	line*coccidia	2	3	2.18E-11	53676.07	40
3235	line	1		306741	128272.7	40
3235	line	2		69487.33	128272.7	40
3235	coccidia		0	239250	181405	40
3235	coccidia		1	431412.5	181405	40
3235	coccidia		2	33718.67	181405	40
3235	coccidia		3	48075.58	181405	40
3235	line*coccidia	1	0	352932.8	256545.4	40
3235	line*coccidia	1	1	796009.8	256545.4	40
3235	line*coccidia	1	2	26123	256545.4	40
3235	line*coccidia	1	3	51898.5	256545.4	40
3235	line*coccidia	2	0	125567.2	256545.4	40
3235	line*coccidia	2	1	66815.17	256545.4	40
3235	line*coccidia	2	2	41314.33	256545.4	40
3235	line*coccidia	2	3	44252.67	256545.4	40
3236	line	1		215572	80525.91	40
3236	line	2		337312.3	80525.91	40

3236	coccidia		0	361768.4	113880.8	40
3236	coccidia		1	330548.3	113880.8	40
3236	coccidia		2	177982.4	113880.8	40
3236	coccidia		3	235469.3	113880.8	40
3236	line*coccidia	1	0	345413.5	161051.8	40
3236	line*coccidia	1	1	45242.83	161051.8	40
3236	line*coccidia	1	2	174740.8	161051.8	40
3236	line*coccidia	1	3	296890.7	161051.8	40
3236	line*coccidia	2	0	378123.3	161051.8	40
3236	line*coccidia	2	1	615853.7	161051.8	40
3236	line*coccidia	2	2	181224	161051.8	40
3236	line*coccidia	2	3	174048	161051.8	40
3238	line	1		215082.4	31265.71	40
3238	line	2		63203.21	31265.71	40
3238	coccidia		0	173000.4	44216.4	40
3238	coccidia		1	187945.9	44216.4	40
3238	coccidia		2	40948.67	44216.4	40
3238	coccidia		3	154676.3	44216.4	40
3238	line*coccidia	1	0	231923.5	62531.43	40
3238	line*coccidia	1	1	314743.8	62531.43	40
3238	line*coccidia	1	2	43715.83	62531.43	40
3238	line*coccidia	1	3	269946.5	62531.43	40
3238	line*coccidia	2	0	114077.3	62531.43	40
3238	line*coccidia	2	1	61148	62531.43	40
3238	line*coccidia	2	2	38181.5	62531.43	40
3238	line*coccidia	2	3	39406	62531.43	40
3302	line	1		1238082	422657.8	40
3302	line	2		1680997	422657.8	40
3302	coccidia		0	1592064	597728.4	40
3302	coccidia		1	2351820	597728.4	40
3302	coccidia		2	908283	597728.4	40
3302	coccidia		3	985992.7	597728.4	40
3302	line*coccidia	1	0	1955169	845315.5	40
3302	line*coccidia	1	1	1086607	845315.5	40
3302	line*coccidia	1	2	388226.8	845315.5	40
3302	line*coccidia	1	3	1522327	845315.5	40
3302	line*coccidia	2	0	1228958	845315.5	40
3302	line*coccidia	2	1	3617034	845315.5	40
3302	line*coccidia	2	2	1428339	845315.5	40
3302	line*coccidia	2	3	449658.7	845315.5	40
3303	line	1		181432.1	31003.65	40
3303	line	2		75224.13	31003.65	40
3303	coccidia		0	60606.83	43845.78	40
3303	coccidia		1	229644.3	43845.78	40
3303	coccidia		2	24148.67	43845.78	40
3303	coccidia		3	198912.6	43845.78	40
3303	line*coccidia	1	0	44033.5	62007.29	40
3303	line*coccidia	1	1	396742.7	62007.29	40
3303	line*coccidia	1	2	25039.33	62007.29	40

3303	line*coccidia	1	3	259912.8	62007.29	40
3303	line*coccidia	2	0	77180.17	62007.29	40
3303	line*coccidia	2	1	62546	62007.29	40
3303	line*coccidia	2	2	23258	62007.29	40
3303	line*coccidia	2	3	137912.3	62007.29	40
3304	line	1		1007018	251362.2	40
3304	line	2		125878.1	251362.2	40
3304	coccidia		0	192825.3	355479.8	40
3304	coccidia		1	1492978	355479.8	40
3304	coccidia		2	49302.58	355479.8	40
3304	coccidia		3	530687.4	355479.8	40
3304	line*coccidia	1	0	301003.5	502724.4	40
3304	line*coccidia	1	1	2865699	502724.4	40
3304	line*coccidia	1	2	42578.83	502724.4	40
3304	line*coccidia	1	3	818792.7	502724.4	40
3304	line*coccidia	2	0	84647	502724.4	40
3304	line*coccidia	2	1	120257	502724.4	40
3304	line*coccidia	2	2	56026.33	502724.4	40
3304	line*coccidia	2	3	242582.2	502724.4	40
3305	line	1		304484.2	103107.4	40
3305	line	2		290144.8	103107.4	40
3305	coccidia		0	239348.4	145815.9	40
3305	coccidia		1	342824.5	145815.9	40
3305	coccidia		2	98306.83	145815.9	40
3305	coccidia		3	508778.2	145815.9	40
3305	line*coccidia	1	0	219846	206214.9	40
3305	line*coccidia	1	1	28926.83	206214.9	40
3305	line*coccidia	1	2	82697.67	206214.9	40
3305	line*coccidia	1	3	886466.3	206214.9	40
3305	line*coccidia	2	0	258850.8	206214.9	40
3305	line*coccidia	2	1	656722.2	206214.9	40
3305	line*coccidia	2	2	113916	206214.9	40
3305	line*coccidia	2	3	131090	206214.9	40
3308	line	1		482845.2	175164.2	40
3308	line	2		419280.3	175164.2	40
3308	coccidia		0	321073	247719.5	40
3308	coccidia		1	557578.4	247719.5	40
3308	coccidia		2	140407.3	247719.5	40
3308	coccidia		3	785192.3	247719.5	40
3308	line*coccidia	1	0	302469.3	350328.3	40
3308	line*coccidia	1	1	213327.8	350328.3	40
3308	line*coccidia	1	2	59071	350328.3	40
3308	line*coccidia	1	3	1356513	350328.3	40
3308	line*coccidia	2	0	339676.7	350328.3	40
3308	line*coccidia	2	1	901829	350328.3	40
3308	line*coccidia	2	2	221743.7	350328.3	40
3308	line*coccidia	2	3	213872	350328.3	40
3309	line	1		264683.7	93892.69	40
3309	line	2		102949.6	93892.69	40

3309	coccidia		0	81887.42	132784.3	40
3309	coccidia		1	188103.2	132784.3	40
3309	coccidia		2	24326.25	132784.3	40
3309	coccidia		3	440949.8	132784.3	40
3309	line*coccidia	1	0	78600	187785.4	40
3309	line*coccidia	1	1	246564.8	187785.4	40
3309	line*coccidia	1	2	10368.17	187785.4	40
3309	line*coccidia	1	3	723201.8	187785.4	40
3309	line*coccidia	2	0	85174.83	187785.4	40
3309	line*coccidia	2	1	129641.5	187785.4	40
3309	line*coccidia	2	2	38284.33	187785.4	40
3309	line*coccidia	2	3	158697.7	187785.4	40
3310	line	1		3724066	809885.1	40
3310	line	2		4841690	809885.1	40
3310	coccidia		0	5206321	1145350	40
3310	coccidia		1	6082565	1145350	40
3310	coccidia		2	2782144	1145350	40
3310	coccidia		3	3060480	1145350	40
3310	line*coccidia	1	0	5828932	1619770	40
3310	line*coccidia	1	1	4030748	1619770	40
3310	line*coccidia	1	2	826922.3	1619770	40
3310	line*coccidia	1	3	4209660	1619770	40
3310	line*coccidia	2	0	4583710	1619770	40
3310	line*coccidia	2	1	8134383	1619770	40
3310	line*coccidia	2	2	4737366	1619770	40
3310	line*coccidia	2	3	1911301	1619770	40
3311	line	1		1217627	291563.7	40
3311	line	2		369758.8	291563.7	40
3311	coccidia		0	1157537	412333.3	40
3311	coccidia		1	680756.9	412333.3	40
3311	coccidia		2	253917.4	412333.3	40
3311	coccidia		3	1082559	412333.3	40
3311	line*coccidia	1	0	2101924	583127.4	40
3311	line*coccidia	1	1	1024009	583127.4	40
3311	line*coccidia	1	2	197078.3	583127.4	40
3311	line*coccidia	1	3	1547495	583127.4	40
3311	line*coccidia	2	0	213150	583127.4	40
3311	line*coccidia	2	1	337504.8	583127.4	40
3311	line*coccidia	2	2	310756.5	583127.4	40
3311	line*coccidia	2	3	617623.7	583127.4	40
3312	line	1		221944.9	46300.12	40
3312	line	2		176697.9	46300.12	40
3312	coccidia		0	289266.9	65478.26	40
3312	coccidia		1	137522.5	65478.26	40
3312	coccidia		2	104136.5	65478.26	40
3312	coccidia		3	266359.6	65478.26	40
3312	line*coccidia	1	0	293046.7	92600.25	40
3312	line*coccidia	1	1	96653.83	92600.25	40
3312	line*coccidia	1	2	55332.83	92600.25	40

3312	line*coccidia	1	3	442746.2	92600.25	40
3312	line*coccidia	2	0	285487.2	92600.25	40
3312	line*coccidia	2	1	178391.2	92600.25	40
3312	line*coccidia	2	2	152940.2	92600.25	40
3312	line*coccidia	2	3	89973	92600.25	40
3313	line	1		2944661	279476.2	40
3313	line	2		2839278	279476.2	40
3313	coccidia		0	4695221	395239.1	40
3313	coccidia		1	2540536	395239.1	40
3313	coccidia		2	1237817	395239.1	40
3313	coccidia		3	3094304	395239.1	40
3313	line*coccidia	1	0	4782477	558952.5	40
3313	line*coccidia	1	1	1872560	558952.5	40
3313	line*coccidia	1	2	829253.5	558952.5	40
3313	line*coccidia	1	3	4294353	558952.5	40
3313	line*coccidia	2	0	4607965	558952.5	40
3313	line*coccidia	2	1	3208512	558952.5	40
3313	line*coccidia	2	2	1646380	558952.5	40
3313	line*coccidia	2	3	1894254	558952.5	40
3314	line	1		1381297	219693.3	40
3314	line	2		1489707	219693.3	40
3314	coccidia		0	1316398	310693.3	40
3314	coccidia		1	2406469	310693.3	40
3314	coccidia		2	910666.2	310693.3	40
3314	coccidia		3	1108474	310693.3	40
3314	line*coccidia	1	0	929878.7	439386.7	40
3314	line*coccidia	1	1	1892395	439386.7	40
3314	line*coccidia	1	2	735769	439386.7	40
3314	line*coccidia	1	3	1967144	439386.7	40
3314	line*coccidia	2	0	1702917	439386.7	40
3314	line*coccidia	2	1	2920543	439386.7	40
3314	line*coccidia	2	2	1085563	439386.7	40
3314	line*coccidia	2	3	249803.5	439386.7	40
3315	line	1		490421.5	116184.8	40
3315	line	2		132314.4	116184.8	40
3315	coccidia		0	198947.9	164310.1	40
3315	coccidia		1	623603.3	164310.1	40
3315	coccidia		2	106658.3	164310.1	40
3315	coccidia		3	316262.4	164310.1	40
3315	line*coccidia	1	0	175151.3	232369.6	40
3315	line*coccidia	1	1	1062852	232369.6	40
3315	line*coccidia	1	2	123853	232369.6	40
3315	line*coccidia	1	3	599829.7	232369.6	40
3315	line*coccidia	2	0	222744.5	232369.6	40
3315	line*coccidia	2	1	184354.5	232369.6	40
3315	line*coccidia	2	2	89463.5	232369.6	40
3315	line*coccidia	2	3	32695.17	232369.6	40
3316	line	1		340512.7	585484.8	40
3316	line	2		835998.6	585484.8	40

3316	coccidia		0	94489.25	828000.6	40
3316	coccidia		1	1747495	828000.6	40
3316	coccidia		2	68203.08	828000.6	40
3316	coccidia		3	442835.1	828000.6	40
3316	line*coccidia	1	0	159876.2	1170970	40
3316	line*coccidia	1	1	234262.3	1170970	40
3316	line*coccidia	1	2	98973.83	1170970	40
3316	line*coccidia	1	3	868938.3	1170970	40
3316	line*coccidia	2	0	29102.33	1170970	40
3316	line*coccidia	2	1	3260728	1170970	40
3316	line*coccidia	2	2	37432.33	1170970	40
3316	line*coccidia	2	3	16731.83	1170970	40
3317	line	1		5249481	605512.6	40
3317	line	2		4213406	605512.6	40
3317	coccidia		0	7968363	856324.1	40
3317	coccidia		1	4270167	856324.1	40
3317	coccidia		2	1773342	856324.1	40
3317	coccidia		3	4913902	856324.1	40
3317	line*coccidia	1	0	9547860	1211025	40
3317	line*coccidia	1	1	3807071	1211025	40
3317	line*coccidia	1	2	1062571	1211025	40
3317	line*coccidia	1	3	6580422	1211025	40
3317	line*coccidia	2	0	6388867	1211025	40
3317	line*coccidia	2	1	4733263	1211025	40
3317	line*coccidia	2	2	2484114	1211025	40
3317	line*coccidia	2	3	3247381	1211025	40
3318	line	1		8577815	755646.7	40
3318	line	2		8093697	755646.7	40
3318	coccidia		0	10488694	1068646	40
3318	coccidia		1	8091183	1068646	40
3318	coccidia		2	6996029	1068646	40
3318	coccidia		3	7767117	1068646	40
3318	line*coccidia	1	0	10613839	1511293	40
3318	line*coccidia	1	1	6181149	1511293	40
3318	line*coccidia	1	2	6204698	1511293	40
3318	line*coccidia	1	3	11311574	1511293	40
3318	line*coccidia	2	0	10363550	1511293	40
3318	line*coccidia	2	1	10001218	1511293	40
3318	line*coccidia	2	2	7787359	1511293	40
3318	line*coccidia	2	3	4222659	1511293	40
3319	line	1		580207.2	1176083	40
3319	line	2		1847270	1176083	40
3319	coccidia		0	335155.8	1663232	40
3319	coccidia		1	3616497	1663232	40
3319	coccidia		2	529732	1663232	40
3319	coccidia		3	373569.1	1663232	40
3319	line*coccidia	1	0	454423.8	2352165	40
3319	line*coccidia	1	1	428620.5	2352165	40
3319	line*coccidia	1	2	922384.3	2352165	40

3319	line*coccidia	1	3	515400.2	2352165	40
3319	line*coccidia	2	0	215887.7	2352165	40
3319	line*coccidia	2	1	6804374	2352165	40
3319	line*coccidia	2	2	137079.7	2352165	40
3319	line*coccidia	2	3	231738	2352165	40
3320	line	1		1051645	148112.6	40
3320	line	2		733598.4	148112.6	40
3320	coccidia		0	933472.8	209462.8	40
3320	coccidia		1	915467.2	209462.8	40
3320	coccidia		2	584051.3	209462.8	40
3320	coccidia		3	1137496	209462.8	40
3320	line*coccidia	1	0	1102658	296225.1	40
3320	line*coccidia	1	1	1008402	296225.1	40
3320	line*coccidia	1	2	389061.8	296225.1	40
3320	line*coccidia	1	3	1706459	296225.1	40
3320	line*coccidia	2	0	764288	296225.1	40
3320	line*coccidia	2	1	822532	296225.1	40
3320	line*coccidia	2	2	779040.8	296225.1	40
3320	line*coccidia	2	3	568532.8	296225.1	40
3321	line	1		536204.9	130525.6	40
3321	line	2		198782	130525.6	40
3321	coccidia		0	458908.6	184591	40
3321	coccidia		1	376006.3	184591	40
3321	coccidia		2	281768.5	184591	40
3321	coccidia		3	353290.4	184591	40
3321	line*coccidia	1	0	799887.7	261051.1	40
3321	line*coccidia	1	1	606817	261051.1	40
3321	line*coccidia	1	2	500569.7	261051.1	40
3321	line*coccidia	1	3	237545.2	261051.1	40
3321	line*coccidia	2	0	117929.5	261051.1	40
3321	line*coccidia	2	1	145195.7	261051.1	40
3321	line*coccidia	2	2	62967.33	261051.1	40
3321	line*coccidia	2	3	469035.7	261051.1	40
3322	line	1		755700	345220	40
3322	line	2		696284.8	345220	40
3322	coccidia		0	558199.3	488214.8	40
3322	coccidia		1	1222067	488214.8	40
3322	coccidia		2	681793	488214.8	40
3322	coccidia		3	441910.1	488214.8	40
3322	line*coccidia	1	0	816278.2	690440	40
3322	line*coccidia	1	1	403584.3	690440	40
3322	line*coccidia	1	2	1176138	690440	40
3322	line*coccidia	1	3	626799.8	690440	40
3322	line*coccidia	2	0	300120.3	690440	40
3322	line*coccidia	2	1	2040550	690440	40
3322	line*coccidia	2	2	187448.3	690440	40
3322	line*coccidia	2	3	257020.3	690440	40
3323	line	1		7552834	546481.6	40
3323	line	2		6275500	546481.6	40

3323	coccidia		0	9665192	772841.8	40
3323	coccidia		1	4883637	772841.8	40
3323	coccidia		2	5588646	772841.8	40
3323	coccidia		3	7519192	772841.8	40
3323	line*coccidia	1	0	9749277	1092963	40
3323	line*coccidia	1	1	4840396	1092963	40
3323	line*coccidia	1	2	4685506	1092963	40
3323	line*coccidia	1	3	10936156	1092963	40
3323	line*coccidia	2	0	9581106	1092963	40
3323	line*coccidia	2	1	4926878	1092963	40
3323	line*coccidia	2	2	6491786	1092963	40
3323	line*coccidia	2	3	4102228	1092963	40
3324	line	1		5943003	500915.8	40
3324	line	2		4434419	500915.8	40
3324	coccidia		0	6604644	708401.9	40
3324	coccidia		1	4772181	708401.9	40
3324	coccidia		2	3805081	708401.9	40
3324	coccidia		3	5572939	708401.9	40
3324	line*coccidia	1	0	6990140	1001832	40
3324	line*coccidia	1	1	4964030	1001832	40
3324	line*coccidia	1	2	3839857	1001832	40
3324	line*coccidia	1	3	7977984	1001832	40
3324	line*coccidia	2	0	6219148	1001832	40
3324	line*coccidia	2	1	4580332	1001832	40
3324	line*coccidia	2	2	3770306	1001832	40
3324	line*coccidia	2	3	3167893	1001832	40
3329	line	1		1268226	272243.9	40
3329	line	2		535849	272243.9	40
3329	coccidia		0	803409	385011.1	40
3329	coccidia		1	603892.9	385011.1	40
3329	coccidia		2	754319.1	385011.1	40
3329	coccidia		3	1446528	385011.1	40
3329	line*coccidia	1	0	956937.3	544487.9	40
3329	line*coccidia	1	1	248103.7	544487.9	40
3329	line*coccidia	1	2	1147670	544487.9	40
3329	line*coccidia	1	3	2720191	544487.9	40
3329	line*coccidia	2	0	649880.7	544487.9	40
3329	line*coccidia	2	1	959682.2	544487.9	40
3329	line*coccidia	2	2	360967.8	544487.9	40
3329	line*coccidia	2	3	172865.3	544487.9	40
3330	line	1		293859	95196.52	40
3330	line	2		460710.6	95196.52	40
3330	coccidia		0	377310.2	134628.2	40
3330	coccidia		1	631916.8	134628.2	40
3330	coccidia		2	231519.8	134628.2	40
3330	coccidia		3	268392.3	134628.2	40
3330	line*coccidia	1	0	414914.5	190393	40
3330	line*coccidia	1	1	316534	190393	40
3330	line*coccidia	1	2	87848.5	190393	40

3330	line*coccidia	1	3	356138.8	190393	40
3330	line*coccidia	2	0	339705.8	190393	40
3330	line*coccidia	2	1	947299.7	190393	40
3330	line*coccidia	2	2	375191.2	190393	40
3330	line*coccidia	2	3	180645.8	190393	40
3331	line	1		12280.67	171626.7	40
3331	line	2		279583.9	171626.7	40
3331	coccidia		0	58239.17	242716.8	40
3331	coccidia		1	499453.4	242716.8	40
3331	coccidia		2	26036.58	242716.8	40
3331	coccidia		3	0	242716.8	40
3331	line*coccidia	1	0	49122.67	343253.4	40
3331	line*coccidia	1	1	-2E-11	343253.4	40
3331	line*coccidia	1	2	-2E-11	343253.4	40
3331	line*coccidia	1	3	-2E-11	343253.4	40
3331	line*coccidia	2	0	67355.67	343253.4	40
3331	line*coccidia	2	1	998906.8	343253.4	40
3331	line*coccidia	2	2	52073.17	343253.4	40
3331	line*coccidia	2	3	2E-11	343253.4	40
3333	line	1		939328.2	182285.2	40
3333	line	2		784896.3	182285.2	40
3333	coccidia		0	688636.8	257790.2	40
3333	coccidia		1	832836.8	257790.2	40
3333	coccidia		2	460428.3	257790.2	40
3333	coccidia		3	1466547	257790.2	40
3333	line*coccidia	1	0	838955.8	364570.3	40
3333	line*coccidia	1	1	1141649	364570.3	40
3333	line*coccidia	1	2	488084.5	364570.3	40
3333	line*coccidia	1	3	1288624	364570.3	40
3333	line*coccidia	2	0	538317.7	364570.3	40
3333	line*coccidia	2	1	524024.8	364570.3	40
3333	line*coccidia	2	2	432772.2	364570.3	40
3333	line*coccidia	2	3	1644471	364570.3	40
3334	line	1		-2.5E-11	26179.47	40
3334	line	2		214197	26179.47	40
3334	coccidia		0	95519.58	37023.36	40
3334	coccidia		1	290372.4	37023.36	40
3334	coccidia		2	42502	37023.36	40
3334	coccidia		3	-2.9E-11	37023.36	40
3334	line*coccidia	1	0	-4.7E-11	52358.94	40
3334	line*coccidia	1	1	-1.8E-11	52358.94	40
3334	line*coccidia	1	2	-1.8E-11	52358.94	40
3334	line*coccidia	1	3	-1.8E-11	52358.94	40
3334	line*coccidia	2	0	191039.2	52358.94	40
3334	line*coccidia	2	1	580744.8	52358.94	40
3334	line*coccidia	2	2	85004	52358.94	40
3334	line*coccidia	2	3	-4E-11	52358.94	40
3335	line	1		892068.8	720185.5	40
3335	line	2		1383724	720185.5	40

3335	coccidia		0	438369.3	1018496	40
3335	coccidia		1	342441.8	1018496	40
3335	coccidia		2	863353.5	1018496	40
3335	coccidia		3	2907422	1018496	40
3335	line*coccidia	1	0	568627.8	1440371	40
3335	line*coccidia	1	1	458815.5	1440371	40
3335	line*coccidia	1	2	1538118	1440371	40
3335	line*coccidia	1	3	1002714	1440371	40
3335	line*coccidia	2	0	308110.7	1440371	40
3335	line*coccidia	2	1	226068	1440371	40
3335	line*coccidia	2	2	188589.3	1440371	40
3335	line*coccidia	2	3	4812130	1440371	40
3401	line	1		11180057	1258530	40
3401	line	2		9443602	1258530	40
3401	coccidia		0	11089284	1779830	40
3401	coccidia		1	10020784	1779830	40
3401	coccidia		2	8205266	1779830	40
3401	coccidia		3	11931985	1779830	40
3401	line*coccidia	1	0	14442669	2517059	40
3401	line*coccidia	1	1	10823014	2517059	40
3401	line*coccidia	1	2	8546842	2517059	40
3401	line*coccidia	1	3	10907704	2517059	40
3401	line*coccidia	2	0	7735898	2517059	40
3401	line*coccidia	2	1	9218554	2517059	40
3401	line*coccidia	2	2	7863690	2517059	40
3401	line*coccidia	2	3	12956267	2517059	40
3403	line	1		1316879	192976.7	40
3403	line	2		787133.6	192976.7	40
3403	coccidia		0	1449882	272910.3	40
3403	coccidia		1	1023794	272910.3	40
3403	coccidia		2	316320.1	272910.3	40
3403	coccidia		3	1418028	272910.3	40
3403	line*coccidia	1	0	2089915	385953.5	40
3403	line*coccidia	1	1	1081808	385953.5	40
3403	line*coccidia	1	2	292683.7	385953.5	40
3403	line*coccidia	1	3	1803108	385953.5	40
3403	line*coccidia	2	0	809850	385953.5	40
3403	line*coccidia	2	1	965779.7	385953.5	40
3403	line*coccidia	2	2	339956.5	385953.5	40
3403	line*coccidia	2	3	1032948	385953.5	40
3405	line	1		7094912	603153.9	40
3405	line	2		6246613	603153.9	40
3405	coccidia		0	8179972	852988.4	40
3405	coccidia		1	6214970	852988.4	40
3405	coccidia		2	5268733	852988.4	40
3405	coccidia		3	7019376	852988.4	40
3405	line*coccidia	1	0	8349201	1206308	40
3405	line*coccidia	1	1	5639791	1206308	40
3405	line*coccidia	1	2	3914809	1206308	40

3405	line*coccidia	1	3	10475847	1206308	40
3405	line*coccidia	2	0	8010743	1206308	40
3405	line*coccidia	2	1	6790149	1206308	40
3405	line*coccidia	2	2	6622657	1206308	40
3405	line*coccidia	2	3	3562906	1206308	40
3407	line	1		1166119	194823	40
3407	line	2		895885.5	194823	40
3407	coccidia		0	1371825	275521.4	40
3407	coccidia		1	877435	275521.4	40
3407	coccidia		2	716007.3	275521.4	40
3407	coccidia		3	1158743	275521.4	40
3407	line*coccidia	1	0	1610917	389646.1	40
3407	line*coccidia	1	1	865956.2	389646.1	40
3407	line*coccidia	1	2	329239.3	389646.1	40
3407	line*coccidia	1	3	1858364	389646.1	40
3407	line*coccidia	2	0	1132732	389646.1	40
3407	line*coccidia	2	1	888913.8	389646.1	40
3407	line*coccidia	2	2	1102775	389646.1	40
3407	line*coccidia	2	3	459121.2	389646.1	40
3408	line	1		1204116	171752.7	40
3408	line	2		849533.1	171752.7	40
3408	coccidia		0	1542423	242895	40
3408	coccidia		1	649064.1	242895	40
3408	coccidia		2	500170.6	242895	40
3408	coccidia		3	1415641	242895	40
3408	line*coccidia	1	0	2068210	343505.4	40
3408	line*coccidia	1	1	505790.5	343505.4	40
3408	line*coccidia	1	2	282989.7	343505.4	40
3408	line*coccidia	1	3	1959475	343505.4	40
3408	line*coccidia	2	0	1016637	343505.4	40
3408	line*coccidia	2	1	792337.7	343505.4	40
3408	line*coccidia	2	2	717351.5	343505.4	40
3408	line*coccidia	2	3	871806.3	343505.4	40
3410	line	1		5229442	401059.7	40
3410	line	2		4852645	401059.7	40
3410	coccidia		0	5753778	567184.1	40
3410	coccidia		1	4171238	567184.1	40
3410	coccidia		2	4412753	567184.1	40
3410	coccidia		3	5826403	567184.1	40
3410	line*coccidia	1	0	6030909	802119.4	40
3410	line*coccidia	1	1	3230821	802119.4	40
3410	line*coccidia	1	2	2987324	802119.4	40
3410	line*coccidia	1	3	8668712	802119.4	40
3410	line*coccidia	2	0	5476647	802119.4	40
3410	line*coccidia	2	1	5111655	802119.4	40
3410	line*coccidia	2	2	5838183	802119.4	40
3410	line*coccidia	2	3	2984094	802119.4	40
3411	line	1		1224810	293748.5	40
3411	line	2		527138	293748.5	40

3411	coccidia		0	1614338	415423.2	40
3411	coccidia		1	440242.4	415423.2	40
3411	coccidia		2	366342	415423.2	40
3411	coccidia		3	1082975	415423.2	40
3411	line*coccidia	1	0	2669781	587497.1	40
3411	line*coccidia	1	1	357237.2	587497.1	40
3411	line*coccidia	1	2	222724.5	587497.1	40
3411	line*coccidia	1	3	1649500	587497.1	40
3411	line*coccidia	2	0	558895.2	587497.1	40
3411	line*coccidia	2	1	523247.7	587497.1	40
3411	line*coccidia	2	2	509959.5	587497.1	40
3411	line*coccidia	2	3	516449.5	587497.1	40
3412	line	1		2120199	262570.7	40
3412	line	2		2009896	262570.7	40
3412	coccidia		0	2782701	371331.1	40
3412	coccidia		1	1212809	371331.1	40
3412	coccidia		2	2023558	371331.1	40
3412	coccidia		3	2241123	371331.1	40
3412	line*coccidia	1	0	3318799	525141.5	40
3412	line*coccidia	1	1	622354.5	525141.5	40
3412	line*coccidia	1	2	2100200	525141.5	40
3412	line*coccidia	1	3	2439442	525141.5	40
3412	line*coccidia	2	0	2246604	525141.5	40
3412	line*coccidia	2	1	1803264	525141.5	40
3412	line*coccidia	2	2	1946915	525141.5	40
3412	line*coccidia	2	3	2042804	525141.5	40
3414	line	1		3616852	479511.1	40
3414	line	2		3283356	479511.1	40
3414	coccidia		0	2834127	678131.2	40
3414	coccidia		1	3679334	678131.2	40
3414	coccidia		2	3754909	678131.2	40
3414	coccidia		3	3532047	678131.2	40
3414	line*coccidia	1	0	2751065	959022.3	40
3414	line*coccidia	1	1	3415482	959022.3	40
3414	line*coccidia	1	2	4383720	959022.3	40
3414	line*coccidia	1	3	3917143	959022.3	40
3414	line*coccidia	2	0	2917190	959022.3	40
3414	line*coccidia	2	1	3943186	959022.3	40
3414	line*coccidia	2	2	3126097	959022.3	40
3414	line*coccidia	2	3	3146952	959022.3	40
3417	line	1		60535741	6713358	40
3417	line	2		51130239	6713358	40
3417	coccidia		0	48435852	9494122	40
3417	coccidia		1	49577294	9494122	40
3417	coccidia		2	73352734	9494122	40
3417	coccidia		3	51966080	9494122	40
3417	line*coccidia	1	0	47857537	13426716	40
3417	line*coccidia	1	1	58548084	13426716	40
3417	line*coccidia	1	2	78079991	13426716	40

3417	line*coccidia	1	3	57657351	13426716	40
3417	line*coccidia	2	0	49014168	13426716	40
3417	line*coccidia	2	1	40606504	13426716	40
3417	line*coccidia	2	2	68625477	13426716	40
3417	line*coccidia	2	3	46274808	13426716	40
3418	line	1		8424245	3068689	40
3418	line	2		5599440	3068689	40
3418	coccidia		0	2924296	4339781	40
3418	coccidia		1	4453106	4339781	40
3418	coccidia		2	16880903	4339781	40
3418	coccidia		3	3789065	4339781	40
3418	line*coccidia	1	0	2595638	6137378	40
3418	line*coccidia	1	1	5776267	6137378	40
3418	line*coccidia	1	2	20206474	6137378	40
3418	line*coccidia	1	3	5118601	6137378	40
3418	line*coccidia	2	0	3252955	6137378	40
3418	line*coccidia	2	1	3129945	6137378	40
3418	line*coccidia	2	2	13555331	6137378	40
3418	line*coccidia	2	3	2459529	6137378	40
3419	line	1		10865134	3724375	40
3419	line	2		5943542	3724375	40
3419	coccidia		0	3683746	5267062	40
3419	coccidia		1	6566282	5267062	40
3419	coccidia		2	13579629	5267062	40
3419	coccidia		3	9787693	5267062	40
3419	line*coccidia	1	0	3684582	7448751	40
3419	line*coccidia	1	1	9880997	7448751	40
3419	line*coccidia	1	2	25356814	7448751	40
3419	line*coccidia	1	3	4538141	7448751	40
3419	line*coccidia	2	0	3682911	7448751	40
3419	line*coccidia	2	1	3251567	7448751	40
3419	line*coccidia	2	2	1802445	7448751	40
3419	line*coccidia	2	3	15037244	7448751	40
3424	line	1		16187384	2089632	40
3424	line	2		19482268	2089632	40
3424	coccidia		0	24097196	2955186	40
3424	coccidia		1	10172918	2955186	40
3424	coccidia		2	21902314	2955186	40
3424	coccidia		3	15166876	2955186	40
3424	line*coccidia	1	0	30628004	4179265	40
3424	line*coccidia	1	1	11772300	4179265	40
3424	line*coccidia	1	2	12672762	4179265	40
3424	line*coccidia	1	3	9676472	4179265	40
3424	line*coccidia	2	0	17566388	4179265	40
3424	line*coccidia	2	1	8573537	4179265	40
3424	line*coccidia	2	2	31131867	4179265	40
3424	line*coccidia	2	3	20657281	4179265	40
3425	line	1		10457250	1011000	40
3425	line	2		11795984	1011000	40

3425	coccidia		0	16605191	1429770	40
3425	coccidia		1	6700408	1429770	40
3425	coccidia		2	13455464	1429770	40
3425	coccidia		3	7745405	1429770	40
3425	line*coccidia	1	0	19361592	2021999	40
3425	line*coccidia	1	1	5217657	2021999	40
3425	line*coccidia	1	2	8570183	2021999	40
3425	line*coccidia	1	3	8679570	2021999	40
3425	line*coccidia	2	0	13848791	2021999	40
3425	line*coccidia	2	1	8183159	2021999	40
3425	line*coccidia	2	2	18340745	2021999	40
3425	line*coccidia	2	3	6811241	2021999	40
3426	line	1		1806249	669972.8	40
3426	line	2		639589.3	669972.8	40
3426	coccidia		0	580133.5	947484.6	40
3426	coccidia		1	1268467	947484.6	40
3426	coccidia		2	331752.9	947484.6	40
3426	coccidia		3	2711323	947484.6	40
3426	line*coccidia	1	0	717812.3	1339946	40
3426	line*coccidia	1	1	1632910	1339946	40
3426	line*coccidia	1	2	390643	1339946	40
3426	line*coccidia	1	3	4483632	1339946	40
3426	line*coccidia	2	0	442454.7	1339946	40
3426	line*coccidia	2	1	904024.8	1339946	40
3426	line*coccidia	2	2	272862.8	1339946	40
3426	line*coccidia	2	3	939015	1339946	40
3427	line	1		1662200	512115	40
3427	line	2		931948.8	512115	40
3427	coccidia		0	1759433	724239.9	40
3427	coccidia		1	634236	724239.9	40
3427	coccidia		2	840821.6	724239.9	40
3427	coccidia		3	1953808	724239.9	40
3427	line*coccidia	1	0	2814209	1024230	40
3427	line*coccidia	1	1	663000.3	1024230	40
3427	line*coccidia	1	2	1527162	1024230	40
3427	line*coccidia	1	3	1644430	1024230	40
3427	line*coccidia	2	0	704655.8	1024230	40
3427	line*coccidia	2	1	605471.7	1024230	40
3427	line*coccidia	2	2	154480.8	1024230	40
3427	line*coccidia	2	3	2263187	1024230	40
3502	line	1		821466.1	124988.3	40
3502	line	2		874243.1	124988.3	40
3502	coccidia		0	666215.2	176760.1	40
3502	coccidia		1	1403840	176760.1	40
3502	coccidia		2	388929.8	176760.1	40
3502	coccidia		3	932433.2	176760.1	40
3502	line*coccidia	1	0	599769	249976.5	40
3502	line*coccidia	1	1	1174966	249976.5	40
3502	line*coccidia	1	2	279341.8	249976.5	40

3502	line*coccidia	1	3	1231788	249976.5	40
3502	line*coccidia	2	0	732661.3	249976.5	40
3502	line*coccidia	2	1	1632715	249976.5	40
3502	line*coccidia	2	2	498517.7	249976.5	40
3502	line*coccidia	2	3	633078.7	249976.5	40
3504	line	1		884760.5	135191.2	40
3504	line	2		650338	135191.2	40
3504	coccidia		0	436426.3	191189.2	40
3504	coccidia		1	1256521	191189.2	40
3504	coccidia		2	560870.7	191189.2	40
3504	coccidia		3	816379.1	191189.2	40
3504	line*coccidia	1	0	502806.8	270382.4	40
3504	line*coccidia	1	1	1531963	270382.4	40
3504	line*coccidia	1	2	432291.8	270382.4	40
3504	line*coccidia	1	3	1071981	270382.4	40
3504	line*coccidia	2	0	370045.7	270382.4	40
3504	line*coccidia	2	1	981079.5	270382.4	40
3504	line*coccidia	2	2	689449.5	270382.4	40
3504	line*coccidia	2	3	560777.3	270382.4	40
3505	line	1		611577	109119.1	40
3505	line	2		923059.4	109119.1	40
3505	coccidia		0	763462.8	154317.7	40
3505	coccidia		1	1116307	154317.7	40
3505	coccidia		2	438109.8	154317.7	40
3505	coccidia		3	751393.4	154317.7	40
3505	line*coccidia	1	0	890628.3	218238.2	40
3505	line*coccidia	1	1	549498.2	218238.2	40
3505	line*coccidia	1	2	173817.5	218238.2	40
3505	line*coccidia	1	3	832363.8	218238.2	40
3505	line*coccidia	2	0	636297.3	218238.2	40
3505	line*coccidia	2	1	1683115	218238.2	40
3505	line*coccidia	2	2	702402	218238.2	40
3505	line*coccidia	2	3	670423	218238.2	40
3506	line	1		638340.9	101525.7	40
3506	line	2		542810.6	101525.7	40
3506	coccidia		0	345693.3	143579	40
3506	coccidia		1	704129.3	143579	40
3506	coccidia		2	600433.4	143579	40
3506	coccidia		3	712047	143579	40
3506	line*coccidia	1	0	413982.2	203051.3	40
3506	line*coccidia	1	1	605487.8	203051.3	40
3506	line*coccidia	1	2	431661.8	203051.3	40
3506	line*coccidia	1	3	1102232	203051.3	40
3506	line*coccidia	2	0	277404.5	203051.3	40
3506	line*coccidia	2	1	802770.8	203051.3	40
3506	line*coccidia	2	2	769205	203051.3	40
3506	line*coccidia	2	3	321862.2	203051.3	40
3508	line	1		1041656	147523	40
3508	line	2		965597.5	147523	40

3508	coccidia		0	1016582	208629.1	40
3508	coccidia		1	1049413	208629.1	40
3508	coccidia		2	617818.7	208629.1	40
3508	coccidia		3	1330693	208629.1	40
3508	line*coccidia	1	0	1200743	295046.1	40
3508	line*coccidia	1	1	582893.5	295046.1	40
3508	line*coccidia	1	2	360262.2	295046.1	40
3508	line*coccidia	1	3	2022723	295046.1	40
3508	line*coccidia	2	0	832420.3	295046.1	40
3508	line*coccidia	2	1	1515933	295046.1	40
3508	line*coccidia	2	2	875375.2	295046.1	40
3508	line*coccidia	2	3	638661.8	295046.1	40
3510	line	1		841766.2	103723.4	40
3510	line	2		1345824	103723.4	40
3510	coccidia		0	1453926	146687.1	40
3510	coccidia		1	934811.5	146687.1	40
3510	coccidia		2	994418.1	146687.1	40
3510	coccidia		3	992024.8	146687.1	40
3510	line*coccidia	1	0	1359384	207446.9	40
3510	line*coccidia	1	1	306662.3	207446.9	40
3510	line*coccidia	1	2	476495.3	207446.9	40
3510	line*coccidia	1	3	1224524	207446.9	40
3510	line*coccidia	2	0	1548469	207446.9	40
3510	line*coccidia	2	1	1562961	207446.9	40
3510	line*coccidia	2	2	1512341	207446.9	40
3510	line*coccidia	2	3	759526	207446.9	40
3512	line	1		994063.9	5452314	40
3512	line	2		14197000	5452314	40
3512	coccidia		0	3016294	7710736	40
3512	coccidia		1	780606.2	7710736	40
3512	coccidia		2	6813630	7710736	40
3512	coccidia		3	19771598	7710736	40
3512	line*coccidia	1	0	1721578	10904628	40
3512	line*coccidia	1	1	410509.8	10904628	40
3512	line*coccidia	1	2	625528.7	10904628	40
3512	line*coccidia	1	3	1218639	10904628	40
3512	line*coccidia	2	0	4311010	10904628	40
3512	line*coccidia	2	1	1150703	10904628	40
3512	line*coccidia	2	2	13001732	10904628	40
3512	line*coccidia	2	3	38324557	10904628	40
3518	line	1		108196	28307.16	40
3518	line	2		84623.96	28307.16	40
3518	coccidia		0	75448.08	40032.37	40
3518	coccidia		1	72908	40032.37	40
3518	coccidia		2	57383.08	40032.37	40
3518	coccidia		3	179900.8	40032.37	40
3518	line*coccidia	1	0	102555	56614.32	40
3518	line*coccidia	1	1	1.46E-11	56614.32	40
3518	line*coccidia	1	2	31752.67	56614.32	40

3518	line*coccidia	1	3	298476.3	56614.32	40
3518	line*coccidia	2	0	48341.17	56614.32	40
3518	line*coccidia	2	1	145816	56614.32	40
3518	line*coccidia	2	2	83013.5	56614.32	40
3518	line*coccidia	2	3	61325.17	56614.32	40
3519	line	1		1074167	387394.9	40
3519	line	2		1636850	387394.9	40
3519	coccidia		0	1928636	547859.1	40
3519	coccidia		1	696766.6	547859.1	40
3519	coccidia		2	1430396	547859.1	40
3519	coccidia		3	1366235	547859.1	40
3519	line*coccidia	1	0	1530074	774789.8	40
3519	line*coccidia	1	1	343107.3	774789.8	40
3519	line*coccidia	1	2	432482.7	774789.8	40
3519	line*coccidia	1	3	1991006	774789.8	40
3519	line*coccidia	2	0	2327199	774789.8	40
3519	line*coccidia	2	1	1050426	774789.8	40
3519	line*coccidia	2	2	2428310	774789.8	40
3519	line*coccidia	2	3	741464.8	774789.8	40
3520	line	1		498348.6	94571.36	40
3520	line	2		518122.9	94571.36	40
3520	coccidia		0	273171.9	133744.1	40
3520	coccidia		1	513662.1	133744.1	40
3520	coccidia		2	639463.6	133744.1	40
3520	coccidia		3	606645.3	133744.1	40
3520	line*coccidia	1	0	261585.3	189142.7	40
3520	line*coccidia	1	1	508821.8	189142.7	40
3520	line*coccidia	1	2	262325.2	189142.7	40
3520	line*coccidia	1	3	960662	189142.7	40
3520	line*coccidia	2	0	284758.5	189142.7	40
3520	line*coccidia	2	1	518502.3	189142.7	40
3520	line*coccidia	2	2	1016602	189142.7	40
3520	line*coccidia	2	3	252628.7	189142.7	40
3521	line	1		348738.7	74152.75	40
3521	line	2		195110.8	74152.75	40
3521	coccidia		0	135336.5	104867.8	40
3521	coccidia		1	315123.8	104867.8	40
3521	coccidia		2	136509.8	104867.8	40
3521	coccidia		3	500729	104867.8	40
3521	line*coccidia	1	0	195855.7	148305.5	40
3521	line*coccidia	1	1	133007.2	148305.5	40
3521	line*coccidia	1	2	154872	148305.5	40
3521	line*coccidia	1	3	911219.8	148305.5	40
3521	line*coccidia	2	0	74817.33	148305.5	40
3521	line*coccidia	2	1	497240.3	148305.5	40
3521	line*coccidia	2	2	118147.5	148305.5	40
3521	line*coccidia	2	3	90238.17	148305.5	40
3523	line	1		19346014	3821368	40
3523	line	2		17185107	3821368	40

3523	coccidia		0	23179364	5404230	40
3523	coccidia		1	7662386	5404230	40
3523	coccidia		2	23722972	5404230	40
3523	coccidia		3	18497520	5404230	40
3523	line*coccidia	1	0	30004924	7642735	40
3523	line*coccidia	1	1	8005200	7642735	40
3523	line*coccidia	1	2	23775037	7642735	40
3523	line*coccidia	1	3	15598895	7642735	40
3523	line*coccidia	2	0	16353804	7642735	40
3523	line*coccidia	2	1	7319573	7642735	40
3523	line*coccidia	2	2	23670906	7642735	40
3523	line*coccidia	2	3	21396144	7642735	40
3526	line	1		1054771	365129.6	40
3526	line	2		1771302	365129.6	40
3526	coccidia		0	1524735	516371.2	40
3526	coccidia		1	947019.6	516371.2	40
3526	coccidia		2	1869475	516371.2	40
3526	coccidia		3	1310917	516371.2	40
3526	line*coccidia	1	0	1292132	730259.2	40
3526	line*coccidia	1	1	377684	730259.2	40
3526	line*coccidia	1	2	363657	730259.2	40
3526	line*coccidia	1	3	2185611	730259.2	40
3526	line*coccidia	2	0	1757338	730259.2	40
3526	line*coccidia	2	1	1516355	730259.2	40
3526	line*coccidia	2	2	3375293	730259.2	40
3526	line*coccidia	2	3	436223.3	730259.2	40
3602	line	1		3596483	778738.4	40
3602	line	2		5018120	778738.4	40
3602	coccidia		0	6795119	1101302	40
3602	coccidia		1	4369457	1101302	40
3602	coccidia		2	3028099	1101302	40
3602	coccidia		3	3036531	1101302	40
3602	line*coccidia	1	0	4877438	1557477	40
3602	line*coccidia	1	1	5604914	1557477	40
3602	line*coccidia	1	2	915490.5	1557477	40
3602	line*coccidia	1	3	2988091	1557477	40
3602	line*coccidia	2	0	8712800	1557477	40
3602	line*coccidia	2	1	3134000	1557477	40
3602	line*coccidia	2	2	5140708	1557477	40
3602	line*coccidia	2	3	3084970	1557477	40
3606	line	1		314404.5	59899.3	40
3606	line	2		169912	59899.3	40
3606	coccidia		0	138524.8	84710.4	40
3606	coccidia		1	552426	84710.4	40
3606	coccidia		2	64721.42	84710.4	40
3606	coccidia		3	212960.7	84710.4	40
3606	line*coccidia	1	0	178041.3	119798.6	40
3606	line*coccidia	1	1	652386.3	119798.6	40
3606	line*coccidia	1	2	79995	119798.6	40

3606	line*coccidia	1	3	347195.3	119798.6	40
3606	line*coccidia	2	0	99008.33	119798.6	40
3606	line*coccidia	2	1	452465.7	119798.6	40
3606	line*coccidia	2	2	49447.83	119798.6	40
3606	line*coccidia	2	3	78726	119798.6	40
3609	line	1		501091.4	210931.9	40
3609	line	2		279989	210931.9	40
3609	coccidia		0	131303.5	298302.8	40
3609	coccidia		1	468456.8	298302.8	40
3609	coccidia		2	57240.08	298302.8	40
3609	coccidia		3	905160.3	298302.8	40
3609	line*coccidia	1	0	209286.3	421863.8	40
3609	line*coccidia	1	1	449711.3	421863.8	40
3609	line*coccidia	1	2	33614.83	421863.8	40
3609	line*coccidia	1	3	1311753	421863.8	40
3609	line*coccidia	2	0	53320.67	421863.8	40
3609	line*coccidia	2	1	487202.3	421863.8	40
3609	line*coccidia	2	2	80865.33	421863.8	40
3609	line*coccidia	2	3	498567.7	421863.8	40
3611	line	1		568636.6	149860.6	40
3611	line	2		806918.5	149860.6	40
3611	coccidia		0	666519.9	211934.9	40
3611	coccidia		1	713558.8	211934.9	40
3611	coccidia		2	484250.5	211934.9	40
3611	coccidia		3	886781.1	211934.9	40
3611	line*coccidia	1	0	487524.8	299721.3	40
3611	line*coccidia	1	1	462386	299721.3	40
3611	line*coccidia	1	2	259201.2	299721.3	40
3611	line*coccidia	1	3	1065435	299721.3	40
3611	line*coccidia	2	0	845515	299721.3	40
3611	line*coccidia	2	1	964731.5	299721.3	40
3611	line*coccidia	2	2	709299.8	299721.3	40
3611	line*coccidia	2	3	708127.7	299721.3	40
3612	line	1		1082829	195087	40
3612	line	2		261648.5	195087	40
3612	coccidia		0	1030901	275894.7	40
3612	coccidia		1	750400.2	275894.7	40
3612	coccidia		2	213921	275894.7	40
3612	coccidia		3	693734.1	275894.7	40
3612	line*coccidia	1	0	1826100	390174	40
3612	line*coccidia	1	1	1084777	390174	40
3612	line*coccidia	1	2	248845	390174	40
3612	line*coccidia	1	3	1171596	390174	40
3612	line*coccidia	2	0	235701.3	390174	40
3612	line*coccidia	2	1	416023.2	390174	40
3612	line*coccidia	2	2	178997	390174	40
3612	line*coccidia	2	3	215872.5	390174	40
3613	line	1		1407116	600954.4	40
3613	line	2		455151.9	600954.4	40

3613	coccidia		0	643383.4	849877.8	40
3613	coccidia		1	396768.7	849877.8	40
3613	coccidia		2	310461.3	849877.8	40
3613	coccidia		3	2373923	849877.8	40
3613	line*coccidia	1	0	924996	1201909	40
3613	line*coccidia	1	1	416932.5	1201909	40
3613	line*coccidia	1	2	132523.8	1201909	40
3613	line*coccidia	1	3	4154012	1201909	40
3613	line*coccidia	2	0	361770.8	1201909	40
3613	line*coccidia	2	1	376604.8	1201909	40
3613	line*coccidia	2	2	488398.7	1201909	40
3613	line*coccidia	2	3	593833.2	1201909	40
3625	line	1		252171.5	117264.8	40
3625	line	2		654444.4	117264.8	40
3625	coccidia		0	472125.3	165837.4	40
3625	coccidia		1	297336.2	165837.4	40
3625	coccidia		2	704587.6	165837.4	40
3625	coccidia		3	339182.8	165837.4	40
3625	line*coccidia	1	0	451680.3	234529.6	40
3625	line*coccidia	1	1	122426.2	234529.6	40
3625	line*coccidia	1	2	118011.2	234529.6	40
3625	line*coccidia	1	3	316568.3	234529.6	40
3625	line*coccidia	2	0	492570.2	234529.6	40
3625	line*coccidia	2	1	472246.2	234529.6	40
3625	line*coccidia	2	2	1291164	234529.6	40
3625	line*coccidia	2	3	361797.2	234529.6	40
3626	line	1		231556.1	90922.25	40
3626	line	2		286475.3	90922.25	40
3626	coccidia		0	225943.8	128583.5	40
3626	coccidia		1	226656.4	128583.5	40
3626	coccidia		2	306590	128583.5	40
3626	coccidia		3	276872.6	128583.5	40
3626	line*coccidia	1	0	314660.7	181844.5	40
3626	line*coccidia	1	1	111488.7	181844.5	40
3626	line*coccidia	1	2	61789.83	181844.5	40
3626	line*coccidia	1	3	438285.3	181844.5	40
3626	line*coccidia	2	0	137227	181844.5	40
3626	line*coccidia	2	1	341824.2	181844.5	40
3626	line*coccidia	2	2	551390.2	181844.5	40
3626	line*coccidia	2	3	115459.8	181844.5	40
3627	line	1		848654.9	205202.8	40
3627	line	2		740383.8	205202.8	40
3627	coccidia		0	583352.2	290200.6	40
3627	coccidia		1	736169.8	290200.6	40
3627	coccidia		2	942856.7	290200.6	40
3627	coccidia		3	915698.8	290200.6	40
3627	line*coccidia	1	0	763969.5	410405.6	40
3627	line*coccidia	1	1	986495	410405.6	40
3627	line*coccidia	1	2	72584.17	410405.6	40

3627	line*coccidia	1	3	1571571	410405.6	40
3627	line*coccidia	2	0	402734.8	410405.6	40
3627	line*coccidia	2	1	485844.5	410405.6	40
3627	line*coccidia	2	2	1813129	410405.6	40
3627	line*coccidia	2	3	259826.8	410405.6	40
3629	line	1		1850223	695086.4	40
3629	line	2		682429.6	695086.4	40
3629	coccidia		0	857646.6	983000.6	40
3629	coccidia		1	396297.3	983000.6	40
3629	coccidia		2	569557.1	983000.6	40
3629	coccidia		3	3241803	983000.6	40
3629	line*coccidia	1	0	1269146	1390173	40
3629	line*coccidia	1	1	181951.8	1390173	40
3629	line*coccidia	1	2	301528.5	1390173	40
3629	line*coccidia	1	3	5648264	1390173	40
3629	line*coccidia	2	0	446147	1390173	40
3629	line*coccidia	2	1	610642.8	1390173	40
3629	line*coccidia	2	2	837585.7	1390173	40
3629	line*coccidia	2	3	835342.8	1390173	40
3630	line	1		600385.1	153668.6	40
3630	line	2		807887.1	153668.6	40
3630	coccidia		0	781151.1	217320.2	40
3630	coccidia		1	637194.7	217320.2	40
3630	coccidia		2	530665.9	217320.2	40
3630	coccidia		3	867532.7	217320.2	40
3630	line*coccidia	1	0	504447.7	307337.2	40
3630	line*coccidia	1	1	327252.2	307337.2	40
3630	line*coccidia	1	2	392291.8	307337.2	40
3630	line*coccidia	1	3	1177549	307337.2	40
3630	line*coccidia	2	0	1057855	307337.2	40
3630	line*coccidia	2	1	947137.2	307337.2	40
3630	line*coccidia	2	2	669040	307337.2	40
3630	line*coccidia	2	3	557516.7	307337.2	40
3701	line	1		394244.2	49265.23	40
3701	line	2		328619.6	49265.23	40
3701	coccidia		0	454217.8	69671.55	40
3701	coccidia		1	427633.5	69671.55	40
3701	coccidia		2	245492.9	69671.55	40
3701	coccidia		3	318383.5	69671.55	40
3701	line*coccidia	1	0	630823.8	98530.45	40
3701	line*coccidia	1	1	345994	98530.45	40
3701	line*coccidia	1	2	181721.7	98530.45	40
3701	line*coccidia	1	3	418437.3	98530.45	40
3701	line*coccidia	2	0	277611.7	98530.45	40
3701	line*coccidia	2	1	509273	98530.45	40
3701	line*coccidia	2	2	309264.2	98530.45	40
3701	line*coccidia	2	3	218329.7	98530.45	40
3702	line	1		947928	183608.8	40
3702	line	2		634506.7	183608.8	40

3702	coccidia		0	620724	259662.1	40
3702	coccidia		1	938183.6	259662.1	40
3702	coccidia		2	305275.3	259662.1	40
3702	coccidia		3	1300687	259662.1	40
3702	line*coccidia	1	0	765441.2	367217.7	40
3702	line*coccidia	1	1	793186.3	367217.7	40
3702	line*coccidia	1	2	156371.5	367217.7	40
3702	line*coccidia	1	3	2076713	367217.7	40
3702	line*coccidia	2	0	476006.8	367217.7	40
3702	line*coccidia	2	1	1083181	367217.7	40
3702	line*coccidia	2	2	454179	367217.7	40
3702	line*coccidia	2	3	524660	367217.7	40
3703	line	1		725941.3	134167.6	40
3703	line	2		786476.9	134167.6	40
3703	coccidia		0	713332	189741.6	40
3703	coccidia		1	1083165	189741.6	40
3703	coccidia		2	417638.5	189741.6	40
3703	coccidia		3	810701.1	189741.6	40
3703	line*coccidia	1	0	898652.8	268335.2	40
3703	line*coccidia	1	1	1080924	268335.2	40
3703	line*coccidia	1	2	207506.8	268335.2	40
3703	line*coccidia	1	3	716682	268335.2	40
3703	line*coccidia	2	0	528011.2	268335.2	40
3703	line*coccidia	2	1	1085406	268335.2	40
3703	line*coccidia	2	2	627770.2	268335.2	40
3703	line*coccidia	2	3	904720.2	268335.2	40
3706	line	1		1040372	311665.2	40
3706	line	2		592262.1	311665.2	40
3706	coccidia		0	1613702	440761.1	40
3706	coccidia		1	551685.8	440761.1	40
3706	coccidia		2	215702.4	440761.1	40
3706	coccidia		3	884178.2	440761.1	40
3706	line*coccidia	1	0	2869175	623330.4	40
3706	line*coccidia	1	1	448125.3	623330.4	40
3706	line*coccidia	1	2	177373.2	623330.4	40
3706	line*coccidia	1	3	666814.8	623330.4	40
3706	line*coccidia	2	0	358229	623330.4	40
3706	line*coccidia	2	1	655246.3	623330.4	40
3706	line*coccidia	2	2	254031.7	623330.4	40
3706	line*coccidia	2	3	1101542	623330.4	40
3707	line	1		444522.5	76149.14	40
3707	line	2		359472.4	76149.14	40
3707	coccidia		0	378849	107691.1	40
3707	coccidia		1	575155.3	107691.1	40
3707	coccidia		2	212794.3	107691.1	40
3707	coccidia		3	441191.3	107691.1	40
3707	line*coccidia	1	0	528852.5	152298.3	40
3707	line*coccidia	1	1	462466.5	152298.3	40
3707	line*coccidia	1	2	220449.2	152298.3	40

3707	line*coccidia	1	3	566322	152298.3	40
3707	line*coccidia	2	0	228845.5	152298.3	40
3707	line*coccidia	2	1	687844	152298.3	40
3707	line*coccidia	2	2	205139.5	152298.3	40
3707	line*coccidia	2	3	316060.7	152298.3	40
3710	line	1		510770	70792.92	40
3710	line	2		302206.5	70792.92	40
3710	coccidia		0	436474.9	100116.3	40
3710	coccidia		1	381416	100116.3	40
3710	coccidia		2	228201.8	100116.3	40
3710	coccidia		3	579860.3	100116.3	40
3710	line*coccidia	1	0	561758.2	141585.8	40
3710	line*coccidia	1	1	303290	141585.8	40
3710	line*coccidia	1	2	215332.5	141585.8	40
3710	line*coccidia	1	3	962699.5	141585.8	40
3710	line*coccidia	2	0	311191.7	141585.8	40
3710	line*coccidia	2	1	459542	141585.8	40
3710	line*coccidia	2	2	241071.2	141585.8	40
3710	line*coccidia	2	3	197021	141585.8	40
3711	line	1		330233.5	120034.9	40
3711	line	2		256719.7	120034.9	40
3711	coccidia		0	219361.3	169755	40
3711	coccidia		1	145953	169755	40
3711	coccidia		2	431850.8	169755	40
3711	coccidia		3	376741.3	169755	40
3711	line*coccidia	1	0	174750.3	240069.9	40
3711	line*coccidia	1	1	116933.2	240069.9	40
3711	line*coccidia	1	2	305842.2	240069.9	40
3711	line*coccidia	1	3	723408.3	240069.9	40
3711	line*coccidia	2	0	263972.3	240069.9	40
3711	line*coccidia	2	1	174972.8	240069.9	40
3711	line*coccidia	2	2	557859.5	240069.9	40
3711	line*coccidia	2	3	30074.17	240069.9	40
3712	line	1		1191839	503813.5	40
3712	line	2		1073531	503813.5	40
3712	coccidia		0	909810.4	712499.9	40
3712	coccidia		1	519547.1	712499.9	40
3712	coccidia		2	1723355	712499.9	40
3712	coccidia		3	1378027	712499.9	40
3712	line*coccidia	1	0	1311764	1007627	40
3712	line*coccidia	1	1	417496.2	1007627	40
3712	line*coccidia	1	2	474171.5	1007627	40
3712	line*coccidia	1	3	2563923	1007627	40
3712	line*coccidia	2	0	507856.8	1007627	40
3712	line*coccidia	2	1	621598	1007627	40
3712	line*coccidia	2	2	2972538	1007627	40
3712	line*coccidia	2	3	192129.8	1007627	40
3715	line	1		352696	131772.4	40
3715	line	2		526712.8	131772.4	40

3715	coccidia		0	615209.9	186354.4	40
3715	coccidia		1	339801.2	186354.4	40
3715	coccidia		2	351313.2	186354.4	40
3715	coccidia		3	452493.5	186354.4	40
3715	line*coccidia	1	0	436304.3	263544.9	40
3715	line*coccidia	1	1	181813	263544.9	40
3715	line*coccidia	1	2	46720	263544.9	40
3715	line*coccidia	1	3	745946.8	263544.9	40
3715	line*coccidia	2	0	794115.5	263544.9	40
3715	line*coccidia	2	1	497789.3	263544.9	40
3715	line*coccidia	2	2	655906.3	263544.9	40
3715	line*coccidia	2	3	159040.2	263544.9	40
3716	line	1		1495385	368220.8	40
3716	line	2		1323624	368220.8	40
3716	coccidia		0	1505152	520742.9	40
3716	coccidia		1	1062205	520742.9	40
3716	coccidia		2	1884304	520742.9	40
3716	coccidia		3	1186358	520742.9	40
3716	line*coccidia	1	0	1653115	736441.7	40
3716	line*coccidia	1	1	1205427	736441.7	40
3716	line*coccidia	1	2	943835.2	736441.7	40
3716	line*coccidia	1	3	2179164	736441.7	40
3716	line*coccidia	2	0	1357188	736441.7	40
3716	line*coccidia	2	1	918984.2	736441.7	40
3716	line*coccidia	2	2	2824772	736441.7	40
3716	line*coccidia	2	3	193551	736441.7	40
3717	line	1		248665.1	126068.4	40
3717	line	2		454682.3	126068.4	40
3717	coccidia		0	373352.2	178287.6	40
3717	coccidia		1	201276.3	178287.6	40
3717	coccidia		2	470940.3	178287.6	40
3717	coccidia		3	361126.3	178287.6	40
3717	line*coccidia	1	0	136142.7	252136.8	40
3717	line*coccidia	1	1	98477.67	252136.8	40
3717	line*coccidia	1	2	98451.5	252136.8	40
3717	line*coccidia	1	3	661588.7	252136.8	40
3717	line*coccidia	2	0	610561.7	252136.8	40
3717	line*coccidia	2	1	304074.8	252136.8	40
3717	line*coccidia	2	2	843429	252136.8	40
3717	line*coccidia	2	3	60663.83	252136.8	40
3718	line	1		942276.5	173188.5	40
3718	line	2		861741.5	173188.5	40
3718	coccidia		0	898301.4	244925.5	40
3718	coccidia		1	497683.3	244925.5	40
3718	coccidia		2	1095299	244925.5	40
3718	coccidia		3	1116752	244925.5	40
3718	line*coccidia	1	0	741919	346376.9	40
3718	line*coccidia	1	1	326575.2	346376.9	40
3718	line*coccidia	1	2	779344	346376.9	40

3718	line*coccidia	1	3	1921268	346376.9	40
3718	line*coccidia	2	0	1054684	346376.9	40
3718	line*coccidia	2	1	668791.5	346376.9	40
3718	line*coccidia	2	2	1411255	346376.9	40
3718	line*coccidia	2	3	312236.2	346376.9	40
3719	line	1		322111.5	437139.7	40
3719	line	2		1441514	437139.7	40
3719	coccidia		0	1134331	618208.9	40
3719	coccidia		1	381880.1	618208.9	40
3719	coccidia		2	1581484	618208.9	40
3719	coccidia		3	429556.3	618208.9	40
3719	line*coccidia	1	0	296299.5	874279.5	40
3719	line*coccidia	1	1	121914.8	874279.5	40
3719	line*coccidia	1	2	160324.8	874279.5	40
3719	line*coccidia	1	3	709907	874279.5	40
3719	line*coccidia	2	0	1972363	874279.5	40
3719	line*coccidia	2	1	641845.3	874279.5	40
3719	line*coccidia	2	2	3002644	874279.5	40
3719	line*coccidia	2	3	149205.7	874279.5	40
3720	line	1		1675885	875692.6	40
3720	line	2		2791606	875692.6	40
3720	coccidia		0	1113600	1238416	40
3720	coccidia		1	2985902	1238416	40
3720	coccidia		2	1398838	1238416	40
3720	coccidia		3	3436641	1238416	40
3720	line*coccidia	1	0	272242.2	1751385	40
3720	line*coccidia	1	1	1811271	1751385	40
3720	line*coccidia	1	2	2051155	1751385	40
3720	line*coccidia	1	3	2568872	1751385	40
3720	line*coccidia	2	0	1954958	1751385	40
3720	line*coccidia	2	1	4160533	1751385	40
3720	line*coccidia	2	2	746519.8	1751385	40
3720	line*coccidia	2	3	4304411	1751385	40
3727	line	1		1449219	638705.5	40
3727	line	2		274636.5	638705.5	40
3727	coccidia		0	695343	903265.9	40
3727	coccidia		1	2038395	903265.9	40
3727	coccidia		2	547986.1	903265.9	40
3727	coccidia		3	165987.3	903265.9	40
3727	line*coccidia	1	0	928961	1277411	40
3727	line*coccidia	1	1	3707585	1277411	40
3727	line*coccidia	1	2	886843.7	1277411	40
3727	line*coccidia	1	3	273487.8	1277411	40
3727	line*coccidia	2	0	461725	1277411	40
3727	line*coccidia	2	1	369205.7	1277411	40
3727	line*coccidia	2	2	209128.5	1277411	40
3727	line*coccidia	2	3	58486.83	1277411	40
3730	line	1		1316169	306166	40
3730	line	2		1043116	306166	40

3730	coccidia		0	745877.9	432984.2	40
3730	coccidia		1	1314255	432984.2	40
3730	coccidia		2	1559115	432984.2	40
3730	coccidia		3	1099320	432984.2	40
3730	line*coccidia	1	0	1054069	612332.1	40
3730	line*coccidia	1	1	1224149	612332.1	40
3730	line*coccidia	1	2	1450461	612332.1	40
3730	line*coccidia	1	3	1535995	612332.1	40
3730	line*coccidia	2	0	437686.8	612332.1	40
3730	line*coccidia	2	1	1404361	612332.1	40
3730	line*coccidia	2	2	1667769	612332.1	40
3730	line*coccidia	2	3	662646	612332.1	40
3732	line	1		2425573	1082604	40
3732	line	2		214877.3	1082604	40
3732	coccidia		0	3130053	1531033	40
3732	coccidia		1	483752.2	1531033	40
3732	coccidia		2	1193803	1531033	40
3732	coccidia		3	473291.8	1531033	40
3732	line*coccidia	1	0	6100506	2165208	40
3732	line*coccidia	1	1	700105.7	2165208	40
3732	line*coccidia	1	2	2077918	2165208	40
3732	line*coccidia	1	3	823762	2165208	40
3732	line*coccidia	2	0	159600.2	2165208	40
3732	line*coccidia	2	1	267398.7	2165208	40
3732	line*coccidia	2	2	309689	2165208	40
3732	line*coccidia	2	3	122821.5	2165208	40
3733	line	1		1461613	408427.1	40
3733	line	2		2399978	408427.1	40
3733	coccidia		0	1574288	577603.1	40
3733	coccidia		1	1290786	577603.1	40
3733	coccidia		2	2455876	577603.1	40
3733	coccidia		3	2402231	577603.1	40
3733	line*coccidia	1	0	259711	816854.1	40
3733	line*coccidia	1	1	1052097	816854.1	40
3733	line*coccidia	1	2	1016894	816854.1	40
3733	line*coccidia	1	3	3517748	816854.1	40
3733	line*coccidia	2	0	2888864	816854.1	40
3733	line*coccidia	2	1	1529475	816854.1	40
3733	line*coccidia	2	2	3894857	816854.1	40
3733	line*coccidia	2	3	1286714	816854.1	40
3803	line	1		2452450	1113866	40
3803	line	2		741155.2	1113866	40
3803	coccidia		0	3902300	1575244	40
3803	coccidia		1	819598	1575244	40
3803	coccidia		2	1195193	1575244	40
3803	coccidia		3	470120.4	1575244	40
3803	line*coccidia	1	0	6501856	2227732	40
3803	line*coccidia	1	1	791024	2227732	40
3803	line*coccidia	1	2	2029892	2227732	40

3803	line*coccidia	1	3	487029.3	2227732	40
3803	line*coccidia	2	0	1302744	2227732	40
3803	line*coccidia	2	1	848172	2227732	40
3803	line*coccidia	2	2	360493.8	2227732	40
3803	line*coccidia	2	3	453211.5	2227732	40
3816	line	1		1246403	549165.7	40
3816	line	2		2231611	549165.7	40
3816	coccidia		0	1333791	776637.6	40
3816	coccidia		1	2033078	776637.6	40
3816	coccidia		2	2026158	776637.6	40
3816	coccidia		3	1563002	776637.6	40
3816	line*coccidia	1	0	188682	1098331	40
3816	line*coccidia	1	1	2948526	1098331	40
3816	line*coccidia	1	2	581291.3	1098331	40
3816	line*coccidia	1	3	1267115	1098331	40
3816	line*coccidia	2	0	2478901	1098331	40
3816	line*coccidia	2	1	1117630	1098331	40
3816	line*coccidia	2	2	3471025	1098331	40
3816	line*coccidia	2	3	1858890	1098331	40
3822	line	1		3191915	1327720	40
3822	line	2		533526.6	1327720	40
3822	coccidia		0	852022.4	1877679	40
3822	coccidia		1	3856817	1877679	40
3822	coccidia		2	1068138	1877679	40
3822	coccidia		3	1673906	1877679	40
3822	line*coccidia	1	0	735340.8	2655440	40
3822	line*coccidia	1	1	7416036	2655440	40
3822	line*coccidia	1	2	1715335	2655440	40
3822	line*coccidia	1	3	2900950	2655440	40
3822	line*coccidia	2	0	968704	2655440	40
3822	line*coccidia	2	1	297598.3	2655440	40
3822	line*coccidia	2	2	420941.5	2655440	40
3822	line*coccidia	2	3	446862.5	2655440	40
3827	line	1		742780.7	311230	40
3827	line	2		1779910	311230	40
3827	coccidia		0	939858.4	440145.7	40
3827	coccidia		1	1348553	440145.7	40
3827	coccidia		2	906540.2	440145.7	40
3827	coccidia		3	1850430	440145.7	40
3827	line*coccidia	1	0	252053.8	622460	40
3827	line*coccidia	1	1	67595.17	622460	40
3827	line*coccidia	1	2	1484357	622460	40
3827	line*coccidia	1	3	1167117	622460	40
3827	line*coccidia	2	0	1627663	622460	40
3827	line*coccidia	2	1	2629511	622460	40
3827	line*coccidia	2	2	328723.3	622460	40
3827	line*coccidia	2	3	2533744	622460	40
3828	line	1		3105136	1379545	40
3828	line	2		965035.7	1379545	40

3828	coccidia		0	1742062	1950972	40
3828	coccidia		1	891402.4	1950972	40
3828	coccidia		2	3908937	1950972	40
3828	coccidia		3	1597944	1950972	40
3828	line*coccidia	1	0	1870410	2759091	40
3828	line*coccidia	1	1	458787.7	2759091	40
3828	line*coccidia	1	2	7567925	2759091	40
3828	line*coccidia	1	3	2523423	2759091	40
3828	line*coccidia	2	0	1613713	2759091	40
3828	line*coccidia	2	1	1324017	2759091	40
3828	line*coccidia	2	2	249948.2	2759091	40
3828	line*coccidia	2	3	672464.2	2759091	40
3833	line	1		1174653	584251.5	40
3833	line	2		2793278	584251.5	40
3833	coccidia		0	1432919	826256.4	40
3833	coccidia		1	1812195	826256.4	40
3833	coccidia		2	1775723	826256.4	40
3833	coccidia		3	2915025	826256.4	40
3833	line*coccidia	1	0	437736.3	1168503	40
3833	line*coccidia	1	1	224672.8	1168503	40
3833	line*coccidia	1	2	907248.2	1168503	40
3833	line*coccidia	1	3	3128953	1168503	40
3833	line*coccidia	2	0	2428103	1168503	40
3833	line*coccidia	2	1	3399716	1168503	40
3833	line*coccidia	2	2	2644198	1168503	40
3833	line*coccidia	2	3	2701096	1168503	40
3845	line	1		3815848	1192939	40
3845	line	2		1502252	1192939	40
3845	coccidia		0	4426140	1687070	40
3845	coccidia		1	1293042	1687070	40
3845	coccidia		2	3814263	1687070	40
3845	coccidia		3	1102754	1687070	40
3845	line*coccidia	1	0	5160164	2385877	40
3845	line*coccidia	1	1	2135125	2385877	40
3845	line*coccidia	1	2	6582486	2385877	40
3845	line*coccidia	1	3	1385617	2385877	40
3845	line*coccidia	2	0	3692115	2385877	40
3845	line*coccidia	2	1	450959.7	2385877	40
3845	line*coccidia	2	2	1046040	2385877	40
3845	line*coccidia	2	3	819891.7	2385877	40
3849	line	1		724566.5	231849.4	40
3849	line	2		110649.1	231849.4	40
3849	coccidia		0	199563.7	327884.6	40
3849	coccidia		1	271398.2	327884.6	40
3849	coccidia		2	119202.6	327884.6	40
3849	coccidia		3	1080267	327884.6	40
3849	line*coccidia	1	0	276228.5	463698.8	40
3849	line*coccidia	1	1	464266.2	463698.8	40
3849	line*coccidia	1	2	105825.5	463698.8	40

3849	line*coccidia	1	3	2051946	463698.8	40
3849	line*coccidia	2	0	122898.8	463698.8	40
3849	line*coccidia	2	1	78530.17	463698.8	40
3849	line*coccidia	2	2	132579.7	463698.8	40
3849	line*coccidia	2	3	108587.8	463698.8	40
3851	line	1		135269.2	48693.21	40
3851	line	2		173766	48693.21	40
3851	coccidia		0	100665.4	68862.6	40
3851	coccidia		1	136456.9	68862.6	40
3851	coccidia		2	190196.5	68862.6	40
3851	coccidia		3	190751.6	68862.6	40
3851	line*coccidia	1	0	63424.83	97386.42	40
3851	line*coccidia	1	1	91811.67	97386.42	40
3851	line*coccidia	1	2	118828.8	97386.42	40
3851	line*coccidia	1	3	267011.5	97386.42	40
3851	line*coccidia	2	0	137906	97386.42	40
3851	line*coccidia	2	1	181102.2	97386.42	40
3851	line*coccidia	2	2	261564.2	97386.42	40
3851	line*coccidia	2	3	114491.7	97386.42	40
3852	line	1		129457.7	108665	40
3852	line	2		654316.5	108665	40
3852	coccidia		0	265017.5	153675.5	40
3852	coccidia		1	434958.3	153675.5	40
3852	coccidia		2	406031.5	153675.5	40
3852	coccidia		3	461541	153675.5	40
3852	line*coccidia	1	0	123693.8	217330	40
3852	line*coccidia	1	1	242543.7	217330	40
3852	line*coccidia	1	2	106093.7	217330	40
3852	line*coccidia	1	3	45499.67	217330	40
3852	line*coccidia	2	0	406341.2	217330	40
3852	line*coccidia	2	1	627373	217330	40
3852	line*coccidia	2	2	705969.3	217330	40
3852	line*coccidia	2	3	877582.3	217330	40
3855	line	1		533521.3	704976.8	40
3855	line	2		2164887	704976.8	40
3855	coccidia		0	2072748	996987.7	40
3855	coccidia		1	729991.1	996987.7	40
3855	coccidia		2	843512.6	996987.7	40
3855	coccidia		3	1750564	996987.7	40
3855	line*coccidia	1	0	302029.7	1409954	40
3855	line*coccidia	1	1	562830.7	1409954	40
3855	line*coccidia	1	2	993862.3	1409954	40
3855	line*coccidia	1	3	275362.3	1409954	40
3855	line*coccidia	2	0	3843466	1409954	40
3855	line*coccidia	2	1	897151.5	1409954	40
3855	line*coccidia	2	2	693162.8	1409954	40
3855	line*coccidia	2	3	3225766	1409954	40
3856	line	1		437712.9	260047.8	40
3856	line	2		612260.3	260047.8	40

3856	coccidia		0	307990.8	367763.2	40
3856	coccidia		1	1053428	367763.2	40
3856	coccidia		2	394296.7	367763.2	40
3856	coccidia		3	344230.7	367763.2	40
3856	line*coccidia	1	0	398263.8	520095.7	40
3856	line*coccidia	1	1	462883	520095.7	40
3856	line*coccidia	1	2	606766.7	520095.7	40
3856	line*coccidia	1	3	282938	520095.7	40
3856	line*coccidia	2	0	217717.7	520095.7	40
3856	line*coccidia	2	1	1643974	520095.7	40
3856	line*coccidia	2	2	181826.7	520095.7	40
3856	line*coccidia	2	3	405523.3	520095.7	40
3857	line	1		174628.3	94197.52	40
3857	line	2		367117.8	94197.52	40
3857	coccidia		0	374366.2	133215.4	40
3857	coccidia		1	232912.9	133215.4	40
3857	coccidia		2	280832.3	133215.4	40
3857	coccidia		3	195380.8	133215.4	40
3857	line*coccidia	1	0	212150.2	188395	40
3857	line*coccidia	1	1	101078.7	188395	40
3857	line*coccidia	1	2	159529.5	188395	40
3857	line*coccidia	1	3	225754.7	188395	40
3857	line*coccidia	2	0	536582.2	188395	40
3857	line*coccidia	2	1	364747.2	188395	40
3857	line*coccidia	2	2	402135.2	188395	40
3857	line*coccidia	2	3	165006.8	188395	40
3858	line	1		330605.8	101617	40
3858	line	2		413722.7	101617	40
3858	coccidia		0	255836.7	143708.1	40
3858	coccidia		1	470191.7	143708.1	40
3858	coccidia		2	275056.8	143708.1	40
3858	coccidia		3	487571.8	143708.1	40
3858	line*coccidia	1	0	424776.5	203234	40
3858	line*coccidia	1	1	426326	203234	40
3858	line*coccidia	1	2	137748.3	203234	40
3858	line*coccidia	1	3	333572.2	203234	40
3858	line*coccidia	2	0	86896.83	203234	40
3858	line*coccidia	2	1	514057.3	203234	40
3858	line*coccidia	2	2	412365.3	203234	40
3858	line*coccidia	2	3	641571.3	203234	40
3859	line	1		659707	594701.7	40
3859	line	2		1379704	594701.7	40
3859	coccidia		0	431579.2	841035.2	40
3859	coccidia		1	2080211	841035.2	40
3859	coccidia		2	366697.9	841035.2	40
3859	coccidia		3	1200333	841035.2	40
3859	line*coccidia	1	0	477928.5	1189403	40
3859	line*coccidia	1	1	676504.5	1189403	40
3859	line*coccidia	1	2	525306	1189403	40

3859	line*coccidia	1	3	959089	1189403	40
3859	line*coccidia	2	0	385229.8	1189403	40
3859	line*coccidia	2	1	3483918	1189403	40
3859	line*coccidia	2	2	208089.8	1189403	40
3859	line*coccidia	2	3	1441577	1189403	40
3860	line	1		496592.1	143850.2	40
3860	line	2		422222.6	143850.2	40
3860	coccidia		0	358360.4	203434.9	40
3860	coccidia		1	148898.3	203434.9	40
3860	coccidia		2	900826	203434.9	40
3860	coccidia		3	429544.8	203434.9	40
3860	line*coccidia	1	0	599883.8	287700.3	40
3860	line*coccidia	1	1	207995	287700.3	40
3860	line*coccidia	1	2	662176.8	287700.3	40
3860	line*coccidia	1	3	516312.8	287700.3	40
3860	line*coccidia	2	0	116837	287700.3	40
3860	line*coccidia	2	1	89801.5	287700.3	40
3860	line*coccidia	2	2	1139475	287700.3	40
3860	line*coccidia	2	3	342776.8	287700.3	40
3861	line	1		377669	718924.4	40
3861	line	2		1452962	718924.4	40
3861	coccidia		0	386735.9	1016713	40
3861	coccidia		1	380349.3	1016713	40
3861	coccidia		2	2144868	1016713	40
3861	coccidia		3	749308.8	1016713	40
3861	line*coccidia	1	0	298804.8	1437849	40
3861	line*coccidia	1	1	507424.3	1437849	40
3861	line*coccidia	1	2	234628.8	1437849	40
3861	line*coccidia	1	3	469817.8	1437849	40
3861	line*coccidia	2	0	474667	1437849	40
3861	line*coccidia	2	1	253274.2	1437849	40
3861	line*coccidia	2	2	4055106	1437849	40
3861	line*coccidia	2	3	1028800	1437849	40
3862	line	1		429001.4	225537.3	40
3862	line	2		565127.3	225537.3	40
3862	coccidia		0	490329.3	318957.9	40
3862	coccidia		1	401365.6	318957.9	40
3862	coccidia		2	347648.5	318957.9	40
3862	coccidia		3	748914.1	318957.9	40
3862	line*coccidia	1	0	586566.5	451074.5	40
3862	line*coccidia	1	1	695157.3	451074.5	40
3862	line*coccidia	1	2	254617.3	451074.5	40
3862	line*coccidia	1	3	179664.5	451074.5	40
3862	line*coccidia	2	0	394092.2	451074.5	40
3862	line*coccidia	2	1	107573.8	451074.5	40
3862	line*coccidia	2	2	440679.7	451074.5	40
3862	line*coccidia	2	3	1318164	451074.5	40
3863	line	1		264995.5	210543.7	40
3863	line	2		561012.5	210543.7	40

3863	coccidia		0	270499.9	297753.7	40
3863	coccidia		1	271683.8	297753.7	40
3863	coccidia		2	925392.5	297753.7	40
3863	coccidia		3	184439.7	297753.7	40
3863	line*coccidia	1	0	138925.5	421087.3	40
3863	line*coccidia	1	1	307340.5	421087.3	40
3863	line*coccidia	1	2	345645.3	421087.3	40
3863	line*coccidia	1	3	268070.5	421087.3	40
3863	line*coccidia	2	0	402074.3	421087.3	40
3863	line*coccidia	2	1	236027.2	421087.3	40
3863	line*coccidia	2	2	1505140	421087.3	40
3863	line*coccidia	2	3	100808.8	421087.3	40
3910	line	1		346538.6	102471.1	39
3910	line	2		189965.3	100001.5	39
3910	coccidia		0	379649.2	141423.5	39
3910	coccidia		1	198606.5	148326.2	39
3910	coccidia		2	182186.5	141423.5	39
3910	coccidia		3	312565.6	141423.5	39
3910	line*coccidia	1	0	668316.8	200003	39
3910	line*coccidia	1	1	214213.6	219092.4	39
3910	line*coccidia	1	2	242419.2	200003	39
3910	line*coccidia	1	3	261204.7	200003	39
3910	line*coccidia	2	0	90981.5	200003	39
3910	line*coccidia	2	1	182999.3	200003	39
3910	line*coccidia	2	2	121953.8	200003	39
3910	line*coccidia	2	3	363926.5	200003	39
3916	line	1		1441603	591623.5	40
3916	line	2		2258196	591623.5	40
3916	coccidia		0	1220257	836681.9	40
3916	coccidia		1	3039355	836681.9	40
3916	coccidia		2	1057080	836681.9	40
3916	coccidia		3	2082907	836681.9	40
3916	line*coccidia	1	0	475826.7	1183247	40
3916	line*coccidia	1	1	2118921	1183247	40
3916	line*coccidia	1	2	1857978	1183247	40
3916	line*coccidia	1	3	1313688	1183247	40
3916	line*coccidia	2	0	1964688	1183247	40
3916	line*coccidia	2	1	3959789	1183247	40
3916	line*coccidia	2	2	256181.8	1183247	40
3916	line*coccidia	2	3	2852126	1183247	40
3917	line	1		215866.8	47714.15	40
3917	line	2		97303.63	47714.15	40
3917	coccidia		0	168859.5	67477.99	40
3917	coccidia		1	194572.8	67477.99	40
3917	coccidia		2	185598.4	67477.99	40
3917	coccidia		3	77310.17	67477.99	40
3917	line*coccidia	1	0	160399.3	95428.29	40
3917	line*coccidia	1	1	214989.7	95428.29	40
3917	line*coccidia	1	2	352994.2	95428.29	40

3917	line*coccidia	1	3	135084.2	95428.29	40
3917	line*coccidia	2	0	177319.7	95428.29	40
3917	line*coccidia	2	1	174156	95428.29	40
3917	line*coccidia	2	2	18202.67	95428.29	40
3917	line*coccidia	2	3	19536.17	95428.29	40
3918	line	1		675175	149562.1	40
3918	line	2		210509.5	149562.1	40
3918	coccidia		0	451377.8	211512.8	40
3918	coccidia		1	488624.3	211512.8	40
3918	coccidia		2	384738	211512.8	40
3918	coccidia		3	446628.8	211512.8	40
3918	line*coccidia	1	0	873751.7	299124.2	40
3918	line*coccidia	1	1	877962.3	299124.2	40
3918	line*coccidia	1	2	208918.3	299124.2	40
3918	line*coccidia	1	3	740067.5	299124.2	40
3918	line*coccidia	2	0	29004	299124.2	40
3918	line*coccidia	2	1	99286.17	299124.2	40
3918	line*coccidia	2	2	560557.7	299124.2	40
3918	line*coccidia	2	3	153190.2	299124.2	40
3919	line	1		724746.9	370036.7	40
3919	line	2		127100.2	370036.7	40
3919	coccidia		0	1135987	523311	40
3919	coccidia		1	211308.9	523311	40
3919	coccidia		2	102218.4	523311	40
3919	coccidia		3	254180.3	523311	40
3919	line*coccidia	1	0	2139698	740073.4	40
3919	line*coccidia	1	1	256769.8	740073.4	40
3919	line*coccidia	1	2	51486.5	740073.4	40
3919	line*coccidia	1	3	451032.8	740073.4	40
3919	line*coccidia	2	0	132274.7	740073.4	40
3919	line*coccidia	2	1	165848	740073.4	40
3919	line*coccidia	2	2	152950.3	740073.4	40
3919	line*coccidia	2	3	57327.67	740073.4	40
3920	line	1		510708.8	156918.6	40
3920	line	2		1087792	156918.6	40
3920	coccidia		0	748777.8	221916.4	40
3920	coccidia		1	715449.4	221916.4	40
3920	coccidia		2	994921	221916.4	40
3920	coccidia		3	737853.2	221916.4	40
3920	line*coccidia	1	0	58843.67	313837.2	40
3920	line*coccidia	1	1	684096.7	313837.2	40
3920	line*coccidia	1	2	312844	313837.2	40
3920	line*coccidia	1	3	987050.8	313837.2	40
3920	line*coccidia	2	0	1438712	313837.2	40
3920	line*coccidia	2	1	746802.2	313837.2	40
3920	line*coccidia	2	2	1676998	313837.2	40
3920	line*coccidia	2	3	488655.5	313837.2	40
3921	line	1		923394.7	464347.7	40
3921	line	2		213912.6	464347.7	40

3921	coccidia		0	1568010	656686.8	40
3921	coccidia		1	310732.8	656686.8	40
3921	coccidia		2	262800.8	656686.8	40
3921	coccidia		3	133070.9	656686.8	40
3921	line*coccidia	1	0	2882177	928695.3	40
3921	line*coccidia	1	1	233837	928695.3	40
3921	line*coccidia	1	2	462778.2	928695.3	40
3921	line*coccidia	1	3	114787	928695.3	40
3921	line*coccidia	2	0	253843.7	928695.3	40
3921	line*coccidia	2	1	387628.5	928695.3	40
3921	line*coccidia	2	2	62823.5	928695.3	40
3921	line*coccidia	2	3	151354.8	928695.3	40
3922	line	1		287482.9	94827.49	40
3922	line	2		848241.5	94827.49	40
3922	coccidia		0	433887.6	134106.3	40
3922	coccidia		1	432337.5	134106.3	40
3922	coccidia		2	426708.7	134106.3	40
3922	coccidia		3	978515.1	134106.3	40
3922	line*coccidia	1	0	40827.33	189655	40
3922	line*coccidia	1	1	481153.7	189655	40
3922	line*coccidia	1	2	196070.2	189655	40
3922	line*coccidia	1	3	431880.3	189655	40
3922	line*coccidia	2	0	826947.8	189655	40
3922	line*coccidia	2	1	383521.3	189655	40
3922	line*coccidia	2	2	657347.2	189655	40
3922	line*coccidia	2	3	1525150	189655	40
3923	line	1		1289499	777787.4	40
3923	line	2		185770	777787.4	40
3923	coccidia		0	234765.1	1099958	40
3923	coccidia		1	2363086	1099958	40
3923	coccidia		2	174649.4	1099958	40
3923	coccidia		3	178038.8	1099958	40
3923	line*coccidia	1	0	232249.5	1555575	40
3923	line*coccidia	1	1	4554020	1555575	40
3923	line*coccidia	1	2	190030	1555575	40
3923	line*coccidia	1	3	181698.5	1555575	40
3923	line*coccidia	2	0	237280.7	1555575	40
3923	line*coccidia	2	1	172151.5	1555575	40
3923	line*coccidia	2	2	159268.8	1555575	40
3923	line*coccidia	2	3	174379.2	1555575	40
3924	line	1		494558.8	141246	40
3924	line	2		927904.9	141246	40
3924	coccidia		0	393811.2	199752	40
3924	coccidia		1	722153.7	199752	40
3924	coccidia		2	965620.3	199752	40
3924	coccidia		3	763342.3	199752	40
3924	line*coccidia	1	0	49798.5	282491.9	40
3924	line*coccidia	1	1	28187.83	282491.9	40
3924	line*coccidia	1	2	1420796	282491.9	40

3924	line*coccidia	1	3	479453	282491.9	40
3924	line*coccidia	2	0	737823.8	282491.9	40
3924	line*coccidia	2	1	1416120	282491.9	40
3924	line*coccidia	2	2	510444.7	282491.9	40
3924	line*coccidia	2	3	1047232	282491.9	40
3925	line	1		1088777	454693.2	40
3925	line	2		191812.3	454693.2	40
3925	coccidia		0	737894.7	643033.4	40
3925	coccidia		1	241778.6	643033.4	40
3925	coccidia		2	1389887	643033.4	40
3925	coccidia		3	191617.3	643033.4	40
3925	line*coccidia	1	0	1346732	909386.5	40
3925	line*coccidia	1	1	125108	909386.5	40
3925	line*coccidia	1	2	2633293	909386.5	40
3925	line*coccidia	1	3	249973.3	909386.5	40
3925	line*coccidia	2	0	129057.3	909386.5	40
3925	line*coccidia	2	1	358449.2	909386.5	40
3925	line*coccidia	2	2	146481.3	909386.5	40
3925	line*coccidia	2	3	133261.2	909386.5	40
3926	line	1		221549.6	172949	40
3926	line	2		864138.6	172949	40
3926	coccidia		0	217220.5	244586.8	40
3926	coccidia		1	518213.7	244586.8	40
3926	coccidia		2	474933.7	244586.8	40
3926	coccidia		3	961008.6	244586.8	40
3926	line*coccidia	1	0	142330	345898	40
3926	line*coccidia	1	1	52364.5	345898	40
3926	line*coccidia	1	2	297522.8	345898	40
3926	line*coccidia	1	3	393981	345898	40
3926	line*coccidia	2	0	292111	345898	40
3926	line*coccidia	2	1	984062.8	345898	40
3926	line*coccidia	2	2	652344.5	345898	40
3926	line*coccidia	2	3	1528036	345898	40
4002	line	1		1247148	488624.4	40
4002	line	2		331523.8	488624.4	40
4002	coccidia		0	996786.5	691019.3	40
4002	coccidia		1	306970.5	691019.3	40
4002	coccidia		2	1631289	691019.3	40
4002	coccidia		3	222297.3	691019.3	40
4002	line*coccidia	1	0	1184457	977248.8	40
4002	line*coccidia	1	1	571398.8	977248.8	40
4002	line*coccidia	1	2	3023421	977248.8	40
4002	line*coccidia	1	3	209315	977248.8	40
4002	line*coccidia	2	0	809115.7	977248.8	40
4002	line*coccidia	2	1	42542.17	977248.8	40
4002	line*coccidia	2	2	239157.7	977248.8	40
4002	line*coccidia	2	3	235279.5	977248.8	40
4006	line	1		1980552	862490.3	40
4006	line	2		1257188	862490.3	40

4006	coccidia		0	407722.7	1219746	40
4006	coccidia		1	961347.7	1219746	40
4006	coccidia		2	894903.8	1219746	40
4006	coccidia		3	4211507	1219746	40
4006	line*coccidia	1	0	91179	1724981	40
4006	line*coccidia	1	1	202738.3	1724981	40
4006	line*coccidia	1	2	31866.33	1724981	40
4006	line*coccidia	1	3	7596425	1724981	40
4006	line*coccidia	2	0	724266.3	1724981	40
4006	line*coccidia	2	1	1719957	1724981	40
4006	line*coccidia	2	2	1757941	1724981	40
4006	line*coccidia	2	3	826589.3	1724981	40
4009	line	1		3498476	1267920	40
4009	line	2		806971.2	1267920	40
4009	coccidia		0	1004725	1793110	40
4009	coccidia		1	1723501	1793110	40
4009	coccidia		2	1845768	1793110	40
4009	coccidia		3	4036901	1793110	40
4009	line*coccidia	1	0	1318480	2535840	40
4009	line*coccidia	1	1	2583529	2535840	40
4009	line*coccidia	1	2	2156557	2535840	40
4009	line*coccidia	1	3	7935339	2535840	40
4009	line*coccidia	2	0	690970.5	2535840	40
4009	line*coccidia	2	1	863472.8	2535840	40
4009	line*coccidia	2	2	1534978	2535840	40
4009	line*coccidia	2	3	138463.7	2535840	40
4013	line	1		295434.8	603931.5	40
4013	line	2		3054715	603931.5	40
4013	coccidia		0	2367093	854088.1	40
4013	coccidia		1	857706.3	854088.1	40
4013	coccidia		2	906558.2	854088.1	40
4013	coccidia		3	2568942	854088.1	40
4013	line*coccidia	1	0	560851.3	1207863	40
4013	line*coccidia	1	1	418023.3	1207863	40
4013	line*coccidia	1	2	177107.7	1207863	40
4013	line*coccidia	1	3	25756.67	1207863	40
4013	line*coccidia	2	0	4173335	1207863	40
4013	line*coccidia	2	1	1297389	1207863	40
4013	line*coccidia	2	2	1636009	1207863	40
4013	line*coccidia	2	3	5112127	1207863	40
4101	line	1		1920353	1273480	40
4101	line	2		2805578	1273480	40
4101	coccidia		0	3828318	1800973	40
4101	coccidia		1	2303191	1800973	40
4101	coccidia		2	2095053	1800973	40
4101	coccidia		3	1225301	1800973	40
4101	line*coccidia	1	0	614711	2546961	40
4101	line*coccidia	1	1	2872271	2546961	40
4101	line*coccidia	1	2	3426291	2546961	40

4101	line*coccidia	1	3	768138.3	2546961	40
4101	line*coccidia	2	0	7041924	2546961	40
4101	line*coccidia	2	1	1734110	2546961	40
4101	line*coccidia	2	2	763814.2	2546961	40
4101	line*coccidia	2	3	1682463	2546961	40
4102	line	1		543563.2	753930.3	40
4102	line	2		3343402	753930.3	40
4102	coccidia		0	410979	1066218	40
4102	coccidia		1	1642612	1066218	40
4102	coccidia		2	1094690	1066218	40
4102	coccidia		3	4625650	1066218	40
4102	line*coccidia	1	0	403138.7	1507861	40
4102	line*coccidia	1	1	823980	1507861	40
4102	line*coccidia	1	2	751878.7	1507861	40
4102	line*coccidia	1	3	195255.7	1507861	40
4102	line*coccidia	2	0	418819.3	1507861	40
4102	line*coccidia	2	1	2461244	1507861	40
4102	line*coccidia	2	2	1437501	1507861	40
4102	line*coccidia	2	3	9056045	1507861	40
4103	line	1		4088747	1665803	40
4103	line	2		3873254	1665803	40
4103	coccidia		0	5623259	2355801	40
4103	coccidia		1	2257838	2355801	40
4103	coccidia		2	5020525	2355801	40
4103	coccidia		3	3022378	2355801	40
4103	line*coccidia	1	0	2946091	3331606	40
4103	line*coccidia	1	1	3128802	3331606	40
4103	line*coccidia	1	2	5089653	3331606	40
4103	line*coccidia	1	3	5190441	3331606	40
4103	line*coccidia	2	0	8300428	3331606	40
4103	line*coccidia	2	1	1386873	3331606	40
4103	line*coccidia	2	2	4951398	3331606	40
4103	line*coccidia	2	3	854315	3331606	40
4105	line	1		795386.1	616580.5	40
4105	line	2		2308657	616580.5	40
4105	coccidia		0	1032347	871976.4	40
4105	coccidia		1	1807723	871976.4	40
4105	coccidia		2	766459.4	871976.4	40
4105	coccidia		3	2601557	871976.4	40
4105	line*coccidia	1	0	1782374	1233161	40
4105	line*coccidia	1	1	863492	1233161	40
4105	line*coccidia	1	2	144218.2	1233161	40
4105	line*coccidia	1	3	391460.5	1233161	40
4105	line*coccidia	2	0	282319.7	1233161	40
4105	line*coccidia	2	1	2751953	1233161	40
4105	line*coccidia	2	2	1388701	1233161	40
4105	line*coccidia	2	3	4811653	1233161	40
4107	line	1		4371723	1065993	40
4107	line	2		2073499	1065993	40

4107	coccidia		0	2650979	1507542	40
4107	coccidia		1	3619174	1507542	40
4107	coccidia		2	1911222	1507542	40
4107	coccidia		3	4709069	1507542	40
4107	line*coccidia	1	0	4683458	2131986	40
4107	line*coccidia	1	1	2043459	2131986	40
4107	line*coccidia	1	2	2819043	2131986	40
4107	line*coccidia	1	3	7940931	2131986	40
4107	line*coccidia	2	0	618500.5	2131986	40
4107	line*coccidia	2	1	5194889	2131986	40
4107	line*coccidia	2	2	1003401	2131986	40
4107	line*coccidia	2	3	1477207	2131986	40
4108	line	1		655838.2	338946.1	40
4108	line	2		1352270	338946.1	40
4108	coccidia		0	866740.5	479342.2	40
4108	coccidia		1	113861	479342.2	40
4108	coccidia		2	1778735	479342.2	40
4108	coccidia		3	1256881	479342.2	40
4108	line*coccidia	1	0	1490088	677892.2	40
4108	line*coccidia	1	1	207589.2	677892.2	40
4108	line*coccidia	1	2	486473.8	677892.2	40
4108	line*coccidia	1	3	439202	677892.2	40
4108	line*coccidia	2	0	243393.2	677892.2	40
4108	line*coccidia	2	1	20132.83	677892.2	40
4108	line*coccidia	2	2	3070995	677892.2	40
4108	line*coccidia	2	3	2074559	677892.2	40
4109	line	1		1922884	908406.3	40
4109	line	2		2478569	908406.3	40
4109	coccidia		0	2010145	1284681	40
4109	coccidia		1	2008586	1284681	40
4109	coccidia		2	3298778	1284681	40
4109	coccidia		3	1485398	1284681	40
4109	line*coccidia	1	0	1646419	1816813	40
4109	line*coccidia	1	1	3420649	1816813	40
4109	line*coccidia	1	2	1764481	1816813	40
4109	line*coccidia	1	3	859985.3	1816813	40
4109	line*coccidia	2	0	2373871	1816813	40
4109	line*coccidia	2	1	596521.8	1816813	40
4109	line*coccidia	2	2	4833075	1816813	40
4109	line*coccidia	2	3	2110811	1816813	40
4110	line	1		801946.4	309185.4	40
4110	line	2		523385.9	309185.4	40
4110	coccidia		0	864591.3	437254.2	40
4110	coccidia		1	738734.8	437254.2	40
4110	coccidia		2	189692.8	437254.2	40
4110	coccidia		3	857645.6	437254.2	40
4110	line*coccidia	1	0	1416701	618370.8	40
4110	line*coccidia	1	1	1187661	618370.8	40
4110	line*coccidia	1	2	203117.5	618370.8	40

4110	line*coccidia	1	3	400306	618370.8	40
4110	line*coccidia	2	0	312481.5	618370.8	40
4110	line*coccidia	2	1	289808.7	618370.8	40
4110	line*coccidia	2	2	176268.2	618370.8	40
4110	line*coccidia	2	3	1314985	618370.8	40
4111	line	1		1324914	1016403	40
4111	line	2		2250330	1016403	40
4111	coccidia		0	1451966	1437411	40
4111	coccidia		1	1454801	1437411	40
4111	coccidia		2	3857072	1437411	40
4111	coccidia		3	386647.3	1437411	40
4111	line*coccidia	1	0	1274644	2032807	40
4111	line*coccidia	1	1	1998857	2032807	40
4111	line*coccidia	1	2	1620145	2032807	40
4111	line*coccidia	1	3	406008.5	2032807	40
4111	line*coccidia	2	0	1629288	2032807	40
4111	line*coccidia	2	1	910745.8	2032807	40
4111	line*coccidia	2	2	6093999	2032807	40
4111	line*coccidia	2	3	367286.2	2032807	40
4112	line	1		559006.7	204738.6	40
4112	line	2		492453.3	204738.6	40
4112	coccidia		0	384977.7	289544.1	40
4112	coccidia		1	364865.6	289544.1	40
4112	coccidia		2	490926.5	289544.1	40
4112	coccidia		3	862150.3	289544.1	40
4112	line*coccidia	1	0	547716.7	409477.1	40
4112	line*coccidia	1	1	394343.7	409477.1	40
4112	line*coccidia	1	2	794999.5	409477.1	40
4112	line*coccidia	1	3	498967	409477.1	40
4112	line*coccidia	2	0	222238.7	409477.1	40
4112	line*coccidia	2	1	335387.5	409477.1	40
4112	line*coccidia	2	2	186853.5	409477.1	40
4112	line*coccidia	2	3	1225334	409477.1	40
4114	line	1		795964.8	526834.5	40
4114	line	2		1684052	526834.5	40
4114	coccidia		0	843900	745056.5	40
4114	coccidia		1	1224522	745056.5	40
4114	coccidia		2	1014895	745056.5	40
4114	coccidia		3	1876717	745056.5	40
4114	line*coccidia	1	0	191977.2	1053669	40
4114	line*coccidia	1	1	762996.5	1053669	40
4114	line*coccidia	1	2	1751236	1053669	40
4114	line*coccidia	1	3	477649.5	1053669	40
4114	line*coccidia	2	0	1495823	1053669	40
4114	line*coccidia	2	1	1686048	1053669	40
4114	line*coccidia	2	2	278554.7	1053669	40
4114	line*coccidia	2	3	3275785	1053669	40
4115	line	1		909420.2	286282.9	40
4115	line	2		283433.7	286282.9	40

4115	coccidia		0	561158.6	404865.1	40
4115	coccidia		1	1105784	404865.1	40
4115	coccidia		2	540222.2	404865.1	40
4115	coccidia		3	178542.9	404865.1	40
4115	line*coccidia	1	0	859533.5	572565.7	40
4115	line*coccidia	1	1	1726156	572565.7	40
4115	line*coccidia	1	2	723838.5	572565.7	40
4115	line*coccidia	1	3	328153	572565.7	40
4115	line*coccidia	2	0	262783.7	572565.7	40
4115	line*coccidia	2	1	485412.5	572565.7	40
4115	line*coccidia	2	2	356605.8	572565.7	40
4115	line*coccidia	2	3	28932.83	572565.7	40
4116	line	1		1632830	444107.4	40
4116	line	2		2050742	444107.4	40
4116	coccidia		0	2196594	628062.7	40
4116	coccidia		1	1587342	628062.7	40
4116	coccidia		2	2379172	628062.7	40
4116	coccidia		3	1204035	628062.7	40
4116	line*coccidia	1	0	2062921	888214.8	40
4116	line*coccidia	1	1	927554.7	888214.8	40
4116	line*coccidia	1	2	1604815	888214.8	40
4116	line*coccidia	1	3	1936030	888214.8	40
4116	line*coccidia	2	0	2330268	888214.8	40
4116	line*coccidia	2	1	2247130	888214.8	40
4116	line*coccidia	2	2	3153530	888214.8	40
4116	line*coccidia	2	3	472039.2	888214.8	40
4117	line	1		2066302	931042.5	40
4117	line	2		559808	931042.5	40
4117	coccidia		0	3103324	1316693	40
4117	coccidia		1	596404.5	1316693	40
4117	coccidia		2	762701.9	1316693	40
4117	coccidia		3	789790.1	1316693	40
4117	line*coccidia	1	0	5610576	1862085	40
4117	line*coccidia	1	1	645640.2	1862085	40
4117	line*coccidia	1	2	670396.8	1862085	40
4117	line*coccidia	1	3	1338596	1862085	40
4117	line*coccidia	2	0	596072.3	1862085	40
4117	line*coccidia	2	1	547168.8	1862085	40
4117	line*coccidia	2	2	855007	1862085	40
4117	line*coccidia	2	3	240984	1862085	40
4118	line	1		1920578	491540.8	40
4118	line	2		2855182	491540.8	40
4118	coccidia		0	1965997	695143.7	40
4118	coccidia		1	2595501	695143.7	40
4118	coccidia		2	2534875	695143.7	40
4118	coccidia		3	2455147	695143.7	40
4118	line*coccidia	1	0	248311.2	983081.6	40
4118	line*coccidia	1	1	2633526	983081.6	40
4118	line*coccidia	1	2	1387304	983081.6	40

4118	line*coccidia	1	3	3413173	983081.6	40
4118	line*coccidia	2	0	3683683	983081.6	40
4118	line*coccidia	2	1	2557477	983081.6	40
4118	line*coccidia	2	2	3682446	983081.6	40
4118	line*coccidia	2	3	1497121	983081.6	40
4119	line	1		2248929	1142137	40
4119	line	2		480508.1	1142137	40
4119	coccidia		0	3794385	1615226	40
4119	coccidia		1	445652.5	1615226	40
4119	coccidia		2	921455	1615226	40
4119	coccidia		3	297380.8	1615226	40
4119	line*coccidia	1	0	6795416	2284275	40
4119	line*coccidia	1	1	382106.8	2284275	40
4119	line*coccidia	1	2	1542308	2284275	40
4119	line*coccidia	1	3	275883.5	2284275	40
4119	line*coccidia	2	0	793354.5	2284275	40
4119	line*coccidia	2	1	509198.2	2284275	40
4119	line*coccidia	2	2	300601.7	2284275	40
4119	line*coccidia	2	3	318878	2284275	40
4120	line	1		1610414	617215.7	40
4120	line	2		2878477	617215.7	40
4120	coccidia		0	2865700	872874.8	40
4120	coccidia		1	1434425	872874.8	40
4120	coccidia		2	1913973	872874.8	40
4120	coccidia		3	2763684	872874.8	40
4120	line*coccidia	1	0	82921.67	1234431	40
4120	line*coccidia	1	1	738775.8	1234431	40
4120	line*coccidia	1	2	2144128	1234431	40
4120	line*coccidia	1	3	3475831	1234431	40
4120	line*coccidia	2	0	5648478	1234431	40
4120	line*coccidia	2	1	2130074	1234431	40
4120	line*coccidia	2	2	1683818	1234431	40
4120	line*coccidia	2	3	2051538	1234431	40
4121	line	1		1681480	863634.4	40
4121	line	2		601928	863634.4	40
4121	coccidia		0	733595.6	1221363	40
4121	coccidia		1	2800606	1221363	40
4121	coccidia		2	408566.2	1221363	40
4121	coccidia		3	624048.2	1221363	40
4121	line*coccidia	1	0	544661	1727269	40
4121	line*coccidia	1	1	5151647	1727269	40
4121	line*coccidia	1	2	345879.2	1727269	40
4121	line*coccidia	1	3	683732	1727269	40
4121	line*coccidia	2	0	922530.2	1727269	40
4121	line*coccidia	2	1	449564.5	1727269	40
4121	line*coccidia	2	2	471253.2	1727269	40
4121	line*coccidia	2	3	564364.3	1727269	40
4128	line	1		613564.5	235858	40
4128	line	2		913837.4	235858	40

4128	coccidia		0	664861.5	333553.6	40
4128	coccidia		1	457413.6	333553.6	40
4128	coccidia		2	1212520	333553.6	40
4128	coccidia		3	720008.8	333553.6	40
4128	line*coccidia	1	0	199229.8	471716.1	40
4128	line*coccidia	1	1	42587.17	471716.1	40
4128	line*coccidia	1	2	1824942	471716.1	40
4128	line*coccidia	1	3	387498.8	471716.1	40
4128	line*coccidia	2	0	1130493	471716.1	40
4128	line*coccidia	2	1	872240	471716.1	40
4128	line*coccidia	2	2	600097.7	471716.1	40
4128	line*coccidia	2	3	1052519	471716.1	40
4129	line	1		1895069	985361.9	40
4129	line	2		669501.5	985361.9	40
4129	coccidia		0	1027284	1393512	40
4129	coccidia		1	630227.3	1393512	40
4129	coccidia		2	2924052	1393512	40
4129	coccidia		3	547577.3	1393512	40
4129	line*coccidia	1	0	839342.5	1970724	40
4129	line*coccidia	1	1	472412.7	1970724	40
4129	line*coccidia	1	2	5592619	1970724	40
4129	line*coccidia	1	3	675900.2	1970724	40
4129	line*coccidia	2	0	1215225	1970724	40
4129	line*coccidia	2	1	788041.8	1970724	40
4129	line*coccidia	2	2	255485.5	1970724	40
4129	line*coccidia	2	3	419254.3	1970724	40
4132	line	1		445901.4	133610.4	40
4132	line	2		713265	133610.4	40
4132	coccidia		0	423678.9	188953.6	40
4132	coccidia		1	856463.8	188953.6	40
4132	coccidia		2	298446.8	188953.6	40
4132	coccidia		3	739743.3	188953.6	40
4132	line*coccidia	1	0	496393	267220.8	40
4132	line*coccidia	1	1	137680.5	267220.8	40
4132	line*coccidia	1	2	117953.5	267220.8	40
4132	line*coccidia	1	3	1031579	267220.8	40
4132	line*coccidia	2	0	350964.8	267220.8	40
4132	line*coccidia	2	1	1575247	267220.8	40
4132	line*coccidia	2	2	478940	267220.8	40
4132	line*coccidia	2	3	447908	267220.8	40
4134	line	1		1625519	717099.8	40
4134	line	2		686937	717099.8	40
4134	coccidia		0	1214982	1014132	40
4134	coccidia		1	331349.9	1014132	40
4134	coccidia		2	2636909	1014132	40
4134	coccidia		3	441671.7	1014132	40
4134	line*coccidia	1	0	1365290	1434200	40
4134	line*coccidia	1	1	464039.5	1434200	40
4134	line*coccidia	1	2	4307128	1434200	40

4134	line*coccidia	1	3	365618.7	1434200	40
4134	line*coccidia	2	0	1064674	1434200	40
4134	line*coccidia	2	1	198660.3	1434200	40
4134	line*coccidia	2	2	966689.2	1434200	40
4134	line*coccidia	2	3	517724.7	1434200	40
4201	line	1		454524.5	393853.1	40
4201	line	2		1270121	393853.1	40
4201	coccidia		0	369461.3	556992.3	40
4201	coccidia		1	803496.2	556992.3	40
4201	coccidia		2	1242792	556992.3	40
4201	coccidia		3	1033541	556992.3	40
4201	line*coccidia	1	0	233830.7	787706.1	40
4201	line*coccidia	1	1	295551.3	787706.1	40
4201	line*coccidia	1	2	84049.67	787706.1	40
4201	line*coccidia	1	3	1204667	787706.1	40
4201	line*coccidia	2	0	505092	787706.1	40
4201	line*coccidia	2	1	1311441	787706.1	40
4201	line*coccidia	2	2	2401534	787706.1	40
4201	line*coccidia	2	3	862415.5	787706.1	40
4202	line	1		2001464	748269.3	40
4202	line	2		645612	748269.3	40
4202	coccidia		0	761595.8	1058213	40
4202	coccidia		1	1371996	1058213	40
4202	coccidia		2	679677.8	1058213	40
4202	coccidia		3	2480883	1058213	40
4202	line*coccidia	1	0	1073751	1496539	40
4202	line*coccidia	1	1	2013473	1496539	40
4202	line*coccidia	1	2	320369.5	1496539	40
4202	line*coccidia	1	3	4598263	1496539	40
4202	line*coccidia	2	0	449440.8	1496539	40
4202	line*coccidia	2	1	730518.8	1496539	40
4202	line*coccidia	2	2	1038986	1496539	40
4202	line*coccidia	2	3	363502.3	1496539	40
4203	line	1		403129.7	372632.3	40
4203	line	2		2011169	372632.3	40
4203	coccidia		0	1097857	526981.6	40
4203	coccidia		1	803452.2	526981.6	40
4203	coccidia		2	1014540	526981.6	40
4203	coccidia		3	1912748	526981.6	40
4203	line*coccidia	1	0	662052	745264.6	40
4203	line*coccidia	1	1	670792.2	745264.6	40
4203	line*coccidia	1	2	248075	745264.6	40
4203	line*coccidia	1	3	31599.5	745264.6	40
4203	line*coccidia	2	0	1533661	745264.6	40
4203	line*coccidia	2	1	936112.2	745264.6	40
4203	line*coccidia	2	2	1781005	745264.6	40
4203	line*coccidia	2	3	3793897	745264.6	40
4204	line	1		1639074	696804.8	40
4204	line	2		1796932	696804.8	40

4204	coccidia		0	2471540	985430.8	40
4204	coccidia		1	1786177	985430.8	40
4204	coccidia		2	1874725	985430.8	40
4204	coccidia		3	739568.2	985430.8	40
4204	line*coccidia	1	0	1525064	1393610	40
4204	line*coccidia	1	1	2043407	1393610	40
4204	line*coccidia	1	2	2570539	1393610	40
4204	line*coccidia	1	3	417284.8	1393610	40
4204	line*coccidia	2	0	3418017	1393610	40
4204	line*coccidia	2	1	1528948	1393610	40
4204	line*coccidia	2	2	1178911	1393610	40
4204	line*coccidia	2	3	1061852	1393610	40
4205	line	1		536735.9	223350.9	40
4205	line	2		1119361	223350.9	40
4205	coccidia		0	293310.8	315865.8	40
4205	coccidia		1	1093954	315865.8	40
4205	coccidia		2	631045	315865.8	40
4205	coccidia		3	1293883	315865.8	40
4205	line*coccidia	1	0	231792.2	446701.8	40
4205	line*coccidia	1	1	1087191	446701.8	40
4205	line*coccidia	1	2	622259.8	446701.8	40
4205	line*coccidia	1	3	205700.7	446701.8	40
4205	line*coccidia	2	0	354829.5	446701.8	40
4205	line*coccidia	2	1	1100718	446701.8	40
4205	line*coccidia	2	2	639830.2	446701.8	40
4205	line*coccidia	2	3	2382065	446701.8	40
4206	line	1		1101995	1108543	40
4206	line	2		2119559	1108543	40
4206	coccidia		0	3812664	1567717	40
4206	coccidia		1	581264.5	1567717	40
4206	coccidia		2	1297040	1567717	40
4206	coccidia		3	752139.8	1567717	40
4206	line*coccidia	1	0	936649	2217086	40
4206	line*coccidia	1	1	727715.2	2217086	40
4206	line*coccidia	1	2	1653511	2217086	40
4206	line*coccidia	1	3	1090105	2217086	40
4206	line*coccidia	2	0	6688678	2217086	40
4206	line*coccidia	2	1	434813.8	2217086	40
4206	line*coccidia	2	2	940569.5	2217086	40
4206	line*coccidia	2	3	414174.5	2217086	40
4209	line	1		981624.2	714790.8	40
4209	line	2		1660557	714790.8	40
4209	coccidia		0	777780.6	1010867	40
4209	coccidia		1	1503008	1010867	40
4209	coccidia		2	2265954	1010867	40
4209	coccidia		3	737619.3	1010867	40
4209	line*coccidia	1	0	1473858	1429582	40
4209	line*coccidia	1	1	1280003	1429582	40
4209	line*coccidia	1	2	655634	1429582	40

4209	line*coccidia	1	3	517002	1429582	40
4209	line*coccidia	2	0	81702.83	1429582	40
4209	line*coccidia	2	1	1726014	1429582	40
4209	line*coccidia	2	2	3876273	1429582	40
4209	line*coccidia	2	3	958236.5	1429582	40
4210	line	1		1028825	598324.8	40
4210	line	2		1492974	598324.8	40
4210	coccidia		0	1147421	846159.1	40
4210	coccidia		1	2103765	846159.1	40
4210	coccidia		2	797749.2	846159.1	40
4210	coccidia		3	994662.6	846159.1	40
4210	line*coccidia	1	0	1841183	1196650	40
4210	line*coccidia	1	1	626863.3	1196650	40
4210	line*coccidia	1	2	579285.3	1196650	40
4210	line*coccidia	1	3	1067969	1196650	40
4210	line*coccidia	2	0	453659.3	1196650	40
4210	line*coccidia	2	1	3580667	1196650	40
4210	line*coccidia	2	2	1016213	1196650	40
4210	line*coccidia	2	3	921355.8	1196650	40
4211	line	1		715896.3	188162.1	40
4211	line	2		445282	188162.1	40
4211	coccidia		0	837077.3	266101.4	40
4211	coccidia		1	284421.1	266101.4	40
4211	coccidia		2	720141.9	266101.4	40
4211	coccidia		3	480716.3	266101.4	40
4211	line*coccidia	1	0	997534.3	376324.3	40
4211	line*coccidia	1	1	528010.8	376324.3	40
4211	line*coccidia	1	2	741400.8	376324.3	40
4211	line*coccidia	1	3	596639	376324.3	40
4211	line*coccidia	2	0	676620.3	376324.3	40
4211	line*coccidia	2	1	40831.33	376324.3	40
4211	line*coccidia	2	2	698883	376324.3	40
4211	line*coccidia	2	3	364793.5	376324.3	40
4214	line	1		686873.8	366041.5	40
4214	line	2		988035.4	366041.5	40
4214	coccidia		0	826762.3	517660.9	40
4214	coccidia		1	423411.9	517660.9	40
4214	coccidia		2	1305075	517660.9	40
4214	coccidia		3	794569.1	517660.9	40
4214	line*coccidia	1	0	523151.8	732083.1	40
4214	line*coccidia	1	1	651850.7	732083.1	40
4214	line*coccidia	1	2	434384.8	732083.1	40
4214	line*coccidia	1	3	1138108	732083.1	40
4214	line*coccidia	2	0	1130373	732083.1	40
4214	line*coccidia	2	1	194973.2	732083.1	40
4214	line*coccidia	2	2	2175766	732083.1	40
4214	line*coccidia	2	3	451030.2	732083.1	40
4215	line	1		363709.6	91483.5	40
4215	line	2		399241.3	91483.5	40

4215	coccidia		0	363397.8	129377.2	40
4215	coccidia		1	331226.2	129377.2	40
4215	coccidia		2	127759	129377.2	40
4215	coccidia		3	703518.8	129377.2	40
4215	line*coccidia	1	0	212226.8	182967	40
4215	line*coccidia	1	1	609908.3	182967	40
4215	line*coccidia	1	2	211517.2	182967	40
4215	line*coccidia	1	3	421186.2	182967	40
4215	line*coccidia	2	0	514568.7	182967	40
4215	line*coccidia	2	1	52544	182967	40
4215	line*coccidia	2	2	44000.83	182967	40
4215	line*coccidia	2	3	985851.5	182967	40
4216	line	1		2164238	518539.4	40
4216	line	2		1797292	518539.4	40
4216	coccidia		0	3822545	733325.4	40
4216	coccidia		1	1664204	733325.4	40
4216	coccidia		2	1699391	733325.4	40
4216	coccidia		3	736920.7	733325.4	40
4216	line*coccidia	1	0	3903102	1037079	40
4216	line*coccidia	1	1	2429279	1037079	40
4216	line*coccidia	1	2	1152567	1037079	40
4216	line*coccidia	1	3	1172004	1037079	40
4216	line*coccidia	2	0	3741987	1037079	40
4216	line*coccidia	2	1	899128	1037079	40
4216	line*coccidia	2	2	2246215	1037079	40
4216	line*coccidia	2	3	301837.3	1037079	40
4217	line	1		581353.3	173864.7	40
4217	line	2		449140.2	173864.7	40
4217	coccidia		0	513456.7	245881.8	40
4217	coccidia		1	232382.5	245881.8	40
4217	coccidia		2	473269.3	245881.8	40
4217	coccidia		3	841878.3	245881.8	40
4217	line*coccidia	1	0	577063.3	347729.4	40
4217	line*coccidia	1	1	222985.5	347729.4	40
4217	line*coccidia	1	2	856909.2	347729.4	40
4217	line*coccidia	1	3	668455	347729.4	40
4217	line*coccidia	2	0	449850	347729.4	40
4217	line*coccidia	2	1	241779.5	347729.4	40
4217	line*coccidia	2	2	89629.5	347729.4	40
4217	line*coccidia	2	3	1015302	347729.4	40
4218	line	1		2332231	1091720	40
4218	line	2		2947231	1091720	40
4218	coccidia		0	2046329	1543926	40
4218	coccidia		1	2081145	1543926	40
4218	coccidia		2	2303628	1543926	40
4218	coccidia		3	4127821	1543926	40
4218	line*coccidia	1	0	1345418	2183441	40
4218	line*coccidia	1	1	1601458	2183441	40
4218	line*coccidia	1	2	4157219	2183441	40

4218	line*coccidia	1	3	2224828	2183441	40
4218	line*coccidia	2	0	2747241	2183441	40
4218	line*coccidia	2	1	2560831	2183441	40
4218	line*coccidia	2	2	450037.8	2183441	40
4218	line*coccidia	2	3	6030815	2183441	40
4219	line	1		887031.1	235280.9	40
4219	line	2		295661.2	235280.9	40
4219	coccidia		0	672153.3	332737.5	40
4219	coccidia		1	1111421	332737.5	40
4219	coccidia		2	433228.3	332737.5	40
4219	coccidia		3	148582.3	332737.5	40
4219	line*coccidia	1	0	938130.7	470561.9	40
4219	line*coccidia	1	1	1599613	470561.9	40
4219	line*coccidia	1	2	795878.8	470561.9	40
4219	line*coccidia	1	3	214502	470561.9	40
4219	line*coccidia	2	0	406175.8	470561.9	40
4219	line*coccidia	2	1	623228.3	470561.9	40
4219	line*coccidia	2	2	70577.83	470561.9	40
4219	line*coccidia	2	3	82662.67	470561.9	40
4220	line	1		3893531	804635.5	40
4220	line	2		1536685	804635.5	40
4220	coccidia		0	2261600	1137926	40
4220	coccidia		1	1585089	1137926	40
4220	coccidia		2	4257441	1137926	40
4220	coccidia		3	2756303	1137926	40
4220	line*coccidia	1	0	3249413	1609271	40
4220	line*coccidia	1	1	1582487	1609271	40
4220	line*coccidia	1	2	5459843	1609271	40
4220	line*coccidia	1	3	5282383	1609271	40
4220	line*coccidia	2	0	1273788	1609271	40
4220	line*coccidia	2	1	1587692	1609271	40
4220	line*coccidia	2	2	3055039	1609271	40
4220	line*coccidia	2	3	230222.5	1609271	40
4221	line	1		2040590	1037971	40
4221	line	2		290117.3	1037971	40
4221	coccidia		0	3166481	1467912	40
4221	coccidia		1	524514.8	1467912	40
4221	coccidia		2	445917.8	1467912	40
4221	coccidia		3	524501.3	1467912	40
4221	line*coccidia	1	0	6099070	2075941	40
4221	line*coccidia	1	1	649793.2	2075941	40
4221	line*coccidia	1	2	454238.5	2075941	40
4221	line*coccidia	1	3	959259.5	2075941	40
4221	line*coccidia	2	0	233892.5	2075941	40
4221	line*coccidia	2	1	399236.5	2075941	40
4221	line*coccidia	2	2	437597.2	2075941	40
4221	line*coccidia	2	3	89743.17	2075941	40
4223	line	1		1242993	298357	40
4223	line	2		1675739	298357	40

4223	coccidia		0	1318669	421940.5	40
4223	coccidia		1	1201443	421940.5	40
4223	coccidia		2	1810555	421940.5	40
4223	coccidia		3	1506797	421940.5	40
4223	line*coccidia	1	0	129638.8	596713.9	40
4223	line*coccidia	1	1	1518605	596713.9	40
4223	line*coccidia	1	2	1287540	596713.9	40
4223	line*coccidia	1	3	2036188	596713.9	40
4223	line*coccidia	2	0	2507699	596713.9	40
4223	line*coccidia	2	1	884281.5	596713.9	40
4223	line*coccidia	2	2	2333570	596713.9	40
4223	line*coccidia	2	3	977405.3	596713.9	40
4225	line	1		1066416	529642.2	40
4225	line	2		392783.7	529642.2	40
4225	coccidia		0	1935456	749027.2	40
4225	coccidia		1	376028.2	749027.2	40
4225	coccidia		2	351625.4	749027.2	40
4225	coccidia		3	255290.2	749027.2	40
4225	line*coccidia	1	0	3258107	1059284	40
4225	line*coccidia	1	1	240027.5	1059284	40
4225	line*coccidia	1	2	491155.7	1059284	40
4225	line*coccidia	1	3	276374.2	1059284	40
4225	line*coccidia	2	0	612804.5	1059284	40
4225	line*coccidia	2	1	512028.8	1059284	40
4225	line*coccidia	2	2	212095.2	1059284	40
4225	line*coccidia	2	3	234206.2	1059284	40
4226	line	1		1531422	545386.6	40
4226	line	2		3387700	545386.6	40
4226	coccidia		0	2255750	771293.1	40
4226	coccidia		1	1749859	771293.1	40
4226	coccidia		2	2050060	771293.1	40
4226	coccidia		3	3782574	771293.1	40
4226	line*coccidia	1	0	80744.33	1090773	40
4226	line*coccidia	1	1	1537196	1090773	40
4226	line*coccidia	1	2	1925814	1090773	40
4226	line*coccidia	1	3	2581932	1090773	40
4226	line*coccidia	2	0	4430756	1090773	40
4226	line*coccidia	2	1	1962522	1090773	40
4226	line*coccidia	2	2	2174307	1090773	40
4226	line*coccidia	2	3	4983216	1090773	40
4227	line	1		1819384	864227.3	40
4227	line	2		546952.4	864227.3	40
4227	coccidia		0	794648	1222202	40
4227	coccidia		1	2617258	1222202	40
4227	coccidia		2	425019.6	1222202	40
4227	coccidia		3	895747.8	1222202	40
4227	line*coccidia	1	0	524647.3	1728455	40
4227	line*coccidia	1	1	5017523	1728455	40
4227	line*coccidia	1	2	375155	1728455	40

4227	line*coccidia	1	3	1360213	1728455	40
4227	line*coccidia	2	0	1064649	1728455	40
4227	line*coccidia	2	1	216993.5	1728455	40
4227	line*coccidia	2	2	474884.2	1728455	40
4227	line*coccidia	2	3	431283.2	1728455	40
4228	line	1		690783.3	270895.5	40
4228	line	2		2079555	270895.5	40
4228	coccidia		0	763869.4	383104.1	40
4228	coccidia		1	1968120	383104.1	40
4228	coccidia		2	1448115	383104.1	40
4228	coccidia		3	1360572	383104.1	40
4228	line*coccidia	1	0	159894.7	541791	40
4228	line*coccidia	1	1	46653.5	541791	40
4228	line*coccidia	1	2	1465308	541791	40
4228	line*coccidia	1	3	1091277	541791	40
4228	line*coccidia	2	0	1367844	541791	40
4228	line*coccidia	2	1	3889587	541791	40
4228	line*coccidia	2	2	1430921	541791	40
4228	line*coccidia	2	3	1629867	541791	40
4229	line	1		2611308	1209170	40
4229	line	2		516909	1209170	40
4229	coccidia		0	1271737	1710025	40
4229	coccidia		1	667586.2	1710025	40
4229	coccidia		2	3607451	1710025	40
4229	coccidia		3	709659.3	1710025	40
4229	line*coccidia	1	0	2313852	2418340	40
4229	line*coccidia	1	1	298381.3	2418340	40
4229	line*coccidia	1	2	7013353	2418340	40
4229	line*coccidia	1	3	819644.8	2418340	40
4229	line*coccidia	2	0	229622.7	2418340	40
4229	line*coccidia	2	1	1036791	2418340	40
4229	line*coccidia	2	2	201548.5	2418340	40
4229	line*coccidia	2	3	599673.7	2418340	40
4230	line	1		427199.9	180555.4	40
4230	line	2		1413904	180555.4	40
4230	coccidia		0	422690.8	255343.8	40
4230	coccidia		1	1088754	255343.8	40
4230	coccidia		2	1009117	255343.8	40
4230	coccidia		3	1161647	255343.8	40
4230	line*coccidia	1	0	463817.8	361110.7	40
4230	line*coccidia	1	1	78710	361110.7	40
4230	line*coccidia	1	2	294095.8	361110.7	40
4230	line*coccidia	1	3	872176	361110.7	40
4230	line*coccidia	2	0	381563.7	361110.7	40
4230	line*coccidia	2	1	2098798	361110.7	40
4230	line*coccidia	2	2	1724137	361110.7	40
4230	line*coccidia	2	3	1451119	361110.7	40
4232	line	1		2465578	998913.6	40
4232	line	2		719098.8	998913.6	40

4232	coccidia		0	1785116	1412677	40
4232	coccidia		1	810020.1	1412677	40
4232	coccidia		2	3345732	1412677	40
4232	coccidia		3	428485.6	1412677	40
4232	line*coccidia	1	0	2306605	1997827	40
4232	line*coccidia	1	1	1338116	1997827	40
4232	line*coccidia	1	2	5936541	1997827	40
4232	line*coccidia	1	3	281049	1997827	40
4232	line*coccidia	2	0	1263627	1997827	40
4232	line*coccidia	2	1	281924.5	1997827	40
4232	line*coccidia	2	2	754921.8	1997827	40
4232	line*coccidia	2	3	575922.2	1997827	40
4233	line	1		449042.3	154712.9	40
4233	line	2		778346.3	154712.9	40
4233	coccidia		0	320744.3	218797.1	40
4233	coccidia		1	487524.8	218797.1	40
4233	coccidia		2	788061.9	218797.1	40
4233	coccidia		3	858446	218797.1	40
4233	line*coccidia	1	0	435444.2	309425.9	40
4233	line*coccidia	1	1	251853.8	309425.9	40
4233	line*coccidia	1	2	76667.67	309425.9	40
4233	line*coccidia	1	3	1032203	309425.9	40
4233	line*coccidia	2	0	206044.5	309425.9	40
4233	line*coccidia	2	1	723195.7	309425.9	40
4233	line*coccidia	2	2	1499456	309425.9	40
4233	line*coccidia	2	3	684688.7	309425.9	40
4236	line	1		1419783	558734.3	40
4236	line	2		433793	558734.3	40
4236	coccidia		0	365287.2	790169.7	40
4236	coccidia		1	790167.6	790169.7	40
4236	coccidia		2	675360.7	790169.7	40
4236	coccidia		3	1876336	790169.7	40
4236	line*coccidia	1	0	453727.5	1117469	40
4236	line*coccidia	1	1	1256779	1117469	40
4236	line*coccidia	1	2	471989.3	1117469	40
4236	line*coccidia	1	3	3496635	1117469	40
4236	line*coccidia	2	0	276846.8	1117469	40
4236	line*coccidia	2	1	323556.3	1117469	40
4236	line*coccidia	2	2	878732	1117469	40
4236	line*coccidia	2	3	256036.7	1117469	40
4238	line	1		224983.3	120083.3	40
4238	line	2		742459.5	120083.3	40
4238	coccidia		0	674129.1	169823.5	40
4238	coccidia		1	326489.2	169823.5	40
4238	coccidia		2	266246.9	169823.5	40
4238	coccidia		3	668020.3	169823.5	40
4238	line*coccidia	1	0	364597.5	240166.7	40
4238	line*coccidia	1	1	389955	240166.7	40
4238	line*coccidia	1	2	126095.7	240166.7	40

4238	line*coccidia	1	3	19284.83	240166.7	40
4238	line*coccidia	2	0	983660.7	240166.7	40
4238	line*coccidia	2	1	263023.3	240166.7	40
4238	line*coccidia	2	2	406398.2	240166.7	40
4238	line*coccidia	2	3	1316756	240166.7	40
4239	line	1		294450.6	397979.2	40
4239	line	2		843362.5	397979.2	40
4239	coccidia		0	1207845	562827.5	40
4239	coccidia		1	176709.5	562827.5	40
4239	coccidia		2	478887.8	562827.5	40
4239	coccidia		3	412184.5	562827.5	40
4239	line*coccidia	1	0	122757.3	795958.3	40
4239	line*coccidia	1	1	206210.7	795958.3	40
4239	line*coccidia	1	2	684634.5	795958.3	40
4239	line*coccidia	1	3	164200	795958.3	40
4239	line*coccidia	2	0	2292932	795958.3	40
4239	line*coccidia	2	1	147208.3	795958.3	40
4239	line*coccidia	2	2	273141.2	795958.3	40
4239	line*coccidia	2	3	660169	795958.3	40
4241	line	1		168050.3	91830.19	40
4241	line	2		449616.3	91830.19	40
4241	coccidia		0	100365.4	129867.5	40
4241	coccidia		1	402531.1	129867.5	40
4241	coccidia		2	252463.9	129867.5	40
4241	coccidia		3	479972.9	129867.5	40
4241	line*coccidia	1	0	139585.3	183660.4	40
4241	line*coccidia	1	1	318573.7	183660.4	40
4241	line*coccidia	1	2	155097.7	183660.4	40
4241	line*coccidia	1	3	58944.67	183660.4	40
4241	line*coccidia	2	0	61145.5	183660.4	40
4241	line*coccidia	2	1	486488.5	183660.4	40
4241	line*coccidia	2	2	349830.2	183660.4	40
4241	line*coccidia	2	3	901001.2	183660.4	40
4243	line	1		340644.7	408946.7	40
4243	line	2		948302.5	408946.7	40
4243	coccidia		0	1227587	578338	40
4243	coccidia		1	166255.9	578338	40
4243	coccidia		2	915190.2	578338	40
4243	coccidia		3	268861.1	578338	40
4243	line*coccidia	1	0	322496.7	817893.5	40
4243	line*coccidia	1	1	137233.7	817893.5	40
4243	line*coccidia	1	2	489066.3	817893.5	40
4243	line*coccidia	1	3	413782	817893.5	40
4243	line*coccidia	2	0	2132678	817893.5	40
4243	line*coccidia	2	1	195278.2	817893.5	40
4243	line*coccidia	2	2	1341314	817893.5	40
4243	line*coccidia	2	3	123940.2	817893.5	40
4244	line	1		315808.6	116329.4	40
4244	line	2		472249	116329.4	40

4244	coccidia		0	255362	164514.6	40
4244	coccidia		1	423315.2	164514.6	40
4244	coccidia		2	299369.1	164514.6	40
4244	coccidia		3	598068.8	164514.6	40
4244	line*coccidia	1	0	436226	232658.8	40
4244	line*coccidia	1	1	426887.8	232658.8	40
4244	line*coccidia	1	2	232406.2	232658.8	40
4244	line*coccidia	1	3	167714.3	232658.8	40
4244	line*coccidia	2	0	74498	232658.8	40
4244	line*coccidia	2	1	419742.5	232658.8	40
4244	line*coccidia	2	2	366332	232658.8	40
4244	line*coccidia	2	3	1028423	232658.8	40
4245	line	1		1431155	782813.6	40
4245	line	2		1378475	782813.6	40
4245	coccidia		0	1086862	1107066	40
4245	coccidia		1	2994414	1107066	40
4245	coccidia		2	459917.3	1107066	40
4245	coccidia		3	1078066	1107066	40
4245	line*coccidia	1	0	1722631	1565627	40
4245	line*coccidia	1	1	1517616	1565627	40
4245	line*coccidia	1	2	700514.7	1565627	40
4245	line*coccidia	1	3	1783858	1565627	40
4245	line*coccidia	2	0	451092.7	1565627	40
4245	line*coccidia	2	1	4471212	1565627	40
4245	line*coccidia	2	2	219319.8	1565627	40
4245	line*coccidia	2	3	372274.3	1565627	40
4246	line	1		423793.6	165590.8	40
4246	line	2		569947.5	165590.8	40
4246	coccidia		0	426047.4	234180.7	40
4246	coccidia		1	125444.8	234180.7	40
4246	coccidia		2	714961.2	234180.7	40
4246	coccidia		3	721028.8	234180.7	40
4246	line*coccidia	1	0	690848.3	331181.6	40
4246	line*coccidia	1	1	217664.2	331181.6	40
4246	line*coccidia	1	2	362428.7	331181.6	40
4246	line*coccidia	1	3	424233.2	331181.6	40
4246	line*coccidia	2	0	161246.5	331181.6	40
4246	line*coccidia	2	1	33225.33	331181.6	40
4246	line*coccidia	2	2	1067494	331181.6	40
4246	line*coccidia	2	3	1017825	331181.6	40
4247	line	1		1769561	827091.7	40
4247	line	2		1794274	827091.7	40
4247	coccidia		0	1893625	1169684	40
4247	coccidia		1	1850557	1169684	40
4247	coccidia		2	2436681	1169684	40
4247	coccidia		3	946807.8	1169684	40
4247	line*coccidia	1	0	1889389	1654183	40
4247	line*coccidia	1	1	3298414	1654183	40
4247	line*coccidia	1	2	372362.5	1654183	40

4247	line*coccidia	1	3	1518080	1654183	40
4247	line*coccidia	2	0	1897862	1654183	40
4247	line*coccidia	2	1	402700.7	1654183	40
4247	line*coccidia	2	2	4500999	1654183	40
4247	line*coccidia	2	3	375536.2	1654183	40
4301	line	1		607868	646257.2	40
4301	line	2		1446399	646257.2	40
4301	coccidia		0	574452	913945.7	40
4301	coccidia		1	661537.6	913945.7	40
4301	coccidia		2	226104.6	913945.7	40
4301	coccidia		3	2646440	913945.7	40
4301	line*coccidia	1	0	725158.8	1292514	40
4301	line*coccidia	1	1	1085960	1292514	40
4301	line*coccidia	1	2	219104.5	1292514	40
4301	line*coccidia	1	3	401248.5	1292514	40
4301	line*coccidia	2	0	423745.2	1292514	40
4301	line*coccidia	2	1	237115	1292514	40
4301	line*coccidia	2	2	233104.7	1292514	40
4301	line*coccidia	2	3	4891631	1292514	40
4302	line	1		2572361	1005542	40
4302	line	2		3632111	1005542	40
4302	coccidia		0	4642692	1422051	40
4302	coccidia		1	3671208	1422051	40
4302	coccidia		2	3248872	1422051	40
4302	coccidia		3	846172.6	1422051	40
4302	line*coccidia	1	0	1342665	2011084	40
4302	line*coccidia	1	1	5757209	2011084	40
4302	line*coccidia	1	2	1817685	2011084	40
4302	line*coccidia	1	3	1371886	2011084	40
4302	line*coccidia	2	0	7942719	2011084	40
4302	line*coccidia	2	1	1585206	2011084	40
4302	line*coccidia	2	2	4680059	2011084	40
4302	line*coccidia	2	3	320459.7	2011084	40
4303	line	1		599743.8	1217934	40
4303	line	2		3250376	1217934	40
4303	coccidia		0	828899.2	1722419	40
4303	coccidia		1	162741.9	1722419	40
4303	coccidia		2	315057.3	1722419	40
4303	coccidia		3	6393542	1722419	40
4303	line*coccidia	1	0	1305730	2435868	40
4303	line*coccidia	1	1	150240.7	2435868	40
4303	line*coccidia	1	2	593775.3	2435868	40
4303	line*coccidia	1	3	349228.7	2435868	40
4303	line*coccidia	2	0	352068	2435868	40
4303	line*coccidia	2	1	175243.2	2435868	40
4303	line*coccidia	2	2	36339.17	2435868	40
4303	line*coccidia	2	3	12437855	2435868	40
4304	line	1		2317282	567321.7	40
4304	line	2		1569418	567321.7	40

4304	coccidia		0	1590248	802314.1	40
4304	coccidia		1	1936765	802314.1	40
4304	coccidia		2	2186402	802314.1	40
4304	coccidia		3	2059985	802314.1	40
4304	line*coccidia	1	0	1993356	1134643	40
4304	line*coccidia	1	1	1703229	1134643	40
4304	line*coccidia	1	2	3314124	1134643	40
4304	line*coccidia	1	3	2258420	1134643	40
4304	line*coccidia	2	0	1187140	1134643	40
4304	line*coccidia	2	1	2170301	1134643	40
4304	line*coccidia	2	2	1058681	1134643	40
4304	line*coccidia	2	3	1861551	1134643	40
4306	line	1		2425084	335026.3	40
4306	line	2		2445692	335026.3	40
4306	coccidia		0	3473593	473798.7	40
4306	coccidia		1	2742294	473798.7	40
4306	coccidia		2	1547125	473798.7	40
4306	coccidia		3	1978539	473798.7	40
4306	line*coccidia	1	0	2594442	670052.6	40
4306	line*coccidia	1	1	2681526	670052.6	40
4306	line*coccidia	1	2	1109401	670052.6	40
4306	line*coccidia	1	3	3314966	670052.6	40
4306	line*coccidia	2	0	4352745	670052.6	40
4306	line*coccidia	2	1	2803063	670052.6	40
4306	line*coccidia	2	2	1984848	670052.6	40
4306	line*coccidia	2	3	642112.8	670052.6	40
4308	line	1		341580.7	41630.35	40
4308	line	2		297811.4	41630.35	40
4308	coccidia		0	459184.8	58874.2	40
4308	coccidia		1	383266.1	58874.2	40
4308	coccidia		2	181285.9	58874.2	40
4308	coccidia		3	255047.3	58874.2	40
4308	line*coccidia	1	0	447597	83260.7	40
4308	line*coccidia	1	1	412531.7	83260.7	40
4308	line*coccidia	1	2	116221	83260.7	40
4308	line*coccidia	1	3	389973.2	83260.7	40
4308	line*coccidia	2	0	470772.7	83260.7	40
4308	line*coccidia	2	1	354000.5	83260.7	40
4308	line*coccidia	2	2	246350.8	83260.7	40
4308	line*coccidia	2	3	120121.5	83260.7	40
4310	line	1		2151539	263171.8	40
4310	line	2		1306144	263171.8	40
4310	coccidia		0	1991240	372181.1	40
4310	coccidia		1	1859529	372181.1	40
4310	coccidia		2	937643.1	372181.1	40
4310	coccidia		3	2126956	372181.1	40
4310	line*coccidia	1	0	2493149	526343.6	40
4310	line*coccidia	1	1	2145221	526343.6	40
4310	line*coccidia	1	2	540335	526343.6	40

4310	line*coccidia	1	3	3427452	526343.6	40
4310	line*coccidia	2	0	1489330	526343.6	40
4310	line*coccidia	2	1	1573836	526343.6	40
4310	line*coccidia	2	2	1334951	526343.6	40
4310	line*coccidia	2	3	826460.5	526343.6	40
4311	line	1		938697.7	141940.8	40
4311	line	2		455226	141940.8	40
4311	coccidia		0	955644.5	200734.5	40
4311	coccidia		1	533376.3	200734.5	40
4311	coccidia		2	647586	200734.5	40
4311	coccidia		3	651240.6	200734.5	40
4311	line*coccidia	1	0	1466220	283881.5	40
4311	line*coccidia	1	1	391248.5	283881.5	40
4311	line*coccidia	1	2	748806.8	283881.5	40
4311	line*coccidia	1	3	1148516	283881.5	40
4311	line*coccidia	2	0	445069.5	283881.5	40
4311	line*coccidia	2	1	675504	283881.5	40
4311	line*coccidia	2	2	546365.2	283881.5	40
4311	line*coccidia	2	3	153965.3	283881.5	40
4312	line	1		0	60394.8	40
4312	line	2		604539.5	60394.8	40
4312	coccidia		0	381227.3	85411.15	40
4312	coccidia		1	361321.6	85411.15	40
4312	coccidia		2	386195.5	85411.15	40
4312	coccidia		3	80334.5	85411.15	40
4312	line*coccidia	1	0	2.91E-11	120789.6	40
4312	line*coccidia	1	1	2.91E-11	120789.6	40
4312	line*coccidia	1	2	2.91E-11	120789.6	40
4312	line*coccidia	1	3	-8.7E-11	120789.6	40
4312	line*coccidia	2	0	762454.7	120789.6	40
4312	line*coccidia	2	1	722643.2	120789.6	40
4312	line*coccidia	2	2	772391	120789.6	40
4312	line*coccidia	2	3	160669	120789.6	40
4313	line	1		2222683	302387.2	40
4313	line	2		3291227	302387.2	40
4313	coccidia		0	4084039	427640.1	40
4313	coccidia		1	2692670	427640.1	40
4313	coccidia		2	1495807	427640.1	40
4313	coccidia		3	2755305	427640.1	40
4313	line*coccidia	1	0	3293613	604774.5	40
4313	line*coccidia	1	1	1223936	604774.5	40
4313	line*coccidia	1	2	1279362	604774.5	40
4313	line*coccidia	1	3	3093822	604774.5	40
4313	line*coccidia	2	0	4874465	604774.5	40
4313	line*coccidia	2	1	4161404	604774.5	40
4313	line*coccidia	2	2	1712252	604774.5	40
4313	line*coccidia	2	3	2416789	604774.5	40
4314	line	1		305359.2	121011.4	40
4314	line	2		92868.75	121011.4	40

4314	coccidia		0	96124.25	171135.9	40
4314	coccidia		1	169014.5	171135.9	40
4314	coccidia		2	72021.92	171135.9	40
4314	coccidia		3	459295.3	171135.9	40
4314	line*coccidia	1	0	115516	242022.7	40
4314	line*coccidia	1	1	164996.5	242022.7	40
4314	line*coccidia	1	2	70701.17	242022.7	40
4314	line*coccidia	1	3	870223.2	242022.7	40
4314	line*coccidia	2	0	76732.5	242022.7	40
4314	line*coccidia	2	1	173032.5	242022.7	40
4314	line*coccidia	2	2	73342.67	242022.7	40
4314	line*coccidia	2	3	48367.33	242022.7	40
4315	line	1		553006.8	58554.44	40
4315	line	2		536497.2	58554.44	40
4315	coccidia		0	544304.9	82808.48	40
4315	coccidia		1	660033	82808.48	40
4315	coccidia		2	400370.7	82808.48	40
4315	coccidia		3	574299.5	82808.48	40
4315	line*coccidia	1	0	520616	117108.9	40
4315	line*coccidia	1	1	531621	117108.9	40
4315	line*coccidia	1	2	318699.8	117108.9	40
4315	line*coccidia	1	3	841090.5	117108.9	40
4315	line*coccidia	2	0	567993.8	117108.9	40
4315	line*coccidia	2	1	788445	117108.9	40
4315	line*coccidia	2	2	482041.5	117108.9	40
4315	line*coccidia	2	3	307508.5	117108.9	40
4316	line	1		1270200	156154.6	40
4316	line	2		616075.6	156154.6	40
4316	coccidia		0	1379672	220835.9	40
4316	coccidia		1	540483.8	220835.9	40
4316	coccidia		2	448827.8	220835.9	40
4316	coccidia		3	1403567	220835.9	40
4316	line*coccidia	1	0	2084368	312309.2	40
4316	line*coccidia	1	1	578783.5	312309.2	40
4316	line*coccidia	1	2	338897.3	312309.2	40
4316	line*coccidia	1	3	2078749	312309.2	40
4316	line*coccidia	2	0	674975.2	312309.2	40
4316	line*coccidia	2	1	502184.2	312309.2	40
4316	line*coccidia	2	2	558758.2	312309.2	40
4316	line*coccidia	2	3	728384.8	312309.2	40
4318	line	1		536145.6	53435.97	40
4318	line	2		198181.5	53435.97	40
4318	coccidia		0	560028.8	75569.88	40
4318	coccidia		1	333276.4	75569.88	40
4318	coccidia		2	150860.9	75569.88	40
4318	coccidia		3	424488.1	75569.88	40
4318	line*coccidia	1	0	819400	106871.9	40
4318	line*coccidia	1	1	425246	106871.9	40
4318	line*coccidia	1	2	106078.7	106871.9	40

4318	line*coccidia	1	3	793857.7	106871.9	40
4318	line*coccidia	2	0	300657.7	106871.9	40
4318	line*coccidia	2	1	241306.8	106871.9	40
4318	line*coccidia	2	2	195643.2	106871.9	40
4318	line*coccidia	2	3	55118.5	106871.9	40
4319	line	1		1274487	171651.1	40
4319	line	2		1205987	171651.1	40
4319	coccidia		0	1729969	242751.4	40
4319	coccidia		1	1610010	242751.4	40
4319	coccidia		2	650109.7	242751.4	40
4319	coccidia		3	970858.1	242751.4	40
4319	line*coccidia	1	0	1754589	343302.3	40
4319	line*coccidia	1	1	1467410	343302.3	40
4319	line*coccidia	1	2	611681.3	343302.3	40
4319	line*coccidia	1	3	1264266	343302.3	40
4319	line*coccidia	2	0	1705350	343302.3	40
4319	line*coccidia	2	1	1752611	343302.3	40
4319	line*coccidia	2	2	688538	343302.3	40
4319	line*coccidia	2	3	677450.5	343302.3	40
4320	line	1		341343.6	58803.08	40
4320	line	2		382521.8	58803.08	40
4320	coccidia		0	738373.8	83160.11	40
4320	coccidia		1	321710.3	83160.11	40
4320	coccidia		2	113369.4	83160.11	40
4320	coccidia		3	274277.4	83160.11	40
4320	line*coccidia	1	0	963847.8	117606.2	40
4320	line*coccidia	1	1	63319.67	117606.2	40
4320	line*coccidia	1	2	84556.67	117606.2	40
4320	line*coccidia	1	3	253650.2	117606.2	40
4320	line*coccidia	2	0	512899.7	117606.2	40
4320	line*coccidia	2	1	580100.8	117606.2	40
4320	line*coccidia	2	2	142182.2	117606.2	40
4320	line*coccidia	2	3	294904.7	117606.2	40
4321	line	1		55663.63	18014.05	40
4321	line	2		59364.04	18014.05	40
4321	coccidia		0	18936.33	25475.71	40
4321	coccidia		1	101822.4	25475.71	40
4321	coccidia		2	54999.83	25475.71	40
4321	coccidia		3	54296.75	25475.71	40
4321	line*coccidia	1	0	6557.667	36028.1	40
4321	line*coccidia	1	1	95190.17	36028.1	40
4321	line*coccidia	1	2	61262.83	36028.1	40
4321	line*coccidia	1	3	59643.83	36028.1	40
4321	line*coccidia	2	0	31315	36028.1	40
4321	line*coccidia	2	1	108454.7	36028.1	40
4321	line*coccidia	2	2	48736.83	36028.1	40
4321	line*coccidia	2	3	48949.67	36028.1	40
4322	line	1		273755	41591.13	40
4322	line	2		238437.6	41591.13	40

4322	coccidia		0	293435.1	58818.75	40
4322	coccidia		1	192316.6	58818.75	40
4322	coccidia		2	270847.6	58818.75	40
4322	coccidia		3	267786	58818.75	40
4322	line*coccidia	1	0	354239	83182.27	40
4322	line*coccidia	1	1	75593.67	83182.27	40
4322	line*coccidia	1	2	250140.8	83182.27	40
4322	line*coccidia	1	3	415046.7	83182.27	40
4322	line*coccidia	2	0	232631.2	83182.27	40
4322	line*coccidia	2	1	309039.5	83182.27	40
4322	line*coccidia	2	2	291554.3	83182.27	40
4322	line*coccidia	2	3	120525.3	83182.27	40
4323	line	1		238856.3	30916.26	40
4323	line	2		301835.5	30916.26	40
4323	coccidia		0	206307.8	43722.2	40
4323	coccidia		1	313395.6	43722.2	40
4323	coccidia		2	273965.6	43722.2	40
4323	coccidia		3	287714.8	43722.2	40
4323	line*coccidia	1	0	165620.3	61832.52	40
4323	line*coccidia	1	1	203135	61832.52	40
4323	line*coccidia	1	2	214112.3	61832.52	40
4323	line*coccidia	1	3	372557.7	61832.52	40
4323	line*coccidia	2	0	246995.2	61832.52	40
4323	line*coccidia	2	1	423656.2	61832.52	40
4323	line*coccidia	2	2	333818.8	61832.52	40
4323	line*coccidia	2	3	202871.8	61832.52	40
4326	line	1		0	20052.13	40
4326	line	2		119576	20052.13	40
4326	coccidia		0	203177.1	28358	40
4326	coccidia		1	-1.1E-11	28358	40
4326	coccidia		2	35975	28358	40
4326	coccidia		3	-7.3E-12	28358	40
4326	line*coccidia	1	0	4.37E-11	40104.26	40
4326	line*coccidia	1	1	-1.5E-11	40104.26	40
4326	line*coccidia	1	2	-1.5E-11	40104.26	40
4326	line*coccidia	1	3	-1.5E-11	40104.26	40
4326	line*coccidia	2	0	406354.2	40104.26	40
4326	line*coccidia	2	1	-7.3E-12	40104.26	40
4326	line*coccidia	2	2	71950	40104.26	40
4326	line*coccidia	2	3	0	40104.26	40
4327	line	1		450000.9	246658.4	40
4327	line	2		252369.6	246658.4	40
4327	coccidia		0	261437.9	348827.7	40
4327	coccidia		1	902985.8	348827.7	40
4327	coccidia		2	70140.75	348827.7	40
4327	coccidia		3	170176.5	348827.7	40
4327	line*coccidia	1	0	173021.8	493316.8	40
4327	line*coccidia	1	1	1517931	493316.8	40
4327	line*coccidia	1	2	34185.17	493316.8	40

4327	line*coccidia	1	3	74866	493316.8	40
4327	line*coccidia	2	0	349854	493316.8	40
4327	line*coccidia	2	1	288041	493316.8	40
4327	line*coccidia	2	2	106096.3	493316.8	40
4327	line*coccidia	2	3	265487	493316.8	40
4328	line	1		791541.3	84707.22	40
4328	line	2		929610.5	84707.22	40
4328	coccidia		0	1477532	119794.1	40
4328	coccidia		1	755763.5	119794.1	40
4328	coccidia		2	408116.3	119794.1	40
4328	coccidia		3	800892.1	119794.1	40
4328	line*coccidia	1	0	1532297	169414.4	40
4328	line*coccidia	1	1	308958	169414.4	40
4328	line*coccidia	1	2	337272.5	169414.4	40
4328	line*coccidia	1	3	987638.2	169414.4	40
4328	line*coccidia	2	0	1422767	169414.4	40
4328	line*coccidia	2	1	1202569	169414.4	40
4328	line*coccidia	2	2	478960.2	169414.4	40
4328	line*coccidia	2	3	614146	169414.4	40
4329	line	1		1197819	389890.4	40
4329	line	2		1213778	389890.4	40
4329	coccidia		0	1265087	551388.3	40
4329	coccidia		1	816620.8	551388.3	40
4329	coccidia		2	528447.3	551388.3	40
4329	coccidia		3	2213038	551388.3	40
4329	line*coccidia	1	0	1238681	779780.9	40
4329	line*coccidia	1	1	803169.7	779780.9	40
4329	line*coccidia	1	2	418037	779780.9	40
4329	line*coccidia	1	3	2331387	779780.9	40
4329	line*coccidia	2	0	1291493	779780.9	40
4329	line*coccidia	2	1	830072	779780.9	40
4329	line*coccidia	2	2	638857.5	779780.9	40
4329	line*coccidia	2	3	2094689	779780.9	40
4338	line	1		203578.7	44702.47	40
4338	line	2		147642	44702.47	40
4338	coccidia		0	235802.8	63218.84	40
4338	coccidia		1	142826.3	63218.84	40
4338	coccidia		2	89285.92	63218.84	40
4338	coccidia		3	234526.3	63218.84	40
4338	line*coccidia	1	0	191286	89404.94	40
4338	line*coccidia	1	1	134529.8	89404.94	40
4338	line*coccidia	1	2	55881	89404.94	40
4338	line*coccidia	1	3	432617.8	89404.94	40
4338	line*coccidia	2	0	280319.7	89404.94	40
4338	line*coccidia	2	1	151122.8	89404.94	40
4338	line*coccidia	2	2	122690.8	89404.94	40
4338	line*coccidia	2	3	36434.83	89404.94	40
4339	line	1		383300	51188.71	40
4339	line	2		319181.5	51188.71	40

4339	coccidia		0	508788.4	72391.77	40
4339	coccidia		1	470661	72391.77	40
4339	coccidia		2	75988.92	72391.77	40
4339	coccidia		3	349524.5	72391.77	40
4339	line*coccidia	1	0	708624.3	102377.4	40
4339	line*coccidia	1	1	212892.7	102377.4	40
4339	line*coccidia	1	2	81199	102377.4	40
4339	line*coccidia	1	3	530483.8	102377.4	40
4339	line*coccidia	2	0	308952.5	102377.4	40
4339	line*coccidia	2	1	728429.3	102377.4	40
4339	line*coccidia	2	2	70778.83	102377.4	40
4339	line*coccidia	2	3	168565.2	102377.4	40
4340	line	1		73582.17	20645.05	40
4340	line	2		46294.38	20645.05	40
4340	coccidia		0	67343.08	29196.51	40
4340	coccidia		1	39744.67	29196.51	40
4340	coccidia		2	38030.5	29196.51	40
4340	coccidia		3	94634.83	29196.51	40
4340	line*coccidia	1	0	51161.83	41290.1	40
4340	line*coccidia	1	1	79489.33	41290.1	40
4340	line*coccidia	1	2	0	41290.1	40
4340	line*coccidia	1	3	163677.5	41290.1	40
4340	line*coccidia	2	0	83524.33	41290.1	40
4340	line*coccidia	2	1	0	41290.1	40
4340	line*coccidia	2	2	76061	41290.1	40
4340	line*coccidia	2	3	25592.17	41290.1	40
4342	line	1		406948.4	75433.39	40
4342	line	2		528731.5	75433.39	40
4342	coccidia		0	570895	106678.9	40
4342	coccidia		1	590821.9	106678.9	40
4342	coccidia		2	225238.4	106678.9	40
4342	coccidia		3	484404.3	106678.9	40
4342	line*coccidia	1	0	579628.8	150866.8	40
4342	line*coccidia	1	1	129745	150866.8	40
4342	line*coccidia	1	2	295819.8	150866.8	40
4342	line*coccidia	1	3	622599.8	150866.8	40
4342	line*coccidia	2	0	562161.2	150866.8	40
4342	line*coccidia	2	1	1051899	150866.8	40
4342	line*coccidia	2	2	154657	150866.8	40
4342	line*coccidia	2	3	346208.8	150866.8	40
4403	line	1		913666	116673.3	40
4403	line	2		688606.8	116673.3	40
4403	coccidia		0	1252270	165000.9	40
4403	coccidia		1	869783.8	165000.9	40
4403	coccidia		2	365077.7	165000.9	40
4403	coccidia		3	717414.1	165000.9	40
4403	line*coccidia	1	0	1532069	233346.5	40
4403	line*coccidia	1	1	1030569	233346.5	40
4403	line*coccidia	1	2	213160	233346.5	40

4403	line*coccidia	1	3	878866.2	233346.5	40
4403	line*coccidia	2	0	972471.3	233346.5	40
4403	line*coccidia	2	1	708998.7	233346.5	40
4403	line*coccidia	2	2	516995.3	233346.5	40
4403	line*coccidia	2	3	555962	233346.5	40
4404	line	1		3094698	973641	40
4404	line	2		3457125	973641	40
4404	coccidia		0	2014286	1376936	40
4404	coccidia		1	6103739	1376936	40
4404	coccidia		2	2265080	1376936	40
4404	coccidia		3	2720541	1376936	40
4404	line*coccidia	1	0	1563239	1947282	40
4404	line*coccidia	1	1	6227234	1947282	40
4404	line*coccidia	1	2	1968034	1947282	40
4404	line*coccidia	1	3	2620284	1947282	40
4404	line*coccidia	2	0	2465332	1947282	40
4404	line*coccidia	2	1	5980244	1947282	40
4404	line*coccidia	2	2	2562127	1947282	40
4404	line*coccidia	2	3	2820798	1947282	40
4406	line	1		21891975	2355277	40
4406	line	2		23338055	2355277	40
4406	coccidia		0	25827371	3330865	40
4406	coccidia		1	20576688	3330865	40
4406	coccidia		2	20902187	3330865	40
4406	coccidia		3	23153815	3330865	40
4406	line*coccidia	1	0	25955373	4710555	40
4406	line*coccidia	1	1	17744255	4710555	40
4406	line*coccidia	1	2	20498085	4710555	40
4406	line*coccidia	1	3	23370188	4710555	40
4406	line*coccidia	2	0	25699369	4710555	40
4406	line*coccidia	2	1	23409120	4710555	40
4406	line*coccidia	2	2	21306288	4710555	40
4406	line*coccidia	2	3	22937442	4710555	40
4407	line	1		9685575	2719625	40
4407	line	2		2902539	2719625	40
4407	coccidia		0	2826485	3846131	40
4407	coccidia		1	8787050	3846131	40
4407	coccidia		2	2571176	3846131	40
4407	coccidia		3	10991517	3846131	40
4407	line*coccidia	1	0	3378904	5439250	40
4407	line*coccidia	1	1	13283131	5439250	40
4407	line*coccidia	1	2	3278702	5439250	40
4407	line*coccidia	1	3	18801564	5439250	40
4407	line*coccidia	2	0	2274067	5439250	40
4407	line*coccidia	2	1	4290970	5439250	40
4407	line*coccidia	2	2	1863651	5439250	40
4407	line*coccidia	2	3	3181471	5439250	40
4408	line	1		6251811	618339.7	40
4408	line	2		5660583	618339.7	40

4408	coccidia		0	6946038	874464.3	40
4408	coccidia		1	7334594	874464.3	40
4408	coccidia		2	3776389	874464.3	40
4408	coccidia		3	5767768	874464.3	40
4408	line*coccidia	1	0	7266890	1236679	40
4408	line*coccidia	1	1	7504731	1236679	40
4408	line*coccidia	1	2	3693179	1236679	40
4408	line*coccidia	1	3	6542446	1236679	40
4408	line*coccidia	2	0	6625186	1236679	40
4408	line*coccidia	2	1	7164458	1236679	40
4408	line*coccidia	2	2	3859599	1236679	40
4408	line*coccidia	2	3	4993091	1236679	40
4409	line	1		588614.2	158193.1	40
4409	line	2		401932.9	158193.1	40
4409	coccidia		0	262033.4	223718.8	40
4409	coccidia		1	1031685	223718.8	40
4409	coccidia		2	304931	223718.8	40
4409	coccidia		3	382444.5	223718.8	40
4409	line*coccidia	1	0	219842	316386.1	40
4409	line*coccidia	1	1	1445975	316386.1	40
4409	line*coccidia	1	2	377777	316386.1	40
4409	line*coccidia	1	3	310863	316386.1	40
4409	line*coccidia	2	0	304224.8	316386.1	40
4409	line*coccidia	2	1	617395.8	316386.1	40
4409	line*coccidia	2	2	232085	316386.1	40
4409	line*coccidia	2	3	454026	316386.1	40
4410	line	1		13860787	1402501	40
4410	line	2		16731527	1402501	40
4410	coccidia		0	14742314	1983436	40
4410	coccidia		1	20450956	1983436	40
4410	coccidia		2	11053975	1983436	40
4410	coccidia		3	14937383	1983436	40
4410	line*coccidia	1	0	15273459	2805002	40
4410	line*coccidia	1	1	14295628	2805002	40
4410	line*coccidia	1	2	10096611	2805002	40
4410	line*coccidia	1	3	15777451	2805002	40
4410	line*coccidia	2	0	14211168	2805002	40
4410	line*coccidia	2	1	26606285	2805002	40
4410	line*coccidia	2	2	12011338	2805002	40
4410	line*coccidia	2	3	14097316	2805002	40
4411	line	1		10434964	1075443	40
4411	line	2		6343135	1075443	40
4411	coccidia		0	10480033	1520907	40
4411	coccidia		1	5131215	1520907	40
4411	coccidia		2	8320526	1520907	40
4411	coccidia		3	9624425	1520907	40
4411	line*coccidia	1	0	13318316	2150887	40
4411	line*coccidia	1	1	7556804	2150887	40
4411	line*coccidia	1	2	6442523	2150887	40

4411	line*coccidia	1	3	14422214	2150887	40
4411	line*coccidia	2	0	7641750	2150887	40
4411	line*coccidia	2	1	2705626	2150887	40
4411	line*coccidia	2	2	10198529	2150887	40
4411	line*coccidia	2	3	4826636	2150887	40
4414	line	1		3791027	572942.6	40
4414	line	2		3099271	572942.6	40
4414	coccidia		0	4757763	810263.3	40
4414	coccidia		1	2632939	810263.3	40
4414	coccidia		2	2347156	810263.3	40
4414	coccidia		3	4042738	810263.3	40
4414	line*coccidia	1	0	5788718	1145885	40
4414	line*coccidia	1	1	2061527	1145885	40
4414	line*coccidia	1	2	2666509	1145885	40
4414	line*coccidia	1	3	4647354	1145885	40
4414	line*coccidia	2	0	3726808	1145885	40
4414	line*coccidia	2	1	3204351	1145885	40
4414	line*coccidia	2	2	2027804	1145885	40
4414	line*coccidia	2	3	3438121	1145885	40
4417	line	1		6462151	1961268	40
4417	line	2		4468118	1961268	40
4417	coccidia		0	4895214	2773652	40
4417	coccidia		1	7275438	2773652	40
4417	coccidia		2	5411469	2773652	40
4417	coccidia		3	4278417	2773652	40
4417	line*coccidia	1	0	2859591	3922536	40
4417	line*coccidia	1	1	11361420	3922536	40
4417	line*coccidia	1	2	8568386	3922536	40
4417	line*coccidia	1	3	3059208	3922536	40
4417	line*coccidia	2	0	6930838	3922536	40
4417	line*coccidia	2	1	3189455	3922536	40
4417	line*coccidia	2	2	2254551	3922536	40
4417	line*coccidia	2	3	5497626	3922536	40
4419	line	1		514361.6	52246.31	40
4419	line	2		524114.8	52246.31	40
4419	coccidia		0	582794.8	73887.44	40
4419	coccidia		1	641184.1	73887.44	40
4419	coccidia		2	377804.4	73887.44	40
4419	coccidia		3	475169.5	73887.44	40
4419	line*coccidia	1	0	545182.5	104492.6	40
4419	line*coccidia	1	1	526005.8	104492.6	40
4419	line*coccidia	1	2	307004	104492.6	40
4419	line*coccidia	1	3	679254.2	104492.6	40
4419	line*coccidia	2	0	620407.2	104492.6	40
4419	line*coccidia	2	1	756362.3	104492.6	40
4419	line*coccidia	2	2	448604.8	104492.6	40
4419	line*coccidia	2	3	271084.8	104492.6	40
4423	line	1		16552931	3295073	40
4423	line	2		20926050	3295073	40

4423	coccidia		0	20678270	4659936	40
4423	coccidia		1	14519299	4659936	40
4423	coccidia		2	20317603	4659936	40
4423	coccidia		3	19442791	4659936	40
4423	line*coccidia	1	0	26336008	6590145	40
4423	line*coccidia	1	1	8186086	6590145	40
4423	line*coccidia	1	2	11284530	6590145	40
4423	line*coccidia	1	3	20405102	6590145	40
4423	line*coccidia	2	0	15020532	6590145	40
4423	line*coccidia	2	1	20852513	6590145	40
4423	line*coccidia	2	2	29350676	6590145	40
4423	line*coccidia	2	3	18480481	6590145	40
4424	line	1		26395005	6865827	40
4424	line	2		35891467	6865827	40
4424	coccidia		0	41897952	9709745	40
4424	coccidia		1	13683614	9709745	40
4424	coccidia		2	29778682	9709745	40
4424	coccidia		3	39212696	9709745	40
4424	line*coccidia	1	0	33646275	13731653	40
4424	line*coccidia	1	1	15742861	13731653	40
4424	line*coccidia	1	2	14354199	13731653	40
4424	line*coccidia	1	3	41836684	13731653	40
4424	line*coccidia	2	0	50149629	13731653	40
4424	line*coccidia	2	1	11624367	13731653	40
4424	line*coccidia	2	2	45203165	13731653	40
4424	line*coccidia	2	3	36588708	13731653	40
4425	line	1		24982158	3067223	40
4425	line	2		21363145	3067223	40
4425	coccidia		0	26947982	4337708	40
4425	coccidia		1	20987701	4337708	40
4425	coccidia		2	19295730	4337708	40
4425	coccidia		3	25459192	4337708	40
4425	line*coccidia	1	0	27958989	6134446	40
4425	line*coccidia	1	1	26810808	6134446	40
4425	line*coccidia	1	2	13907521	6134446	40
4425	line*coccidia	1	3	31251312	6134446	40
4425	line*coccidia	2	0	25936975	6134446	40
4425	line*coccidia	2	1	15164594	6134446	40
4425	line*coccidia	2	2	24683939	6134446	40
4425	line*coccidia	2	3	19667072	6134446	40
4426	line	1		2215916	218957.2	40
4426	line	2		2194285	218957.2	40
4426	coccidia		0	3348566	309652.2	40
4426	coccidia		1	2116874	309652.2	40
4426	coccidia		2	842612.5	309652.2	40
4426	coccidia		3	2512351	309652.2	40
4426	line*coccidia	1	0	3958124	437914.3	40
4426	line*coccidia	1	1	881140.3	437914.3	40
4426	line*coccidia	1	2	653196.2	437914.3	40

4426	line*coccidia	1	3	3371205	437914.3	40
4426	line*coccidia	2	0	2739009	437914.3	40
4426	line*coccidia	2	1	3352607	437914.3	40
4426	line*coccidia	2	2	1032029	437914.3	40
4426	line*coccidia	2	3	1653497	437914.3	40
4427	line	1		2211450	232361.8	40
4427	line	2		2079194	232361.8	40
4427	coccidia		0	3570692	328609.2	40
4427	coccidia		1	1747022	328609.2	40
4427	coccidia		2	846796.6	328609.2	40
4427	coccidia		3	2416778	328609.2	40
4427	line*coccidia	1	0	3510209	464723.6	40
4427	line*coccidia	1	1	1169388	464723.6	40
4427	line*coccidia	1	2	473072.5	464723.6	40
4427	line*coccidia	1	3	3693132	464723.6	40
4427	line*coccidia	2	0	3631175	464723.6	40
4427	line*coccidia	2	1	2324657	464723.6	40
4427	line*coccidia	2	2	1220521	464723.6	40
4427	line*coccidia	2	3	1140424	464723.6	40
4429	line	1		13489511	2291074	40
4429	line	2		20501279	2291074	40
4429	coccidia		0	28969996	3240068	40
4429	coccidia		1	11725271	3240068	40
4429	coccidia		2	12920542	3240068	40
4429	coccidia		3	14365772	3240068	40
4429	line*coccidia	1	0	25512051	4582148	40
4429	line*coccidia	1	1	11202123	4582148	40
4429	line*coccidia	1	2	5947498	4582148	40
4429	line*coccidia	1	3	11296374	4582148	40
4429	line*coccidia	2	0	32427941	4582148	40
4429	line*coccidia	2	1	12248418	4582148	40
4429	line*coccidia	2	2	19893587	4582148	40
4429	line*coccidia	2	3	17435171	4582148	40
4503	line	1		497288.9	140018.3	40
4503	line	2		736068.3	140018.3	40
4503	coccidia		0	559219.4	198015.7	40
4503	coccidia		1	636216.3	198015.7	40
4503	coccidia		2	803327.5	198015.7	40
4503	coccidia		3	467951.3	198015.7	40
4503	line*coccidia	1	0	493755	280036.5	40
4503	line*coccidia	1	1	450708.7	280036.5	40
4503	line*coccidia	1	2	453902.5	280036.5	40
4503	line*coccidia	1	3	590789.5	280036.5	40
4503	line*coccidia	2	0	624683.8	280036.5	40
4503	line*coccidia	2	1	821723.8	280036.5	40
4503	line*coccidia	2	2	1152753	280036.5	40
4503	line*coccidia	2	3	345113.2	280036.5	40
4505	line	1		1663471	436931.8	40
4505	line	2		527141.8	436931.8	40

4505	coccidia		0	546527.8	617914.9	40
4505	coccidia		1	1420714	617914.9	40
4505	coccidia		2	1514767	617914.9	40
4505	coccidia		3	899217.1	617914.9	40
4505	line*coccidia	1	0	776151.8	873863.6	40
4505	line*coccidia	1	1	1702999	873863.6	40
4505	line*coccidia	1	2	2613144	873863.6	40
4505	line*coccidia	1	3	1561589	873863.6	40
4505	line*coccidia	2	0	316903.7	873863.6	40
4505	line*coccidia	2	1	1138428	873863.6	40
4505	line*coccidia	2	2	416390.3	873863.6	40
4505	line*coccidia	2	3	236844.8	873863.6	40
4506	line	1		2135955	389472.3	40
4506	line	2		1542500	389472.3	40
4506	coccidia		0	1999781	550797.1	40
4506	coccidia		1	1465548	550797.1	40
4506	coccidia		2	2169152	550797.1	40
4506	coccidia		3	1722431	550797.1	40
4506	line*coccidia	1	0	2236455	778944.7	40
4506	line*coccidia	1	1	681696.7	778944.7	40
4506	line*coccidia	1	2	3080776	778944.7	40
4506	line*coccidia	1	3	2544893	778944.7	40
4506	line*coccidia	2	0	1763107	778944.7	40
4506	line*coccidia	2	1	2249399	778944.7	40
4506	line*coccidia	2	2	1257527	778944.7	40
4506	line*coccidia	2	3	899969.2	778944.7	40
4508	line	1		1520660	547453.4	40
4508	line	2		286354.4	547453.4	40
4508	coccidia		0	240556.4	774216	40
4508	coccidia		1	1155140	774216	40
4508	coccidia		2	1985562	774216	40
4508	coccidia		3	232770.9	774216	40
4508	line*coccidia	1	0	151106.8	1094907	40
4508	line*coccidia	1	1	2008303	1094907	40
4508	line*coccidia	1	2	3568375	1094907	40
4508	line*coccidia	1	3	354856.2	1094907	40
4508	line*coccidia	2	0	330006	1094907	40
4508	line*coccidia	2	1	301977.8	1094907	40
4508	line*coccidia	2	2	402748.2	1094907	40
4508	line*coccidia	2	3	110685.7	1094907	40
4510	line	1		1577857	170061.9	40
4510	line	2		1428968	170061.9	40
4510	coccidia		0	1957680	240503.8	40
4510	coccidia		1	1301255	240503.8	40
4510	coccidia		2	934007.4	240503.8	40
4510	coccidia		3	1820707	240503.8	40
4510	line*coccidia	1	0	2205900	340123.7	40
4510	line*coccidia	1	1	95695.5	340123.7	40
4510	line*coccidia	1	2	1027575	340123.7	40

4510	line*coccidia	1	3	2982257	340123.7	40
4510	line*coccidia	2	0	1709459	340123.7	40
4510	line*coccidia	2	1	2506815	340123.7	40
4510	line*coccidia	2	2	840439.8	340123.7	40
4510	line*coccidia	2	3	659156.8	340123.7	40
4512	line	1		5828935	2651261	40
4512	line	2		15233495	2651261	40
4512	coccidia		0	7708196	3749449	40
4512	coccidia		1	2507647	3749449	40
4512	coccidia		2	21378603	3749449	40
4512	coccidia		3	10530416	3749449	40
4512	line*coccidia	1	0	10088020	5302522	40
4512	line*coccidia	1	1	1414236	5302522	40
4512	line*coccidia	1	2	1906037	5302522	40
4512	line*coccidia	1	3	9907448	5302522	40
4512	line*coccidia	2	0	5328372	5302522	40
4512	line*coccidia	2	1	3601058	5302522	40
4512	line*coccidia	2	2	40851168	5302522	40
4512	line*coccidia	2	3	11153384	5302522	40
4516	line	1		1690419	658894.6	40
4516	line	2		0	658894.6	40
4516	coccidia		0	93259.17	931817.7	40
4516	coccidia		1	846181.5	931817.7	40
4516	coccidia		2	2441397	931817.7	40
4516	coccidia		3	-2.3E-10	931817.7	40
4516	line*coccidia	1	0	186518.3	1317789	40
4516	line*coccidia	1	1	1692363	1317789	40
4516	line*coccidia	1	2	4882794	1317789	40
4516	line*coccidia	1	3	-4.7E-10	1317789	40
4516	line*coccidia	2	0	0	1317789	40
4516	line*coccidia	2	1	0	1317789	40
4516	line*coccidia	2	2	0	1317789	40
4516	line*coccidia	2	3	0	1317789	40
4517	line	1		1245105	656878	40
4517	line	2		155014.4	656878	40
4517	coccidia		0	289736.7	928965.8	40
4517	coccidia		1	2026294	928965.8	40
4517	coccidia		2	322878.3	928965.8	40
4517	coccidia		3	161330.5	928965.8	40
4517	line*coccidia	1	0	307798.7	1313756	40
4517	line*coccidia	1	1	3895512	1313756	40
4517	line*coccidia	1	2	523898.7	1313756	40
4517	line*coccidia	1	3	253212.3	1313756	40
4517	line*coccidia	2	0	271674.7	1313756	40
4517	line*coccidia	2	1	157076.5	1313756	40
4517	line*coccidia	2	2	121857.8	1313756	40
4517	line*coccidia	2	3	69448.67	1313756	40
4518	line	1		61414.63	8510.521	40
4518	line	2		21358.92	8510.521	40

4518	coccidia		0	57098.75	12035.69	40
4518	coccidia		1	0	12035.69	40
4518	coccidia		2	7670.417	12035.69	40
4518	coccidia		3	100777.9	12035.69	40
4518	line*coccidia	1	0	79041.17	17021.04	40
4518	line*coccidia	1	1	0	17021.04	40
4518	line*coccidia	1	2	-7.3E-12	17021.04	40
4518	line*coccidia	1	3	166617.3	17021.04	40
4518	line*coccidia	2	0	35156.33	17021.04	40
4518	line*coccidia	2	1	0	17021.04	40
4518	line*coccidia	2	2	15340.83	17021.04	40
4518	line*coccidia	2	3	34938.5	17021.04	40
4519	line	1		38614.29	10026.93	40
4519	line	2		72467.5	10026.93	40
4519	coccidia		0	70686.67	14180.21	40
4519	coccidia		1	68033	14180.21	40
4519	coccidia		2	7.28E-12	14180.21	40
4519	coccidia		3	83443.92	14180.21	40
4519	line*coccidia	1	0	7.28E-12	20053.85	40
4519	line*coccidia	1	1	-2.2E-11	20053.85	40
4519	line*coccidia	1	2	7.28E-12	20053.85	40
4519	line*coccidia	1	3	154457.2	20053.85	40
4519	line*coccidia	2	0	141373.3	20053.85	40
4519	line*coccidia	2	1	136066	20053.85	40
4519	line*coccidia	2	2	7.28E-12	20053.85	40
4519	line*coccidia	2	3	12430.67	20053.85	40
4520	line	1		18015033	2747540	40
4520	line	2		12139781	2747540	40
4520	coccidia		0	21105446	3885608	40
4520	coccidia		1	8747962	3885608	40
4520	coccidia		2	12268169	3885608	40
4520	coccidia		3	18188053	3885608	40
4520	line*coccidia	1	0	22713119	5495079	40
4520	line*coccidia	1	1	11620089	5495079	40
4520	line*coccidia	1	2	14391048	5495079	40
4520	line*coccidia	1	3	23335878	5495079	40
4520	line*coccidia	2	0	19497773	5495079	40
4520	line*coccidia	2	1	5875834	5495079	40
4520	line*coccidia	2	2	10145291	5495079	40
4520	line*coccidia	2	3	13040227	5495079	40
4521	line	1		24130.92	4066.461	40
4521	line	2		17143.83	4066.461	40
4521	coccidia		0	34287.67	5750.845	40
4521	coccidia		1	3.64E-12	5750.845	40
4521	coccidia		2	-3.6E-12	5750.845	40
4521	coccidia		3	48261.83	5750.845	40
4521	line*coccidia	1	0	1.09E-11	8132.922	40
4521	line*coccidia	1	1	3.64E-12	8132.922	40
4521	line*coccidia	1	2	-3.6E-12	8132.922	40

4521	line*coccidia	1	3	96523.67	8132.922	40
4521	line*coccidia	2	0	68575.33	8132.922	40
4521	line*coccidia	2	1	3.64E-12	8132.922	40
4521	line*coccidia	2	2	-3.6E-12	8132.922	40
4521	line*coccidia	2	3	-3.6E-12	8132.922	40
4522	line	1		0	23715.47	40
4522	line	2		299786.8	23715.47	40
4522	coccidia		0	364532.8	33538.74	40
4522	coccidia		1	2.91E-11	33538.74	40
4522	coccidia		2	156795.3	33538.74	40
4522	coccidia		3	78245.5	33538.74	40
4522	line*coccidia	1	0	-8.7E-11	47430.95	40
4522	line*coccidia	1	1	2.91E-11	47430.95	40
4522	line*coccidia	1	2	2.91E-11	47430.95	40
4522	line*coccidia	1	3	2.91E-11	47430.95	40
4522	line*coccidia	2	0	729065.5	47430.95	40
4522	line*coccidia	2	1	2.91E-11	47430.95	40
4522	line*coccidia	2	2	313590.5	47430.95	40
4522	line*coccidia	2	3	156491	47430.95	40
4523	line	1		11957478	1878379	40
4523	line	2		11297016	1878379	40
4523	coccidia		0	17096483	2656430	40
4523	coccidia		1	8084800	2656430	40
4523	coccidia		2	9802996	2656430	40
4523	coccidia		3	11524709	2656430	40
4523	line*coccidia	1	0	16816853	3756759	40
4523	line*coccidia	1	1	7748820	3756759	40
4523	line*coccidia	1	2	10160425	3756759	40
4523	line*coccidia	1	3	13103812	3756759	40
4523	line*coccidia	2	0	17376112	3756759	40
4523	line*coccidia	2	1	8420780	3756759	40
4523	line*coccidia	2	2	9445567	3756759	40
4523	line*coccidia	2	3	9945606	3756759	40
4605	line	1		262218.6	114042.8	40
4605	line	2		381598.9	114042.8	40
4605	coccidia		0	203912.6	161280.9	40
4605	coccidia		1	149773.5	161280.9	40
4605	coccidia		2	572033.1	161280.9	40
4605	coccidia		3	361915.8	161280.9	40
4605	line*coccidia	1	0	197967.2	228085.6	40
4605	line*coccidia	1	1	57579.5	228085.6	40
4605	line*coccidia	1	2	232544.8	228085.6	40
4605	line*coccidia	1	3	560782.8	228085.6	40
4605	line*coccidia	2	0	209858	228085.6	40
4605	line*coccidia	2	1	241967.5	228085.6	40
4605	line*coccidia	2	2	911521.3	228085.6	40
4605	line*coccidia	2	3	163048.8	228085.6	40
4606	line	1		919473.9	278742.5	40
4606	line	2		252478.6	278742.5	40

4606	coccidia		0	339702.8	394201.4	40
4606	coccidia		1	823427.5	394201.4	40
4606	coccidia		2	238895.6	394201.4	40
4606	coccidia		3	941879.3	394201.4	40
4606	line*coccidia	1	0	462648.2	557485	40
4606	line*coccidia	1	1	1227455	557485	40
4606	line*coccidia	1	2	337966.8	557485	40
4606	line*coccidia	1	3	1649826	557485	40
4606	line*coccidia	2	0	216757.3	557485	40
4606	line*coccidia	2	1	419400.3	557485	40
4606	line*coccidia	2	2	139824.3	557485	40
4606	line*coccidia	2	3	233932.5	557485	40
4608	line	1		525121.8	195703.3	40
4608	line	2		257844.3	195703.3	40
4608	coccidia		0	192294.8	276766.3	40
4608	coccidia		1	252011.8	276766.3	40
4608	coccidia		2	347071.6	276766.3	40
4608	coccidia		3	774554.1	276766.3	40
4608	line*coccidia	1	0	219388.3	391406.6	40
4608	line*coccidia	1	1	65761.83	391406.6	40
4608	line*coccidia	1	2	402714.2	391406.6	40
4608	line*coccidia	1	3	1412623	391406.6	40
4608	line*coccidia	2	0	165201.3	391406.6	40
4608	line*coccidia	2	1	438261.7	391406.6	40
4608	line*coccidia	2	2	291429	391406.6	40
4608	line*coccidia	2	3	136485.3	391406.6	40
4609	line	1		697483.5	128610.9	40
4609	line	2		224211.5	128610.9	40
4609	coccidia		0	275571.7	181883.3	40
4609	coccidia		1	556381.5	181883.3	40
4609	coccidia		2	445676.7	181883.3	40
4609	coccidia		3	565760.1	181883.3	40
4609	line*coccidia	1	0	347187.2	257221.9	40
4609	line*coccidia	1	1	678052.7	257221.9	40
4609	line*coccidia	1	2	789718.8	257221.9	40
4609	line*coccidia	1	3	974975.3	257221.9	40
4609	line*coccidia	2	0	203956.2	257221.9	40
4609	line*coccidia	2	1	434710.3	257221.9	40
4609	line*coccidia	2	2	101634.5	257221.9	40
4609	line*coccidia	2	3	156544.8	257221.9	40
4611	line	1		420908.8	131178.3	40
4611	line	2		502649.7	131178.3	40
4611	coccidia		0	360836.8	185514.2	40
4611	coccidia		1	563120.6	185514.2	40
4611	coccidia		2	667956.2	185514.2	40
4611	coccidia		3	255203.3	185514.2	40
4611	line*coccidia	1	0	226570.2	262356.7	40
4611	line*coccidia	1	1	793809.7	262356.7	40
4611	line*coccidia	1	2	368436.5	262356.7	40

4611	line*coccidia	1	3	294818.8	262356.7	40
4611	line*coccidia	2	0	495103.5	262356.7	40
4611	line*coccidia	2	1	332431.5	262356.7	40
4611	line*coccidia	2	2	967475.8	262356.7	40
4611	line*coccidia	2	3	215587.8	262356.7	40
4613	line	1		255455.7	61695.58	40
4613	line	2		395655	61695.58	40
4613	coccidia		0	458977.6	87250.72	40
4613	coccidia		1	383867.8	87250.72	40
4613	coccidia		2	331818.3	87250.72	40
4613	coccidia		3	127557.8	87250.72	40
4613	line*coccidia	1	0	340338.7	123391.2	40
4613	line*coccidia	1	1	319005.3	123391.2	40
4613	line*coccidia	1	2	259051.7	123391.2	40
4613	line*coccidia	1	3	103427.2	123391.2	40
4613	line*coccidia	2	0	577616.5	123391.2	40
4613	line*coccidia	2	1	448730.3	123391.2	40
4613	line*coccidia	2	2	404584.8	123391.2	40
4613	line*coccidia	2	3	151688.5	123391.2	40
4616	line	1		740104.3	220729.8	40
4616	line	2		266217.6	220729.8	40
4616	coccidia		0	287440.7	312159.1	40
4616	coccidia		1	216545.3	312159.1	40
4616	coccidia		2	478292.9	312159.1	40
4616	coccidia		3	1030365	312159.1	40
4616	line*coccidia	1	0	305501.3	441459.6	40
4616	line*coccidia	1	1	338017.2	441459.6	40
4616	line*coccidia	1	2	646074	441459.6	40
4616	line*coccidia	1	3	1670825	441459.6	40
4616	line*coccidia	2	0	269380	441459.6	40
4616	line*coccidia	2	1	95073.33	441459.6	40
4616	line*coccidia	2	2	310511.8	441459.6	40
4616	line*coccidia	2	3	389905.2	441459.6	40
4623	line	1		612676.1	180395.4	40
4623	line	2		511488.5	180395.4	40
4623	coccidia		0	355963.5	255117.7	40
4623	coccidia		1	441584.2	255117.7	40
4623	coccidia		2	682221.1	255117.7	40
4623	coccidia		3	768560.6	255117.7	40
4623	line*coccidia	1	0	250414.7	360790.9	40
4623	line*coccidia	1	1	725629.5	360790.9	40
4623	line*coccidia	1	2	195867	360790.9	40
4623	line*coccidia	1	3	1278793	360790.9	40
4623	line*coccidia	2	0	461512.3	360790.9	40
4623	line*coccidia	2	1	157538.8	360790.9	40
4623	line*coccidia	2	2	1168575	360790.9	40
4623	line*coccidia	2	3	258327.8	360790.9	40
4625	line	1		106336.2	22918.73	40
4625	line	2		57026.5	22918.73	40

4625	coccidia		0	25180.83	32411.98	40
4625	coccidia		1	19257.83	32411.98	40
4625	coccidia		2	124419.3	32411.98	40
4625	coccidia		3	157867.4	32411.98	40
4625	line*coccidia	1	0	50361.67	45837.46	40
4625	line*coccidia	1	1	38515.67	45837.46	40
4625	line*coccidia	1	2	116891	45837.46	40
4625	line*coccidia	1	3	219576.5	45837.46	40
4625	line*coccidia	2	0	0	45837.46	40
4625	line*coccidia	2	1	1.46E-11	45837.46	40
4625	line*coccidia	2	2	131947.7	45837.46	40
4625	line*coccidia	2	3	96158.33	45837.46	40
4627	line	1		242370.3	67768.24	40
4627	line	2		272734.2	67768.24	40
4627	coccidia		0	272999.9	95838.77	40
4627	coccidia		1	194390	95838.77	40
4627	coccidia		2	353727	95838.77	40
4627	coccidia		3	209092	95838.77	40
4627	line*coccidia	1	0	98716.17	135536.5	40
4627	line*coccidia	1	1	264327.8	135536.5	40
4627	line*coccidia	1	2	357713.8	135536.5	40
4627	line*coccidia	1	3	248723.2	135536.5	40
4627	line*coccidia	2	0	447283.7	135536.5	40
4627	line*coccidia	2	1	124452.2	135536.5	40
4627	line*coccidia	2	2	349740.2	135536.5	40
4627	line*coccidia	2	3	169460.8	135536.5	40
4628	line	1		258333.9	27987.99	40
4628	line	2		184777.2	27987.99	40
4628	coccidia		0	263473.9	39581	40
4628	coccidia		1	186102.1	39581	40
4628	coccidia		2	136558.3	39581	40
4628	coccidia		3	300087.9	39581	40
4628	line*coccidia	1	0	389255.8	55975.98	40
4628	line*coccidia	1	1	74774.17	55975.98	40
4628	line*coccidia	1	2	152043.5	55975.98	40
4628	line*coccidia	1	3	417262	55975.98	40
4628	line*coccidia	2	0	137692	55975.98	40
4628	line*coccidia	2	1	297430	55975.98	40
4628	line*coccidia	2	2	121073	55975.98	40
4628	line*coccidia	2	3	182913.8	55975.98	40
4629	line	1		2.91E-11	24763.96	40
4629	line	2		329537	24763.96	40
4629	coccidia		0	184069.3	35021.53	40
4629	coccidia		1	305192.3	35021.53	40
4629	coccidia		2	89369.25	35021.53	40
4629	coccidia		3	80443.17	35021.53	40
4629	line*coccidia	1	0	2.91E-11	49527.93	40
4629	line*coccidia	1	1	2.91E-11	49527.93	40
4629	line*coccidia	1	2	2.91E-11	49527.93	40

4629	line*coccidia	1	3	2.91E-11	49527.93	40
4629	line*coccidia	2	0	368138.5	49527.93	40
4629	line*coccidia	2	1	610384.7	49527.93	40
4629	line*coccidia	2	2	178738.5	49527.93	40
4629	line*coccidia	2	3	160886.3	49527.93	40
4632	line	1		153339.7	16709.25	40
4632	line	2		66132.13	16709.25	40
4632	coccidia		0	205986.8	23630.45	40
4632	coccidia		1	90972.67	23630.45	40
4632	coccidia		2	53717.58	23630.45	40
4632	coccidia		3	88266.58	23630.45	40
4632	line*coccidia	1	0	321884.8	33418.5	40
4632	line*coccidia	1	1	93621.83	33418.5	40
4632	line*coccidia	1	2	51896.33	33418.5	40
4632	line*coccidia	1	3	145955.8	33418.5	40
4632	line*coccidia	2	0	90088.83	33418.5	40
4632	line*coccidia	2	1	88323.5	33418.5	40
4632	line*coccidia	2	2	55538.83	33418.5	40
4632	line*coccidia	2	3	30577.33	33418.5	40
4633	line	1		70252.29	14010.11	40
4633	line	2		32833.83	14010.11	40
4633	coccidia		0	80398.17	19813.29	40
4633	coccidia		1	25165.17	19813.29	40
4633	coccidia		2	-7.3E-12	19813.29	40
4633	coccidia		3	100608.9	19813.29	40
4633	line*coccidia	1	0	110755.8	28020.22	40
4633	line*coccidia	1	1	-1.5E-11	28020.22	40
4633	line*coccidia	1	2	-7.3E-12	28020.22	40
4633	line*coccidia	1	3	170253.3	28020.22	40
4633	line*coccidia	2	0	50040.5	28020.22	40
4633	line*coccidia	2	1	50330.33	28020.22	40
4633	line*coccidia	2	2	-7.3E-12	28020.22	40
4633	line*coccidia	2	3	30964.5	28020.22	40
4634	line	1		171065.9	40490.4	40
4634	line	2		-1.8E-12	40490.4	40
4634	coccidia		0	63373.58	57262.07	40
4634	coccidia		1	66722.5	57262.07	40
4634	coccidia		2	59505.33	57262.07	40
4634	coccidia		3	152530.4	57262.07	40
4634	line*coccidia	1	0	126747.2	80980.8	40
4634	line*coccidia	1	1	133445	80980.8	40
4634	line*coccidia	1	2	119010.7	80980.8	40
4634	line*coccidia	1	3	305060.8	80980.8	40
4634	line*coccidia	2	0	0	80980.8	40
4634	line*coccidia	2	1	-7.3E-12	80980.8	40
4634	line*coccidia	2	2	7.28E-12	80980.8	40
4634	line*coccidia	2	3	-7.3E-12	80980.8	40
4701	line	1		1591700	657274.1	40
4701	line	2		1741834	657274.1	40

4701	coccidia		0	2635486	929526	40
4701	coccidia		1	174746.9	929526	40
4701	coccidia		2	2247552	929526	40
4701	coccidia		3	1609283	929526	40
4701	line*coccidia	1	0	2177245	1314548	40
4701	line*coccidia	1	1	162790.7	1314548	40
4701	line*coccidia	1	2	1985998	1314548	40
4701	line*coccidia	1	3	2040766	1314548	40
4701	line*coccidia	2	0	3093726	1314548	40
4701	line*coccidia	2	1	186703.2	1314548	40
4701	line*coccidia	2	2	2509106	1314548	40
4701	line*coccidia	2	3	1177800	1314548	40
4707	line	1		577955.4	123316.6	40
4707	line	2		171550.8	123316.6	40
4707	coccidia		0	206421.8	174396	40
4707	coccidia		1	310094	174396	40
4707	coccidia		2	708468.4	174396	40
4707	coccidia		3	274028.3	174396	40
4707	line*coccidia	1	0	245839	246633.2	40
4707	line*coccidia	1	1	493086.2	246633.2	40
4707	line*coccidia	1	2	1200527	246633.2	40
4707	line*coccidia	1	3	372369.5	246633.2	40
4707	line*coccidia	2	0	167004.5	246633.2	40
4707	line*coccidia	2	1	127101.8	246633.2	40
4707	line*coccidia	2	2	216409.8	246633.2	40
4707	line*coccidia	2	3	175687.2	246633.2	40
4708	line	1		548782.5	68756.54	40
4708	line	2		447787.3	68756.54	40
4708	coccidia		0	373751.8	97236.44	40
4708	coccidia		1	514978.7	97236.44	40
4708	coccidia		2	544759.4	97236.44	40
4708	coccidia		3	559649.7	97236.44	40
4708	line*coccidia	1	0	320666.5	137513.1	40
4708	line*coccidia	1	1	520627.7	137513.1	40
4708	line*coccidia	1	2	489549	137513.1	40
4708	line*coccidia	1	3	864287	137513.1	40
4708	line*coccidia	2	0	426837.2	137513.1	40
4708	line*coccidia	2	1	509329.7	137513.1	40
4708	line*coccidia	2	2	599969.8	137513.1	40
4708	line*coccidia	2	3	255012.3	137513.1	40
4711	line	1		321651.5	71843.71	40
4711	line	2		147847.8	71843.71	40
4711	coccidia		0	91336.83	101602.3	40
4711	coccidia		1	352716.9	101602.3	40
4711	coccidia		2	126125.2	101602.3	40
4711	coccidia		3	368819.6	101602.3	40
4711	line*coccidia	1	0	113296.8	143687.4	40
4711	line*coccidia	1	1	558965.2	143687.4	40
4711	line*coccidia	1	2	72580.67	143687.4	40

4711	line*coccidia	1	3	541763.2	143687.4	40
4711	line*coccidia	2	0	69376.83	143687.4	40
4711	line*coccidia	2	1	146468.7	143687.4	40
4711	line*coccidia	2	2	179669.7	143687.4	40
4711	line*coccidia	2	3	195876	143687.4	40
4713	line	1		120979.5	48673.76	40
4713	line	2		236161.4	48673.76	40
4713	coccidia		0	195581	68835.09	40
4713	coccidia		1	280510.7	68835.09	40
4713	coccidia		2	68511	68835.09	40
4713	coccidia		3	169679.2	68835.09	40
4713	line*coccidia	1	0	216171.5	97347.51	40
4713	line*coccidia	1	1	52989.83	97347.51	40
4713	line*coccidia	1	2	78193	97347.51	40
4713	line*coccidia	1	3	136563.7	97347.51	40
4713	line*coccidia	2	0	174990.5	97347.51	40
4713	line*coccidia	2	1	508031.5	97347.51	40
4713	line*coccidia	2	2	58829	97347.51	40
4713	line*coccidia	2	3	202794.7	97347.51	40
4718	line	1		407832.7	42870.12	40
4718	line	2		284442.4	42870.12	40
4718	coccidia		0	403329.7	60627.5	40
4718	coccidia		1	444041.2	60627.5	40
4718	coccidia		2	189358.9	60627.5	40
4718	coccidia		3	347820.5	60627.5	40
4718	line*coccidia	1	0	524742.3	85740.23	40
4718	line*coccidia	1	1	397048.8	85740.23	40
4718	line*coccidia	1	2	183000.3	85740.23	40
4718	line*coccidia	1	3	526539.3	85740.23	40
4718	line*coccidia	2	0	281917	85740.23	40
4718	line*coccidia	2	1	491033.5	85740.23	40
4718	line*coccidia	2	2	195717.5	85740.23	40
4718	line*coccidia	2	3	169101.7	85740.23	40
4719	line	1		0	50978.65	40
4719	line	2		252814.8	50978.65	40
4719	coccidia		0	86352.25	72094.7	40
4719	coccidia		1	363201	72094.7	40
4719	coccidia		2	38959.08	72094.7	40
4719	coccidia		3	17117.25	72094.7	40
4719	line*coccidia	1	0	2.91E-11	101957.3	40
4719	line*coccidia	1	1	-8.7E-11	101957.3	40
4719	line*coccidia	1	2	2.91E-11	101957.3	40
4719	line*coccidia	1	3	2.91E-11	101957.3	40
4719	line*coccidia	2	0	172704.5	101957.3	40
4719	line*coccidia	2	1	726402	101957.3	40
4719	line*coccidia	2	2	77918.17	101957.3	40
4719	line*coccidia	2	3	34234.5	101957.3	40
4720	line	1		371477.2	81282.36	40
4720	line	2		281939.5	81282.36	40

4720	coccidia		0	328790.3	114950.6	40
4720	coccidia		1	259101.6	114950.6	40
4720	coccidia		2	193293.2	114950.6	40
4720	coccidia		3	525648.3	114950.6	40
4720	line*coccidia	1	0	303458	162564.7	40
4720	line*coccidia	1	1	57240.17	162564.7	40
4720	line*coccidia	1	2	170086.3	162564.7	40
4720	line*coccidia	1	3	955124.3	162564.7	40
4720	line*coccidia	2	0	354122.7	162564.7	40
4720	line*coccidia	2	1	460963	162564.7	40
4720	line*coccidia	2	2	216500	162564.7	40
4720	line*coccidia	2	3	96172.17	162564.7	40
4722	line	1		228311.4	31538.47	40
4722	line	2		171027	31538.47	40
4722	coccidia		0	187432.5	44602.14	40
4722	coccidia		1	222362.7	44602.14	40
4722	coccidia		2	164723	44602.14	40
4722	coccidia		3	224158.8	44602.14	40
4722	line*coccidia	1	0	234316.5	63076.95	40
4722	line*coccidia	1	1	229639.3	63076.95	40
4722	line*coccidia	1	2	136712.7	63076.95	40
4722	line*coccidia	1	3	312577.2	63076.95	40
4722	line*coccidia	2	0	140548.5	63076.95	40
4722	line*coccidia	2	1	215086	63076.95	40
4722	line*coccidia	2	2	192733.3	63076.95	40
4722	line*coccidia	2	3	135740.3	63076.95	40
4723	line	1		486115	44725.76	40
4723	line	2		183804.7	44725.76	40
4723	coccidia		0	314228.8	63251.78	40
4723	coccidia		1	264306.9	63251.78	40
4723	coccidia		2	309573.3	63251.78	40
4723	coccidia		3	451730.3	63251.78	40
4723	line*coccidia	1	0	526158.3	89451.52	40
4723	line*coccidia	1	1	363255.5	89451.52	40
4723	line*coccidia	1	2	375682.2	89451.52	40
4723	line*coccidia	1	3	679363.8	89451.52	40
4723	line*coccidia	2	0	102299.2	89451.52	40
4723	line*coccidia	2	1	165358.3	89451.52	40
4723	line*coccidia	2	2	243464.3	89451.52	40
4723	line*coccidia	2	3	224096.8	89451.52	40
4728	line	1		420221	92249.12	40
4728	line	2		262837.7	92249.12	40
4728	coccidia		0	237859.9	130460	40
4728	coccidia		1	269411.8	130460	40
4728	coccidia		2	314818	130460	40
4728	coccidia		3	544027.6	130460	40
4728	line*coccidia	1	0	245928.7	184498.2	40
4728	line*coccidia	1	1	236685.5	184498.2	40
4728	line*coccidia	1	2	230887	184498.2	40

4728	line*coccidia	1	3	967382.7	184498.2	40
4728	line*coccidia	2	0	229791.2	184498.2	40
4728	line*coccidia	2	1	302138.2	184498.2	40
4728	line*coccidia	2	2	398749	184498.2	40
4728	line*coccidia	2	3	120672.5	184498.2	40
4735	line	1		90957.42	19409.22	40
4735	line	2		98810	19409.22	40
4735	coccidia		0	77884.08	27448.78	40
4735	coccidia		1	141314.7	27448.78	40
4735	coccidia		2	59556.25	27448.78	40
4735	coccidia		3	100779.8	27448.78	40
4735	line*coccidia	1	0	74690.67	38818.44	40
4735	line*coccidia	1	1	132250.5	38818.44	40
4735	line*coccidia	1	2	33778.17	38818.44	40
4735	line*coccidia	1	3	123110.3	38818.44	40
4735	line*coccidia	2	0	81077.5	38818.44	40
4735	line*coccidia	2	1	150378.8	38818.44	40
4735	line*coccidia	2	2	85334.33	38818.44	40
4735	line*coccidia	2	3	78449.33	38818.44	40
4736	line	1		391479.5	132275.4	40
4736	line	2		266041.5	132275.4	40
4736	coccidia		0	288302.9	187065.6	40
4736	coccidia		1	217148.7	187065.6	40
4736	coccidia		2	286386.3	187065.6	40
4736	coccidia		3	523204.3	187065.6	40
4736	line*coccidia	1	0	264036.5	264550.8	40
4736	line*coccidia	1	1	109749.3	264550.8	40
4736	line*coccidia	1	2	307147.8	264550.8	40
4736	line*coccidia	1	3	884984.5	264550.8	40
4736	line*coccidia	2	0	312569.3	264550.8	40
4736	line*coccidia	2	1	324548	264550.8	40
4736	line*coccidia	2	2	265624.8	264550.8	40
4736	line*coccidia	2	3	161424	264550.8	40
4738	line	1		150115.8	40705.25	40
4738	line	2		31932.58	40705.25	40
4738	coccidia		0	33921.33	57565.92	40
4738	coccidia		1	33492.5	57565.92	40
4738	coccidia		2	125394.7	57565.92	40
4738	coccidia		3	171288.3	57565.92	40
4738	line*coccidia	1	0	49951.83	81410.5	40
4738	line*coccidia	1	1	30903.67	81410.5	40
4738	line*coccidia	1	2	191281.3	81410.5	40
4738	line*coccidia	1	3	328326.5	81410.5	40
4738	line*coccidia	2	0	17890.83	81410.5	40
4738	line*coccidia	2	1	36081.33	81410.5	40
4738	line*coccidia	2	2	59508	81410.5	40
4738	line*coccidia	2	3	14250.17	81410.5	40
4740	line	1		80828.33	12098.81	40
4740	line	2		121770.7	12098.81	40

4740	coccidia		0	104275.8	17110.31	40
4740	coccidia		1	113225.7	17110.31	40
4740	coccidia		2	75252.42	17110.31	40
4740	coccidia		3	112444.2	17110.31	40
4740	line*coccidia	1	0	80448.33	24197.63	40
4740	line*coccidia	1	1	61896.83	24197.63	40
4740	line*coccidia	1	2	75513.67	24197.63	40
4740	line*coccidia	1	3	105454.5	24197.63	40
4740	line*coccidia	2	0	128103.2	24197.63	40
4740	line*coccidia	2	1	164554.5	24197.63	40
4740	line*coccidia	2	2	74991.17	24197.63	40
4740	line*coccidia	2	3	119433.8	24197.63	40
4741	line	1		89632.54	16923.93	40
4741	line	2		105490.7	16923.93	40
4741	coccidia		0	99323.83	23934.05	40
4741	coccidia		1	84680.25	23934.05	40
4741	coccidia		2	84009.83	23934.05	40
4741	coccidia		3	122232.6	23934.05	40
4741	line*coccidia	1	0	104580.7	33847.86	40
4741	line*coccidia	1	1	43704	33847.86	40
4741	line*coccidia	1	2	89258.83	33847.86	40
4741	line*coccidia	1	3	120986.7	33847.86	40
4741	line*coccidia	2	0	94067	33847.86	40
4741	line*coccidia	2	1	125656.5	33847.86	40
4741	line*coccidia	2	2	78760.83	33847.86	40
4741	line*coccidia	2	3	123478.5	33847.86	40
4803	line	1		596005.8	144737.7	40
4803	line	2		581717.7	144737.7	40
4803	coccidia		0	779378.5	204690.1	40
4803	coccidia		1	414498.1	204690.1	40
4803	coccidia		2	485767.5	204690.1	40
4803	coccidia		3	675802.9	204690.1	40
4803	line*coccidia	1	0	828351.8	289475.5	40
4803	line*coccidia	1	1	312065.5	289475.5	40
4803	line*coccidia	1	2	391869.3	289475.5	40
4803	line*coccidia	1	3	851736.5	289475.5	40
4803	line*coccidia	2	0	730405.2	289475.5	40
4803	line*coccidia	2	1	516930.7	289475.5	40
4803	line*coccidia	2	2	579665.7	289475.5	40
4803	line*coccidia	2	3	499869.3	289475.5	40
4805	line	1		663316.9	136805.5	40
4805	line	2		463080.6	136805.5	40
4805	coccidia		0	537974.8	193472.2	40
4805	coccidia		1	360628.3	193472.2	40
4805	coccidia		2	748279	193472.2	40
4805	coccidia		3	605912.9	193472.2	40
4805	line*coccidia	1	0	517628.7	273610.9	40
4805	line*coccidia	1	1	546610.3	273610.9	40
4805	line*coccidia	1	2	806092.3	273610.9	40

4805	line*coccidia	1	3	782936.3	273610.9	40
4805	line*coccidia	2	0	558320.8	273610.9	40
4805	line*coccidia	2	1	174646.3	273610.9	40
4805	line*coccidia	2	2	690465.7	273610.9	40
4805	line*coccidia	2	3	428889.5	273610.9	40
4806	line	1		974541.8	291826.3	40
4806	line	2		605230	291826.3	40
4806	coccidia		0	925221	412704.7	40
4806	coccidia		1	253097.2	412704.7	40
4806	coccidia		2	1001247	412704.7	40
4806	coccidia		3	979978.2	412704.7	40
4806	line*coccidia	1	0	284733.8	583652.7	40
4806	line*coccidia	1	1	319665.2	583652.7	40
4806	line*coccidia	1	2	1438810	583652.7	40
4806	line*coccidia	1	3	1854959	583652.7	40
4806	line*coccidia	2	0	1565708	583652.7	40
4806	line*coccidia	2	1	186529.2	583652.7	40
4806	line*coccidia	2	2	563685.2	583652.7	40
4806	line*coccidia	2	3	104997.5	583652.7	40
4808	line	1		772925.7	181102.3	40
4808	line	2		490510.7	181102.3	40
4808	coccidia		0	455270.6	256117.4	40
4808	coccidia		1	620253.3	256117.4	40
4808	coccidia		2	870449.5	256117.4	40
4808	coccidia		3	580899.3	256117.4	40
4808	line*coccidia	1	0	509958	362204.6	40
4808	line*coccidia	1	1	608991.7	362204.6	40
4808	line*coccidia	1	2	895700.2	362204.6	40
4808	line*coccidia	1	3	1077053	362204.6	40
4808	line*coccidia	2	0	400583.2	362204.6	40
4808	line*coccidia	2	1	631515	362204.6	40
4808	line*coccidia	2	2	845198.8	362204.6	40
4808	line*coccidia	2	3	84745.83	362204.6	40
4810	line	1		866230.7	184437.6	40
4810	line	2		566992.1	184437.6	40
4810	coccidia		0	451217.7	260834.2	40
4810	coccidia		1	494833.7	260834.2	40
4810	coccidia		2	1189726	260834.2	40
4810	coccidia		3	730668.8	260834.2	40
4810	line*coccidia	1	0	328787.3	368875.2	40
4810	line*coccidia	1	1	750719.8	368875.2	40
4810	line*coccidia	1	2	1366361	368875.2	40
4810	line*coccidia	1	3	1019055	368875.2	40
4810	line*coccidia	2	0	573648	368875.2	40
4810	line*coccidia	2	1	238947.5	368875.2	40
4810	line*coccidia	2	2	1013090	368875.2	40
4810	line*coccidia	2	3	442282.8	368875.2	40
4816	line	1		695521	147147.9	40
4816	line	2		750556.3	147147.9	40

4816	coccidia		0	816053.8	208098.5	40
4816	coccidia		1	1051024	208098.5	40
4816	coccidia		2	432518.9	208098.5	40
4816	coccidia		3	592557.9	208098.5	40
4816	line*coccidia	1	0	795810	294295.7	40
4816	line*coccidia	1	1	709945.5	294295.7	40
4816	line*coccidia	1	2	437532.3	294295.7	40
4816	line*coccidia	1	3	838796	294295.7	40
4816	line*coccidia	2	0	836297.7	294295.7	40
4816	line*coccidia	2	1	1392102	294295.7	40
4816	line*coccidia	2	2	427505.5	294295.7	40
4816	line*coccidia	2	3	346319.8	294295.7	40
4817	line	1		391386.9	57977.47	40
4817	line	2		539179.6	57977.47	40
4817	coccidia		0	644749.3	81992.53	40
4817	coccidia		1	448633.8	81992.53	40
4817	coccidia		2	406278.1	81992.53	40
4817	coccidia		3	361471.9	81992.53	40
4817	line*coccidia	1	0	639760.3	115954.9	40
4817	line*coccidia	1	1	271000.7	115954.9	40
4817	line*coccidia	1	2	174519.7	115954.9	40
4817	line*coccidia	1	3	480267	115954.9	40
4817	line*coccidia	2	0	649738.3	115954.9	40
4817	line*coccidia	2	1	626266.8	115954.9	40
4817	line*coccidia	2	2	638036.5	115954.9	40
4817	line*coccidia	2	3	242676.8	115954.9	40
4821	line	1		278070.7	72399.42	40
4821	line	2		408577.7	72399.42	40
4821	coccidia		0	508273.4	102388.2	40
4821	coccidia		1	328749.7	102388.2	40
4821	coccidia		2	295351.7	102388.2	40
4821	coccidia		3	240922	102388.2	40
4821	line*coccidia	1	0	325600.7	144798.8	40
4821	line*coccidia	1	1	301952.5	144798.8	40
4821	line*coccidia	1	2	195208.2	144798.8	40
4821	line*coccidia	1	3	289521.3	144798.8	40
4821	line*coccidia	2	0	690946.2	144798.8	40
4821	line*coccidia	2	1	355546.8	144798.8	40
4821	line*coccidia	2	2	395495.2	144798.8	40
4821	line*coccidia	2	3	192322.7	144798.8	40
4822	line	1		258934	85308.67	40
4822	line	2		606291.3	85308.67	40
4822	coccidia		0	541508.9	120644.7	40
4822	coccidia		1	438778.3	120644.7	40
4822	coccidia		2	563104.7	120644.7	40
4822	coccidia		3	187058.7	120644.7	40
4822	line*coccidia	1	0	342266.5	170617.3	40
4822	line*coccidia	1	1	138954.3	170617.3	40
4822	line*coccidia	1	2	288620.2	170617.3	40

4822	line*coccidia	1	3	265894.8	170617.3	40
4822	line*coccidia	2	0	740751.3	170617.3	40
4822	line*coccidia	2	1	738602.2	170617.3	40
4822	line*coccidia	2	2	837589.2	170617.3	40
4822	line*coccidia	2	3	108222.5	170617.3	40
4823	line	1		1005191	195183.5	40
4823	line	2		1108155	195183.5	40
4823	coccidia		0	1489185	276031.1	40
4823	coccidia		1	1202405	276031.1	40
4823	coccidia		2	759523.1	276031.1	40
4823	coccidia		3	775579.4	276031.1	40
4823	line*coccidia	1	0	1600474	390367	40
4823	line*coccidia	1	1	1175906	390367	40
4823	line*coccidia	1	2	308859	390367	40
4823	line*coccidia	1	3	935525.2	390367	40
4823	line*coccidia	2	0	1377897	390367	40
4823	line*coccidia	2	1	1228903	390367	40
4823	line*coccidia	2	2	1210187	390367	40
4823	line*coccidia	2	3	615633.7	390367	40
4824	line	1		31510.88	7865.233	40
4824	line	2		32608.04	7865.233	40
4824	coccidia		0	45212.33	11123.12	40
4824	coccidia		1	34320.92	11123.12	40
4824	coccidia		2	6193.333	11123.12	40
4824	coccidia		3	42511.25	11123.12	40
4824	line*coccidia	1	0	50882.67	15730.47	40
4824	line*coccidia	1	1	25097	15730.47	40
4824	line*coccidia	1	2	-1.8E-12	15730.47	40
4824	line*coccidia	1	3	50063.83	15730.47	40
4824	line*coccidia	2	0	39542	15730.47	40
4824	line*coccidia	2	1	43544.83	15730.47	40
4824	line*coccidia	2	2	12386.67	15730.47	40
4824	line*coccidia	2	3	34958.67	15730.47	40
4826	line	1		35887.33	6795.892	40
4826	line	2		40283.08	6795.892	40
4826	coccidia		0	45676.67	9610.843	40
4826	coccidia		1	48735.92	9610.843	40
4826	coccidia		2	7064.833	9610.843	40
4826	coccidia		3	50863.42	9610.843	40
4826	line*coccidia	1	0	35277.33	13591.78	40
4826	line*coccidia	1	1	47998.33	13591.78	40
4826	line*coccidia	1	2	-7.3E-12	13591.78	40
4826	line*coccidia	1	3	60273.67	13591.78	40
4826	line*coccidia	2	0	56076	13591.78	40
4826	line*coccidia	2	1	49473.5	13591.78	40
4826	line*coccidia	2	2	14129.67	13591.78	40
4826	line*coccidia	2	3	41453.17	13591.78	40
4830	line	1		168883.5	36738.93	40
4830	line	2		140110.3	36738.93	40

4830	coccidia		0	227383.2	51956.69	40
4830	coccidia		1	191338.8	51956.69	40
4830	coccidia		2	63074.83	51956.69	40
4830	coccidia		3	136190.8	51956.69	40
4830	line*coccidia	1	0	384494.8	73477.86	40
4830	line*coccidia	1	1	58472	73477.86	40
4830	line*coccidia	1	2	22104.83	73477.86	40
4830	line*coccidia	1	3	210462.3	73477.86	40
4830	line*coccidia	2	0	70271.5	73477.86	40
4830	line*coccidia	2	1	324205.5	73477.86	40
4830	line*coccidia	2	2	104044.8	73477.86	40
4830	line*coccidia	2	3	61919.33	73477.86	40
4831	line	1		88056.67	19627.47	40
4831	line	2		80058.58	19627.47	40
4831	coccidia		0	177281	27757.43	40
4831	coccidia		1	32324.67	27757.43	40
4831	coccidia		2	59464.25	27757.43	40
4831	coccidia		3	67160.58	27757.43	40
4831	line*coccidia	1	0	146258.7	39254.93	40
4831	line*coccidia	1	1	33426.67	39254.93	40
4831	line*coccidia	1	2	89121.5	39254.93	40
4831	line*coccidia	1	3	83419.83	39254.93	40
4831	line*coccidia	2	0	208303.3	39254.93	40
4831	line*coccidia	2	1	31222.67	39254.93	40
4831	line*coccidia	2	2	29807	39254.93	40
4831	line*coccidia	2	3	50901.33	39254.93	40
4832	line	1		33458.83	8030.272	40
4832	line	2		38252.13	8030.272	40
4832	coccidia		0	54749.08	11356.52	40
4832	coccidia		1	23956.58	11356.52	40
4832	coccidia		2	15010.58	11356.52	40
4832	coccidia		3	49705.67	11356.52	40
4832	line*coccidia	1	0	56123.17	16060.54	40
4832	line*coccidia	1	1	9953	16060.54	40
4832	line*coccidia	1	2	-3.6E-12	16060.54	40
4832	line*coccidia	1	3	67759.17	16060.54	40
4832	line*coccidia	2	0	53375	16060.54	40
4832	line*coccidia	2	1	37960.17	16060.54	40
4832	line*coccidia	2	2	30021.17	16060.54	40
4832	line*coccidia	2	3	31652.17	16060.54	40
4833	line	1		136528	33281.63	40
4833	line	2		179413.7	33281.63	40
4833	coccidia		0	182457.5	47067.33	40
4833	coccidia		1	161890.8	47067.33	40
4833	coccidia		2	102850.2	47067.33	40
4833	coccidia		3	184684.8	47067.33	40
4833	line*coccidia	1	0	184624.8	66563.26	40
4833	line*coccidia	1	1	62012.67	66563.26	40
4833	line*coccidia	1	2	139914	66563.26	40

4833	line*coccidia	1	3	159560.3	66563.26	40
4833	line*coccidia	2	0	180290.2	66563.26	40
4833	line*coccidia	2	1	261769	66563.26	40
4833	line*coccidia	2	2	65786.33	66563.26	40
4833	line*coccidia	2	3	209809.3	66563.26	40
4835	line	1		89154	18584.1	40
4835	line	2		67588.17	18584.1	40
4835	coccidia		0	113184.4	26281.89	40
4835	coccidia		1	62855.25	26281.89	40
4835	coccidia		2	71245.92	26281.89	40
4835	coccidia		3	66198.75	26281.89	40
4835	line*coccidia	1	0	186762.8	37168.2	40
4835	line*coccidia	1	1	16868.17	37168.2	40
4835	line*coccidia	1	2	46918.33	37168.2	40
4835	line*coccidia	1	3	106066.7	37168.2	40
4835	line*coccidia	2	0	39606	37168.2	40
4835	line*coccidia	2	1	108842.3	37168.2	40
4835	line*coccidia	2	2	95573.5	37168.2	40
4835	line*coccidia	2	3	26330.83	37168.2	40
4837	line	1		52101.46	27153.71	40
4837	line	2		116429	27153.71	40
4837	coccidia		0	71724.17	38401.14	40
4837	coccidia		1	92919.08	38401.14	40
4837	coccidia		2	70300	38401.14	40
4837	coccidia		3	102117.7	38401.14	40
4837	line*coccidia	1	0	51239	54307.41	40
4837	line*coccidia	1	1	29353.17	54307.41	40
4837	line*coccidia	1	2	56357.33	54307.41	40
4837	line*coccidia	1	3	71456.33	54307.41	40
4837	line*coccidia	2	0	92209.33	54307.41	40
4837	line*coccidia	2	1	156485	54307.41	40
4837	line*coccidia	2	2	84242.67	54307.41	40
4837	line*coccidia	2	3	132779	54307.41	40
4901	line	1		299298.9	58671.36	40
4901	line	2		349918.5	58671.36	40
4901	coccidia		0	278380.2	82973.84	40
4901	coccidia		1	276336.1	82973.84	40
4901	coccidia		2	456589.1	82973.84	40
4901	coccidia		3	287129.4	82973.84	40
4901	line*coccidia	1	0	251826.7	117342.7	40
4901	line*coccidia	1	1	93339	117342.7	40
4901	line*coccidia	1	2	536481.5	117342.7	40
4901	line*coccidia	1	3	315548.3	117342.7	40
4901	line*coccidia	2	0	304933.7	117342.7	40
4901	line*coccidia	2	1	459333.2	117342.7	40
4901	line*coccidia	2	2	376696.7	117342.7	40
4901	line*coccidia	2	3	258710.5	117342.7	40
4902	line	1		308867	64710.68	40
4902	line	2		113810	64710.68	40

4902	coccidia		0	285502.6	91514.72	40
4902	coccidia		1	150109.1	91514.72	40
4902	coccidia		2	212917.2	91514.72	40
4902	coccidia		3	196825.1	91514.72	40
4902	line*coccidia	1	0	499826	129421.4	40
4902	line*coccidia	1	1	146858.5	129421.4	40
4902	line*coccidia	1	2	371914	129421.4	40
4902	line*coccidia	1	3	216869.5	129421.4	40
4902	line*coccidia	2	0	71179.17	129421.4	40
4902	line*coccidia	2	1	153359.7	129421.4	40
4902	line*coccidia	2	2	53920.33	129421.4	40
4902	line*coccidia	2	3	176780.7	129421.4	40
4903	line	1		415359.3	93295.75	40
4903	line	2		171575.2	93295.75	40
4903	coccidia		0	191434.8	131940.1	40
4903	coccidia		1	206823.6	131940.1	40
4903	coccidia		2	387300.8	131940.1	40
4903	coccidia		3	388309.7	131940.1	40
4903	line*coccidia	1	0	190958.5	186591.5	40
4903	line*coccidia	1	1	158425.8	186591.5	40
4903	line*coccidia	1	2	635858.2	186591.5	40
4903	line*coccidia	1	3	676194.5	186591.5	40
4903	line*coccidia	2	0	191911	186591.5	40
4903	line*coccidia	2	1	255221.3	186591.5	40
4903	line*coccidia	2	2	138743.5	186591.5	40
4903	line*coccidia	2	3	100424.8	186591.5	40
4911	line	1		307177.5	72688.34	40
4911	line	2		312781.4	72688.34	40
4911	coccidia		0	446784.1	102796.8	40
4911	coccidia		1	258652.5	102796.8	40
4911	coccidia		2	273539.7	102796.8	40
4911	coccidia		3	260941.7	102796.8	40
4911	line*coccidia	1	0	157676.7	145376.7	40
4911	line*coccidia	1	1	324577.3	145376.7	40
4911	line*coccidia	1	2	285377	145376.7	40
4911	line*coccidia	1	3	461079.2	145376.7	40
4911	line*coccidia	2	0	735891.5	145376.7	40
4911	line*coccidia	2	1	192727.7	145376.7	40
4911	line*coccidia	2	2	261702.3	145376.7	40
4911	line*coccidia	2	3	60804.17	145376.7	40
5003	line	1		55000.83	11167.21	40
5003	line	2		61280.17	11167.21	40
5003	coccidia		0	37688.42	15792.83	40
5003	coccidia		1	44902.58	15792.83	40
5003	coccidia		2	67269	15792.83	40
5003	coccidia		3	82702	15792.83	40
5003	line*coccidia	1	0	32958.83	22334.43	40
5003	line*coccidia	1	1	62323.17	22334.43	40
5003	line*coccidia	1	2	69721	22334.43	40

5003	line*coccidia	1	3	55000.33	22334.43	40
5003	line*coccidia	2	0	42418	22334.43	40
5003	line*coccidia	2	1	27482	22334.43	40
5003	line*coccidia	2	2	64817	22334.43	40
5003	line*coccidia	2	3	110403.7	22334.43	40
5005	line	1		1.05E-11	8971.279	40
5005	line	2		50440.58	8971.279	40
5005	coccidia		0	26327	12687.3	40
5005	coccidia		1	33226.83	12687.3	40
5005	coccidia		2	16074.42	12687.3	40
5005	coccidia		3	25252.92	12687.3	40
5005	line*coccidia	1	0	1.09E-11	17942.56	40
5005	line*coccidia	1	1	1.27E-11	17942.56	40
5005	line*coccidia	1	2	1.09E-11	17942.56	40
5005	line*coccidia	1	3	7.28E-12	17942.56	40
5005	line*coccidia	2	0	52654	17942.56	40
5005	line*coccidia	2	1	66453.67	17942.56	40
5005	line*coccidia	2	2	32148.83	17942.56	40
5005	line*coccidia	2	3	50505.83	17942.56	40
5006	line	1		29743.67	5126.93	40
5006	line	2		58685.04	5126.93	40
5006	coccidia		0	56272.08	7250.574	40
5006	coccidia		1	51259.17	7250.574	40
5006	coccidia		2	37730.83	7250.574	40
5006	coccidia		3	31595.33	7250.574	40
5006	line*coccidia	1	0	27585	10253.86	40
5006	line*coccidia	1	1	27422.17	10253.86	40
5006	line*coccidia	1	2	31512.83	10253.86	40
5006	line*coccidia	1	3	32454.67	10253.86	40
5006	line*coccidia	2	0	84959.17	10253.86	40
5006	line*coccidia	2	1	75096.17	10253.86	40
5006	line*coccidia	2	2	43948.83	10253.86	40
5006	line*coccidia	2	3	30736	10253.86	40
5007	line	1		16959371	629655.3	40
5007	line	2		19646696	629655.3	40
5007	coccidia		0	24064365	890467.1	40
5007	coccidia		1	13289361	890467.1	40
5007	coccidia		2	16337747	890467.1	40
5007	coccidia		3	19520661	890467.1	40
5007	line*coccidia	1	0	22159376	1259311	40
5007	line*coccidia	1	1	8140742	1259311	40
5007	line*coccidia	1	2	12122137	1259311	40
5007	line*coccidia	1	3	25415229	1259311	40
5007	line*coccidia	2	0	25969354	1259311	40
5007	line*coccidia	2	1	18437979	1259311	40
5007	line*coccidia	2	2	20553358	1259311	40
5007	line*coccidia	2	3	13626092	1259311	40
5008	line	1		512622.8	1032342	40
5008	line	2		2328388	1032342	40

5008	coccidia		0	744968.1	1459952	40
5008	coccidia		1	3593793	1459952	40
5008	coccidia		2	505991.3	1459952	40
5008	coccidia		3	837269.2	1459952	40
5008	line*coccidia	1	0	698627.3	2064684	40
5008	line*coccidia	1	1	252305	2064684	40
5008	line*coccidia	1	2	213101.2	2064684	40
5008	line*coccidia	1	3	886457.8	2064684	40
5008	line*coccidia	2	0	791308.8	2064684	40
5008	line*coccidia	2	1	6935282	2064684	40
5008	line*coccidia	2	2	798881.5	2064684	40
5008	line*coccidia	2	3	788080.5	2064684	40
5010	line	1		120267.4	29883.38	40
5010	line	2		113390.1	29883.38	40
5010	coccidia		0	73314.33	42261.48	40
5010	coccidia		1	198809.9	42261.48	40
5010	coccidia		2	92901.17	42261.48	40
5010	coccidia		3	102289.6	42261.48	40
5010	line*coccidia	1	0	91240.5	59766.76	40
5010	line*coccidia	1	1	200273.8	59766.76	40
5010	line*coccidia	1	2	48088.5	59766.76	40
5010	line*coccidia	1	3	141466.8	59766.76	40
5010	line*coccidia	2	0	55388.17	59766.76	40
5010	line*coccidia	2	1	197346	59766.76	40
5010	line*coccidia	2	2	137713.8	59766.76	40
5010	line*coccidia	2	3	63112.33	59766.76	40
5101	line	1		-1.8E-12	2139.026	40
5101	line	2		19002.83	2139.026	40
5101	coccidia		0	8203.167	3025.039	40
5101	coccidia		1	-1.8E-12	3025.039	40
5101	coccidia		2	29802.5	3025.039	40
5101	coccidia		3	-1.8E-12	3025.039	40
5101	line*coccidia	1	0	-1.8E-12	4278.051	40
5101	line*coccidia	1	1	-1.8E-12	4278.051	40
5101	line*coccidia	1	2	-1.8E-12	4278.051	40
5101	line*coccidia	1	3	-1.8E-12	4278.051	40
5101	line*coccidia	2	0	16406.33	4278.051	40
5101	line*coccidia	2	1	-1.8E-12	4278.051	40
5101	line*coccidia	2	2	59605	4278.051	40
5101	line*coccidia	2	3	-1.8E-12	4278.051	40
5102	line	1		142037.9	9997.054	40
5102	line	2		204914	9997.054	40
5102	coccidia		0	232130.4	14137.97	40
5102	coccidia		1	141108.5	14137.97	40
5102	coccidia		2	147980.4	14137.97	40
5102	coccidia		3	172684.6	14137.97	40
5102	line*coccidia	1	0	222703.5	19994.11	40
5102	line*coccidia	1	1	57396.33	19994.11	40
5102	line*coccidia	1	2	107188.3	19994.11	40

5102	line*coccidia	1	3	180863.5	19994.11	40
5102	line*coccidia	2	0	241557.3	19994.11	40
5102	line*coccidia	2	1	224820.7	19994.11	40
5102	line*coccidia	2	2	188772.5	19994.11	40
5102	line*coccidia	2	3	164505.7	19994.11	40
5103	line	1		279225.3	39735	40
5103	line	2		399784	39735	40
5103	coccidia		0	415074	56193.78	40
5103	coccidia		1	281724.5	56193.78	40
5103	coccidia		2	223789.8	56193.78	40
5103	coccidia		3	437430.3	56193.78	40
5103	line*coccidia	1	0	424797.5	79470.01	40
5103	line*coccidia	1	1	202568.8	79470.01	40
5103	line*coccidia	1	2	95059	79470.01	40
5103	line*coccidia	1	3	394476	79470.01	40
5103	line*coccidia	2	0	405350.5	79470.01	40
5103	line*coccidia	2	1	360880.2	79470.01	40
5103	line*coccidia	2	2	352520.7	79470.01	40
5103	line*coccidia	2	3	480384.7	79470.01	40
5105	line	1		728510.6	86729.72	40
5105	line	2		420308.3	86729.72	40
5105	coccidia		0	699106.4	122654.3	40
5105	coccidia		1	425636.2	122654.3	40
5105	coccidia		2	271781.5	122654.3	40
5105	coccidia		3	901113.6	122654.3	40
5105	line*coccidia	1	0	1003021	173459.4	40
5105	line*coccidia	1	1	183467.7	173459.4	40
5105	line*coccidia	1	2	267563	173459.4	40
5105	line*coccidia	1	3	1459991	173459.4	40
5105	line*coccidia	2	0	395191.8	173459.4	40
5105	line*coccidia	2	1	667804.7	173459.4	40
5105	line*coccidia	2	2	276000	173459.4	40
5105	line*coccidia	2	3	342236.5	173459.4	40
5106	line	1		343374.4	136511.9	40
5106	line	2		171494.5	136511.9	40
5106	coccidia		0	126298.3	193057	40
5106	coccidia		1	191311.7	193057	40
5106	coccidia		2	124229.1	193057	40
5106	coccidia		3	587898.7	193057	40
5106	line*coccidia	1	0	128649.7	273023.8	40
5106	line*coccidia	1	1	113351.5	273023.8	40
5106	line*coccidia	1	2	158393.7	273023.8	40
5106	line*coccidia	1	3	973102.7	273023.8	40
5106	line*coccidia	2	0	123946.8	273023.8	40
5106	line*coccidia	2	1	269271.8	273023.8	40
5106	line*coccidia	2	2	90064.5	273023.8	40
5106	line*coccidia	2	3	202694.7	273023.8	40
5107	line	1		2582149	208461.2	40
5107	line	2		2123986	208461.2	40

5107	coccidia		0	2620164	294808.7	40
5107	coccidia		1	1955011	294808.7	40
5107	coccidia		2	1631266	294808.7	40
5107	coccidia		3	3205829	294808.7	40
5107	line*coccidia	1	0	3292617	416922.4	40
5107	line*coccidia	1	1	1145225	416922.4	40
5107	line*coccidia	1	2	977054.5	416922.4	40
5107	line*coccidia	1	3	4913700	416922.4	40
5107	line*coccidia	2	0	1947711	416922.4	40
5107	line*coccidia	2	1	2764798	416922.4	40
5107	line*coccidia	2	2	2285477	416922.4	40
5107	line*coccidia	2	3	1497958	416922.4	40
5109	line	1		234062.2	17236.84	40
5109	line	2		279550	17236.84	40
5109	coccidia		0	267971.3	24376.58	40
5109	coccidia		1	287903.8	24376.58	40
5109	coccidia		2	182140.8	24376.58	40
5109	coccidia		3	289208.6	24376.58	40
5109	line*coccidia	1	0	275127.3	34473.69	40
5109	line*coccidia	1	1	208112.7	34473.69	40
5109	line*coccidia	1	2	139929.7	34473.69	40
5109	line*coccidia	1	3	313079	34473.69	40
5109	line*coccidia	2	0	260815.2	34473.69	40
5109	line*coccidia	2	1	367694.8	34473.69	40
5109	line*coccidia	2	2	224351.8	34473.69	40
5109	line*coccidia	2	3	265338.2	34473.69	40
5110	line	1		41876.13	11821.15	40
5110	line	2		79130.25	11821.15	40
5110	coccidia		0	87892.67	16717.63	40
5110	coccidia		1	48448.58	16717.63	40
5110	coccidia		2	41365.75	16717.63	40
5110	coccidia		3	64305.75	16717.63	40
5110	line*coccidia	1	0	48872.5	23642.3	40
5110	line*coccidia	1	1	17855.17	23642.3	40
5110	line*coccidia	1	2	33787.33	23642.3	40
5110	line*coccidia	1	3	66989.5	23642.3	40
5110	line*coccidia	2	0	126912.8	23642.3	40
5110	line*coccidia	2	1	79042	23642.3	40
5110	line*coccidia	2	2	48944.17	23642.3	40
5110	line*coccidia	2	3	61622	23642.3	40
5111	line	1		96280.42	18518.9	40
5111	line	2		147790.5	18518.9	40
5111	coccidia		0	161794.8	26189.68	40
5111	coccidia		1	103671.8	26189.68	40
5111	coccidia		2	87916.58	26189.68	40
5111	coccidia		3	134758.8	26189.68	40
5111	line*coccidia	1	0	166283.7	37037.81	40
5111	line*coccidia	1	1	2.91E-11	37037.81	40
5111	line*coccidia	1	2	47237.67	37037.81	40

5111	line*coccidia	1	3	171600.3	37037.81	40
5111	line*coccidia	2	0	157306	37037.81	40
5111	line*coccidia	2	1	207343.5	37037.81	40
5111	line*coccidia	2	2	128595.5	37037.81	40
5111	line*coccidia	2	3	97917.17	37037.81	40
5112	line	1		125463	15756.79	40
5112	line	2		149007.6	15756.79	40
5112	coccidia		0	165984.2	22283.46	40
5112	coccidia		1	150737.8	22283.46	40
5112	coccidia		2	102021.7	22283.46	40
5112	coccidia		3	130197.7	22283.46	40
5112	line*coccidia	1	0	130512.3	31513.57	40
5112	line*coccidia	1	1	125259.5	31513.57	40
5112	line*coccidia	1	2	93287	31513.57	40
5112	line*coccidia	1	3	152793.3	31513.57	40
5112	line*coccidia	2	0	201456	31513.57	40
5112	line*coccidia	2	1	176216	31513.57	40
5112	line*coccidia	2	2	110756.3	31513.57	40
5112	line*coccidia	2	3	107602	31513.57	40
5113	line	1		534863.1	31938.26	40
5113	line	2		546605.7	31938.26	40
5113	coccidia		0	636118.8	45167.52	40
5113	coccidia		1	506625.9	45167.52	40
5113	coccidia		2	347829.1	45167.52	40
5113	coccidia		3	672363.7	45167.52	40
5113	line*coccidia	1	0	665907.3	63876.51	40
5113	line*coccidia	1	1	289321.7	63876.51	40
5113	line*coccidia	1	2	201665.2	63876.51	40
5113	line*coccidia	1	3	982558.2	63876.51	40
5113	line*coccidia	2	0	606330.3	63876.51	40
5113	line*coccidia	2	1	723930.2	63876.51	40
5113	line*coccidia	2	2	493993	63876.51	40
5113	line*coccidia	2	3	362169.2	63876.51	40
5114	line	1		30349.5	5628.532	40
5114	line	2		32251.96	5628.532	40
5114	coccidia		0	32349.92	7959.946	40
5114	coccidia		1	33101.5	7959.946	40
5114	coccidia		2	20366.92	7959.946	40
5114	coccidia		3	39384.58	7959.946	40
5114	line*coccidia	1	0	36232.67	11257.06	40
5114	line*coccidia	1	1	22489.67	11257.06	40
5114	line*coccidia	1	2	19308.5	11257.06	40
5114	line*coccidia	1	3	43367.17	11257.06	40
5114	line*coccidia	2	0	28467.17	11257.06	40
5114	line*coccidia	2	1	43713.33	11257.06	40
5114	line*coccidia	2	2	21425.33	11257.06	40
5114	line*coccidia	2	3	35402	11257.06	40
5115	line	1		79141.5	19824.8	40
5115	line	2		76641.29	19824.8	40

5115	coccidia		0	98806.25	28036.5	40
5115	coccidia		1	86529.17	28036.5	40
5115	coccidia		2	32423.17	28036.5	40
5115	coccidia		3	93807	28036.5	40
5115	line*coccidia	1	0	104414.7	39649.6	40
5115	line*coccidia	1	1	29572.67	39649.6	40
5115	line*coccidia	1	2	30937.5	39649.6	40
5115	line*coccidia	1	3	151641.2	39649.6	40
5115	line*coccidia	2	0	93197.83	39649.6	40
5115	line*coccidia	2	1	143485.7	39649.6	40
5115	line*coccidia	2	2	33908.83	39649.6	40
5115	line*coccidia	2	3	35972.83	39649.6	40
5118	line	1		161122.8	18798.72	40
5118	line	2		185539.5	18798.72	40
5118	coccidia		0	148014.2	26585.41	40
5118	coccidia		1	207481.1	26585.41	40
5118	coccidia		2	146165	26585.41	40
5118	coccidia		3	191664.3	26585.41	40
5118	line*coccidia	1	0	154805.3	37597.45	40
5118	line*coccidia	1	1	123711.3	37597.45	40
5118	line*coccidia	1	2	169093.7	37597.45	40
5118	line*coccidia	1	3	196881	37597.45	40
5118	line*coccidia	2	0	141223	37597.45	40
5118	line*coccidia	2	1	291250.8	37597.45	40
5118	line*coccidia	2	2	123236.3	37597.45	40
5118	line*coccidia	2	3	186447.7	37597.45	40
5119	line	1		69204.21	10262.78	40
5119	line	2		74632.42	10262.78	40
5119	coccidia		0	76277.33	14513.77	40
5119	coccidia		1	60613.75	14513.77	40
5119	coccidia		2	71519.08	14513.77	40
5119	coccidia		3	79263.08	14513.77	40
5119	line*coccidia	1	0	66427.33	20525.57	40
5119	line*coccidia	1	1	53859	20525.57	40
5119	line*coccidia	1	2	91834.67	20525.57	40
5119	line*coccidia	1	3	64695.83	20525.57	40
5119	line*coccidia	2	0	86127.33	20525.57	40
5119	line*coccidia	2	1	67368.5	20525.57	40
5119	line*coccidia	2	2	51203.5	20525.57	40
5119	line*coccidia	2	3	93830.33	20525.57	40
5121	line	1		577300	116184.7	40
5121	line	2		504463.2	116184.7	40
5121	coccidia		0	815133.8	164310	40
5121	coccidia		1	403044.3	164310	40
5121	coccidia		2	246629.2	164310	40
5121	coccidia		3	698719.3	164310	40
5121	line*coccidia	1	0	1105055	232369.4	40
5121	line*coccidia	1	1	306019.3	232369.4	40
5121	line*coccidia	1	2	352596.3	232369.4	40

5121	line*coccidia	1	3	545529.3	232369.4	40
5121	line*coccidia	2	0	525212.5	232369.4	40
5121	line*coccidia	2	1	500069.2	232369.4	40
5121	line*coccidia	2	2	140662	232369.4	40
5121	line*coccidia	2	3	851909.2	232369.4	40
5125	line	1		38257	9214.238	40
5125	line	2		0	9214.238	40
5125	coccidia		0	0	13030.9	40
5125	coccidia		1	23515.42	13030.9	40
5125	coccidia		2	52998.58	13030.9	40
5125	coccidia		3	0	13030.9	40
5125	line*coccidia	1	0	0	18428.48	40
5125	line*coccidia	1	1	47030.83	18428.48	40
5125	line*coccidia	1	2	105997.2	18428.48	40
5125	line*coccidia	1	3	0	18428.48	40
5125	line*coccidia	2	0	0	18428.48	40
5125	line*coccidia	2	1	0	18428.48	40
5125	line*coccidia	2	2	0	18428.48	40
5125	line*coccidia	2	3	0	18428.48	40
5126	line	1		1232546	147620.9	40
5126	line	2		925678.4	147620.9	40
5126	coccidia		0	1523133	208767.5	40
5126	coccidia		1	571821.3	208767.5	40
5126	coccidia		2	787807.4	208767.5	40
5126	coccidia		3	1433688	208767.5	40
5126	line*coccidia	1	0	1593207	295241.8	40
5126	line*coccidia	1	1	606437.8	295241.8	40
5126	line*coccidia	1	2	508011.5	295241.8	40
5126	line*coccidia	1	3	2222530	295241.8	40
5126	line*coccidia	2	0	1453060	295241.8	40
5126	line*coccidia	2	1	537204.7	295241.8	40
5126	line*coccidia	2	2	1067603	295241.8	40
5126	line*coccidia	2	3	644846	295241.8	40
5127	line	1		-2.9E-11	13325.41	40
5127	line	2		283824.4	13325.41	40
5127	coccidia		0	85777.25	18844.98	40
5127	coccidia		1	315204.4	18844.98	40
5127	coccidia		2	111571.9	18844.98	40
5127	coccidia		3	55095.17	18844.98	40
5127	line*coccidia	1	0	-2.9E-11	26650.82	40
5127	line*coccidia	1	1	-2.9E-11	26650.82	40
5127	line*coccidia	1	2	-2.9E-11	26650.82	40
5127	line*coccidia	1	3	-2.9E-11	26650.82	40
5127	line*coccidia	2	0	171554.5	26650.82	40
5127	line*coccidia	2	1	630408.8	26650.82	40
5127	line*coccidia	2	2	223143.8	26650.82	40
5127	line*coccidia	2	3	110190.3	26650.82	40
5128	line	1		507283.1	117802	40
5128	line	2		245177.7	117802	40

5128	coccidia		0	932335.6	166597.2	40
5128	coccidia		1	356213.9	166597.2	40
5128	coccidia		2	0	166597.2	40
5128	coccidia		3	216372.2	166597.2	40
5128	line*coccidia	1	0	1316705	235604	40
5128	line*coccidia	1	1	712427.8	235604	40
5128	line*coccidia	1	2	0	235604	40
5128	line*coccidia	1	3	5.82E-11	235604	40
5128	line*coccidia	2	0	547966.5	235604	40
5128	line*coccidia	2	1	0	235604	40
5128	line*coccidia	2	2	0	235604	40
5128	line*coccidia	2	3	432744.3	235604	40
5131	line	1		32663.17	12509.83	40
5131	line	2		43740.79	12509.83	40
5131	coccidia		0	29878.67	17691.57	40
5131	coccidia		1	91048.17	17691.57	40
5131	coccidia		2	9505.167	17691.57	40
5131	coccidia		3	22375.92	17691.57	40
5131	line*coccidia	1	0	3.64E-12	25019.66	40
5131	line*coccidia	1	1	85900.83	25019.66	40
5131	line*coccidia	1	2	0	25019.66	40
5131	line*coccidia	1	3	44751.83	25019.66	40
5131	line*coccidia	2	0	59757.33	25019.66	40
5131	line*coccidia	2	1	96195.5	25019.66	40
5131	line*coccidia	2	2	19010.33	25019.66	40
5131	line*coccidia	2	3	-1.8E-11	25019.66	40
5133	line	1		538977.9	92453.48	40
5133	line	2		314363.7	92453.48	40
5133	coccidia		0	471530.7	130749	40
5133	coccidia		1	504596.8	130749	40
5133	coccidia		2	350074.7	130749	40
5133	coccidia		3	380481.1	130749	40
5133	line*coccidia	1	0	462568.2	184907	40
5133	line*coccidia	1	1	726073.7	184907	40
5133	line*coccidia	1	2	373535.8	184907	40
5133	line*coccidia	1	3	593733.8	184907	40
5133	line*coccidia	2	0	480493.2	184907	40
5133	line*coccidia	2	1	283119.8	184907	40
5133	line*coccidia	2	2	326613.5	184907	40
5133	line*coccidia	2	3	167228.3	184907	40
5134	line	1		3930.208	3250.831	40
5134	line	2		20351.75	3250.831	40
5134	coccidia		0	3284.917	4597.369	40
5134	coccidia		1	23231.75	4597.369	40
5134	coccidia		2	3801.083	4597.369	40
5134	coccidia		3	18246.17	4597.369	40
5134	line*coccidia	1	0	3.64E-12	6501.662	40
5134	line*coccidia	1	1	15720.83	6501.662	40
5134	line*coccidia	1	2	0	6501.662	40

5134	line*coccidia	1	3	0	6501.662	40
5134	line*coccidia	2	0	6569.833	6501.662	40
5134	line*coccidia	2	1	30742.67	6501.662	40
5134	line*coccidia	2	2	7602.167	6501.662	40
5134	line*coccidia	2	3	36492.33	6501.662	40
5201	line	1		578742.1	55205.07	40
5201	line	2		565628.6	55205.07	40
5201	coccidia		0	610438.4	78071.76	40
5201	coccidia		1	576646.1	78071.76	40
5201	coccidia		2	487481.1	78071.76	40
5201	coccidia		3	614175.8	78071.76	40
5201	line*coccidia	1	0	730926.8	110410.1	40
5201	line*coccidia	1	1	312107.8	110410.1	40
5201	line*coccidia	1	2	463407.7	110410.1	40
5201	line*coccidia	1	3	808526	110410.1	40
5201	line*coccidia	2	0	489950	110410.1	40
5201	line*coccidia	2	1	841184.3	110410.1	40
5201	line*coccidia	2	2	511554.5	110410.1	40
5201	line*coccidia	2	3	419825.7	110410.1	40
5202	line	1		30631.21	12156.29	40
5202	line	2		40519.88	12156.29	40
5202	coccidia		0	21183.25	17191.58	40
5202	coccidia		1	28812.5	17191.58	40
5202	coccidia		2	31915.92	17191.58	40
5202	coccidia		3	60390.5	17191.58	40
5202	line*coccidia	1	0	22282.67	24312.57	40
5202	line*coccidia	1	1	19124.83	24312.57	40
5202	line*coccidia	1	2	46503	24312.57	40
5202	line*coccidia	1	3	34614.33	24312.57	40
5202	line*coccidia	2	0	20083.83	24312.57	40
5202	line*coccidia	2	1	38500.17	24312.57	40
5202	line*coccidia	2	2	17328.83	24312.57	40
5202	line*coccidia	2	3	86166.67	24312.57	40
5203	line	1		577288.9	76937.74	40
5203	line	2		603977.6	76937.74	40
5203	coccidia		0	779184.7	108806.4	40
5203	coccidia		1	443017.2	108806.4	40
5203	coccidia		2	410904.8	108806.4	40
5203	coccidia		3	729426.3	108806.4	40
5203	line*coccidia	1	0	933418	153875.5	40
5203	line*coccidia	1	1	172435.3	153875.5	40
5203	line*coccidia	1	2	400806.7	153875.5	40
5203	line*coccidia	1	3	802495.5	153875.5	40
5203	line*coccidia	2	0	624951.3	153875.5	40
5203	line*coccidia	2	1	713599	153875.5	40
5203	line*coccidia	2	2	421003	153875.5	40
5203	line*coccidia	2	3	656357.2	153875.5	40
5204	line	1		51712.92	19169.19	40
5204	line	2		184863.8	19169.19	40

5204	coccidia		0	132659.3	27109.33	40
5204	coccidia		1	146709.8	27109.33	40
5204	coccidia		2	52780.17	27109.33	40
5204	coccidia		3	141004.3	27109.33	40
5204	line*coccidia	1	0	82602.67	38338.38	40
5204	line*coccidia	1	1	16914	38338.38	40
5204	line*coccidia	1	2	25587.5	38338.38	40
5204	line*coccidia	1	3	81747.5	38338.38	40
5204	line*coccidia	2	0	182715.8	38338.38	40
5204	line*coccidia	2	1	276505.5	38338.38	40
5204	line*coccidia	2	2	79972.83	38338.38	40
5204	line*coccidia	2	3	200261.2	38338.38	40
5205	line	1		817980.3	41812.4	40
5205	line	2		853655	41812.4	40
5205	coccidia		0	1301860	59131.66	40
5205	coccidia		1	697723	59131.66	40
5205	coccidia		2	557024.9	59131.66	40
5205	coccidia		3	786662.8	59131.66	40
5205	line*coccidia	1	0	1435305	83624.79	40
5205	line*coccidia	1	1	361476	83624.79	40
5205	line*coccidia	1	2	412488	83624.79	40
5205	line*coccidia	1	3	1062653	83624.79	40
5205	line*coccidia	2	0	1168415	83624.79	40
5205	line*coccidia	2	1	1033970	83624.79	40
5205	line*coccidia	2	2	701561.8	83624.79	40
5205	line*coccidia	2	3	510673	83624.79	40
5206	line	1		52320.83	91325.93	40
5206	line	2		223106.3	91325.93	40
5206	coccidia		0	87542.92	129154.4	40
5206	coccidia		1	329789.6	129154.4	40
5206	coccidia		2	51776.83	129154.4	40
5206	coccidia		3	81744.83	129154.4	40
5206	line*coccidia	1	0	59069.83	182651.9	40
5206	line*coccidia	1	1	53839.67	182651.9	40
5206	line*coccidia	1	2	46714	182651.9	40
5206	line*coccidia	1	3	49659.83	182651.9	40
5206	line*coccidia	2	0	116016	182651.9	40
5206	line*coccidia	2	1	605739.5	182651.9	40
5206	line*coccidia	2	2	56839.67	182651.9	40
5206	line*coccidia	2	3	113829.8	182651.9	40
5207	line	1		43012.67	57470.93	40
5207	line	2		120015.2	57470.93	40
5207	coccidia		0	33008.75	81276.17	40
5207	coccidia		1	214779.3	81276.17	40
5207	coccidia		2	30098.75	81276.17	40
5207	coccidia		3	48169	81276.17	40
5207	line*coccidia	1	0	36808.17	114941.9	40
5207	line*coccidia	1	1	46907.17	114941.9	40
5207	line*coccidia	1	2	33404.17	114941.9	40

5207	line*coccidia	1	3	54931.17	114941.9	40
5207	line*coccidia	2	0	29209.33	114941.9	40
5207	line*coccidia	2	1	382651.3	114941.9	40
5207	line*coccidia	2	2	26793.33	114941.9	40
5207	line*coccidia	2	3	41406.83	114941.9	40
5208	line	1		35066.38	5478.895	40
5208	line	2		38684.88	5478.895	40
5208	coccidia		0	39041.33	7748.327	40
5208	coccidia		1	34875.75	7748.327	40
5208	coccidia		2	28343.42	7748.327	40
5208	coccidia		3	45242	7748.327	40
5208	line*coccidia	1	0	37962	10957.79	40
5208	line*coccidia	1	1	23726.5	10957.79	40
5208	line*coccidia	1	2	30049.5	10957.79	40
5208	line*coccidia	1	3	48527.5	10957.79	40
5208	line*coccidia	2	0	40120.67	10957.79	40
5208	line*coccidia	2	1	46025	10957.79	40
5208	line*coccidia	2	2	26637.33	10957.79	40
5208	line*coccidia	2	3	41956.5	10957.79	40
5210	line	1		205504.7	24511.78	40
5210	line	2		154818.4	24511.78	40
5210	coccidia		0	224679	34664.9	40
5210	coccidia		1	227004	34664.9	40
5210	coccidia		2	90532.33	34664.9	40
5210	coccidia		3	178430.8	34664.9	40
5210	line*coccidia	1	0	285064	49023.57	40
5210	line*coccidia	1	1	148142	49023.57	40
5210	line*coccidia	1	2	109551.7	49023.57	40
5210	line*coccidia	1	3	279261.2	49023.57	40
5210	line*coccidia	2	0	164294	49023.57	40
5210	line*coccidia	2	1	305866	49023.57	40
5210	line*coccidia	2	2	71513	49023.57	40
5210	line*coccidia	2	3	77600.5	49023.57	40
5212	line	1		46956.75	8521.044	40
5212	line	2		30742.96	8521.044	40
5212	coccidia		0	50768.5	12050.58	40
5212	coccidia		1	38132.75	12050.58	40
5212	coccidia		2	25842.67	12050.58	40
5212	coccidia		3	40655.5	12050.58	40
5212	line*coccidia	1	0	36923.33	17042.09	40
5212	line*coccidia	1	1	76265.5	17042.09	40
5212	line*coccidia	1	2	25891	17042.09	40
5212	line*coccidia	1	3	48747.17	17042.09	40
5212	line*coccidia	2	0	64613.67	17042.09	40
5212	line*coccidia	2	1	-7.3E-12	17042.09	40
5212	line*coccidia	2	2	25794.33	17042.09	40
5212	line*coccidia	2	3	32563.83	17042.09	40
5216	line	1		5797.958	1331.906	40
5216	line	2		10156.88	1331.906	40

5216	coccidia		0	9485	1883.599	40
5216	coccidia		1	9412.167	1883.599	40
5216	coccidia		2	7456.917	1883.599	40
5216	coccidia		3	5555.583	1883.599	40
5216	line*coccidia	1	0	12080.67	2663.811	40
5216	line*coccidia	1	1	0	2663.811	40
5216	line*coccidia	1	2	0	2663.811	40
5216	line*coccidia	1	3	11111.17	2663.811	40
5216	line*coccidia	2	0	6889.333	2663.811	40
5216	line*coccidia	2	1	18824.33	2663.811	40
5216	line*coccidia	2	2	14913.83	2663.811	40
5216	line*coccidia	2	3	1.82E-12	2663.811	40
5217	line	1		24543	65773.32	40
5217	line	2		159142.9	65773.32	40
5217	coccidia		0	56927	93017.53	40
5217	coccidia		1	48515.08	93017.53	40
5217	coccidia		2	29748.5	93017.53	40
5217	coccidia		3	232181.3	93017.53	40
5217	line*coccidia	1	0	20724.67	131546.6	40
5217	line*coccidia	1	1	15181.33	131546.6	40
5217	line*coccidia	1	2	22591.33	131546.6	40
5217	line*coccidia	1	3	39674.67	131546.6	40
5217	line*coccidia	2	0	93129.33	131546.6	40
5217	line*coccidia	2	1	81848.83	131546.6	40
5217	line*coccidia	2	2	36905.67	131546.6	40
5217	line*coccidia	2	3	424687.8	131546.6	40
5218	line	1		48223.17	6738.326	40
5218	line	2		68060.25	6738.326	40
5218	coccidia		0	72460.83	9529.432	40
5218	coccidia		1	65865.08	9529.432	40
5218	coccidia		2	8853.667	9529.432	40
5218	coccidia		3	85387.25	9529.432	40
5218	line*coccidia	1	0	106774.5	13476.65	40
5218	line*coccidia	1	1	21450.67	13476.65	40
5218	line*coccidia	1	2	1.09E-11	13476.65	40
5218	line*coccidia	1	3	64667.5	13476.65	40
5218	line*coccidia	2	0	38147.17	13476.65	40
5218	line*coccidia	2	1	110279.5	13476.65	40
5218	line*coccidia	2	2	17707.33	13476.65	40
5218	line*coccidia	2	3	106107	13476.65	40
5301	line	1		27951.79	5010.556	40
5301	line	2		22027.5	5010.556	40
5301	coccidia		0	42052.58	7085.996	40
5301	coccidia		1	0	7085.996	40
5301	coccidia		2	4170.167	7085.996	40
5301	coccidia		3	53735.83	7085.996	40
5301	line*coccidia	1	0	48962.67	10021.11	40
5301	line*coccidia	1	1	0	10021.11	40
5301	line*coccidia	1	2	-7.3E-12	10021.11	40

5301	line*coccidia	1	3	62844.5	10021.11	40
5301	line*coccidia	2	0	35142.5	10021.11	40
5301	line*coccidia	2	1	0	10021.11	40
5301	line*coccidia	2	2	8340.333	10021.11	40
5301	line*coccidia	2	3	44627.17	10021.11	40
5302	line	1		95424.88	11395.35	40
5302	line	2		151649	11395.35	40
5302	coccidia		0	141927.6	16115.45	40
5302	coccidia		1	122564	16115.45	40
5302	coccidia		2	110992.5	16115.45	40
5302	coccidia		3	118663.6	16115.45	40
5302	line*coccidia	1	0	128559.3	22790.69	40
5302	line*coccidia	1	1	68042.5	22790.69	40
5302	line*coccidia	1	2	39840	22790.69	40
5302	line*coccidia	1	3	145257.7	22790.69	40
5302	line*coccidia	2	0	155295.8	22790.69	40
5302	line*coccidia	2	1	177085.5	22790.69	40
5302	line*coccidia	2	2	182145	22790.69	40
5302	line*coccidia	2	3	92069.5	22790.69	40
5304	line	1		67998.88	25669.15	40
5304	line	2		128464.8	25669.15	40
5304	coccidia		0	154772.5	36301.66	40
5304	coccidia		1	110208.3	36301.66	40
5304	coccidia		2	32010.67	36301.66	40
5304	coccidia		3	95935.92	36301.66	40
5304	line*coccidia	1	0	85580.83	51338.3	40
5304	line*coccidia	1	1	87908.5	51338.3	40
5304	line*coccidia	1	2	3.64E-12	51338.3	40
5304	line*coccidia	1	3	98506.17	51338.3	40
5304	line*coccidia	2	0	223964.2	51338.3	40
5304	line*coccidia	2	1	132508	51338.3	40
5304	line*coccidia	2	2	64021.33	51338.3	40
5304	line*coccidia	2	3	93365.67	51338.3	40
5305	line	1		372746.9	148712.7	40
5305	line	2		515379.3	148712.7	40
5305	coccidia		0	959392.1	210311.5	40
5305	coccidia		1	248312.7	210311.5	40
5305	coccidia		2	218707.2	210311.5	40
5305	coccidia		3	349840.4	210311.5	40
5305	line*coccidia	1	0	708924.8	297425.4	40
5305	line*coccidia	1	1	39031.17	297425.4	40
5305	line*coccidia	1	2	156684.3	297425.4	40
5305	line*coccidia	1	3	586347.2	297425.4	40
5305	line*coccidia	2	0	1209859	297425.4	40
5305	line*coccidia	2	1	457594.2	297425.4	40
5305	line*coccidia	2	2	280730	297425.4	40
5305	line*coccidia	2	3	113333.7	297425.4	40
5308	line	1		156179.1	24431.59	40
5308	line	2		246964	24431.59	40

5308	coccidia		0	195457.1	34551.48	40
5308	coccidia		1	191326.9	34551.48	40
5308	coccidia		2	184448	34551.48	40
5308	coccidia		3	235054.2	34551.48	40
5308	line*coccidia	1	0	169423	48863.17	40
5308	line*coccidia	1	1	11650	48863.17	40
5308	line*coccidia	1	2	151310.2	48863.17	40
5308	line*coccidia	1	3	292333.2	48863.17	40
5308	line*coccidia	2	0	221491.2	48863.17	40
5308	line*coccidia	2	1	371003.8	48863.17	40
5308	line*coccidia	2	2	217585.8	48863.17	40
5308	line*coccidia	2	3	177775.2	48863.17	40
5309	line	1		591868.7	39865.65	40
5309	line	2		672632.7	39865.65	40
5309	coccidia		0	954929.8	56378.55	40
5309	coccidia		1	576099.9	56378.55	40
5309	coccidia		2	384879.5	56378.55	40
5309	coccidia		3	613093.5	56378.55	40
5309	line*coccidia	1	0	892849.5	79731.31	40
5309	line*coccidia	1	1	269115	79731.31	40
5309	line*coccidia	1	2	357125.2	79731.31	40
5309	line*coccidia	1	3	848385	79731.31	40
5309	line*coccidia	2	0	1017010	79731.31	40
5309	line*coccidia	2	1	883084.8	79731.31	40
5309	line*coccidia	2	2	412633.8	79731.31	40
5309	line*coccidia	2	3	377802	79731.31	40
5310	line	1		92735.71	11724.33	40
5310	line	2		118695.7	11724.33	40
5310	coccidia		0	135123.9	16580.7	40
5310	coccidia		1	70133.08	16580.7	40
5310	coccidia		2	73079.83	16580.7	40
5310	coccidia		3	144526	16580.7	40
5310	line*coccidia	1	0	124721.2	23448.66	40
5310	line*coccidia	1	1	22635.83	23448.66	40
5310	line*coccidia	1	2	53408.33	23448.66	40
5310	line*coccidia	1	3	170177.5	23448.66	40
5310	line*coccidia	2	0	145526.7	23448.66	40
5310	line*coccidia	2	1	117630.3	23448.66	40
5310	line*coccidia	2	2	92751.33	23448.66	40
5310	line*coccidia	2	3	118874.5	23448.66	40
5311	line	1		50570.5	15240.01	40
5311	line	2		60290.29	15240.01	40
5311	coccidia		0	93527.08	21552.63	40
5311	coccidia		1	61769.25	21552.63	40
5311	coccidia		2	26834	21552.63	40
5311	coccidia		3	39591.25	21552.63	40
5311	line*coccidia	1	0	86792.5	30480.03	40
5311	line*coccidia	1	1	41037.33	30480.03	40
5311	line*coccidia	1	2	21230.5	30480.03	40

5311	line*coccidia	1	3	53221.67	30480.03	40
5311	line*coccidia	2	0	100261.7	30480.03	40
5311	line*coccidia	2	1	82501.17	30480.03	40
5311	line*coccidia	2	2	32437.5	30480.03	40
5311	line*coccidia	2	3	25960.83	30480.03	40
5312	line	1		253935.9	18660.52	40
5312	line	2		256743.8	18660.52	40
5312	coccidia		0	278649.2	26389.96	40
5312	coccidia		1	265985.7	26389.96	40
5312	coccidia		2	224170.3	26389.96	40
5312	coccidia		3	252554.4	26389.96	40
5312	line*coccidia	1	0	318830.8	37321.04	40
5312	line*coccidia	1	1	140419.7	37321.04	40
5312	line*coccidia	1	2	140966.2	37321.04	40
5312	line*coccidia	1	3	415527	37321.04	40
5312	line*coccidia	2	0	238467.5	37321.04	40
5312	line*coccidia	2	1	391551.7	37321.04	40
5312	line*coccidia	2	2	307374.3	37321.04	40
5312	line*coccidia	2	3	89581.83	37321.04	40
5313	line	1		166701.6	22234.12	40
5313	line	2		106245.6	22234.12	40
5313	coccidia		0	175702.2	31443.8	40
5313	coccidia		1	116169	31443.8	40
5313	coccidia		2	73779.92	31443.8	40
5313	coccidia		3	180243.4	31443.8	40
5313	line*coccidia	1	0	274842.3	44468.25	40
5313	line*coccidia	1	1	52936.67	44468.25	40
5313	line*coccidia	1	2	22142.17	44468.25	40
5313	line*coccidia	1	3	316885.3	44468.25	40
5313	line*coccidia	2	0	76562	44468.25	40
5313	line*coccidia	2	1	179401.3	44468.25	40
5313	line*coccidia	2	2	125417.7	44468.25	40
5313	line*coccidia	2	3	43601.5	44468.25	40
5314	line	1		228840	23908.97	40
5314	line	2		186516.8	23908.97	40
5314	coccidia		0	372471.4	33812.38	40
5314	coccidia		1	198001.7	33812.38	40
5314	coccidia		2	86598.83	33812.38	40
5314	coccidia		3	173641.8	33812.38	40
5314	line*coccidia	1	0	361558.5	47817.93	40
5314	line*coccidia	1	1	238615	47817.93	40
5314	line*coccidia	1	2	95950.33	47817.93	40
5314	line*coccidia	1	3	219236.3	47817.93	40
5314	line*coccidia	2	0	383384.3	47817.93	40
5314	line*coccidia	2	1	157388.3	47817.93	40
5314	line*coccidia	2	2	77247.33	47817.93	40
5314	line*coccidia	2	3	128047.3	47817.93	40
5315	line	1		49105.92	7966.345	40
5315	line	2		76995.13	7966.345	40

5315	coccidia		0	129126.7	11266.11	40
5315	coccidia		1	54713.67	11266.11	40
5315	coccidia		2	35445.17	11266.11	40
5315	coccidia		3	32916.58	11266.11	40
5315	line*coccidia	1	0	96984	15932.69	40
5315	line*coccidia	1	1	30555	15932.69	40
5315	line*coccidia	1	2	40129.17	15932.69	40
5315	line*coccidia	1	3	28755.5	15932.69	40
5315	line*coccidia	2	0	161269.3	15932.69	40
5315	line*coccidia	2	1	78872.33	15932.69	40
5315	line*coccidia	2	2	30761.17	15932.69	40
5315	line*coccidia	2	3	37077.67	15932.69	40
5317	line	1		216457.6	27401.42	40
5317	line	2		206061	27401.42	40
5317	coccidia		0	355261.4	38751.46	40
5317	coccidia		1	113334.8	38751.46	40
5317	coccidia		2	101714.2	38751.46	40
5317	coccidia		3	274726.8	38751.46	40
5317	line*coccidia	1	0	362015.3	54802.84	40
5317	line*coccidia	1	1	30514.5	54802.84	40
5317	line*coccidia	1	2	90685.83	54802.84	40
5317	line*coccidia	1	3	382614.7	54802.84	40
5317	line*coccidia	2	0	348507.5	54802.84	40
5317	line*coccidia	2	1	196155.2	54802.84	40
5317	line*coccidia	2	2	112742.5	54802.84	40
5317	line*coccidia	2	3	166838.8	54802.84	40
5318	line	1		145827.3	41080.07	40
5318	line	2		209831.7	41080.07	40
5318	coccidia		0	158822.2	58095.99	40
5318	coccidia		1	205364.3	58095.99	40
5318	coccidia		2	169378.7	58095.99	40
5318	coccidia		3	177752.9	58095.99	40
5318	line*coccidia	1	0	170635.7	82160.14	40
5318	line*coccidia	1	1	42434	82160.14	40
5318	line*coccidia	1	2	78395.5	82160.14	40
5318	line*coccidia	1	3	291844	82160.14	40
5318	line*coccidia	2	0	147008.7	82160.14	40
5318	line*coccidia	2	1	368294.5	82160.14	40
5318	line*coccidia	2	2	260361.8	82160.14	40
5318	line*coccidia	2	3	63661.83	82160.14	40
5319	line	1		106915.4	161616.6	40
5319	line	2		366513.9	161616.6	40
5319	coccidia		0	540918.3	228560.3	40
5319	coccidia		1	277929.2	228560.3	40
5319	coccidia		2	55226.42	228560.3	40
5319	coccidia		3	72784.67	228560.3	40
5319	line*coccidia	1	0	144739.3	323233.1	40
5319	line*coccidia	1	1	108622.3	323233.1	40
5319	line*coccidia	1	2	89154.17	323233.1	40

5319	line*coccidia	1	3	85145.67	323233.1	40
5319	line*coccidia	2	0	937097.2	323233.1	40
5319	line*coccidia	2	1	447236	323233.1	40
5319	line*coccidia	2	2	21298.67	323233.1	40
5319	line*coccidia	2	3	60423.67	323233.1	40
5320	line	1		46168.79	9204.646	40
5320	line	2		47992.54	9204.646	40
5320	coccidia		0	56708	13017.34	40
5320	coccidia		1	36359.92	13017.34	40
5320	coccidia		2	33698.67	13017.34	40
5320	coccidia		3	61556.08	13017.34	40
5320	line*coccidia	1	0	68761.83	18409.29	40
5320	line*coccidia	1	1	7.28E-12	18409.29	40
5320	line*coccidia	1	2	30917.17	18409.29	40
5320	line*coccidia	1	3	84996.17	18409.29	40
5320	line*coccidia	2	0	44654.17	18409.29	40
5320	line*coccidia	2	1	72719.83	18409.29	40
5320	line*coccidia	2	2	36480.17	18409.29	40
5320	line*coccidia	2	3	38116	18409.29	40
5321	line	1		71023.33	16739.21	40
5321	line	2		166320.9	16739.21	40
5321	coccidia		0	126386.8	23672.82	40
5321	coccidia		1	153142	23672.82	40
5321	coccidia		2	90280.83	23672.82	40
5321	coccidia		3	104878.8	23672.82	40
5321	line*coccidia	1	0	119760.7	33478.43	40
5321	line*coccidia	1	1	-1.5E-11	33478.43	40
5321	line*coccidia	1	2	37503.67	33478.43	40
5321	line*coccidia	1	3	126829	33478.43	40
5321	line*coccidia	2	0	133012.8	33478.43	40
5321	line*coccidia	2	1	306284	33478.43	40
5321	line*coccidia	2	2	143058	33478.43	40
5321	line*coccidia	2	3	82928.67	33478.43	40
5322	line	1		48974.33	11042.99	40
5322	line	2		57787.17	11042.99	40
5322	coccidia		0	68657.75	15617.15	40
5322	coccidia		1	37348.75	15617.15	40
5322	coccidia		2	40283.33	15617.15	40
5322	coccidia		3	67233.17	15617.15	40
5322	line*coccidia	1	0	66657.83	22085.98	40
5322	line*coccidia	1	1	3.64E-12	22085.98	40
5322	line*coccidia	1	2	43200.67	22085.98	40
5322	line*coccidia	1	3	86038.83	22085.98	40
5322	line*coccidia	2	0	70657.67	22085.98	40
5322	line*coccidia	2	1	74697.5	22085.98	40
5322	line*coccidia	2	2	37366	22085.98	40
5322	line*coccidia	2	3	48427.5	22085.98	40
5323	line	1		106553.5	17591	40
5323	line	2		146120	17591	40

5323	coccidia		0	115410.8	24877.42	40
5323	coccidia		1	163685	24877.42	40
5323	coccidia		2	76807.92	24877.42	40
5323	coccidia		3	149443.4	24877.42	40
5323	line*coccidia	1	0	140703	35181.99	40
5323	line*coccidia	1	1	42753	35181.99	40
5323	line*coccidia	1	2	67785.67	35181.99	40
5323	line*coccidia	1	3	174972.3	35181.99	40
5323	line*coccidia	2	0	90118.5	35181.99	40
5323	line*coccidia	2	1	284617	35181.99	40
5323	line*coccidia	2	2	85830.17	35181.99	40
5323	line*coccidia	2	3	123914.5	35181.99	40
5325	line	1		151743.2	28111.4	40
5325	line	2		116645.3	28111.4	40
5325	coccidia		0	115297.5	39755.52	40
5325	coccidia		1	153271.9	39755.52	40
5325	coccidia		2	157567.2	39755.52	40
5325	coccidia		3	110640.5	39755.52	40
5325	line*coccidia	1	0	133694.5	56222.8	40
5325	line*coccidia	1	1	204004.3	56222.8	40
5325	line*coccidia	1	2	149858	56222.8	40
5325	line*coccidia	1	3	119416	56222.8	40
5325	line*coccidia	2	0	96900.5	56222.8	40
5325	line*coccidia	2	1	102539.5	56222.8	40
5325	line*coccidia	2	2	165276.3	56222.8	40
5325	line*coccidia	2	3	101865	56222.8	40
5326	line	1		30598.33	4969.689	40
5326	line	2		53159.08	4969.689	40
5326	coccidia		0	65152.67	7028.202	40
5326	coccidia		1	29444.42	7028.202	40
5326	coccidia		2	35448.83	7028.202	40
5326	coccidia		3	37468.92	7028.202	40
5326	line*coccidia	1	0	52560	9939.378	40
5326	line*coccidia	1	1	7.28E-12	9939.378	40
5326	line*coccidia	1	2	27020	9939.378	40
5326	line*coccidia	1	3	42813.33	9939.378	40
5326	line*coccidia	2	0	77745.33	9939.378	40
5326	line*coccidia	2	1	58888.83	9939.378	40
5326	line*coccidia	2	2	43877.67	9939.378	40
5326	line*coccidia	2	3	32124.5	9939.378	40
5327	line	1		-7.3E-12	37534.22	40
5327	line	2		260316.4	37534.22	40
5327	coccidia		0	236002.1	53081.41	40
5327	coccidia		1	199965.9	53081.41	40
5327	coccidia		2	84664.75	53081.41	40
5327	coccidia		3	0	53081.41	40
5327	line*coccidia	1	0	-2.2E-11	75068.45	40
5327	line*coccidia	1	1	3.64E-11	75068.45	40
5327	line*coccidia	1	2	-2.2E-11	75068.45	40

5327	line*coccidia	1	3	-2.2E-11	75068.45	40
5327	line*coccidia	2	0	472004.2	75068.45	40
5327	line*coccidia	2	1	399931.8	75068.45	40
5327	line*coccidia	2	2	169329.5	75068.45	40
5327	line*coccidia	2	3	2.18E-11	75068.45	40
5328	line	1		39284.75	8611.114	40
5328	line	2		35263.13	8611.114	40
5328	coccidia		0	55599.17	12177.95	40
5328	coccidia		1	0	12177.95	40
5328	coccidia		2	41826.67	12177.95	40
5328	coccidia		3	51669.92	12177.95	40
5328	line*coccidia	1	0	53799.17	17222.23	40
5328	line*coccidia	1	1	0	17222.23	40
5328	line*coccidia	1	2	0	17222.23	40
5328	line*coccidia	1	3	103339.8	17222.23	40
5328	line*coccidia	2	0	57399.17	17222.23	40
5328	line*coccidia	2	1	0	17222.23	40
5328	line*coccidia	2	2	83653.33	17222.23	40
5328	line*coccidia	2	3	0	17222.23	40
5330	line	1		37278.33	5245.714	40
5330	line	2		7769.208	5245.714	40
5330	coccidia		0	29571.75	7418.56	40
5330	coccidia		1	3269.333	7418.56	40
5330	coccidia		2	19772.08	7418.56	40
5330	coccidia		3	37481.92	7418.56	40
5330	line*coccidia	1	0	41735.67	10491.43	40
5330	line*coccidia	1	1	6538.667	10491.43	40
5330	line*coccidia	1	2	25875.17	10491.43	40
5330	line*coccidia	1	3	74963.83	10491.43	40
5330	line*coccidia	2	0	17407.83	10491.43	40
5330	line*coccidia	2	1	-3.6E-12	10491.43	40
5330	line*coccidia	2	2	13669	10491.43	40
5330	line*coccidia	2	3	1.82E-12	10491.43	40
5335	line	1		10437.63	19532.17	39
5335	line	2		53292.63	20014.51	39
5335	coccidia		0	47980.08	27622.65	39
5335	coccidia		1	-7.3E-12	27622.65	39
5335	coccidia		2	9305.25	27622.65	39
5335	coccidia		3	70175.17	28970.88	39
5335	line*coccidia	1	0	11886.17	39064.33	39
5335	line*coccidia	1	1	-1.5E-11	39064.33	39
5335	line*coccidia	1	2	-7.3E-12	39064.33	39
5335	line*coccidia	1	3	29864.33	39064.33	39
5335	line*coccidia	2	0	84074	39064.33	39
5335	line*coccidia	2	1	0	39064.33	39
5335	line*coccidia	2	2	18610.5	39064.33	39
5335	line*coccidia	2	3	110486	42792.83	39
5337	line	1		48690.63	49241.79	40
5337	line	2		126839.8	49241.79	40

5337	coccidia		0	70884.25	69638.41	40
5337	coccidia		1	175388.6	69638.41	40
5337	coccidia		2	36439.67	69638.41	40
5337	coccidia		3	68348.42	69638.41	40
5337	line*coccidia	1	0	62743.33	98483.59	40
5337	line*coccidia	1	1	-2.9E-11	98483.59	40
5337	line*coccidia	1	2	31852.33	98483.59	40
5337	line*coccidia	1	3	100166.8	98483.59	40
5337	line*coccidia	2	0	79025.17	98483.59	40
5337	line*coccidia	2	1	350777.2	98483.59	40
5337	line*coccidia	2	2	41027	98483.59	40
5337	line*coccidia	2	3	36530	98483.59	40
5339	line	1		159799.9	19214.17	40
5339	line	2		182357.7	19214.17	40
5339	coccidia		0	310200.5	27172.95	40
5339	coccidia		1	124242.9	27172.95	40
5339	coccidia		2	67872.42	27172.95	40
5339	coccidia		3	181999.3	27172.95	40
5339	line*coccidia	1	0	325530	38428.35	40
5339	line*coccidia	1	1	59580.33	38428.35	40
5339	line*coccidia	1	2	57780.33	38428.35	40
5339	line*coccidia	1	3	196308.8	38428.35	40
5339	line*coccidia	2	0	294871	38428.35	40
5339	line*coccidia	2	1	188905.5	38428.35	40
5339	line*coccidia	2	2	77964.5	38428.35	40
5339	line*coccidia	2	3	167689.8	38428.35	40
5344	line	1		176674.7	16709.36	40
5344	line	2		170531.5	16709.36	40
5344	coccidia		0	326643.3	23630.61	40
5344	coccidia		1	180906.8	23630.61	40
5344	coccidia		2	49285	23630.61	40
5344	coccidia		3	137577.3	23630.61	40
5344	line*coccidia	1	0	385059	33418.73	40
5344	line*coccidia	1	1	74338.67	33418.73	40
5344	line*coccidia	1	2	34472.67	33418.73	40
5344	line*coccidia	1	3	212828.5	33418.73	40
5344	line*coccidia	2	0	268227.7	33418.73	40
5344	line*coccidia	2	1	287475	33418.73	40
5344	line*coccidia	2	2	64097.33	33418.73	40
5344	line*coccidia	2	3	62326.17	33418.73	40
5346	line	1		69842.33	20761.28	40
5346	line	2		86967.63	20761.28	40
5346	coccidia		0	61576.08	29360.88	40
5346	coccidia		1	175192.3	29360.88	40
5346	coccidia		2	10846.25	29360.88	40
5346	coccidia		3	66005.33	29360.88	40
5346	line*coccidia	1	0	20621.67	41522.56	40
5346	line*coccidia	1	1	220775.7	41522.56	40
5346	line*coccidia	1	2	7.28E-12	41522.56	40

5346	line*coccidia	1	3	37972	41522.56	40
5346	line*coccidia	2	0	102530.5	41522.56	40
5346	line*coccidia	2	1	129608.8	41522.56	40
5346	line*coccidia	2	2	21692.5	41522.56	40
5346	line*coccidia	2	3	94038.67	41522.56	40
5348	line	1		6348.708	2038.826	40
5348	line	2		9990.208	2038.826	40
5348	coccidia		0	11342.42	2883.335	40
5348	coccidia		1	9335.5	2883.335	40
5348	coccidia		2	5860.25	2883.335	40
5348	coccidia		3	6139.667	2883.335	40
5348	line*coccidia	1	0	13115.5	4077.651	40
5348	line*coccidia	1	1	-4.5E-12	4077.651	40
5348	line*coccidia	1	2	-9.1E-13	4077.651	40
5348	line*coccidia	1	3	12279.33	4077.651	40
5348	line*coccidia	2	0	9569.333	4077.651	40
5348	line*coccidia	2	1	18671	4077.651	40
5348	line*coccidia	2	2	11720.5	4077.651	40
5348	line*coccidia	2	3	-9.1E-13	4077.651	40
5350	line	1		253060.9	34398.69	40
5350	line	2		69808.92	34398.69	40
5350	coccidia		0	154895.2	48647.1	40
5350	coccidia		1	101716.6	48647.1	40
5350	coccidia		2	154097	48647.1	40
5350	coccidia		3	235030.9	48647.1	40
5350	line*coccidia	1	0	258091.5	68797.39	40
5350	line*coccidia	1	1	116855	68797.39	40
5350	line*coccidia	1	2	272208.8	68797.39	40
5350	line*coccidia	1	3	365088.3	68797.39	40
5350	line*coccidia	2	0	51698.83	68797.39	40
5350	line*coccidia	2	1	86578.17	68797.39	40
5350	line*coccidia	2	2	35985.17	68797.39	40
5350	line*coccidia	2	3	104973.5	68797.39	40
5401	line	1		8872774	869303.8	40
5401	line	2		12044828	869303.8	40
5401	coccidia		0	14216803	1229381	40
5401	coccidia		1	9087145	1229381	40
5401	coccidia		2	7187980	1229381	40
5401	coccidia		3	11343276	1229381	40
5401	line*coccidia	1	0	10976661	1738608	40
5401	line*coccidia	1	1	5471576	1738608	40
5401	line*coccidia	1	2	7306455	1738608	40
5401	line*coccidia	1	3	11736403	1738608	40
5401	line*coccidia	2	0	17456945	1738608	40
5401	line*coccidia	2	1	12702714	1738608	40
5401	line*coccidia	2	2	7069504	1738608	40
5401	line*coccidia	2	3	10950148	1738608	40
5402	line	1		12230739	665710.5	40
5402	line	2		14679714	665710.5	40

5402	coccidia		0	17511786	941456.8	40
5402	coccidia		1	11311600	941456.8	40
5402	coccidia		2	9523165	941456.8	40
5402	coccidia		3	15474356	941456.8	40
5402	line*coccidia	1	0	14068527	1331421	40
5402	line*coccidia	1	1	9032791	1331421	40
5402	line*coccidia	1	2	10101256	1331421	40
5402	line*coccidia	1	3	15720384	1331421	40
5402	line*coccidia	2	0	20955046	1331421	40
5402	line*coccidia	2	1	13590409	1331421	40
5402	line*coccidia	2	2	8945074	1331421	40
5402	line*coccidia	2	3	15228329	1331421	40
5403	line	1		591731.5	124245.4	40
5403	line	2		670697.8	124245.4	40
5403	coccidia		0	229267.5	175709.5	40
5403	coccidia		1	1280519	175709.5	40
5403	coccidia		2	238460	175709.5	40
5403	coccidia		3	776612.7	175709.5	40
5403	line*coccidia	1	0	284164.7	248490.8	40
5403	line*coccidia	1	1	468171.8	248490.8	40
5403	line*coccidia	1	2	266922.2	248490.8	40
5403	line*coccidia	1	3	1347667	248490.8	40
5403	line*coccidia	2	0	174370.3	248490.8	40
5403	line*coccidia	2	1	2092865	248490.8	40
5403	line*coccidia	2	2	209997.8	248490.8	40
5403	line*coccidia	2	3	205558	248490.8	40
5404	line	1		2351060	184359	40
5404	line	2		2185295	184359	40
5404	coccidia		0	4194250	260723	40
5404	coccidia		1	1703542	260723	40
5404	coccidia		2	1040027	260723	40
5404	coccidia		3	2134891	260723	40
5404	line*coccidia	1	0	4685798	368718.1	40
5404	line*coccidia	1	1	826001.3	368718.1	40
5404	line*coccidia	1	2	789777	368718.1	40
5404	line*coccidia	1	3	3102666	368718.1	40
5404	line*coccidia	2	0	3702702	368718.1	40
5404	line*coccidia	2	1	2581082	368718.1	40
5404	line*coccidia	2	2	1290278	368718.1	40
5404	line*coccidia	2	3	1167116	368718.1	40
5405	line	1		716473.4	105249.4	40
5405	line	2		914752.1	105249.4	40
5405	coccidia		0	981117.6	148845.2	40
5405	coccidia		1	1000069	148845.2	40
5405	coccidia		2	553173.7	148845.2	40
5405	coccidia		3	728091.1	148845.2	40
5405	line*coccidia	1	0	990215.5	210498.9	40
5405	line*coccidia	1	1	382224.3	210498.9	40
5405	line*coccidia	1	2	439061.3	210498.9	40

5405	line*coccidia	1	3	1054392	210498.9	40
5405	line*coccidia	2	0	972019.7	210498.9	40
5405	line*coccidia	2	1	1617913	210498.9	40
5405	line*coccidia	2	2	667286	210498.9	40
5405	line*coccidia	2	3	401789.8	210498.9	40
5406	line	1		6281718	531171.6	40
5406	line	2		8193038	531171.6	40
5406	coccidia		0	8810696	751190	40
5406	coccidia		1	7058396	751190	40
5406	coccidia		2	4950679	751190	40
5406	coccidia		3	8129741	751190	40
5406	line*coccidia	1	0	8028258	1062343	40
5406	line*coccidia	1	1	3042703	1062343	40
5406	line*coccidia	1	2	4094218	1062343	40
5406	line*coccidia	1	3	9961691	1062343	40
5406	line*coccidia	2	0	9593133	1062343	40
5406	line*coccidia	2	1	11074089	1062343	40
5406	line*coccidia	2	2	5807140	1062343	40
5406	line*coccidia	2	3	6297791	1062343	40
5407	line	1		21152072	1079208	40
5407	line	2		25429461	1079208	40
5407	coccidia		0	29587692	1526231	40
5407	coccidia		1	18716977	1526231	40
5407	coccidia		2	18321796	1526231	40
5407	coccidia		3	26536601	1526231	40
5407	line*coccidia	1	0	26552026	2158417	40
5407	line*coccidia	1	1	13429139	2158417	40
5407	line*coccidia	1	2	16934227	2158417	40
5407	line*coccidia	1	3	27692896	2158417	40
5407	line*coccidia	2	0	32623359	2158417	40
5407	line*coccidia	2	1	24004816	2158417	40
5407	line*coccidia	2	2	19709365	2158417	40
5407	line*coccidia	2	3	25380305	2158417	40
5409	line	1		1723877	244654.4	40
5409	line	2		2708978	244654.4	40
5409	coccidia		0	2302999	345993.6	40
5409	coccidia		1	2203807	345993.6	40
5409	coccidia		2	1627338	345993.6	40
5409	coccidia		3	2731565	345993.6	40
5409	line*coccidia	1	0	2344312	489308.8	40
5409	line*coccidia	1	1	567809.3	489308.8	40
5409	line*coccidia	1	2	795579.7	489308.8	40
5409	line*coccidia	1	3	3187808	489308.8	40
5409	line*coccidia	2	0	2261686	489308.8	40
5409	line*coccidia	2	1	3839805	489308.8	40
5409	line*coccidia	2	2	2459096	489308.8	40
5409	line*coccidia	2	3	2275323	489308.8	40
5412	line	1		1266324	171667.4	40
5412	line	2		1808350	171667.4	40

5412	coccidia		0	1734632	242774.4	40
5412	coccidia		1	1881604	242774.4	40
5412	coccidia		2	1115787	242774.4	40
5412	coccidia		3	1417325	242774.4	40
5412	line*coccidia	1	0	1641582	343334.8	40
5412	line*coccidia	1	1	740840.8	343334.8	40
5412	line*coccidia	1	2	929303	343334.8	40
5412	line*coccidia	1	3	1753571	343334.8	40
5412	line*coccidia	2	0	1827682	343334.8	40
5412	line*coccidia	2	1	3022367	343334.8	40
5412	line*coccidia	2	2	1302272	343334.8	40
5412	line*coccidia	2	3	1081079	343334.8	40
5413	line	1		12930603	633470.4	40
5413	line	2		15186931	633470.4	40
5413	coccidia		0	17877174	895862.5	40
5413	coccidia		1	11701453	895862.5	40
5413	coccidia		2	10246158	895862.5	40
5413	coccidia		3	16410283	895862.5	40
5413	line*coccidia	1	0	17591221	1266941	40
5413	line*coccidia	1	1	6366971	1266941	40
5413	line*coccidia	1	2	8342020	1266941	40
5413	line*coccidia	1	3	19422200	1266941	40
5413	line*coccidia	2	0	18163128	1266941	40
5413	line*coccidia	2	1	17035935	1266941	40
5413	line*coccidia	2	2	12150296	1266941	40
5413	line*coccidia	2	3	13398366	1266941	40
5414	line	1		275004.9	62188.28	40
5414	line	2		294570.1	62188.28	40
5414	coccidia		0	161993.9	87947.51	40
5414	coccidia		1	496138.5	87947.51	40
5414	coccidia		2	156780.2	87947.51	40
5414	coccidia		3	324237.3	87947.51	40
5414	line*coccidia	1	0	164736.3	124376.6	40
5414	line*coccidia	1	1	339755.7	124376.6	40
5414	line*coccidia	1	2	169142.8	124376.6	40
5414	line*coccidia	1	3	426384.7	124376.6	40
5414	line*coccidia	2	0	159251.5	124376.6	40
5414	line*coccidia	2	1	652521.3	124376.6	40
5414	line*coccidia	2	2	144417.5	124376.6	40
5414	line*coccidia	2	3	222090	124376.6	40
5415	line	1		2227541	532238.9	40
5415	line	2		8052389	532238.9	40
5415	coccidia		0	7372249	752699.4	40
5415	coccidia		1	4358038	752699.4	40
5415	coccidia		2	5022283	752699.4	40
5415	coccidia		3	3807291	752699.4	40
5415	line*coccidia	1	0	5087454	1064478	40
5415	line*coccidia	1	1	1527947	1064478	40
5415	line*coccidia	1	2	594599.7	1064478	40

5415	line*coccidia	1	3	1700165	1064478	40
5415	line*coccidia	2	0	9657044	1064478	40
5415	line*coccidia	2	1	7188129	1064478	40
5415	line*coccidia	2	2	9449966	1064478	40
5415	line*coccidia	2	3	5914417	1064478	40
5417	line	1		829936.3	141020.9	40
5417	line	2		436300	141020.9	40
5417	coccidia		0	919067.9	199433.7	40
5417	coccidia		1	472813.7	199433.7	40
5417	coccidia		2	335247.6	199433.7	40
5417	coccidia		3	805343.4	199433.7	40
5417	line*coccidia	1	0	1352779	282041.8	40
5417	line*coccidia	1	1	420996.2	282041.8	40
5417	line*coccidia	1	2	232457.2	282041.8	40
5417	line*coccidia	1	3	1313513	282041.8	40
5417	line*coccidia	2	0	485356.7	282041.8	40
5417	line*coccidia	2	1	524631.2	282041.8	40
5417	line*coccidia	2	2	438038	282041.8	40
5417	line*coccidia	2	3	297174.2	282041.8	40
5418	line	1		3689575	252819.5	40
5418	line	2		4580895	252819.5	40
5418	coccidia		0	5348187	357540.8	40
5418	coccidia		1	3890585	357540.8	40
5418	coccidia		2	2660976	357540.8	40
5418	coccidia		3	4641192	357540.8	40
5418	line*coccidia	1	0	5439559	505639	40
5418	line*coccidia	1	1	1533818	505639	40
5418	line*coccidia	1	2	1821923	505639	40
5418	line*coccidia	1	3	5962999	505639	40
5418	line*coccidia	2	0	5256814	505639	40
5418	line*coccidia	2	1	6247351	505639	40
5418	line*coccidia	2	2	3500029	505639	40
5418	line*coccidia	2	3	3319385	505639	40
5419	line	1		282317.4	53881.14	40
5419	line	2		234505.4	53881.14	40
5419	coccidia		0	195312	76199.44	40
5419	coccidia		1	298229.1	76199.44	40
5419	coccidia		2	123080.6	76199.44	40
5419	coccidia		3	417023.8	76199.44	40
5419	line*coccidia	1	0	258549.3	107762.3	40
5419	line*coccidia	1	1	317757.7	107762.3	40
5419	line*coccidia	1	2	119973	107762.3	40
5419	line*coccidia	1	3	432989.5	107762.3	40
5419	line*coccidia	2	0	132074.7	107762.3	40
5419	line*coccidia	2	1	278700.5	107762.3	40
5419	line*coccidia	2	2	126188.2	107762.3	40
5419	line*coccidia	2	3	401058.2	107762.3	40
5420	line	1		6314172	372034.3	40
5420	line	2		7155595	372034.3	40

5420	coccidia		0	9416544	526136	40
5420	coccidia		1	4860206	526136	40
5420	coccidia		2	4528360	526136	40
5420	coccidia		3	8134424	526136	40
5420	line*coccidia	1	0	9219898	744068.6	40
5420	line*coccidia	1	1	2328119	744068.6	40
5420	line*coccidia	1	2	3084601	744068.6	40
5420	line*coccidia	1	3	10624069	744068.6	40
5420	line*coccidia	2	0	9613190	744068.6	40
5420	line*coccidia	2	1	7392294	744068.6	40
5420	line*coccidia	2	2	5972118	744068.6	40
5420	line*coccidia	2	3	5644779	744068.6	40
5422	line	1		150399.8	24259.31	40
5422	line	2		138227.9	24259.31	40
5422	coccidia		0	213950.9	34307.84	40
5422	coccidia		1	118879.4	34307.84	40
5422	coccidia		2	48707.25	34307.84	40
5422	coccidia		3	195717.8	34307.84	40
5422	line*coccidia	1	0	242951.2	48518.61	40
5422	line*coccidia	1	1	167130	48518.61	40
5422	line*coccidia	1	2	38290.5	48518.61	40
5422	line*coccidia	1	3	153227.3	48518.61	40
5422	line*coccidia	2	0	184950.7	48518.61	40
5422	line*coccidia	2	1	70628.83	48518.61	40
5422	line*coccidia	2	2	59124	48518.61	40
5422	line*coccidia	2	3	238208.2	48518.61	40
5425	line	1		6915737	1434859	40
5425	line	2		7440187	1434859	40
5425	coccidia		0	11000265	2029197	40
5425	coccidia		1	7125443	2029197	40
5425	coccidia		2	5163005	2029197	40
5425	coccidia		3	5423134	2029197	40
5425	line*coccidia	1	0	10473384	2869717	40
5425	line*coccidia	1	1	7344610	2869717	40
5425	line*coccidia	1	2	4231868	2869717	40
5425	line*coccidia	1	3	5613085	2869717	40
5425	line*coccidia	2	0	11527147	2869717	40
5425	line*coccidia	2	1	6906276	2869717	40
5425	line*coccidia	2	2	6094142	2869717	40
5425	line*coccidia	2	3	5233183	2869717	40
5426	line	1		2733022	361948.7	40
5426	line	2		4278529	361948.7	40
5426	coccidia		0	3567804	511872.7	40
5426	coccidia		1	2827590	511872.7	40
5426	coccidia		2	2692852	511872.7	40
5426	coccidia		3	4934858	511872.7	40
5426	line*coccidia	1	0	1823563	723897.4	40
5426	line*coccidia	1	1	1275502	723897.4	40
5426	line*coccidia	1	2	2351698	723897.4	40

5426	line*coccidia	1	3	5481325	723897.4	40
5426	line*coccidia	2	0	5312044	723897.4	40
5426	line*coccidia	2	1	4379677	723897.4	40
5426	line*coccidia	2	2	3034005	723897.4	40
5426	line*coccidia	2	3	4388390	723897.4	40
5427	line	1		204327.9	40380.67	40
5427	line	2		234706.2	40380.67	40
5427	coccidia		0	137932.8	57106.9	40
5427	coccidia		1	266060.1	57106.9	40
5427	coccidia		2	123348.6	57106.9	40
5427	coccidia		3	350726.7	57106.9	40
5427	line*coccidia	1	0	179137.5	80761.35	40
5427	line*coccidia	1	1	181034.8	80761.35	40
5427	line*coccidia	1	2	121692.5	80761.35	40
5427	line*coccidia	1	3	335446.7	80761.35	40
5427	line*coccidia	2	0	96728	80761.35	40
5427	line*coccidia	2	1	351085.3	80761.35	40
5427	line*coccidia	2	2	125004.7	80761.35	40
5427	line*coccidia	2	3	366006.7	80761.35	40
5429	line	1		1181065	164152.4	40
5429	line	2		1843004	164152.4	40
5429	coccidia		0	2254704	232146.6	40
5429	coccidia		1	1615830	232146.6	40
5429	coccidia		2	1067694	232146.6	40
5429	coccidia		3	1109910	232146.6	40
5429	line*coccidia	1	0	2338435	328304.8	40
5429	line*coccidia	1	1	179832	328304.8	40
5429	line*coccidia	1	2	171275.8	328304.8	40
5429	line*coccidia	1	3	2034717	328304.8	40
5429	line*coccidia	2	0	2170972	328304.8	40
5429	line*coccidia	2	1	3051828	328304.8	40
5429	line*coccidia	2	2	1964113	328304.8	40
5429	line*coccidia	2	3	185102.5	328304.8	40
5501	line	1		288640.1	70247.9	40
5501	line	2		191073.9	70247.9	40
5501	coccidia		0	180549.4	99345.53	40
5501	coccidia		1	207067.7	99345.53	40
5501	coccidia		2	244742.8	99345.53	40
5501	coccidia		3	327068.2	99345.53	40
5501	line*coccidia	1	0	174477.5	140495.8	40
5501	line*coccidia	1	1	179640.5	140495.8	40
5501	line*coccidia	1	2	323029	140495.8	40
5501	line*coccidia	1	3	477413.5	140495.8	40
5501	line*coccidia	2	0	186621.3	140495.8	40
5501	line*coccidia	2	1	234494.8	140495.8	40
5501	line*coccidia	2	2	166456.5	140495.8	40
5501	line*coccidia	2	3	176722.8	140495.8	40
5504	line	1		522863	65431.67	40
5504	line	2		481018.2	65431.67	40

5504	coccidia		0	743639.7	92534.35	40
5504	coccidia		1	528814.2	92534.35	40
5504	coccidia		2	270499.7	92534.35	40
5504	coccidia		3	464808.8	92534.35	40
5504	line*coccidia	1	0	549777.3	130863.3	40
5504	line*coccidia	1	1	542398.2	130863.3	40
5504	line*coccidia	1	2	179693.3	130863.3	40
5504	line*coccidia	1	3	819583	130863.3	40
5504	line*coccidia	2	0	937502	130863.3	40
5504	line*coccidia	2	1	515230.2	130863.3	40
5504	line*coccidia	2	2	361306	130863.3	40
5504	line*coccidia	2	3	110034.5	130863.3	40
5506	line	1		356568.3	82514.01	40
5506	line	2		155603.6	82514.01	40
5506	coccidia		0	222477.3	116692.4	40
5506	coccidia		1	153812.3	116692.4	40
5506	coccidia		2	198156.8	116692.4	40
5506	coccidia		3	449897.3	116692.4	40
5506	line*coccidia	1	0	215848.7	165028	40
5506	line*coccidia	1	1	170685.3	165028	40
5506	line*coccidia	1	2	227542.7	165028	40
5506	line*coccidia	1	3	812196.3	165028	40
5506	line*coccidia	2	0	229106	165028	40
5506	line*coccidia	2	1	136939.3	165028	40
5506	line*coccidia	2	2	168770.8	165028	40
5506	line*coccidia	2	3	87598.33	165028	40
5509	line	1		1118122	132432.6	40
5509	line	2		780724.3	132432.6	40
5509	coccidia		0	1004067	187288	40
5509	coccidia		1	699904.9	187288	40
5509	coccidia		2	384747.3	187288	40
5509	coccidia		3	1708974	187288	40
5509	line*coccidia	1	0	1376145	264865.3	40
5509	line*coccidia	1	1	58016	264865.3	40
5509	line*coccidia	1	2	449576.7	264865.3	40
5509	line*coccidia	1	3	2588751	264865.3	40
5509	line*coccidia	2	0	631988	264865.3	40
5509	line*coccidia	2	1	1341794	264865.3	40
5509	line*coccidia	2	2	319917.8	264865.3	40
5509	line*coccidia	2	3	829197.7	264865.3	40
5510	line	1		1250726	204212.4	40
5510	line	2		1944662	204212.4	40
5510	coccidia		0	2719292	288799.9	40
5510	coccidia		1	1424424	288799.9	40
5510	coccidia		2	900797.7	288799.9	40
5510	coccidia		3	1346261	288799.9	40
5510	line*coccidia	1	0	2378520	408424.7	40
5510	line*coccidia	1	1	129093.8	408424.7	40
5510	line*coccidia	1	2	355991.7	408424.7	40

5510	line*coccidia	1	3	2139297	408424.7	40
5510	line*coccidia	2	0	3060064	408424.7	40
5510	line*coccidia	2	1	2719753	408424.7	40
5510	line*coccidia	2	2	1445604	408424.7	40
5510	line*coccidia	2	3	553226.2	408424.7	40
5511	line	1		230904.4	27117.34	40
5511	line	2		269131.3	27117.34	40
5511	coccidia		0	327586.5	38349.71	40
5511	coccidia		1	189536.3	38349.71	40
5511	coccidia		2	214047.5	38349.71	40
5511	coccidia		3	268901.1	38349.71	40
5511	line*coccidia	1	0	318999	54234.69	40
5511	line*coccidia	1	1	44903.67	54234.69	40
5511	line*coccidia	1	2	206503.2	54234.69	40
5511	line*coccidia	1	3	353211.8	54234.69	40
5511	line*coccidia	2	0	336174	54234.69	40
5511	line*coccidia	2	1	334169	54234.69	40
5511	line*coccidia	2	2	221591.8	54234.69	40
5511	line*coccidia	2	3	184590.3	54234.69	40
5512	line	1		688400.2	71541.38	40
5512	line	2		729512.1	71541.38	40
5512	coccidia		0	1046761	101174.8	40
5512	coccidia		1	527150.7	101174.8	40
5512	coccidia		2	301911.3	101174.8	40
5512	coccidia		3	960001.8	101174.8	40
5512	line*coccidia	1	0	971605	143082.8	40
5512	line*coccidia	1	1	192459.7	143082.8	40
5512	line*coccidia	1	2	148364.2	143082.8	40
5512	line*coccidia	1	3	1441172	143082.8	40
5512	line*coccidia	2	0	1121917	143082.8	40
5512	line*coccidia	2	1	861841.7	143082.8	40
5512	line*coccidia	2	2	455458.3	143082.8	40
5512	line*coccidia	2	3	478831.5	143082.8	40
5513	line	1		597758.2	57175.95	40
5513	line	2		498061.7	57175.95	40
5513	coccidia		0	521817	80859	40
5513	coccidia		1	788234.8	80859	40
5513	coccidia		2	266942.3	80859	40
5513	coccidia		3	614645.7	80859	40
5513	line*coccidia	1	0	685314.5	114351.9	40
5513	line*coccidia	1	1	577045.3	114351.9	40
5513	line*coccidia	1	2	185225.8	114351.9	40
5513	line*coccidia	1	3	943447	114351.9	40
5513	line*coccidia	2	0	358319.5	114351.9	40
5513	line*coccidia	2	1	999424.2	114351.9	40
5513	line*coccidia	2	2	348658.7	114351.9	40
5513	line*coccidia	2	3	285844.3	114351.9	40
5514	line	1		396185.9	39022.21	40
5514	line	2		363944.6	39022.21	40

5514	coccidia		0	445903.4	55185.74	40
5514	coccidia		1	363067.5	55185.74	40
5514	coccidia		2	284689.7	55185.74	40
5514	coccidia		3	426600.3	55185.74	40
5514	line*coccidia	1	0	453206	78044.42	40
5514	line*coccidia	1	1	267336.5	78044.42	40
5514	line*coccidia	1	2	216311.2	78044.42	40
5514	line*coccidia	1	3	647889.8	78044.42	40
5514	line*coccidia	2	0	438600.8	78044.42	40
5514	line*coccidia	2	1	458798.5	78044.42	40
5514	line*coccidia	2	2	353068.2	78044.42	40
5514	line*coccidia	2	3	205310.8	78044.42	40
5516	line	1		1220310	159430.2	40
5516	line	2		1155996	159430.2	40
5516	coccidia		0	1853235	225468.3	40
5516	coccidia		1	780079.3	225468.3	40
5516	coccidia		2	771176.3	225468.3	40
5516	coccidia		3	1348121	225468.3	40
5516	line*coccidia	1	0	2218670	318860.4	40
5516	line*coccidia	1	1	254343.2	318860.4	40
5516	line*coccidia	1	2	495901.2	318860.4	40
5516	line*coccidia	1	3	1912325	318860.4	40
5516	line*coccidia	2	0	1487800	318860.4	40
5516	line*coccidia	2	1	1305815	318860.4	40
5516	line*coccidia	2	2	1046452	318860.4	40
5516	line*coccidia	2	3	783916.2	318860.4	40
5517	line	1		86249.83	29885.97	40
5517	line	2		180630.7	29885.97	40
5517	coccidia		0	162655.2	42265.14	40
5517	coccidia		1	298088.5	42265.14	40
5517	coccidia		2	73017.33	42265.14	40
5517	coccidia		3	-1.5E-11	42265.14	40
5517	line*coccidia	1	0	146964.2	59771.94	40
5517	line*coccidia	1	1	198035.2	59771.94	40
5517	line*coccidia	1	2	-1.5E-11	59771.94	40
5517	line*coccidia	1	3	-1.5E-11	59771.94	40
5517	line*coccidia	2	0	178346.2	59771.94	40
5517	line*coccidia	2	1	398141.8	59771.94	40
5517	line*coccidia	2	2	146034.7	59771.94	40
5517	line*coccidia	2	3	-1.5E-11	59771.94	40
5518	line	1		1221865	202870.8	40
5518	line	2		1675497	202870.8	40
5518	coccidia		0	2684193	286902.6	40
5518	coccidia		1	1292669	286902.6	40
5518	coccidia		2	882221.3	286902.6	40
5518	coccidia		3	935642.3	286902.6	40
5518	line*coccidia	1	0	2441160	405741.5	40
5518	line*coccidia	1	1	287634.2	405741.5	40
5518	line*coccidia	1	2	532890.2	405741.5	40

5518	line*coccidia	1	3	1625776	405741.5	40
5518	line*coccidia	2	0	2927225	405741.5	40
5518	line*coccidia	2	1	2297704	405741.5	40
5518	line*coccidia	2	2	1231552	405741.5	40
5518	line*coccidia	2	3	245508.8	405741.5	40
5519	line	1		910293.3	118952.8	40
5519	line	2		696828.3	118952.8	40
5519	coccidia		0	456666.2	168224.6	40
5519	coccidia		1	741635.3	168224.6	40
5519	coccidia		2	236947.3	168224.6	40
5519	coccidia		3	1778994	168224.6	40
5519	line*coccidia	1	0	537512.2	237905.6	40
5519	line*coccidia	1	1	45122.83	237905.6	40
5519	line*coccidia	1	2	210739.5	237905.6	40
5519	line*coccidia	1	3	2847799	237905.6	40
5519	line*coccidia	2	0	375820.2	237905.6	40
5519	line*coccidia	2	1	1438148	237905.6	40
5519	line*coccidia	2	2	263155.2	237905.6	40
5519	line*coccidia	2	3	710190.2	237905.6	40
5520	line	1		13501670	1586039	40
5520	line	2		9830062	1586039	40
5520	coccidia		0	22716627	2242998	40
5520	coccidia		1	6678753	2242998	40
5520	coccidia		2	7304111	2242998	40
5520	coccidia		3	9963973	2242998	40
5520	line*coccidia	1	0	30874797	3172078	40
5520	line*coccidia	1	1	5937406	3172078	40
5520	line*coccidia	1	2	5093142	3172078	40
5520	line*coccidia	1	3	12101334	3172078	40
5520	line*coccidia	2	0	14558458	3172078	40
5520	line*coccidia	2	1	7420100	3172078	40
5520	line*coccidia	2	2	9515080	3172078	40
5520	line*coccidia	2	3	7826611	3172078	40
5526	line	1		9520010	2477916	40
5526	line	2		13906298	2477916	40
5526	coccidia		0	13323571	3504302	40
5526	coccidia		1	6148445	3504302	40
5526	coccidia		2	14446602	3504302	40
5526	coccidia		3	12934001	3504302	40
5526	line*coccidia	1	0	13750936	4955832	40
5526	line*coccidia	1	1	3224510	4955832	40
5526	line*coccidia	1	2	6163256	4955832	40
5526	line*coccidia	1	3	14941340	4955832	40
5526	line*coccidia	2	0	12896205	4955832	40
5526	line*coccidia	2	1	9072379	4955832	40
5526	line*coccidia	2	2	22729948	4955832	40
5526	line*coccidia	2	3	10926662	4955832	40
5527	line	1		6102274	1442674	40
5527	line	2		8000785	1442674	40

5527	coccidia		0	11637320	2040249	40
5527	coccidia		1	4439364	2040249	40
5527	coccidia		2	6410318	2040249	40
5527	coccidia		3	5719115	2040249	40
5527	line*coccidia	1	0	11875646	2885348	40
5527	line*coccidia	1	1	3333226	2885348	40
5527	line*coccidia	1	2	2230781	2885348	40
5527	line*coccidia	1	3	6969443	2885348	40
5527	line*coccidia	2	0	11398994	2885348	40
5527	line*coccidia	2	1	5545502	2885348	40
5527	line*coccidia	2	2	10589856	2885348	40
5527	line*coccidia	2	3	4468788	2885348	40
5601	line	1		176016.9	39735.27	40
5601	line	2		199857.1	39735.27	40
5601	coccidia		0	258411.7	56194.16	40
5601	coccidia		1	131075.8	56194.16	40
5601	coccidia		2	208753.7	56194.16	40
5601	coccidia		3	153506.8	56194.16	40
5601	line*coccidia	1	0	253738.7	79470.54	40
5601	line*coccidia	1	1	98774.67	79470.54	40
5601	line*coccidia	1	2	124358.8	79470.54	40
5601	line*coccidia	1	3	227195.3	79470.54	40
5601	line*coccidia	2	0	263084.7	79470.54	40
5601	line*coccidia	2	1	163376.8	79470.54	40
5601	line*coccidia	2	2	293148.5	79470.54	40
5601	line*coccidia	2	3	79818.33	79470.54	40
5605	line	1		73201.29	21212.2	40
5605	line	2		71576.71	21212.2	40
5605	coccidia		0	62855.75	29998.58	40
5605	coccidia		1	118963.5	29998.58	40
5605	coccidia		2	43127.33	29998.58	40
5605	coccidia		3	64609.42	29998.58	40
5605	line*coccidia	1	0	70137.17	42424.41	40
5605	line*coccidia	1	1	55462.67	42424.41	40
5605	line*coccidia	1	2	61560.83	42424.41	40
5605	line*coccidia	1	3	105644.5	42424.41	40
5605	line*coccidia	2	0	55574.33	42424.41	40
5605	line*coccidia	2	1	182464.3	42424.41	40
5605	line*coccidia	2	2	24693.83	42424.41	40
5605	line*coccidia	2	3	23574.33	42424.41	40
5609	line	1		429058.5	62435.47	40
5609	line	2		620939.2	62435.47	40
5609	coccidia		0	693939.1	88297.09	40
5609	coccidia		1	373666	88297.09	40
5609	coccidia		2	495250.9	88297.09	40
5609	coccidia		3	537139.4	88297.09	40
5609	line*coccidia	1	0	652623.2	124870.9	40
5609	line*coccidia	1	1	156628.8	124870.9	40
5609	line*coccidia	1	2	226847.7	124870.9	40

5609	line*coccidia	1	3	680134.3	124870.9	40
5609	line*coccidia	2	0	735255	124870.9	40
5609	line*coccidia	2	1	590703.2	124870.9	40
5609	line*coccidia	2	2	763654.2	124870.9	40
5609	line*coccidia	2	3	394144.5	124870.9	40
5611	line	1		189167.3	36583.38	40
5611	line	2		236966.4	36583.38	40
5611	coccidia		0	197314.4	51736.71	40
5611	coccidia		1	249369.7	51736.71	40
5611	coccidia		2	96954.92	51736.71	40
5611	coccidia		3	308628.3	51736.71	40
5611	line*coccidia	1	0	210925.5	73166.76	40
5611	line*coccidia	1	1	100284.3	73166.76	40
5611	line*coccidia	1	2	100618.7	73166.76	40
5611	line*coccidia	1	3	344840.5	73166.76	40
5611	line*coccidia	2	0	183703.3	73166.76	40
5611	line*coccidia	2	1	398455	73166.76	40
5611	line*coccidia	2	2	93291.17	73166.76	40
5611	line*coccidia	2	3	272416	73166.76	40
5612	line	1		879954.6	104980.8	40
5612	line	2		889081.8	104980.8	40
5612	coccidia		0	894094.5	148465.3	40
5612	coccidia		1	701375.9	148465.3	40
5612	coccidia		2	632670.4	148465.3	40
5612	coccidia		3	1309932	148465.3	40
5612	line*coccidia	1	0	1002162	209961.7	40
5612	line*coccidia	1	1	281222.3	209961.7	40
5612	line*coccidia	1	2	394245.2	209961.7	40
5612	line*coccidia	1	3	1842190	209961.7	40
5612	line*coccidia	2	0	786027.5	209961.7	40
5612	line*coccidia	2	1	1121530	209961.7	40
5612	line*coccidia	2	2	871095.7	209961.7	40
5612	line*coccidia	2	3	777674.5	209961.7	40
5613	line	1		100981.8	19169.9	40
5613	line	2		83845.42	19169.9	40
5613	coccidia		0	219195.3	27110.33	40
5613	coccidia		1	1.09E-11	27110.33	40
5613	coccidia		2	119394	27110.33	40
5613	coccidia		3	31065.08	27110.33	40
5613	line*coccidia	1	0	267799.5	38339.8	40
5613	line*coccidia	1	1	7.28E-12	38339.8	40
5613	line*coccidia	1	2	136127.7	38339.8	40
5613	line*coccidia	1	3	-7.3E-12	38339.8	40
5613	line*coccidia	2	0	170591.2	38339.8	40
5613	line*coccidia	2	1	1.46E-11	38339.8	40
5613	line*coccidia	2	2	102660.3	38339.8	40
5613	line*coccidia	2	3	62130.17	38339.8	40
5617	line	1		328899.8	34937.8	40
5617	line	2		413795.9	34937.8	40

5617	coccidia		0	463532.3	49409.51	40
5617	coccidia		1	463583.8	49409.51	40
5617	coccidia		2	216635.6	49409.51	40
5617	coccidia		3	341639.8	49409.51	40
5617	line*coccidia	1	0	462940	69875.6	40
5617	line*coccidia	1	1	189488.2	69875.6	40
5617	line*coccidia	1	2	179187.7	69875.6	40
5617	line*coccidia	1	3	483983.5	69875.6	40
5617	line*coccidia	2	0	464124.7	69875.6	40
5617	line*coccidia	2	1	737679.3	69875.6	40
5617	line*coccidia	2	2	254083.5	69875.6	40
5617	line*coccidia	2	3	199296	69875.6	40
5618	line	1		104139	19667.44	40
5618	line	2		74413.96	19667.44	40
5618	coccidia		0	293886.7	27813.95	40
5618	coccidia		1	1.09E-11	27813.95	40
5618	coccidia		2	38545.08	27813.95	40
5618	coccidia		3	24674.17	27813.95	40
5618	line*coccidia	1	0	416556	39334.87	40
5618	line*coccidia	1	1	7.28E-12	39334.87	40
5618	line*coccidia	1	2	0	39334.87	40
5618	line*coccidia	1	3	0	39334.87	40
5618	line*coccidia	2	0	171217.3	39334.87	40
5618	line*coccidia	2	1	1.46E-11	39334.87	40
5618	line*coccidia	2	2	77090.17	39334.87	40
5618	line*coccidia	2	3	49348.33	39334.87	40
5620	line	1		1251674	137044.5	40
5620	line	2		1251934	137044.5	40
5620	coccidia		0	2047781	193810.2	40
5620	coccidia		1	891623	193810.2	40
5620	coccidia		2	755738.6	193810.2	40
5620	coccidia		3	1312074	193810.2	40
5620	line*coccidia	1	0	2144083	274089.1	40
5620	line*coccidia	1	1	166306.2	274089.1	40
5620	line*coccidia	1	2	671139	274089.1	40
5620	line*coccidia	1	3	2025170	274089.1	40
5620	line*coccidia	2	0	1951479	274089.1	40
5620	line*coccidia	2	1	1616940	274089.1	40
5620	line*coccidia	2	2	840338.2	274089.1	40
5620	line*coccidia	2	3	598978.5	274089.1	40
5621	line	1		2232473	286112.9	40
5621	line	2		2727532	286112.9	40
5621	coccidia		0	3292088	404624.7	40
5621	coccidia		1	2025676	404624.7	40
5621	coccidia		2	1899622	404624.7	40
5621	coccidia		3	2702622	404624.7	40
5621	line*coccidia	1	0	3246941	572225.7	40
5621	line*coccidia	1	1	1433852	572225.7	40
5621	line*coccidia	1	2	943510.3	572225.7	40

5621	line*coccidia	1	3	3305587	572225.7	40
5621	line*coccidia	2	0	3337235	572225.7	40
5621	line*coccidia	2	1	2617501	572225.7	40
5621	line*coccidia	2	2	2855734	572225.7	40
5621	line*coccidia	2	3	2099658	572225.7	40
5624	line	1		414680	50394.98	40
5624	line	2		334269	50394.98	40
5624	coccidia		0	464666.2	71269.27	40
5624	coccidia		1	318166.5	71269.27	40
5624	coccidia		2	293281.1	71269.27	40
5624	coccidia		3	421784.3	71269.27	40
5624	line*coccidia	1	0	611053.3	100790	40
5624	line*coccidia	1	1	187398.5	100790	40
5624	line*coccidia	1	2	297227.8	100790	40
5624	line*coccidia	1	3	563040.3	100790	40
5624	line*coccidia	2	0	318279	100790	40
5624	line*coccidia	2	1	448934.5	100790	40
5624	line*coccidia	2	2	289334.3	100790	40
5624	line*coccidia	2	3	280528.3	100790	40
5626	line	1		245610.2	132793.2	40
5626	line	2		264812.5	132793.2	40
5626	coccidia		0	273694.7	187798	40
5626	coccidia		1	84958.75	187798	40
5626	coccidia		2	629145.1	187798	40
5626	coccidia		3	33046.83	187798	40
5626	line*coccidia	1	0	91444.5	265586.5	40
5626	line*coccidia	1	1	85331.83	265586.5	40
5626	line*coccidia	1	2	805664.5	265586.5	40
5626	line*coccidia	1	3	-2.9E-11	265586.5	40
5626	line*coccidia	2	0	455944.8	265586.5	40
5626	line*coccidia	2	1	84585.67	265586.5	40
5626	line*coccidia	2	2	452625.7	265586.5	40
5626	line*coccidia	2	3	66093.67	265586.5	40
5630	line	1		85851.29	15859.37	40
5630	line	2		83165.92	15859.37	40
5630	coccidia		0	93983.5	22428.54	40
5630	coccidia		1	73227.08	22428.54	40
5630	coccidia		2	63484.92	22428.54	40
5630	coccidia		3	107338.9	22428.54	40
5630	line*coccidia	1	0	72972.17	31718.74	40
5630	line*coccidia	1	1	46616.17	31718.74	40
5630	line*coccidia	1	2	77719.5	31718.74	40
5630	line*coccidia	1	3	146097.3	31718.74	40
5630	line*coccidia	2	0	114994.8	31718.74	40
5630	line*coccidia	2	1	99838	31718.74	40
5630	line*coccidia	2	2	49250.33	31718.74	40
5630	line*coccidia	2	3	68580.5	31718.74	40
5635	line	1		271218.3	26312.24	40
5635	line	2		248715.6	26312.24	40

5635	coccidia		0	296908.2	37211.13	40
5635	coccidia		1	178728.6	37211.13	40
5635	coccidia		2	238130.8	37211.13	40
5635	coccidia		3	326100.4	37211.13	40
5635	line*coccidia	1	0	385373.5	52624.48	40
5635	line*coccidia	1	1	60040.5	52624.48	40
5635	line*coccidia	1	2	142216	52624.48	40
5635	line*coccidia	1	3	497243.3	52624.48	40
5635	line*coccidia	2	0	208442.8	52624.48	40
5635	line*coccidia	2	1	297416.7	52624.48	40
5635	line*coccidia	2	2	334045.5	52624.48	40
5635	line*coccidia	2	3	154957.5	52624.48	40
5636	line	1		453197.3	112036.7	40
5636	line	2		1134512	112036.7	40
5636	coccidia		0	1358710	158443.8	40
5636	coccidia		1	996938.8	158443.8	40
5636	coccidia		2	819770.3	158443.8	40
5636	coccidia		3	5.82E-11	158443.8	40
5636	line*coccidia	1	0	1033509	224073.4	40
5636	line*coccidia	1	1	337257.7	224073.4	40
5636	line*coccidia	1	2	442022.2	224073.4	40
5636	line*coccidia	1	3	8.73E-11	224073.4	40
5636	line*coccidia	2	0	1683911	224073.4	40
5636	line*coccidia	2	1	1656620	224073.4	40
5636	line*coccidia	2	2	1197519	224073.4	40
5636	line*coccidia	2	3	2.91E-11	224073.4	40
5637	line	1		815268	86988.15	40
5637	line	2		467991.3	86988.15	40
5637	coccidia		0	704418.5	123019.8	40
5637	coccidia		1	320769.8	123019.8	40
5637	coccidia		2	213764.1	123019.8	40
5637	coccidia		3	1327566	123019.8	40
5637	line*coccidia	1	0	1097553	173976.3	40
5637	line*coccidia	1	1	264113.8	173976.3	40
5637	line*coccidia	1	2	288187	173976.3	40
5637	line*coccidia	1	3	1611218	173976.3	40
5637	line*coccidia	2	0	311283.7	173976.3	40
5637	line*coccidia	2	1	377425.7	173976.3	40
5637	line*coccidia	2	2	139341.2	173976.3	40
5637	line*coccidia	2	3	1043915	173976.3	40
5642	line	1		1592770	146523.4	40
5642	line	2		1092245	146523.4	40
5642	coccidia		0	1679653	207215.4	40
5642	coccidia		1	1035290	207215.4	40
5642	coccidia		2	776852.8	207215.4	40
5642	coccidia		3	1878232	207215.4	40
5642	line*coccidia	1	0	2063164	293046.8	40
5642	line*coccidia	1	1	917588.7	293046.8	40
5642	line*coccidia	1	2	651028.8	293046.8	40

5642	line*coccidia	1	3	2739298	293046.8	40
5642	line*coccidia	2	0	1296143	293046.8	40
5642	line*coccidia	2	1	1152992	293046.8	40
5642	line*coccidia	2	2	902676.8	293046.8	40
5642	line*coccidia	2	3	1017167	293046.8	40
5643	line	1		302720.5	55580.58	40
5643	line	2		646294.8	55580.58	40
5643	coccidia		0	606569.8	78602.8	40
5643	coccidia		1	474362.9	78602.8	40
5643	coccidia		2	366229.5	78602.8	40
5643	coccidia		3	450868.5	78602.8	40
5643	line*coccidia	1	0	284351.5	111161.2	40
5643	line*coccidia	1	1	297289.2	111161.2	40
5643	line*coccidia	1	2	151621	111161.2	40
5643	line*coccidia	1	3	477620.3	111161.2	40
5643	line*coccidia	2	0	928788	111161.2	40
5643	line*coccidia	2	1	651436.7	111161.2	40
5643	line*coccidia	2	2	580838	111161.2	40
5643	line*coccidia	2	3	424116.7	111161.2	40
5644	line	1		641049.2	107299.2	40
5644	line	2		726231.5	107299.2	40
5644	coccidia		0	936975	151744	40
5644	coccidia		1	659063.2	151744	40
5644	coccidia		2	572412	151744	40
5644	coccidia		3	566111.2	151744	40
5644	line*coccidia	1	0	1052138	214598.4	40
5644	line*coccidia	1	1	672836	214598.4	40
5644	line*coccidia	1	2	258888.3	214598.4	40
5644	line*coccidia	1	3	580334.7	214598.4	40
5644	line*coccidia	2	0	821812.2	214598.4	40
5644	line*coccidia	2	1	645290.3	214598.4	40
5644	line*coccidia	2	2	885935.7	214598.4	40
5644	line*coccidia	2	3	551887.7	214598.4	40
5645	line	1		1175622	137374.2	40
5645	line	2		1202011	137374.2	40
5645	coccidia		0	1521002	194276.5	40
5645	coccidia		1	1056752	194276.5	40
5645	coccidia		2	762880.6	194276.5	40
5645	coccidia		3	1414633	194276.5	40
5645	line*coccidia	1	0	1456324	274748.5	40
5645	line*coccidia	1	1	565717.7	274748.5	40
5645	line*coccidia	1	2	475354.3	274748.5	40
5645	line*coccidia	1	3	2205094	274748.5	40
5645	line*coccidia	2	0	1585679	274748.5	40
5645	line*coccidia	2	1	1547785	274748.5	40
5645	line*coccidia	2	2	1050407	274748.5	40
5645	line*coccidia	2	3	624171.8	274748.5	40
5646	line	1		24694.17	10400.17	40
5646	line	2		55155.46	10400.17	40

5646	coccidia		0	73256.25	14708.07	40
5646	coccidia		1	73429.67	14708.07	40
5646	coccidia		2	13013.33	14708.07	40
5646	coccidia		3	3.64E-12	14708.07	40
5646	line*coccidia	1	0	98776.67	20800.35	40
5646	line*coccidia	1	1	0	20800.35	40
5646	line*coccidia	1	2	0	20800.35	40
5646	line*coccidia	1	3	0	20800.35	40
5646	line*coccidia	2	0	47735.83	20800.35	40
5646	line*coccidia	2	1	146859.3	20800.35	40
5646	line*coccidia	2	2	26026.67	20800.35	40
5646	line*coccidia	2	3	7.28E-12	20800.35	40
5647	line	1		7.28E-12	13262.03	40
5647	line	2		138781.2	13262.03	40
5647	coccidia		0	200507.3	18755.34	40
5647	coccidia		1	-1.5E-11	18755.34	40
5647	coccidia		2	77055.08	18755.34	40
5647	coccidia		3	-1.5E-11	18755.34	40
5647	line*coccidia	1	0	5.09E-11	26524.06	40
5647	line*coccidia	1	1	-7.3E-12	26524.06	40
5647	line*coccidia	1	2	-7.3E-12	26524.06	40
5647	line*coccidia	1	3	-7.3E-12	26524.06	40
5647	line*coccidia	2	0	401014.5	26524.06	40
5647	line*coccidia	2	1	-2.2E-11	26524.06	40
5647	line*coccidia	2	2	154110.2	26524.06	40
5647	line*coccidia	2	3	-2.2E-11	26524.06	40
5648	line	1		135496.7	22205.19	40
5648	line	2		170879.5	22205.19	40
5648	coccidia		0	183723.3	31402.88	40
5648	coccidia		1	197538.9	31402.88	40
5648	coccidia		2	93992.58	31402.88	40
5648	coccidia		3	137497.5	31402.88	40
5648	line*coccidia	1	0	150760.2	44410.38	40
5648	line*coccidia	1	1	150912	44410.38	40
5648	line*coccidia	1	2	1.46E-11	44410.38	40
5648	line*coccidia	1	3	240314.5	44410.38	40
5648	line*coccidia	2	0	216686.3	44410.38	40
5648	line*coccidia	2	1	244165.8	44410.38	40
5648	line*coccidia	2	2	187985.2	44410.38	40
5648	line*coccidia	2	3	34680.5	44410.38	40
5649	line	1		360124.8	44777.81	40
5649	line	2		0	44777.81	40
5649	coccidia		0	348090.8	63325.39	40
5649	coccidia		1	116264.1	63325.39	40
5649	coccidia		2	110060.6	63325.39	40
5649	coccidia		3	145834.2	63325.39	40
5649	line*coccidia	1	0	696181.5	89555.62	40
5649	line*coccidia	1	1	232528.2	89555.62	40
5649	line*coccidia	1	2	220121.2	89555.62	40

5649	line*coccidia	1	3	291668.3	89555.62	40
5649	line*coccidia	2	0	-7.3E-12	89555.62	40
5649	line*coccidia	2	1	-7.3E-12	89555.62	40
5649	line*coccidia	2	2	3.64E-12	89555.62	40
5649	line*coccidia	2	3	1.09E-11	89555.62	40
5701	line	1		71443.5	20259.53	40
5701	line	2		103105.8	20259.53	40
5701	coccidia		0	77577.75	28651.3	40
5701	coccidia		1	114807.6	28651.3	40
5701	coccidia		2	71889.25	28651.3	40
5701	coccidia		3	84824	28651.3	40
5701	line*coccidia	1	0	53267.67	40519.06	40
5701	line*coccidia	1	1	47469.17	40519.06	40
5701	line*coccidia	1	2	72398.67	40519.06	40
5701	line*coccidia	1	3	112638.5	40519.06	40
5701	line*coccidia	2	0	101887.8	40519.06	40
5701	line*coccidia	2	1	182146	40519.06	40
5701	line*coccidia	2	2	71379.83	40519.06	40
5701	line*coccidia	2	3	57009.5	40519.06	40
5703	line	1		833263.1	86129.56	40
5703	line	2		528099.1	86129.56	40
5703	coccidia		0	901072.7	121805.6	40
5703	coccidia		1	592695.7	121805.6	40
5703	coccidia		2	492372	121805.6	40
5703	coccidia		3	736584	121805.6	40
5703	line*coccidia	1	0	1090201	172259.1	40
5703	line*coccidia	1	1	615572.3	172259.1	40
5703	line*coccidia	1	2	536418.5	172259.1	40
5703	line*coccidia	1	3	1090860	172259.1	40
5703	line*coccidia	2	0	711944	172259.1	40
5703	line*coccidia	2	1	569819	172259.1	40
5703	line*coccidia	2	2	448325.5	172259.1	40
5703	line*coccidia	2	3	382307.8	172259.1	40
5705	line	1		142592.5	25344.44	40
5705	line	2		65478.5	25344.44	40
5705	coccidia		0	55957.75	35842.45	40
5705	coccidia		1	58783	35842.45	40
5705	coccidia		2	117271.2	35842.45	40
5705	coccidia		3	184130.1	35842.45	40
5705	line*coccidia	1	0	70512.83	50688.88	40
5705	line*coccidia	1	1	21919.33	50688.88	40
5705	line*coccidia	1	2	188117.8	50688.88	40
5705	line*coccidia	1	3	289820	50688.88	40
5705	line*coccidia	2	0	41402.67	50688.88	40
5705	line*coccidia	2	1	95646.67	50688.88	40
5705	line*coccidia	2	2	46424.5	50688.88	40
5705	line*coccidia	2	3	78440.17	50688.88	40
5707	line	1		1615080	196668.2	40
5707	line	2		1911600	196668.2	40

5707	coccidia		0	2258450	278130.9	40
5707	coccidia		1	1682298	278130.9	40
5707	coccidia		2	1487069	278130.9	40
5707	coccidia		3	1625546	278130.9	40
5707	line*coccidia	1	0	2393481	393336.5	40
5707	line*coccidia	1	1	772190.5	393336.5	40
5707	line*coccidia	1	2	1090698	393336.5	40
5707	line*coccidia	1	3	2203952	393336.5	40
5707	line*coccidia	2	0	2123419	393336.5	40
5707	line*coccidia	2	1	2592405	393336.5	40
5707	line*coccidia	2	2	1883439	393336.5	40
5707	line*coccidia	2	3	1047139	393336.5	40
5708	line	1		76490.54	23686.63	40
5708	line	2		169932.3	23686.63	40
5708	coccidia		0	234084.8	33497.96	40
5708	coccidia		1	100085.5	33497.96	40
5708	coccidia		2	73159.08	33497.96	40
5708	coccidia		3	85516.17	33497.96	40
5708	line*coccidia	1	0	154972	47373.27	40
5708	line*coccidia	1	1	19913.83	47373.27	40
5708	line*coccidia	1	2	33255.33	47373.27	40
5708	line*coccidia	1	3	97821	47373.27	40
5708	line*coccidia	2	0	313197.7	47373.27	40
5708	line*coccidia	2	1	180257.2	47373.27	40
5708	line*coccidia	2	2	113062.8	47373.27	40
5708	line*coccidia	2	3	73211.33	47373.27	40
5709	line	1		237760.5	60065.48	40
5709	line	2		366712	60065.48	40
5709	coccidia		0	341411.5	84945.41	40
5709	coccidia		1	287636.3	84945.41	40
5709	coccidia		2	256008.5	84945.41	40
5709	coccidia		3	323888.6	84945.41	40
5709	line*coccidia	1	0	388692.2	120131	40
5709	line*coccidia	1	1	73344.5	120131	40
5709	line*coccidia	1	2	68613.5	120131	40
5709	line*coccidia	1	3	420391.8	120131	40
5709	line*coccidia	2	0	294130.8	120131	40
5709	line*coccidia	2	1	501928.2	120131	40
5709	line*coccidia	2	2	443403.5	120131	40
5709	line*coccidia	2	3	227385.3	120131	40
5710	line	1		2913971	355893	40
5710	line	2		3595266	355893	40
5710	coccidia		0	4427623	503308.7	40
5710	coccidia		1	2736918	503308.7	40
5710	coccidia		2	2335247	503308.7	40
5710	coccidia		3	3518685	503308.7	40
5710	line*coccidia	1	0	4713472	711786	40
5710	line*coccidia	1	1	1081720	711786	40
5710	line*coccidia	1	2	1480410	711786	40

5710	line*coccidia	1	3	4380282	711786	40
5710	line*coccidia	2	0	4141775	711786	40
5710	line*coccidia	2	1	4392116	711786	40
5710	line*coccidia	2	2	3190085	711786	40
5710	line*coccidia	2	3	2657087	711786	40
5711	line	1		203494.7	61612.4	40
5711	line	2		128861.3	61612.4	40
5711	coccidia		0	49618.5	87133.09	40
5711	coccidia		1	134448.6	87133.09	40
5711	coccidia		2	82782.5	87133.09	40
5711	coccidia		3	397862.3	87133.09	40
5711	line*coccidia	1	0	83469	123224.8	40
5711	line*coccidia	1	1	109151.8	123224.8	40
5711	line*coccidia	1	2	119381.8	123224.8	40
5711	line*coccidia	1	3	501976	123224.8	40
5711	line*coccidia	2	0	15768	123224.8	40
5711	line*coccidia	2	1	159745.3	123224.8	40
5711	line*coccidia	2	2	46183.17	123224.8	40
5711	line*coccidia	2	3	293748.5	123224.8	40
5712	line	1		2.18E-11	24435.46	40
5712	line	2		147129	24435.46	40
5712	coccidia		0	109844.1	34556.96	40
5712	coccidia		1	84345.75	34556.96	40
5712	coccidia		2	83782.92	34556.96	40
5712	coccidia		3	16285.25	34556.96	40
5712	line*coccidia	1	0	2.18E-11	48870.93	40
5712	line*coccidia	1	1	2.18E-11	48870.93	40
5712	line*coccidia	1	2	2.18E-11	48870.93	40
5712	line*coccidia	1	3	2.18E-11	48870.93	40
5712	line*coccidia	2	0	219688.2	48870.93	40
5712	line*coccidia	2	1	168691.5	48870.93	40
5712	line*coccidia	2	2	167565.8	48870.93	40
5712	line*coccidia	2	3	32570.5	48870.93	40
5713	line	1		3592178	520918	40
5713	line	2		5404018	520918	40
5713	coccidia		0	5039469	736689.3	40
5713	coccidia		1	4729898	736689.3	40
5713	coccidia		2	3411610	736689.3	40
5713	coccidia		3	4811415	736689.3	40
5713	line*coccidia	1	0	4922047	1041836	40
5713	line*coccidia	1	1	1712011	1041836	40
5713	line*coccidia	1	2	1570872	1041836	40
5713	line*coccidia	1	3	6163781	1041836	40
5713	line*coccidia	2	0	5156890	1041836	40
5713	line*coccidia	2	1	7747785	1041836	40
5713	line*coccidia	2	2	5252348	1041836	40
5713	line*coccidia	2	3	3459048	1041836	40
5714	line	1		313463.4	76276.74	40
5714	line	2		539288.4	76276.74	40

5714	coccidia		0	487186.4	107871.6	40
5714	coccidia		1	533934.7	107871.6	40
5714	coccidia		2	244036.4	107871.6	40
5714	coccidia		3	440346.1	107871.6	40
5714	line*coccidia	1	0	441284.8	152553.5	40
5714	line*coccidia	1	1	105965	152553.5	40
5714	line*coccidia	1	2	102786.8	152553.5	40
5714	line*coccidia	1	3	603817	152553.5	40
5714	line*coccidia	2	0	533088	152553.5	40
5714	line*coccidia	2	1	961904.3	152553.5	40
5714	line*coccidia	2	2	385286	152553.5	40
5714	line*coccidia	2	3	276875.2	152553.5	40
5715	line	1		229976.4	20645.41	40
5715	line	2		131683.7	20645.41	40
5715	coccidia		0	121155.8	29197.02	40
5715	coccidia		1	218541	29197.02	40
5715	coccidia		2	93355.42	29197.02	40
5715	coccidia		3	290268	29197.02	40
5715	line*coccidia	1	0	188164.5	41290.83	40
5715	line*coccidia	1	1	158953.7	41290.83	40
5715	line*coccidia	1	2	121592.8	41290.83	40
5715	line*coccidia	1	3	451194.7	41290.83	40
5715	line*coccidia	2	0	54147	41290.83	40
5715	line*coccidia	2	1	278128.3	41290.83	40
5715	line*coccidia	2	2	65118	41290.83	40
5715	line*coccidia	2	3	129341.3	41290.83	40
5716	line	1		1969303	253627.7	40
5716	line	2		2882814	253627.7	40
5716	coccidia		0	2575697	358683.7	40
5716	coccidia		1	2453105	358683.7	40
5716	coccidia		2	1923368	358683.7	40
5716	coccidia		3	2752065	358683.7	40
5716	line*coccidia	1	0	2387681	507255.3	40
5716	line*coccidia	1	1	1326022	507255.3	40
5716	line*coccidia	1	2	696119.7	507255.3	40
5716	line*coccidia	1	3	3467390	507255.3	40
5716	line*coccidia	2	0	2763712	507255.3	40
5716	line*coccidia	2	1	3580188	507255.3	40
5716	line*coccidia	2	2	3150617	507255.3	40
5716	line*coccidia	2	3	2036740	507255.3	40
5717	line	1		148556.5	44846	40
5717	line	2		255274.1	44846	40
5717	coccidia		0	254736.4	63421.83	40
5717	coccidia		1	230425.6	63421.83	40
5717	coccidia		2	162977.7	63421.83	40
5717	coccidia		3	159521.6	63421.83	40
5717	line*coccidia	1	0	171379	89692.01	40
5717	line*coccidia	1	1	57757.17	89692.01	40
5717	line*coccidia	1	2	121329.7	89692.01	40

5717	line*coccidia	1	3	243760.2	89692.01	40
5717	line*coccidia	2	0	338093.8	89692.01	40
5717	line*coccidia	2	1	403094	89692.01	40
5717	line*coccidia	2	2	204625.7	89692.01	40
5717	line*coccidia	2	3	75283	89692.01	40
5718	line	1		208262.5	26537.07	40
5718	line	2		194842.3	26537.07	40
5718	coccidia		0	185219.3	37529.08	40
5718	coccidia		1	293433.8	37529.08	40
5718	coccidia		2	123309.1	37529.08	40
5718	coccidia		3	204247.3	37529.08	40
5718	line*coccidia	1	0	244276.5	53074.14	40
5718	line*coccidia	1	1	159053	53074.14	40
5718	line*coccidia	1	2	125687.8	53074.14	40
5718	line*coccidia	1	3	304032.7	53074.14	40
5718	line*coccidia	2	0	126162.2	53074.14	40
5718	line*coccidia	2	1	427814.5	53074.14	40
5718	line*coccidia	2	2	120930.3	53074.14	40
5718	line*coccidia	2	3	104462	53074.14	40
5719	line	1		338624.3	105827.6	40
5719	line	2		77681.38	105827.6	40
5719	coccidia		0	101710.3	149662.8	40
5719	coccidia		1	514186.7	149662.8	40
5719	coccidia		2	126340.8	149662.8	40
5719	coccidia		3	90373.58	149662.8	40
5719	line*coccidia	1	0	136585	211655.2	40
5719	line*coccidia	1	1	836364.3	211655.2	40
5719	line*coccidia	1	2	224822	211655.2	40
5719	line*coccidia	1	3	156725.8	211655.2	40
5719	line*coccidia	2	0	66835.67	211655.2	40
5719	line*coccidia	2	1	192009	211655.2	40
5719	line*coccidia	2	2	27859.5	211655.2	40
5719	line*coccidia	2	3	24021.33	211655.2	40
5720	line	1		1903323	548191.3	40
5720	line	2		1764401	548191.3	40
5720	coccidia		0	744302.3	775259.5	40
5720	coccidia		1	4284602	775259.5	40
5720	coccidia		2	1241017	775259.5	40
5720	coccidia		3	1065526	775259.5	40
5720	line*coccidia	1	0	781025.3	1096383	40
5720	line*coccidia	1	1	4011285	1096383	40
5720	line*coccidia	1	2	1274563	1096383	40
5720	line*coccidia	1	3	1546418	1096383	40
5720	line*coccidia	2	0	707579.3	1096383	40
5720	line*coccidia	2	1	4557919	1096383	40
5720	line*coccidia	2	2	1207471	1096383	40
5720	line*coccidia	2	3	584634.7	1096383	40
5721	line	1		1074786	158335.1	40
5721	line	2		704411.9	158335.1	40

5721	coccidia		0	808586.6	223919.6	40
5721	coccidia		1	1117595	223919.6	40
5721	coccidia		2	638721.9	223919.6	40
5721	coccidia		3	993491.9	223919.6	40
5721	line*coccidia	1	0	1150021	316670.1	40
5721	line*coccidia	1	1	1190010	316670.1	40
5721	line*coccidia	1	2	461039.2	316670.1	40
5721	line*coccidia	1	3	1498073	316670.1	40
5721	line*coccidia	2	0	467151.8	316670.1	40
5721	line*coccidia	2	1	1045181	316670.1	40
5721	line*coccidia	2	2	816404.7	316670.1	40
5721	line*coccidia	2	3	488910.7	316670.1	40
5722	line	1		173904.3	22182.67	40
5722	line	2		87763.96	22182.67	40
5722	coccidia		0	128388.8	31371.04	40
5722	coccidia		1	103456.4	31371.04	40
5722	coccidia		2	71811.75	31371.04	40
5722	coccidia		3	219679.5	31371.04	40
5722	line*coccidia	1	0	176402.7	44365.35	40
5722	line*coccidia	1	1	91414.67	44365.35	40
5722	line*coccidia	1	2	91435.17	44365.35	40
5722	line*coccidia	1	3	336364.5	44365.35	40
5722	line*coccidia	2	0	80374.83	44365.35	40
5722	line*coccidia	2	1	115498.2	44365.35	40
5722	line*coccidia	2	2	52188.33	44365.35	40
5722	line*coccidia	2	3	102994.5	44365.35	40
5723	line	1		270515.5	35538.38	40
5723	line	2		258421.5	35538.38	40
5723	coccidia		0	358422.1	50258.86	40
5723	coccidia		1	213680.6	50258.86	40
5723	coccidia		2	199159.4	50258.86	40
5723	coccidia		3	286611.9	50258.86	40
5723	line*coccidia	1	0	287095.5	71076.76	40
5723	line*coccidia	1	1	161625.3	71076.76	40
5723	line*coccidia	1	2	251381.2	71076.76	40
5723	line*coccidia	1	3	381960.2	71076.76	40
5723	line*coccidia	2	0	429748.7	71076.76	40
5723	line*coccidia	2	1	265735.8	71076.76	40
5723	line*coccidia	2	2	146937.7	71076.76	40
5723	line*coccidia	2	3	191263.7	71076.76	40
5724	line	1		1474216	259683.5	40
5724	line	2		1327996	259683.5	40
5724	coccidia		0	1339578	367248	40
5724	coccidia		1	1659630	367248	40
5724	coccidia		2	974807.9	367248	40
5724	coccidia		3	1630409	367248	40
5724	line*coccidia	1	0	1565922	519367.1	40
5724	line*coccidia	1	1	1072040	519367.1	40
5724	line*coccidia	1	2	689957	519367.1	40

5724	line*coccidia	1	3	2568947	519367.1	40
5724	line*coccidia	2	0	1113235	519367.1	40
5724	line*coccidia	2	1	2247221	519367.1	40
5724	line*coccidia	2	2	1259659	519367.1	40
5724	line*coccidia	2	3	691870.5	519367.1	40
5725	line	1		325681.5	72870.8	40
5725	line	2		134007.2	72870.8	40
5725	coccidia		0	136820.8	103054.9	40
5725	coccidia		1	580887.9	103054.9	40
5725	coccidia		2	116705.3	103054.9	40
5725	coccidia		3	84963.25	103054.9	40
5725	line*coccidia	1	0	131602.5	145741.6	40
5725	line*coccidia	1	1	906131.5	145741.6	40
5725	line*coccidia	1	2	119695.2	145741.6	40
5725	line*coccidia	1	3	145296.7	145741.6	40
5725	line*coccidia	2	0	142039.2	145741.6	40
5725	line*coccidia	2	1	255644.3	145741.6	40
5725	line*coccidia	2	2	113715.5	145741.6	40
5725	line*coccidia	2	3	24629.83	145741.6	40
5726	line	1		5.09E-11	29126.54	40
5726	line	2		300916.1	29126.54	40
5726	coccidia		0	0	41191.15	40
5726	coccidia		1	301399.3	41191.15	40
5726	coccidia		2	211056.2	41191.15	40
5726	coccidia		3	89376.67	41191.15	40
5726	line*coccidia	1	0	0	58253.08	40
5726	line*coccidia	1	1	8.73E-11	58253.08	40
5726	line*coccidia	1	2	2.91E-11	58253.08	40
5726	line*coccidia	1	3	8.73E-11	58253.08	40
5726	line*coccidia	2	0	0	58253.08	40
5726	line*coccidia	2	1	602798.7	58253.08	40
5726	line*coccidia	2	2	422112.3	58253.08	40
5726	line*coccidia	2	3	178753.3	58253.08	40
5728	line	1		42127.79	10493.23	40
5728	line	2		-1.3E-12	10493.23	40
5728	coccidia		0	33904.75	14839.67	40
5728	coccidia		1	50350.83	14839.67	40
5728	coccidia		2	1.21E-12	14839.67	40
5728	coccidia		3	-3.6E-12	14839.67	40
5728	line*coccidia	1	0	67809.5	20986.46	40
5728	line*coccidia	1	1	100701.7	20986.46	40
5728	line*coccidia	1	2	2.14E-12	20986.46	40
5728	line*coccidia	1	3	-2.7E-12	20986.46	40
5728	line*coccidia	2	0	-3.6E-12	20986.46	40
5728	line*coccidia	2	1	2.71E-12	20986.46	40
5728	line*coccidia	2	2	2.82E-13	20986.46	40
5728	line*coccidia	2	3	-4.6E-12	20986.46	40
5801	line	1		1190386	194103.1	40
5801	line	2		1499533	194103.1	40

5801	coccidia		0	1581774	274503.2	40
5801	coccidia		1	1968174	274503.2	40
5801	coccidia		2	721332.9	274503.2	40
5801	coccidia		3	1108556	274503.2	40
5801	line*coccidia	1	0	966698.2	388206.1	40
5801	line*coccidia	1	1	1810489	388206.1	40
5801	line*coccidia	1	2	511918.8	388206.1	40
5801	line*coccidia	1	3	1472437	388206.1	40
5801	line*coccidia	2	0	2196850	388206.1	40
5801	line*coccidia	2	1	2125859	388206.1	40
5801	line*coccidia	2	2	930747	388206.1	40
5801	line*coccidia	2	3	744675.5	388206.1	40
5806	line	1		1488733	298721.5	40
5806	line	2		1602986	298721.5	40
5806	coccidia		0	1821941	422455.9	40
5806	coccidia		1	2055048	422455.9	40
5806	coccidia		2	1175470	422455.9	40
5806	coccidia		3	1130979	422455.9	40
5806	line*coccidia	1	0	1639512	597442.9	40
5806	line*coccidia	1	1	2289157	597442.9	40
5806	line*coccidia	1	2	516659.7	597442.9	40
5806	line*coccidia	1	3	1509602	597442.9	40
5806	line*coccidia	2	0	2004371	597442.9	40
5806	line*coccidia	2	1	1820938	597442.9	40
5806	line*coccidia	2	2	1834280	597442.9	40
5806	line*coccidia	2	3	752356.2	597442.9	40
5807	line	1		313130.4	57074.19	40
5807	line	2		389780.7	57074.19	40
5807	coccidia		0	279662.3	80715.1	40
5807	coccidia		1	567647.8	80715.1	40
5807	coccidia		2	242045.2	80715.1	40
5807	coccidia		3	316466.8	80715.1	40
5807	line*coccidia	1	0	308612.2	114148.4	40
5807	line*coccidia	1	1	374369.7	114148.4	40
5807	line*coccidia	1	2	148138.2	114148.4	40
5807	line*coccidia	1	3	421401.5	114148.4	40
5807	line*coccidia	2	0	250712.5	114148.4	40
5807	line*coccidia	2	1	760925.8	114148.4	40
5807	line*coccidia	2	2	335952.2	114148.4	40
5807	line*coccidia	2	3	211532.2	114148.4	40
5811	line	1		1673885	305705.5	40
5811	line	2		1645229	305705.5	40
5811	coccidia		0	2035457	432332.8	40
5811	coccidia		1	2309987	432332.8	40
5811	coccidia		2	903918.8	432332.8	40
5811	coccidia		3	1388865	432332.8	40
5811	line*coccidia	1	0	1601886	611411	40
5811	line*coccidia	1	1	2875727	611411	40
5811	line*coccidia	1	2	609524.2	611411	40

5811	line*coccidia	1	3	1608405	611411	40
5811	line*coccidia	2	0	2469029	611411	40
5811	line*coccidia	2	1	1744247	611411	40
5811	line*coccidia	2	2	1198314	611411	40
5811	line*coccidia	2	3	1169325	611411	40
5813	line	1		1772772	412017.8	40
5813	line	2		2307506	412017.8	40
5813	coccidia		0	1958880	582681.2	40
5813	coccidia		1	3816772	582681.2	40
5813	coccidia		2	1089108	582681.2	40
5813	coccidia		3	1295796	582681.2	40
5813	line*coccidia	1	0	1599494	824035.7	40
5813	line*coccidia	1	1	3034868	824035.7	40
5813	line*coccidia	1	2	795580	824035.7	40
5813	line*coccidia	1	3	1661147	824035.7	40
5813	line*coccidia	2	0	2318265	824035.7	40
5813	line*coccidia	2	1	4598676	824035.7	40
5813	line*coccidia	2	2	1382635	824035.7	40
5813	line*coccidia	2	3	930446.2	824035.7	40
5821	line	1		603237.5	298359.4	40
5821	line	2		1252243	298359.4	40
5821	coccidia		0	913337.2	421943.9	40
5821	coccidia		1	1360879	421943.9	40
5821	coccidia		2	842552.3	421943.9	40
5821	coccidia		3	594193.4	421943.9	40
5821	line*coccidia	1	0	498821.5	596718.8	40
5821	line*coccidia	1	1	501116	596718.8	40
5821	line*coccidia	1	2	446064	596718.8	40
5821	line*coccidia	1	3	966948.7	596718.8	40
5821	line*coccidia	2	0	1327853	596718.8	40
5821	line*coccidia	2	1	2220642	596718.8	40
5821	line*coccidia	2	2	1239041	596718.8	40
5821	line*coccidia	2	3	221438.2	596718.8	40
5825	line	1		1132458	394454.5	40
5825	line	2		1615688	394454.5	40
5825	coccidia		0	1341162	557842.9	40
5825	coccidia		1	2346672	557842.9	40
5825	coccidia		2	699317.6	557842.9	40
5825	coccidia		3	1109140	557842.9	40
5825	line*coccidia	1	0	1131765	788909.1	40
5825	line*coccidia	1	1	1386369	788909.1	40
5825	line*coccidia	1	2	268590.5	788909.1	40
5825	line*coccidia	1	3	1743107	788909.1	40
5825	line*coccidia	2	0	1550558	788909.1	40
5825	line*coccidia	2	1	3306975	788909.1	40
5825	line*coccidia	2	2	1130045	788909.1	40
5825	line*coccidia	2	3	475172.7	788909.1	40
5828	line	1		0	28647.97	40
5828	line	2		263976.9	28647.97	40

5828	coccidia		0	76728.83	40514.35	40
5828	coccidia		1	166915.4	40514.35	40
5828	coccidia		2	101258.8	40514.35	40
5828	coccidia		3	183050.8	40514.35	40
5828	line*coccidia	1	0	0	57295.94	40
5828	line*coccidia	1	1	0	57295.94	40
5828	line*coccidia	1	2	0	57295.94	40
5828	line*coccidia	1	3	0	57295.94	40
5828	line*coccidia	2	0	153457.7	57295.94	40
5828	line*coccidia	2	1	333830.8	57295.94	40
5828	line*coccidia	2	2	202517.5	57295.94	40
5828	line*coccidia	2	3	366101.5	57295.94	40
5830	line	1		118158.4	32497.51	40
5830	line	2		70494.42	32497.51	40
5830	coccidia		0	137687.6	45958.42	40
5830	coccidia		1	125499.1	45958.42	40
5830	coccidia		2	72111.83	45958.42	40
5830	coccidia		3	42007.17	45958.42	40
5830	line*coccidia	1	0	190563.5	64995.02	40
5830	line*coccidia	1	1	110071.7	64995.02	40
5830	line*coccidia	1	2	121667.3	64995.02	40
5830	line*coccidia	1	3	50331.17	64995.02	40
5830	line*coccidia	2	0	84811.67	64995.02	40
5830	line*coccidia	2	1	140926.5	64995.02	40
5830	line*coccidia	2	2	22556.33	64995.02	40
5830	line*coccidia	2	3	33683.17	64995.02	40
5835	line	1		207921.8	32847.38	40
5835	line	2		215339.2	32847.38	40
5835	coccidia		0	271441.7	46453.22	40
5835	coccidia		1	288813.8	46453.22	40
5835	coccidia		2	113841.9	46453.22	40
5835	coccidia		3	172424.7	46453.22	40
5835	line*coccidia	1	0	390446	65694.77	40
5835	line*coccidia	1	1	153429.5	65694.77	40
5835	line*coccidia	1	2	38370.5	65694.77	40
5835	line*coccidia	1	3	249441.2	65694.77	40
5835	line*coccidia	2	0	152437.3	65694.77	40
5835	line*coccidia	2	1	424198	65694.77	40
5835	line*coccidia	2	2	189313.3	65694.77	40
5835	line*coccidia	2	3	95408.17	65694.77	40
5836	line	1		1224041	329649.7	40
5836	line	2		1271956	329649.7	40
5836	coccidia		0	1414418	466195.1	40
5836	coccidia		1	1805101	466195.1	40
5836	coccidia		2	654199.8	466195.1	40
5836	coccidia		3	1118275	466195.1	40
5836	line*coccidia	1	0	518207.7	659299.5	40
5836	line*coccidia	1	1	2494239	659299.5	40
5836	line*coccidia	1	2	275363.3	659299.5	40

5836	line*coccidia	1	3	1608355	659299.5	40
5836	line*coccidia	2	0	2310628	659299.5	40
5836	line*coccidia	2	1	1115963	659299.5	40
5836	line*coccidia	2	2	1033036	659299.5	40
5836	line*coccidia	2	3	628195.2	659299.5	40
5837	line	1		242816.7	43152.91	40
5837	line	2		259121.9	43152.91	40
5837	coccidia		0	282738.7	61027.44	40
5837	coccidia		1	251632	61027.44	40
5837	coccidia		2	150444	61027.44	40
5837	coccidia		3	319062.6	61027.44	40
5837	line*coccidia	1	0	345992.2	86305.83	40
5837	line*coccidia	1	1	107749.7	86305.83	40
5837	line*coccidia	1	2	169317.7	86305.83	40
5837	line*coccidia	1	3	348207.3	86305.83	40
5837	line*coccidia	2	0	219485.2	86305.83	40
5837	line*coccidia	2	1	395514.3	86305.83	40
5837	line*coccidia	2	2	131570.3	86305.83	40
5837	line*coccidia	2	3	289917.8	86305.83	40
5838	line	1		87520.48	14989.54	39
5838	line	2		103867.8	14628.29	39
5838	coccidia		0	109498.7	20687.53	39
5838	coccidia		1	117805.1	20687.53	39
5838	coccidia		2	44279.12	21697.26	39
5838	coccidia		3	111193.8	20687.53	39
5838	line*coccidia	1	0	105343.5	29256.58	39
5838	line*coccidia	1	1	67867.5	29256.58	39
5838	line*coccidia	1	2	41185.4	32048.98	39
5838	line*coccidia	1	3	135685.5	29256.58	39
5838	line*coccidia	2	0	113653.8	29256.58	39
5838	line*coccidia	2	1	167742.7	29256.58	39
5838	line*coccidia	2	2	47372.83	29256.58	39
5838	line*coccidia	2	3	86702	29256.58	39
5839	line	1		7.28E-12	31423.73	40
5839	line	2		293501.4	31423.73	40
5839	coccidia		0	67536.58	44439.87	40
5839	coccidia		1	209720.3	44439.87	40
5839	coccidia		2	146169.5	44439.87	40
5839	coccidia		3	163576.3	44439.87	40
5839	line*coccidia	1	0	2.91E-11	62847.46	40
5839	line*coccidia	1	1	0	62847.46	40
5839	line*coccidia	1	2	0	62847.46	40
5839	line*coccidia	1	3	0	62847.46	40
5839	line*coccidia	2	0	135073.2	62847.46	40
5839	line*coccidia	2	1	419440.7	62847.46	40
5839	line*coccidia	2	2	292339	62847.46	40
5839	line*coccidia	2	3	327152.7	62847.46	40
5840	line	1		2.55E-11	13799.09	40
5840	line	2		138269.3	13799.09	40

5840	coccidia		0	63364.83	19514.86	40
5840	coccidia		1	80265.92	19514.86	40
5840	coccidia		2	105320.2	19514.86	40
5840	coccidia		3	27587.75	19514.86	40
5840	line*coccidia	1	0	4.37E-11	27598.18	40
5840	line*coccidia	1	1	2.91E-11	27598.18	40
5840	line*coccidia	1	2	1.46E-11	27598.18	40
5840	line*coccidia	1	3	1.46E-11	27598.18	40
5840	line*coccidia	2	0	126729.7	27598.18	40
5840	line*coccidia	2	1	160531.8	27598.18	40
5840	line*coccidia	2	2	210640.3	27598.18	40
5840	line*coccidia	2	3	55175.5	27598.18	40
5915	line	1		763420	79210.22	40
5915	line	2		501772.3	79210.22	40
5915	coccidia		0	699797.6	112020.2	40
5915	coccidia		1	588412.7	112020.2	40
5915	coccidia		2	525944.5	112020.2	40
5915	coccidia		3	716229.9	112020.2	40
5915	line*coccidia	1	0	856635	158420.4	40
5915	line*coccidia	1	1	665298.2	158420.4	40
5915	line*coccidia	1	2	391032.8	158420.4	40
5915	line*coccidia	1	3	1140714	158420.4	40
5915	line*coccidia	2	0	542960.2	158420.4	40
5915	line*coccidia	2	1	511527.2	158420.4	40
5915	line*coccidia	2	2	660856.2	158420.4	40
5915	line*coccidia	2	3	291745.8	158420.4	40
6001	line	1		50114.92	12120.71	40
6001	line	2		72538.17	12120.71	40
6001	coccidia		0	64154.58	17141.27	40
6001	coccidia		1	67901.58	17141.27	40
6001	coccidia		2	51864.5	17141.27	40
6001	coccidia		3	61385.5	17141.27	40
6001	line*coccidia	1	0	59517.5	24241.42	40
6001	line*coccidia	1	1	49395.17	24241.42	40
6001	line*coccidia	1	2	33391.5	24241.42	40
6001	line*coccidia	1	3	58155.5	24241.42	40
6001	line*coccidia	2	0	68791.67	24241.42	40
6001	line*coccidia	2	1	86408	24241.42	40
6001	line*coccidia	2	2	70337.5	24241.42	40
6001	line*coccidia	2	3	64615.5	24241.42	40
6002	line	1		26936.79	6560.678	40
6002	line	2		51459.58	6560.678	40
6002	coccidia		0	46312.75	9278.2	40
6002	coccidia		1	37406.67	9278.2	40
6002	coccidia		2	42019.08	9278.2	40
6002	coccidia		3	31054.25	9278.2	40
6002	line*coccidia	1	0	24308.67	13121.36	40
6002	line*coccidia	1	1	-3.6E-12	13121.36	40
6002	line*coccidia	1	2	38995.33	13121.36	40

6002	line*coccidia	1	3	44443.17	13121.36	40
6002	line*coccidia	2	0	68316.83	13121.36	40
6002	line*coccidia	2	1	74813.33	13121.36	40
6002	line*coccidia	2	2	45042.83	13121.36	40
6002	line*coccidia	2	3	17665.33	13121.36	40
6010	line	1		186027.9	41287.05	40
6010	line	2		328238.8	41287.05	40
6010	coccidia		0	345172	58388.71	40
6010	coccidia		1	217122.6	58388.71	40
6010	coccidia		2	227311.4	58388.71	40
6010	coccidia		3	238927.4	58388.71	40
6010	line*coccidia	1	0	270892.7	82574.1	40
6010	line*coccidia	1	1	78607.33	82574.1	40
6010	line*coccidia	1	2	99803.83	82574.1	40
6010	line*coccidia	1	3	294807.7	82574.1	40
6010	line*coccidia	2	0	419451.3	82574.1	40
6010	line*coccidia	2	1	355637.8	82574.1	40
6010	line*coccidia	2	2	354819	82574.1	40
6010	line*coccidia	2	3	183047.2	82574.1	40
6011	line	1		40073.92	6377.283	40
6011	line	2		55129.38	6377.283	40
6011	coccidia		0	51497.25	9018.84	40
6011	coccidia		1	19950.17	9018.84	40
6011	coccidia		2	63628.67	9018.84	40
6011	coccidia		3	55330.5	9018.84	40
6011	line*coccidia	1	0	29391	12754.57	40
6011	line*coccidia	1	1	1.46E-11	12754.57	40
6011	line*coccidia	1	2	63159.67	12754.57	40
6011	line*coccidia	1	3	67745	12754.57	40
6011	line*coccidia	2	0	73603.5	12754.57	40
6011	line*coccidia	2	1	39900.33	12754.57	40
6011	line*coccidia	2	2	64097.67	12754.57	40
6011	line*coccidia	2	3	42916	12754.57	40
6014	line	1		65325.58	14520.03	40
6014	line	2		93949.54	14520.03	40
6014	coccidia		0	91525.25	20534.42	40
6014	coccidia		1	85762.42	20534.42	40
6014	coccidia		2	78223.33	20534.42	40
6014	coccidia		3	63039.25	20534.42	40
6014	line*coccidia	1	0	75919.83	29040.06	40
6014	line*coccidia	1	1	35330	29040.06	40
6014	line*coccidia	1	2	78180.67	29040.06	40
6014	line*coccidia	1	3	71871.83	29040.06	40
6014	line*coccidia	2	0	107130.7	29040.06	40
6014	line*coccidia	2	1	136194.8	29040.06	40
6014	line*coccidia	2	2	78266	29040.06	40
6014	line*coccidia	2	3	54206.67	29040.06	40
6015	line	1		112409.3	27956.62	40
6015	line	2		26343.42	27956.62	40

6015	coccidia		0	19879.75	39536.63	40
6015	coccidia		1	239298	39536.63	40
6015	coccidia		2	9910.167	39536.63	40
6015	coccidia		3	8417.417	39536.63	40
6015	line*coccidia	1	0	7361.333	55913.24	40
6015	line*coccidia	1	1	442275.7	55913.24	40
6015	line*coccidia	1	2	2.27E-11	55913.24	40
6015	line*coccidia	1	3	3.64E-12	55913.24	40
6015	line*coccidia	2	0	32398.17	55913.24	40
6015	line*coccidia	2	1	36320.33	55913.24	40
6015	line*coccidia	2	2	19820.33	55913.24	40
6015	line*coccidia	2	3	16834.83	55913.24	40
6017	line	1		211820.7	50729.11	40
6017	line	2		49525.54	50729.11	40
6017	coccidia		0	28623	71741.79	40
6017	coccidia		1	394361.8	71741.79	40
6017	coccidia		2	36350.75	71741.79	40
6017	coccidia		3	63357	71741.79	40
6017	line*coccidia	1	0	12443.17	101458.2	40
6017	line*coccidia	1	1	762047	101458.2	40
6017	line*coccidia	1	2	43484	101458.2	40
6017	line*coccidia	1	3	29308.67	101458.2	40
6017	line*coccidia	2	0	44802.83	101458.2	40
6017	line*coccidia	2	1	26676.5	101458.2	40
6017	line*coccidia	2	2	29217.5	101458.2	40
6017	line*coccidia	2	3	97405.33	101458.2	40
6019	line	1		26048.88	3273.408	40
6019	line	2		21806.25	3273.408	40
6019	coccidia		0	21711	4629.298	40
6019	coccidia		1	23881.58	4629.298	40
6019	coccidia		2	20330.08	4629.298	40
6019	coccidia		3	29787.58	4629.298	40
6019	line*coccidia	1	0	22784.83	6546.815	40
6019	line*coccidia	1	1	23303	6546.815	40
6019	line*coccidia	1	2	26803	6546.815	40
6019	line*coccidia	1	3	31304.67	6546.815	40
6019	line*coccidia	2	0	20637.17	6546.815	40
6019	line*coccidia	2	1	24460.17	6546.815	40
6019	line*coccidia	2	2	13857.17	6546.815	40
6019	line*coccidia	2	3	28270.5	6546.815	40
6022	line	1		14289.58	1924.013	40
6022	line	2		-1.1E-13	1924.013	40
6022	coccidia		0	4458.083	2720.966	40
6022	coccidia		1	5315.167	2720.966	40
6022	coccidia		2	11174.83	2720.966	40
6022	coccidia		3	7631.083	2720.966	40
6022	line*coccidia	1	0	8916.167	3848.027	40
6022	line*coccidia	1	1	10630.33	3848.027	40
6022	line*coccidia	1	2	22349.67	3848.027	40

6022	line*coccidia	1	3	15262.17	3848.027	40
6022	line*coccidia	2	0	-4.5E-13	3848.027	40
6022	line*coccidia	2	1	-4.5E-13	3848.027	40
6022	line*coccidia	2	2	-1.1E-12	3848.027	40
6022	line*coccidia	2	3	1.59E-12	3848.027	40
6101	line	1		70086.96	12973.39	40
6101	line	2		102714.2	12973.39	40
6101	coccidia		0	97175.33	18347.15	40
6101	coccidia		1	123367.3	18347.15	40
6101	coccidia		2	46790.08	18347.15	40
6101	coccidia		3	78269.67	18347.15	40
6101	line*coccidia	1	0	95552.67	25946.79	40
6101	line*coccidia	1	1	49194.17	25946.79	40
6101	line*coccidia	1	2	30013.83	25946.79	40
6101	line*coccidia	1	3	105587.2	25946.79	40
6101	line*coccidia	2	0	98798	25946.79	40
6101	line*coccidia	2	1	197540.3	25946.79	40
6101	line*coccidia	2	2	63566.33	25946.79	40
6101	line*coccidia	2	3	50952.17	25946.79	40
6103	line	1		35996.42	16211.52	40
6103	line	2		80971.58	16211.52	40
6103	coccidia		0	20611.5	22926.56	40
6103	coccidia		1	106385.1	22926.56	40
6103	coccidia		2	34831.83	22926.56	40
6103	coccidia		3	72107.58	22926.56	40
6103	line*coccidia	1	0	8751.5	32423.05	40
6103	line*coccidia	1	1	69038.17	32423.05	40
6103	line*coccidia	1	2	16196	32423.05	40
6103	line*coccidia	1	3	50000	32423.05	40
6103	line*coccidia	2	0	32471.5	32423.05	40
6103	line*coccidia	2	1	143732	32423.05	40
6103	line*coccidia	2	2	53467.67	32423.05	40
6103	line*coccidia	2	3	94215.17	32423.05	40
6104	line	1		142476.5	10816.36	40
6104	line	2		230499.8	10816.36	40
6104	coccidia		0	210304.3	15296.64	40
6104	coccidia		1	196447.8	15296.64	40
6104	coccidia		2	134203.1	15296.64	40
6104	coccidia		3	204997.4	15296.64	40
6104	line*coccidia	1	0	158067	21632.71	40
6104	line*coccidia	1	1	109519.2	21632.71	40
6104	line*coccidia	1	2	68154.17	21632.71	40
6104	line*coccidia	1	3	234165.8	21632.71	40
6104	line*coccidia	2	0	262541.7	21632.71	40
6104	line*coccidia	2	1	283376.5	21632.71	40
6104	line*coccidia	2	2	200252	21632.71	40
6104	line*coccidia	2	3	175829	21632.71	40
6105	line	1		20269.71	9862.02	40
6105	line	2		66912.54	9862.02	40

6105	coccidia		0	15036.42	13947	40
6105	coccidia		1	58124.33	13947	40
6105	coccidia		2	62143.08	13947	40
6105	coccidia		3	39060.67	13947	40
6105	line*coccidia	1	0	15007.33	19724.04	40
6105	line*coccidia	1	1	17901.33	19724.04	40
6105	line*coccidia	1	2	24892.17	19724.04	40
6105	line*coccidia	1	3	23278	19724.04	40
6105	line*coccidia	2	0	15065.5	19724.04	40
6105	line*coccidia	2	1	98347.33	19724.04	40
6105	line*coccidia	2	2	99394	19724.04	40
6105	line*coccidia	2	3	54843.33	19724.04	40
6106	line	1		68371.04	17433.33	40
6106	line	2		45814.58	17433.33	40
6106	coccidia		0	33922.33	24654.46	40
6106	coccidia		1	110403.3	24654.46	40
6106	coccidia		2	20966.25	24654.46	40
6106	coccidia		3	63079.42	24654.46	40
6106	line*coccidia	1	0	31898.17	34866.67	40
6106	line*coccidia	1	1	183665.5	34866.67	40
6106	line*coccidia	1	2	14075	34866.67	40
6106	line*coccidia	1	3	43845.5	34866.67	40
6106	line*coccidia	2	0	35946.5	34866.67	40
6106	line*coccidia	2	1	37141	34866.67	40
6106	line*coccidia	2	2	27857.5	34866.67	40
6106	line*coccidia	2	3	82313.33	34866.67	40
6108	line	1		26068.54	4601.987	40
6108	line	2		38337.46	4601.987	40
6108	coccidia		0	31813.58	6508.193	40
6108	coccidia		1	35839.08	6508.193	40
6108	coccidia		2	17451.25	6508.193	40
6108	coccidia		3	43708.08	6508.193	40
6108	line*coccidia	1	0	28047.83	9203.974	40
6108	line*coccidia	1	1	16781.67	9203.974	40
6108	line*coccidia	1	2	15099.83	9203.974	40
6108	line*coccidia	1	3	44344.83	9203.974	40
6108	line*coccidia	2	0	35579.33	9203.974	40
6108	line*coccidia	2	1	54896.5	9203.974	40
6108	line*coccidia	2	2	19802.67	9203.974	40
6108	line*coccidia	2	3	43071.33	9203.974	40
6109	line	1		36703	9215.546	40
6109	line	2		57002.71	9215.546	40
6109	coccidia		0	40868.58	13032.75	40
6109	coccidia		1	53046.42	13032.75	40
6109	coccidia		2	36849.67	13032.75	40
6109	coccidia		3	56646.75	13032.75	40
6109	line*coccidia	1	0	31685.5	18431.09	40
6109	line*coccidia	1	1	35719.5	18431.09	40
6109	line*coccidia	1	2	23835.67	18431.09	40

6109	line*coccidia	1	3	55571.33	18431.09	40
6109	line*coccidia	2	0	50051.67	18431.09	40
6109	line*coccidia	2	1	70373.33	18431.09	40
6109	line*coccidia	2	2	49863.67	18431.09	40
6109	line*coccidia	2	3	57722.17	18431.09	40
6110	line	1		145997.8	32974.79	40
6110	line	2		72272.13	32974.79	40
6110	coccidia		0	76428.58	46633.4	40
6110	coccidia		1	230647	46633.4	40
6110	coccidia		2	43946.67	46633.4	40
6110	coccidia		3	85517.5	46633.4	40
6110	line*coccidia	1	0	64423.33	65949.59	40
6110	line*coccidia	1	1	375735	65949.59	40
6110	line*coccidia	1	2	43516.83	65949.59	40
6110	line*coccidia	1	3	100315.8	65949.59	40
6110	line*coccidia	2	0	88433.83	65949.59	40
6110	line*coccidia	2	1	85559	65949.59	40
6110	line*coccidia	2	2	44376.5	65949.59	40
6110	line*coccidia	2	3	70719.17	65949.59	40
6111	line	1		354927	21711.29	40
6111	line	2		420403.2	21711.29	40
6111	coccidia		0	393587.3	30704.4	40
6111	coccidia		1	522512.7	30704.4	40
6111	coccidia		2	265976.2	30704.4	40
6111	coccidia		3	368584.3	30704.4	40
6111	line*coccidia	1	0	363669.7	43422.58	40
6111	line*coccidia	1	1	440327.2	43422.58	40
6111	line*coccidia	1	2	172667.3	43422.58	40
6111	line*coccidia	1	3	443043.7	43422.58	40
6111	line*coccidia	2	0	423504.8	43422.58	40
6111	line*coccidia	2	1	604698.2	43422.58	40
6111	line*coccidia	2	2	359285	43422.58	40
6111	line*coccidia	2	3	294124.8	43422.58	40
6113	line	1		401188.3	41855.28	40
6113	line	2		343565.5	41855.28	40
6113	coccidia		0	525935.9	59192.31	40
6113	coccidia		1	269336.1	59192.31	40
6113	coccidia		2	254634.8	59192.31	40
6113	coccidia		3	439600.8	59192.31	40
6113	line*coccidia	1	0	635470	83710.56	40
6113	line*coccidia	1	1	142152.3	83710.56	40
6113	line*coccidia	1	2	225531.8	83710.56	40
6113	line*coccidia	1	3	601598.8	83710.56	40
6113	line*coccidia	2	0	416401.8	83710.56	40
6113	line*coccidia	2	1	396519.8	83710.56	40
6113	line*coccidia	2	2	283737.8	83710.56	40
6113	line*coccidia	2	3	277602.7	83710.56	40
6115	line	1		308992.3	38481.34	40
6115	line	2		323675.3	38481.34	40

6115	coccidia		0	305926.9	54420.83	40
6115	coccidia		1	478236.9	54420.83	40
6115	coccidia		2	159752.4	54420.83	40
6115	coccidia		3	321418.9	54420.83	40
6115	line*coccidia	1	0	357437.8	76962.68	40
6115	line*coccidia	1	1	320975.3	76962.68	40
6115	line*coccidia	1	2	100096.5	76962.68	40
6115	line*coccidia	1	3	457459.7	76962.68	40
6115	line*coccidia	2	0	254416	76962.68	40
6115	line*coccidia	2	1	635498.5	76962.68	40
6115	line*coccidia	2	2	219408.3	76962.68	40
6115	line*coccidia	2	3	185378.2	76962.68	40
6116	line	1		792974.3	125907.7	40
6116	line	2		614350	125907.7	40
6116	coccidia		0	837232	178060.3	40
6116	coccidia		1	458530.1	178060.3	40
6116	coccidia		2	658978.4	178060.3	40
6116	coccidia		3	859908.2	178060.3	40
6116	line*coccidia	1	0	1274093	251815.3	40
6116	line*coccidia	1	1	217120.3	251815.3	40
6116	line*coccidia	1	2	554968	251815.3	40
6116	line*coccidia	1	3	1125717	251815.3	40
6116	line*coccidia	2	0	400371.5	251815.3	40
6116	line*coccidia	2	1	699939.8	251815.3	40
6116	line*coccidia	2	2	762988.8	251815.3	40
6116	line*coccidia	2	3	594099.8	251815.3	40
6117	line	1		117676.8	41362.78	40
6117	line	2		83683.79	41362.78	40
6117	coccidia		0	82660	58495.8	40
6117	coccidia		1	80794	58495.8	40
6117	coccidia		2	38139.42	58495.8	40
6117	coccidia		3	201127.7	58495.8	40
6117	line*coccidia	1	0	105567.2	82725.56	40
6117	line*coccidia	1	1	36780.67	82725.56	40
6117	line*coccidia	1	2	27223.67	82725.56	40
6117	line*coccidia	1	3	301135.5	82725.56	40
6117	line*coccidia	2	0	59752.83	82725.56	40
6117	line*coccidia	2	1	124807.3	82725.56	40
6117	line*coccidia	2	2	49055.17	82725.56	40
6117	line*coccidia	2	3	101119.8	82725.56	40
6118	line	1		328853.4	27133.89	40
6118	line	2		433852.8	27133.89	40
6118	coccidia		0	517912.6	38373.11	40
6118	coccidia		1	351075.3	38373.11	40
6118	coccidia		2	243911.9	38373.11	40
6118	coccidia		3	412512.6	38373.11	40
6118	line*coccidia	1	0	545236.3	54267.78	40
6118	line*coccidia	1	1	157322	54267.78	40
6118	line*coccidia	1	2	138929.5	54267.78	40

6118	line*coccidia	1	3	473925.7	54267.78	40
6118	line*coccidia	2	0	490588.8	54267.78	40
6118	line*coccidia	2	1	544828.5	54267.78	40
6118	line*coccidia	2	2	348894.3	54267.78	40
6118	line*coccidia	2	3	351099.5	54267.78	40
6119	line	1		46344.79	5106.967	40
6119	line	2		65041.13	5106.967	40
6119	coccidia		0	70017.67	7222.342	40
6119	coccidia		1	48941.33	7222.342	40
6119	coccidia		2	46999.17	7222.342	40
6119	coccidia		3	56813.67	7222.342	40
6119	line*coccidia	1	0	56958.33	10213.93	40
6119	line*coccidia	1	1	18980	10213.93	40
6119	line*coccidia	1	2	39082	10213.93	40
6119	line*coccidia	1	3	70358.83	10213.93	40
6119	line*coccidia	2	0	83077	10213.93	40
6119	line*coccidia	2	1	78902.67	10213.93	40
6119	line*coccidia	2	2	54916.33	10213.93	40
6119	line*coccidia	2	3	43268.5	10213.93	40
6120	line	1		917564.4	43920.22	40
6120	line	2		945854.4	43920.22	40
6120	coccidia		0	1104027	62112.57	40
6120	coccidia		1	1020087	62112.57	40
6120	coccidia		2	681082.1	62112.57	40
6120	coccidia		3	921641.8	62112.57	40
6120	line*coccidia	1	0	1164739	87840.43	40
6120	line*coccidia	1	1	816284.5	87840.43	40
6120	line*coccidia	1	2	481288.3	87840.43	40
6120	line*coccidia	1	3	1207946	87840.43	40
6120	line*coccidia	2	0	1043316	87840.43	40
6120	line*coccidia	2	1	1223889	87840.43	40
6120	line*coccidia	2	2	880875.8	87840.43	40
6120	line*coccidia	2	3	635337.3	87840.43	40
6121	line	1		12814.67	2020.936	40
6121	line	2		14429.04	2020.936	40
6121	coccidia		0	20884.25	2858.034	40
6121	coccidia		1	10712	2858.034	40
6121	coccidia		2	6526.167	2858.034	40
6121	coccidia		3	16365	2858.034	40
6121	line*coccidia	1	0	21031.5	4041.871	40
6121	line*coccidia	1	1	3675.167	4041.871	40
6121	line*coccidia	1	2	4471	4041.871	40
6121	line*coccidia	1	3	22081	4041.871	40
6121	line*coccidia	2	0	20737	4041.871	40
6121	line*coccidia	2	1	17748.83	4041.871	40
6121	line*coccidia	2	2	8581.333	4041.871	40
6121	line*coccidia	2	3	10649	4041.871	40
6126	line	1		31979.67	6895.313	40
6126	line	2		48125.96	6895.313	40

6126	coccidia		0	47452.83	9751.445	40
6126	coccidia		1	52557.75	9751.445	40
6126	coccidia		2	23142	9751.445	40
6126	coccidia		3	37058.67	9751.445	40
6126	line*coccidia	1	0	35407.5	13790.63	40
6126	line*coccidia	1	1	37129	13790.63	40
6126	line*coccidia	1	2	17446	13790.63	40
6126	line*coccidia	1	3	37936.17	13790.63	40
6126	line*coccidia	2	0	59498.17	13790.63	40
6126	line*coccidia	2	1	67986.5	13790.63	40
6126	line*coccidia	2	2	28838	13790.63	40
6126	line*coccidia	2	3	36181.17	13790.63	40
6128	line	1		376417	42448.36	40
6128	line	2		507893.3	42448.36	40
6128	coccidia		0	639642.2	60031.05	40
6128	coccidia		1	516874.5	60031.05	40
6128	coccidia		2	243740.9	60031.05	40
6128	coccidia		3	368363.1	60031.05	40
6128	line*coccidia	1	0	686566	84896.73	40
6128	line*coccidia	1	1	146437.8	84896.73	40
6128	line*coccidia	1	2	187845.5	84896.73	40
6128	line*coccidia	1	3	484818.7	84896.73	40
6128	line*coccidia	2	0	592718.3	84896.73	40
6128	line*coccidia	2	1	887311.2	84896.73	40
6128	line*coccidia	2	2	299636.3	84896.73	40
6128	line*coccidia	2	3	251907.5	84896.73	40
6129	line	1		131570	28141.59	40
6129	line	2		62871.88	28141.59	40
6129	coccidia		0	66875.92	39798.22	40
6129	coccidia		1	214976.6	39798.22	40
6129	coccidia		2	40042.92	39798.22	40
6129	coccidia		3	66988.25	39798.22	40
6129	line*coccidia	1	0	59554.67	56283.18	40
6129	line*coccidia	1	1	353497	56283.18	40
6129	line*coccidia	1	2	23046.33	56283.18	40
6129	line*coccidia	1	3	90181.83	56283.18	40
6129	line*coccidia	2	0	74197.17	56283.18	40
6129	line*coccidia	2	1	76456.17	56283.18	40
6129	line*coccidia	2	2	57039.5	56283.18	40
6129	line*coccidia	2	3	43794.67	56283.18	40
6131	line	1		159382.5	38068.36	40
6131	line	2		122023.4	38068.36	40
6131	coccidia		0	54581.33	53836.78	40
6131	coccidia		1	149346.8	53836.78	40
6131	coccidia		2	33845.5	53836.78	40
6131	coccidia		3	325038.1	53836.78	40
6131	line*coccidia	1	0	41872.5	76136.71	40
6131	line*coccidia	1	1	87120.83	76136.71	40
6131	line*coccidia	1	2	25111.83	76136.71	40

6131	line*coccidia	1	3	483424.7	76136.71	40
6131	line*coccidia	2	0	67290.17	76136.71	40
6131	line*coccidia	2	1	211572.7	76136.71	40
6131	line*coccidia	2	2	42579.17	76136.71	40
6131	line*coccidia	2	3	166651.5	76136.71	40
6132	line	1		415306.1	98973.51	40
6132	line	2		165374.8	98973.51	40
6132	coccidia		0	529895.2	139969.7	40
6132	coccidia		1	89382.92	139969.7	40
6132	coccidia		2	123211.6	139969.7	40
6132	coccidia		3	418872	139969.7	40
6132	line*coccidia	1	0	877754.3	197947	40
6132	line*coccidia	1	1	98825.5	197947	40
6132	line*coccidia	1	2	115939.5	197947	40
6132	line*coccidia	1	3	568705	197947	40
6132	line*coccidia	2	0	182036	197947	40
6132	line*coccidia	2	1	79940.33	197947	40
6132	line*coccidia	2	2	130483.7	197947	40
6132	line*coccidia	2	3	269039	197947	40
6201	line	1		101674.1	18448.86	40
6201	line	2		135609.6	18448.86	40
6201	coccidia		0	166564.1	26090.63	40
6201	coccidia		1	100993.3	26090.63	40
6201	coccidia		2	129026.8	26090.63	40
6201	coccidia		3	77983.33	26090.63	40
6201	line*coccidia	1	0	55625	36897.72	40
6201	line*coccidia	1	1	80812	36897.72	40
6201	line*coccidia	1	2	168912.2	36897.72	40
6201	line*coccidia	1	3	101347.2	36897.72	40
6201	line*coccidia	2	0	277503.2	36897.72	40
6201	line*coccidia	2	1	121174.5	36897.72	40
6201	line*coccidia	2	2	89141.33	36897.72	40
6201	line*coccidia	2	3	54619.5	36897.72	40
6202	line	1		25513.5	8821.395	40
6202	line	2		19625.13	8821.395	40
6202	coccidia		0	9295.833	12475.34	40
6202	coccidia		1	39254.92	12475.34	40
6202	coccidia		2	21858.5	12475.34	40
6202	coccidia		3	19868	12475.34	40
6202	line*coccidia	1	0	10494	17642.79	40
6202	line*coccidia	1	1	21137.17	17642.79	40
6202	line*coccidia	1	2	30686.83	17642.79	40
6202	line*coccidia	1	3	39736	17642.79	40
6202	line*coccidia	2	0	8097.667	17642.79	40
6202	line*coccidia	2	1	57372.67	17642.79	40
6202	line*coccidia	2	2	13030.17	17642.79	40
6202	line*coccidia	2	3	0	17642.79	40
6203	line	1		50896.92	11527.25	40
6203	line	2		99099.04	11527.25	40

6203	coccidia		0	52994.08	16301.99	40
6203	coccidia		1	55415.75	16301.99	40
6203	coccidia		2	40454.25	16301.99	40
6203	coccidia		3	151127.8	16301.99	40
6203	line*coccidia	1	0	53719.67	23054.5	40
6203	line*coccidia	1	1	38750.83	23054.5	40
6203	line*coccidia	1	2	28247.83	23054.5	40
6203	line*coccidia	1	3	82869.33	23054.5	40
6203	line*coccidia	2	0	52268.5	23054.5	40
6203	line*coccidia	2	1	72080.67	23054.5	40
6203	line*coccidia	2	2	52660.67	23054.5	40
6203	line*coccidia	2	3	219386.3	23054.5	40
6204	line	1		720125.8	68804.42	40
6204	line	2		814873	68804.42	40
6204	coccidia		0	799645.9	97304.15	40
6204	coccidia		1	822769.9	97304.15	40
6204	coccidia		2	611974	97304.15	40
6204	coccidia		3	835607.9	97304.15	40
6204	line*coccidia	1	0	693363.8	137608.8	40
6204	line*coccidia	1	1	533727.5	137608.8	40
6204	line*coccidia	1	2	551705.3	137608.8	40
6204	line*coccidia	1	3	1101707	137608.8	40
6204	line*coccidia	2	0	905928	137608.8	40
6204	line*coccidia	2	1	1111812	137608.8	40
6204	line*coccidia	2	2	672242.7	137608.8	40
6204	line*coccidia	2	3	569509.2	137608.8	40
6205	line	1		33445.58	49030.94	40
6205	line	2		149206.3	49030.94	40
6205	coccidia		0	63550.25	69340.22	40
6205	coccidia		1	184344.5	69340.22	40
6205	coccidia		2	32790.5	69340.22	40
6205	coccidia		3	84618.58	69340.22	40
6205	line*coccidia	1	0	32779.17	98061.88	40
6205	line*coccidia	1	1	38014	98061.88	40
6205	line*coccidia	1	2	27346.5	98061.88	40
6205	line*coccidia	1	3	35642.67	98061.88	40
6205	line*coccidia	2	0	94321.33	98061.88	40
6205	line*coccidia	2	1	330675	98061.88	40
6205	line*coccidia	2	2	38234.5	98061.88	40
6205	line*coccidia	2	3	133594.5	98061.88	40
6206	line	1		66992.13	72622.48	40
6206	line	2		165995.1	72622.48	40
6206	coccidia		0	57575.33	102703.7	40
6206	coccidia		1	227502.4	102703.7	40
6206	coccidia		2	73901.92	102703.7	40
6206	coccidia		3	106994.8	102703.7	40
6206	line*coccidia	1	0	39958.83	145245	40
6206	line*coccidia	1	1	18419	145245	40
6206	line*coccidia	1	2	75286.5	145245	40

6206	line*coccidia	1	3	134304.2	145245	40
6206	line*coccidia	2	0	75191.83	145245	40
6206	line*coccidia	2	1	436585.8	145245	40
6206	line*coccidia	2	2	72517.33	145245	40
6206	line*coccidia	2	3	79685.5	145245	40
6207	line	1		192520.7	24532.1	40
6207	line	2		151743.2	24532.1	40
6207	coccidia		0	221110.3	34693.62	40
6207	coccidia		1	162169.8	34693.62	40
6207	coccidia		2	95140.67	34693.62	40
6207	coccidia		3	210107.1	34693.62	40
6207	line*coccidia	1	0	265235.7	49064.19	40
6207	line*coccidia	1	1	123997.3	49064.19	40
6207	line*coccidia	1	2	131462	49064.19	40
6207	line*coccidia	1	3	249387.8	49064.19	40
6207	line*coccidia	2	0	176984.8	49064.19	40
6207	line*coccidia	2	1	200342.3	49064.19	40
6207	line*coccidia	2	2	58819.33	49064.19	40
6207	line*coccidia	2	3	170826.3	49064.19	40
6208	line	1		46923.33	31670.27	40
6208	line	2		104982.7	31670.27	40
6208	coccidia		0	58958.5	44788.52	40
6208	coccidia		1	166785.4	44788.52	40
6208	coccidia		2	55269.08	44788.52	40
6208	coccidia		3	22799	44788.52	40
6208	line*coccidia	1	0	34544.67	63340.54	40
6208	line*coccidia	1	1	32314.67	63340.54	40
6208	line*coccidia	1	2	87195.5	63340.54	40
6208	line*coccidia	1	3	33638.5	63340.54	40
6208	line*coccidia	2	0	83372.33	63340.54	40
6208	line*coccidia	2	1	301256.2	63340.54	40
6208	line*coccidia	2	2	23342.67	63340.54	40
6208	line*coccidia	2	3	11959.5	63340.54	40
6209	line	1		40353.38	11550.55	40
6209	line	2		74667.63	11550.55	40
6209	coccidia		0	53294.42	16334.94	40
6209	coccidia		1	73334.25	16334.94	40
6209	coccidia		2	43608.17	16334.94	40
6209	coccidia		3	59805.17	16334.94	40
6209	line*coccidia	1	0	44837.67	23101.09	40
6209	line*coccidia	1	1	12123.5	23101.09	40
6209	line*coccidia	1	2	32047.17	23101.09	40
6209	line*coccidia	1	3	72405.17	23101.09	40
6209	line*coccidia	2	0	61751.17	23101.09	40
6209	line*coccidia	2	1	134545	23101.09	40
6209	line*coccidia	2	2	55169.17	23101.09	40
6209	line*coccidia	2	3	47205.17	23101.09	40
6211	line	1		106492.6	9018.361	40
6211	line	2		126844.4	9018.361	40

6211	coccidia		0	159508.1	12753.89	40
6211	coccidia		1	102614	12753.89	40
6211	coccidia		2	73960.75	12753.89	40
6211	coccidia		3	130591.1	12753.89	40
6211	line*coccidia	1	0	174884.8	18036.72	40
6211	line*coccidia	1	1	32660.83	18036.72	40
6211	line*coccidia	1	2	65189.67	18036.72	40
6211	line*coccidia	1	3	153235	18036.72	40
6211	line*coccidia	2	0	144131.3	18036.72	40
6211	line*coccidia	2	1	172567.2	18036.72	40
6211	line*coccidia	2	2	82731.83	18036.72	40
6211	line*coccidia	2	3	107947.2	18036.72	40
6212	line	1		104523.6	10281.56	40
6212	line	2		82113.21	10281.56	40
6212	coccidia		0	92760.75	14540.32	40
6212	coccidia		1	76807.42	14540.32	40
6212	coccidia		2	83950.67	14540.32	40
6212	coccidia		3	119754.8	14540.32	40
6212	line*coccidia	1	0	120236.2	20563.12	40
6212	line*coccidia	1	1	48792	20563.12	40
6212	line*coccidia	1	2	81580.33	20563.12	40
6212	line*coccidia	1	3	167486	20563.12	40
6212	line*coccidia	2	0	65285.33	20563.12	40
6212	line*coccidia	2	1	104822.8	20563.12	40
6212	line*coccidia	2	2	86321	20563.12	40
6212	line*coccidia	2	3	72023.67	20563.12	40
6213	line	1		39705.13	28773.97	40
6213	line	2		88120.21	28773.97	40
6213	coccidia		0	50009	40692.54	40
6213	coccidia		1	129206.8	40692.54	40
6213	coccidia		2	33741.58	40692.54	40
6213	coccidia		3	42693.25	40692.54	40
6213	line*coccidia	1	0	35704.17	57547.95	40
6213	line*coccidia	1	1	31234.67	57547.95	40
6213	line*coccidia	1	2	36685.17	57547.95	40
6213	line*coccidia	1	3	55196.5	57547.95	40
6213	line*coccidia	2	0	64313.83	57547.95	40
6213	line*coccidia	2	1	227179	57547.95	40
6213	line*coccidia	2	2	30798	57547.95	40
6213	line*coccidia	2	3	30190	57547.95	40
6214	line	1		49889.92	28383.63	40
6214	line	2		94758.13	28383.63	40
6214	coccidia		0	44743	40140.52	40
6214	coccidia		1	54380.33	40140.52	40
6214	coccidia		2	38635.92	40140.52	40
6214	coccidia		3	151536.8	40140.52	40
6214	line*coccidia	1	0	44391.67	56767.26	40
6214	line*coccidia	1	1	16912.17	56767.26	40
6214	line*coccidia	1	2	42161	56767.26	40

6214	line*coccidia	1	3	96094.83	56767.26	40
6214	line*coccidia	2	0	45094.33	56767.26	40
6214	line*coccidia	2	1	91848.5	56767.26	40
6214	line*coccidia	2	2	35110.83	56767.26	40
6214	line*coccidia	2	3	206978.8	56767.26	40
6215	line	1		45420.75	26976.9	40
6215	line	2		130924.6	26976.9	40
6215	coccidia		0	69792.92	38151.09	40
6215	coccidia		1	114920.3	38151.09	40
6215	coccidia		2	27742.42	38151.09	40
6215	coccidia		3	140235.1	38151.09	40
6215	line*coccidia	1	0	75617.5	53953.79	40
6215	line*coccidia	1	1	21234.17	53953.79	40
6215	line*coccidia	1	2	9191.167	53953.79	40
6215	line*coccidia	1	3	75640.17	53953.79	40
6215	line*coccidia	2	0	63968.33	53953.79	40
6215	line*coccidia	2	1	208606.3	53953.79	40
6215	line*coccidia	2	2	46293.67	53953.79	40
6215	line*coccidia	2	3	204830	53953.79	40
6216	line	1		57525.21	22448.31	40
6216	line	2		71649.54	22448.31	40
6216	coccidia		0	39424.67	31746.71	40
6216	coccidia		1	109577.2	31746.71	40
6216	coccidia		2	61083.75	31746.71	40
6216	coccidia		3	48263.92	31746.71	40
6216	line*coccidia	1	0	52460.17	44896.63	40
6216	line*coccidia	1	1	55345.17	44896.63	40
6216	line*coccidia	1	2	84131	44896.63	40
6216	line*coccidia	1	3	38164.5	44896.63	40
6216	line*coccidia	2	0	26389.17	44896.63	40
6216	line*coccidia	2	1	163809.2	44896.63	40
6216	line*coccidia	2	2	38036.5	44896.63	40
6216	line*coccidia	2	3	58363.33	44896.63	40
6218	line	1		150451.5	9851.724	40
6218	line	2		183763.9	9851.724	40
6218	coccidia		0	200075	13932.44	40
6218	coccidia		1	150107.6	13932.44	40
6218	coccidia		2	135834.1	13932.44	40
6218	coccidia		3	182414	13932.44	40
6218	line*coccidia	1	0	181550.2	19703.45	40
6218	line*coccidia	1	1	42930.17	19703.45	40
6218	line*coccidia	1	2	126129.8	19703.45	40
6218	line*coccidia	1	3	251195.7	19703.45	40
6218	line*coccidia	2	0	218599.8	19703.45	40
6218	line*coccidia	2	1	257285	19703.45	40
6218	line*coccidia	2	2	145538.3	19703.45	40
6218	line*coccidia	2	3	113632.3	19703.45	40
6219	line	1		8576.25	2562.434	40
6219	line	2		21893.42	2562.434	40

6219	coccidia		0	15188.83	3623.829	40
6219	coccidia		1	27203.42	3623.829	40
6219	coccidia		2	18547.08	3623.829	40
6219	coccidia		3	9.09E-13	3623.829	40
6219	line*coccidia	1	0	8825.833	5124.869	40
6219	line*coccidia	1	1	-1.4E-12	5124.869	40
6219	line*coccidia	1	2	25479.17	5124.869	40
6219	line*coccidia	1	3	-1.4E-12	5124.869	40
6219	line*coccidia	2	0	21551.83	5124.869	40
6219	line*coccidia	2	1	54406.83	5124.869	40
6219	line*coccidia	2	2	11615	5124.869	40
6219	line*coccidia	2	3	3.18E-12	5124.869	40
6220	line	1		47177.67	23267.13	40
6220	line	2		58677.88	23267.13	40
6220	coccidia		0	72313.75	32904.7	40
6220	coccidia		1	130822.9	32904.7	40
6220	coccidia		2	8574.417	32904.7	40
6220	coccidia		3	1.46E-11	32904.7	40
6220	line*coccidia	1	0	105151.8	46534.27	40
6220	line*coccidia	1	1	83558.83	46534.27	40
6220	line*coccidia	1	2	0	46534.27	40
6220	line*coccidia	1	3	1.09E-11	46534.27	40
6220	line*coccidia	2	0	39475.67	46534.27	40
6220	line*coccidia	2	1	178087	46534.27	40
6220	line*coccidia	2	2	17148.83	46534.27	40
6220	line*coccidia	2	3	1.82E-11	46534.27	40
6222	line	1		3699.333	1684.541	40
6222	line	2		10477.79	1684.541	40
6222	coccidia		0	12659.5	2382.301	40
6222	coccidia		1	0	2382.301	40
6222	coccidia		2	15694.75	2382.301	40
6222	coccidia		3	0	2382.301	40
6222	line*coccidia	1	0	14797.33	3369.082	40
6222	line*coccidia	1	1	0	3369.082	40
6222	line*coccidia	1	2	0	3369.082	40
6222	line*coccidia	1	3	0	3369.082	40
6222	line*coccidia	2	0	10521.67	3369.082	40
6222	line*coccidia	2	1	0	3369.082	40
6222	line*coccidia	2	2	31389.5	3369.082	40
6222	line*coccidia	2	3	0	3369.082	40
6231	line	1		32633.92	11836.29	40
6231	line	2		13142.88	11836.29	40
6231	coccidia		0	20658.58	16739.04	40
6231	coccidia		1	9576.75	16739.04	40
6231	coccidia		2	7109.75	16739.04	40
6231	coccidia		3	54208.5	16739.04	40
6231	line*coccidia	1	0	20989.17	23672.58	40
6231	line*coccidia	1	1	19153.5	23672.58	40
6231	line*coccidia	1	2	0	23672.58	40

6231	line*coccidia	1	3	90393	23672.58	40
6231	line*coccidia	2	0	20328	23672.58	40
6231	line*coccidia	2	1	0	23672.58	40
6231	line*coccidia	2	2	14219.5	23672.58	40
6231	line*coccidia	2	3	18024	23672.58	40
6233	line	1		24554.88	3502.121	40
6233	line	2		12891.63	3502.121	40
6233	coccidia		0	32747.75	4952.747	40
6233	coccidia		1	3827.583	4952.747	40
6233	coccidia		2	22379.25	4952.747	40
6233	coccidia		3	15938.42	4952.747	40
6233	line*coccidia	1	0	30980	7004.243	40
6233	line*coccidia	1	1	7655.167	7004.243	40
6233	line*coccidia	1	2	27707.5	7004.243	40
6233	line*coccidia	1	3	31876.83	7004.243	40
6233	line*coccidia	2	0	34515.5	7004.243	40
6233	line*coccidia	2	1	0	7004.243	40
6233	line*coccidia	2	2	17051	7004.243	40
6233	line*coccidia	2	3	2.73E-12	7004.243	40
6235	line	1		33182.13	6265.035	40
6235	line	2		33909.29	6265.035	40
6235	coccidia		0	33985.33	8860.097	40
6235	coccidia		1	34840.25	8860.097	40
6235	coccidia		2	26708.25	8860.097	40
6235	coccidia		3	38649	8860.097	40
6235	line*coccidia	1	0	38163.33	12530.07	40
6235	line*coccidia	1	1	18543	12530.07	40
6235	line*coccidia	1	2	37095	12530.07	40
6235	line*coccidia	1	3	38927.17	12530.07	40
6235	line*coccidia	2	0	29807.33	12530.07	40
6235	line*coccidia	2	1	51137.5	12530.07	40
6235	line*coccidia	2	2	16321.5	12530.07	40
6235	line*coccidia	2	3	38370.83	12530.07	40
6236	line	1		59029.33	16946.08	40
6236	line	2		71576.54	16946.08	40
6236	coccidia		0	54162.67	23965.38	40
6236	coccidia		1	84897.92	23965.38	40
6236	coccidia		2	39720.67	23965.38	40
6236	coccidia		3	82430.5	23965.38	40
6236	line*coccidia	1	0	37248	33892.16	40
6236	line*coccidia	1	1	-2.5E-11	33892.16	40
6236	line*coccidia	1	2	53487.17	33892.16	40
6236	line*coccidia	1	3	145382.2	33892.16	40
6236	line*coccidia	2	0	71077.33	33892.16	40
6236	line*coccidia	2	1	169795.8	33892.16	40
6236	line*coccidia	2	2	25954.17	33892.16	40
6236	line*coccidia	2	3	19478.83	33892.16	40
6239	line	1		30217.5	30341.51	40
6239	line	2		70930.25	30341.51	40

6239	coccidia		0	40270.83	42909.37	40
6239	coccidia		1	114365.6	42909.37	40
6239	coccidia		2	13448.92	42909.37	40
6239	coccidia		3	34210.17	42909.37	40
6239	line*coccidia	1	0	44271.17	60683.02	40
6239	line*coccidia	1	1	19739.33	60683.02	40
6239	line*coccidia	1	2	19521.67	60683.02	40
6239	line*coccidia	1	3	37337.83	60683.02	40
6239	line*coccidia	2	0	36270.5	60683.02	40
6239	line*coccidia	2	1	208991.8	60683.02	40
6239	line*coccidia	2	2	7376.167	60683.02	40
6239	line*coccidia	2	3	31082.5	60683.02	40
6302	line	1		148969.4	24907.21	40
6302	line	2		97615.83	24907.21	40
6302	coccidia		0	81401.67	35224.12	40
6302	coccidia		1	233655.8	35224.12	40
6302	coccidia		2	68719.08	35224.12	40
6302	coccidia		3	109393.9	35224.12	40
6302	line*coccidia	1	0	90639.67	49814.43	40
6302	line*coccidia	1	1	367017.3	49814.43	40
6302	line*coccidia	1	2	44692.67	49814.43	40
6302	line*coccidia	1	3	93527.83	49814.43	40
6302	line*coccidia	2	0	72163.67	49814.43	40
6302	line*coccidia	2	1	100294.2	49814.43	40
6302	line*coccidia	2	2	92745.5	49814.43	40
6302	line*coccidia	2	3	125260	49814.43	40
6303	line	1		169533.1	33974.03	40
6303	line	2		56906.13	33974.03	40
6303	coccidia		0	27930.83	48046.54	40
6303	coccidia		1	272460.1	48046.54	40
6303	coccidia		2	32531.42	48046.54	40
6303	coccidia		3	119956.1	48046.54	40
6303	line*coccidia	1	0	36755	67948.07	40
6303	line*coccidia	1	1	501835.5	67948.07	40
6303	line*coccidia	1	2	27657.83	67948.07	40
6303	line*coccidia	1	3	111884	67948.07	40
6303	line*coccidia	2	0	19106.67	67948.07	40
6303	line*coccidia	2	1	43084.67	67948.07	40
6303	line*coccidia	2	2	37405	67948.07	40
6303	line*coccidia	2	3	128028.2	67948.07	40
6304	line	1		104779.5	26392.64	40
6304	line	2		166470.4	26392.64	40
6304	coccidia		0	111063.8	37324.83	40
6304	coccidia		1	226434.8	37324.83	40
6304	coccidia		2	77496.92	37324.83	40
6304	coccidia		3	127504.4	37324.83	40
6304	line*coccidia	1	0	128417.7	52785.28	40
6304	line*coccidia	1	1	72612	52785.28	40
6304	line*coccidia	1	2	52398.5	52785.28	40

6304	line*coccidia	1	3	165690	52785.28	40
6304	line*coccidia	2	0	93709.83	52785.28	40
6304	line*coccidia	2	1	380257.7	52785.28	40
6304	line*coccidia	2	2	102595.3	52785.28	40
6304	line*coccidia	2	3	89318.83	52785.28	40
6306	line	1		100067.4	11759.06	40
6306	line	2		133838.7	11759.06	40
6306	coccidia		0	141398.1	16629.82	40
6306	coccidia		1	120980.7	16629.82	40
6306	coccidia		2	94450.08	16629.82	40
6306	coccidia		3	110983.4	16629.82	40
6306	line*coccidia	1	0	139094.5	23518.12	40
6306	line*coccidia	1	1	35972.67	23518.12	40
6306	line*coccidia	1	2	61279.83	23518.12	40
6306	line*coccidia	1	3	163922.7	23518.12	40
6306	line*coccidia	2	0	143701.7	23518.12	40
6306	line*coccidia	2	1	205988.7	23518.12	40
6306	line*coccidia	2	2	127620.3	23518.12	40
6306	line*coccidia	2	3	58044.17	23518.12	40
6307	line	1		310651.3	101192.9	40
6307	line	2		462283.3	101192.9	40
6307	coccidia		0	379717.9	143108.3	40
6307	coccidia		1	580496.8	143108.3	40
6307	coccidia		2	246898.3	143108.3	40
6307	coccidia		3	338756.2	143108.3	40
6307	line*coccidia	1	0	398985.5	202385.7	40
6307	line*coccidia	1	1	133759.5	202385.7	40
6307	line*coccidia	1	2	206203.7	202385.7	40
6307	line*coccidia	1	3	503656.3	202385.7	40
6307	line*coccidia	2	0	360450.3	202385.7	40
6307	line*coccidia	2	1	1027234	202385.7	40
6307	line*coccidia	2	2	287592.8	202385.7	40
6307	line*coccidia	2	3	173856	202385.7	40
6309	line	1		82792.46	13800.54	40
6309	line	2		128740.4	13800.54	40
6309	coccidia		0	127501.8	19516.91	40
6309	coccidia		1	135308.1	19516.91	40
6309	coccidia		2	70465.17	19516.91	40
6309	coccidia		3	89790.67	19516.91	40
6309	line*coccidia	1	0	73410.83	27601.07	40
6309	line*coccidia	1	1	91474.5	27601.07	40
6309	line*coccidia	1	2	68124.17	27601.07	40
6309	line*coccidia	1	3	98160.33	27601.07	40
6309	line*coccidia	2	0	181592.8	27601.07	40
6309	line*coccidia	2	1	179141.7	27601.07	40
6309	line*coccidia	2	2	72806.17	27601.07	40
6309	line*coccidia	2	3	81421	27601.07	40
6310	line	1		209635.7	68250.58	40
6310	line	2		153476.6	68250.58	40

6310	coccidia		0	118405.1	96520.9	40
6310	coccidia		1	483674.8	96520.9	40
6310	coccidia		2	40745.17	96520.9	40
6310	coccidia		3	83399.67	96520.9	40
6310	line*coccidia	1	0	82830.17	136501.2	40
6310	line*coccidia	1	1	585898.8	136501.2	40
6310	line*coccidia	1	2	42402.67	136501.2	40
6310	line*coccidia	1	3	127411.2	136501.2	40
6310	line*coccidia	2	0	153980	136501.2	40
6310	line*coccidia	2	1	381450.7	136501.2	40
6310	line*coccidia	2	2	39087.67	136501.2	40
6310	line*coccidia	2	3	39388.17	136501.2	40
6313	line	1		51975.92	14634.57	40
6313	line	2		68398.83	14634.57	40
6313	coccidia		0	50805.17	20696.4	40
6313	coccidia		1	95278.58	20696.4	40
6313	coccidia		2	42863	20696.4	40
6313	coccidia		3	51802.75	20696.4	40
6313	line*coccidia	1	0	49650.83	29269.13	40
6313	line*coccidia	1	1	35093.67	29269.13	40
6313	line*coccidia	1	2	53358.83	29269.13	40
6313	line*coccidia	1	3	69800.33	29269.13	40
6313	line*coccidia	2	0	51959.5	29269.13	40
6313	line*coccidia	2	1	155463.5	29269.13	40
6313	line*coccidia	2	2	32367.17	29269.13	40
6313	line*coccidia	2	3	33805.17	29269.13	40
6316	line	1		361019	41850.9	40
6316	line	2		291317.7	41850.9	40
6316	coccidia		0	396698.1	59186.11	40
6316	coccidia		1	347692.2	59186.11	40
6316	coccidia		2	170120.3	59186.11	40
6316	coccidia		3	390162.9	59186.11	40
6316	line*coccidia	1	0	474441.7	83701.81	40
6316	line*coccidia	1	1	186600.3	83701.81	40
6316	line*coccidia	1	2	170681.8	83701.81	40
6316	line*coccidia	1	3	612352.3	83701.81	40
6316	line*coccidia	2	0	318954.5	83701.81	40
6316	line*coccidia	2	1	508784	83701.81	40
6316	line*coccidia	2	2	169558.7	83701.81	40
6316	line*coccidia	2	3	167973.5	83701.81	40
6317	line	1		209129	525242.5	40
6317	line	2		928170.2	525242.5	40
6317	coccidia		0	177667.2	742805	40
6317	coccidia		1	1769130	742805	40
6317	coccidia		2	151800.9	742805	40
6317	coccidia		3	176000.3	742805	40
6317	line*coccidia	1	0	175616.8	1050485	40
6317	line*coccidia	1	1	368607.5	1050485	40
6317	line*coccidia	1	2	82091	1050485	40

6317	line*coccidia	1	3	210200.8	1050485	40
6317	line*coccidia	2	0	179717.5	1050485	40
6317	line*coccidia	2	1	3169653	1050485	40
6317	line*coccidia	2	2	221510.8	1050485	40
6317	line*coccidia	2	3	141799.7	1050485	40
6318	line	1		583438.3	43168.08	40
6318	line	2		733768	43168.08	40
6318	coccidia		0	906733.6	61048.88	40
6318	coccidia		1	542775.6	61048.88	40
6318	coccidia		2	527297.3	61048.88	40
6318	coccidia		3	657605.9	61048.88	40
6318	line*coccidia	1	0	819752.3	86336.16	40
6318	line*coccidia	1	1	230814	86336.16	40
6318	line*coccidia	1	2	352774.2	86336.16	40
6318	line*coccidia	1	3	930412.5	86336.16	40
6318	line*coccidia	2	0	993714.8	86336.16	40
6318	line*coccidia	2	1	854737.2	86336.16	40
6318	line*coccidia	2	2	701820.5	86336.16	40
6318	line*coccidia	2	3	384799.3	86336.16	40
6319	line	1		158445.5	356807.5	40
6319	line	2		541096.3	356807.5	40
6319	coccidia		0	42142.92	504601.9	40
6319	coccidia		1	1164480	504601.9	40
6319	coccidia		2	155661.6	504601.9	40
6319	coccidia		3	36798.92	504601.9	40
6319	line*coccidia	1	0	42268.5	713614.9	40
6319	line*coccidia	1	1	265462.3	713614.9	40
6319	line*coccidia	1	2	271013.3	713614.9	40
6319	line*coccidia	1	3	55037.67	713614.9	40
6319	line*coccidia	2	0	42017.33	713614.9	40
6319	line*coccidia	2	1	2063498	713614.9	40
6319	line*coccidia	2	2	40309.83	713614.9	40
6319	line*coccidia	2	3	18560.17	713614.9	40
6321	line	1		17512.54	34269.02	40
6321	line	2		72340.33	34269.02	40
6321	coccidia		0	26805.67	48463.71	40
6321	coccidia		1	113570.4	48463.71	40
6321	coccidia		2	4651.5	48463.71	40
6321	coccidia		3	34678.17	48463.71	40
6321	line*coccidia	1	0	26704.5	68538.04	40
6321	line*coccidia	1	1	13500.17	68538.04	40
6321	line*coccidia	1	2	-1.6E-11	68538.04	40
6321	line*coccidia	1	3	29845.5	68538.04	40
6321	line*coccidia	2	0	26906.83	68538.04	40
6321	line*coccidia	2	1	213640.7	68538.04	40
6321	line*coccidia	2	2	9303	68538.04	40
6321	line*coccidia	2	3	39510.83	68538.04	40
6324	line	1		50456.29	95853.72	40
6324	line	2		181743.8	95853.72	40

6324	coccidia		0	46909.58	135557.6	40
6324	coccidia		1	309356	135557.6	40
6324	coccidia		2	70586.17	135557.6	40
6324	coccidia		3	37548.42	135557.6	40
6324	line*coccidia	1	0	54241.67	191707.4	40
6324	line*coccidia	1	1	30059.83	191707.4	40
6324	line*coccidia	1	2	74895	191707.4	40
6324	line*coccidia	1	3	42628.67	191707.4	40
6324	line*coccidia	2	0	39577.5	191707.4	40
6324	line*coccidia	2	1	588652.2	191707.4	40
6324	line*coccidia	2	2	66277.33	191707.4	40
6324	line*coccidia	2	3	32468.17	191707.4	40
6325	line	1		35452.58	7393.959	40
6325	line	2		25200.75	7393.959	40
6325	coccidia		0	65559.25	10456.64	40
6325	coccidia		1	8.19E-12	10456.64	40
6325	coccidia		2	7622.833	10456.64	40
6325	coccidia		3	48124.58	10456.64	40
6325	line*coccidia	1	0	85984.17	14787.92	40
6325	line*coccidia	1	1	9.09E-12	14787.92	40
6325	line*coccidia	1	2	5.46E-12	14787.92	40
6325	line*coccidia	1	3	55826.17	14787.92	40
6325	line*coccidia	2	0	45134.33	14787.92	40
6325	line*coccidia	2	1	7.28E-12	14787.92	40
6325	line*coccidia	2	2	15245.67	14787.92	40
6325	line*coccidia	2	3	40423	14787.92	40
6337	line	1		64543.21	9833.281	40
6337	line	2		102339.5	9833.281	40
6337	coccidia		0	90079.67	13906.36	40
6337	coccidia		1	95294.25	13906.36	40
6337	coccidia		2	78145.17	13906.36	40
6337	coccidia		3	70246.33	13906.36	40
6337	line*coccidia	1	0	84986.5	19666.56	40
6337	line*coccidia	1	1	25302.83	19666.56	40
6337	line*coccidia	1	2	44151.83	19666.56	40
6337	line*coccidia	1	3	103731.7	19666.56	40
6337	line*coccidia	2	0	95172.83	19666.56	40
6337	line*coccidia	2	1	165285.7	19666.56	40
6337	line*coccidia	2	2	112138.5	19666.56	40
6337	line*coccidia	2	3	36761	19666.56	40
6338	line	1		6968.083	1551.863	40
6338	line	2		7292.583	1551.863	40
6338	coccidia		0	8674.083	2194.665	40
6338	coccidia		1	6794.833	2194.665	40
6338	coccidia		2	5911.083	2194.665	40
6338	coccidia		3	7141.333	2194.665	40
6338	line*coccidia	1	0	4.55E-12	3103.725	40
6338	line*coccidia	1	1	13589.67	3103.725	40
6338	line*coccidia	1	2	9.09E-13	3103.725	40

6338	line*coccidia	1	3	14282.67	3103.725	40
6338	line*coccidia	2	0	17348.17	3103.725	40
6338	line*coccidia	2	1	0	3103.725	40
6338	line*coccidia	2	2	11822.17	3103.725	40
6338	line*coccidia	2	3	-2.7E-12	3103.725	40
6339	line	1		115566.6	40224.42	40
6339	line	2		52674.04	40224.42	40
6339	coccidia		0	30283.33	56885.92	40
6339	coccidia		1	244731.7	56885.92	40
6339	coccidia		2	45185.67	56885.92	40
6339	coccidia		3	16280.58	56885.92	40
6339	line*coccidia	1	0	11687.83	80448.84	40
6339	line*coccidia	1	1	332677.8	80448.84	40
6339	line*coccidia	1	2	85339.5	80448.84	40
6339	line*coccidia	1	3	32561.17	80448.84	40
6339	line*coccidia	2	0	48878.83	80448.84	40
6339	line*coccidia	2	1	156785.5	80448.84	40
6339	line*coccidia	2	2	5031.833	80448.84	40
6339	line*coccidia	2	3	2.91E-11	80448.84	40
6342	line	1		52877.67	8160.989	40
6342	line	2		29786.17	8160.989	40
6342	coccidia		0	26924.92	11541.38	40
6342	coccidia		1	58606.83	11541.38	40
6342	coccidia		2	45875.83	11541.38	40
6342	coccidia		3	33920.08	11541.38	40
6342	line*coccidia	1	0	32728	16321.98	40
6342	line*coccidia	1	1	75263.5	16321.98	40
6342	line*coccidia	1	2	74151.67	16321.98	40
6342	line*coccidia	1	3	29367.5	16321.98	40
6342	line*coccidia	2	0	21121.83	16321.98	40
6342	line*coccidia	2	1	41950.17	16321.98	40
6342	line*coccidia	2	2	17600	16321.98	40
6342	line*coccidia	2	3	38472.67	16321.98	40
6343	line	1		56940.42	145724.3	40
6343	line	2		252233.3	145724.3	40
6343	coccidia		0	46523.83	206085.2	40
6343	coccidia		1	474992.1	206085.2	40
6343	coccidia		2	46535.5	206085.2	40
6343	coccidia		3	50296	206085.2	40
6343	line*coccidia	1	0	30935.33	291448.5	40
6343	line*coccidia	1	1	66849.33	291448.5	40
6343	line*coccidia	1	2	66661.17	291448.5	40
6343	line*coccidia	1	3	63315.83	291448.5	40
6343	line*coccidia	2	0	62112.33	291448.5	40
6343	line*coccidia	2	1	883134.8	291448.5	40
6343	line*coccidia	2	2	26409.83	291448.5	40
6343	line*coccidia	2	3	37276.17	291448.5	40
6347	line	1		93063.79	22045.04	40
6347	line	2		45412	22045.04	40

6347	coccidia		0	52974.92	31176.4	40
6347	coccidia		1	92660.83	31176.4	40
6347	coccidia		2	84687	31176.4	40
6347	coccidia		3	46628.83	31176.4	40
6347	line*coccidia	1	0	52517.83	44090.08	40
6347	line*coccidia	1	1	96957.33	44090.08	40
6347	line*coccidia	1	2	150886.8	44090.08	40
6347	line*coccidia	1	3	71893.17	44090.08	40
6347	line*coccidia	2	0	53432	44090.08	40
6347	line*coccidia	2	1	88364.33	44090.08	40
6347	line*coccidia	2	2	18487.17	44090.08	40
6347	line*coccidia	2	3	21364.5	44090.08	40
6348	line	1		13659.58	3228.009	40
6348	line	2		11092.04	3228.009	40
6348	coccidia		0	23032.33	4565.095	40
6348	coccidia		1	7554.667	4565.095	40
6348	coccidia		2	5494.083	4565.095	40
6348	coccidia		3	13422.17	4565.095	40
6348	line*coccidia	1	0	27794	6456.019	40
6348	line*coccidia	1	1	-3.6E-12	6456.019	40
6348	line*coccidia	1	2	1.82E-12	6456.019	40
6348	line*coccidia	1	3	26844.33	6456.019	40
6348	line*coccidia	2	0	18270.67	6456.019	40
6348	line*coccidia	2	1	15109.33	6456.019	40
6348	line*coccidia	2	2	10988.17	6456.019	40
6348	line*coccidia	2	3	-1.8E-12	6456.019	40
6350	line	1		34662.25	91153.87	40
6350	line	2		201653	91153.87	40
6350	coccidia		0	182551.9	128911	40
6350	coccidia		1	223035	128911	40
6350	coccidia		2	23315.5	128911	40
6350	coccidia		3	43728	128911	40
6350	line*coccidia	1	0	12588.83	182307.7	40
6350	line*coccidia	1	1	4.91E-11	182307.7	40
6350	line*coccidia	1	2	38604.17	182307.7	40
6350	line*coccidia	1	3	87456	182307.7	40
6350	line*coccidia	2	0	352515	182307.7	40
6350	line*coccidia	2	1	446070	182307.7	40
6350	line*coccidia	2	2	8026.833	182307.7	40
6350	line*coccidia	2	3	5.46E-12	182307.7	40
6351	line	1		75215.96	19349.93	40
6351	line	2		80322.21	19349.93	40
6351	coccidia		0	82028.08	27364.94	40
6351	coccidia		1	75833.67	27364.94	40
6351	coccidia		2	80638.5	27364.94	40
6351	coccidia		3	72576.08	27364.94	40
6351	line*coccidia	1	0	74266.67	38699.87	40
6351	line*coccidia	1	1	23046.67	38699.87	40
6351	line*coccidia	1	2	124794	38699.87	40

6351	line*coccidia	1	3	78756.5	38699.87	40
6351	line*coccidia	2	0	89789.5	38699.87	40
6351	line*coccidia	2	1	128620.7	38699.87	40
6351	line*coccidia	2	2	36483	38699.87	40
6351	line*coccidia	2	3	66395.67	38699.87	40
6352	line	1		35973.5	6085.095	40
6352	line	2		42717.63	6085.095	40
6352	coccidia		0	44721.58	8605.624	40
6352	coccidia		1	51274.08	8605.624	40
6352	coccidia		2	21459.58	8605.624	40
6352	coccidia		3	39927	8605.624	40
6352	line*coccidia	1	0	53639.33	12170.19	40
6352	line*coccidia	1	1	24808	12170.19	40
6352	line*coccidia	1	2	17398	12170.19	40
6352	line*coccidia	1	3	48048.67	12170.19	40
6352	line*coccidia	2	0	35803.83	12170.19	40
6352	line*coccidia	2	1	77740.17	12170.19	40
6352	line*coccidia	2	2	25521.17	12170.19	40
6352	line*coccidia	2	3	31805.33	12170.19	40
6355	line	1		26374.29	25489.25	40
6355	line	2		44963.25	25489.25	40
6355	coccidia		0	94871.58	36047.24	40
6355	coccidia		1	-3.6E-12	36047.24	40
6355	coccidia		2	21204.25	36047.24	40
6355	coccidia		3	26599.25	36047.24	40
6355	line*coccidia	1	0	22020.83	50978.5	40
6355	line*coccidia	1	1	-3.6E-12	50978.5	40
6355	line*coccidia	1	2	30277.83	50978.5	40
6355	line*coccidia	1	3	53198.5	50978.5	40
6355	line*coccidia	2	0	167722.3	50978.5	40
6355	line*coccidia	2	1	-3.6E-12	50978.5	40
6355	line*coccidia	2	2	12130.67	50978.5	40
6355	line*coccidia	2	3	2E-11	50978.5	40
6356	line	1		76062.38	10996.72	40
6356	line	2		74066.88	10996.72	40
6356	coccidia		0	66245.83	15551.71	40
6356	coccidia		1	60104.25	15551.71	40
6356	coccidia		2	114585.3	15551.71	40
6356	coccidia		3	59323.08	15551.71	40
6356	line*coccidia	1	0	58006.33	21993.44	40
6356	line*coccidia	1	1	7.28E-12	21993.44	40
6356	line*coccidia	1	2	159805.5	21993.44	40
6356	line*coccidia	1	3	86437.67	21993.44	40
6356	line*coccidia	2	0	74485.33	21993.44	40
6356	line*coccidia	2	1	120208.5	21993.44	40
6356	line*coccidia	2	2	69365.17	21993.44	40
6356	line*coccidia	2	3	32208.5	21993.44	40
6403	line	1		61669.13	15598.76	40
6403	line	2		52070.79	15598.76	40

6403	coccidia		0	38383.58	22059.97	40
6403	coccidia		1	100373.9	22059.97	40
6403	coccidia		2	50235.17	22059.97	40
6403	coccidia		3	38487.17	22059.97	40
6403	line*coccidia	1	0	14088.83	31197.51	40
6403	line*coccidia	1	1	79542.33	31197.51	40
6403	line*coccidia	1	2	76071	31197.51	40
6403	line*coccidia	1	3	76974.33	31197.51	40
6403	line*coccidia	2	0	62678.33	31197.51	40
6403	line*coccidia	2	1	121205.5	31197.51	40
6403	line*coccidia	2	2	24399.33	31197.51	40
6403	line*coccidia	2	3	1.82E-11	31197.51	40
6404	line	1		4639042	434148.6	40
6404	line	2		5948956	434148.6	40
6404	coccidia		0	6808945	613978.8	40
6404	coccidia		1	3992187	613978.8	40
6404	coccidia		2	4705372	613978.8	40
6404	coccidia		3	5669494	613978.8	40
6404	line*coccidia	1	0	5748583	868297.2	40
6404	line*coccidia	1	1	3371357	868297.2	40
6404	line*coccidia	1	2	3183162	868297.2	40
6404	line*coccidia	1	3	6253068	868297.2	40
6404	line*coccidia	2	0	7869307	868297.2	40
6404	line*coccidia	2	1	4613016	868297.2	40
6404	line*coccidia	2	2	6227583	868297.2	40
6404	line*coccidia	2	3	5085920	868297.2	40
6406	line	1		4808593	565021.6	40
6406	line	2		5596551	565021.6	40
6406	coccidia		0	6575008	799061.2	40
6406	coccidia		1	4766226	799061.2	40
6406	coccidia		2	3466971	799061.2	40
6406	coccidia		3	6002083	799061.2	40
6406	line*coccidia	1	0	7012442	1130043	40
6406	line*coccidia	1	1	1564140	1130043	40
6406	line*coccidia	1	2	2702533	1130043	40
6406	line*coccidia	1	3	7955258	1130043	40
6406	line*coccidia	2	0	6137574	1130043	40
6406	line*coccidia	2	1	7968313	1130043	40
6406	line*coccidia	2	2	4231409	1130043	40
6406	line*coccidia	2	3	4048909	1130043	40
6408	line	1		15292821	840567.5	40
6408	line	2		12121794	840567.5	40
6408	coccidia		0	19698392	1188742	40
6408	coccidia		1	8717514	1188742	40
6408	coccidia		2	9863447	1188742	40
6408	coccidia		3	16549876	1188742	40
6408	line*coccidia	1	0	20435988	1681135	40
6408	line*coccidia	1	1	5890170	1681135	40
6408	line*coccidia	1	2	11058739	1681135	40

6408	line*coccidia	1	3	23786387	1681135	40
6408	line*coccidia	2	0	18960796	1681135	40
6408	line*coccidia	2	1	11544859	1681135	40
6408	line*coccidia	2	2	8668155	1681135	40
6408	line*coccidia	2	3	9313365	1681135	40
6411	line	1		7603283	509280.7	40
6411	line	2		6716806	509280.7	40
6411	coccidia		0	10254890	720231.7	40
6411	coccidia		1	4405014	720231.7	40
6411	coccidia		2	4537317	720231.7	40
6411	coccidia		3	9442958	720231.7	40
6411	line*coccidia	1	0	11413626	1018561	40
6411	line*coccidia	1	1	1087270	1018561	40
6411	line*coccidia	1	2	4633845	1018561	40
6411	line*coccidia	1	3	13278389	1018561	40
6411	line*coccidia	2	0	9096154	1018561	40
6411	line*coccidia	2	1	7722758	1018561	40
6411	line*coccidia	2	2	4440788	1018561	40
6411	line*coccidia	2	3	5607526	1018561	40
6414	line	1		103699.6	28330.42	40
6414	line	2		76459.5	28330.42	40
6414	coccidia		0	24110.33	40065.27	40
6414	coccidia		1	158701.2	40065.27	40
6414	coccidia		2	97899.17	40065.27	40
6414	coccidia		3	79607.5	40065.27	40
6414	line*coccidia	1	0	25553	56660.85	40
6414	line*coccidia	1	1	190648	56660.85	40
6414	line*coccidia	1	2	179561.7	56660.85	40
6414	line*coccidia	1	3	19035.67	56660.85	40
6414	line*coccidia	2	0	22667.67	56660.85	40
6414	line*coccidia	2	1	126754.3	56660.85	40
6414	line*coccidia	2	2	16236.67	56660.85	40
6414	line*coccidia	2	3	140179.3	56660.85	40
6415	line	1		1291340	259348	40
6415	line	2		1754044	259348	40
6415	coccidia		0	2202642	366773.4	40
6415	coccidia		1	1376272	366773.4	40
6415	coccidia		2	944109.8	366773.4	40
6415	coccidia		3	1567744	366773.4	40
6415	line*coccidia	1	0	2102611	518696	40
6415	line*coccidia	1	1	312679	518696	40
6415	line*coccidia	1	2	537417.3	518696	40
6415	line*coccidia	1	3	2212652	518696	40
6415	line*coccidia	2	0	2302672	518696	40
6415	line*coccidia	2	1	2439865	518696	40
6415	line*coccidia	2	2	1350802	518696	40
6415	line*coccidia	2	3	922835	518696	40
6427	line	1		1189629	268442.1	40
6427	line	2		1047181	268442.1	40

6427	coccidia		0	1479508	379634.5	40
6427	coccidia		1	581011.7	379634.5	40
6427	coccidia		2	690373.2	379634.5	40
6427	coccidia		3	1722727	379634.5	40
6427	line*coccidia	1	0	1406912	536884.3	40
6427	line*coccidia	1	1	193130.7	536884.3	40
6427	line*coccidia	1	2	564292	536884.3	40
6427	line*coccidia	1	3	2594181	536884.3	40
6427	line*coccidia	2	0	1552105	536884.3	40
6427	line*coccidia	2	1	968892.7	536884.3	40
6427	line*coccidia	2	2	816454.3	536884.3	40
6427	line*coccidia	2	3	851273	536884.3	40
6430	line	1		1450255	444335.7	40
6430	line	2		1898457	444335.7	40
6430	coccidia		0	2332475	628385.6	40
6430	coccidia		1	1980996	628385.6	40
6430	coccidia		2	629228.3	628385.6	40
6430	coccidia		3	1754726	628385.6	40
6430	line*coccidia	1	0	2714638	888671.5	40
6430	line*coccidia	1	1	215649.7	888671.5	40
6430	line*coccidia	1	2	378005.2	888671.5	40
6430	line*coccidia	1	3	2492729	888671.5	40
6430	line*coccidia	2	0	1950312	888671.5	40
6430	line*coccidia	2	1	3746343	888671.5	40
6430	line*coccidia	2	2	880451.3	888671.5	40
6430	line*coccidia	2	3	1016723	888671.5	40
6431	line	1		261680.2	45012.57	40
6431	line	2		161813	45012.57	40
6431	coccidia		0	439295.7	63657.39	40
6431	coccidia		1	282259.8	63657.39	40
6431	coccidia		2	125430.9	63657.39	40
6431	coccidia		3	6.55E-11	63657.39	40
6431	line*coccidia	1	0	710751.3	90025.15	40
6431	line*coccidia	1	1	158807.8	90025.15	40
6431	line*coccidia	1	2	177161.5	90025.15	40
6431	line*coccidia	1	3	4.37E-11	90025.15	40
6431	line*coccidia	2	0	167840	90025.15	40
6431	line*coccidia	2	1	405711.7	90025.15	40
6431	line*coccidia	2	2	73700.33	90025.15	40
6431	line*coccidia	2	3	8.73E-11	90025.15	40
6432	line	1		367063.3	84397.5	40
6432	line	2		443297.5	84397.5	40
6432	coccidia		0	471786.2	119356.1	40
6432	coccidia		1	421483.7	119356.1	40
6432	coccidia		2	250296.9	119356.1	40
6432	coccidia		3	477154.8	119356.1	40
6432	line*coccidia	1	0	479668.2	168795	40
6432	line*coccidia	1	1	360006	168795	40
6432	line*coccidia	1	2	365605.7	168795	40

6432	line*coccidia	1	3	262973.5	168795	40
6432	line*coccidia	2	0	463904.2	168795	40
6432	line*coccidia	2	1	482961.3	168795	40
6432	line*coccidia	2	2	134988.2	168795	40
6432	line*coccidia	2	3	691336.2	168795	40
6434	line	1		81987.79	28496.25	40
6434	line	2		169447.6	28496.25	40
6434	coccidia		0	248982.8	40299.78	40
6434	coccidia		1	200066.6	40299.78	40
6434	coccidia		2	53821.42	40299.78	40
6434	coccidia		3	1.46E-11	40299.78	40
6434	line*coccidia	1	0	277672	56992.5	40
6434	line*coccidia	1	1	50279.17	56992.5	40
6434	line*coccidia	1	2	7.28E-12	56992.5	40
6434	line*coccidia	1	3	7.28E-12	56992.5	40
6434	line*coccidia	2	0	220293.7	56992.5	40
6434	line*coccidia	2	1	349854	56992.5	40
6434	line*coccidia	2	2	107642.8	56992.5	40
6434	line*coccidia	2	3	2.18E-11	56992.5	40
6501	line	1		630054.3	179852.9	40
6501	line	2		642775	179852.9	40
6501	coccidia		0	802107.7	254350.3	40
6501	coccidia		1	613889.8	254350.3	40
6501	coccidia		2	329370.8	254350.3	40
6501	coccidia		3	800290.3	254350.3	40
6501	line*coccidia	1	0	585991.7	359705.7	40
6501	line*coccidia	1	1	382611	359705.7	40
6501	line*coccidia	1	2	129688.5	359705.7	40
6501	line*coccidia	1	3	1421926	359705.7	40
6501	line*coccidia	2	0	1018224	359705.7	40
6501	line*coccidia	2	1	845168.5	359705.7	40
6501	line*coccidia	2	2	529053	359705.7	40
6501	line*coccidia	2	3	178654.8	359705.7	40
6502	line	1		625246	115681.9	40
6502	line	2		687432.1	115681.9	40
6502	coccidia		0	834095.8	163598.9	40
6502	coccidia		1	525984.3	163598.9	40
6502	coccidia		2	529615.8	163598.9	40
6502	coccidia		3	735660.3	163598.9	40
6502	line*coccidia	1	0	741649.5	231363.8	40
6502	line*coccidia	1	1	614358	231363.8	40
6502	line*coccidia	1	2	125835.5	231363.8	40
6502	line*coccidia	1	3	1019141	231363.8	40
6502	line*coccidia	2	0	926542.2	231363.8	40
6502	line*coccidia	2	1	437610.7	231363.8	40
6502	line*coccidia	2	2	933396	231363.8	40
6502	line*coccidia	2	3	452179.5	231363.8	40
6503	line	1		487886.4	94486.07	40
6503	line	2		424115.3	94486.07	40

6503	coccidia		0	320577	133623.5	40
6503	coccidia		1	671060.9	133623.5	40
6503	coccidia		2	475271.6	133623.5	40
6503	coccidia		3	357093.8	133623.5	40
6503	line*coccidia	1	0	316859.8	188972.1	40
6503	line*coccidia	1	1	859372.7	188972.1	40
6503	line*coccidia	1	2	280175.8	188972.1	40
6503	line*coccidia	1	3	495137.3	188972.1	40
6503	line*coccidia	2	0	324294.2	188972.1	40
6503	line*coccidia	2	1	482749.2	188972.1	40
6503	line*coccidia	2	2	670367.3	188972.1	40
6503	line*coccidia	2	3	219050.3	188972.1	40
6504	line	1		216475	45339.14	40
6504	line	2		374333.7	45339.14	40
6504	coccidia		0	151309.3	64119.22	40
6504	coccidia		1	646233.1	64119.22	40
6504	coccidia		2	166271.5	64119.22	40
6504	coccidia		3	217803.5	64119.22	40
6504	line*coccidia	1	0	77084.17	90678.27	40
6504	line*coccidia	1	1	321296.3	90678.27	40
6504	line*coccidia	1	2	123448	90678.27	40
6504	line*coccidia	1	3	344071.7	90678.27	40
6504	line*coccidia	2	0	225534.5	90678.27	40
6504	line*coccidia	2	1	971169.8	90678.27	40
6504	line*coccidia	2	2	209095	90678.27	40
6504	line*coccidia	2	3	91535.33	90678.27	40
6505	line	1		9442463	1903094	40
6505	line	2		8688619	1903094	40
6505	coccidia		0	13820258	2691382	40
6505	coccidia		1	4692223	2691382	40
6505	coccidia		2	8104035	2691382	40
6505	coccidia		3	9645650	2691382	40
6505	line*coccidia	1	0	14009375	3806189	40
6505	line*coccidia	1	1	7120652	3806189	40
6505	line*coccidia	1	2	4729016	3806189	40
6505	line*coccidia	1	3	11910809	3806189	40
6505	line*coccidia	2	0	13631140	3806189	40
6505	line*coccidia	2	1	2263793	3806189	40
6505	line*coccidia	2	2	11479054	3806189	40
6505	line*coccidia	2	3	7380490	3806189	40
6506	line	1		1964989	218176.2	40
6506	line	2		1353579	218176.2	40
6506	coccidia		0	2749572	308547.7	40
6506	coccidia		1	1212299	308547.7	40
6506	coccidia		2	670416.3	308547.7	40
6506	coccidia		3	2004850	308547.7	40
6506	line*coccidia	1	0	3304394	436352.3	40
6506	line*coccidia	1	1	648216.3	436352.3	40
6506	line*coccidia	1	2	420644.5	436352.3	40

6506	line*coccidia	1	3	3486702	436352.3	40
6506	line*coccidia	2	0	2194749	436352.3	40
6506	line*coccidia	2	1	1776383	436352.3	40
6506	line*coccidia	2	2	920188	436352.3	40
6506	line*coccidia	2	3	522998	436352.3	40
6507	line	1		799629.8	91054.23	40
6507	line	2		589385.1	91054.23	40
6507	coccidia		0	724753.7	128770.1	40
6507	coccidia		1	935455.7	128770.1	40
6507	coccidia		2	460385.7	128770.1	40
6507	coccidia		3	657434.8	128770.1	40
6507	line*coccidia	1	0	584844.3	182108.5	40
6507	line*coccidia	1	1	1239237	182108.5	40
6507	line*coccidia	1	2	441804.3	182108.5	40
6507	line*coccidia	1	3	932633.3	182108.5	40
6507	line*coccidia	2	0	864663	182108.5	40
6507	line*coccidia	2	1	631674.2	182108.5	40
6507	line*coccidia	2	2	478967	182108.5	40
6507	line*coccidia	2	3	382236.3	182108.5	40
6508	line	1		1195534	300591.2	40
6508	line	2		1211177	300591.2	40
6508	coccidia		0	1508442	425100.1	40
6508	coccidia		1	1639789	425100.1	40
6508	coccidia		2	453682.4	425100.1	40
6508	coccidia		3	1211509	425100.1	40
6508	line*coccidia	1	0	1826301	601182.4	40
6508	line*coccidia	1	1	1101666	601182.4	40
6508	line*coccidia	1	2	308334.3	601182.4	40
6508	line*coccidia	1	3	1545836	601182.4	40
6508	line*coccidia	2	0	1190582	601182.4	40
6508	line*coccidia	2	1	2177913	601182.4	40
6508	line*coccidia	2	2	599030.5	601182.4	40
6508	line*coccidia	2	3	877182.3	601182.4	40
6509	line	1		864906.5	173030	40
6509	line	2		971178.2	173030	40
6509	coccidia		0	1667222	244701.4	40
6509	coccidia		1	829088.6	244701.4	40
6509	coccidia		2	252825	244701.4	40
6509	coccidia		3	923034.1	244701.4	40
6509	line*coccidia	1	0	1463873	346060	40
6509	line*coccidia	1	1	226184.8	346060	40
6509	line*coccidia	1	2	293168	346060	40
6509	line*coccidia	1	3	1476400	346060	40
6509	line*coccidia	2	0	1870570	346060	40
6509	line*coccidia	2	1	1431992	346060	40
6509	line*coccidia	2	2	212482	346060	40
6509	line*coccidia	2	3	369668.2	346060	40
6511	line	1		1125048	172524.6	40
6511	line	2		1054972	172524.6	40

6511	coccidia		0	1844316	243986.6	40
6511	coccidia		1	659382.8	243986.6	40
6511	coccidia		2	545614.3	243986.6	40
6511	coccidia		3	1310727	243986.6	40
6511	line*coccidia	1	0	1941328	345049.2	40
6511	line*coccidia	1	1	292540.5	345049.2	40
6511	line*coccidia	1	2	185414.2	345049.2	40
6511	line*coccidia	1	3	2080908	345049.2	40
6511	line*coccidia	2	0	1747303	345049.2	40
6511	line*coccidia	2	1	1026225	345049.2	40
6511	line*coccidia	2	2	905814.3	345049.2	40
6511	line*coccidia	2	3	540546.2	345049.2	40
6512	line	1		1188008	154688.3	40
6512	line	2		919717.1	154688.3	40
6512	coccidia		0	798212.1	218762.2	40
6512	coccidia		1	1626592	218762.2	40
6512	coccidia		2	922701.4	218762.2	40
6512	coccidia		3	867945.3	218762.2	40
6512	line*coccidia	1	0	780524	309376.5	40
6512	line*coccidia	1	1	1480719	309376.5	40
6512	line*coccidia	1	2	1147216	309376.5	40
6512	line*coccidia	1	3	1343575	309376.5	40
6512	line*coccidia	2	0	815900.2	309376.5	40
6512	line*coccidia	2	1	1772465	309376.5	40
6512	line*coccidia	2	2	698186.8	309376.5	40
6512	line*coccidia	2	3	392316	309376.5	40
6516	line	1		417313.2	87470.73	40
6516	line	2		473279.9	87470.73	40
6516	coccidia		0	645362	123702.3	40
6516	coccidia		1	334547.4	123702.3	40
6516	coccidia		2	274410.3	123702.3	40
6516	coccidia		3	526866.4	123702.3	40
6516	line*coccidia	1	0	507933	174941.5	40
6516	line*coccidia	1	1	213544.5	174941.5	40
6516	line*coccidia	1	2	132145.2	174941.5	40
6516	line*coccidia	1	3	815630	174941.5	40
6516	line*coccidia	2	0	782791	174941.5	40
6516	line*coccidia	2	1	455550.3	174941.5	40
6516	line*coccidia	2	2	416675.5	174941.5	40
6516	line*coccidia	2	3	238102.8	174941.5	40
6517	line	1		435769.6	80564.18	40
6517	line	2		100740	80564.18	40
6517	coccidia		0	375367.2	113935	40
6517	coccidia		1	227436.7	113935	40
6517	coccidia		2	83049.33	113935	40
6517	coccidia		3	387165.9	113935	40
6517	line*coccidia	1	0	689016.7	161128.4	40
6517	line*coccidia	1	1	131954.5	161128.4	40
6517	line*coccidia	1	2	147775.3	161128.4	40

6517	line*coccidia	1	3	774331.8	161128.4	40
6517	line*coccidia	2	0	61717.67	161128.4	40
6517	line*coccidia	2	1	322918.8	161128.4	40
6517	line*coccidia	2	2	18323.33	161128.4	40
6517	line*coccidia	2	3	-1.2E-10	161128.4	40
6518	line	1		2475638	236575.9	40
6518	line	2		2019559	236575.9	40
6518	coccidia		0	3959240	334568.9	40
6518	coccidia		1	1380836	334568.9	40
6518	coccidia		2	1077734	334568.9	40
6518	coccidia		3	2572583	334568.9	40
6518	line*coccidia	1	0	4083197	473151.8	40
6518	line*coccidia	1	1	579927	473151.8	40
6518	line*coccidia	1	2	755267.5	473151.8	40
6518	line*coccidia	1	3	4484159	473151.8	40
6518	line*coccidia	2	0	3835283	473151.8	40
6518	line*coccidia	2	1	2181745	473151.8	40
6518	line*coccidia	2	2	1400201	473151.8	40
6518	line*coccidia	2	3	661006.5	473151.8	40
6519	line	1		4205184	464491.5	40
6519	line	2		3174196	464491.5	40
6519	coccidia		0	3395754	656890.1	40
6519	coccidia		1	4660106	656890.1	40
6519	coccidia		2	3429107	656890.1	40
6519	coccidia		3	3273793	656890.1	40
6519	line*coccidia	1	0	3058948	928982.9	40
6519	line*coccidia	1	1	5389011	928982.9	40
6519	line*coccidia	1	2	4173025	928982.9	40
6519	line*coccidia	1	3	4199752	928982.9	40
6519	line*coccidia	2	0	3732561	928982.9	40
6519	line*coccidia	2	1	3931201	928982.9	40
6519	line*coccidia	2	2	2685189	928982.9	40
6519	line*coccidia	2	3	2347834	928982.9	40
6521	line	1		487320.2	99394.88	40
6521	line	2		476153.6	99394.88	40
6521	coccidia		0	578734.8	140565.6	40
6521	coccidia		1	178389.5	140565.6	40
6521	coccidia		2	270608.8	140565.6	40
6521	coccidia		3	899214.5	140565.6	40
6521	line*coccidia	1	0	443748.2	198789.8	40
6521	line*coccidia	1	1	82048.5	198789.8	40
6521	line*coccidia	1	2	60764.83	198789.8	40
6521	line*coccidia	1	3	1362719	198789.8	40
6521	line*coccidia	2	0	713721.5	198789.8	40
6521	line*coccidia	2	1	274730.5	198789.8	40
6521	line*coccidia	2	2	480452.8	198789.8	40
6521	line*coccidia	2	3	435709.7	198789.8	40
6522	line	1		1438733	205639.5	40
6522	line	2		1377279	205639.5	40

6522	coccidia		0	1901599	290818.1	40
6522	coccidia		1	1454794	290818.1	40
6522	coccidia		2	825146.6	290818.1	40
6522	coccidia		3	1450486	290818.1	40
6522	line*coccidia	1	0	2122819	411278.9	40
6522	line*coccidia	1	1	1018193	411278.9	40
6522	line*coccidia	1	2	274242.3	411278.9	40
6522	line*coccidia	1	3	2339679	411278.9	40
6522	line*coccidia	2	0	1680380	411278.9	40
6522	line*coccidia	2	1	1891395	411278.9	40
6522	line*coccidia	2	2	1376051	411278.9	40
6522	line*coccidia	2	3	561292.2	411278.9	40
6524	line	1		138841.8	46376.85	40
6524	line	2		117315.3	46376.85	40
6524	coccidia		0	272007.3	65586.77	40
6524	coccidia		1	210615.8	65586.77	40
6524	coccidia		2	29691.17	65586.77	40
6524	coccidia		3	2.91E-11	65586.77	40
6524	line*coccidia	1	0	364572.2	92753.69	40
6524	line*coccidia	1	1	190795.2	92753.69	40
6524	line*coccidia	1	2	3.64E-12	92753.69	40
6524	line*coccidia	1	3	2.55E-11	92753.69	40
6524	line*coccidia	2	0	179442.3	92753.69	40
6524	line*coccidia	2	1	230436.3	92753.69	40
6524	line*coccidia	2	2	59382.33	92753.69	40
6524	line*coccidia	2	3	3.27E-11	92753.69	40
6526	line	1		718600.3	115370.1	40
6526	line	2		628277.1	115370.1	40
6526	coccidia		0	1125115	163157.9	40
6526	coccidia		1	313509	163157.9	40
6526	coccidia		2	332244.6	163157.9	40
6526	coccidia		3	922886.1	163157.9	40
6526	line*coccidia	1	0	1478365	230740.1	40
6526	line*coccidia	1	1	142049.5	230740.1	40
6526	line*coccidia	1	2	79187	230740.1	40
6526	line*coccidia	1	3	1174800	230740.1	40
6526	line*coccidia	2	0	771865.2	230740.1	40
6526	line*coccidia	2	1	484968.5	230740.1	40
6526	line*coccidia	2	2	585302.2	230740.1	40
6526	line*coccidia	2	3	670972.7	230740.1	40
6527	line	1		13312888	1258095	40
6527	line	2		9636521	1258095	40
6527	coccidia		0	12494604	1779215	40
6527	coccidia		1	10564646	1779215	40
6527	coccidia		2	10112451	1779215	40
6527	coccidia		3	12727117	1779215	40
6527	line*coccidia	1	0	13420787	2516189	40
6527	line*coccidia	1	1	13628198	2516189	40
6527	line*coccidia	1	2	12115384	2516189	40

6527	line*coccidia	1	3	14087183	2516189	40
6527	line*coccidia	2	0	11568420	2516189	40
6527	line*coccidia	2	1	7501095	2516189	40
6527	line*coccidia	2	2	8109517	2516189	40
6527	line*coccidia	2	3	11367052	2516189	40
6529	line	1		2609789	346074.3	40
6529	line	2		2482924	346074.3	40
6529	coccidia		0	4561887	489423	40
6529	coccidia		1	1504687	489423	40
6529	coccidia		2	1629446	489423	40
6529	coccidia		3	2489407	489423	40
6529	line*coccidia	1	0	5050847	692148.6	40
6529	line*coccidia	1	1	389080.2	692148.6	40
6529	line*coccidia	1	2	1283769	692148.6	40
6529	line*coccidia	1	3	3715460	692148.6	40
6529	line*coccidia	2	0	4072927	692148.6	40
6529	line*coccidia	2	1	2620294	692148.6	40
6529	line*coccidia	2	2	1975122	692148.6	40
6529	line*coccidia	2	3	1263353	692148.6	40
6531	line	1		1606146	412133.6	40
6531	line	2		1489435	412133.6	40
6531	coccidia		0	1148516	582844.9	40
6531	coccidia		1	2718474	582844.9	40
6531	coccidia		2	1079285	582844.9	40
6531	coccidia		3	1244887	582844.9	40
6531	line*coccidia	1	0	1013258	824267.1	40
6531	line*coccidia	1	1	2296888	824267.1	40
6531	line*coccidia	1	2	1297500	824267.1	40
6531	line*coccidia	1	3	1816938	824267.1	40
6531	line*coccidia	2	0	1283775	824267.1	40
6531	line*coccidia	2	1	3140059	824267.1	40
6531	line*coccidia	2	2	861069.5	824267.1	40
6531	line*coccidia	2	3	672836	824267.1	40
6532	line	1		17792643	2061154	40
6532	line	2		16231473	2061154	40
6532	coccidia		0	20993786	2914912	40
6532	coccidia		1	16431972	2914912	40
6532	coccidia		2	16222584	2914912	40
6532	coccidia		3	14399889	2914912	40
6532	line*coccidia	1	0	25807797	4122308	40
6532	line*coccidia	1	1	16607720	4122308	40
6532	line*coccidia	1	2	12954455	4122308	40
6532	line*coccidia	1	3	15800600	4122308	40
6532	line*coccidia	2	0	16179776	4122308	40
6532	line*coccidia	2	1	16256224	4122308	40
6532	line*coccidia	2	2	19490713	4122308	40
6532	line*coccidia	2	3	12999179	4122308	40
6533	line	1		2049321	534380.4	40
6533	line	2		1598971	534380.4	40

6533	coccidia		0	2544590	755728	40
6533	coccidia		1	2062029	755728	40
6533	coccidia		2	614464	755728	40
6533	coccidia		3	2075502	755728	40
6533	line*coccidia	1	0	1606283	1068761	40
6533	line*coccidia	1	1	2485699	1068761	40
6533	line*coccidia	1	2	700509.3	1068761	40
6533	line*coccidia	1	3	3404792	1068761	40
6533	line*coccidia	2	0	3482896	1068761	40
6533	line*coccidia	2	1	1638360	1068761	40
6533	line*coccidia	2	2	528418.7	1068761	40
6533	line*coccidia	2	3	746210.7	1068761	40
6534	line	1		6445976	1225549	40
6534	line	2		5458373	1225549	40
6534	coccidia		0	7450134	1733189	40
6534	coccidia		1	3936630	1733189	40
6534	coccidia		2	5424083	1733189	40
6534	coccidia		3	6997852	1733189	40
6534	line*coccidia	1	0	9761319	2451099	40
6534	line*coccidia	1	1	4090903	2451099	40
6534	line*coccidia	1	2	3650350	2451099	40
6534	line*coccidia	1	3	8281331	2451099	40
6534	line*coccidia	2	0	5138948	2451099	40
6534	line*coccidia	2	1	3782357	2451099	40
6534	line*coccidia	2	2	7197815	2451099	40
6534	line*coccidia	2	3	5714373	2451099	40
6535	line	1		427451.1	130248.9	40
6535	line	2		333919.1	130248.9	40
6535	coccidia		0	169239	184199.8	40
6535	coccidia		1	1070576	184199.8	40
6535	coccidia		2	60289.33	184199.8	40
6535	coccidia		3	222635.8	184199.8	40
6535	line*coccidia	1	0	249426	260497.8	40
6535	line*coccidia	1	1	1127910	260497.8	40
6535	line*coccidia	1	2	34611	260497.8	40
6535	line*coccidia	1	3	297857.3	260497.8	40
6535	line*coccidia	2	0	89052	260497.8	40
6535	line*coccidia	2	1	1013243	260497.8	40
6535	line*coccidia	2	2	85967.67	260497.8	40
6535	line*coccidia	2	3	147414.2	260497.8	40
6536	line	1		333751.3	63092.69	40
6536	line	2		327688.6	63092.69	40
6536	coccidia		0	374215.6	89226.54	40
6536	coccidia		1	549369.8	89226.54	40
6536	coccidia		2	165958.6	89226.54	40
6536	coccidia		3	233335.8	89226.54	40
6536	line*coccidia	1	0	431118.5	126185.4	40
6536	line*coccidia	1	1	211074.3	126185.4	40
6536	line*coccidia	1	2	226140.8	126185.4	40

6536	line*coccidia	1	3	466671.5	126185.4	40
6536	line*coccidia	2	0	317312.7	126185.4	40
6536	line*coccidia	2	1	887665.3	126185.4	40
6536	line*coccidia	2	2	105776.3	126185.4	40
6536	line*coccidia	2	3	-1.5E-11	126185.4	40
6539	line	1		2946896	573587.3	40
6539	line	2		2917368	573587.3	40
6539	coccidia		0	2219750	811174.9	40
6539	coccidia		1	3072937	811174.9	40
6539	coccidia		2	2042736	811174.9	40
6539	coccidia		3	4393104	811174.9	40
6539	line*coccidia	1	0	2431392	1147175	40
6539	line*coccidia	1	1	2099854	1147175	40
6539	line*coccidia	1	2	2799957	1147175	40
6539	line*coccidia	1	3	4456380	1147175	40
6539	line*coccidia	2	0	2008109	1147175	40
6539	line*coccidia	2	1	4046020	1147175	40
6539	line*coccidia	2	2	1285515	1147175	40
6539	line*coccidia	2	3	4329828	1147175	40
6540	line	1		3725340	761859	40
6540	line	2		5000227	761859	40
6540	coccidia		0	8667040	1077431	40
6540	coccidia		1	2224916	1077431	40
6540	coccidia		2	4494380	1077431	40
6540	coccidia		3	2064797	1077431	40
6540	line*coccidia	1	0	9777543	1523718	40
6540	line*coccidia	1	1	1575349	1523718	40
6540	line*coccidia	1	2	1833191	1523718	40
6540	line*coccidia	1	3	1715276	1523718	40
6540	line*coccidia	2	0	7556538	1523718	40
6540	line*coccidia	2	1	2874484	1523718	40
6540	line*coccidia	2	2	7155568	1523718	40
6540	line*coccidia	2	3	2414319	1523718	40
6542	line	1		5001395	643640.3	40
6542	line	2		4730182	643640.3	40
6542	coccidia		0	3838138	910244.8	40
6542	coccidia		1	7328572	910244.8	40
6542	coccidia		2	4004776	910244.8	40
6542	coccidia		3	4291668	910244.8	40
6542	line*coccidia	1	0	3741476	1287281	40
6542	line*coccidia	1	1	5926792	1287281	40
6542	line*coccidia	1	2	5093686	1287281	40
6542	line*coccidia	1	3	5243626	1287281	40
6542	line*coccidia	2	0	3934800	1287281	40
6542	line*coccidia	2	1	8730352	1287281	40
6542	line*coccidia	2	2	2915865	1287281	40
6542	line*coccidia	2	3	3339711	1287281	40
6543	line	1		6858435	1816720	40
6543	line	2		7789544	1816720	40

6543	coccidia		0	16531621	2569230	40
6543	coccidia		1	2000775	2569230	40
6543	coccidia		2	5326819	2569230	40
6543	coccidia		3	5436744	2569230	40
6543	line*coccidia	1	0	14761713	3633440	40
6543	line*coccidia	1	1	2306487	3633440	40
6543	line*coccidia	1	2	3762841	3633440	40
6543	line*coccidia	1	3	6602699	3633440	40
6543	line*coccidia	2	0	18301529	3633440	40
6543	line*coccidia	2	1	1695064	3633440	40
6543	line*coccidia	2	2	6890796	3633440	40
6543	line*coccidia	2	3	4270788	3633440	40
6544	line	1		7755519	1102370	40
6544	line	2		5490949	1102370	40
6544	coccidia		0	14104246	1558987	40
6544	coccidia		1	2764449	1558987	40
6544	coccidia		2	4885394	1558987	40
6544	coccidia		3	4738848	1558987	40
6544	line*coccidia	1	0	19941164	2204741	40
6544	line*coccidia	1	1	3340737	2204741	40
6544	line*coccidia	1	2	2075651	2204741	40
6544	line*coccidia	1	3	5664526	2204741	40
6544	line*coccidia	2	0	8267328	2204741	40
6544	line*coccidia	2	1	2188161	2204741	40
6544	line*coccidia	2	2	7695138	2204741	40
6544	line*coccidia	2	3	3813169	2204741	40
6601	line	1		922754.3	82886.95	40
6601	line	2		626311.7	82886.95	40
6601	coccidia		0	1047559	117219.8	40
6601	coccidia		1	513137.7	117219.8	40
6601	coccidia		2	373374.5	117219.8	40
6601	coccidia		3	1164061	117219.8	40
6601	line*coccidia	1	0	1233493	165773.9	40
6601	line*coccidia	1	1	374641.3	165773.9	40
6601	line*coccidia	1	2	294554.8	165773.9	40
6601	line*coccidia	1	3	1788328	165773.9	40
6601	line*coccidia	2	0	861624	165773.9	40
6601	line*coccidia	2	1	651634	165773.9	40
6601	line*coccidia	2	2	452194.2	165773.9	40
6601	line*coccidia	2	3	539794.7	165773.9	40
6602	line	1		1688569	152524	40
6602	line	2		1630948	152524	40
6602	coccidia		0	2144862	215701.5	40
6602	coccidia		1	1462826	215701.5	40
6602	coccidia		2	1144229	215701.5	40
6602	coccidia		3	1887118	215701.5	40
6602	line*coccidia	1	0	2175863	305048	40
6602	line*coccidia	1	1	923584.3	305048	40
6602	line*coccidia	1	2	995341.7	305048	40

6602	line*coccidia	1	3	2659488	305048	40
6602	line*coccidia	2	0	2113860	305048	40
6602	line*coccidia	2	1	2002069	305048	40
6602	line*coccidia	2	2	1293116	305048	40
6602	line*coccidia	2	3	1114749	305048	40
6604	line	1		559007.7	69134.21	40
6604	line	2		432687.7	69134.21	40
6604	coccidia		0	610304.8	97770.54	40
6604	coccidia		1	540407.2	97770.54	40
6604	coccidia		2	255318	97770.54	40
6604	coccidia		3	577360.7	97770.54	40
6604	line*coccidia	1	0	722900	138268.4	40
6604	line*coccidia	1	1	447341.3	138268.4	40
6604	line*coccidia	1	2	279331.7	138268.4	40
6604	line*coccidia	1	3	786457.7	138268.4	40
6604	line*coccidia	2	0	497709.7	138268.4	40
6604	line*coccidia	2	1	633473	138268.4	40
6604	line*coccidia	2	2	231304.3	138268.4	40
6604	line*coccidia	2	3	368263.7	138268.4	40
6605	line	1		4770018	386607.4	40
6605	line	2		4228058	386607.4	40
6605	coccidia		0	5748367	546745.4	40
6605	coccidia		1	3076667	546745.4	40
6605	coccidia		2	3108265	546745.4	40
6605	coccidia		3	6062853	546745.4	40
6605	line*coccidia	1	0	6121232	773214.7	40
6605	line*coccidia	1	1	2786371	773214.7	40
6605	line*coccidia	1	2	2279358	773214.7	40
6605	line*coccidia	1	3	7893112	773214.7	40
6605	line*coccidia	2	0	5375503	773214.7	40
6605	line*coccidia	2	1	3366964	773214.7	40
6605	line*coccidia	2	2	3937172	773214.7	40
6605	line*coccidia	2	3	4232594	773214.7	40
6606	line	1		1433253	112885.6	40
6606	line	2		1389021	112885.6	40
6606	coccidia		0	2079668	159644.3	40
6606	coccidia		1	921986.8	159644.3	40
6606	coccidia		2	755652.7	159644.3	40
6606	coccidia		3	1887242	159644.3	40
6606	line*coccidia	1	0	2086333	225771.1	40
6606	line*coccidia	1	1	755336.5	225771.1	40
6606	line*coccidia	1	2	506330.7	225771.1	40
6606	line*coccidia	1	3	2385014	225771.1	40
6606	line*coccidia	2	0	2073003	225771.1	40
6606	line*coccidia	2	1	1088637	225771.1	40
6606	line*coccidia	2	2	1004975	225771.1	40
6606	line*coccidia	2	3	1389470	225771.1	40
6607	line	1		459215.5	36306.27	40
6607	line	2		413435.1	36306.27	40

6607	coccidia		0	469133.6	51344.82	40
6607	coccidia		1	530429.3	51344.82	40
6607	coccidia		2	270687.7	51344.82	40
6607	coccidia		3	475050.6	51344.82	40
6607	line*coccidia	1	0	634107.8	72612.54	40
6607	line*coccidia	1	1	254249.3	72612.54	40
6607	line*coccidia	1	2	288628.5	72612.54	40
6607	line*coccidia	1	3	659876.2	72612.54	40
6607	line*coccidia	2	0	304159.3	72612.54	40
6607	line*coccidia	2	1	806609.2	72612.54	40
6607	line*coccidia	2	2	252746.8	72612.54	40
6607	line*coccidia	2	3	290225	72612.54	40
6608	line	1		1721020	156132.2	40
6608	line	2		1674023	156132.2	40
6608	coccidia		0	1958892	220804.3	40
6608	coccidia		1	1545561	220804.3	40
6608	coccidia		2	1106013	220804.3	40
6608	coccidia		3	2179621	220804.3	40
6608	line*coccidia	1	0	2103862	312264.5	40
6608	line*coccidia	1	1	950042.3	312264.5	40
6608	line*coccidia	1	2	893604.3	312264.5	40
6608	line*coccidia	1	3	2936571	312264.5	40
6608	line*coccidia	2	0	1813921	312264.5	40
6608	line*coccidia	2	1	2141080	312264.5	40
6608	line*coccidia	2	2	1318422	312264.5	40
6608	line*coccidia	2	3	1422672	312264.5	40
6609	line	1		1135413	120699.3	40
6609	line	2		1233485	120699.3	40
6609	coccidia		0	1787486	170694.6	40
6609	coccidia		1	809521	170694.6	40
6609	coccidia		2	647606.5	170694.6	40
6609	coccidia		3	1493182	170694.6	40
6609	line*coccidia	1	0	1702787	241398.6	40
6609	line*coccidia	1	1	490551	241398.6	40
6609	line*coccidia	1	2	405788.3	241398.6	40
6609	line*coccidia	1	3	1942526	241398.6	40
6609	line*coccidia	2	0	1872186	241398.6	40
6609	line*coccidia	2	1	1128491	241398.6	40
6609	line*coccidia	2	2	889424.7	241398.6	40
6609	line*coccidia	2	3	1043838	241398.6	40
6610	line	1		1031919	144996.9	40
6610	line	2		1207283	144996.9	40
6610	coccidia		0	1279451	205056.6	40
6610	coccidia		1	1071024	205056.6	40
6610	coccidia		2	741023.5	205056.6	40
6610	coccidia		3	1386906	205056.6	40
6610	line*coccidia	1	0	1295959	289993.9	40
6610	line*coccidia	1	1	458434	289993.9	40
6610	line*coccidia	1	2	565596	289993.9	40

6610	line*coccidia	1	3	1807686	289993.9	40
6610	line*coccidia	2	0	1262943	289993.9	40
6610	line*coccidia	2	1	1683615	289993.9	40
6610	line*coccidia	2	2	916451	289993.9	40
6610	line*coccidia	2	3	966125	289993.9	40
6611	line	1		4889058	406196.8	40
6611	line	2		4601559	406196.8	40
6611	coccidia		0	5925855	574449	40
6611	coccidia		1	3611346	574449	40
6611	coccidia		2	3358923	574449	40
6611	coccidia		3	6085112	574449	40
6611	line*coccidia	1	0	6454976	812393.5	40
6611	line*coccidia	1	1	2898273	812393.5	40
6611	line*coccidia	1	2	2817174	812393.5	40
6611	line*coccidia	1	3	7385811	812393.5	40
6611	line*coccidia	2	0	5396734	812393.5	40
6611	line*coccidia	2	1	4324419	812393.5	40
6611	line*coccidia	2	2	3900672	812393.5	40
6611	line*coccidia	2	3	4784413	812393.5	40
6612	line	1		1036271	81055.92	40
6612	line	2		940374.8	81055.92	40
6612	coccidia		0	1359688	114630.4	40
6612	coccidia		1	771291.1	114630.4	40
6612	coccidia		2	592526.2	114630.4	40
6612	coccidia		3	1229787	114630.4	40
6612	line*coccidia	1	0	1348218	162111.8	40
6612	line*coccidia	1	1	421052.3	162111.8	40
6612	line*coccidia	1	2	560510	162111.8	40
6612	line*coccidia	1	3	1815305	162111.8	40
6612	line*coccidia	2	0	1371158	162111.8	40
6612	line*coccidia	2	1	1121530	162111.8	40
6612	line*coccidia	2	2	624542.3	162111.8	40
6612	line*coccidia	2	3	644269.3	162111.8	40
6614	line	1		664208	70642.04	40
6614	line	2		751628.4	70642.04	40
6614	coccidia		0	903412.5	99902.93	40
6614	coccidia		1	582731.5	99902.93	40
6614	coccidia		2	456079.1	99902.93	40
6614	coccidia		3	889449.6	99902.93	40
6614	line*coccidia	1	0	1036643	141284.1	40
6614	line*coccidia	1	1	197364.7	141284.1	40
6614	line*coccidia	1	2	364255.2	141284.1	40
6614	line*coccidia	1	3	1058569	141284.1	40
6614	line*coccidia	2	0	770181.8	141284.1	40
6614	line*coccidia	2	1	968098.3	141284.1	40
6614	line*coccidia	2	2	547903	141284.1	40
6614	line*coccidia	2	3	720330.3	141284.1	40
6615	line	1		401680.4	73950.26	40
6615	line	2		690381.6	73950.26	40

6615	coccidia		0	618739.5	104581.5	40
6615	coccidia		1	615061.8	104581.5	40
6615	coccidia		2	413824.7	104581.5	40
6615	coccidia		3	536497.9	104581.5	40
6615	line*coccidia	1	0	600755	147900.5	40
6615	line*coccidia	1	1	205628.7	147900.5	40
6615	line*coccidia	1	2	277437.8	147900.5	40
6615	line*coccidia	1	3	522900	147900.5	40
6615	line*coccidia	2	0	636724	147900.5	40
6615	line*coccidia	2	1	1024495	147900.5	40
6615	line*coccidia	2	2	550211.5	147900.5	40
6615	line*coccidia	2	3	550095.8	147900.5	40
6616	line	1		5709439	432488.6	40
6616	line	2		4723218	432488.6	40
6616	coccidia		0	6765244	611631.2	40
6616	coccidia		1	3809454	611631.2	40
6616	coccidia		2	3903425	611631.2	40
6616	coccidia		3	6387192	611631.2	40
6616	line*coccidia	1	0	8129177	864977.2	40
6616	line*coccidia	1	1	3801821	864977.2	40
6616	line*coccidia	1	2	3504708	864977.2	40
6616	line*coccidia	1	3	7402052	864977.2	40
6616	line*coccidia	2	0	5401312	864977.2	40
6616	line*coccidia	2	1	3817086	864977.2	40
6616	line*coccidia	2	2	4302143	864977.2	40
6616	line*coccidia	2	3	5372332	864977.2	40
6621	line	1		1481240	257969.8	40
6621	line	2		1525189	257969.8	40
6621	coccidia		0	2512453	364824.4	40
6621	coccidia		1	1160379	364824.4	40
6621	coccidia		2	876924.1	364824.4	40
6621	coccidia		3	1463102	364824.4	40
6621	line*coccidia	1	0	2952043	515939.6	40
6621	line*coccidia	1	1	299674.8	515939.6	40
6621	line*coccidia	1	2	660166.8	515939.6	40
6621	line*coccidia	1	3	2013074	515939.6	40
6621	line*coccidia	2	0	2072863	515939.6	40
6621	line*coccidia	2	1	2021083	515939.6	40
6621	line*coccidia	2	2	1093681	515939.6	40
6621	line*coccidia	2	3	913129.5	515939.6	40
6624	line	1		505952.3	132825.3	40
6624	line	2		790831.4	132825.3	40
6624	coccidia		0	426574.8	187843.3	40
6624	coccidia		1	1447885	187843.3	40
6624	coccidia		2	422762.9	187843.3	40
6624	coccidia		3	296345.1	187843.3	40
6624	line*coccidia	1	0	442575.8	265650.6	40
6624	line*coccidia	1	1	1044242	265650.6	40
6624	line*coccidia	1	2	129983.3	265650.6	40

6624	line*coccidia	1	3	407007.8	265650.6	40
6624	line*coccidia	2	0	410573.7	265650.6	40
6624	line*coccidia	2	1	1851527	265650.6	40
6624	line*coccidia	2	2	715542.5	265650.6	40
6624	line*coccidia	2	3	185682.3	265650.6	40
6629	line	1		1544761	172967.9	40
6629	line	2		1716931	172967.9	40
6629	coccidia		0	2101840	244613.6	40
6629	coccidia		1	1325583	244613.6	40
6629	coccidia		2	1252924	244613.6	40
6629	coccidia		3	1843038	244613.6	40
6629	line*coccidia	1	0	2255329	345935.8	40
6629	line*coccidia	1	1	700560.5	345935.8	40
6629	line*coccidia	1	2	972502.8	345935.8	40
6629	line*coccidia	1	3	2250651	345935.8	40
6629	line*coccidia	2	0	1948350	345935.8	40
6629	line*coccidia	2	1	1950605	345935.8	40
6629	line*coccidia	2	2	1533346	345935.8	40
6629	line*coccidia	2	3	1435424	345935.8	40
6630	line	1		912804.7	101058.7	40
6630	line	2		863639.7	101058.7	40
6630	coccidia		0	1112417	142918.6	40
6630	coccidia		1	693145.7	142918.6	40
6630	coccidia		2	484376.1	142918.6	40
6630	coccidia		3	1262951	142918.6	40
6630	line*coccidia	1	0	1120363	202117.4	40
6630	line*coccidia	1	1	417029.2	202117.4	40
6630	line*coccidia	1	2	359355.3	202117.4	40
6630	line*coccidia	1	3	1754471	202117.4	40
6630	line*coccidia	2	0	1104470	202117.4	40
6630	line*coccidia	2	1	969262.2	202117.4	40
6630	line*coccidia	2	2	609396.8	202117.4	40
6630	line*coccidia	2	3	771430	202117.4	40
6631	line	1		1717831	235446.3	40
6631	line	2		2392149	235446.3	40
6631	coccidia		0	2415812	332971.3	40
6631	coccidia		1	1554180	332971.3	40
6631	coccidia		2	1788571	332971.3	40
6631	coccidia		3	2461397	332971.3	40
6631	line*coccidia	1	0	1980511	470892.6	40
6631	line*coccidia	1	1	792911	470892.6	40
6631	line*coccidia	1	2	1145655	470892.6	40
6631	line*coccidia	1	3	2952245	470892.6	40
6631	line*coccidia	2	0	2851113	470892.6	40
6631	line*coccidia	2	1	2315448	470892.6	40
6631	line*coccidia	2	2	2431487	470892.6	40
6631	line*coccidia	2	3	1970549	470892.6	40
6632	line	1		394241	45803.85	40
6632	line	2		390027.6	45803.85	40

6632	coccidia		0	507800.6	64776.43	40
6632	coccidia		1	256691.8	64776.43	40
6632	coccidia		2	243017.7	64776.43	40
6632	coccidia		3	561027.1	64776.43	40
6632	line*coccidia	1	0	598857	91607.71	40
6632	line*coccidia	1	1	38562.5	91607.71	40
6632	line*coccidia	1	2	132569	91607.71	40
6632	line*coccidia	1	3	806975.3	91607.71	40
6632	line*coccidia	2	0	416744.2	91607.71	40
6632	line*coccidia	2	1	474821	91607.71	40
6632	line*coccidia	2	2	353466.3	91607.71	40
6632	line*coccidia	2	3	315078.8	91607.71	40
6633	line	1		455272.2	60560.33	40
6633	line	2		627059	60560.33	40
6633	coccidia		0	385535.3	85645.24	40
6633	coccidia		1	424505.9	85645.24	40
6633	coccidia		2	401583.8	85645.24	40
6633	coccidia		3	953037.3	85645.24	40
6633	line*coccidia	1	0	382245.3	121120.7	40
6633	line*coccidia	1	1	158304.2	121120.7	40
6633	line*coccidia	1	2	276689.2	121120.7	40
6633	line*coccidia	1	3	1003850	121120.7	40
6633	line*coccidia	2	0	388825.2	121120.7	40
6633	line*coccidia	2	1	690707.7	121120.7	40
6633	line*coccidia	2	2	526478.5	121120.7	40
6633	line*coccidia	2	3	902224.5	121120.7	40
6634	line	1		1370867	190228.1	40
6634	line	2		1454925	190228.1	40
6634	coccidia		0	2054152	269023.1	40
6634	coccidia		1	1224327	269023.1	40
6634	coccidia		2	773356.4	269023.1	40
6634	coccidia		3	1599748	269023.1	40
6634	line*coccidia	1	0	2342567	380456.2	40
6634	line*coccidia	1	1	503116.7	380456.2	40
6634	line*coccidia	1	2	580543.7	380456.2	40
6634	line*coccidia	1	3	2057241	380456.2	40
6634	line*coccidia	2	0	1765736	380456.2	40
6634	line*coccidia	2	1	1945537	380456.2	40
6634	line*coccidia	2	2	966169.2	380456.2	40
6634	line*coccidia	2	3	1142256	380456.2	40
6635	line	1		82963.92	40760.83	40
6635	line	2		291062.7	40760.83	40
6635	coccidia		0	361158.6	57644.52	40
6635	coccidia		1	38547.42	57644.52	40
6635	coccidia		2	100566.7	57644.52	40
6635	coccidia		3	247780.5	57644.52	40
6635	line*coccidia	1	0	254760.8	81521.67	40
6635	line*coccidia	1	1	77094.83	81521.67	40
6635	line*coccidia	1	2	-5.8E-11	81521.67	40

6635	line*coccidia	1	3	-5.8E-11	81521.67	40
6635	line*coccidia	2	0	467556.3	81521.67	40
6635	line*coccidia	2	1	-1.2E-10	81521.67	40
6635	line*coccidia	2	2	201133.3	81521.67	40
6635	line*coccidia	2	3	495561	81521.67	40
6637	line	1		1782777	283660.6	40
6637	line	2		2088051	283660.6	40
6637	coccidia		0	2773385	401156.7	40
6637	coccidia		1	1494517	401156.7	40
6637	coccidia		2	1262784	401156.7	40
6637	coccidia		3	2210969	401156.7	40
6637	line*coccidia	1	0	2391163	567321.3	40
6637	line*coccidia	1	1	845729.3	567321.3	40
6637	line*coccidia	1	2	973668.3	567321.3	40
6637	line*coccidia	1	3	2920546	567321.3	40
6637	line*coccidia	2	0	3155607	567321.3	40
6637	line*coccidia	2	1	2143304	567321.3	40
6637	line*coccidia	2	2	1551900	567321.3	40
6637	line*coccidia	2	3	1501392	567321.3	40
6703	line	1		1565181	182545.2	40
6703	line	2		1354939	182545.2	40
6703	coccidia		0	1442454	258157.9	40
6703	coccidia		1	1692210	258157.9	40
6703	coccidia		2	1112279	258157.9	40
6703	coccidia		3	1593297	258157.9	40
6703	line*coccidia	1	0	1808839	365090.4	40
6703	line*coccidia	1	1	1359555	365090.4	40
6703	line*coccidia	1	2	1019882	365090.4	40
6703	line*coccidia	1	3	2072448	365090.4	40
6703	line*coccidia	2	0	1076069	365090.4	40
6703	line*coccidia	2	1	2024866	365090.4	40
6703	line*coccidia	2	2	1204676	365090.4	40
6703	line*coccidia	2	3	1114147	365090.4	40
6707	line	1		94646.83	17099.53	40
6707	line	2		90476.58	17099.53	40
6707	coccidia		0	116027.2	24182.39	40
6707	coccidia		1	116604.8	24182.39	40
6707	coccidia		2	84498.92	24182.39	40
6707	coccidia		3	53116	24182.39	40
6707	line*coccidia	1	0	103333.3	34199.06	40
6707	line*coccidia	1	1	139099.7	34199.06	40
6707	line*coccidia	1	2	84277	34199.06	40
6707	line*coccidia	1	3	51877.33	34199.06	40
6707	line*coccidia	2	0	128721	34199.06	40
6707	line*coccidia	2	1	94109.83	34199.06	40
6707	line*coccidia	2	2	84720.83	34199.06	40
6707	line*coccidia	2	3	54354.67	34199.06	40
6713	line	1		97940.88	19139.63	40
6713	line	2		145797.4	19139.63	40

6713	coccidia		0	136985.1	27067.52	40
6713	coccidia		1	96316.58	27067.52	40
6713	coccidia		2	187418.8	27067.52	40
6713	coccidia		3	66756	27067.52	40
6713	line*coccidia	1	0	67654.67	38279.26	40
6713	line*coccidia	1	1	97379.33	38279.26	40
6713	line*coccidia	1	2	149693.8	38279.26	40
6713	line*coccidia	1	3	77035.67	38279.26	40
6713	line*coccidia	2	0	206315.5	38279.26	40
6713	line*coccidia	2	1	95253.83	38279.26	40
6713	line*coccidia	2	2	225143.8	38279.26	40
6713	line*coccidia	2	3	56476.33	38279.26	40
6715	line	1		282301.9	165220.3	40
6715	line	2		753483.3	165220.3	40
6715	coccidia		0	972177.9	233656.8	40
6715	coccidia		1	359075.5	233656.8	40
6715	coccidia		2	266581	233656.8	40
6715	coccidia		3	473736.1	233656.8	40
6715	line*coccidia	1	0	533178	330440.6	40
6715	line*coccidia	1	1	131961.7	330440.6	40
6715	line*coccidia	1	2	139389.8	330440.6	40
6715	line*coccidia	1	3	324678.2	330440.6	40
6715	line*coccidia	2	0	1411178	330440.6	40
6715	line*coccidia	2	1	586189.3	330440.6	40
6715	line*coccidia	2	2	393772.2	330440.6	40
6715	line*coccidia	2	3	622794	330440.6	40
6717	line	1		318373.2	118221.5	40
6717	line	2		667075.5	118221.5	40
6717	coccidia		0	646775.3	167190.5	40
6717	coccidia		1	588716.1	167190.5	40
6717	coccidia		2	261138.3	167190.5	40
6717	coccidia		3	474267.7	167190.5	40
6717	line*coccidia	1	0	584268.3	236443.1	40
6717	line*coccidia	1	1	6951.5	236443.1	40
6717	line*coccidia	1	2	176157.8	236443.1	40
6717	line*coccidia	1	3	506115.2	236443.1	40
6717	line*coccidia	2	0	709282.3	236443.1	40
6717	line*coccidia	2	1	1170481	236443.1	40
6717	line*coccidia	2	2	346118.7	236443.1	40
6717	line*coccidia	2	3	442420.2	236443.1	40
6718	line	1		783720.3	106309	40
6718	line	2		800861.1	106309	40
6718	coccidia		0	803306.5	150343.6	40
6718	coccidia		1	1066163	150343.6	40
6718	coccidia		2	591866.3	150343.6	40
6718	coccidia		3	707827.2	150343.6	40
6718	line*coccidia	1	0	690976.2	212618	40
6718	line*coccidia	1	1	974910.5	212618	40
6718	line*coccidia	1	2	497714.2	212618	40

6718	line*coccidia	1	3	971280.2	212618	40
6718	line*coccidia	2	0	915636.8	212618	40
6718	line*coccidia	2	1	1157415	212618	40
6718	line*coccidia	2	2	686018.5	212618	40
6718	line*coccidia	2	3	444374.2	212618	40
6720	line	1		456598.4	116637.4	40
6720	line	2		103214.7	116637.4	40
6720	coccidia		0	98897.08	164950.2	40
6720	coccidia		1	695590.8	164950.2	40
6720	coccidia		2	177257.8	164950.2	40
6720	coccidia		3	147880.5	164950.2	40
6720	line*coccidia	1	0	96023.83	233274.8	40
6720	line*coccidia	1	1	1226882	233274.8	40
6720	line*coccidia	1	2	274814.5	233274.8	40
6720	line*coccidia	1	3	228673.7	233274.8	40
6720	line*coccidia	2	0	101770.3	233274.8	40
6720	line*coccidia	2	1	164300	233274.8	40
6720	line*coccidia	2	2	79701.17	233274.8	40
6720	line*coccidia	2	3	67087.33	233274.8	40
6721	line	1		1453037	334821.1	40
6721	line	2		1429132	334821.1	40
6721	coccidia		0	993144.1	473508.6	40
6721	coccidia		1	1890608	473508.6	40
6721	coccidia		2	1443964	473508.6	40
6721	coccidia		3	1436621	473508.6	40
6721	line*coccidia	1	0	800638	669642.3	40
6721	line*coccidia	1	1	1291702	669642.3	40
6721	line*coccidia	1	2	1774220	669642.3	40
6721	line*coccidia	1	3	1945587	669642.3	40
6721	line*coccidia	2	0	1185650	669642.3	40
6721	line*coccidia	2	1	2489514	669642.3	40
6721	line*coccidia	2	2	1113708	669642.3	40
6721	line*coccidia	2	3	927655	669642.3	40
6722	line	1		1309398	206819	40
6722	line	2		1241370	206819	40
6722	coccidia		0	763622.8	292486.3	40
6722	coccidia		1	2383921	292486.3	40
6722	coccidia		2	1143800	292486.3	40
6722	coccidia		3	810190.7	292486.3	40
6722	line*coccidia	1	0	791550.7	413638	40
6722	line*coccidia	1	1	2002931	413638	40
6722	line*coccidia	1	2	1220934	413638	40
6722	line*coccidia	1	3	1222175	413638	40
6722	line*coccidia	2	0	735695	413638	40
6722	line*coccidia	2	1	2764911	413638	40
6722	line*coccidia	2	2	1066667	413638	40
6722	line*coccidia	2	3	398206	413638	40
6723	line	1		1148325	153079.9	40
6723	line	2		982352.5	153079.9	40

6723	coccidia		0	1055169	216487.7	40
6723	coccidia		1	1142646	216487.7	40
6723	coccidia		2	902472.8	216487.7	40
6723	coccidia		3	1161068	216487.7	40
6723	line*coccidia	1	0	972630.8	306159.8	40
6723	line*coccidia	1	1	1090803	306159.8	40
6723	line*coccidia	1	2	975670.2	306159.8	40
6723	line*coccidia	1	3	1554197	306159.8	40
6723	line*coccidia	2	0	1137707	306159.8	40
6723	line*coccidia	2	1	1194489	306159.8	40
6723	line*coccidia	2	2	829275.5	306159.8	40
6723	line*coccidia	2	3	767939.3	306159.8	40
6724	line	1		251055.3	45538.62	40
6724	line	2		155876	45538.62	40
6724	coccidia		0	136714.7	64401.34	40
6724	coccidia		1	212094.4	64401.34	40
6724	coccidia		2	132371.9	64401.34	40
6724	coccidia		3	332681.4	64401.34	40
6724	line*coccidia	1	0	120296.5	91077.25	40
6724	line*coccidia	1	1	190034.7	91077.25	40
6724	line*coccidia	1	2	99720.67	91077.25	40
6724	line*coccidia	1	3	594169.2	91077.25	40
6724	line*coccidia	2	0	153132.8	91077.25	40
6724	line*coccidia	2	1	234154.2	91077.25	40
6724	line*coccidia	2	2	165023.2	91077.25	40
6724	line*coccidia	2	3	71193.67	91077.25	40
6729	line	1		754133.8	196698.4	40
6729	line	2		974664.2	196698.4	40
6729	coccidia		0	643021.3	278173.5	40
6729	coccidia		1	1183868	278173.5	40
6729	coccidia		2	945618.3	278173.5	40
6729	coccidia		3	685088.3	278173.5	40
6729	line*coccidia	1	0	480717.5	393396.8	40
6729	line*coccidia	1	1	752028	393396.8	40
6729	line*coccidia	1	2	682405.5	393396.8	40
6729	line*coccidia	1	3	1101384	393396.8	40
6729	line*coccidia	2	0	805325.2	393396.8	40
6729	line*coccidia	2	1	1615708	393396.8	40
6729	line*coccidia	2	2	1208831	393396.8	40
6729	line*coccidia	2	3	268792.3	393396.8	40
6732	line	1		14111.25	4088.595	40
6732	line	2		-3.9E-13	4088.595	40
6732	coccidia		0	0	5782.146	40
6732	coccidia		1	28222.5	5782.146	40
6732	coccidia		2	0	5782.146	40
6732	coccidia		3	0	5782.146	40
6732	line*coccidia	1	0	0	8177.19	40
6732	line*coccidia	1	1	56445	8177.19	40
6732	line*coccidia	1	2	5.22E-13	8177.19	40

6732	line*coccidia	1	3	5.22E-13	8177.19	40
6732	line*coccidia	2	0	0	8177.19	40
6732	line*coccidia	2	1	-5.2E-13	8177.19	40
6732	line*coccidia	2	2	-5.2E-13	8177.19	40
6732	line*coccidia	2	3	-5.2E-13	8177.19	40
6736	line	1		150459.8	25456.08	40
6736	line	2		107005.8	25456.08	40
6736	coccidia		0	171668	36000.33	40
6736	coccidia		1	164305.7	36000.33	40
6736	coccidia		2	65841.08	36000.33	40
6736	coccidia		3	113116.5	36000.33	40
6736	line*coccidia	1	0	159709.5	50912.16	40
6736	line*coccidia	1	1	209855.5	50912.16	40
6736	line*coccidia	1	2	59903.33	50912.16	40
6736	line*coccidia	1	3	172371	50912.16	40
6736	line*coccidia	2	0	183626.5	50912.16	40
6736	line*coccidia	2	1	118755.8	50912.16	40
6736	line*coccidia	2	2	71778.83	50912.16	40
6736	line*coccidia	2	3	53862	50912.16	40
6737	line	1		32859.54	6433.665	40
6737	line	2		27509.58	6433.665	40
6737	coccidia		0	55809.25	9098.576	40
6737	coccidia		1	18385.67	9098.576	40
6737	coccidia		2	28534.42	9098.576	40
6737	coccidia		3	18008.92	9098.576	40
6737	line*coccidia	1	0	58649	12867.33	40
6737	line*coccidia	1	1	36771.33	12867.33	40
6737	line*coccidia	1	2	0	12867.33	40
6737	line*coccidia	1	3	36017.83	12867.33	40
6737	line*coccidia	2	0	52969.5	12867.33	40
6737	line*coccidia	2	1	0	12867.33	40
6737	line*coccidia	2	2	57068.83	12867.33	40
6737	line*coccidia	2	3	0	12867.33	40
6738	line	1		153457.9	25858.13	40
6738	line	2		89902.42	25858.13	40
6738	coccidia		0	127826.9	36568.92	40
6738	coccidia		1	142903	36568.92	40
6738	coccidia		2	103915.9	36568.92	40
6738	coccidia		3	112074.8	36568.92	40
6738	line*coccidia	1	0	126190	51716.26	40
6738	line*coccidia	1	1	164828.7	51716.26	40
6738	line*coccidia	1	2	144416.2	51716.26	40
6738	line*coccidia	1	3	178396.8	51716.26	40
6738	line*coccidia	2	0	129463.8	51716.26	40
6738	line*coccidia	2	1	120977.3	51716.26	40
6738	line*coccidia	2	2	63415.67	51716.26	40
6738	line*coccidia	2	3	45752.83	51716.26	40
6739	line	1		77050.25	14705.11	40
6739	line	2		72187.08	14705.11	40

6739	coccidia		0	65503.75	20796.16	40
6739	coccidia		1	92238.33	20796.16	40
6739	coccidia		2	83743.92	20796.16	40
6739	coccidia		3	56988.67	20796.16	40
6739	line*coccidia	1	0	59725.33	29410.22	40
6739	line*coccidia	1	1	95853	29410.22	40
6739	line*coccidia	1	2	89662.5	29410.22	40
6739	line*coccidia	1	3	62960.17	29410.22	40
6739	line*coccidia	2	0	71282.17	29410.22	40
6739	line*coccidia	2	1	88623.67	29410.22	40
6739	line*coccidia	2	2	77825.33	29410.22	40
6739	line*coccidia	2	3	51017.17	29410.22	40
6740	line	1		46673.46	15666.5	40
6740	line	2		-1.2E-12	15666.5	40
6740	coccidia		0	51342.5	22155.78	40
6740	coccidia		1	42004.42	22155.78	40
6740	coccidia		2	1.21E-12	22155.78	40
6740	coccidia		3	-3.6E-12	22155.78	40
6740	line*coccidia	1	0	102685	31333	40
6740	line*coccidia	1	1	84008.83	31333	40
6740	line*coccidia	1	2	3.2E-12	31333	40
6740	line*coccidia	1	3	-6.5E-12	31333	40
6740	line*coccidia	2	0	0	31333	40
6740	line*coccidia	2	1	-3.2E-12	31333	40
6740	line*coccidia	2	2	-7.8E-13	31333	40
6740	line*coccidia	2	3	-7.8E-13	31333	40
6741	line	1		1439084	262391.2	40
6741	line	2		1481806	262391.2	40
6741	coccidia		0	1095698	371077.1	40
6741	coccidia		1	1872564	371077.1	40
6741	coccidia		2	1431265	371077.1	40
6741	coccidia		3	1442252	371077.1	40
6741	line*coccidia	1	0	858730.7	524782.3	40
6741	line*coccidia	1	1	1428018	524782.3	40
6741	line*coccidia	1	2	1706715	524782.3	40
6741	line*coccidia	1	3	1762871	524782.3	40
6741	line*coccidia	2	0	1332665	524782.3	40
6741	line*coccidia	2	1	2317109	524782.3	40
6741	line*coccidia	2	2	1155815	524782.3	40
6741	line*coccidia	2	3	1121634	524782.3	40
6801	line	1		-3.5E-10	56442.4	40
6801	line	2		583749.9	56442.4	40
6801	coccidia		0	270099.5	79821.6	40
6801	coccidia		1	360878.6	79821.6	40
6801	coccidia		2	189119.9	79821.6	40
6801	coccidia		3	347401.8	79821.6	40
6801	line*coccidia	1	0	-3.5E-10	112884.8	40
6801	line*coccidia	1	1	-3.5E-10	112884.8	40
6801	line*coccidia	1	2	-3.5E-10	112884.8	40

6801	line*coccidia	1	3	-3.5E-10	112884.8	40
6801	line*coccidia	2	0	540199	112884.8	40
6801	line*coccidia	2	1	721757.2	112884.8	40
6801	line*coccidia	2	2	378239.8	112884.8	40
6801	line*coccidia	2	3	694803.5	112884.8	40
6802	line	1		891604.8	171040.6	40
6802	line	2		1068091	171040.6	40
6802	coccidia		0	978151.1	241887.9	40
6802	coccidia		1	1234219	241887.9	40
6802	coccidia		2	847779.3	241887.9	40
6802	coccidia		3	859242.8	241887.9	40
6802	line*coccidia	1	0	738824.5	342081.2	40
6802	line*coccidia	1	1	899834.5	342081.2	40
6802	line*coccidia	1	2	538505.7	342081.2	40
6802	line*coccidia	1	3	1389255	342081.2	40
6802	line*coccidia	2	0	1217478	342081.2	40
6802	line*coccidia	2	1	1568604	342081.2	40
6802	line*coccidia	2	2	1157053	342081.2	40
6802	line*coccidia	2	3	329231	342081.2	40
6805	line	1		1.16E-10	60656	40
6805	line	2		548794.9	60656	40
6805	coccidia		0	163502.8	85780.54	40
6805	coccidia		1	474697.9	85780.54	40
6805	coccidia		2	176770.5	85780.54	40
6805	coccidia		3	282618.6	85780.54	40
6805	line*coccidia	1	0	1.16E-10	121312	40
6805	line*coccidia	1	1	1.16E-10	121312	40
6805	line*coccidia	1	2	1.16E-10	121312	40
6805	line*coccidia	1	3	1.16E-10	121312	40
6805	line*coccidia	2	0	327005.5	121312	40
6805	line*coccidia	2	1	949395.8	121312	40
6805	line*coccidia	2	2	353541	121312	40
6805	line*coccidia	2	3	565237.2	121312	40
6807	line	1		-5.8E-11	42425.39	40
6807	line	2		496267	42425.39	40
6807	coccidia		0	196855.6	59998.56	40
6807	coccidia		1	296514.3	59998.56	40
6807	coccidia		2	247434.7	59998.56	40
6807	coccidia		3	251729.4	59998.56	40
6807	line*coccidia	1	0	-5.8E-11	84850.77	40
6807	line*coccidia	1	1	-5.8E-11	84850.77	40
6807	line*coccidia	1	2	-5.8E-11	84850.77	40
6807	line*coccidia	1	3	-5.8E-11	84850.77	40
6807	line*coccidia	2	0	393711.2	84850.77	40
6807	line*coccidia	2	1	593028.7	84850.77	40
6807	line*coccidia	2	2	494869.3	84850.77	40
6807	line*coccidia	2	3	503458.8	84850.77	40
6808	line	1		661347.9	137051	40
6808	line	2		681650.7	137051	40

6808	coccidia		0	472109.8	193819.4	40
6808	coccidia		1	1075317	193819.4	40
6808	coccidia		2	528226.7	193819.4	40
6808	coccidia		3	610344	193819.4	40
6808	line*coccidia	1	0	712625.3	274102.1	40
6808	line*coccidia	1	1	572380.3	274102.1	40
6808	line*coccidia	1	2	388376.8	274102.1	40
6808	line*coccidia	1	3	972009	274102.1	40
6808	line*coccidia	2	0	231594.3	274102.1	40
6808	line*coccidia	2	1	1578253	274102.1	40
6808	line*coccidia	2	2	668076.5	274102.1	40
6808	line*coccidia	2	3	248679	274102.1	40
6809	line	1		-2.5E-10	55875.79	40
6809	line	2		682054.5	55875.79	40
6809	coccidia		0	209803.2	79020.3	40
6809	coccidia		1	479711.5	79020.3	40
6809	coccidia		2	323265.8	79020.3	40
6809	coccidia		3	351328.6	79020.3	40
6809	line*coccidia	1	0	-2.9E-10	111751.6	40
6809	line*coccidia	1	1	-2.6E-10	111751.6	40
6809	line*coccidia	1	2	-2.3E-10	111751.6	40
6809	line*coccidia	1	3	-2.3E-10	111751.6	40
6809	line*coccidia	2	0	419606.3	111751.6	40
6809	line*coccidia	2	1	959423	111751.6	40
6809	line*coccidia	2	2	646531.5	111751.6	40
6809	line*coccidia	2	3	702657.2	111751.6	40
6810	line	1		5.82E-11	66423.31	40
6810	line	2		664692.5	66423.31	40
6810	coccidia		0	248105.3	93936.75	40
6810	coccidia		1	422899.1	93936.75	40
6810	coccidia		2	309439.7	93936.75	40
6810	coccidia		3	348940.9	93936.75	40
6810	line*coccidia	1	0	5.82E-11	132846.6	40
6810	line*coccidia	1	1	8.73E-11	132846.6	40
6810	line*coccidia	1	2	8.73E-11	132846.6	40
6810	line*coccidia	1	3	0	132846.6	40
6810	line*coccidia	2	0	496210.5	132846.6	40
6810	line*coccidia	2	1	845798.2	132846.6	40
6810	line*coccidia	2	2	618879.3	132846.6	40
6810	line*coccidia	2	3	697881.8	132846.6	40
6811	line	1		684377.9	74932.16	40
6811	line	2		778688.7	74932.16	40
6811	coccidia		0	767767.3	105970.1	40
6811	coccidia		1	777799.2	105970.1	40
6811	coccidia		2	686401.9	105970.1	40
6811	coccidia		3	694164.9	105970.1	40
6811	line*coccidia	1	0	775326.2	149864.3	40
6811	line*coccidia	1	1	439414.3	149864.3	40
6811	line*coccidia	1	2	371257.5	149864.3	40

6811	line*coccidia	1	3	1151514	149864.3	40
6811	line*coccidia	2	0	760208.3	149864.3	40
6811	line*coccidia	2	1	1116184	149864.3	40
6811	line*coccidia	2	2	1001546	149864.3	40
6811	line*coccidia	2	3	236816.2	149864.3	40
6813	line	1		565663.1	95931.72	40
6813	line	2		727896	95931.72	40
6813	coccidia		0	840650.1	135667.9	40
6813	coccidia		1	668109.1	135667.9	40
6813	coccidia		2	455067.2	135667.9	40
6813	coccidia		3	623291.9	135667.9	40
6813	line*coccidia	1	0	751147.2	191863.4	40
6813	line*coccidia	1	1	364766.5	191863.4	40
6813	line*coccidia	1	2	195182	191863.4	40
6813	line*coccidia	1	3	951556.7	191863.4	40
6813	line*coccidia	2	0	930153	191863.4	40
6813	line*coccidia	2	1	971451.7	191863.4	40
6813	line*coccidia	2	2	714952.3	191863.4	40
6813	line*coccidia	2	3	295027.2	191863.4	40
6814	line	1		-1.2E-10	114675.7	40
6814	line	2		663386.4	114675.7	40
6814	coccidia		0	200489	162175.9	40
6814	coccidia		1	596188.8	162175.9	40
6814	coccidia		2	233865.8	162175.9	40
6814	coccidia		3	296229.2	162175.9	40
6814	line*coccidia	1	0	-1.5E-10	229351.4	40
6814	line*coccidia	1	1	-1.2E-10	229351.4	40
6814	line*coccidia	1	2	-1.2E-10	229351.4	40
6814	line*coccidia	1	3	-1.2E-10	229351.4	40
6814	line*coccidia	2	0	400978	229351.4	40
6814	line*coccidia	2	1	1192378	229351.4	40
6814	line*coccidia	2	2	467731.5	229351.4	40
6814	line*coccidia	2	3	592458.3	229351.4	40
6816	line	1		743461.3	139985.4	40
6816	line	2		645719	139985.4	40
6816	coccidia		0	574980.3	197969.2	40
6816	coccidia		1	704204.1	197969.2	40
6816	coccidia		2	518155.8	197969.2	40
6816	coccidia		3	981020.3	197969.2	40
6816	line*coccidia	1	0	529424.3	279970.7	40
6816	line*coccidia	1	1	337358.8	279970.7	40
6816	line*coccidia	1	2	423334.5	279970.7	40
6816	line*coccidia	1	3	1683727	279970.7	40
6816	line*coccidia	2	0	620536.2	279970.7	40
6816	line*coccidia	2	1	1071049	279970.7	40
6816	line*coccidia	2	2	612977.2	279970.7	40
6816	line*coccidia	2	3	278313.3	279970.7	40
6817	line	1		5.64E-11	69984.44	40
6817	line	2		561990	69984.44	40

6817	coccidia		0	207144.7	98972.95	40
6817	coccidia		1	373632.5	98972.95	40
6817	coccidia		2	260758.5	98972.95	40
6817	coccidia		3	282444.3	98972.95	40
6817	line*coccidia	1	0	8.73E-11	139968.9	40
6817	line*coccidia	1	1	5.82E-11	139968.9	40
6817	line*coccidia	1	2	8E-11	139968.9	40
6817	line*coccidia	1	3	0	139968.9	40
6817	line*coccidia	2	0	414289.3	139968.9	40
6817	line*coccidia	2	1	747265	139968.9	40
6817	line*coccidia	2	2	521517	139968.9	40
6817	line*coccidia	2	3	564888.5	139968.9	40
6818	line	1		449110.1	263069.8	40
6818	line	2		1226404	263069.8	40
6818	coccidia		0	560022.4	372036.9	40
6818	coccidia		1	1523843	372036.9	40
6818	coccidia		2	641821.5	372036.9	40
6818	coccidia		3	625340.5	372036.9	40
6818	line*coccidia	1	0	434076.8	526139.6	40
6818	line*coccidia	1	1	237274	526139.6	40
6818	line*coccidia	1	2	288126.5	526139.6	40
6818	line*coccidia	1	3	836963.2	526139.6	40
6818	line*coccidia	2	0	685968	526139.6	40
6818	line*coccidia	2	1	2810413	526139.6	40
6818	line*coccidia	2	2	995516.5	526139.6	40
6818	line*coccidia	2	3	413717.8	526139.6	40
6824	line	1		72570.75	17485.21	40
6824	line	2		82492.08	17485.21	40
6824	coccidia		0	68083.33	24727.83	40
6824	coccidia		1	74903.08	24727.83	40
6824	coccidia		2	63308.25	24727.83	40
6824	coccidia		3	103831	24727.83	40
6824	line*coccidia	1	0	33700.33	34970.43	40
6824	line*coccidia	1	1	90046	34970.43	40
6824	line*coccidia	1	2	60366.33	34970.43	40
6824	line*coccidia	1	3	106170.3	34970.43	40
6824	line*coccidia	2	0	102466.3	34970.43	40
6824	line*coccidia	2	1	59760.17	34970.43	40
6824	line*coccidia	2	2	66250.17	34970.43	40
6824	line*coccidia	2	3	101491.7	34970.43	40
6825	line	1		55296.08	20916.01	40
6825	line	2		65324.21	20916.01	40
6825	coccidia		0	41678.67	29579.7	40
6825	coccidia		1	106530.8	29579.7	40
6825	coccidia		2	48588.42	29579.7	40
6825	coccidia		3	44442.67	29579.7	40
6825	line*coccidia	1	0	21390.33	41832.01	40
6825	line*coccidia	1	1	71931.33	41832.01	40
6825	line*coccidia	1	2	77204.67	41832.01	40

6825	line*coccidia	1	3	50658	41832.01	40
6825	line*coccidia	2	0	61967	41832.01	40
6825	line*coccidia	2	1	141130.3	41832.01	40
6825	line*coccidia	2	2	19972.17	41832.01	40
6825	line*coccidia	2	3	38227.33	41832.01	40
6904	line	1		663713.3	214345.5	40
6904	line	2		830748.5	214345.5	40
6904	coccidia		0	695698.6	303130.4	40
6904	coccidia		1	1387544	303130.4	40
6904	coccidia		2	398452.9	303130.4	40
6904	coccidia		3	507227.9	303130.4	40
6904	line*coccidia	1	0	672637.3	428691.1	40
6904	line*coccidia	1	1	755295.8	428691.1	40
6904	line*coccidia	1	2	334532.5	428691.1	40
6904	line*coccidia	1	3	892387.7	428691.1	40
6904	line*coccidia	2	0	718759.8	428691.1	40
6904	line*coccidia	2	1	2019793	428691.1	40
6904	line*coccidia	2	2	462373.3	428691.1	40
6904	line*coccidia	2	3	122068.2	428691.1	40
6905	line	1		857330.3	275887.4	40
6905	line	2		1295308	275887.4	40
6905	coccidia		0	1107373	390163.7	40
6905	coccidia		1	1484353	390163.7	40
6905	coccidia		2	650334	390163.7	40
6905	coccidia		3	1063216	390163.7	40
6905	line*coccidia	1	0	1074244	551774.8	40
6905	line*coccidia	1	1	208790	551774.8	40
6905	line*coccidia	1	2	241546	551774.8	40
6905	line*coccidia	1	3	1904742	551774.8	40
6905	line*coccidia	2	0	1140502	551774.8	40
6905	line*coccidia	2	1	2759917	551774.8	40
6905	line*coccidia	2	2	1059122	551774.8	40
6905	line*coccidia	2	3	221690.5	551774.8	40
7001	line	1		4.55E-12	13556.07	40
7001	line	2		72489.33	13556.07	40
7001	coccidia		0	52929.5	19171.17	40
7001	coccidia		1	22397.08	19171.17	40
7001	coccidia		2	26909.42	19171.17	40
7001	coccidia		3	42742.67	19171.17	40
7001	line*coccidia	1	0	0	27112.13	40
7001	line*coccidia	1	1	0	27112.13	40
7001	line*coccidia	1	2	3.64E-12	27112.13	40
7001	line*coccidia	1	3	1.46E-11	27112.13	40
7001	line*coccidia	2	0	105859	27112.13	40
7001	line*coccidia	2	1	44794.17	27112.13	40
7001	line*coccidia	2	2	53818.83	27112.13	40
7001	line*coccidia	2	3	85485.33	27112.13	40
7002	line	1		53784.58	10366.7	40
7002	line	2		66939.13	10366.7	40

7002	coccidia		0	61070.75	14660.73	40
7002	coccidia		1	73476.08	14660.73	40
7002	coccidia		2	40078.17	14660.73	40
7002	coccidia		3	66822.42	14660.73	40
7002	line*coccidia	1	0	55051.67	20733.4	40
7002	line*coccidia	1	1	65549.67	20733.4	40
7002	line*coccidia	1	2	33414.17	20733.4	40
7002	line*coccidia	1	3	61122.83	20733.4	40
7002	line*coccidia	2	0	67089.83	20733.4	40
7002	line*coccidia	2	1	81402.5	20733.4	40
7002	line*coccidia	2	2	46742.17	20733.4	40
7002	line*coccidia	2	3	72522	20733.4	40
7003	line	1		41707.42	7096.459	40
7003	line	2		69001.54	7096.459	40
7003	coccidia		0	59308.25	10035.91	40
7003	coccidia		1	57267.83	10035.91	40
7003	coccidia		2	47050.42	10035.91	40
7003	coccidia		3	57791.42	10035.91	40
7003	line*coccidia	1	0	34964.83	14192.92	40
7003	line*coccidia	1	1	32227.83	14192.92	40
7003	line*coccidia	1	2	31566.33	14192.92	40
7003	line*coccidia	1	3	68070.67	14192.92	40
7003	line*coccidia	2	0	83651.67	14192.92	40
7003	line*coccidia	2	1	82307.83	14192.92	40
7003	line*coccidia	2	2	62534.5	14192.92	40
7003	line*coccidia	2	3	47512.17	14192.92	40
7005	line	1		8332.792	4632.909	40
7005	line	2		40317.96	4632.909	40
7005	coccidia		0	28124.17	6551.923	40
7005	coccidia		1	34848.42	6551.923	40
7005	coccidia		2	22389.17	6551.923	40
7005	coccidia		3	11939.75	6551.923	40
7005	line*coccidia	1	0	-7.3E-12	9265.819	40
7005	line*coccidia	1	1	33331.17	9265.819	40
7005	line*coccidia	1	2	0	9265.819	40
7005	line*coccidia	1	3	0	9265.819	40
7005	line*coccidia	2	0	56248.33	9265.819	40
7005	line*coccidia	2	1	36365.67	9265.819	40
7005	line*coccidia	2	2	44778.33	9265.819	40
7005	line*coccidia	2	3	23879.5	9265.819	40
7007	line	1		85579.96	39974.35	40
7007	line	2		219219.4	39974.35	40
7007	coccidia		0	101663.4	56532.27	40
7007	coccidia		1	148376.8	56532.27	40
7007	coccidia		2	244554.3	56532.27	40
7007	coccidia		3	115004.2	56532.27	40
7007	line*coccidia	1	0	62271.5	79948.7	40
7007	line*coccidia	1	1	75736.67	79948.7	40
7007	line*coccidia	1	2	62087.83	79948.7	40

7007	line*coccidia	1	3	142223.8	79948.7	40
7007	line*coccidia	2	0	141055.3	79948.7	40
7007	line*coccidia	2	1	221017	79948.7	40
7007	line*coccidia	2	2	427020.7	79948.7	40
7007	line*coccidia	2	3	87784.5	79948.7	40
7011	line	1		6568.5	4803.399	40
7011	line	2		18083.17	4803.399	40
7011	coccidia		0	17460.58	6793.032	40
7011	coccidia		1	0	6793.032	40
7011	coccidia		2	6112.833	6793.032	40
7011	coccidia		3	25729.92	6793.032	40
7011	line*coccidia	1	0	13711.17	9606.798	40
7011	line*coccidia	1	1	0	9606.798	40
7011	line*coccidia	1	2	-1.8E-12	9606.798	40
7011	line*coccidia	1	3	12562.83	9606.798	40
7011	line*coccidia	2	0	21210	9606.798	40
7011	line*coccidia	2	1	0	9606.798	40
7011	line*coccidia	2	2	12225.67	9606.798	40
7011	line*coccidia	2	3	38897	9606.798	40
7012	line	1		527558.5	144225.1	40
7012	line	2		4984.833	144225.1	40
7012	coccidia		0	0	203965.1	40
7012	coccidia		1	1019414	203965.1	40
7012	coccidia		2	9969.667	203965.1	40
7012	coccidia		3	35703.17	203965.1	40
7012	line*coccidia	1	0	0	288450.2	40
7012	line*coccidia	1	1	2038828	288450.2	40
7012	line*coccidia	1	2	6.18E-11	288450.2	40
7012	line*coccidia	1	3	71406.33	288450.2	40
7012	line*coccidia	2	0	0	288450.2	40
7012	line*coccidia	2	1	2.18E-11	288450.2	40
7012	line*coccidia	2	2	19939.33	288450.2	40
7012	line*coccidia	2	3	-1.8E-11	288450.2	40
7101	line	1		447707.3	103478.7	40
7101	line	2		326428.2	103478.7	40
7101	coccidia		0	542245.3	146341	40
7101	coccidia		1	178823.3	146341	40
7101	coccidia		2	310367.3	146341	40
7101	coccidia		3	516835.3	146341	40
7101	line*coccidia	1	0	914230.3	206957.4	40
7101	line*coccidia	1	1	80308.83	206957.4	40
7101	line*coccidia	1	2	183780.5	206957.4	40
7101	line*coccidia	1	3	612509.7	206957.4	40
7101	line*coccidia	2	0	170260.2	206957.4	40
7101	line*coccidia	2	1	277337.8	206957.4	40
7101	line*coccidia	2	2	436954	206957.4	40
7101	line*coccidia	2	3	421160.8	206957.4	40
7102	line	1		195455	44146.79	40
7102	line	2		57459.71	44146.79	40

7102	coccidia		0	67892.08	62432.99	40
7102	coccidia		1	354745.8	62432.99	40
7102	coccidia		2	36535.58	62432.99	40
7102	coccidia		3	46655.83	62432.99	40
7102	line*coccidia	1	0	59442.67	88293.58	40
7102	line*coccidia	1	1	628631.7	88293.58	40
7102	line*coccidia	1	2	27484.33	88293.58	40
7102	line*coccidia	1	3	66261.17	88293.58	40
7102	line*coccidia	2	0	76341.5	88293.58	40
7102	line*coccidia	2	1	80860	88293.58	40
7102	line*coccidia	2	2	45586.83	88293.58	40
7102	line*coccidia	2	3	27050.5	88293.58	40
7108	line	1		390605.2	38944.97	40
7108	line	2		503259.9	38944.97	40
7108	coccidia		0	396158.7	55076.5	40
7108	coccidia		1	491287	55076.5	40
7108	coccidia		2	350002	55076.5	40
7108	coccidia		3	550282.5	55076.5	40
7108	line*coccidia	1	0	285618	77889.94	40
7108	line*coccidia	1	1	458899.3	77889.94	40
7108	line*coccidia	1	2	217102.2	77889.94	40
7108	line*coccidia	1	3	600801.3	77889.94	40
7108	line*coccidia	2	0	506699.3	77889.94	40
7108	line*coccidia	2	1	523674.7	77889.94	40
7108	line*coccidia	2	2	482901.8	77889.94	40
7108	line*coccidia	2	3	499763.7	77889.94	40
7109	line	1		139314.1	24184.68	40
7109	line	2		165171.6	24184.68	40
7109	coccidia		0	92343.08	34202.3	40
7109	coccidia		1	220158.6	34202.3	40
7109	coccidia		2	156121.9	34202.3	40
7109	coccidia		3	140347.9	34202.3	40
7109	line*coccidia	1	0	97115.33	48369.35	40
7109	line*coccidia	1	1	164571.3	48369.35	40
7109	line*coccidia	1	2	105661.7	48369.35	40
7109	line*coccidia	1	3	189908.2	48369.35	40
7109	line*coccidia	2	0	87570.83	48369.35	40
7109	line*coccidia	2	1	275745.8	48369.35	40
7109	line*coccidia	2	2	206582.2	48369.35	40
7109	line*coccidia	2	3	90787.67	48369.35	40
7110	line	1		586835.4	167735.7	40
7110	line	2		335983.5	167735.7	40
7110	coccidia		0	508052.9	237214.1	40
7110	coccidia		1	203051	237214.1	40
7110	coccidia		2	276992.8	237214.1	40
7110	coccidia		3	857541.3	237214.1	40
7110	line*coccidia	1	0	899709.7	335471.3	40
7110	line*coccidia	1	1	77732.17	335471.3	40
7110	line*coccidia	1	2	181427.7	335471.3	40

7110	line*coccidia	1	3	1188472	335471.3	40
7110	line*coccidia	2	0	116396.2	335471.3	40
7110	line*coccidia	2	1	328369.8	335471.3	40
7110	line*coccidia	2	2	372557.8	335471.3	40
7110	line*coccidia	2	3	526610.3	335471.3	40
7111	line	1		14088.63	23466.96	40
7111	line	2		52840.42	23466.96	40
7111	coccidia		0	6588.417	33187.29	40
7111	coccidia		1	96393.75	33187.29	40
7111	coccidia		2	24869.67	33187.29	40
7111	coccidia		3	6006.25	33187.29	40
7111	line*coccidia	1	0	4.09E-12	46933.91	40
7111	line*coccidia	1	1	30272.83	46933.91	40
7111	line*coccidia	1	2	26081.67	46933.91	40
7111	line*coccidia	1	3	-5.5E-12	46933.91	40
7111	line*coccidia	2	0	13176.83	46933.91	40
7111	line*coccidia	2	1	162514.7	46933.91	40
7111	line*coccidia	2	2	23657.67	46933.91	40
7111	line*coccidia	2	3	12012.5	46933.91	40
7114	line	1		555088.6	61365.58	40
7114	line	2		793338.8	61365.58	40
7114	coccidia		0	575841.3	86784.04	40
7114	coccidia		1	558483.1	86784.04	40
7114	coccidia		2	789703.5	86784.04	40
7114	coccidia		3	772827	86784.04	40
7114	line*coccidia	1	0	515867.3	122731.2	40
7114	line*coccidia	1	1	318167.2	122731.2	40
7114	line*coccidia	1	2	405386	122731.2	40
7114	line*coccidia	1	3	980934	122731.2	40
7114	line*coccidia	2	0	635815.2	122731.2	40
7114	line*coccidia	2	1	798799	122731.2	40
7114	line*coccidia	2	2	1174021	122731.2	40
7114	line*coccidia	2	3	564720	122731.2	40
7115	line	1		13037.92	3858.139	40
7115	line	2		21848.92	3858.139	40
7115	coccidia		0	20201	5456.233	40
7115	coccidia		1	17526.25	5456.233	40
7115	coccidia		2	15918.5	5456.233	40
7115	coccidia		3	16127.92	5456.233	40
7115	line*coccidia	1	0	9207.833	7716.279	40
7115	line*coccidia	1	1	18856.5	7716.279	40
7115	line*coccidia	1	2	16900.17	7716.279	40
7115	line*coccidia	1	3	7187.167	7716.279	40
7115	line*coccidia	2	0	31194.17	7716.279	40
7115	line*coccidia	2	1	16196	7716.279	40
7115	line*coccidia	2	2	14936.83	7716.279	40
7115	line*coccidia	2	3	25068.67	7716.279	40
7116	line	1		297902.6	29121.03	40
7116	line	2		354611.9	29121.03	40

7116	coccidia		0	351417.2	41183.36	40
7116	coccidia		1	308255.7	41183.36	40
7116	coccidia		2	206953.5	41183.36	40
7116	coccidia		3	438402.7	41183.36	40
7116	line*coccidia	1	0	298749.7	58242.07	40
7116	line*coccidia	1	1	149385.2	58242.07	40
7116	line*coccidia	1	2	165186.3	58242.07	40
7116	line*coccidia	1	3	578289.3	58242.07	40
7116	line*coccidia	2	0	404084.7	58242.07	40
7116	line*coccidia	2	1	467126.2	58242.07	40
7116	line*coccidia	2	2	248720.7	58242.07	40
7116	line*coccidia	2	3	298516	58242.07	40
7117	line	1		64418.46	29912.89	40
7117	line	2		109688.7	29912.89	40
7117	coccidia		0	61548.25	42303.21	40
7117	coccidia		1	133511.3	42303.21	40
7117	coccidia		2	54531.58	42303.21	40
7117	coccidia		3	98623.25	42303.21	40
7117	line*coccidia	1	0	45483.5	59825.78	40
7117	line*coccidia	1	1	30285.33	59825.78	40
7117	line*coccidia	1	2	48219	59825.78	40
7117	line*coccidia	1	3	133686	59825.78	40
7117	line*coccidia	2	0	77613	59825.78	40
7117	line*coccidia	2	1	236737.2	59825.78	40
7117	line*coccidia	2	2	60844.17	59825.78	40
7117	line*coccidia	2	3	63560.5	59825.78	40
7118	line	1		669962.9	163841.7	40
7118	line	2		528547	163841.7	40
7118	coccidia		0	921537.7	231707.1	40
7118	coccidia		1	106750	231707.1	40
7118	coccidia		2	438944.8	231707.1	40
7118	coccidia		3	929787.2	231707.1	40
7118	line*coccidia	1	0	1322595	327683.4	40
7118	line*coccidia	1	1	93797.67	327683.4	40
7118	line*coccidia	1	2	374265.3	327683.4	40
7118	line*coccidia	1	3	889193.7	327683.4	40
7118	line*coccidia	2	0	520480.5	327683.4	40
7118	line*coccidia	2	1	119702.3	327683.4	40
7118	line*coccidia	2	2	503624.3	327683.4	40
7118	line*coccidia	2	3	970380.7	327683.4	40
7119	line	1		18210.08	15573.24	40
7119	line	2		89833.21	15573.24	40
7119	coccidia		0	44493	22023.88	40
7119	coccidia		1	96233.33	22023.88	40
7119	coccidia		2	55569.92	22023.88	40
7119	coccidia		3	19790.33	22023.88	40
7119	line*coccidia	1	0	31492.67	31146.47	40
7119	line*coccidia	1	1	-1.5E-11	31146.47	40
7119	line*coccidia	1	2	41347.67	31146.47	40

7119	line*coccidia	1	3	1.46E-11	31146.47	40
7119	line*coccidia	2	0	57493.33	31146.47	40
7119	line*coccidia	2	1	192466.7	31146.47	40
7119	line*coccidia	2	2	69792.17	31146.47	40
7119	line*coccidia	2	3	39580.67	31146.47	40
7120	line	1		34399.25	12505.9	40
7120	line	2		50228.46	12505.9	40
7120	coccidia		0	70855.5	17686.01	40
7120	coccidia		1	55334.33	17686.01	40
7120	coccidia		2	21792.42	17686.01	40
7120	coccidia		3	21273.17	17686.01	40
7120	line*coccidia	1	0	88106.17	25011.8	40
7120	line*coccidia	1	1	16649.33	25011.8	40
7120	line*coccidia	1	2	12286.5	25011.8	40
7120	line*coccidia	1	3	20555	25011.8	40
7120	line*coccidia	2	0	53604.83	25011.8	40
7120	line*coccidia	2	1	94019.33	25011.8	40
7120	line*coccidia	2	2	31298.33	25011.8	40
7120	line*coccidia	2	3	21991.33	25011.8	40
7121	line	1		27361.54	4319.293	40
7121	line	2		41207.38	4319.293	40
7121	coccidia		0	41029.5	6108.402	40
7121	coccidia		1	48205.67	6108.402	40
7121	coccidia		2	22074.17	6108.402	40
7121	coccidia		3	25828.5	6108.402	40
7121	line*coccidia	1	0	23385.33	8638.586	40
7121	line*coccidia	1	1	41320	8638.586	40
7121	line*coccidia	1	2	15447.83	8638.586	40
7121	line*coccidia	1	3	29293	8638.586	40
7121	line*coccidia	2	0	58673.67	8638.586	40
7121	line*coccidia	2	1	55091.33	8638.586	40
7121	line*coccidia	2	2	28700.5	8638.586	40
7121	line*coccidia	2	3	22364	8638.586	40
7122	line	1		31597.54	6844.833	40
7122	line	2		19788.33	6844.833	40
7122	coccidia		0	20699.67	9680.055	40
7122	coccidia		1	48487.08	9680.055	40
7122	coccidia		2	18506	9680.055	40
7122	coccidia		3	15079	9680.055	40
7122	line*coccidia	1	0	26837.83	13689.67	40
7122	line*coccidia	1	1	60720.5	13689.67	40
7122	line*coccidia	1	2	16499.83	13689.67	40
7122	line*coccidia	1	3	22332	13689.67	40
7122	line*coccidia	2	0	14561.5	13689.67	40
7122	line*coccidia	2	1	36253.67	13689.67	40
7122	line*coccidia	2	2	20512.17	13689.67	40
7122	line*coccidia	2	3	7826	13689.67	40
7123	line	1		14506.42	1823.636	40
7123	line	2		8681.875	1823.636	40

7123	coccidia		0	11809.17	2579.011	40
7123	coccidia		1	14740.83	2579.011	40
7123	coccidia		2	11794.25	2579.011	40
7123	coccidia		3	8032.333	2579.011	40
7123	line*coccidia	1	0	12782.17	3647.272	40
7123	line*coccidia	1	1	13492	3647.272	40
7123	line*coccidia	1	2	15686.83	3647.272	40
7123	line*coccidia	1	3	16064.67	3647.272	40
7123	line*coccidia	2	0	10836.17	3647.272	40
7123	line*coccidia	2	1	15989.67	3647.272	40
7123	line*coccidia	2	2	7901.667	3647.272	40
7123	line*coccidia	2	3	6.37E-12	3647.272	40
7129	line	1		160305.3	17494.29	40
7129	line	2		214881.5	17494.29	40
7129	coccidia		0	276402.9	24740.67	40
7129	coccidia		1	190692.1	24740.67	40
7129	coccidia		2	140406.7	24740.67	40
7129	coccidia		3	142872	24740.67	40
7129	line*coccidia	1	0	276276.7	34988.59	40
7129	line*coccidia	1	1	99120.67	34988.59	40
7129	line*coccidia	1	2	42305.67	34988.59	40
7129	line*coccidia	1	3	223518.2	34988.59	40
7129	line*coccidia	2	0	276529.2	34988.59	40
7129	line*coccidia	2	1	282263.5	34988.59	40
7129	line*coccidia	2	2	238507.7	34988.59	40
7129	line*coccidia	2	3	62225.83	34988.59	40
7130	line	1		117791.8	39159.54	40
7130	line	2		3.68E-13	39159.54	40
7130	coccidia		0	0	55379.96	40
7130	coccidia		1	235583.6	55379.96	40
7130	coccidia		2	0	55379.96	40
7130	coccidia		3	0	55379.96	40
7130	line*coccidia	1	0	0	78319.09	40
7130	line*coccidia	1	1	471167.2	78319.09	40
7130	line*coccidia	1	2	9.21E-12	78319.09	40
7130	line*coccidia	1	3	-1E-11	78319.09	40
7130	line*coccidia	2	0	0	78319.09	40
7130	line*coccidia	2	1	4.9E-13	78319.09	40
7130	line*coccidia	2	2	-9.2E-12	78319.09	40
7130	line*coccidia	2	3	1.02E-11	78319.09	40
7131	line	1		257785.8	259267.5	40
7131	line	2		620718	259267.5	40
7131	coccidia		0	187849.5	366659.6	40
7131	coccidia		1	1159550	366659.6	40
7131	coccidia		2	141452.7	366659.6	40
7131	coccidia		3	268155.8	366659.6	40
7131	line*coccidia	1	0	124049.7	518534.9	40
7131	line*coccidia	1	1	584798.2	518534.9	40
7131	line*coccidia	1	2	83663	518534.9	40

7131	line*coccidia	1	3	238632.3	518534.9	40
7131	line*coccidia	2	0	251649.3	518534.9	40
7131	line*coccidia	2	1	1734301	518534.9	40
7131	line*coccidia	2	2	199242.3	518534.9	40
7131	line*coccidia	2	3	297679.3	518534.9	40
7201	line	1		12511.5	136917.7	40
7201	line	2		208827.2	136917.7	40
7201	coccidia		0	9165.833	193630.9	40
7201	coccidia		1	394950	193630.9	40
7201	coccidia		2	25330.17	193630.9	40
7201	coccidia		3	13231.33	193630.9	40
7201	line*coccidia	1	0	8354.167	273835.4	40
7201	line*coccidia	1	1	2.73E-12	273835.4	40
7201	line*coccidia	1	2	29840.83	273835.4	40
7201	line*coccidia	1	3	11851	273835.4	40
7201	line*coccidia	2	0	9977.5	273835.4	40
7201	line*coccidia	2	1	789900	273835.4	40
7201	line*coccidia	2	2	20819.5	273835.4	40
7201	line*coccidia	2	3	14611.67	273835.4	40
7202	line	1		28461.71	43673.84	40
7202	line	2		95398.08	43673.84	40
7202	coccidia		0	27822.5	61764.14	40
7202	coccidia		1	172093.6	61764.14	40
7202	coccidia		2	18311.33	61764.14	40
7202	coccidia		3	29492.17	61764.14	40
7202	line*coccidia	1	0	27802.33	87347.68	40
7202	line*coccidia	1	1	44138.5	87347.68	40
7202	line*coccidia	1	2	13222.83	87347.68	40
7202	line*coccidia	1	3	28683.17	87347.68	40
7202	line*coccidia	2	0	27842.67	87347.68	40
7202	line*coccidia	2	1	300048.7	87347.68	40
7202	line*coccidia	2	2	23399.83	87347.68	40
7202	line*coccidia	2	3	30301.17	87347.68	40
7203	line	1		49103.75	5542.6	40
7203	line	2		56136.21	5542.6	40
7203	coccidia		0	53929.25	7838.42	40
7203	coccidia		1	45262.25	7838.42	40
7203	coccidia		2	44597.17	7838.42	40
7203	coccidia		3	66691.25	7838.42	40
7203	line*coccidia	1	0	53043.67	11085.2	40
7203	line*coccidia	1	1	22109	11085.2	40
7203	line*coccidia	1	2	36254.5	11085.2	40
7203	line*coccidia	1	3	85007.83	11085.2	40
7203	line*coccidia	2	0	54814.83	11085.2	40
7203	line*coccidia	2	1	68415.5	11085.2	40
7203	line*coccidia	2	2	52939.83	11085.2	40
7203	line*coccidia	2	3	48374.67	11085.2	40
7204	line	1		38482.96	608837.4	40
7204	line	2		1041473	608837.4	40

7204	coccidia		0	305984.6	861026.2	40
7204	coccidia		1	1766931	861026.2	40
7204	coccidia		2	32002.08	861026.2	40
7204	coccidia		3	54994.08	861026.2	40
7204	line*coccidia	1	0	37280	1217675	40
7204	line*coccidia	1	1	11975.83	1217675	40
7204	line*coccidia	1	2	23010.83	1217675	40
7204	line*coccidia	1	3	81665.17	1217675	40
7204	line*coccidia	2	0	574689.2	1217675	40
7204	line*coccidia	2	1	3521886	1217675	40
7204	line*coccidia	2	2	40993.33	1217675	40
7204	line*coccidia	2	3	28323	1217675	40
7205	line	1		27549.42	6944.346	40
7205	line	2		29783.79	6944.346	40
7205	coccidia		0	24917.25	9820.788	40
7205	coccidia		1	18822.75	9820.788	40
7205	coccidia		2	19320.67	9820.788	40
7205	coccidia		3	51605.75	9820.788	40
7205	line*coccidia	1	0	19971.83	13888.69	40
7205	line*coccidia	1	1	15707.67	13888.69	40
7205	line*coccidia	1	2	17540	13888.69	40
7205	line*coccidia	1	3	56978.17	13888.69	40
7205	line*coccidia	2	0	29862.67	13888.69	40
7205	line*coccidia	2	1	21937.83	13888.69	40
7205	line*coccidia	2	2	21101.33	13888.69	40
7205	line*coccidia	2	3	46233.33	13888.69	40
7206	line	1		43389.46	6613.331	40
7206	line	2		50885.29	6613.331	40
7206	coccidia		0	51544.92	9352.663	40
7206	coccidia		1	56646	9352.663	40
7206	coccidia		2	49708.17	9352.663	40
7206	coccidia		3	30650.42	9352.663	40
7206	line*coccidia	1	0	34748.5	13226.66	40
7206	line*coccidia	1	1	29599.5	13226.66	40
7206	line*coccidia	1	2	66033.5	13226.66	40
7206	line*coccidia	1	3	43176.33	13226.66	40
7206	line*coccidia	2	0	68341.33	13226.66	40
7206	line*coccidia	2	1	83692.5	13226.66	40
7206	line*coccidia	2	2	33382.83	13226.66	40
7206	line*coccidia	2	3	18124.5	13226.66	40
7207	line	1		81014.13	40491.13	40
7207	line	2		151944.3	40491.13	40
7207	coccidia		0	192017.9	57263.11	40
7207	coccidia		1	143382.1	57263.11	40
7207	coccidia		2	35724.75	57263.11	40
7207	coccidia		3	94792.17	57263.11	40
7207	line*coccidia	1	0	61126.67	80982.27	40
7207	line*coccidia	1	1	121365.7	80982.27	40
7207	line*coccidia	1	2	23616	80982.27	40

7207	line*coccidia	1	3	117948.2	80982.27	40
7207	line*coccidia	2	0	322909.2	80982.27	40
7207	line*coccidia	2	1	165398.5	80982.27	40
7207	line*coccidia	2	2	47833.5	80982.27	40
7207	line*coccidia	2	3	71636.17	80982.27	40
7208	line	1		115392.1	23604.38	40
7208	line	2		62145.58	23604.38	40
7208	coccidia		0	67639.67	33381.63	40
7208	coccidia		1	185919.3	33381.63	40
7208	coccidia		2	46053.83	33381.63	40
7208	coccidia		3	55462.58	33381.63	40
7208	line*coccidia	1	0	54078.67	47208.76	40
7208	line*coccidia	1	1	304116	47208.76	40
7208	line*coccidia	1	2	29168.67	47208.76	40
7208	line*coccidia	1	3	74205	47208.76	40
7208	line*coccidia	2	0	81200.67	47208.76	40
7208	line*coccidia	2	1	67722.5	47208.76	40
7208	line*coccidia	2	2	62939	47208.76	40
7208	line*coccidia	2	3	36720.17	47208.76	40
7211	line	1		8640.917	3201.912	40
7211	line	2		22545.04	3201.912	40
7211	coccidia		0	20862.75	4528.188	40
7211	coccidia		1	16411.58	4528.188	40
7211	coccidia		2	10707	4528.188	40
7211	coccidia		3	14390.58	4528.188	40
7211	line*coccidia	1	0	16112.33	6403.825	40
7211	line*coccidia	1	1	-7.3E-12	6403.825	40
7211	line*coccidia	1	2	-1.8E-12	6403.825	40
7211	line*coccidia	1	3	18451.33	6403.825	40
7211	line*coccidia	2	0	25613.17	6403.825	40
7211	line*coccidia	2	1	32823.17	6403.825	40
7211	line*coccidia	2	2	21414	6403.825	40
7211	line*coccidia	2	3	10329.83	6403.825	40
7212	line	1		47988.83	96937.97	40
7212	line	2		184184.6	96937.97	40
7212	coccidia		0	40923.5	137091	40
7212	coccidia		1	332173.2	137091	40
7212	coccidia		2	34482.75	137091	40
7212	coccidia		3	56767.5	137091	40
7212	line*coccidia	1	0	36688.5	193875.9	40
7212	line*coccidia	1	1	59654.67	193875.9	40
7212	line*coccidia	1	2	40492.17	193875.9	40
7212	line*coccidia	1	3	55120	193875.9	40
7212	line*coccidia	2	0	45158.5	193875.9	40
7212	line*coccidia	2	1	604691.7	193875.9	40
7212	line*coccidia	2	2	28473.33	193875.9	40
7212	line*coccidia	2	3	58415	193875.9	40
7213	line	1		25471.08	19070.01	40
7213	line	2		44868.54	19070.01	40

7213	coccidia		0	18731.92	26969.07	40
7213	coccidia		1	72022.33	26969.07	40
7213	coccidia		2	31105.67	26969.07	40
7213	coccidia		3	18819.33	26969.07	40
7213	line*coccidia	1	0	14317.5	38140.02	40
7213	line*coccidia	1	1	17207.33	38140.02	40
7213	line*coccidia	1	2	52645	38140.02	40
7213	line*coccidia	1	3	17714.5	38140.02	40
7213	line*coccidia	2	0	23146.33	38140.02	40
7213	line*coccidia	2	1	126837.3	38140.02	40
7213	line*coccidia	2	2	9566.333	38140.02	40
7213	line*coccidia	2	3	19924.17	38140.02	40
7214	line	1		26434.63	7885.484	40
7214	line	2		13839.75	7885.484	40
7214	coccidia		0	12701.92	11151.76	40
7214	coccidia		1	36950.33	11151.76	40
7214	coccidia		2	4688.583	11151.76	40
7214	coccidia		3	26207.92	11151.76	40
7214	line*coccidia	1	0	10749	15770.97	40
7214	line*coccidia	1	1	73900.67	15770.97	40
7214	line*coccidia	1	2	3.64E-12	15770.97	40
7214	line*coccidia	1	3	21088.83	15770.97	40
7214	line*coccidia	2	0	14654.83	15770.97	40
7214	line*coccidia	2	1	3.64E-12	15770.97	40
7214	line*coccidia	2	2	9377.167	15770.97	40
7214	line*coccidia	2	3	31327	15770.97	40
7215	line	1		31141.38	328051	40
7215	line	2		582632	328051	40
7215	coccidia		0	220404.7	463934.2	40
7215	coccidia		1	939874.8	463934.2	40
7215	coccidia		2	5411.75	463934.2	40
7215	coccidia		3	61855.5	463934.2	40
7215	line*coccidia	1	0	14573.17	656102.1	40
7215	line*coccidia	1	1	13720.83	656102.1	40
7215	line*coccidia	1	2	1.82E-11	656102.1	40
7215	line*coccidia	1	3	96271.5	656102.1	40
7215	line*coccidia	2	0	426236.2	656102.1	40
7215	line*coccidia	2	1	1866029	656102.1	40
7215	line*coccidia	2	2	10823.5	656102.1	40
7215	line*coccidia	2	3	27439.5	656102.1	40
7218	line	1		19915.08	5190.828	40
7218	line	2		1.72E-12	5190.828	40
7218	coccidia		0	1.82E-12	7340.939	40
7218	coccidia		1	39830.17	7340.939	40
7218	coccidia		2	3.64E-12	7340.939	40
7218	coccidia		3	3.64E-12	7340.939	40
7218	line*coccidia	1	0	1.82E-12	10381.66	40
7218	line*coccidia	1	1	79660.33	10381.66	40
7218	line*coccidia	1	2	3.16E-12	10381.66	40

7218	line*coccidia	1	3	3.16E-12	10381.66	40
7218	line*coccidia	2	0	1.82E-12	10381.66	40
7218	line*coccidia	2	1	-3.2E-12	10381.66	40
7218	line*coccidia	2	2	4.11E-12	10381.66	40
7218	line*coccidia	2	3	4.11E-12	10381.66	40
7219	line	1		37799.04	14010.03	40
7219	line	2		-1E-12	14010.03	40
7219	coccidia		0	0	19813.18	40
7219	coccidia		1	75598.08	19813.18	40
7219	coccidia		2	0	19813.18	40
7219	coccidia		3	0	19813.18	40
7219	line*coccidia	1	0	0	28020.06	40
7219	line*coccidia	1	1	151196.2	28020.06	40
7219	line*coccidia	1	2	-3.5E-12	28020.06	40
7219	line*coccidia	1	3	-3.5E-12	28020.06	40
7219	line*coccidia	2	0	0	28020.06	40
7219	line*coccidia	2	1	-1.1E-11	28020.06	40
7219	line*coccidia	2	2	3.45E-12	28020.06	40
7219	line*coccidia	2	3	3.45E-12	28020.06	40
7220	line	1		56571.42	13338.36	40
7220	line	2		3.28E-12	13338.36	40
7220	coccidia		0	0	18863.29	40
7220	coccidia		1	113142.8	18863.29	40
7220	coccidia		2	4.85E-12	18863.29	40
7220	coccidia		3	1.46E-11	18863.29	40
7220	line*coccidia	1	0	0	26676.73	40
7220	line*coccidia	1	1	226285.7	26676.73	40
7220	line*coccidia	1	2	6.94E-12	26676.73	40
7220	line*coccidia	1	3	1.66E-11	26676.73	40
7220	line*coccidia	2	0	0	26676.73	40
7220	line*coccidia	2	1	-2.1E-12	26676.73	40
7220	line*coccidia	2	2	2.76E-12	26676.73	40
7220	line*coccidia	2	3	1.25E-11	26676.73	40
7221	line	1		73604.71	22492.64	40
7221	line	2		68515.92	22492.64	40
7221	coccidia		0	26323.58	31809.4	40
7221	coccidia		1	207628.3	31809.4	40
7221	coccidia		2	17791.5	31809.4	40
7221	coccidia		3	32497.83	31809.4	40
7221	line*coccidia	1	0	-1.8E-12	44985.29	40
7221	line*coccidia	1	1	294418.8	44985.29	40
7221	line*coccidia	1	2	-7.3E-12	44985.29	40
7221	line*coccidia	1	3	-7.3E-12	44985.29	40
7221	line*coccidia	2	0	52647.17	44985.29	40
7221	line*coccidia	2	1	120837.8	44985.29	40
7221	line*coccidia	2	2	35583	44985.29	40
7221	line*coccidia	2	3	64995.67	44985.29	40
7301	line	1		30743.33	5753.508	40
7301	line	2		8044.458	5753.508	40

7301	coccidia		0	47787.08	8136.689	40
7301	coccidia		1	2853.833	8136.689	40
7301	coccidia		2	4827.75	8136.689	40
7301	coccidia		3	22106.92	8136.689	40
7301	line*coccidia	1	0	73051.83	11507.02	40
7301	line*coccidia	1	1	5707.667	11507.02	40
7301	line*coccidia	1	2	-3.6E-12	11507.02	40
7301	line*coccidia	1	3	44213.83	11507.02	40
7301	line*coccidia	2	0	22522.33	11507.02	40
7301	line*coccidia	2	1	-3.6E-12	11507.02	40
7301	line*coccidia	2	2	9655.5	11507.02	40
7301	line*coccidia	2	3	-3.6E-12	11507.02	40
7302	line	1		11132.5	58881.7	40
7302	line	2		132045.5	58881.7	40
7302	coccidia		0	39874.42	83271.3	40
7302	coccidia		1	180514.7	83271.3	40
7302	coccidia		2	16224.5	83271.3	40
7302	coccidia		3	49742.33	83271.3	40
7302	line*coccidia	1	0	33784.5	117763.4	40
7302	line*coccidia	1	1	-1.5E-11	117763.4	40
7302	line*coccidia	1	2	3.64E-11	117763.4	40
7302	line*coccidia	1	3	10745.5	117763.4	40
7302	line*coccidia	2	0	45964.33	117763.4	40
7302	line*coccidia	2	1	361029.3	117763.4	40
7302	line*coccidia	2	2	32449	117763.4	40
7302	line*coccidia	2	3	88739.17	117763.4	40
7304	line	1		35779.63	19126.5	40
7304	line	2		43447.38	19126.5	40
7304	coccidia		0	18417.42	27048.95	40
7304	coccidia		1	101275.6	27048.95	40
7304	coccidia		2	5688.833	27048.95	40
7304	coccidia		3	33072.17	27048.95	40
7304	line*coccidia	1	0	15127.83	38252.99	40
7304	line*coccidia	1	1	90192.5	38252.99	40
7304	line*coccidia	1	2	-7.3E-12	38252.99	40
7304	line*coccidia	1	3	37798.17	38252.99	40
7304	line*coccidia	2	0	21707	38252.99	40
7304	line*coccidia	2	1	112358.7	38252.99	40
7304	line*coccidia	2	2	11377.67	38252.99	40
7304	line*coccidia	2	3	28346.17	38252.99	40
7305	line	1		132133.1	23261.54	40
7305	line	2		100846.2	23261.54	40
7305	coccidia		0	61137.67	32896.79	40
7305	coccidia		1	197045.2	32896.79	40
7305	coccidia		2	96572.75	32896.79	40
7305	coccidia		3	111203	32896.79	40
7305	line*coccidia	1	0	47200.17	46523.09	40
7305	line*coccidia	1	1	221861.3	46523.09	40
7305	line*coccidia	1	2	150853.7	46523.09	40

7305	line*coccidia	1	3	108617.2	46523.09	40
7305	line*coccidia	2	0	75075.17	46523.09	40
7305	line*coccidia	2	1	172229	46523.09	40
7305	line*coccidia	2	2	42291.83	46523.09	40
7305	line*coccidia	2	3	113788.8	46523.09	40
7306	line	1		84092.46	17432.75	40
7306	line	2		130174.6	17432.75	40
7306	coccidia		0	125472.2	24653.64	40
7306	coccidia		1	117581.1	24653.64	40
7306	coccidia		2	103638.7	24653.64	40
7306	coccidia		3	81842.17	24653.64	40
7306	line*coccidia	1	0	103936.5	34865.51	40
7306	line*coccidia	1	1	62898.33	34865.51	40
7306	line*coccidia	1	2	94425.67	34865.51	40
7306	line*coccidia	1	3	75109.33	34865.51	40
7306	line*coccidia	2	0	147007.8	34865.51	40
7306	line*coccidia	2	1	172263.8	34865.51	40
7306	line*coccidia	2	2	112851.7	34865.51	40
7306	line*coccidia	2	3	88575	34865.51	40
7307	line	1		663380.8	154210	40
7307	line	2		314522.4	154210	40
7307	coccidia		0	438454.4	218085.8	40
7307	coccidia		1	975900.7	218085.8	40
7307	coccidia		2	308546.9	218085.8	40
7307	coccidia		3	232904.4	218085.8	40
7307	line*coccidia	1	0	657448.7	308419.9	40
7307	line*coccidia	1	1	1175779	308419.9	40
7307	line*coccidia	1	2	472246	308419.9	40
7307	line*coccidia	1	3	348049.2	308419.9	40
7307	line*coccidia	2	0	219460.2	308419.9	40
7307	line*coccidia	2	1	776022	308419.9	40
7307	line*coccidia	2	2	144847.8	308419.9	40
7307	line*coccidia	2	3	117759.7	308419.9	40
7309	line	1		172633.9	141632.1	40
7309	line	2		287731.4	141632.1	40
7309	coccidia		0	105395.2	200298.1	40
7309	coccidia		1	658576.3	200298.1	40
7309	coccidia		2	57713.17	200298.1	40
7309	coccidia		3	99045.92	200298.1	40
7309	line*coccidia	1	0	41845.17	283264.3	40
7309	line*coccidia	1	1	425947.5	283264.3	40
7309	line*coccidia	1	2	85673.33	283264.3	40
7309	line*coccidia	1	3	137069.5	283264.3	40
7309	line*coccidia	2	0	168945.2	283264.3	40
7309	line*coccidia	2	1	891205	283264.3	40
7309	line*coccidia	2	2	29753	283264.3	40
7309	line*coccidia	2	3	61022.33	283264.3	40
7311	line	1		71482.25	10493.02	40
7311	line	2		103573.9	10493.02	40

7311	coccidia		0	99494	14839.37	40
7311	coccidia		1	123394.2	14839.37	40
7311	coccidia		2	44517.92	14839.37	40
7311	coccidia		3	82706.17	14839.37	40
7311	line*coccidia	1	0	101868.3	20986.05	40
7311	line*coccidia	1	1	53012.67	20986.05	40
7311	line*coccidia	1	2	27792.83	20986.05	40
7311	line*coccidia	1	3	103255.2	20986.05	40
7311	line*coccidia	2	0	97119.67	20986.05	40
7311	line*coccidia	2	1	193775.7	20986.05	40
7311	line*coccidia	2	2	61243	20986.05	40
7311	line*coccidia	2	3	62157.17	20986.05	40
7312	line	1		113531.5	11021.56	40
7312	line	2		153634.6	11021.56	40
7312	coccidia		0	143982.2	15586.84	40
7312	coccidia		1	127916.1	15586.84	40
7312	coccidia		2	99817.5	15586.84	40
7312	coccidia		3	162616.4	15586.84	40
7312	line*coccidia	1	0	137268.7	22043.12	40
7312	line*coccidia	1	1	82044.83	22043.12	40
7312	line*coccidia	1	2	43092.67	22043.12	40
7312	line*coccidia	1	3	191719.7	22043.12	40
7312	line*coccidia	2	0	150695.7	22043.12	40
7312	line*coccidia	2	1	173787.3	22043.12	40
7312	line*coccidia	2	2	156542.3	22043.12	40
7312	line*coccidia	2	3	133513.2	22043.12	40
7313	line	1		163912.6	35943.99	40
7313	line	2		118193	35943.99	40
7313	coccidia		0	238667.1	50832.48	40
7313	coccidia		1	88964.25	50832.48	40
7313	coccidia		2	100264	50832.48	40
7313	coccidia		3	136315.8	50832.48	40
7313	line*coccidia	1	0	281020	71887.99	40
7313	line*coccidia	1	1	91509.67	71887.99	40
7313	line*coccidia	1	2	102506.8	71887.99	40
7313	line*coccidia	1	3	180613.8	71887.99	40
7313	line*coccidia	2	0	196314.2	71887.99	40
7313	line*coccidia	2	1	86418.83	71887.99	40
7313	line*coccidia	2	2	98021.17	71887.99	40
7313	line*coccidia	2	3	92017.67	71887.99	40
7314	line	1		168518.3	38913.72	40
7314	line	2		117649.6	38913.72	40
7314	coccidia		0	150754.1	55032.31	40
7314	coccidia		1	243064.6	55032.31	40
7314	coccidia		2	100137.5	55032.31	40
7314	coccidia		3	78379.75	55032.31	40
7314	line*coccidia	1	0	195671.5	77827.44	40
7314	line*coccidia	1	1	230698	77827.44	40
7314	line*coccidia	1	2	134310.3	77827.44	40

7314	line*coccidia	1	3	113393.5	77827.44	40
7314	line*coccidia	2	0	105836.7	77827.44	40
7314	line*coccidia	2	1	255431.2	77827.44	40
7314	line*coccidia	2	2	65964.67	77827.44	40
7314	line*coccidia	2	3	43366	77827.44	40
7316	line	1		58593	15041.58	40
7316	line	2		86256.71	15041.58	40
7316	coccidia		0	93976.33	21272.01	40
7316	coccidia		1	75552	21272.01	40
7316	coccidia		2	53224.33	21272.01	40
7316	coccidia		3	66946.75	21272.01	40
7316	line*coccidia	1	0	100637.2	30083.17	40
7316	line*coccidia	1	1	22217.83	30083.17	40
7316	line*coccidia	1	2	21883.17	30083.17	40
7316	line*coccidia	1	3	89633.83	30083.17	40
7316	line*coccidia	2	0	87315.5	30083.17	40
7316	line*coccidia	2	1	128886.2	30083.17	40
7316	line*coccidia	2	2	84565.5	30083.17	40
7316	line*coccidia	2	3	44259.67	30083.17	40
7318	line	1		158928.2	16597.24	40
7318	line	2		148456.5	16597.24	40
7318	coccidia		0	170633.3	23472.04	40
7318	coccidia		1	132088	23472.04	40
7318	coccidia		2	97149.42	23472.04	40
7318	coccidia		3	214898.7	23472.04	40
7318	line*coccidia	1	0	220786.5	33194.48	40
7318	line*coccidia	1	1	140364.3	33194.48	40
7318	line*coccidia	1	2	55522.33	33194.48	40
7318	line*coccidia	1	3	219039.5	33194.48	40
7318	line*coccidia	2	0	120480	33194.48	40
7318	line*coccidia	2	1	123811.7	33194.48	40
7318	line*coccidia	2	2	138776.5	33194.48	40
7318	line*coccidia	2	3	210757.8	33194.48	40
7319	line	1		165211.3	39588.47	40
7319	line	2		234635.5	39588.47	40
7319	coccidia		0	180217.8	55986.54	40
7319	coccidia		1	346098.2	55986.54	40
7319	coccidia		2	129258.4	55986.54	40
7319	coccidia		3	144119.2	55986.54	40
7319	line*coccidia	1	0	142191.8	79176.93	40
7319	line*coccidia	1	1	236559.2	79176.93	40
7319	line*coccidia	1	2	86217	79176.93	40
7319	line*coccidia	1	3	195877.2	79176.93	40
7319	line*coccidia	2	0	218243.7	79176.93	40
7319	line*coccidia	2	1	455637.2	79176.93	40
7319	line*coccidia	2	2	172299.8	79176.93	40
7319	line*coccidia	2	3	92361.17	79176.93	40
7322	line	1		309391.3	53613.76	40
7322	line	2		69902.29	53613.76	40

7322	coccidia		0	103663.8	75821.3	40
7322	coccidia		1	348662.5	75821.3	40
7322	coccidia		2	214107.3	75821.3	40
7322	coccidia		3	92153.75	75821.3	40
7322	line*coccidia	1	0	143526.2	107227.5	40
7322	line*coccidia	1	1	611666.7	107227.5	40
7322	line*coccidia	1	2	373642.2	107227.5	40
7322	line*coccidia	1	3	108730.3	107227.5	40
7322	line*coccidia	2	0	63801.33	107227.5	40
7322	line*coccidia	2	1	85658.33	107227.5	40
7322	line*coccidia	2	2	54572.33	107227.5	40
7322	line*coccidia	2	3	75577.17	107227.5	40
7327	line	1		41227.63	10534.09	40
7327	line	2		38285.25	10534.09	40
7327	coccidia		0	54487.67	14897.46	40
7327	coccidia		1	27968	14897.46	40
7327	coccidia		2	34349.92	14897.46	40
7327	coccidia		3	42220.17	14897.46	40
7327	line*coccidia	1	0	24534.17	21068.19	40
7327	line*coccidia	1	1	55936	21068.19	40
7327	line*coccidia	1	2	7.28E-12	21068.19	40
7327	line*coccidia	1	3	84440.33	21068.19	40
7327	line*coccidia	2	0	84441.17	21068.19	40
7327	line*coccidia	2	1	7.28E-12	21068.19	40
7327	line*coccidia	2	2	68699.83	21068.19	40
7327	line*coccidia	2	3	7.28E-12	21068.19	40
7328	line	1		143778.1	33180.16	40
7328	line	2		-1.8E-12	33180.16	40
7328	coccidia		0	0	46923.83	40
7328	coccidia		1	171678.9	46923.83	40
7328	coccidia		2	115877.3	46923.83	40
7328	coccidia		3	0	46923.83	40
7328	line*coccidia	1	0	0	66360.31	40
7328	line*coccidia	1	1	343357.8	66360.31	40
7328	line*coccidia	1	2	231754.7	66360.31	40
7328	line*coccidia	1	3	0	66360.31	40
7328	line*coccidia	2	0	0	66360.31	40
7328	line*coccidia	2	1	-7.3E-12	66360.31	40
7328	line*coccidia	2	2	0	66360.31	40
7328	line*coccidia	2	3	0	66360.31	40
7330	line	1		11331.71	7835.695	40
7330	line	2		46318.79	7835.695	40
7330	coccidia		0	32306	11081.35	40
7330	coccidia		1	10961.08	11081.35	40
7330	coccidia		2	12673.5	11081.35	40
7330	coccidia		3	59360.42	11081.35	40
7330	line*coccidia	1	0	18979.67	15671.39	40
7330	line*coccidia	1	1	7.28E-12	15671.39	40
7330	line*coccidia	1	2	1.46E-11	15671.39	40

7330	line*coccidia	1	3	26347.17	15671.39	40
7330	line*coccidia	2	0	45632.33	15671.39	40
7330	line*coccidia	2	1	21922.17	15671.39	40
7330	line*coccidia	2	2	25347	15671.39	40
7330	line*coccidia	2	3	92373.67	15671.39	40
7331	line	1		264356.7	68916.6	40
7331	line	2		56833.54	68916.6	40
7331	coccidia		0	95780.83	97462.78	40
7331	coccidia		1	430250.3	97462.78	40
7331	coccidia		2	35115.17	97462.78	40
7331	coccidia		3	81234.08	97462.78	40
7331	line*coccidia	1	0	122235	137833.2	40
7331	line*coccidia	1	1	799388.3	137833.2	40
7331	line*coccidia	1	2	24348.67	137833.2	40
7331	line*coccidia	1	3	111454.7	137833.2	40
7331	line*coccidia	2	0	69326.67	137833.2	40
7331	line*coccidia	2	1	61112.33	137833.2	40
7331	line*coccidia	2	2	45881.67	137833.2	40
7331	line*coccidia	2	3	51013.5	137833.2	40
7332	line	1		42636.58	13919.7	40
7332	line	2		-1.2E-12	13919.7	40
7332	coccidia		0	0	19685.43	40
7332	coccidia		1	85273.17	19685.43	40
7332	coccidia		2	0	19685.43	40
7332	coccidia		3	0	19685.43	40
7332	line*coccidia	1	0	0	27839.4	40
7332	line*coccidia	1	1	170546.3	27839.4	40
7332	line*coccidia	1	2	6.43E-12	27839.4	40
7332	line*coccidia	1	3	6.43E-12	27839.4	40
7332	line*coccidia	2	0	0	27839.4	40
7332	line*coccidia	2	1	8.12E-12	27839.4	40
7332	line*coccidia	2	2	-6.4E-12	27839.4	40
7332	line*coccidia	2	3	-6.4E-12	27839.4	40
7333	line	1		131251.1	21799.32	40
7333	line	2		6.82E-12	21799.32	40
7333	coccidia		0	60030.58	30828.89	40
7333	coccidia		1	138571	30828.89	40
7333	coccidia		2	18588.42	30828.89	40
7333	coccidia		3	45312.25	30828.89	40
7333	line*coccidia	1	0	120061.2	43598.63	40
7333	line*coccidia	1	1	277142	43598.63	40
7333	line*coccidia	1	2	37176.83	43598.63	40
7333	line*coccidia	1	3	90624.5	43598.63	40
7333	line*coccidia	2	0	7.28E-12	43598.63	40
7333	line*coccidia	2	1	2E-11	43598.63	40
7333	line*coccidia	2	2	3.64E-12	43598.63	40
7333	line*coccidia	2	3	-3.6E-12	43598.63	40
7401	line	1		84735.58	32856.46	40
7401	line	2		105296.9	32856.46	40

7401	coccidia		0	40322.75	46466.06	40
7401	coccidia		1	118758.3	46466.06	40
7401	coccidia		2	88603.17	46466.06	40
7401	coccidia		3	132380.8	46466.06	40
7401	line*coccidia	1	0	25256.17	65712.93	40
7401	line*coccidia	1	1	149745.5	65712.93	40
7401	line*coccidia	1	2	114611	65712.93	40
7401	line*coccidia	1	3	49329.67	65712.93	40
7401	line*coccidia	2	0	55389.33	65712.93	40
7401	line*coccidia	2	1	87771.17	65712.93	40
7401	line*coccidia	2	2	62595.33	65712.93	40
7401	line*coccidia	2	3	215431.8	65712.93	40
7402	line	1		1244294	474346.3	40
7402	line	2		526559.4	474346.3	40
7402	coccidia		0	378610.4	670826.9	40
7402	coccidia		1	1817772	670826.9	40
7402	coccidia		2	406821.3	670826.9	40
7402	coccidia		3	938504	670826.9	40
7402	line*coccidia	1	0	573003	948692.6	40
7402	line*coccidia	1	1	2986849	948692.6	40
7402	line*coccidia	1	2	434338.7	948692.6	40
7402	line*coccidia	1	3	982986.2	948692.6	40
7402	line*coccidia	2	0	184217.8	948692.6	40
7402	line*coccidia	2	1	648694	948692.6	40
7402	line*coccidia	2	2	379303.8	948692.6	40
7402	line*coccidia	2	3	894021.8	948692.6	40
7403	line	1		70077.79	15001.5	40
7403	line	2		20410.29	15001.5	40
7403	coccidia		0	76531.58	21215.33	40
7403	coccidia		1	41971.75	21215.33	40
7403	coccidia		2	26606.67	21215.33	40
7403	coccidia		3	35866.17	21215.33	40
7403	line*coccidia	1	0	124635.3	30003.01	40
7403	line*coccidia	1	1	83943.5	30003.01	40
7403	line*coccidia	1	2	3.64E-12	30003.01	40
7403	line*coccidia	1	3	71732.33	30003.01	40
7403	line*coccidia	2	0	28427.83	30003.01	40
7403	line*coccidia	2	1	3.64E-12	30003.01	40
7403	line*coccidia	2	2	53213.33	30003.01	40
7403	line*coccidia	2	3	-1.1E-11	30003.01	40
7404	line	1		2350013	192790.9	40
7404	line	2		2292183	192790.9	40
7404	coccidia		0	3168922	272647.5	40
7404	coccidia		1	1862720	272647.5	40
7404	coccidia		2	1328471	272647.5	40
7404	coccidia		3	2924279	272647.5	40
7404	line*coccidia	1	0	3923257	385581.9	40
7404	line*coccidia	1	1	936864.5	385581.9	40
7404	line*coccidia	1	2	612020.5	385581.9	40

7404	line*coccidia	1	3	3927910	385581.9	40
7404	line*coccidia	2	0	2414588	385581.9	40
7404	line*coccidia	2	1	2788576	385581.9	40
7404	line*coccidia	2	2	2044921	385581.9	40
7404	line*coccidia	2	3	1920648	385581.9	40
7407	line	1		80660.92	16231.69	40
7407	line	2		82448.46	16231.69	40
7407	coccidia		0	43376.92	22955.08	40
7407	coccidia		1	125661.2	22955.08	40
7407	coccidia		2	40287.5	22955.08	40
7407	coccidia		3	116893.2	22955.08	40
7407	line*coccidia	1	0	59096.33	32463.38	40
7407	line*coccidia	1	1	164255.5	32463.38	40
7407	line*coccidia	1	2	62253.5	32463.38	40
7407	line*coccidia	1	3	37038.33	32463.38	40
7407	line*coccidia	2	0	27657.5	32463.38	40
7407	line*coccidia	2	1	87066.83	32463.38	40
7407	line*coccidia	2	2	18321.5	32463.38	40
7407	line*coccidia	2	3	196748	32463.38	40
7409	line	1		580489.2	83985.19	40
7409	line	2		395822.3	83985.19	40
7409	coccidia		0	639008.6	118773	40
7409	coccidia		1	283567.5	118773	40
7409	coccidia		2	270924.8	118773	40
7409	coccidia		3	759122.3	118773	40
7409	line*coccidia	1	0	924448.5	167970.4	40
7409	line*coccidia	1	1	166225	167970.4	40
7409	line*coccidia	1	2	300061.8	167970.4	40
7409	line*coccidia	1	3	931221.5	167970.4	40
7409	line*coccidia	2	0	353568.7	167970.4	40
7409	line*coccidia	2	1	400910	167970.4	40
7409	line*coccidia	2	2	241787.7	167970.4	40
7409	line*coccidia	2	3	587023	167970.4	40
7411	line	1		110682.7	29884.31	40
7411	line	2		115574.3	29884.31	40
7411	coccidia		0	64177.5	42262.79	40
7411	coccidia		1	168174.1	42262.79	40
7411	coccidia		2	130969.8	42262.79	40
7411	coccidia		3	89192.67	42262.79	40
7411	line*coccidia	1	0	40812	59768.61	40
7411	line*coccidia	1	1	118282.2	59768.61	40
7411	line*coccidia	1	2	226642.3	59768.61	40
7411	line*coccidia	1	3	56994.17	59768.61	40
7411	line*coccidia	2	0	87543	59768.61	40
7411	line*coccidia	2	1	218066	59768.61	40
7411	line*coccidia	2	2	35297.17	59768.61	40
7411	line*coccidia	2	3	121391.2	59768.61	40
7413	line	1		673217.6	114137.3	40
7413	line	2		729041	114137.3	40

7413	coccidia		0	907042.3	161414.6	40
7413	coccidia		1	463631.6	161414.6	40
7413	coccidia		2	468407.5	161414.6	40
7413	coccidia		3	965435.8	161414.6	40
7413	line*coccidia	1	0	973892.7	228274.7	40
7413	line*coccidia	1	1	150586.5	228274.7	40
7413	line*coccidia	1	2	558353.2	228274.7	40
7413	line*coccidia	1	3	1010038	228274.7	40
7413	line*coccidia	2	0	840192	228274.7	40
7413	line*coccidia	2	1	776676.7	228274.7	40
7413	line*coccidia	2	2	378461.8	228274.7	40
7413	line*coccidia	2	3	920833.5	228274.7	40
7414	line	1		1692972	270706.7	40
7414	line	2		2166278	270706.7	40
7414	coccidia		0	3106290	382837	40
7414	coccidia		1	1360292	382837	40
7414	coccidia		2	1207013	382837	40
7414	coccidia		3	2044904	382837	40
7414	line*coccidia	1	0	3610196	541413.3	40
7414	line*coccidia	1	1	353922	541413.3	40
7414	line*coccidia	1	2	343206.5	541413.3	40
7414	line*coccidia	1	3	2464562	541413.3	40
7414	line*coccidia	2	0	2602384	541413.3	40
7414	line*coccidia	2	1	2366663	541413.3	40
7414	line*coccidia	2	2	2070819	541413.3	40
7414	line*coccidia	2	3	1625246	541413.3	40
7415	line	1		740083.8	194093.9	40
7415	line	2		251991.5	194093.9	40
7415	coccidia		0	351971.8	274490.2	40
7415	coccidia		1	1086537	274490.2	40
7415	coccidia		2	142493.4	274490.2	40
7415	coccidia		3	403148.7	274490.2	40
7415	line*coccidia	1	0	550904.8	388187.7	40
7415	line*coccidia	1	1	1938199	388187.7	40
7415	line*coccidia	1	2	122998.8	388187.7	40
7415	line*coccidia	1	3	348233.2	388187.7	40
7415	line*coccidia	2	0	153038.7	388187.7	40
7415	line*coccidia	2	1	234875	388187.7	40
7415	line*coccidia	2	2	161988	388187.7	40
7415	line*coccidia	2	3	458064.2	388187.7	40
7418	line	1		605032.7	260003.3	40
7418	line	2		16345.96	260003.3	40
7418	coccidia		0	13842.08	367700.1	40
7418	coccidia		1	1118813	367700.1	40
7418	coccidia		2	110102	367700.1	40
7418	coccidia		3	0	367700.1	40
7418	line*coccidia	1	0	0	520006.5	40
7418	line*coccidia	1	1	2237627	520006.5	40
7418	line*coccidia	1	2	182504.3	520006.5	40

7418	line*coccidia	1	3	9.46E-11	520006.5	40
7418	line*coccidia	2	0	27684.17	520006.5	40
7418	line*coccidia	2	1	2.18E-11	520006.5	40
7418	line*coccidia	2	2	37699.67	520006.5	40
7418	line*coccidia	2	3	-9.5E-11	520006.5	40
7422	line	1		186568.9	64373.99	40
7422	line	2		261387.5	64373.99	40
7422	coccidia		0	333418	91038.57	40
7422	coccidia		1	160415.2	91038.57	40
7422	coccidia		2	81385.33	91038.57	40
7422	coccidia		3	320694.3	91038.57	40
7422	line*coccidia	1	0	336233.8	128748	40
7422	line*coccidia	1	1	0	128748	40
7422	line*coccidia	1	2	0	128748	40
7422	line*coccidia	1	3	410041.7	128748	40
7422	line*coccidia	2	0	330602.2	128748	40
7422	line*coccidia	2	1	320830.3	128748	40
7422	line*coccidia	2	2	162770.7	128748	40
7422	line*coccidia	2	3	231346.8	128748	40
7423	line	1		632206.2	111308.8	40
7423	line	2		520398.2	111308.8	40
7423	coccidia		0	836353.3	157414.5	40
7423	coccidia		1	438211.1	157414.5	40
7423	coccidia		2	233528.1	157414.5	40
7423	coccidia		3	797116.3	157414.5	40
7423	line*coccidia	1	0	1076468	222617.7	40
7423	line*coccidia	1	1	196785.2	222617.7	40
7423	line*coccidia	1	2	136197.3	222617.7	40
7423	line*coccidia	1	3	1119375	222617.7	40
7423	line*coccidia	2	0	596239	222617.7	40
7423	line*coccidia	2	1	679637	222617.7	40
7423	line*coccidia	2	2	330858.8	222617.7	40
7423	line*coccidia	2	3	474857.8	222617.7	40
7430	line	1		1067851	157081.1	40
7430	line	2		1237712	157081.1	40
7430	coccidia		0	1612519	222146.2	40
7430	coccidia		1	887580.2	222146.2	40
7430	coccidia		2	774606.3	222146.2	40
7430	coccidia		3	1336421	222146.2	40
7430	line*coccidia	1	0	1603769	314162.1	40
7430	line*coccidia	1	1	604949.8	314162.1	40
7430	line*coccidia	1	2	244189.8	314162.1	40
7430	line*coccidia	1	3	1818497	314162.1	40
7430	line*coccidia	2	0	1621268	314162.1	40
7430	line*coccidia	2	1	1170211	314162.1	40
7430	line*coccidia	2	2	1305023	314162.1	40
7430	line*coccidia	2	3	854344.8	314162.1	40
7431	line	1		669546.5	122736.6	40
7431	line	2		732047.8	122736.6	40

7431	coccidia		0	919125.3	173575.8	40
7431	coccidia		1	808556.8	173575.8	40
7431	coccidia		2	308908.8	173575.8	40
7431	coccidia		3	766597.7	173575.8	40
7431	line*coccidia	1	0	964368.5	245473.3	40
7431	line*coccidia	1	1	704461.8	245473.3	40
7431	line*coccidia	1	2	64771.33	245473.3	40
7431	line*coccidia	1	3	944584.3	245473.3	40
7431	line*coccidia	2	0	873882	245473.3	40
7431	line*coccidia	2	1	912651.8	245473.3	40
7431	line*coccidia	2	2	553046.2	245473.3	40
7431	line*coccidia	2	3	588611	245473.3	40
7433	line	1		2900004	1077678	40
7433	line	2		3673688	1077678	40
7433	coccidia		0	3126835	1524067	40
7433	coccidia		1	2055177	1524067	40
7433	coccidia		2	1109251	1524067	40
7433	coccidia		3	6856122	1524067	40
7433	line*coccidia	1	0	3942026	2155356	40
7433	line*coccidia	1	1	609153.5	2155356	40
7433	line*coccidia	1	2	1632601	2155356	40
7433	line*coccidia	1	3	5416237	2155356	40
7433	line*coccidia	2	0	2311644	2155356	40
7433	line*coccidia	2	1	3501201	2155356	40
7433	line*coccidia	2	2	585902.3	2155356	40
7433	line*coccidia	2	3	8296006	2155356	40
7436	line	1		3203042	781778.3	40
7436	line	2		2683547	781778.3	40
7436	coccidia		0	4264523	1105601	40
7436	coccidia		1	1369328	1105601	40
7436	coccidia		2	1954090	1105601	40
7436	coccidia		3	4185236	1105601	40
7436	line*coccidia	1	0	6191409	1563557	40
7436	line*coccidia	1	1	1359216	1563557	40
7436	line*coccidia	1	2	1229389	1563557	40
7436	line*coccidia	1	3	4032154	1563557	40
7436	line*coccidia	2	0	2337637	1563557	40
7436	line*coccidia	2	1	1379440	1563557	40
7436	line*coccidia	2	2	2678792	1563557	40
7436	line*coccidia	2	3	4338318	1563557	40
7437	line	1		425545.5	153388.8	40
7437	line	2		693498.8	153388.8	40
7437	coccidia		0	734626.6	216924.5	40
7437	coccidia		1	348050.1	216924.5	40
7437	coccidia		2	235684.9	216924.5	40
7437	coccidia		3	919727.1	216924.5	40
7437	line*coccidia	1	0	599138.8	306777.6	40
7437	line*coccidia	1	1	327980.7	306777.6	40
7437	line*coccidia	1	2	161126.8	306777.6	40

7437	line*coccidia	1	3	613935.8	306777.6	40
7437	line*coccidia	2	0	870114.3	306777.6	40
7437	line*coccidia	2	1	368119.5	306777.6	40
7437	line*coccidia	2	2	310243	306777.6	40
7437	line*coccidia	2	3	1225518	306777.6	40
7501	line	1		639530.2	288276.8	40
7501	line	2		679218.2	288276.8	40
7501	coccidia		0	824093	407685	40
7501	coccidia		1	346057	407685	40
7501	coccidia		2	946610.1	407685	40
7501	coccidia		3	520736.7	407685	40
7501	line*coccidia	1	0	1290469	576553.7	40
7501	line*coccidia	1	1	357198	576553.7	40
7501	line*coccidia	1	2	0	576553.7	40
7501	line*coccidia	1	3	910453.5	576553.7	40
7501	line*coccidia	2	0	357716.7	576553.7	40
7501	line*coccidia	2	1	334916	576553.7	40
7501	line*coccidia	2	2	1893220	576553.7	40
7501	line*coccidia	2	3	131019.8	576553.7	40
7503	line	1		723008	134527.7	40
7503	line	2		1248783	134527.7	40
7503	coccidia		0	1565264	190251	40
7503	coccidia		1	401486.8	190251	40
7503	coccidia		2	671849.2	190251	40
7503	coccidia		3	1304982	190251	40
7503	line*coccidia	1	0	1485373	269055.5	40
7503	line*coccidia	1	1	-1.7E-10	269055.5	40
7503	line*coccidia	1	2	-2.3E-10	269055.5	40
7503	line*coccidia	1	3	1406659	269055.5	40
7503	line*coccidia	2	0	1645156	269055.5	40
7503	line*coccidia	2	1	802973.5	269055.5	40
7503	line*coccidia	2	2	1343698	269055.5	40
7503	line*coccidia	2	3	1203304	269055.5	40
7504	line	1		46892224	4496236	40
7504	line	2		54487296	4496236	40
7504	coccidia		0	61861715	6358638	40
7504	coccidia		1	40803116	6358638	40
7504	coccidia		2	47349073	6358638	40
7504	coccidia		3	52745136	6358638	40
7504	line*coccidia	1	0	55902884	8992472	40
7504	line*coccidia	1	1	31574381	8992472	40
7504	line*coccidia	1	2	40568221	8992472	40
7504	line*coccidia	1	3	59523410	8992472	40
7504	line*coccidia	2	0	67820547	8992472	40
7504	line*coccidia	2	1	50031851	8992472	40
7504	line*coccidia	2	2	54129924	8992472	40
7504	line*coccidia	2	3	45966863	8992472	40
7505	line	1		9976393	1562664	40
7505	line	2		7504146	1562664	40

7505	coccidia		0	13078213	2209941	40
7505	coccidia		1	3615028	2209941	40
7505	coccidia		2	7968306	2209941	40
7505	coccidia		3	10299531	2209941	40
7505	line*coccidia	1	0	20179916	3125329	40
7505	line*coccidia	1	1	4094940	3125329	40
7505	line*coccidia	1	2	6239397	3125329	40
7505	line*coccidia	1	3	9391319	3125329	40
7505	line*coccidia	2	0	5976511	3125329	40
7505	line*coccidia	2	1	3135116	3125329	40
7505	line*coccidia	2	2	9697216	3125329	40
7505	line*coccidia	2	3	11207743	3125329	40
7506	line	1		1259258	1585913	40
7506	line	2		3178315	1585913	40
7506	coccidia		0	515923.3	2242820	40
7506	coccidia		1	5532237	2242820	40
7506	coccidia		2	684553.4	2242820	40
7506	coccidia		3	2142432	2242820	40
7506	line*coccidia	1	0	516446.2	3171827	40
7506	line*coccidia	1	1	1048134	3171827	40
7506	line*coccidia	1	2	927216.8	3171827	40
7506	line*coccidia	1	3	2545236	3171827	40
7506	line*coccidia	2	0	515400.5	3171827	40
7506	line*coccidia	2	1	10016340	3171827	40
7506	line*coccidia	2	2	441890	3171827	40
7506	line*coccidia	2	3	1739628	3171827	40
7507	line	1		1003097	249661.5	40
7507	line	2		737623.4	249661.5	40
7507	coccidia		0	1359467	353074.7	40
7507	coccidia		1	840912.1	353074.7	40
7507	coccidia		2	307128.7	353074.7	40
7507	coccidia		3	973934.2	353074.7	40
7507	line*coccidia	1	0	1958882	499323.1	40
7507	line*coccidia	1	1	345645.8	499323.1	40
7507	line*coccidia	1	2	278674.5	499323.1	40
7507	line*coccidia	1	3	1429187	499323.1	40
7507	line*coccidia	2	0	760051.3	499323.1	40
7507	line*coccidia	2	1	1336178	499323.1	40
7507	line*coccidia	2	2	335582.8	499323.1	40
7507	line*coccidia	2	3	518681	499323.1	40
7508	line	1		3320602	352423.2	40
7508	line	2		2521397	352423.2	40
7508	coccidia		0	4362003	498401.7	40
7508	coccidia		1	2374120	498401.7	40
7508	coccidia		2	1800021	498401.7	40
7508	coccidia		3	3147854	498401.7	40
7508	line*coccidia	1	0	4522517	704846.4	40
7508	line*coccidia	1	1	2947431	704846.4	40
7508	line*coccidia	1	2	908621.7	704846.4	40

7508	line*coccidia	1	3	4903839	704846.4	40
7508	line*coccidia	2	0	4201490	704846.4	40
7508	line*coccidia	2	1	1800809	704846.4	40
7508	line*coccidia	2	2	2691420	704846.4	40
7508	line*coccidia	2	3	1391869	704846.4	40
7512	line	1		18290863	1426892	40
7512	line	2		24106517	1426892	40
7512	coccidia		0	23095199	2017930	40
7512	coccidia		1	20570819	2017930	40
7512	coccidia		2	17587155	2017930	40
7512	coccidia		3	23541588	2017930	40
7512	line*coccidia	1	0	22296379	2853785	40
7512	line*coccidia	1	1	11342909	2853785	40
7512	line*coccidia	1	2	12972757	2853785	40
7512	line*coccidia	1	3	26551408	2853785	40
7512	line*coccidia	2	0	23894018	2853785	40
7512	line*coccidia	2	1	29798730	2853785	40
7512	line*coccidia	2	2	22201554	2853785	40
7512	line*coccidia	2	3	20531767	2853785	40
7514	line	1		4927378	820973	40
7514	line	2		3757512	820973	40
7514	coccidia		0	4751538	1161031	40
7514	coccidia		1	3919328	1161031	40
7514	coccidia		2	2121207	1161031	40
7514	coccidia		3	6577705	1161031	40
7514	line*coccidia	1	0	4378920	1641946	40
7514	line*coccidia	1	1	4099872	1641946	40
7514	line*coccidia	1	2	1482511	1641946	40
7514	line*coccidia	1	3	9748208	1641946	40
7514	line*coccidia	2	0	5124157	1641946	40
7514	line*coccidia	2	1	3738785	1641946	40
7514	line*coccidia	2	2	2759904	1641946	40
7514	line*coccidia	2	3	3407203	1641946	40
7515	line	1		11478887	2203759	40
7515	line	2		9280696	2203759	40
7515	coccidia		0	13317914	3116586	40
7515	coccidia		1	4140789	3116586	40
7515	coccidia		2	10304869	3116586	40
7515	coccidia		3	13755594	3116586	40
7515	line*coccidia	1	0	19927226	4407518	40
7515	line*coccidia	1	1	1647440	4407518	40
7515	line*coccidia	1	2	9754458	4407518	40
7515	line*coccidia	1	3	14586423	4407518	40
7515	line*coccidia	2	0	6708603	4407518	40
7515	line*coccidia	2	1	6634138	4407518	40
7515	line*coccidia	2	2	10855279	4407518	40
7515	line*coccidia	2	3	12924765	4407518	40
7516	line	1		1612011	310268.6	40
7516	line	2		819621.3	310268.6	40

7516	coccidia		0	1261872	438786.1	40
7516	coccidia		1	756449.9	438786.1	40
7516	coccidia		2	632152.7	438786.1	40
7516	coccidia		3	2212791	438786.1	40
7516	line*coccidia	1	0	1699314	620537.2	40
7516	line*coccidia	1	1	799832.3	620537.2	40
7516	line*coccidia	1	2	707937.5	620537.2	40
7516	line*coccidia	1	3	3240961	620537.2	40
7516	line*coccidia	2	0	824429.2	620537.2	40
7516	line*coccidia	2	1	713067.5	620537.2	40
7516	line*coccidia	2	2	556367.8	620537.2	40
7516	line*coccidia	2	3	1184621	620537.2	40
7518	line	1		967351.1	154924.9	40
7518	line	2		637384.6	154924.9	40
7518	coccidia		0	759342.4	219096.9	40
7518	coccidia		1	614696.9	219096.9	40
7518	coccidia		2	198609.5	219096.9	40
7518	coccidia		3	1636823	219096.9	40
7518	line*coccidia	1	0	1015514	309849.8	40
7518	line*coccidia	1	1	516538.7	309849.8	40
7518	line*coccidia	1	2	193329.7	309849.8	40
7518	line*coccidia	1	3	2144022	309849.8	40
7518	line*coccidia	2	0	503170.7	309849.8	40
7518	line*coccidia	2	1	712855.2	309849.8	40
7518	line*coccidia	2	2	203889.3	309849.8	40
7518	line*coccidia	2	3	1129623	309849.8	40
7519	line	1		15241401	2865578	40
7519	line	2		12954999	2865578	40
7519	coccidia		0	12925511	4052539	40
7519	coccidia		1	5530895	4052539	40
7519	coccidia		2	13617038	4052539	40
7519	coccidia		3	24319355	4052539	40
7519	line*coccidia	1	0	12096815	5731156	40
7519	line*coccidia	1	1	4056103	5731156	40
7519	line*coccidia	1	2	11104069	5731156	40
7519	line*coccidia	1	3	33708617	5731156	40
7519	line*coccidia	2	0	13754207	5731156	40
7519	line*coccidia	2	1	7005687	5731156	40
7519	line*coccidia	2	2	16130008	5731156	40
7519	line*coccidia	2	3	14930094	5731156	40
7520	line	1		972128.8	846106	40
7520	line	2		4695110	846106	40
7520	coccidia		0	1514129	1196575	40
7520	coccidia		1	8266097	1196575	40
7520	coccidia		2	584637.8	1196575	40
7520	coccidia		3	969612.8	1196575	40
7520	line*coccidia	1	0	1131893	1692212	40
7520	line*coccidia	1	1	901891.5	1692212	40
7520	line*coccidia	1	2	558318	1692212	40

7520	line*coccidia	1	3	1296412	1692212	40
7520	line*coccidia	2	0	1896365	1692212	40
7520	line*coccidia	2	1	15630303	1692212	40
7520	line*coccidia	2	2	610957.5	1692212	40
7520	line*coccidia	2	3	642813.3	1692212	40
7521	line	1		7968983	1047054	40
7521	line	2		8468935	1047054	40
7521	coccidia		0	12575986	1480758	40
7521	coccidia		1	7914171	1480758	40
7521	coccidia		2	5134996	1480758	40
7521	coccidia		3	7250684	1480758	40
7521	line*coccidia	1	0	10803585	2094108	40
7521	line*coccidia	1	1	7575650	2094108	40
7521	line*coccidia	1	2	3557854	2094108	40
7521	line*coccidia	1	3	9938844	2094108	40
7521	line*coccidia	2	0	14348387	2094108	40
7521	line*coccidia	2	1	8252692	2094108	40
7521	line*coccidia	2	2	6712138	2094108	40
7521	line*coccidia	2	3	4562524	2094108	40
7522	line	1		11619329	2382607	40
7522	line	2		9808095	2382607	40
7522	coccidia		0	16906054	3369515	40
7522	coccidia		1	2382166	3369515	40
7522	coccidia		2	10955478	3369515	40
7522	coccidia		3	12611150	3369515	40
7522	line*coccidia	1	0	27441729	4765215	40
7522	line*coccidia	1	1	521397	4765215	40
7522	line*coccidia	1	2	4564872	4765215	40
7522	line*coccidia	1	3	13949319	4765215	40
7522	line*coccidia	2	0	6370379	4765215	40
7522	line*coccidia	2	1	4242935	4765215	40
7522	line*coccidia	2	2	17346083	4765215	40
7522	line*coccidia	2	3	11272981	4765215	40
7530	line	1		8651118	4238761	40
7530	line	2		2473774	4238761	40
7530	coccidia		0	2646534	5994514	40
7530	coccidia		1	1319447	5994514	40
7530	coccidia		2	1611356	5994514	40
7530	coccidia		3	16672445	5994514	40
7530	line*coccidia	1	0	3516341	8477523	40
7530	line*coccidia	1	1	412956.5	8477523	40
7530	line*coccidia	1	2	2078908	8477523	40
7530	line*coccidia	1	3	28596266	8477523	40
7530	line*coccidia	2	0	1776728	8477523	40
7530	line*coccidia	2	1	2225938	8477523	40
7530	line*coccidia	2	2	1143804	8477523	40
7530	line*coccidia	2	3	4748624	8477523	40
7543	line	1		7124818	1383850	40
7543	line	2		13877433	1383850	40

7543	coccidia		0	13389329	1957060	40
7543	coccidia		1	8932212	1957060	40
7543	coccidia		2	9632643	1957060	40
7543	coccidia		3	10050318	1957060	40
7543	line*coccidia	1	0	8878732	2767701	40
7543	line*coccidia	1	1	4137527	2767701	40
7543	line*coccidia	1	2	7614714	2767701	40
7543	line*coccidia	1	3	7868300	2767701	40
7543	line*coccidia	2	0	17899927	2767701	40
7543	line*coccidia	2	1	13726896	2767701	40
7543	line*coccidia	2	2	11650573	2767701	40
7543	line*coccidia	2	3	12232336	2767701	40
7544	line	1		253914.9	241137.5	40
7544	line	2		838220.4	241137.5	40
7544	coccidia		0	708758.9	341019.9	40
7544	coccidia		1	967026.9	341019.9	40
7544	coccidia		2	130984.6	341019.9	40
7544	coccidia		3	377500.2	341019.9	40
7544	line*coccidia	1	0	2.33E-10	482274.9	40
7544	line*coccidia	1	1	260659.3	482274.9	40
7544	line*coccidia	1	2	-8.7E-11	482274.9	40
7544	line*coccidia	1	3	755000.3	482274.9	40
7544	line*coccidia	2	0	1417518	482274.9	40
7544	line*coccidia	2	1	1673395	482274.9	40
7544	line*coccidia	2	2	261969.2	482274.9	40
7544	line*coccidia	2	3	1.16E-10	482274.9	40
7546	line	1		12537699	3297779	40
7546	line	2		19635751	3297779	40
7546	coccidia		0	15376170	4663764	40
7546	coccidia		1	15744767	4663764	40
7546	coccidia		2	14063666	4663764	40
7546	coccidia		3	19162296	4663764	40
7546	line*coccidia	1	0	7724584	6595558	40
7546	line*coccidia	1	1	3188916	6595558	40
7546	line*coccidia	1	2	17271157	6595558	40
7546	line*coccidia	1	3	21966138	6595558	40
7546	line*coccidia	2	0	23027757	6595558	40
7546	line*coccidia	2	1	28300618	6595558	40
7546	line*coccidia	2	2	10856174	6595558	40
7546	line*coccidia	2	3	16358455	6595558	40
7549	line	1		8376494	1338740	40
7549	line	2		10347279	1338740	40
7549	coccidia		0	11275602	1893264	40
7549	coccidia		1	10664718	1893264	40
7549	coccidia		2	4497817	1893264	40
7549	coccidia		3	11009411	1893264	40
7549	line*coccidia	1	0	7342842	2677480	40
7549	line*coccidia	1	1	6332674	2677480	40
7549	line*coccidia	1	2	2150099	2677480	40

7549	line*coccidia	1	3	17680364	2677480	40
7549	line*coccidia	2	0	15208362	2677480	40
7549	line*coccidia	2	1	14996762	2677480	40
7549	line*coccidia	2	2	6845534	2677480	40
7549	line*coccidia	2	3	4338459	2677480	40
7551	line	1		9558447	2419178	40
7551	line	2		13377412	2419178	40
7551	coccidia		0	19582371	3421235	40
7551	coccidia		1	7674696	3421235	40
7551	coccidia		2	7393239	3421235	40
7551	coccidia		3	11221413	3421235	40
7551	line*coccidia	1	0	8467403	4838356	40
7551	line*coccidia	1	1	5382042	4838356	40
7551	line*coccidia	1	2	7776039	4838356	40
7551	line*coccidia	1	3	16608306	4838356	40
7551	line*coccidia	2	0	30697339	4838356	40
7551	line*coccidia	2	1	9967349	4838356	40
7551	line*coccidia	2	2	7010439	4838356	40
7551	line*coccidia	2	3	5834521	4838356	40
7601	line	1		2222696	326431.1	40
7601	line	2		2252649	326431.1	40
7601	coccidia		0	2381103	461643.3	40
7601	coccidia		1	1846921	461643.3	40
7601	coccidia		2	1247989	461643.3	40
7601	coccidia		3	3474678	461643.3	40
7601	line*coccidia	1	0	2047670	652862.2	40
7601	line*coccidia	1	1	545012	652862.2	40
7601	line*coccidia	1	2	1079814	652862.2	40
7601	line*coccidia	1	3	5218289	652862.2	40
7601	line*coccidia	2	0	2714536	652862.2	40
7601	line*coccidia	2	1	3148830	652862.2	40
7601	line*coccidia	2	2	1416164	652862.2	40
7601	line*coccidia	2	3	1731068	652862.2	40
7604	line	1		4413835	365899.9	40
7604	line	2		4155917	365899.9	40
7604	coccidia		0	5943564	517460.6	40
7604	coccidia		1	3111371	517460.6	40
7604	coccidia		2	3260819	517460.6	40
7604	coccidia		3	4823749	517460.6	40
7604	line*coccidia	1	0	6170690	731799.9	40
7604	line*coccidia	1	1	3227992	731799.9	40
7604	line*coccidia	1	2	2704134	731799.9	40
7604	line*coccidia	1	3	5552525	731799.9	40
7604	line*coccidia	2	0	5716439	731799.9	40
7604	line*coccidia	2	1	2994750	731799.9	40
7604	line*coccidia	2	2	3817505	731799.9	40
7604	line*coccidia	2	3	4094973	731799.9	40
7608	line	1		3665002	295032.8	40
7608	line	2		3072607	295032.8	40

7608	coccidia		0	4214467	417239.4	40
7608	coccidia		1	2881007	417239.4	40
7608	coccidia		2	2359487	417239.4	40
7608	coccidia		3	4020258	417239.4	40
7608	line*coccidia	1	0	4851392	590065.6	40
7608	line*coccidia	1	1	2806515	590065.6	40
7608	line*coccidia	1	2	2030678	590065.6	40
7608	line*coccidia	1	3	4971424	590065.6	40
7608	line*coccidia	2	0	3577542	590065.6	40
7608	line*coccidia	2	1	2955500	590065.6	40
7608	line*coccidia	2	2	2688296	590065.6	40
7608	line*coccidia	2	3	3069092	590065.6	40
7615	line	1		2032573	471144.8	40
7615	line	2		4103947	471144.8	40
7615	coccidia		0	2887627	666299.4	40
7615	coccidia		1	3811717	666299.4	40
7615	coccidia		2	1702215	666299.4	40
7615	coccidia		3	3871481	666299.4	40
7615	line*coccidia	1	0	1579540	942289.7	40
7615	line*coccidia	1	1	549268	942289.7	40
7615	line*coccidia	1	2	1010730	942289.7	40
7615	line*coccidia	1	3	4990754	942289.7	40
7615	line*coccidia	2	0	4195714	942289.7	40
7615	line*coccidia	2	1	7074166	942289.7	40
7615	line*coccidia	2	2	2393700	942289.7	40
7615	line*coccidia	2	3	2752208	942289.7	40
7622	line	1		2204132	769240.8	40
7622	line	2		3862426	769240.8	40
7622	coccidia		0	4596028	1087871	40
7622	coccidia		1	3172012	1087871	40
7622	coccidia		2	1425947	1087871	40
7622	coccidia		3	2939128	1087871	40
7622	line*coccidia	1	0	1706401	1538482	40
7622	line*coccidia	1	1	1196740	1538482	40
7622	line*coccidia	1	2	1410243	1538482	40
7622	line*coccidia	1	3	4503146	1538482	40
7622	line*coccidia	2	0	7485656	1538482	40
7622	line*coccidia	2	1	5147285	1538482	40
7622	line*coccidia	2	2	1441651	1538482	40
7622	line*coccidia	2	3	1375111	1538482	40
7632	line	1		2515555	479703.2	40
7632	line	2		3704840	479703.2	40
7632	coccidia		0	4220001	678402.8	40
7632	coccidia		1	3991991	678402.8	40
7632	coccidia		2	1594915	678402.8	40
7632	coccidia		3	2633883	678402.8	40
7632	line*coccidia	1	0	3681250	959406.4	40
7632	line*coccidia	1	1	2100157	959406.4	40
7632	line*coccidia	1	2	1313573	959406.4	40

7632	line*coccidia	1	3	2967239	959406.4	40
7632	line*coccidia	2	0	4758753	959406.4	40
7632	line*coccidia	2	1	5883824	959406.4	40
7632	line*coccidia	2	2	1876256	959406.4	40
7632	line*coccidia	2	3	2300528	959406.4	40
7635	line	1		1160375	358665.4	40
7635	line	2		2417242	358665.4	40
7635	coccidia		0	2334266	507229.4	40
7635	coccidia		1	2279328	507229.4	40
7635	coccidia		2	902060.8	507229.4	40
7635	coccidia		3	1639580	507229.4	40
7635	line*coccidia	1	0	1407911	717330.7	40
7635	line*coccidia	1	1	288157.7	717330.7	40
7635	line*coccidia	1	2	640958.2	717330.7	40
7635	line*coccidia	1	3	2304474	717330.7	40
7635	line*coccidia	2	0	3260622	717330.7	40
7635	line*coccidia	2	1	4270499	717330.7	40
7635	line*coccidia	2	2	1163164	717330.7	40
7635	line*coccidia	2	3	974685	717330.7	40
7703	line	1		412731.1	141498.5	40
7703	line	2		796550.8	141498.5	40
7703	coccidia		0	907308.2	200109.1	40
7703	coccidia		1	411438.1	200109.1	40
7703	coccidia		2	362734.2	200109.1	40
7703	coccidia		3	737083.5	200109.1	40
7703	line*coccidia	1	0	359619.3	282997.1	40
7703	line*coccidia	1	1	93629.67	282997.1	40
7703	line*coccidia	1	2	278477.8	282997.1	40
7703	line*coccidia	1	3	919197.7	282997.1	40
7703	line*coccidia	2	0	1454997	282997.1	40
7703	line*coccidia	2	1	729246.5	282997.1	40
7703	line*coccidia	2	2	446990.5	282997.1	40
7703	line*coccidia	2	3	554969.3	282997.1	40
7706	line	1		861642.3	186102.5	40
7706	line	2		891778.8	186102.5	40
7706	coccidia		0	805062.1	263188.7	40
7706	coccidia		1	855721.7	263188.7	40
7706	coccidia		2	637769.8	263188.7	40
7706	coccidia		3	1208289	263188.7	40
7706	line*coccidia	1	0	485232.7	372205	40
7706	line*coccidia	1	1	494081.3	372205	40
7706	line*coccidia	1	2	907443.7	372205	40
7706	line*coccidia	1	3	1559812	372205	40
7706	line*coccidia	2	0	1124892	372205	40
7706	line*coccidia	2	1	1217362	372205	40
7706	line*coccidia	2	2	368096	372205	40
7706	line*coccidia	2	3	856765.5	372205	40
7709	line	1		647442.5	269564.5	40
7709	line	2		638834.4	269564.5	40

7709	coccidia		0	814515.4	381221.7	40
7709	coccidia		1	156468.6	381221.7	40
7709	coccidia		2	357572.9	381221.7	40
7709	coccidia		3	1243997	381221.7	40
7709	line*coccidia	1	0	496985.8	539128.9	40
7709	line*coccidia	1	1	31362.83	539128.9	40
7709	line*coccidia	1	2	168449.7	539128.9	40
7709	line*coccidia	1	3	1892972	539128.9	40
7709	line*coccidia	2	0	1132045	539128.9	40
7709	line*coccidia	2	1	281574.3	539128.9	40
7709	line*coccidia	2	2	546696.2	539128.9	40
7709	line*coccidia	2	3	595022.2	539128.9	40
7711	line	1		581941.6	299242.3	40
7711	line	2		1391381	299242.3	40
7711	coccidia		0	905954.5	423192.5	40
7711	coccidia		1	1666107	423192.5	40
7711	coccidia		2	450333.4	423192.5	40
7711	coccidia		3	924250.8	423192.5	40
7711	line*coccidia	1	0	424045.5	598484.6	40
7711	line*coccidia	1	1	202497	598484.6	40
7711	line*coccidia	1	2	485926.7	598484.6	40
7711	line*coccidia	1	3	1215297	598484.6	40
7711	line*coccidia	2	0	1387864	598484.6	40
7711	line*coccidia	2	1	3129716	598484.6	40
7711	line*coccidia	2	2	414740.2	598484.6	40
7711	line*coccidia	2	3	633204.5	598484.6	40
7715	line	1		719998.3	100757.2	40
7715	line	2		905794	100757.2	40
7715	coccidia		0	900374.4	142492.1	40
7715	coccidia		1	1361307	142492.1	40
7715	coccidia		2	452880	142492.1	40
7715	coccidia		3	537023.4	142492.1	40
7715	line*coccidia	1	0	594173.7	201514.3	40
7715	line*coccidia	1	1	1230491	201514.3	40
7715	line*coccidia	1	2	342567.2	201514.3	40
7715	line*coccidia	1	3	712761.3	201514.3	40
7715	line*coccidia	2	0	1206575	201514.3	40
7715	line*coccidia	2	1	1492123	201514.3	40
7715	line*coccidia	2	2	563192.8	201514.3	40
7715	line*coccidia	2	3	361285.5	201514.3	40
7724	line	1		2206529	572105	40
7724	line	2		4237210	572105	40
7724	coccidia		0	5241514	809078.7	40
7724	coccidia		1	3325495	809078.7	40
7724	coccidia		2	1869489	809078.7	40
7724	coccidia		3	2450979	809078.7	40
7724	line*coccidia	1	0	3592384	1144210	40
7724	line*coccidia	1	1	1641096	1144210	40
7724	line*coccidia	1	2	687455.3	1144210	40

7724	line*coccidia	1	3	2905179	1144210	40
7724	line*coccidia	2	0	6890644	1144210	40
7724	line*coccidia	2	1	5009894	1144210	40
7724	line*coccidia	2	2	3051523	1144210	40
7724	line*coccidia	2	3	1996779	1144210	40
7725	line	1		2406933	514204.5	40
7725	line	2		3505060	514204.5	40
7725	coccidia		0	4416280	727194.9	40
7725	coccidia		1	3624808	727194.9	40
7725	coccidia		2	1072075	727194.9	40
7725	coccidia		3	2710823	727194.9	40
7725	line*coccidia	1	0	3109736	1028409	40
7725	line*coccidia	1	1	2428829	1028409	40
7725	line*coccidia	1	2	605774.5	1028409	40
7725	line*coccidia	1	3	3483392	1028409	40
7725	line*coccidia	2	0	5722824	1028409	40
7725	line*coccidia	2	1	4820787	1028409	40
7725	line*coccidia	2	2	1538376	1028409	40
7725	line*coccidia	2	3	1938255	1028409	40
7726	line	1		2312251	293527.7	40
7726	line	2		3005612	293527.7	40
7726	coccidia		0	3551906	415110.9	40
7726	coccidia		1	2974621	415110.9	40
7726	coccidia		2	1453696	415110.9	40
7726	coccidia		3	2655501	415110.9	40
7726	line*coccidia	1	0	2890206	587055.5	40
7726	line*coccidia	1	1	2330312	587055.5	40
7726	line*coccidia	1	2	692586	587055.5	40
7726	line*coccidia	1	3	3335898	587055.5	40
7726	line*coccidia	2	0	4213607	587055.5	40
7726	line*coccidia	2	1	3618930	587055.5	40
7726	line*coccidia	2	2	2214807	587055.5	40
7726	line*coccidia	2	3	1975104	587055.5	40
7727	line	1		1387170	270879	40
7727	line	2		1865905	270879	40
7727	coccidia		0	2033699	383080.7	40
7727	coccidia		1	2518679	383080.7	40
7727	coccidia		2	564097.7	383080.7	40
7727	coccidia		3	1389674	383080.7	40
7727	line*coccidia	1	0	983212.7	541758	40
7727	line*coccidia	1	1	2259835	541758	40
7727	line*coccidia	1	2	356841.7	541758	40
7727	line*coccidia	1	3	1948792	541758	40
7727	line*coccidia	2	0	3084186	541758	40
7727	line*coccidia	2	1	2777523	541758	40
7727	line*coccidia	2	2	771353.7	541758	40
7727	line*coccidia	2	3	830556.5	541758	40
7728	line	1		539697.3	146020.5	40
7728	line	2		905095.7	146020.5	40

7728	coccidia		0	833049.7	206504.2	40
7728	coccidia		1	570900	206504.2	40
7728	coccidia		2	833168.2	206504.2	40
7728	coccidia		3	652468	206504.2	40
7728	line*coccidia	1	0	552028.7	292041	40
7728	line*coccidia	1	1	227396.2	292041	40
7728	line*coccidia	1	2	543321.8	292041	40
7728	line*coccidia	1	3	836042.3	292041	40
7728	line*coccidia	2	0	1114071	292041	40
7728	line*coccidia	2	1	914403.8	292041	40
7728	line*coccidia	2	2	1123015	292041	40
7728	line*coccidia	2	3	468893.7	292041	40
7732	line	1		576911.1	154125.9	40
7732	line	2		898023.2	154125.9	40
7732	coccidia		0	636868.8	217967	40
7732	coccidia		1	730465.4	217967	40
7732	coccidia		2	665113.2	217967	40
7732	coccidia		3	917421.3	217967	40
7732	line*coccidia	1	0	482217.5	308251.9	40
7732	line*coccidia	1	1	89916	308251.9	40
7732	line*coccidia	1	2	329843.8	308251.9	40
7732	line*coccidia	1	3	1405667	308251.9	40
7732	line*coccidia	2	0	791520.2	308251.9	40
7732	line*coccidia	2	1	1371015	308251.9	40
7732	line*coccidia	2	2	1000383	308251.9	40
7732	line*coccidia	2	3	429175.3	308251.9	40
7733	line	1		1240698	563272.7	40
7733	line	2		1719273	563272.7	40
7733	coccidia		0	1141004	796588	40
7733	coccidia		1	1161662	796588	40
7733	coccidia		2	715835.1	796588	40
7733	coccidia		3	2901441	796588	40
7733	line*coccidia	1	0	537352.5	1126545	40
7733	line*coccidia	1	1	157121.3	1126545	40
7733	line*coccidia	1	2	641995	1126545	40
7733	line*coccidia	1	3	3626324	1126545	40
7733	line*coccidia	2	0	1744655	1126545	40
7733	line*coccidia	2	1	2166203	1126545	40
7733	line*coccidia	2	2	789675.2	1126545	40
7733	line*coccidia	2	3	2176558	1126545	40
7736	line	1		779801.8	512763.3	40
7736	line	2		3277409	512763.3	40
7736	coccidia		0	3779203	725156.9	40
7736	coccidia		1	698296.7	725156.9	40
7736	coccidia		2	1597834	725156.9	40
7736	coccidia		3	2039089	725156.9	40
7736	line*coccidia	1	0	705602.7	1025527	40
7736	line*coccidia	1	1	97895.17	1025527	40
7736	line*coccidia	1	2	1621160	1025527	40

7736	line*coccidia	1	3	694549.5	1025527	40
7736	line*coccidia	2	0	6852802	1025527	40
7736	line*coccidia	2	1	1298698	1025527	40
7736	line*coccidia	2	2	1574508	1025527	40
7736	line*coccidia	2	3	3383629	1025527	40
7737	line	1		1670036	826502.7	40
7737	line	2		1597325	826502.7	40
7737	coccidia		0	1494419	1168851	40
7737	coccidia		1	1043029	1168851	40
7737	coccidia		2	1177267	1168851	40
7737	coccidia		3	2820007	1168851	40
7737	line*coccidia	1	0	444596.5	1653005	40
7737	line*coccidia	1	1	87163.83	1653005	40
7737	line*coccidia	1	2	1047160	1653005	40
7737	line*coccidia	1	3	5101224	1653005	40
7737	line*coccidia	2	0	2544241	1653005	40
7737	line*coccidia	2	1	1998894	1653005	40
7737	line*coccidia	2	2	1307374	1653005	40
7737	line*coccidia	2	3	538789.7	1653005	40
7739	line	1		887379.4	411174.7	40
7739	line	2		2256333	411174.7	40
7739	coccidia		0	1717917	581488.8	40
7739	coccidia		1	902311.2	581488.8	40
7739	coccidia		2	2435428	581488.8	40
7739	coccidia		3	1231769	581488.8	40
7739	line*coccidia	1	0	838272	822349.3	40
7739	line*coccidia	1	1	110960	822349.3	40
7739	line*coccidia	1	2	1185124	822349.3	40
7739	line*coccidia	1	3	1415161	822349.3	40
7739	line*coccidia	2	0	2597561	822349.3	40
7739	line*coccidia	2	1	1693662	822349.3	40
7739	line*coccidia	2	2	3685732	822349.3	40
7739	line*coccidia	2	3	1048376	822349.3	40
7743	line	1		178799.9	41078.14	40
7743	line	2		188762.8	41078.14	40
7743	coccidia		0	109166.3	58093.26	40
7743	coccidia		1	510259.8	58093.26	40
7743	coccidia		2	34784.5	58093.26	40
7743	coccidia		3	80914.75	58093.26	40
7743	line*coccidia	1	0	83296.33	82156.28	40
7743	line*coccidia	1	1	486164.7	82156.28	40
7743	line*coccidia	1	2	38913.17	82156.28	40
7743	line*coccidia	1	3	106825.3	82156.28	40
7743	line*coccidia	2	0	135036.3	82156.28	40
7743	line*coccidia	2	1	534355	82156.28	40
7743	line*coccidia	2	2	30655.83	82156.28	40
7743	line*coccidia	2	3	55004.17	82156.28	40
7801	line	1		1.09E-11	66322.08	40
7801	line	2		497294.8	66322.08	40

7801	coccidia		0	181469.6	93793.58	40
7801	coccidia		1	425310.6	93793.58	40
7801	coccidia		2	151987.3	93793.58	40
7801	coccidia		3	235822.1	93793.58	40
7801	line*coccidia	1	0	1.46E-11	132644.2	40
7801	line*coccidia	1	1	0	132644.2	40
7801	line*coccidia	1	2	2.91E-11	132644.2	40
7801	line*coccidia	1	3	0	132644.2	40
7801	line*coccidia	2	0	362939.2	132644.2	40
7801	line*coccidia	2	1	850621.2	132644.2	40
7801	line*coccidia	2	2	303974.5	132644.2	40
7801	line*coccidia	2	3	471644.2	132644.2	40
7804	line	1		475869.5	114944	40
7804	line	2		688772.2	114944	40
7804	coccidia		0	578958.8	162555.4	40
7804	coccidia		1	520698	162555.4	40
7804	coccidia		2	678113.1	162555.4	40
7804	coccidia		3	551513.6	162555.4	40
7804	line*coccidia	1	0	432560.5	229888.1	40
7804	line*coccidia	1	1	317710.2	229888.1	40
7804	line*coccidia	1	2	350044.8	229888.1	40
7804	line*coccidia	1	3	803162.7	229888.1	40
7804	line*coccidia	2	0	725357.2	229888.1	40
7804	line*coccidia	2	1	723685.8	229888.1	40
7804	line*coccidia	2	2	1006181	229888.1	40
7804	line*coccidia	2	3	299864.5	229888.1	40
7805	line	1		1.24E-10	80514.61	40
7805	line	2		660064.1	80514.61	40
7805	coccidia		0	234164.3	113864.9	40
7805	coccidia		1	596033.3	113864.9	40
7805	coccidia		2	187096.4	113864.9	40
7805	coccidia		3	302834.2	113864.9	40
7805	line*coccidia	1	0	1.46E-10	161029.2	40
7805	line*coccidia	1	1	1.16E-10	161029.2	40
7805	line*coccidia	1	2	1.16E-10	161029.2	40
7805	line*coccidia	1	3	1.16E-10	161029.2	40
7805	line*coccidia	2	0	468328.7	161029.2	40
7805	line*coccidia	2	1	1192067	161029.2	40
7805	line*coccidia	2	2	374192.8	161029.2	40
7805	line*coccidia	2	3	605668.3	161029.2	40
7807	line	1		388653.7	232341.7	40
7807	line	2		1500844	232341.7	40
7807	coccidia		0	872358.5	328580.8	40
7807	coccidia		1	1430204	328580.8	40
7807	coccidia		2	873774.9	328580.8	40
7807	coccidia		3	602658.8	328580.8	40
7807	line*coccidia	1	0	120051.8	464683.5	40
7807	line*coccidia	1	1	73491.33	464683.5	40
7807	line*coccidia	1	2	511335	464683.5	40

7807	line*coccidia	1	3	849736.5	464683.5	40
7807	line*coccidia	2	0	1624665	464683.5	40
7807	line*coccidia	2	1	2786916	464683.5	40
7807	line*coccidia	2	2	1236215	464683.5	40
7807	line*coccidia	2	3	355581	464683.5	40
7811	line	1		923932.3	359678.4	40
7811	line	2		1026960	359678.4	40
7811	coccidia		0	498089	508662.1	40
7811	coccidia		1	1327620	508662.1	40
7811	coccidia		2	1390758	508662.1	40
7811	coccidia		3	685318.4	508662.1	40
7811	line*coccidia	1	0	436170.2	719356.9	40
7811	line*coccidia	1	1	48499.33	719356.9	40
7811	line*coccidia	1	2	2314181	719356.9	40
7811	line*coccidia	1	3	896878.7	719356.9	40
7811	line*coccidia	2	0	560007.8	719356.9	40
7811	line*coccidia	2	1	2606741	719356.9	40
7811	line*coccidia	2	2	467334.3	719356.9	40
7811	line*coccidia	2	3	473758.2	719356.9	40
7812	line	1		448353.3	422406	40
7812	line	2		2176398	422406	40
7812	coccidia		0	1400057	597372.2	40
7812	coccidia		1	2181701	597372.2	40
7812	coccidia		2	891290.8	597372.2	40
7812	coccidia		3	776454.5	597372.2	40
7812	line*coccidia	1	0	489369.3	844811.9	40
7812	line*coccidia	1	1	94863.83	844811.9	40
7812	line*coccidia	1	2	220950	844811.9	40
7812	line*coccidia	1	3	988230.2	844811.9	40
7812	line*coccidia	2	0	2310744	844811.9	40
7812	line*coccidia	2	1	4268538	844811.9	40
7812	line*coccidia	2	2	1561632	844811.9	40
7812	line*coccidia	2	3	564678.8	844811.9	40
7819	line	1		345179.9	140871.8	40
7819	line	2		1113391	140871.8	40
7819	coccidia		0	537908.2	199222.8	40
7819	coccidia		1	1268143	199222.8	40
7819	coccidia		2	450208.8	199222.8	40
7819	coccidia		3	660882.6	199222.8	40
7819	line*coccidia	1	0	319561	281743.6	40
7819	line*coccidia	1	1	83384.83	281743.6	40
7819	line*coccidia	1	2	139125.2	281743.6	40
7819	line*coccidia	1	3	838648.5	281743.6	40
7819	line*coccidia	2	0	756255.3	281743.6	40
7819	line*coccidia	2	1	2452900	281743.6	40
7819	line*coccidia	2	2	761292.3	281743.6	40
7819	line*coccidia	2	3	483116.7	281743.6	40
7821	line	1		533800.8	246701.9	40
7821	line	2		1328095	246701.9	40

7821	coccidia		0	848162.3	348889.2	40
7821	coccidia		1	1252490	348889.2	40
7821	coccidia		2	828016.8	348889.2	40
7821	coccidia		3	795123.1	348889.2	40
7821	line*coccidia	1	0	452041.5	493403.9	40
7821	line*coccidia	1	1	271320	493403.9	40
7821	line*coccidia	1	2	313935	493403.9	40
7821	line*coccidia	1	3	1097907	493403.9	40
7821	line*coccidia	2	0	1244283	493403.9	40
7821	line*coccidia	2	1	2233659	493403.9	40
7821	line*coccidia	2	2	1342099	493403.9	40
7821	line*coccidia	2	3	492339.5	493403.9	40
7822	line	1		1394151	379730.8	40
7822	line	2		1061231	379730.8	40
7822	coccidia		0	542735.7	537020.4	40
7822	coccidia		1	1368604	537020.4	40
7822	coccidia		2	1659589	537020.4	40
7822	coccidia		3	1339836	537020.4	40
7822	line*coccidia	1	0	632588.7	759461.5	40
7822	line*coccidia	1	1	63132.33	759461.5	40
7822	line*coccidia	1	2	2880304	759461.5	40
7822	line*coccidia	1	3	2000577	759461.5	40
7822	line*coccidia	2	0	452882.7	759461.5	40
7822	line*coccidia	2	1	2674075	759461.5	40
7822	line*coccidia	2	2	438873.3	759461.5	40
7822	line*coccidia	2	3	679094.5	759461.5	40
8001	line	1		33269.79	15652.05	40
8001	line	2		70224.92	15652.05	40
8001	coccidia		0	70424.92	22135.34	40
8001	coccidia		1	38544.5	22135.34	40
8001	coccidia		2	40343	22135.34	40
8001	coccidia		3	57677	22135.34	40
8001	line*coccidia	1	0	31039	31304.1	40
8001	line*coccidia	1	1	44844.67	31304.1	40
8001	line*coccidia	1	2	26247	31304.1	40
8001	line*coccidia	1	3	30948.5	31304.1	40
8001	line*coccidia	2	0	109810.8	31304.1	40
8001	line*coccidia	2	1	32244.33	31304.1	40
8001	line*coccidia	2	2	54439	31304.1	40
8001	line*coccidia	2	3	84405.5	31304.1	40
8002	line	1		36797.17	8761.952	40
8002	line	2		62516.13	8761.952	40
8002	coccidia		0	33913.25	12391.27	40
8002	coccidia		1	64240.83	12391.27	40
8002	coccidia		2	41237.08	12391.27	40
8002	coccidia		3	59235.42	12391.27	40
8002	line*coccidia	1	0	25736.5	17523.9	40
8002	line*coccidia	1	1	26287.17	17523.9	40
8002	line*coccidia	1	2	35264.17	17523.9	40

8002	line*coccidia	1	3	59900.83	17523.9	40
8002	line*coccidia	2	0	42090	17523.9	40
8002	line*coccidia	2	1	102194.5	17523.9	40
8002	line*coccidia	2	2	47210	17523.9	40
8002	line*coccidia	2	3	58570	17523.9	40
8003	line	1		1.82E-12	7367.715	40
8003	line	2		33272.29	7367.715	40
8003	coccidia		0	11609.25	10419.52	40
8003	coccidia		1	0	10419.52	40
8003	coccidia		2	54935.33	10419.52	40
8003	coccidia		3	0	10419.52	40
8003	line*coccidia	1	0	0	14735.43	40
8003	line*coccidia	1	1	0	14735.43	40
8003	line*coccidia	1	2	3.64E-12	14735.43	40
8003	line*coccidia	1	3	3.64E-12	14735.43	40
8003	line*coccidia	2	0	23218.5	14735.43	40
8003	line*coccidia	2	1	0	14735.43	40
8003	line*coccidia	2	2	109870.7	14735.43	40
8003	line*coccidia	2	3	-3.6E-12	14735.43	40
8004	line	1		58636.63	13264.98	40
8004	line	2		82579.96	13264.98	40
8004	coccidia		0	49976.75	18759.52	40
8004	coccidia		1	124498.8	18759.52	40
8004	coccidia		2	42790.33	18759.52	40
8004	coccidia		3	65167.25	18759.52	40
8004	line*coccidia	1	0	27846.83	26529.96	40
8004	line*coccidia	1	1	68504	26529.96	40
8004	line*coccidia	1	2	34965.5	26529.96	40
8004	line*coccidia	1	3	103230.2	26529.96	40
8004	line*coccidia	2	0	72106.67	26529.96	40
8004	line*coccidia	2	1	180493.7	26529.96	40
8004	line*coccidia	2	2	50615.17	26529.96	40
8004	line*coccidia	2	3	27104.33	26529.96	40
8005	line	1		203501.1	29269.85	40
8005	line	2		268352.7	29269.85	40
8005	coccidia		0	255033.6	41393.82	40
8005	coccidia		1	285509.5	41393.82	40
8005	coccidia		2	161916.7	41393.82	40
8005	coccidia		3	241247.8	41393.82	40
8005	line*coccidia	1	0	181434.2	58539.7	40
8005	line*coccidia	1	1	145886.2	58539.7	40
8005	line*coccidia	1	2	163227.8	58539.7	40
8005	line*coccidia	1	3	323456.3	58539.7	40
8005	line*coccidia	2	0	328633	58539.7	40
8005	line*coccidia	2	1	425132.8	58539.7	40
8005	line*coccidia	2	2	160605.5	58539.7	40
8005	line*coccidia	2	3	159039.3	58539.7	40
8006	line	1		273188.7	68636.33	40
8006	line	2		368746	68636.33	40

8006	coccidia		0	278713.6	97066.43	40
8006	coccidia		1	306974.8	97066.43	40
8006	coccidia		2	248951.9	97066.43	40
8006	coccidia		3	449228.9	97066.43	40
8006	line*coccidia	1	0	129189.7	137272.7	40
8006	line*coccidia	1	1	76354.67	137272.7	40
8006	line*coccidia	1	2	195278.8	137272.7	40
8006	line*coccidia	1	3	691931.5	137272.7	40
8006	line*coccidia	2	0	428237.5	137272.7	40
8006	line*coccidia	2	1	537595	137272.7	40
8006	line*coccidia	2	2	302625	137272.7	40
8006	line*coccidia	2	3	206526.3	137272.7	40
8009	line	1		419270.3	92602.29	40
8009	line	2		486441.9	92602.29	40
8009	coccidia		0	372485.3	130959.4	40
8009	coccidia		1	629886.4	130959.4	40
8009	coccidia		2	494205.1	130959.4	40
8009	coccidia		3	314847.5	130959.4	40
8009	line*coccidia	1	0	357143.3	185204.6	40
8009	line*coccidia	1	1	649163.8	185204.6	40
8009	line*coccidia	1	2	177468	185204.6	40
8009	line*coccidia	1	3	493305.8	185204.6	40
8009	line*coccidia	2	0	387827.2	185204.6	40
8009	line*coccidia	2	1	610609	185204.6	40
8009	line*coccidia	2	2	810942.2	185204.6	40
8009	line*coccidia	2	3	136389.2	185204.6	40
8010	line	1		473721	58124.74	40
8010	line	2		514176.6	58124.74	40
8010	coccidia		0	587309.8	82200.79	40
8010	coccidia		1	551140.7	82200.79	40
8010	coccidia		2	331898.5	82200.79	40
8010	coccidia		3	505446.4	82200.79	40
8010	line*coccidia	1	0	450388.2	116249.5	40
8010	line*coccidia	1	1	370221.8	116249.5	40
8010	line*coccidia	1	2	343924.2	116249.5	40
8010	line*coccidia	1	3	730350	116249.5	40
8010	line*coccidia	2	0	724231.3	116249.5	40
8010	line*coccidia	2	1	732059.5	116249.5	40
8010	line*coccidia	2	2	319872.8	116249.5	40
8010	line*coccidia	2	3	280542.8	116249.5	40
8011	line	1		707954.2	80065.54	40
8011	line	2		747207.3	80065.54	40
8011	coccidia		0	853194.2	113229.8	40
8011	coccidia		1	786725.1	113229.8	40
8011	coccidia		2	435245.7	113229.8	40
8011	coccidia		3	835158	113229.8	40
8011	line*coccidia	1	0	658438.2	160131.1	40
8011	line*coccidia	1	1	566988.3	160131.1	40
8011	line*coccidia	1	2	380400.5	160131.1	40

8011	line*coccidia	1	3	1225990	160131.1	40
8011	line*coccidia	2	0	1047950	160131.1	40
8011	line*coccidia	2	1	1006462	160131.1	40
8011	line*coccidia	2	2	490090.8	160131.1	40
8011	line*coccidia	2	3	444326.2	160131.1	40
8012	line	1		4844965	923802.3	40
8012	line	2		5431926	923802.3	40
8012	coccidia		0	5283654	1306454	40
8012	coccidia		1	3871041	1306454	40
8012	coccidia		2	5618451	1306454	40
8012	coccidia		3	5780636	1306454	40
8012	line*coccidia	1	0	4227090	1847605	40
8012	line*coccidia	1	1	4392331	1847605	40
8012	line*coccidia	1	2	1416675	1847605	40
8012	line*coccidia	1	3	9343766	1847605	40
8012	line*coccidia	2	0	6340218	1847605	40
8012	line*coccidia	2	1	3349752	1847605	40
8012	line*coccidia	2	2	9820228	1847605	40
8012	line*coccidia	2	3	2217505	1847605	40
8014	line	1		65931.13	6224.426	40
8014	line	2		50289.13	6224.426	40
8014	coccidia		0	45056.92	8802.668	40
8014	coccidia		1	95587	8802.668	40
8014	coccidia		2	38417.5	8802.668	40
8014	coccidia		3	53379.08	8802.668	40
8014	line*coccidia	1	0	35247.83	12448.85	40
8014	line*coccidia	1	1	111520.3	12448.85	40
8014	line*coccidia	1	2	32120.33	12448.85	40
8014	line*coccidia	1	3	84836	12448.85	40
8014	line*coccidia	2	0	54866	12448.85	40
8014	line*coccidia	2	1	79653.67	12448.85	40
8014	line*coccidia	2	2	44714.67	12448.85	40
8014	line*coccidia	2	3	21922.17	12448.85	40
8016	line	1		95307.75	20386.38	40
8016	line	2		77724.79	20386.38	40
8016	coccidia		0	101442.3	28830.7	40
8016	coccidia		1	70968	28830.7	40
8016	coccidia		2	110497.3	28830.7	40
8016	coccidia		3	63157.5	28830.7	40
8016	line*coccidia	1	0	126659	40772.76	40
8016	line*coccidia	1	1	81402.83	40772.76	40
8016	line*coccidia	1	2	77767.83	40772.76	40
8016	line*coccidia	1	3	95401.33	40772.76	40
8016	line*coccidia	2	0	76225.67	40772.76	40
8016	line*coccidia	2	1	60533.17	40772.76	40
8016	line*coccidia	2	2	143226.7	40772.76	40
8016	line*coccidia	2	3	30913.67	40772.76	40
8017	line	1		3066989	719192.8	40
8017	line	2		4986585	719192.8	40

8017	coccidia		0	3538650	1017092	40
8017	coccidia		1	2392601	1017092	40
8017	coccidia		2	5723869	1017092	40
8017	coccidia		3	4452028	1017092	40
8017	line*coccidia	1	0	2999769	1438386	40
8017	line*coccidia	1	1	2765543	1438386	40
8017	line*coccidia	1	2	1380211	1438386	40
8017	line*coccidia	1	3	5122435	1438386	40
8017	line*coccidia	2	0	4077532	1438386	40
8017	line*coccidia	2	1	2019658	1438386	40
8017	line*coccidia	2	2	10067527	1438386	40
8017	line*coccidia	2	3	3781621	1438386	40
8018	line	1		1929110	197913.7	40
8018	line	2		1546032	197913.7	40
8018	coccidia		0	1978726	279892.3	40
8018	coccidia		1	1782621	279892.3	40
8018	coccidia		2	1577696	279892.3	40
8018	coccidia		3	1611241	279892.3	40
8018	line*coccidia	1	0	2104785	395827.5	40
8018	line*coccidia	1	1	2495863	395827.5	40
8018	line*coccidia	1	2	427716	395827.5	40
8018	line*coccidia	1	3	2688075	395827.5	40
8018	line*coccidia	2	0	1852667	395827.5	40
8018	line*coccidia	2	1	1069379	395827.5	40
8018	line*coccidia	2	2	2727675	395827.5	40
8018	line*coccidia	2	3	534408.2	395827.5	40
8019	line	1		66855.13	29511.91	40
8019	line	2		96347.5	29511.91	40
8019	coccidia		0	154976.9	41736.15	40
8019	coccidia		1	42375	41736.15	40
8019	coccidia		2	108365.3	41736.15	40
8019	coccidia		3	20688	41736.15	40
8019	line*coccidia	1	0	198217.8	59023.82	40
8019	line*coccidia	1	1	69202.67	59023.82	40
8019	line*coccidia	1	2	2.18E-11	59023.82	40
8019	line*coccidia	1	3	2.18E-11	59023.82	40
8019	line*coccidia	2	0	111736	59023.82	40
8019	line*coccidia	2	1	15547.33	59023.82	40
8019	line*coccidia	2	2	216730.7	59023.82	40
8019	line*coccidia	2	3	41376	59023.82	40
8025	line	1		17811.21	3363.677	40
8025	line	2		-1.4E-12	3363.677	40
8025	coccidia		0	-1.8E-12	4756.958	40
8025	coccidia		1	35622.42	4756.958	40
8025	coccidia		2	0	4756.958	40
8025	coccidia		3	0	4756.958	40
8025	line*coccidia	1	0	-1.8E-12	6727.354	40
8025	line*coccidia	1	1	71244.83	6727.354	40
8025	line*coccidia	1	2	1.27E-12	6727.354	40

8025	line*coccidia	1	3	-3.6E-12	6727.354	40
8025	line*coccidia	2	0	-1.8E-12	6727.354	40
8025	line*coccidia	2	1	-6.1E-12	6727.354	40
8025	line*coccidia	2	2	-1.3E-12	6727.354	40
8025	line*coccidia	2	3	3.59E-12	6727.354	40
8026	line	1		46384.71	22770.41	40
8026	line	2		-1.3E-12	22770.41	40
8026	coccidia		0	0	32202.22	40
8026	coccidia		1	92769.42	32202.22	40
8026	coccidia		2	0	32202.22	40
8026	coccidia		3	0	32202.22	40
8026	line*coccidia	1	0	0	45540.81	40
8026	line*coccidia	1	1	185538.8	45540.81	40
8026	line*coccidia	1	2	6.57E-12	45540.81	40
8026	line*coccidia	1	3	6.57E-12	45540.81	40
8026	line*coccidia	2	0	0	45540.81	40
8026	line*coccidia	2	1	7.98E-12	45540.81	40
8026	line*coccidia	2	2	-6.6E-12	45540.81	40
8026	line*coccidia	2	3	-6.6E-12	45540.81	40
8027	line	1		229952.5	65288.44	40
8027	line	2		-4E-12	65288.44	40
8027	coccidia		0	0	92331.8	40
8027	coccidia		1	459904.9	92331.8	40
8027	coccidia		2	9.7E-12	92331.8	40
8027	coccidia		3	-2.9E-11	92331.8	40
8027	line*coccidia	1	0	0	130576.9	40
8027	line*coccidia	1	1	919809.8	130576.9	40
8027	line*coccidia	1	2	1.82E-11	130576.9	40
8027	line*coccidia	1	3	-2.1E-11	130576.9	40
8027	line*coccidia	2	0	0	130576.9	40
8027	line*coccidia	2	1	2.06E-11	130576.9	40
8027	line*coccidia	2	2	1.19E-12	130576.9	40
8027	line*coccidia	2	3	-3.8E-11	130576.9	40
8028	line	1		51579.88	16127.66	40
8028	line	2		39486.96	16127.66	40
8028	coccidia		0	23392	22807.96	40
8028	coccidia		1	89612.92	22807.96	40
8028	coccidia		2	56341.58	22807.96	40
8028	coccidia		3	12787.17	22807.96	40
8028	line*coccidia	1	0	10666.67	32255.32	40
8028	line*coccidia	1	1	94658.83	32255.32	40
8028	line*coccidia	1	2	100994	32255.32	40
8028	line*coccidia	1	3	-3.6E-12	32255.32	40
8028	line*coccidia	2	0	36117.33	32255.32	40
8028	line*coccidia	2	1	84567	32255.32	40
8028	line*coccidia	2	2	11689.17	32255.32	40
8028	line*coccidia	2	3	25574.33	32255.32	40
8101	line	1		1400842	206685.3	40
8101	line	2		1172996	206685.3	40

8101	coccidia		0	922639.7	292297.1	40
8101	coccidia		1	1949964	292297.1	40
8101	coccidia		2	734298.4	292297.1	40
8101	coccidia		3	1540775	292297.1	40
8101	line*coccidia	1	0	1165302	413370.6	40
8101	line*coccidia	1	1	1838355	413370.6	40
8101	line*coccidia	1	2	467051.5	413370.6	40
8101	line*coccidia	1	3	2132658	413370.6	40
8101	line*coccidia	2	0	679977	413370.6	40
8101	line*coccidia	2	1	2061573	413370.6	40
8101	line*coccidia	2	2	1001545	413370.6	40
8101	line*coccidia	2	3	948890.8	413370.6	40
8104	line	1		1145811	410110.8	40
8104	line	2		885261.8	410110.8	40
8104	coccidia		0	1887590	579984.2	40
8104	coccidia		1	287783.3	579984.2	40
8104	coccidia		2	892841.1	579984.2	40
8104	coccidia		3	993931.3	579984.2	40
8104	line*coccidia	1	0	2773736	820221.6	40
8104	line*coccidia	1	1	156519	820221.6	40
8104	line*coccidia	1	2	296392	820221.6	40
8104	line*coccidia	1	3	1356597	820221.6	40
8104	line*coccidia	2	0	1001444	820221.6	40
8104	line*coccidia	2	1	419047.7	820221.6	40
8104	line*coccidia	2	2	1489290	820221.6	40
8104	line*coccidia	2	3	631265.3	820221.6	40
8105	line	1		3074844	326812.1	40
8105	line	2		2767359	326812.1	40
8105	coccidia		0	2461695	462182.1	40
8105	coccidia		1	3324972	462182.1	40
8105	coccidia		2	1093680	462182.1	40
8105	coccidia		3	4804060	462182.1	40
8105	line*coccidia	1	0	2635922	653624.2	40
8105	line*coccidia	1	1	3103617	653624.2	40
8105	line*coccidia	1	2	856467.2	653624.2	40
8105	line*coccidia	1	3	5703371	653624.2	40
8105	line*coccidia	2	0	2287469	653624.2	40
8105	line*coccidia	2	1	3546327	653624.2	40
8105	line*coccidia	2	2	1330892	653624.2	40
8105	line*coccidia	2	3	3904749	653624.2	40
8106	line	1		333025	84194.3	40
8106	line	2		521088.9	84194.3	40
8106	coccidia		0	388313.3	119068.7	40
8106	coccidia		1	631618.3	119068.7	40
8106	coccidia		2	313502.3	119068.7	40
8106	coccidia		3	374794	119068.7	40
8106	line*coccidia	1	0	146024.7	168388.6	40
8106	line*coccidia	1	1	483099.7	168388.6	40
8106	line*coccidia	1	2	215506.3	168388.6	40

8106	line*coccidia	1	3	487469.3	168388.6	40
8106	line*coccidia	2	0	630602	168388.6	40
8106	line*coccidia	2	1	780136.8	168388.6	40
8106	line*coccidia	2	2	411498.2	168388.6	40
8106	line*coccidia	2	3	262118.7	168388.6	40
8110	line	1		693872.6	269178.4	40
8110	line	2		1204792	269178.4	40
8110	coccidia		0	743006.8	380675.7	40
8110	coccidia		1	731468.3	380675.7	40
8110	coccidia		2	936584.5	380675.7	40
8110	coccidia		3	1386270	380675.7	40
8110	line*coccidia	1	0	144808.5	538356.8	40
8110	line*coccidia	1	1	265525.8	538356.8	40
8110	line*coccidia	1	2	79975	538356.8	40
8110	line*coccidia	1	3	2285181	538356.8	40
8110	line*coccidia	2	0	1341205	538356.8	40
8110	line*coccidia	2	1	1197411	538356.8	40
8110	line*coccidia	2	2	1793194	538356.8	40
8110	line*coccidia	2	3	487359.8	538356.8	40
8115	line	1		25653.79	12423.55	40
8115	line	2		62525.67	12423.55	40
8115	coccidia		0	0	17569.55	40
8115	coccidia		1	124325.8	17569.55	40
8115	coccidia		2	52033.08	17569.55	40
8115	coccidia		3	-7.3E-12	17569.55	40
8115	line*coccidia	1	0	0	24847.1	40
8115	line*coccidia	1	1	102615.2	24847.1	40
8115	line*coccidia	1	2	-3.6E-12	24847.1	40
8115	line*coccidia	1	3	1.09E-11	24847.1	40
8115	line*coccidia	2	0	0	24847.1	40
8115	line*coccidia	2	1	146036.5	24847.1	40
8115	line*coccidia	2	2	104066.2	24847.1	40
8115	line*coccidia	2	3	-2.5E-11	24847.1	40
8119	line	1		762248.6	90465.33	40
8119	line	2		610318.5	90465.33	40
8119	coccidia		0	570565.3	127937.3	40
8119	coccidia		1	925966.8	127937.3	40
8119	coccidia		2	499973.2	127937.3	40
8119	coccidia		3	748628.9	127937.3	40
8119	line*coccidia	1	0	545929.7	180930.7	40
8119	line*coccidia	1	1	1130637	180930.7	40
8119	line*coccidia	1	2	264849.3	180930.7	40
8119	line*coccidia	1	3	1107579	180930.7	40
8119	line*coccidia	2	0	595201	180930.7	40
8119	line*coccidia	2	1	721297	180930.7	40
8119	line*coccidia	2	2	735097	180930.7	40
8119	line*coccidia	2	3	389679	180930.7	40
8122	line	1		775025	166021.7	40
8122	line	2		989790.9	166021.7	40

8122	coccidia		0	949596.6	234790.1	40
8122	coccidia		1	383777.7	234790.1	40
8122	coccidia		2	531955.3	234790.1	40
8122	coccidia		3	1664302	234790.1	40
8122	line*coccidia	1	0	935191.7	332043.4	40
8122	line*coccidia	1	1	292241.8	332043.4	40
8122	line*coccidia	1	2	191919.7	332043.4	40
8122	line*coccidia	1	3	1680747	332043.4	40
8122	line*coccidia	2	0	964001.5	332043.4	40
8122	line*coccidia	2	1	475313.5	332043.4	40
8122	line*coccidia	2	2	871990.8	332043.4	40
8122	line*coccidia	2	3	1647858	332043.4	40
8126	line	1		3928990	410363.1	40
8126	line	2		5735137	410363.1	40
8126	coccidia		0	5998302	580341	40
8126	coccidia		1	4625830	580341	40
8126	coccidia		2	2861563	580341	40
8126	coccidia		3	5842558	580341	40
8126	line*coccidia	1	0	3399127	820726.1	40
8126	line*coccidia	1	1	3445336	820726.1	40
8126	line*coccidia	1	2	1184895	820726.1	40
8126	line*coccidia	1	3	7686601	820726.1	40
8126	line*coccidia	2	0	8597477	820726.1	40
8126	line*coccidia	2	1	5806324	820726.1	40
8126	line*coccidia	2	2	4538231	820726.1	40
8126	line*coccidia	2	3	3998516	820726.1	40
8130	line	1		648487.5	140968.4	40
8130	line	2		336399.7	140968.4	40
8130	coccidia		0	709482.7	199359.4	40
8130	coccidia		1	155991.4	199359.4	40
8130	coccidia		2	493903.1	199359.4	40
8130	coccidia		3	610397.2	199359.4	40
8130	line*coccidia	1	0	1071306	281936.8	40
8130	line*coccidia	1	1	73522.5	281936.8	40
8130	line*coccidia	1	2	546119	281936.8	40
8130	line*coccidia	1	3	903002.8	281936.8	40
8130	line*coccidia	2	0	347659.8	281936.8	40
8130	line*coccidia	2	1	238460.3	281936.8	40
8130	line*coccidia	2	2	441687.2	281936.8	40
8130	line*coccidia	2	3	317791.5	281936.8	40
8134	line	1		159131.2	20939.92	40
8134	line	2		153500.5	20939.92	40
8134	coccidia		0	162496.3	29613.53	40
8134	coccidia		1	171435	29613.53	40
8134	coccidia		2	92862.83	29613.53	40
8134	coccidia		3	198469.4	29613.53	40
8134	line*coccidia	1	0	121341.7	41879.85	40
8134	line*coccidia	1	1	189800.5	41879.85	40
8134	line*coccidia	1	2	60855.67	41879.85	40

8134	line*coccidia	1	3	264527	41879.85	40
8134	line*coccidia	2	0	203650.8	41879.85	40
8134	line*coccidia	2	1	153069.5	41879.85	40
8134	line*coccidia	2	2	124870	41879.85	40
8134	line*coccidia	2	3	132411.8	41879.85	40
8139	line	1		1199422	150165.5	40
8139	line	2		894214.1	150165.5	40
8139	coccidia		0	1861718	212366.1	40
8139	coccidia		1	782049.8	212366.1	40
8139	coccidia		2	488504.2	212366.1	40
8139	coccidia		3	1055000	212366.1	40
8139	line*coccidia	1	0	2481047	300331.1	40
8139	line*coccidia	1	1	784944.7	300331.1	40
8139	line*coccidia	1	2	516957.5	300331.1	40
8139	line*coccidia	1	3	1014739	300331.1	40
8139	line*coccidia	2	0	1242389	300331.1	40
8139	line*coccidia	2	1	779154.8	300331.1	40
8139	line*coccidia	2	2	460050.8	300331.1	40
8139	line*coccidia	2	3	1095262	300331.1	40
8141	line	1		267404.2	61586.29	40
8141	line	2		7.13E-12	61586.29	40
8141	coccidia		0	0	87096.16	40
8141	coccidia		1	534808.3	87096.16	40
8141	coccidia		2	0	87096.16	40
8141	coccidia		3	0	87096.16	40
8141	line*coccidia	1	0	0	123172.6	40
8141	line*coccidia	1	1	1069617	123172.6	40
8141	line*coccidia	1	2	-9.5E-12	123172.6	40
8141	line*coccidia	1	3	6.81E-11	123172.6	40
8141	line*coccidia	2	0	0	123172.6	40
8141	line*coccidia	2	1	8.71E-11	123172.6	40
8141	line*coccidia	2	2	9.5E-12	123172.6	40
8141	line*coccidia	2	3	-6.8E-11	123172.6	40
8142	line	1		71054.83	17260.64	40
8142	line	2		116109.8	17260.64	40
8142	coccidia		0	116564.6	24410.23	40
8142	coccidia		1	155181.6	24410.23	40
8142	coccidia		2	39939.33	24410.23	40
8142	coccidia		3	62643.75	24410.23	40
8142	line*coccidia	1	0	97844.17	34521.27	40
8142	line*coccidia	1	1	166235.7	34521.27	40
8142	line*coccidia	1	2	0	34521.27	40
8142	line*coccidia	1	3	20139.5	34521.27	40
8142	line*coccidia	2	0	135285	34521.27	40
8142	line*coccidia	2	1	144127.5	34521.27	40
8142	line*coccidia	2	2	79878.67	34521.27	40
8142	line*coccidia	2	3	105148	34521.27	40
8143	line	1		120301.1	56380.41	40
8143	line	2		165023.3	56380.41	40

8143	coccidia		0	207233.3	79733.93	40
8143	coccidia		1	247766.6	79733.93	40
8143	coccidia		2	13491.08	79733.93	40
8143	coccidia		3	102157.8	79733.93	40
8143	line*coccidia	1	0	51921.17	112760.8	40
8143	line*coccidia	1	1	242046.7	112760.8	40
8143	line*coccidia	1	2	-1.5E-11	112760.8	40
8143	line*coccidia	1	3	187236.7	112760.8	40
8143	line*coccidia	2	0	362545.5	112760.8	40
8143	line*coccidia	2	1	253486.5	112760.8	40
8143	line*coccidia	2	2	26982.17	112760.8	40
8143	line*coccidia	2	3	17078.83	112760.8	40
8144	line	1		91149.5	14135.15	40
8144	line	2		77143.04	14135.15	40
8144	coccidia		0	121828.1	19990.12	40
8144	coccidia		1	65166.25	19990.12	40
8144	coccidia		2	72978.83	19990.12	40
8144	coccidia		3	76611.92	19990.12	40
8144	line*coccidia	1	0	93535.67	28270.3	40
8144	line*coccidia	1	1	77215.17	28270.3	40
8144	line*coccidia	1	2	106011.7	28270.3	40
8144	line*coccidia	1	3	87835.5	28270.3	40
8144	line*coccidia	2	0	150120.5	28270.3	40
8144	line*coccidia	2	1	53117.33	28270.3	40
8144	line*coccidia	2	2	39946	28270.3	40
8144	line*coccidia	2	3	65388.33	28270.3	40
8145	line	1		334823.1	57115.34	40
8145	line	2		159130.9	57115.34	40
8145	coccidia		0	388603.7	80773.28	40
8145	coccidia		1	346632.3	80773.28	40
8145	coccidia		2	126832.8	80773.28	40
8145	coccidia		3	125839.3	80773.28	40
8145	line*coccidia	1	0	580532	114230.7	40
8145	line*coccidia	1	1	492160.8	114230.7	40
8145	line*coccidia	1	2	104142.3	114230.7	40
8145	line*coccidia	1	3	162457.3	114230.7	40
8145	line*coccidia	2	0	196675.3	114230.7	40
8145	line*coccidia	2	1	201103.7	114230.7	40
8145	line*coccidia	2	2	149523.2	114230.7	40
8145	line*coccidia	2	3	89221.33	114230.7	40
8146	line	1		1447822	452391.7	40
8146	line	2		-4E-11	452391.7	40
8146	coccidia		0	0	639778.5	40
8146	coccidia		1	2895643	639778.5	40
8146	coccidia		2	0	639778.5	40
8146	coccidia		3	0	639778.5	40
8146	line*coccidia	1	0	0	904783.4	40
8146	line*coccidia	1	1	5791287	904783.4	40
8146	line*coccidia	1	2	2.09E-10	904783.4	40

8146	line*coccidia	1	3	2.09E-10	904783.4	40
8146	line*coccidia	2	0	0	904783.4	40
8146	line*coccidia	2	1	2.57E-10	904783.4	40
8146	line*coccidia	2	2	-2.1E-10	904783.4	40
8146	line*coccidia	2	3	-2.1E-10	904783.4	40
8148	line	1		1053122	347724.2	40
8148	line	2		2E-11	347724.2	40
8148	coccidia		0	0	491756.3	40
8148	coccidia		1	2025464	491756.3	40
8148	coccidia		2	-4.4E-11	491756.3	40
8148	coccidia		3	80780.5	491756.3	40
8148	line*coccidia	1	0	0	695448.5	40
8148	line*coccidia	1	1	4050928	695448.5	40
8148	line*coccidia	1	2	-8.7E-11	695448.5	40
8148	line*coccidia	1	3	161561	695448.5	40
8148	line*coccidia	2	0	0	695448.5	40
8148	line*coccidia	2	1	8E-11	695448.5	40
8148	line*coccidia	2	2	0	695448.5	40
8148	line*coccidia	2	3	0	695448.5	40
8149	line	1		199198.4	58643.04	40
8149	line	2		36114.04	58643.04	40
8149	coccidia		0	0	82933.79	40
8149	coccidia		1	319232.4	82933.79	40
8149	coccidia		2	72228.08	82933.79	40
8149	coccidia		3	79164.33	82933.79	40
8149	line*coccidia	1	0	0	117286.1	40
8149	line*coccidia	1	1	638464.8	117286.1	40
8149	line*coccidia	1	2	2.91E-11	117286.1	40
8149	line*coccidia	1	3	158328.7	117286.1	40
8149	line*coccidia	2	0	0	117286.1	40
8149	line*coccidia	2	1	-2.9E-11	117286.1	40
8149	line*coccidia	2	2	144456.2	117286.1	40
8149	line*coccidia	2	3	0	117286.1	40
8201	line	1		54776.83	17645.9	40
8201	line	2		56572.33	17645.9	40
8201	coccidia		0	66686.42	24955.07	40
8201	coccidia		1	100837.8	24955.07	40
8201	coccidia		2	28886.75	24955.07	40
8201	coccidia		3	26287.33	24955.07	40
8201	line*coccidia	1	0	18721.83	35291.79	40
8201	line*coccidia	1	1	138915.5	35291.79	40
8201	line*coccidia	1	2	38786.17	35291.79	40
8201	line*coccidia	1	3	22683.83	35291.79	40
8201	line*coccidia	2	0	114651	35291.79	40
8201	line*coccidia	2	1	62760.17	35291.79	40
8201	line*coccidia	2	2	18987.33	35291.79	40
8201	line*coccidia	2	3	29890.83	35291.79	40
8202	line	1		56208.04	14294.15	40
8202	line	2		66225.17	14294.15	40

8202	coccidia		0	56494.33	20214.97	40
8202	coccidia		1	106186.8	20214.97	40
8202	coccidia		2	29545.58	20214.97	40
8202	coccidia		3	52639.67	20214.97	40
8202	line*coccidia	1	0	21560.17	28588.29	40
8202	line*coccidia	1	1	145648	28588.29	40
8202	line*coccidia	1	2	27139.83	28588.29	40
8202	line*coccidia	1	3	30484.17	28588.29	40
8202	line*coccidia	2	0	91428.5	28588.29	40
8202	line*coccidia	2	1	66725.67	28588.29	40
8202	line*coccidia	2	2	31951.33	28588.29	40
8202	line*coccidia	2	3	74795.17	28588.29	40
8203	line	1		38205.79	17434.73	40
8203	line	2		64921.58	17434.73	40
8203	coccidia		0	33509.42	24656.44	40
8203	coccidia		1	84132.67	24656.44	40
8203	coccidia		2	27690.25	24656.44	40
8203	coccidia		3	60922.42	24656.44	40
8203	line*coccidia	1	0	28949.67	34869.47	40
8203	line*coccidia	1	1	-7.3E-12	34869.47	40
8203	line*coccidia	1	2	37568.17	34869.47	40
8203	line*coccidia	1	3	86305.33	34869.47	40
8203	line*coccidia	2	0	38069.17	34869.47	40
8203	line*coccidia	2	1	168265.3	34869.47	40
8203	line*coccidia	2	2	17812.33	34869.47	40
8203	line*coccidia	2	3	35539.5	34869.47	40
8204	line	1		43055.54	8229.659	40
8204	line	2		56841.38	8229.659	40
8204	coccidia		0	49141.75	11638.5	40
8204	coccidia		1	50275.17	11638.5	40
8204	coccidia		2	55019.17	11638.5	40
8204	coccidia		3	45357.75	11638.5	40
8204	line*coccidia	1	0	43137.33	16459.32	40
8204	line*coccidia	1	1	1.82E-11	16459.32	40
8204	line*coccidia	1	2	69753.5	16459.32	40
8204	line*coccidia	1	3	59331.33	16459.32	40
8204	line*coccidia	2	0	55146.17	16459.32	40
8204	line*coccidia	2	1	100550.3	16459.32	40
8204	line*coccidia	2	2	40284.83	16459.32	40
8204	line*coccidia	2	3	31384.17	16459.32	40
8213	line	1		193199.2	34348.34	40
8213	line	2		48478.25	34348.34	40
8213	coccidia		0	44125.67	48575.89	40
8213	coccidia		1	237622.3	48575.89	40
8213	coccidia		2	64833.17	48575.89	40
8213	coccidia		3	136773.8	48575.89	40
8213	line*coccidia	1	0	41765.33	68696.68	40
8213	line*coccidia	1	1	409067.3	68696.68	40
8213	line*coccidia	1	2	83700.5	68696.68	40

8213	line*coccidia	1	3	238263.7	68696.68	40
8213	line*coccidia	2	0	46486	68696.68	40
8213	line*coccidia	2	1	66177.17	68696.68	40
8213	line*coccidia	2	2	45965.83	68696.68	40
8213	line*coccidia	2	3	35284	68696.68	40
8215	line	1		273014	35203.87	40
8215	line	2		247983.4	35203.87	40
8215	coccidia		0	184734.8	49785.79	40
8215	coccidia		1	292161.6	49785.79	40
8215	coccidia		2	253890.3	49785.79	40
8215	coccidia		3	311208.2	49785.79	40
8215	line*coccidia	1	0	175883.7	70407.74	40
8215	line*coccidia	1	1	292442.2	70407.74	40
8215	line*coccidia	1	2	137593.3	70407.74	40
8215	line*coccidia	1	3	486136.7	70407.74	40
8215	line*coccidia	2	0	193585.8	70407.74	40
8215	line*coccidia	2	1	291881	70407.74	40
8215	line*coccidia	2	2	370187.2	70407.74	40
8215	line*coccidia	2	3	136279.7	70407.74	40
8216	line	1		371755.3	78230.26	40
8216	line	2		178779.2	78230.26	40
8216	coccidia		0	217687.2	110634.3	40
8216	coccidia		1	514399.5	110634.3	40
8216	coccidia		2	170204.6	110634.3	40
8216	coccidia		3	198777.7	110634.3	40
8216	line*coccidia	1	0	195205.5	156460.5	40
8216	line*coccidia	1	1	870825.3	156460.5	40
8216	line*coccidia	1	2	132681.8	156460.5	40
8216	line*coccidia	1	3	288308.3	156460.5	40
8216	line*coccidia	2	0	240168.8	156460.5	40
8216	line*coccidia	2	1	157973.7	156460.5	40
8216	line*coccidia	2	2	207727.3	156460.5	40
8216	line*coccidia	2	3	109247	156460.5	40
8218	line	1		191119.6	44896.59	40
8218	line	2		195998.6	44896.59	40
8218	coccidia		0	158726.2	63493.37	40
8218	coccidia		1	267054.3	63493.37	40
8218	coccidia		2	169957.4	63493.37	40
8218	coccidia		3	178498.5	63493.37	40
8218	line*coccidia	1	0	139028.5	89793.18	40
8218	line*coccidia	1	1	318287.7	89793.18	40
8218	line*coccidia	1	2	105175.8	89793.18	40
8218	line*coccidia	1	3	201986.3	89793.18	40
8218	line*coccidia	2	0	178423.8	89793.18	40
8218	line*coccidia	2	1	215820.8	89793.18	40
8218	line*coccidia	2	2	234739	89793.18	40
8218	line*coccidia	2	3	155010.7	89793.18	40
8224	line	1		1438975	541507.1	40
8224	line	2		10354.58	541507.1	40

8224	coccidia		0	-1.2E-10	765806.6	40
8224	coccidia		1	2740118	765806.6	40
8224	coccidia		2	20709.17	765806.6	40
8224	coccidia		3	137832.3	765806.6	40
8224	line*coccidia	1	0	-1.2E-10	1083014	40
8224	line*coccidia	1	1	5480236	1083014	40
8224	line*coccidia	1	2	1.02E-10	1083014	40
8224	line*coccidia	1	3	275664.5	1083014	40
8224	line*coccidia	2	0	-1.2E-10	1083014	40
8224	line*coccidia	2	1	2.18E-10	1083014	40
8224	line*coccidia	2	2	41418.33	1083014	40
8224	line*coccidia	2	3	-8.7E-11	1083014	40
8233	line	1		156651.6	35540.84	40
8233	line	2		-1.2E-11	35540.84	40
8233	coccidia		0	-1.5E-11	50262.33	40
8233	coccidia		1	313303.3	50262.33	40
8233	coccidia		2	0	50262.33	40
8233	coccidia		3	0	50262.33	40
8233	line*coccidia	1	0	-1.5E-11	71081.67	40
8233	line*coccidia	1	1	626606.5	71081.67	40
8233	line*coccidia	1	2	1.07E-11	71081.67	40
8233	line*coccidia	1	3	1.07E-11	71081.67	40
8233	line*coccidia	2	0	-1.5E-11	71081.67	40
8233	line*coccidia	2	1	-1.1E-11	71081.67	40
8233	line*coccidia	2	2	-1.1E-11	71081.67	40
8233	line*coccidia	2	3	-1.1E-11	71081.67	40
8234	line	1		1.36E-12	20264.76	40
8234	line	2		104474.1	20264.76	40
8234	coccidia		0	95168.58	28658.69	40
8234	coccidia		1	28161.25	28658.69	40
8234	coccidia		2	51242.25	28658.69	40
8234	coccidia		3	34376.08	28658.69	40
8234	line*coccidia	1	0	1.46E-11	40529.51	40
8234	line*coccidia	1	1	-1.8E-12	40529.51	40
8234	line*coccidia	1	2	-7.3E-12	40529.51	40
8234	line*coccidia	1	3	0	40529.51	40
8234	line*coccidia	2	0	190337.2	40529.51	40
8234	line*coccidia	2	1	56322.5	40529.51	40
8234	line*coccidia	2	2	102484.5	40529.51	40
8234	line*coccidia	2	3	68752.17	40529.51	40
8235	line	1		342958.2	91735.24	40
8235	line	2		51345.46	91735.24	40
8235	coccidia		0	44380.58	129733.2	40
8235	coccidia		1	635859.3	129733.2	40
8235	coccidia		2	31369.67	129733.2	40
8235	coccidia		3	76997.75	129733.2	40
8235	line*coccidia	1	0	53229.33	183470.5	40
8235	line*coccidia	1	1	1182094	183470.5	40
8235	line*coccidia	1	2	31759	183470.5	40

8235	line*coccidia	1	3	104750.2	183470.5	40
8235	line*coccidia	2	0	35531.83	183470.5	40
8235	line*coccidia	2	1	89624.33	183470.5	40
8235	line*coccidia	2	2	30980.33	183470.5	40
8235	line*coccidia	2	3	49245.33	183470.5	40
8236	line	1		25173.71	7544.975	40
8236	line	2		41315.92	7544.975	40
8236	coccidia		0	19017.92	10670.21	40
8236	coccidia		1	22791.17	10670.21	40
8236	coccidia		2	16261.42	10670.21	40
8236	coccidia		3	74908.75	10670.21	40
8236	line*coccidia	1	0	6427.5	15089.95	40
8236	line*coccidia	1	1	-1.5E-11	15089.95	40
8236	line*coccidia	1	2	-1.5E-11	15089.95	40
8236	line*coccidia	1	3	94267.33	15089.95	40
8236	line*coccidia	2	0	31608.33	15089.95	40
8236	line*coccidia	2	1	45582.33	15089.95	40
8236	line*coccidia	2	2	32522.83	15089.95	40
8236	line*coccidia	2	3	55550.17	15089.95	40
8237	line	1		130554.5	35624.26	40
8237	line	2		1.6E-14	35624.26	40
8237	coccidia		0	0	50380.31	40
8237	coccidia		1	261109.1	50380.31	40
8237	coccidia		2	0	50380.31	40
8237	coccidia		3	0	50380.31	40
8237	line*coccidia	1	0	0	71248.51	40
8237	line*coccidia	1	1	522218.2	71248.51	40
8237	line*coccidia	1	2	9.68E-12	71248.51	40
8237	line*coccidia	1	3	-9.7E-12	71248.51	40
8237	line*coccidia	2	0	0	71248.51	40
8237	line*coccidia	2	1	2.13E-14	71248.51	40
8237	line*coccidia	2	2	-9.7E-12	71248.51	40
8237	line*coccidia	2	3	9.72E-12	71248.51	40
8240	line	1		932043.2	235455.9	39
8240	line	2		1.92E-10	229781.5	39
8240	coccidia		0	686.6667	324960.1	39
8240	coccidia		1	1863400	340821	39
8240	coccidia		2	9.47E-11	324960.1	39
8240	coccidia		3	1.16E-10	324960.1	39
8240	line*coccidia	1	0	1373.333	459562.9	39
8240	line*coccidia	1	1	3726800	503426	39
8240	line*coccidia	1	2	-5.6E-11	459562.9	39
8240	line*coccidia	1	3	-3.5E-10	459562.9	39
8240	line*coccidia	2	0	1.3E-11	459562.9	39
8240	line*coccidia	2	1	-7.1E-11	459562.9	39
8240	line*coccidia	2	2	2.46E-10	459562.9	39
8240	line*coccidia	2	3	5.8E-10	459562.9	39
8241	line	1		493696.8	147034.5	40
8241	line	2		8.53E-13	147034.5	40

8241	coccidia		0	0	207938.1	40
8241	coccidia		1	987393.6	207938.1	40
8241	coccidia		2	0	207938.1	40
8241	coccidia		3	0	207938.1	40
8241	line*coccidia	1	0	0	294068.9	40
8241	line*coccidia	1	1	1974787	294068.9	40
8241	line*coccidia	1	2	3.77E-11	294068.9	40
8241	line*coccidia	1	3	-4E-11	294068.9	40
8241	line*coccidia	2	0	0	294068.9	40
8241	line*coccidia	2	1	1.14E-12	294068.9	40
8241	line*coccidia	2	2	-3.8E-11	294068.9	40
8241	line*coccidia	2	3	3.99E-11	294068.9	40
8243	line	1		112355.7	29301.2	40
8243	line	2		4.21E-12	29301.2	40
8243	coccidia		0	8471.5	41438.16	40
8243	coccidia		1	167712	41438.16	40
8243	coccidia		2	20524	41438.16	40
8243	coccidia		3	28003.83	41438.16	40
8243	line*coccidia	1	0	16943	58602.41	40
8243	line*coccidia	1	1	335424	58602.41	40
8243	line*coccidia	1	2	41048	58602.41	40
8243	line*coccidia	1	3	56007.67	58602.41	40
8243	line*coccidia	2	0	0	58602.41	40
8243	line*coccidia	2	1	3.14E-11	58602.41	40
8243	line*coccidia	2	2	2.73E-12	58602.41	40
8243	line*coccidia	2	3	-1.7E-11	58602.41	40
8244	line	1		208867.9	50678.16	40
8244	line	2		61866.04	50678.16	40
8244	coccidia		0	72608.25	71669.74	40
8244	coccidia		1	335328.1	71669.74	40
8244	coccidia		2	87242.67	71669.74	40
8244	coccidia		3	46288.83	71669.74	40
8244	line*coccidia	1	0	57564.33	101356.3	40
8244	line*coccidia	1	1	608102.8	101356.3	40
8244	line*coccidia	1	2	77226.67	101356.3	40
8244	line*coccidia	1	3	92577.67	101356.3	40
8244	line*coccidia	2	0	87652.17	101356.3	40
8244	line*coccidia	2	1	62553.33	101356.3	40
8244	line*coccidia	2	2	97258.67	101356.3	40
8244	line*coccidia	2	3	1.16E-10	101356.3	40
8245	line	1		95941.42	27180.94	40
8245	line	2		57714.08	27180.94	40
8245	coccidia		0	43826.75	38439.66	40
8245	coccidia		1	203390.1	38439.66	40
8245	coccidia		2	32353.67	38439.66	40
8245	coccidia		3	27740.5	38439.66	40
8245	line*coccidia	1	0	14227.83	54361.88	40
8245	line*coccidia	1	1	303751.8	54361.88	40
8245	line*coccidia	1	2	29186.67	54361.88	40

8245	line*coccidia	1	3	36599.33	54361.88	40
8245	line*coccidia	2	0	73425.67	54361.88	40
8245	line*coccidia	2	1	103028.3	54361.88	40
8245	line*coccidia	2	2	35520.67	54361.88	40
8245	line*coccidia	2	3	18881.67	54361.88	40
8301	line	1		227444.5	45018.13	40
8301	line	2		241539.6	45018.13	40
8301	coccidia		0	238179.2	63665.26	40
8301	coccidia		1	341414.5	63665.26	40
8301	coccidia		2	123096.9	63665.26	40
8301	coccidia		3	235277.8	63665.26	40
8301	line*coccidia	1	0	199917.7	90036.27	40
8301	line*coccidia	1	1	436850.5	90036.27	40
8301	line*coccidia	1	2	43063.5	90036.27	40
8301	line*coccidia	1	3	229946.5	90036.27	40
8301	line*coccidia	2	0	276440.7	90036.27	40
8301	line*coccidia	2	1	245978.5	90036.27	40
8301	line*coccidia	2	2	203130.3	90036.27	40
8301	line*coccidia	2	3	240609	90036.27	40
8311	line	1		679766.8	186638.7	40
8311	line	2		178411.3	186638.7	40
8311	coccidia		0	210404.8	263947	40
8311	coccidia		1	1189310	263947	40
8311	coccidia		2	146517.7	263947	40
8311	coccidia		3	170123.4	263947	40
8311	line*coccidia	1	0	209282.3	373277.4	40
8311	line*coccidia	1	1	2175206	373277.4	40
8311	line*coccidia	1	2	119737.5	373277.4	40
8311	line*coccidia	1	3	214841.2	373277.4	40
8311	line*coccidia	2	0	211527.3	373277.4	40
8311	line*coccidia	2	1	203414.2	373277.4	40
8311	line*coccidia	2	2	173297.8	373277.4	40
8311	line*coccidia	2	3	125405.7	373277.4	40
8313	line	1		258422.6	46212.7	40
8313	line	2		183246.8	46212.7	40
8313	coccidia		0	184408.6	65354.63	40
8313	coccidia		1	349629.8	65354.63	40
8313	coccidia		2	167668.5	65354.63	40
8313	coccidia		3	181631.8	65354.63	40
8313	line*coccidia	1	0	195267.2	92425.41	40
8313	line*coccidia	1	1	497068.7	92425.41	40
8313	line*coccidia	1	2	45120.17	92425.41	40
8313	line*coccidia	1	3	296234.3	92425.41	40
8313	line*coccidia	2	0	173550	92425.41	40
8313	line*coccidia	2	1	202190.8	92425.41	40
8313	line*coccidia	2	2	290216.8	92425.41	40
8313	line*coccidia	2	3	67029.33	92425.41	40
8323	line	1		279015.4	69720.19	40
8323	line	2		168748.5	69720.19	40

8323	coccidia		0	164822.6	98599.23	40
8323	coccidia		1	488000.3	98599.23	40
8323	coccidia		2	223444.4	98599.23	40
8323	coccidia		3	19260.67	98599.23	40
8323	line*coccidia	1	0	109280.3	139440.4	40
8323	line*coccidia	1	1	787485.7	139440.4	40
8323	line*coccidia	1	2	180774.3	139440.4	40
8323	line*coccidia	1	3	38521.33	139440.4	40
8323	line*coccidia	2	0	220364.8	139440.4	40
8323	line*coccidia	2	1	188514.8	139440.4	40
8323	line*coccidia	2	2	266114.5	139440.4	40
8323	line*coccidia	2	3	2.55E-11	139440.4	40
8330	line	1		439897.1	115533.4	40
8330	line	2		-9.1E-12	115533.4	40
8330	coccidia		0	49367.58	163388.9	40
8330	coccidia		1	685243.7	163388.9	40
8330	coccidia		2	55453.67	163388.9	40
8330	coccidia		3	89729.33	163388.9	40
8330	line*coccidia	1	0	98735.17	231066.8	40
8330	line*coccidia	1	1	1370487	231066.8	40
8330	line*coccidia	1	2	110907.3	231066.8	40
8330	line*coccidia	1	3	179458.7	231066.8	40
8330	line*coccidia	2	0	0	231066.8	40
8330	line*coccidia	2	1	-1.2E-10	231066.8	40
8330	line*coccidia	2	2	4E-11	231066.8	40
8330	line*coccidia	2	3	4E-11	231066.8	40
8331	line	1		319000.8	67657.05	40
8331	line	2		4.55E-12	67657.05	40
8331	coccidia		0	105789.8	95681.52	40
8331	coccidia		1	335129.2	95681.52	40
8331	coccidia		2	72318.17	95681.52	40
8331	coccidia		3	124764.6	95681.52	40
8331	line*coccidia	1	0	211579.5	135314.1	40
8331	line*coccidia	1	1	670258.3	135314.1	40
8331	line*coccidia	1	2	144636.3	135314.1	40
8331	line*coccidia	1	3	249529.2	135314.1	40
8331	line*coccidia	2	0	7.28E-12	135314.1	40
8331	line*coccidia	2	1	3.64E-12	135314.1	40
8331	line*coccidia	2	2	3.64E-12	135314.1	40
8331	line*coccidia	2	3	3.64E-12	135314.1	40
8333	line	1		301187.7	58953.99	40
8333	line	2		293341.9	58953.99	40
8333	coccidia		0	222490.8	83373.53	40
8333	coccidia		1	358997.3	83373.53	40
8333	coccidia		2	371931.1	83373.53	40
8333	coccidia		3	235639.9	83373.53	40
8333	line*coccidia	1	0	234784.5	117908	40
8333	line*coccidia	1	1	492151.2	117908	40
8333	line*coccidia	1	2	128943.8	117908	40

8333	line*coccidia	1	3	348871.2	117908	40
8333	line*coccidia	2	0	210197	117908	40
8333	line*coccidia	2	1	225843.5	117908	40
8333	line*coccidia	2	2	614918.3	117908	40
8333	line*coccidia	2	3	122408.7	117908	40
8334	line	1		64000.5	19323.63	40
8334	line	2		52997.83	19323.63	40
8334	coccidia		0	40843.42	27327.74	40
8334	coccidia		1	104258.3	27327.74	40
8334	coccidia		2	37484.08	27327.74	40
8334	coccidia		3	51410.92	27327.74	40
8334	line*coccidia	1	0	23397.17	38647.27	40
8334	line*coccidia	1	1	94503.33	38647.27	40
8334	line*coccidia	1	2	35279.67	38647.27	40
8334	line*coccidia	1	3	102821.8	38647.27	40
8334	line*coccidia	2	0	58289.67	38647.27	40
8334	line*coccidia	2	1	114013.2	38647.27	40
8334	line*coccidia	2	2	39688.5	38647.27	40
8334	line*coccidia	2	3	-1.5E-11	38647.27	40
8402	line	1		2121766	463297.3	40
8402	line	2		2440069	463297.3	40
8402	coccidia		0	2807509	655201.3	40
8402	coccidia		1	2521002	655201.3	40
8402	coccidia		2	834101.3	655201.3	40
8402	coccidia		3	2961056	655201.3	40
8402	line*coccidia	1	0	1874274	926594.6	40
8402	line*coccidia	1	1	3012361	926594.6	40
8402	line*coccidia	1	2	182773.5	926594.6	40
8402	line*coccidia	1	3	3417655	926594.6	40
8402	line*coccidia	2	0	3740745	926594.6	40
8402	line*coccidia	2	1	2029644	926594.6	40
8402	line*coccidia	2	2	1485429	926594.6	40
8402	line*coccidia	2	3	2504458	926594.6	40
8403	line	1		1031914	261828.9	40
8403	line	2		313638.2	261828.9	40
8403	coccidia		0	443478.4	370282	40
8403	coccidia		1	1405813	370282	40
8403	coccidia		2	355115.8	370282	40
8403	coccidia		3	486697.3	370282	40
8403	line*coccidia	1	0	567079	523657.9	40
8403	line*coccidia	1	1	2577810	523657.9	40
8403	line*coccidia	1	2	455264.8	523657.9	40
8403	line*coccidia	1	3	527503.3	523657.9	40
8403	line*coccidia	2	0	319877.8	523657.9	40
8403	line*coccidia	2	1	233817.2	523657.9	40
8403	line*coccidia	2	2	254966.7	523657.9	40
8403	line*coccidia	2	3	445891.2	523657.9	40
8411	line	1		1018933	258745.4	40
8411	line	2		236568.9	258745.4	40

8411	coccidia		0	252839.4	365921.2	40
8411	coccidia		1	1526304	365921.2	40
8411	coccidia		2	445453.3	365921.2	40
8411	coccidia		3	286407.2	365921.2	40
8411	line*coccidia	1	0	347116	517490.7	40
8411	line*coccidia	1	1	2750819	517490.7	40
8411	line*coccidia	1	2	552424.3	517490.7	40
8411	line*coccidia	1	3	425373.2	517490.7	40
8411	line*coccidia	2	0	158562.8	517490.7	40
8411	line*coccidia	2	1	301789.3	517490.7	40
8411	line*coccidia	2	2	338482.3	517490.7	40
8411	line*coccidia	2	3	147441.2	517490.7	40
8415	line	1		2283540	647084.5	40
8415	line	2		465441.1	647084.5	40
8415	coccidia		0	395996.7	915115.6	40
8415	coccidia		1	4052206	915115.6	40
8415	coccidia		2	671321.3	915115.6	40
8415	coccidia		3	378437.8	915115.6	40
8415	line*coccidia	1	0	574457	1294169	40
8415	line*coccidia	1	1	7835586	1294169	40
8415	line*coccidia	1	2	138341.8	1294169	40
8415	line*coccidia	1	3	585773.5	1294169	40
8415	line*coccidia	2	0	217536.3	1294169	40
8415	line*coccidia	2	1	268825.2	1294169	40
8415	line*coccidia	2	2	1204301	1294169	40
8415	line*coccidia	2	3	171102.2	1294169	40
8417	line	1		552705.4	106297.8	40
8417	line	2		354577.3	106297.8	40
8417	coccidia		0	315666.9	150327.8	40
8417	coccidia		1	760150.8	150327.8	40
8417	coccidia		2	312602.9	150327.8	40
8417	coccidia		3	426144.8	150327.8	40
8417	line*coccidia	1	0	398047.8	212595.7	40
8417	line*coccidia	1	1	1211333	212595.7	40
8417	line*coccidia	1	2	193693	212595.7	40
8417	line*coccidia	1	3	407748.2	212595.7	40
8417	line*coccidia	2	0	233286	212595.7	40
8417	line*coccidia	2	1	308969	212595.7	40
8417	line*coccidia	2	2	431512.8	212595.7	40
8417	line*coccidia	2	3	444541.3	212595.7	40
8419	line	1		382714.6	81129.54	40
8419	line	2		140856.7	81129.54	40
8419	coccidia		0	61372.58	114734.5	40
8419	coccidia		1	716432.3	114734.5	40
8419	coccidia		2	181794.4	114734.5	40
8419	coccidia		3	87543.33	114734.5	40
8419	line*coccidia	1	0	45737.67	162259.1	40
8419	line*coccidia	1	1	1147632	162259.1	40
8419	line*coccidia	1	2	297151.8	162259.1	40

8419	line*coccidia	1	3	40337.33	162259.1	40
8419	line*coccidia	2	0	77007.5	162259.1	40
8419	line*coccidia	2	1	285233	162259.1	40
8419	line*coccidia	2	2	66437	162259.1	40
8419	line*coccidia	2	3	134749.3	162259.1	40
8420	line	1		118584.2	39038.51	40
8420	line	2		118327.2	39038.51	40
8420	coccidia		0	106836.8	55208.8	40
8420	coccidia		1	296780.1	55208.8	40
8420	coccidia		2	20317.5	55208.8	40
8420	coccidia		3	49888.42	55208.8	40
8420	line*coccidia	1	0	110145.7	78077.03	40
8420	line*coccidia	1	1	364191	78077.03	40
8420	line*coccidia	1	2	1.46E-11	78077.03	40
8420	line*coccidia	1	3	1.46E-11	78077.03	40
8420	line*coccidia	2	0	103527.8	78077.03	40
8420	line*coccidia	2	1	229369.2	78077.03	40
8420	line*coccidia	2	2	40635	78077.03	40
8420	line*coccidia	2	3	99776.83	78077.03	40
8421	line	1		-5.1E-11	88875.36	40
8421	line	2		271583.9	88875.36	40
8421	coccidia		0	299249.5	125688.7	40
8421	coccidia		1	58250.75	125688.7	40
8421	coccidia		2	22060.42	125688.7	40
8421	coccidia		3	163607.1	125688.7	40
8421	line*coccidia	1	0	-5.8E-11	177750.7	40
8421	line*coccidia	1	1	-2.9E-11	177750.7	40
8421	line*coccidia	1	2	-5.8E-11	177750.7	40
8421	line*coccidia	1	3	-5.8E-11	177750.7	40
8421	line*coccidia	2	0	598499	177750.7	40
8421	line*coccidia	2	1	116501.5	177750.7	40
8421	line*coccidia	2	2	44120.83	177750.7	40
8421	line*coccidia	2	3	327214.2	177750.7	40
8426	line	1		17264728	2697475	40
8426	line	2		17985900	2697475	40
8426	coccidia		0	30746102	3814806	40
8426	coccidia		1	1690299	3814806	40
8426	coccidia		2	11117574	3814806	40
8426	coccidia		3	26947279	3814806	40
8426	line*coccidia	1	0	43792363	5394951	40
8426	line*coccidia	1	1	2820116	5394951	40
8426	line*coccidia	1	2	2575277	5394951	40
8426	line*coccidia	1	3	19871155	5394951	40
8426	line*coccidia	2	0	17699842	5394951	40
8426	line*coccidia	2	1	560482.8	5394951	40
8426	line*coccidia	2	2	19659870	5394951	40
8426	line*coccidia	2	3	34023403	5394951	40
8429	line	1		876055.1	204766.8	40
8429	line	2		1080231	204766.8	40

8429	coccidia		0	873270.5	289583.9	40
8429	coccidia		1	273298.1	289583.9	40
8429	coccidia		2	1002829	289583.9	40
8429	coccidia		3	1763173	289583.9	40
8429	line*coccidia	1	0	1387612	409533.5	40
8429	line*coccidia	1	1	153256.5	409533.5	40
8429	line*coccidia	1	2	429973.7	409533.5	40
8429	line*coccidia	1	3	1533378	409533.5	40
8429	line*coccidia	2	0	358928.7	409533.5	40
8429	line*coccidia	2	1	393339.7	409533.5	40
8429	line*coccidia	2	2	1575685	409533.5	40
8429	line*coccidia	2	3	1992969	409533.5	40
8433	line	1		1227415	277038.1	40
8433	line	2		277037.8	277038.1	40
8433	coccidia		0	344666.4	391791.1	40
8433	coccidia		1	1733608	391791.1	40
8433	coccidia		2	477305	391791.1	40
8433	coccidia		3	453326.6	391791.1	40
8433	line*coccidia	1	0	483386.7	554076.2	40
8433	line*coccidia	1	1	3082691	554076.2	40
8433	line*coccidia	1	2	609108.2	554076.2	40
8433	line*coccidia	1	3	734474.5	554076.2	40
8433	line*coccidia	2	0	205946.2	554076.2	40
8433	line*coccidia	2	1	384524.5	554076.2	40
8433	line*coccidia	2	2	345501.8	554076.2	40
8433	line*coccidia	2	3	172178.7	554076.2	40
8434	line	1		1247178	657169	40
8434	line	2		846213	657169	40
8434	coccidia		0	293394.3	929377.3	40
8434	coccidia		1	785226	929377.3	40
8434	coccidia		2	2626190	929377.3	40
8434	coccidia		3	481972.5	929377.3	40
8434	line*coccidia	1	0	325280.5	1314338	40
8434	line*coccidia	1	1	552713	1314338	40
8434	line*coccidia	1	2	3764956	1314338	40
8434	line*coccidia	1	3	345763.8	1314338	40
8434	line*coccidia	2	0	261508.2	1314338	40
8434	line*coccidia	2	1	1017739	1314338	40
8434	line*coccidia	2	2	1487424	1314338	40
8434	line*coccidia	2	3	618181.2	1314338	40
8439	line	1		176439.8	58642.58	40
8439	line	2		1.33E-11	58642.58	40
8439	coccidia		0	1.46E-11	82933.14	40
8439	coccidia		1	352879.6	82933.14	40
8439	coccidia		2	2.91E-11	82933.14	40
8439	coccidia		3	2.91E-11	82933.14	40
8439	line*coccidia	1	0	1.46E-11	117285.2	40
8439	line*coccidia	1	1	705759.2	117285.2	40
8439	line*coccidia	1	2	2.59E-11	117285.2	40

8439	line*coccidia	1	3	2.59E-11	117285.2	40
8439	line*coccidia	2	0	1.46E-11	117285.2	40
8439	line*coccidia	2	1	-2.6E-11	117285.2	40
8439	line*coccidia	2	2	3.23E-11	117285.2	40
8439	line*coccidia	2	3	3.23E-11	117285.2	40
8440	line	1		140251.5	44838.92	40
8440	line	2		1.43E-11	44838.92	40
8440	coccidia		0	1.46E-11	63411.8	40
8440	coccidia		1	280503.1	63411.8	40
8440	coccidia		2	2.91E-11	63411.8	40
8440	coccidia		3	2.91E-11	63411.8	40
8440	line*coccidia	1	0	1.46E-11	89677.83	40
8440	line*coccidia	1	1	561006.2	89677.83	40
8440	line*coccidia	1	2	4.4E-11	89677.83	40
8440	line*coccidia	1	3	5.19E-12	89677.83	40
8440	line*coccidia	2	0	1.46E-11	89677.83	40
8440	line*coccidia	2	1	-2.5E-11	89677.83	40
8440	line*coccidia	2	2	1.42E-11	89677.83	40
8440	line*coccidia	2	3	5.3E-11	89677.83	40
8441	line	1		142598.9	53780.83	40
8441	line	2		-1.1E-11	53780.83	40
8441	coccidia		0	-1.5E-11	76057.58	40
8441	coccidia		1	285197.8	76057.58	40
8441	coccidia		2	0	76057.58	40
8441	coccidia		3	0	76057.58	40
8441	line*coccidia	1	0	-1.5E-11	107561.7	40
8441	line*coccidia	1	1	570395.5	107561.7	40
8441	line*coccidia	1	2	1.01E-11	107561.7	40
8441	line*coccidia	1	3	1.01E-11	107561.7	40
8441	line*coccidia	2	0	-1.5E-11	107561.7	40
8441	line*coccidia	2	1	-1E-11	107561.7	40
8441	line*coccidia	2	2	-1E-11	107561.7	40
8441	line*coccidia	2	3	-1E-11	107561.7	40
8442	line	1		513794	141829.1	40
8442	line	2		-7.3E-12	141829.1	40
8442	coccidia		0	180275.3	200576.6	40
8442	coccidia		1	555756.9	200576.6	40
8442	coccidia		2	63974.42	200576.6	40
8442	coccidia		3	227581.3	200576.6	40
8442	line*coccidia	1	0	360550.5	283658.2	40
8442	line*coccidia	1	1	1111514	283658.2	40
8442	line*coccidia	1	2	127948.8	283658.2	40
8442	line*coccidia	1	3	455162.7	283658.2	40
8442	line*coccidia	2	0	0	283658.2	40
8442	line*coccidia	2	1	2.91E-11	283658.2	40
8442	line*coccidia	2	2	-1.5E-11	283658.2	40
8442	line*coccidia	2	3	-4.4E-11	283658.2	40
8445	line	1		514264.8	268737	40
8445	line	2		972669.7	268737	40

8445	coccidia		0	476639	380051.5	40
8445	coccidia		1	209563.6	380051.5	40
8445	coccidia		2	894986.2	380051.5	40
8445	coccidia		3	1392680	380051.5	40
8445	line*coccidia	1	0	621070.3	537473.9	40
8445	line*coccidia	1	1	122899.7	537473.9	40
8445	line*coccidia	1	2	350977.8	537473.9	40
8445	line*coccidia	1	3	962111.2	537473.9	40
8445	line*coccidia	2	0	332207.7	537473.9	40
8445	line*coccidia	2	1	296227.5	537473.9	40
8445	line*coccidia	2	2	1438995	537473.9	40
8445	line*coccidia	2	3	1823249	537473.9	40
8446	line	1		1335421	650537.1	40
8446	line	2		1795381	650537.1	40
8446	coccidia		0	1779353	919998.4	40
8446	coccidia		1	854576.8	919998.4	40
8446	coccidia		2	1797785	919998.4	40
8446	coccidia		3	1829888	919998.4	40
8446	line*coccidia	1	0	691765.7	1301074	40
8446	line*coccidia	1	1	517549	1301074	40
8446	line*coccidia	1	2	3327948	1301074	40
8446	line*coccidia	1	3	804420.7	1301074	40
8446	line*coccidia	2	0	2866940	1301074	40
8446	line*coccidia	2	1	1191605	1301074	40
8446	line*coccidia	2	2	267623.2	1301074	40
8446	line*coccidia	2	3	2855355	1301074	40
8528	line	1		3936500	1054994	40
8528	line	2		5248053	1054994	40
8528	coccidia		0	6269409	1491987	40
8528	coccidia		1	2585575	1491987	40
8528	coccidia		2	4693308	1491987	40
8528	coccidia		3	4820815	1491987	40
8528	line*coccidia	1	0	5715579	2109988	40
8528	line*coccidia	1	1	2916961	2109988	40
8528	line*coccidia	1	2	3286144	2109988	40
8528	line*coccidia	1	3	3827317	2109988	40
8528	line*coccidia	2	0	6823238	2109988	40
8528	line*coccidia	2	1	2254189	2109988	40
8528	line*coccidia	2	2	6100473	2109988	40
8528	line*coccidia	2	3	5814314	2109988	40
8547	line	1		16700392	2197290	40
8547	line	2		13398218	2197290	40
8547	coccidia		0	16352625	3107437	40
8547	coccidia		1	10165434	3107437	40
8547	coccidia		2	17870593	3107437	40
8547	coccidia		3	15808568	3107437	40
8547	line*coccidia	1	0	15408752	4394580	40
8547	line*coccidia	1	1	9837217	4394580	40
8547	line*coccidia	1	2	18070133	4394580	40

8547	line*coccidia	1	3	23485464	4394580	40
8547	line*coccidia	2	0	17296498	4394580	40
8547	line*coccidia	2	1	10493650	4394580	40
8547	line*coccidia	2	2	17671054	4394580	40
8547	line*coccidia	2	3	8131671	4394580	40
8549	line	1		2942232	1028380	40
8549	line	2		6952093	1028380	40
8549	coccidia		0	3160871	1454348	40
8549	coccidia		1	3579980	1454348	40
8549	coccidia		2	4932147	1454348	40
8549	coccidia		3	8115651	1454348	40
8549	line*coccidia	1	0	1829011	2056759	40
8549	line*coccidia	1	1	2742626	2056759	40
8549	line*coccidia	1	2	766804.7	2056759	40
8549	line*coccidia	1	3	6430485	2056759	40
8549	line*coccidia	2	0	4492731	2056759	40
8549	line*coccidia	2	1	4417333	2056759	40
8549	line*coccidia	2	2	9097490	2056759	40
8549	line*coccidia	2	3	9800818	2056759	40
8550	line	1		1777514	323261.2	40
8550	line	2		1875050	323261.2	40
8550	coccidia		0	1769401	457160.4	40
8550	coccidia		1	1549405	457160.4	40
8550	coccidia		2	1539302	457160.4	40
8550	coccidia		3	2447020	457160.4	40
8550	line*coccidia	1	0	2125520	646522.4	40
8550	line*coccidia	1	1	1128994	646522.4	40
8550	line*coccidia	1	2	297819.7	646522.4	40
8550	line*coccidia	1	3	3557721	646522.4	40
8550	line*coccidia	2	0	1413282	646522.4	40
8550	line*coccidia	2	1	1969816	646522.4	40
8550	line*coccidia	2	2	2780785	646522.4	40
8550	line*coccidia	2	3	1336319	646522.4	40
8557	line	1		11411313	3790063	40
8557	line	2		13146971	3790063	40
8557	coccidia		0	10297362	5359959	40
8557	coccidia		1	6418686	5359959	40
8557	coccidia		2	20448711	5359959	40
8557	coccidia		3	11951809	5359959	40
8557	line*coccidia	1	0	5531751	7580126	40
8557	line*coccidia	1	1	2788642	7580126	40
8557	line*coccidia	1	2	28226076	7580126	40
8557	line*coccidia	1	3	9098782	7580126	40
8557	line*coccidia	2	0	15062973	7580126	40
8557	line*coccidia	2	1	10048730	7580126	40
8557	line*coccidia	2	2	12671346	7580126	40
8557	line*coccidia	2	3	14804835	7580126	40
8560	line	1		30114494	5254424	40
8560	line	2		34758528	5254424	40

8560	coccidia		0	51404511	7430877	40
8560	coccidia		1	4926704	7430877	40
8560	coccidia		2	25721585	7430877	40
8560	coccidia		3	47693242	7430877	40
8560	line*coccidia	1	0	59951579	10508847	40
8560	line*coccidia	1	1	6066094	10508847	40
8560	line*coccidia	1	2	6771753	10508847	40
8560	line*coccidia	1	3	47668548	10508847	40
8560	line*coccidia	2	0	42857444	10508847	40
8560	line*coccidia	2	1	3787313	10508847	40
8560	line*coccidia	2	2	44671418	10508847	40
8560	line*coccidia	2	3	47717937	10508847	40
8561	line	1		21217766	3966100	40
8561	line	2		20771979	3966100	40
8561	coccidia		0	33498156	5608913	40
8561	coccidia		1	3389813	5608913	40
8561	coccidia		2	24744173	5608913	40
8561	coccidia		3	22347347	5608913	40
8561	line*coccidia	1	0	36352307	7932201	40
8561	line*coccidia	1	1	2831042	7932201	40
8561	line*coccidia	1	2	15467670	7932201	40
8561	line*coccidia	1	3	30220044	7932201	40
8561	line*coccidia	2	0	30644004	7932201	40
8561	line*coccidia	2	1	3948584	7932201	40
8561	line*coccidia	2	2	34020676	7932201	40
8561	line*coccidia	2	3	14474651	7932201	40
8563	line	1		5131773	1078737	40
8563	line	2		7517106	1078737	40
8563	coccidia		0	5527337	1525564	40
8563	coccidia		1	5144001	1525564	40
8563	coccidia		2	7113553	1525564	40
8563	coccidia		3	7512869	1525564	40
8563	line*coccidia	1	0	4029163	2157474	40
8563	line*coccidia	1	1	5327692	2157474	40
8563	line*coccidia	1	2	3819568	2157474	40
8563	line*coccidia	1	3	7350671	2157474	40
8563	line*coccidia	2	0	7025511	2157474	40
8563	line*coccidia	2	1	4960310	2157474	40
8563	line*coccidia	2	2	10407539	2157474	40
8563	line*coccidia	2	3	7675066	2157474	40
8567	line	1		876298.5	341985.7	40
8567	line	2		1013523	341985.7	40
8567	coccidia		0	1640868	483640.9	40
8567	coccidia		1	1366887	483640.9	40
8567	coccidia		2	614279.3	483640.9	40
8567	coccidia		3	157608.2	483640.9	40
8567	line*coccidia	1	0	621895.3	683971.5	40
8567	line*coccidia	1	1	1950280	683971.5	40
8567	line*coccidia	1	2	933018.3	683971.5	40

8567	line*coccidia	1	3	5.82E-11	683971.5	40
8567	line*coccidia	2	0	2659840	683971.5	40
8567	line*coccidia	2	1	783494	683971.5	40
8567	line*coccidia	2	2	295540.2	683971.5	40
8567	line*coccidia	2	3	315216.3	683971.5	40
8569	line	1		2666707	678377.6	40
8569	line	2		2185478	678377.6	40
8569	coccidia		0	1979164	959370.8	40
8569	coccidia		1	4008425	959370.8	40
8569	coccidia		2	695414.7	959370.8	40
8569	coccidia		3	3021367	959370.8	40
8569	line*coccidia	1	0	1407942	1356755	40
8569	line*coccidia	1	1	7385285	1356755	40
8569	line*coccidia	1	2	489193.3	1356755	40
8569	line*coccidia	1	3	1384408	1356755	40
8569	line*coccidia	2	0	2550385	1356755	40
8569	line*coccidia	2	1	631564.5	1356755	40
8569	line*coccidia	2	2	901636	1356755	40
8569	line*coccidia	2	3	4658326	1356755	40
8570	line	1		6253686	1450186	40
8570	line	2		6222282	1450186	40
8570	coccidia		0	9933174	2050872	40
8570	coccidia		1	2151167	2050872	40
8570	coccidia		2	6243523	2050872	40
8570	coccidia		3	6624072	2050872	40
8570	line*coccidia	1	0	13217228	2900371	40
8570	line*coccidia	1	1	2221673	2900371	40
8570	line*coccidia	1	2	4390073	2900371	40
8570	line*coccidia	1	3	5185769	2900371	40
8570	line*coccidia	2	0	6649120	2900371	40
8570	line*coccidia	2	1	2080661	2900371	40
8570	line*coccidia	2	2	8096972	2900371	40
8570	line*coccidia	2	3	8062374	2900371	40
8620	line	1		1811630	461595.1	40
8620	line	2		2364387	461595.1	40
8620	coccidia		0	2085434	652794.1	40
8620	coccidia		1	2531845	652794.1	40
8620	coccidia		2	2055612	652794.1	40
8620	coccidia		3	1679143	652794.1	40
8620	line*coccidia	1	0	1069030	923190.2	40
8620	line*coccidia	1	1	2763602	923190.2	40
8620	line*coccidia	1	2	1887562	923190.2	40
8620	line*coccidia	1	3	1526323	923190.2	40
8620	line*coccidia	2	0	3101838	923190.2	40
8620	line*coccidia	2	1	2300087	923190.2	40
8620	line*coccidia	2	2	2223662	923190.2	40
8620	line*coccidia	2	3	1831962	923190.2	40
8622	line	1		2231122	854593.5	40
8622	line	2		4842979	854593.5	40

8622	coccidia		0	3085683	1208578	40
8622	coccidia		1	4842881	1208578	40
8622	coccidia		2	1897287	1208578	40
8622	coccidia		3	4322350	1208578	40
8622	line*coccidia	1	0	2265663	1709187	40
8622	line*coccidia	1	1	533803.5	1709187	40
8622	line*coccidia	1	2	1530883	1709187	40
8622	line*coccidia	1	3	4594139	1709187	40
8622	line*coccidia	2	0	3905704	1709187	40
8622	line*coccidia	2	1	9151959	1709187	40
8622	line*coccidia	2	2	2263691	1709187	40
8622	line*coccidia	2	3	4050562	1709187	40
8623	line	1		3270053	1161882	40
8623	line	2		4880137	1161882	40
8623	coccidia		0	7067044	1643150	40
8623	coccidia		1	2024473	1643150	40
8623	coccidia		2	1379199	1643150	40
8623	coccidia		3	5829664	1643150	40
8623	line*coccidia	1	0	6330744	2323765	40
8623	line*coccidia	1	1	496471	2323765	40
8623	line*coccidia	1	2	1233968	2323765	40
8623	line*coccidia	1	3	5019028	2323765	40
8623	line*coccidia	2	0	7803344	2323765	40
8623	line*coccidia	2	1	3552475	2323765	40
8623	line*coccidia	2	2	1524429	2323765	40
8623	line*coccidia	2	3	6640300	2323765	40
8632	line	1		16929656	2543812	40
8632	line	2		15417337	2543812	40
8632	coccidia		0	21255505	3597494	40
8632	coccidia		1	3587960	3597494	40
8632	coccidia		2	13721198	3597494	40
8632	coccidia		3	26129323	3597494	40
8632	line*coccidia	1	0	24755224	5087624	40
8632	line*coccidia	1	1	1880673	5087624	40
8632	line*coccidia	1	2	4406733	5087624	40
8632	line*coccidia	1	3	36675992	5087624	40
8632	line*coccidia	2	0	17755787	5087624	40
8632	line*coccidia	2	1	5295247	5087624	40
8632	line*coccidia	2	2	23035663	5087624	40
8632	line*coccidia	2	3	15582653	5087624	40
8721	line	1		1508472	304668.9	40
8721	line	2		1967497	304668.9	40
8721	coccidia		0	2478456	430866.9	40
8721	coccidia		1	603261.2	430866.9	40
8721	coccidia		2	1295078	430866.9	40
8721	coccidia		3	2575143	430866.9	40
8721	line*coccidia	1	0	3255051	609337.8	40
8721	line*coccidia	1	1	44801.67	609337.8	40
8721	line*coccidia	1	2	396626.2	609337.8	40

8721	line*coccidia	1	3	2337408	609337.8	40
8721	line*coccidia	2	0	1701861	609337.8	40
8721	line*coccidia	2	1	1161721	609337.8	40
8721	line*coccidia	2	2	2193531	609337.8	40
8721	line*coccidia	2	3	2812877	609337.8	40
8722	line	1		375792.5	101116.3	40
8722	line	2		574575.1	101116.3	40
8722	coccidia		0	682802.6	143000.1	40
8722	coccidia		1	498773.1	143000.1	40
8722	coccidia		2	210121.3	143000.1	40
8722	coccidia		3	509038.1	143000.1	40
8722	line*coccidia	1	0	951734.8	202232.6	40
8722	line*coccidia	1	1	26074.83	202232.6	40
8722	line*coccidia	1	2	114956.2	202232.6	40
8722	line*coccidia	1	3	410404	202232.6	40
8722	line*coccidia	2	0	413870.3	202232.6	40
8722	line*coccidia	2	1	971471.3	202232.6	40
8722	line*coccidia	2	2	305286.5	202232.6	40
8722	line*coccidia	2	3	607672.2	202232.6	40
8725	line	1		1559514	254035.6	40
8725	line	2		1408205	254035.6	40
8725	coccidia		0	2607635	359260.6	40
8725	coccidia		1	900593	359260.6	40
8725	coccidia		2	832026.3	359260.6	40
8725	coccidia		3	1595183	359260.6	40
8725	line*coccidia	1	0	3257738	508071.2	40
8725	line*coccidia	1	1	884382.8	508071.2	40
8725	line*coccidia	1	2	562932.2	508071.2	40
8725	line*coccidia	1	3	1533003	508071.2	40
8725	line*coccidia	2	0	1957533	508071.2	40
8725	line*coccidia	2	1	916803.2	508071.2	40
8725	line*coccidia	2	2	1101121	508071.2	40
8725	line*coccidia	2	3	1657363	508071.2	40
8735	line	1		156632.2	73804.64	40
8735	line	2		365446.1	73804.64	40
8735	coccidia		0	490975.8	104375.5	40
8735	coccidia		1	239681.1	104375.5	40
8735	coccidia		2	86771.17	104375.5	40
8735	coccidia		3	226728.6	104375.5	40
8735	line*coccidia	1	0	372981.8	147609.3	40
8735	line*coccidia	1	1	47966	147609.3	40
8735	line*coccidia	1	2	109575.2	147609.3	40
8735	line*coccidia	1	3	96005.83	147609.3	40
8735	line*coccidia	2	0	608969.7	147609.3	40
8735	line*coccidia	2	1	431396.2	147609.3	40
8735	line*coccidia	2	2	63967.17	147609.3	40
8735	line*coccidia	2	3	357451.3	147609.3	40
8738	line	1		6978694	782266.4	40
8738	line	2		7873379	782266.4	40

8738	coccidia		0	10615797	1106292	40
8738	coccidia		1	1601869	1106292	40
8738	coccidia		2	6834921	1106292	40
8738	coccidia		3	10651558	1106292	40
8738	line*coccidia	1	0	11113793	1564533	40
8738	line*coccidia	1	1	854284.7	1564533	40
8738	line*coccidia	1	2	2256025	1564533	40
8738	line*coccidia	1	3	13690675	1564533	40
8738	line*coccidia	2	0	10117801	1564533	40
8738	line*coccidia	2	1	2349453	1564533	40
8738	line*coccidia	2	2	11413818	1564533	40
8738	line*coccidia	2	3	7612441	1564533	40
8739	line	1		6857081	997734.7	40
8739	line	2		6280034	997734.7	40
8739	coccidia		0	8401834	1411010	40
8739	coccidia		1	878203.3	1411010	40
8739	coccidia		2	7818657	1411010	40
8739	coccidia		3	9175535	1411010	40
8739	line*coccidia	1	0	11355235	1995469	40
8739	line*coccidia	1	1	334115.5	1995469	40
8739	line*coccidia	1	2	3387697	1995469	40
8739	line*coccidia	1	3	12351275	1995469	40
8739	line*coccidia	2	0	5448434	1995469	40
8739	line*coccidia	2	1	1422291	1995469	40
8739	line*coccidia	2	2	12249616	1995469	40
8739	line*coccidia	2	3	5999795	1995469	40
8818	line	1		365160.1	153176.4	40
8818	line	2		722932	153176.4	40
8818	coccidia		0	764163.8	216624.1	40
8818	coccidia		1	224749.8	216624.1	40
8818	coccidia		2	331894.6	216624.1	40
8818	coccidia		3	855376	216624.1	40
8818	line*coccidia	1	0	898954.2	306352.7	40
8818	line*coccidia	1	1	27192.67	306352.7	40
8818	line*coccidia	1	2	78952.83	306352.7	40
8818	line*coccidia	1	3	455540.8	306352.7	40
8818	line*coccidia	2	0	629373.5	306352.7	40
8818	line*coccidia	2	1	422307	306352.7	40
8818	line*coccidia	2	2	584836.3	306352.7	40
8818	line*coccidia	2	3	1255211	306352.7	40
8833	line	1		5782168	1370781	40
8833	line	2		9081849	1370781	40
8833	coccidia		0	10671868	1938578	40
8833	coccidia		1	1827800	1938578	40
8833	coccidia		2	6473127	1938578	40
8833	coccidia		3	10755240	1938578	40
8833	line*coccidia	1	0	11325419	2741563	40
8833	line*coccidia	1	1	211754.7	2741563	40
8833	line*coccidia	1	2	2723659	2741563	40

8833	line*coccidia	1	3	8867840	2741563	40
8833	line*coccidia	2	0	10018316	2741563	40
8833	line*coccidia	2	1	3443846	2741563	40
8833	line*coccidia	2	2	10222595	2741563	40
8833	line*coccidia	2	3	12642640	2741563	40
8834	line	1		9746446	1614382	40
8834	line	2		10267672	1614382	40
8834	coccidia		0	14102798	2283081	40
8834	coccidia		1	966297.5	2283081	40
8834	coccidia		2	10517534	2283081	40
8834	coccidia		3	14441607	2283081	40
8834	line*coccidia	1	0	10809505	3228764	40
8834	line*coccidia	1	1	385391.3	3228764	40
8834	line*coccidia	1	2	6690370	3228764	40
8834	line*coccidia	1	3	21100519	3228764	40
8834	line*coccidia	2	0	17396091	3228764	40
8834	line*coccidia	2	1	1547204	3228764	40
8834	line*coccidia	2	2	14344698	3228764	40
8834	line*coccidia	2	3	7782695	3228764	40
8835	line	1		3750964	604853.8	40
8835	line	2		2779783	604853.8	40
8835	coccidia		0	3470376	855392.5	40
8835	coccidia		1	651980.2	855392.5	40
8835	coccidia		2	3076567	855392.5	40
8835	coccidia		3	5862571	855392.5	40
8835	line*coccidia	1	0	4074648	1209708	40
8835	line*coccidia	1	1	477611	1209708	40
8835	line*coccidia	1	2	2167466	1209708	40
8835	line*coccidia	1	3	8284132	1209708	40
8835	line*coccidia	2	0	2866105	1209708	40
8835	line*coccidia	2	1	826349.3	1209708	40
8835	line*coccidia	2	2	3985667	1209708	40
8835	line*coccidia	2	3	3441011	1209708	40
8836	line	1		5306916	640623.3	40
8836	line	2		4314832	640623.3	40
8836	coccidia		0	9226282	905978.1	40
8836	coccidia		1	153896.3	905978.1	40
8836	coccidia		2	4596782	905978.1	40
8836	coccidia		3	5266536	905978.1	40
8836	line*coccidia	1	0	12510962	1281247	40
8836	line*coccidia	1	1	139882.3	1281247	40
8836	line*coccidia	1	2	1744827	1281247	40
8836	line*coccidia	1	3	6831993	1281247	40
8836	line*coccidia	2	0	5941602	1281247	40
8836	line*coccidia	2	1	167910.3	1281247	40
8836	line*coccidia	2	2	7448736	1281247	40
8836	line*coccidia	2	3	3701078	1281247	40
8908	line	1		744955.9	271580.4	40
8908	line	2		751501.3	271580.4	40

8908	coccidia		0	1866634	384072.7	40
8908	coccidia		1	76923.42	384072.7	40
8908	coccidia		2	301951.7	384072.7	40
8908	coccidia		3	747405	384072.7	40
8908	line*coccidia	1	0	2022367	543160.8	40
8908	line*coccidia	1	1	22251.17	543160.8	40
8908	line*coccidia	1	2	143618.2	543160.8	40
8908	line*coccidia	1	3	791587	543160.8	40
8908	line*coccidia	2	0	1710901	543160.8	40
8908	line*coccidia	2	1	131595.7	543160.8	40
8908	line*coccidia	2	2	460285.2	543160.8	40
8908	line*coccidia	2	3	703223	543160.8	40
9001	line	1		51931.92	6009.337	40
9001	line	2		45679.63	6009.337	40
9001	coccidia		0	53081.08	8498.487	40
9001	coccidia		1	43519.17	8498.487	40
9001	coccidia		2	47133.75	8498.487	40
9001	coccidia		3	51489.08	8498.487	40
9001	line*coccidia	1	0	47975.83	12018.67	40
9001	line*coccidia	1	1	33686.67	12018.67	40
9001	line*coccidia	1	2	61201.17	12018.67	40
9001	line*coccidia	1	3	64864	12018.67	40
9001	line*coccidia	2	0	58186.33	12018.67	40
9001	line*coccidia	2	1	53351.67	12018.67	40
9001	line*coccidia	2	2	33066.33	12018.67	40
9001	line*coccidia	2	3	38114.17	12018.67	40
9025	line	1		1052438	142989.3	40
9025	line	2		1061138	142989.3	40
9025	coccidia		0	1492459	202217.4	40
9025	coccidia		1	410563.1	202217.4	40
9025	coccidia		2	666473.8	202217.4	40
9025	coccidia		3	1657655	202217.4	40
9025	line*coccidia	1	0	1364003	285978.6	40
9025	line*coccidia	1	1	162738.5	285978.6	40
9025	line*coccidia	1	2	472200.5	285978.6	40
9025	line*coccidia	1	3	2210808	285978.6	40
9025	line*coccidia	2	0	1620915	285978.6	40
9025	line*coccidia	2	1	658387.7	285978.6	40
9025	line*coccidia	2	2	860747.2	285978.6	40
9025	line*coccidia	2	3	1104502	285978.6	40
9026	line	1		1116562	120726	40
9026	line	2		696759.8	120726	40
9026	coccidia		0	1135381	170732.3	40
9026	coccidia		1	567876.7	170732.3	40
9026	coccidia		2	787894.8	170732.3	40
9026	coccidia		3	1135491	170732.3	40
9026	line*coccidia	1	0	1204071	241451.9	40
9026	line*coccidia	1	1	393960.8	241451.9	40
9026	line*coccidia	1	2	859311.8	241451.9	40

9026	line*coccidia	1	3	2008905	241451.9	40
9026	line*coccidia	2	0	1066691	241451.9	40
9026	line*coccidia	2	1	741792.5	241451.9	40
9026	line*coccidia	2	2	716477.8	241451.9	40
9026	line*coccidia	2	3	262078.2	241451.9	40
9027	line	1		468402.1	135505.5	40
9027	line	2		88448.13	135505.5	40
9027	coccidia		0	222738.8	191633.6	40
9027	coccidia		1	557444.1	191633.6	40
9027	coccidia		2	181136.5	191633.6	40
9027	coccidia		3	152381.2	191633.6	40
9027	line*coccidia	1	0	358246.2	271010.9	40
9027	line*coccidia	1	1	1078942	271010.9	40
9027	line*coccidia	1	2	171934.7	271010.9	40
9027	line*coccidia	1	3	264485.8	271010.9	40
9027	line*coccidia	2	0	87231.33	271010.9	40
9027	line*coccidia	2	1	35946.33	271010.9	40
9027	line*coccidia	2	2	190338.3	271010.9	40
9027	line*coccidia	2	3	40276.5	271010.9	40
9028	line	1		2848817	397266.2	40
9028	line	2		3039055	397266.2	40
9028	coccidia		0	3645525	561819.2	40
9028	coccidia		1	1968141	561819.2	40
9028	coccidia		2	1992141	561819.2	40
9028	coccidia		3	4169936	561819.2	40
9028	line*coccidia	1	0	3067632	794532.4	40
9028	line*coccidia	1	1	1080914	794532.4	40
9028	line*coccidia	1	2	1714519	794532.4	40
9028	line*coccidia	1	3	5532202	794532.4	40
9028	line*coccidia	2	0	4223419	794532.4	40
9028	line*coccidia	2	1	2855368	794532.4	40
9028	line*coccidia	2	2	2269763	794532.4	40
9028	line*coccidia	2	3	2807671	794532.4	40
9034	line	1		364693.6	96314.06	40
9034	line	2		2.63E-11	96314.06	40
9034	coccidia		0	2.91E-11	136208.7	40
9034	coccidia		1	729387.2	136208.7	40
9034	coccidia		2	5.82E-11	136208.7	40
9034	coccidia		3	5.82E-11	136208.7	40
9034	line*coccidia	1	0	2.91E-11	192628.1	40
9034	line*coccidia	1	1	1458774	192628.1	40
9034	line*coccidia	1	2	5.23E-11	192628.1	40
9034	line*coccidia	1	3	5.23E-11	192628.1	40
9034	line*coccidia	2	0	2.91E-11	192628.1	40
9034	line*coccidia	2	1	-5.2E-11	192628.1	40
9034	line*coccidia	2	2	6.41E-11	192628.1	40
9034	line*coccidia	2	3	6.41E-11	192628.1	40
9035	line	1		96546.17	23689.2	40
9035	line	2		9.58E-13	23689.2	40

9035	coccidia		0	0	33501.59	40
9035	coccidia		1	193092.3	33501.59	40
9035	coccidia		2	0	33501.59	40
9035	coccidia		3	0	33501.59	40
9035	line*coccidia	1	0	0	47378.41	40
9035	line*coccidia	1	1	386184.7	47378.41	40
9035	line*coccidia	1	2	8.42E-12	47378.41	40
9035	line*coccidia	1	3	8.42E-12	47378.41	40
9035	line*coccidia	2	0	0	47378.41	40
9035	line*coccidia	2	1	2.07E-11	47378.41	40
9035	line*coccidia	2	2	-8.4E-12	47378.41	40
9035	line*coccidia	2	3	-8.4E-12	47378.41	40
9104	line	1		214266.9	38995.24	40
9104	line	2		209536	38995.24	40
9104	coccidia		0	293129.2	55147.6	40
9104	coccidia		1	83196.25	55147.6	40
9104	coccidia		2	169401.1	55147.6	40
9104	coccidia		3	301879.3	55147.6	40
9104	line*coccidia	1	0	208386.7	77990.48	40
9104	line*coccidia	1	1	71676.17	77990.48	40
9104	line*coccidia	1	2	135236.3	77990.48	40
9104	line*coccidia	1	3	441768.5	77990.48	40
9104	line*coccidia	2	0	377871.7	77990.48	40
9104	line*coccidia	2	1	94716.33	77990.48	40
9104	line*coccidia	2	2	203565.8	77990.48	40
9104	line*coccidia	2	3	161990	77990.48	40
9105	line	1		659218.2	70009.89	40
9105	line	2		682375	70009.89	40
9105	coccidia		0	913207.3	99008.93	40
9105	coccidia		1	286308.5	99008.93	40
9105	coccidia		2	687591.7	99008.93	40
9105	coccidia		3	796078.8	99008.93	40
9105	line*coccidia	1	0	827297	140019.8	40
9105	line*coccidia	1	1	206640.3	140019.8	40
9105	line*coccidia	1	2	513535.3	140019.8	40
9105	line*coccidia	1	3	1089400	140019.8	40
9105	line*coccidia	2	0	999117.7	140019.8	40
9105	line*coccidia	2	1	365976.7	140019.8	40
9105	line*coccidia	2	2	861648	140019.8	40
9105	line*coccidia	2	3	502757.7	140019.8	40
9106	line	1		12923905	583233.5	40
9106	line	2		9326496	583233.5	40
9106	coccidia		0	16185487	824816.7	40
9106	coccidia		1	6282094	824816.7	40
9106	coccidia		2	6992844	824816.7	40
9106	coccidia		3	15040378	824816.7	40
9106	line*coccidia	1	0	20259304	1166467	40
9106	line*coccidia	1	1	3632124	1166467	40
9106	line*coccidia	1	2	4133322	1166467	40

9106	line*coccidia	1	3	23670871	1166467	40
9106	line*coccidia	2	0	12111670	1166467	40
9106	line*coccidia	2	1	8932064	1166467	40
9106	line*coccidia	2	2	9852367	1166467	40
9106	line*coccidia	2	3	6409885	1166467	40
9107	line	1		307850.7	28617.35	40
9107	line	2		298619.2	28617.35	40
9107	coccidia		0	290211.4	40471.04	40
9107	coccidia		1	109512.2	40471.04	40
9107	coccidia		2	312537.6	40471.04	40
9107	coccidia		3	500678.6	40471.04	40
9107	line*coccidia	1	0	294893.2	57234.7	40
9107	line*coccidia	1	1	87176	57234.7	40
9107	line*coccidia	1	2	185461.8	57234.7	40
9107	line*coccidia	1	3	663871.7	57234.7	40
9107	line*coccidia	2	0	285529.7	57234.7	40
9107	line*coccidia	2	1	131848.3	57234.7	40
9107	line*coccidia	2	2	439613.3	57234.7	40
9107	line*coccidia	2	3	337485.5	57234.7	40
9203	line	1		690746	117152.6	40
9203	line	2		631414.5	117152.6	40
9203	coccidia		0	844580.4	165678.8	40
9203	coccidia		1	484017.3	165678.8	40
9203	coccidia		2	617911.9	165678.8	40
9203	coccidia		3	697811.3	165678.8	40
9203	line*coccidia	1	0	961744.8	234305.2	40
9203	line*coccidia	1	1	469182.3	234305.2	40
9203	line*coccidia	1	2	363530.7	234305.2	40
9203	line*coccidia	1	3	968526.2	234305.2	40
9203	line*coccidia	2	0	727416	234305.2	40
9203	line*coccidia	2	1	498852.3	234305.2	40
9203	line*coccidia	2	2	872293.2	234305.2	40
9203	line*coccidia	2	3	427096.3	234305.2	40
9204	line	1		586951.8	59005	40
9204	line	2		480597.2	59005	40
9204	coccidia		0	655218.9	83445.67	40
9204	coccidia		1	255063.8	83445.67	40
9204	coccidia		2	420061.7	83445.67	40
9204	coccidia		3	804753.5	83445.67	40
9204	line*coccidia	1	0	665003.8	118010	40
9204	line*coccidia	1	1	235181.7	118010	40
9204	line*coccidia	1	2	361382.5	118010	40
9204	line*coccidia	1	3	1086239	118010	40
9204	line*coccidia	2	0	645434	118010	40
9204	line*coccidia	2	1	274945.8	118010	40
9204	line*coccidia	2	2	478740.8	118010	40
9204	line*coccidia	2	3	523268	118010	40
9206	line	1		334972.8	49454.52	40
9206	line	2		393737.3	49454.52	40

9206	coccidia		0	418682.8	69939.25	40
9206	coccidia		1	210254.7	69939.25	40
9206	coccidia		2	316791.4	69939.25	40
9206	coccidia		3	511691.1	69939.25	40
9206	line*coccidia	1	0	438630.5	98909.04	40
9206	line*coccidia	1	1	142003.7	98909.04	40
9206	line*coccidia	1	2	219468.2	98909.04	40
9206	line*coccidia	1	3	539788.7	98909.04	40
9206	line*coccidia	2	0	398735.2	98909.04	40
9206	line*coccidia	2	1	278505.7	98909.04	40
9206	line*coccidia	2	2	414114.7	98909.04	40
9206	line*coccidia	2	3	483593.5	98909.04	40
9207	line	1		150376.9	37272.22	40
9207	line	2		270464.4	37272.22	40
9207	coccidia		0	297957.8	52710.88	40
9207	coccidia		1	163776.9	52710.88	40
9207	coccidia		2	194987.5	52710.88	40
9207	coccidia		3	184960.4	52710.88	40
9207	line*coccidia	1	0	223787	74544.44	40
9207	line*coccidia	1	1	73100.67	74544.44	40
9207	line*coccidia	1	2	90570.83	74544.44	40
9207	line*coccidia	1	3	214049.2	74544.44	40
9207	line*coccidia	2	0	372128.5	74544.44	40
9207	line*coccidia	2	1	254453.2	74544.44	40
9207	line*coccidia	2	2	299404.2	74544.44	40
9207	line*coccidia	2	3	155871.7	74544.44	40
9304	line	1		1395813	150804.4	40
9304	line	2		838776.9	150804.4	40
9304	coccidia		0	979646.2	213269.6	40
9304	coccidia		1	946628.5	213269.6	40
9304	coccidia		2	598427.9	213269.6	40
9304	coccidia		3	1944476	213269.6	40
9304	line*coccidia	1	0	1200262	301608.8	40
9304	line*coccidia	1	1	479788	301608.8	40
9304	line*coccidia	1	2	688196.7	301608.8	40
9304	line*coccidia	1	3	3215004	301608.8	40
9304	line*coccidia	2	0	759030.8	301608.8	40
9304	line*coccidia	2	1	1413469	301608.8	40
9304	line*coccidia	2	2	508659.2	301608.8	40
9304	line*coccidia	2	3	673948.5	301608.8	40
9305	line	1		1335810	155500.3	40
9305	line	2		1577372	155500.3	40
9305	coccidia		0	1945221	219910.7	40
9305	coccidia		1	1000005	219910.7	40
9305	coccidia		2	1186941	219910.7	40
9305	coccidia		3	1694197	219910.7	40
9305	line*coccidia	1	0	1810972	311000.7	40
9305	line*coccidia	1	1	636017.7	311000.7	40
9305	line*coccidia	1	2	686797.8	311000.7	40

9305	line*coccidia	1	3	2209452	311000.7	40
9305	line*coccidia	2	0	2079471	311000.7	40
9305	line*coccidia	2	1	1363992	311000.7	40
9305	line*coccidia	2	2	1687083	311000.7	40
9305	line*coccidia	2	3	1178942	311000.7	40
9307	line	1		3935951	543037.1	40
9307	line	2		6139743	543037.1	40
9307	coccidia		0	6428481	767970.4	40
9307	coccidia		1	5024921	767970.4	40
9307	coccidia		2	2968013	767970.4	40
9307	coccidia		3	5729975	767970.4	40
9307	line*coccidia	1	0	4209502	1086074	40
9307	line*coccidia	1	1	1846133	1086074	40
9307	line*coccidia	1	2	1848208	1086074	40
9307	line*coccidia	1	3	7839963	1086074	40
9307	line*coccidia	2	0	8647460	1086074	40
9307	line*coccidia	2	1	8203709	1086074	40
9307	line*coccidia	2	2	4087817	1086074	40
9307	line*coccidia	2	3	3619987	1086074	40
9310	line	1		1803351	272938.7	40
9310	line	2		2460422	272938.7	40
9310	coccidia		0	2860919	385993.7	40
9310	coccidia		1	1467854	385993.7	40
9310	coccidia		2	1465076	385993.7	40
9310	coccidia		3	2733697	385993.7	40
9310	line*coccidia	1	0	2260216	545877.5	40
9310	line*coccidia	1	1	1272705	545877.5	40
9310	line*coccidia	1	2	1143802	545877.5	40
9310	line*coccidia	1	3	2536683	545877.5	40
9310	line*coccidia	2	0	3461621	545877.5	40
9310	line*coccidia	2	1	1663003	545877.5	40
9310	line*coccidia	2	2	1786351	545877.5	40
9310	line*coccidia	2	3	2930712	545877.5	40
9311	line	1		678367.5	156222.9	40
9311	line	2		158511.6	156222.9	40
9311	coccidia		0	136841.3	220932.5	40
9311	coccidia		1	1270182	220932.5	40
9311	coccidia		2	113659.9	220932.5	40
9311	coccidia		3	153075.4	220932.5	40
9311	line*coccidia	1	0	108103.5	312445.8	40
9311	line*coccidia	1	1	2289796	312445.8	40
9311	line*coccidia	1	2	124138.3	312445.8	40
9311	line*coccidia	1	3	191432.5	312445.8	40
9311	line*coccidia	2	0	165579	312445.8	40
9311	line*coccidia	2	1	250567.7	312445.8	40
9311	line*coccidia	2	2	103181.5	312445.8	40
9311	line*coccidia	2	3	114718.3	312445.8	40
9404	line	1		3375730	253426.4	40
9404	line	2		2983446	253426.4	40

9404	coccidia		0	4466566	358399	40
9404	coccidia		1	2667686	358399	40
9404	coccidia		2	2477314	358399	40
9404	coccidia		3	3106787	358399	40
9404	line*coccidia	1	0	4886237	506852.8	40
9404	line*coccidia	1	1	2262402	506852.8	40
9404	line*coccidia	1	2	2083396	506852.8	40
9404	line*coccidia	1	3	4270885	506852.8	40
9404	line*coccidia	2	0	4046894	506852.8	40
9404	line*coccidia	2	1	3072969	506852.8	40
9404	line*coccidia	2	2	2871232	506852.8	40
9404	line*coccidia	2	3	1942688	506852.8	40
9405	line	1		1276321	258662	40
9405	line	2		1138221	258662	40
9405	coccidia		0	815877.2	365803.3	40
9405	coccidia		1	668328.8	365803.3	40
9405	coccidia		2	681250	365803.3	40
9405	coccidia		3	2663628	365803.3	40
9405	line*coccidia	1	0	1045403	517324	40
9405	line*coccidia	1	1	682614.2	517324	40
9405	line*coccidia	1	2	772499.5	517324	40
9405	line*coccidia	1	3	2604768	517324	40
9405	line*coccidia	2	0	586351	517324	40
9405	line*coccidia	2	1	654043.3	517324	40
9405	line*coccidia	2	2	590000.5	517324	40
9405	line*coccidia	2	3	2722488	517324	40
9406	line	1		6170633	784637.8	40
9406	line	2		6834847	784637.8	40
9406	coccidia		0	5829822	1109645	40
9406	coccidia		1	6184634	1109645	40
9406	coccidia		2	3992022	1109645	40
9406	coccidia		3	10004482	1109645	40
9406	line*coccidia	1	0	5577146	1569276	40
9406	line*coccidia	1	1	8299665	1569276	40
9406	line*coccidia	1	2	3470649	1569276	40
9406	line*coccidia	1	3	7335071	1569276	40
9406	line*coccidia	2	0	6082499	1569276	40
9406	line*coccidia	2	1	4069603	1569276	40
9406	line*coccidia	2	2	4513395	1569276	40
9406	line*coccidia	2	3	12673893	1569276	40
9407	line	1		1914464	219758.9	40
9407	line	2		2113192	219758.9	40
9407	coccidia		0	2907022	310786	40
9407	coccidia		1	1664456	310786	40
9407	coccidia		2	1628711	310786	40
9407	coccidia		3	1855122	310786	40
9407	line*coccidia	1	0	2137560	439517.8	40
9407	line*coccidia	1	1	1334823	439517.8	40
9407	line*coccidia	1	2	1002808	439517.8	40

9407	line*coccidia	1	3	3182665	439517.8	40
9407	line*coccidia	2	0	3676484	439517.8	40
9407	line*coccidia	2	1	1994089	439517.8	40
9407	line*coccidia	2	2	2254615	439517.8	40
9407	line*coccidia	2	3	527579.3	439517.8	40
9410	line	1		13730221	2150602	40
9410	line	2		14659606	2150602	40
9410	coccidia		0	18067708	3041410	40
9410	coccidia		1	20563880	3041410	40
9410	coccidia		2	7584995	3041410	40
9410	coccidia		3	10563073	3041410	40
9410	line*coccidia	1	0	14074721	4301203	40
9410	line*coccidia	1	1	24805519	4301203	40
9410	line*coccidia	1	2	6925089	4301203	40
9410	line*coccidia	1	3	9115556	4301203	40
9410	line*coccidia	2	0	22060696	4301203	40
9410	line*coccidia	2	1	16322241	4301203	40
9410	line*coccidia	2	2	8244900	4301203	40
9410	line*coccidia	2	3	12010590	4301203	40
9412	line	1		40668.13	35143.32	40
9412	line	2		200270.9	35143.32	40
9412	coccidia		0	236753.3	49700.16	40
9412	coccidia		1	69498.17	49700.16	40
9412	coccidia		2	95004.58	49700.16	40
9412	coccidia		3	80622	49700.16	40
9412	line*coccidia	1	0	115026.5	70286.64	40
9412	line*coccidia	1	1	47646	70286.64	40
9412	line*coccidia	1	2	0	70286.64	40
9412	line*coccidia	1	3	0	70286.64	40
9412	line*coccidia	2	0	358480	70286.64	40
9412	line*coccidia	2	1	91350.33	70286.64	40
9412	line*coccidia	2	2	190009.2	70286.64	40
9412	line*coccidia	2	3	161244	70286.64	40
9506	line	1		1055801	156383.9	40
9506	line	2		1134311	156383.9	40
9506	coccidia		0	1002415	221160.3	40
9506	coccidia		1	1267451	221160.3	40
9506	coccidia		2	775313.6	221160.3	40
9506	coccidia		3	1335044	221160.3	40
9506	line*coccidia	1	0	1010381	312767.8	40
9506	line*coccidia	1	1	1264481	312767.8	40
9506	line*coccidia	1	2	404741	312767.8	40
9506	line*coccidia	1	3	1543601	312767.8	40
9506	line*coccidia	2	0	994449.2	312767.8	40
9506	line*coccidia	2	1	1270421	312767.8	40
9506	line*coccidia	2	2	1145886	312767.8	40
9506	line*coccidia	2	3	1126486	312767.8	40
9508	line	1		58930853	5426753	40
9508	line	2		90014320	5426753	40

9508	coccidia		0	1.13E+08	7674588	40
9508	coccidia		1	45367713	7674588	40
9508	coccidia		2	67842841	7674588	40
9508	coccidia		3	71895825	7674588	40
9508	line*coccidia	1	0	84288244	10853506	40
9508	line*coccidia	1	1	20218010	10853506	40
9508	line*coccidia	1	2	40894630	10853506	40
9508	line*coccidia	1	3	90322529	10853506	40
9508	line*coccidia	2	0	1.41E+08	10853506	40
9508	line*coccidia	2	1	70517417	10853506	40
9508	line*coccidia	2	2	94791053	10853506	40
9508	line*coccidia	2	3	53469121	10853506	40
9512	line	1		734218.9	352210.3	40
9512	line	2		1450276	352210.3	40
9512	coccidia		0	2441454	498100.6	40
9512	coccidia		1	344813.3	498100.6	40
9512	coccidia		2	1072528	498100.6	40
9512	coccidia		3	510194.7	498100.6	40
9512	line*coccidia	1	0	1806059	704420.7	40
9512	line*coccidia	1	1	144796.3	704420.7	40
9512	line*coccidia	1	2	276871.5	704420.7	40
9512	line*coccidia	1	3	709148.7	704420.7	40
9512	line*coccidia	2	0	3076849	704420.7	40
9512	line*coccidia	2	1	544830.2	704420.7	40
9512	line*coccidia	2	2	1868185	704420.7	40
9512	line*coccidia	2	3	311240.7	704420.7	40
9515	line	1		3987598	1922414	40
9515	line	2		2099518	1922414	40
9515	coccidia		0	3199776	2718703	40
9515	coccidia		1	781851.3	2718703	40
9515	coccidia		2	7385107	2718703	40
9515	coccidia		3	807497.8	2718703	40
9515	line*coccidia	1	0	3032942	3844827	40
9515	line*coccidia	1	1	516290	3844827	40
9515	line*coccidia	1	2	11305131	3844827	40
9515	line*coccidia	1	3	1096029	3844827	40
9515	line*coccidia	2	0	3366609	3844827	40
9515	line*coccidia	2	1	1047413	3844827	40
9515	line*coccidia	2	2	3465083	3844827	40
9515	line*coccidia	2	3	518966.3	3844827	40
9521	line	1		509709.3	356111.1	40
9521	line	2		811860.2	356111.1	40
9521	coccidia		0	88976.83	503617.2	40
9521	coccidia		1	69010.17	503617.2	40
9521	coccidia		2	838173.2	503617.2	40
9521	coccidia		3	1646979	503617.2	40
9521	line*coccidia	1	0	92674.33	712222.3	40
9521	line*coccidia	1	1	12876	712222.3	40
9521	line*coccidia	1	2	1593771	712222.3	40

9521	line*coccidia	1	3	339515.8	712222.3	40
9521	line*coccidia	2	0	85279.33	712222.3	40
9521	line*coccidia	2	1	125144.3	712222.3	40
9521	line*coccidia	2	2	82575.17	712222.3	40
9521	line*coccidia	2	3	2954442	712222.3	40
9523	line	1		1053585	580060	40
9523	line	2		892016.9	580060	40
9523	coccidia		0	276458.8	820328.7	40
9523	coccidia		1	115911.4	820328.7	40
9523	coccidia		2	1895104	820328.7	40
9523	coccidia		3	1603730	820328.7	40
9523	line*coccidia	1	0	174127.7	1160120	40
9523	line*coccidia	1	1	80475.67	1160120	40
9523	line*coccidia	1	2	3700638	1160120	40
9523	line*coccidia	1	3	259098	1160120	40
9523	line*coccidia	2	0	378790	1160120	40
9523	line*coccidia	2	1	151347.2	1160120	40
9523	line*coccidia	2	2	89569.33	1160120	40
9523	line*coccidia	2	3	2948361	1160120	40
9601	line	1		1191795	125964.4	40
9601	line	2		1450490	125964.4	40
9601	coccidia		0	1775422	178140.6	40
9601	coccidia		1	683694.6	178140.6	40
9601	coccidia		2	1375685	178140.6	40
9601	coccidia		3	1449770	178140.6	40
9601	line*coccidia	1	0	1750530	251928.9	40
9601	line*coccidia	1	1	669268	251928.9	40
9601	line*coccidia	1	2	616752.7	251928.9	40
9601	line*coccidia	1	3	1730631	251928.9	40
9601	line*coccidia	2	0	1800314	251928.9	40
9601	line*coccidia	2	1	698121.2	251928.9	40
9601	line*coccidia	2	2	2134617	251928.9	40
9601	line*coccidia	2	3	1168909	251928.9	40
9602	line	1		2095598	182172.1	40
9602	line	2		2059119	182172.1	40
9602	coccidia		0	2440119	257630.3	40
9602	coccidia		1	1365138	257630.3	40
9602	coccidia		2	1895869	257630.3	40
9602	coccidia		3	2608309	257630.3	40
9602	line*coccidia	1	0	2214837	364344.2	40
9602	line*coccidia	1	1	1646123	364344.2	40
9602	line*coccidia	1	2	1480722	364344.2	40
9602	line*coccidia	1	3	3040708	364344.2	40
9602	line*coccidia	2	0	2665400	364344.2	40
9602	line*coccidia	2	1	1084152	364344.2	40
9602	line*coccidia	2	2	2311015	364344.2	40
9602	line*coccidia	2	3	2175909	364344.2	40
9603	line	1		3860813	286881	40
9603	line	2		4658978	286881	40

9603	coccidia		0	5050357	405711	40
9603	coccidia		1	2836742	405711	40
9603	coccidia		2	3587977	405711	40
9603	coccidia		3	5564505	405711	40
9603	line*coccidia	1	0	4119866	573762	40
9603	line*coccidia	1	1	2913345	573762	40
9603	line*coccidia	1	2	2471681	573762	40
9603	line*coccidia	1	3	5938358	573762	40
9603	line*coccidia	2	0	5980848	573762	40
9603	line*coccidia	2	1	2760138	573762	40
9603	line*coccidia	2	2	4704274	573762	40
9603	line*coccidia	2	3	5190652	573762	40
9607	line	1		1127104	114731.4	40
9607	line	2		1633316	114731.4	40
9607	coccidia		0	1481138	162254.7	40
9607	coccidia		1	1425646	162254.7	40
9607	coccidia		2	992503.8	162254.7	40
9607	coccidia		3	1621553	162254.7	40
9607	line*coccidia	1	0	1170808	229462.8	40
9607	line*coccidia	1	1	779174.8	229462.8	40
9607	line*coccidia	1	2	811163.2	229462.8	40
9607	line*coccidia	1	3	1747271	229462.8	40
9607	line*coccidia	2	0	1791467	229462.8	40
9607	line*coccidia	2	1	2072118	229462.8	40
9607	line*coccidia	2	2	1173845	229462.8	40
9607	line*coccidia	2	3	1495836	229462.8	40
9610	line	1		308875.3	64690.11	40
9610	line	2		9.09E-12	64690.11	40
9610	coccidia		0	1.46E-11	91485.64	40
9610	coccidia		1	520083.8	91485.64	40
9610	coccidia		2	1.46E-11	91485.64	40
9610	coccidia		3	97666.83	91485.64	40
9610	line*coccidia	1	0	1.46E-11	129380.2	40
9610	line*coccidia	1	1	1040168	129380.2	40
9610	line*coccidia	1	2	5.82E-11	129380.2	40
9610	line*coccidia	1	3	195333.7	129380.2	40
9610	line*coccidia	2	0	1.46E-11	129380.2	40
9610	line*coccidia	2	1	7.28E-12	129380.2	40
9610	line*coccidia	2	2	-2.9E-11	129380.2	40
9610	line*coccidia	2	3	4.37E-11	129380.2	40
9620	line	1		261006.9	99144.42	40
9620	line	2		387116.1	99144.42	40
9620	coccidia		0	625977.4	140211.4	40
9620	coccidia		1	161623.8	140211.4	40
9620	coccidia		2	326009.8	140211.4	40
9620	coccidia		3	182635	140211.4	40
9620	line*coccidia	1	0	356646.3	198288.8	40
9620	line*coccidia	1	1	38004.67	198288.8	40
9620	line*coccidia	1	2	482618.3	198288.8	40

9620	line*coccidia	1	3	166758.2	198288.8	40
9620	line*coccidia	2	0	895308.5	198288.8	40
9620	line*coccidia	2	1	285242.8	198288.8	40
9620	line*coccidia	2	2	169401.3	198288.8	40
9620	line*coccidia	2	3	198511.8	198288.8	40
9701	line	1		1094851	307140.4	40
9701	line	2		1233573	307140.4	40
9701	coccidia		0	2174701	434362.1	40
9701	coccidia		1	625734	434362.1	40
9701	coccidia		2	1295249	434362.1	40
9701	coccidia		3	561165.3	434362.1	40
9701	line*coccidia	1	0	2882312	614280.7	40
9701	line*coccidia	1	1	56504.33	614280.7	40
9701	line*coccidia	1	2	989405.7	614280.7	40
9701	line*coccidia	1	3	451182.7	614280.7	40
9701	line*coccidia	2	0	1467089	614280.7	40
9701	line*coccidia	2	1	1194964	614280.7	40
9701	line*coccidia	2	2	1601093	614280.7	40
9701	line*coccidia	2	3	671148	614280.7	40
9709	line	1		566740.4	97791	40
9709	line	2		1067091	97791	40
9709	coccidia		0	1004049	138297.4	40
9709	coccidia		1	420514.5	138297.4	40
9709	coccidia		2	784683.2	138297.4	40
9709	coccidia		3	1058415	138297.4	40
9709	line*coccidia	1	0	692481	195582	40
9709	line*coccidia	1	1	306632.5	195582	40
9709	line*coccidia	1	2	364997.3	195582	40
9709	line*coccidia	1	3	902850.8	195582	40
9709	line*coccidia	2	0	1315617	195582	40
9709	line*coccidia	2	1	534396.5	195582	40
9709	line*coccidia	2	2	1204369	195582	40
9709	line*coccidia	2	3	1213980	195582	40
9712	line	1		507044.7	206444.4	40
9712	line	2		984457.8	206444.4	40
9712	coccidia		0	1468535	291956.5	40
9712	coccidia		1	321120.4	291956.5	40
9712	coccidia		2	759532.4	291956.5	40
9712	coccidia		3	433816.9	291956.5	40
9712	line*coccidia	1	0	2028179	412888.9	40
9712	line*coccidia	1	1	0	412888.9	40
9712	line*coccidia	1	2	0	412888.9	40
9712	line*coccidia	1	3	0	412888.9	40
9712	line*coccidia	2	0	908891.8	412888.9	40
9712	line*coccidia	2	1	642240.8	412888.9	40
9712	line*coccidia	2	2	1519065	412888.9	40
9712	line*coccidia	2	3	867633.8	412888.9	40
9714	line	1		129194.3	45756.43	40
9714	line	2		277784.4	45756.43	40

9714	coccidia		0	393296.1	64709.36	40
9714	coccidia		1	249398.9	64709.36	40
9714	coccidia		2	118560.8	64709.36	40
9714	coccidia		3	52701.5	64709.36	40
9714	line*coccidia	1	0	516777.2	91512.85	40
9714	line*coccidia	1	1	-8.7E-11	91512.85	40
9714	line*coccidia	1	2	-2.9E-11	91512.85	40
9714	line*coccidia	1	3	-2.9E-11	91512.85	40
9714	line*coccidia	2	0	269815	91512.85	40
9714	line*coccidia	2	1	498797.8	91512.85	40
9714	line*coccidia	2	2	237121.7	91512.85	40
9714	line*coccidia	2	3	105403	91512.85	40
9718	line	1		1043870	126824.5	40
9718	line	2		1521142	126824.5	40
9718	coccidia		0	1486314	179356.9	40
9718	coccidia		1	1507436	179356.9	40
9718	coccidia		2	1205759	179356.9	40
9718	coccidia		3	930516.9	179356.9	40
9718	line*coccidia	1	0	1183378	253649	40
9718	line*coccidia	1	1	798457.8	253649	40
9718	line*coccidia	1	2	744388	253649	40
9718	line*coccidia	1	3	1449258	253649	40
9718	line*coccidia	2	0	1789250	253649	40
9718	line*coccidia	2	1	2216414	253649	40
9718	line*coccidia	2	2	1667129	253649	40
9718	line*coccidia	2	3	411776.3	253649	40
9719	line	1		701227.1	86663.33	40
9719	line	2		1093539	86663.33	40
9719	coccidia		0	706743.5	122560.5	40
9719	coccidia		1	1017198	122560.5	40
9719	coccidia		2	581456.6	122560.5	40
9719	coccidia		3	1284133	122560.5	40
9719	line*coccidia	1	0	541488.3	173326.7	40
9719	line*coccidia	1	1	440114.2	173326.7	40
9719	line*coccidia	1	2	488551.3	173326.7	40
9719	line*coccidia	1	3	1334755	173326.7	40
9719	line*coccidia	2	0	871998.7	173326.7	40
9719	line*coccidia	2	1	1594282	173326.7	40
9719	line*coccidia	2	2	674361.8	173326.7	40
9719	line*coccidia	2	3	1233512	173326.7	40
9801	line	1		216947.6	74675.88	40
9801	line	2		222826.1	74675.88	40
9801	coccidia		0	364600.8	105607.6	40
9801	coccidia		1	94517.67	105607.6	40
9801	coccidia		2	229435.3	105607.6	40
9801	coccidia		3	190993.8	105607.6	40
9801	line*coccidia	1	0	640019.5	149351.8	40
9801	line*coccidia	1	1	-1.5E-11	149351.8	40
9801	line*coccidia	1	2	165347.7	149351.8	40

9801	line*coccidia	1	3	62423.17	149351.8	40
9801	line*coccidia	2	0	89182	149351.8	40
9801	line*coccidia	2	1	189035.3	149351.8	40
9801	line*coccidia	2	2	293522.8	149351.8	40
9801	line*coccidia	2	3	319564.3	149351.8	40
9802	line	1		284492.3	106271.3	40
9802	line	2		285782	106271.3	40
9802	coccidia		0	672568.1	150290.3	40
9802	coccidia		1	106412.5	150290.3	40
9802	coccidia		2	168863.8	150290.3	40
9802	coccidia		3	192704.2	150290.3	40
9802	line*coccidia	1	0	906880	212542.6	40
9802	line*coccidia	1	1	1.46E-11	212542.6	40
9802	line*coccidia	1	2	131063.8	212542.6	40
9802	line*coccidia	1	3	100025.2	212542.6	40
9802	line*coccidia	2	0	438256.2	212542.6	40
9802	line*coccidia	2	1	212825	212542.6	40
9802	line*coccidia	2	2	206663.8	212542.6	40
9802	line*coccidia	2	3	285383.2	212542.6	40
9818	line	1		639949.3	85084.4	40
9818	line	2		1055539	85084.4	40
9818	coccidia		0	1250768	120327.5	40
9818	coccidia		1	663847.1	120327.5	40
9818	coccidia		2	807793.8	120327.5	40
9818	coccidia		3	668568.8	120327.5	40
9818	line*coccidia	1	0	854084.3	170168.8	40
9818	line*coccidia	1	1	544866	170168.8	40
9818	line*coccidia	1	2	362818.5	170168.8	40
9818	line*coccidia	1	3	798028.3	170168.8	40
9818	line*coccidia	2	0	1647451	170168.8	40
9818	line*coccidia	2	1	782828.2	170168.8	40
9818	line*coccidia	2	2	1252769	170168.8	40
9818	line*coccidia	2	3	539109.2	170168.8	40
9906	line	1		757172.3	113166.9	40
9906	line	2		969832.9	113166.9	40
9906	coccidia		0	1415846	160042.2	40
9906	coccidia		1	568680.2	160042.2	40
9906	coccidia		2	554253.3	160042.2	40
9906	coccidia		3	915230.7	160042.2	40
9906	line*coccidia	1	0	1038810	226333.9	40
9906	line*coccidia	1	1	581084.5	226333.9	40
9906	line*coccidia	1	2	418272.8	226333.9	40
9906	line*coccidia	1	3	990521.3	226333.9	40
9906	line*coccidia	2	0	1792882	226333.9	40
9906	line*coccidia	2	1	556275.8	226333.9	40
9906	line*coccidia	2	2	690233.8	226333.9	40
9906	line*coccidia	2	3	839940	226333.9	40
9909	line	1		1554356	163693.7	40
9909	line	2		2186660	163693.7	40

9909	coccidia		0	2623954	231497.8	40
9909	coccidia		1	1959611	231497.8	40
9909	coccidia		2	946715.9	231497.8	40
9909	coccidia		3	1951751	231497.8	40
9909	line*coccidia	1	0	2241411	327387.3	40
9909	line*coccidia	1	1	974957.2	327387.3	40
9909	line*coccidia	1	2	839941.2	327387.3	40
9909	line*coccidia	1	3	2161116	327387.3	40
9909	line*coccidia	2	0	3006498	327387.3	40
9909	line*coccidia	2	1	2944265	327387.3	40
9909	line*coccidia	2	2	1053491	327387.3	40
9909	line*coccidia	2	3	1742387	327387.3	40
9910	line	1		252232.5	107888.2	40
9910	line	2		298794.3	107888.2	40
9910	coccidia		0	670743	152576.9	40
9910	coccidia		1	67357.58	152576.9	40
9910	coccidia		2	196584.2	152576.9	40
9910	coccidia		3	167368.8	152576.9	40
9910	line*coccidia	1	0	723493.8	215776.4	40
9910	line*coccidia	1	1	2028	215776.4	40
9910	line*coccidia	1	2	185954.2	215776.4	40
9910	line*coccidia	1	3	97454	215776.4	40
9910	line*coccidia	2	0	617992.2	215776.4	40
9910	line*coccidia	2	1	132687.2	215776.4	40
9910	line*coccidia	2	2	207214.2	215776.4	40
9910	line*coccidia	2	3	237283.5	215776.4	40
9911	line	1		192921.1	64721.34	40
9911	line	2		256737.1	64721.34	40
9911	coccidia		0	365327	91529.8	40
9911	coccidia		1	58361.92	91529.8	40
9911	coccidia		2	150498	91529.8	40
9911	coccidia		3	325129.5	91529.8	40
9911	line*coccidia	1	0	482469.2	129442.7	40
9911	line*coccidia	1	1	0	129442.7	40
9911	line*coccidia	1	2	154053.8	129442.7	40
9911	line*coccidia	1	3	135161.3	129442.7	40
9911	line*coccidia	2	0	248184.8	129442.7	40
9911	line*coccidia	2	1	116723.8	129442.7	40
9911	line*coccidia	2	2	146942.2	129442.7	40
9911	line*coccidia	2	3	515097.7	129442.7	40

[1]Broiler genetic line: 1= Line A, 2= Line B

[2]*Eimeria* infection treatment: 1= control, 2= *E. acervulina*, 3= *E. maxima*, 4= *E. tenella*

[3]Estimate: LS means for main effects and interaction

[4]StdErr: Pooled standard error

[5]DF= Degrees of freedom

1301860	697723	557024.9	786662.8
